# A Dynamic Thermodynamic Resolution Strategy for the Stereocontrolled Synthesis of Streptonigrin

**DOI:** 10.1002/anie.202213692

**Published:** 2022-12-07

**Authors:** Luis F. Valdez Pérez, Sylvestre P. J. T. Bachollet, Nikolai V. Orlov, Kenji P. M. Kopf, Joseph P. A. Harrity

**Affiliations:** ^1^ Department of Chemistry The University of Sheffield Sheffield S3 7HF UK

**Keywords:** Atropisomers, Boronic Esters, Diastereoselectivity, Dynamic Thermodynamic Resolution, Total Synthesis

## Abstract

We report that axially chiral biaryl boronic esters can be generated with control of atroposelectivity by a Binol‐mediated dynamic thermodynamic resolution process. These intermediates can be progressed to enantioenriched products through stereoretentive functionalization of the carbon–boron bond. Finally, we have exploited this method in the first highly stereoselective total synthesis of *P*‐streptonigrin.

## Graphical abstract

The long‐standing challenge of preparing *P*‐streptonigrin with high levels of enantioenrichment has been addressed by the use of an atroposelective dynamic thermodynamic resolution process. The strategy highlights the versatility of organoboron chemistry, as it plays a central role in ring synthesis, atropisomer resolution and functional group installation.

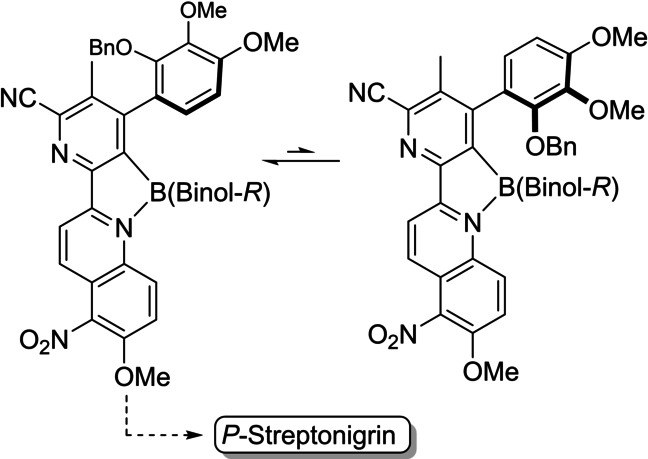

The discovery and synthesis of natural products that contain axial chirality has attracted the attention of the synthetic organic chemistry community, not only because of their promising properties, but also because of the intellectual and practical challenges that their syntheses represent.^[1]^ In this context, streptonigrin **1** is a natural product first isolated by Rao and Cullen from *Streptomyces flocculus* that has been the focus of significant synthetic and biological studies.[^2^, ^3^] Indeed, streptonigrin has recently been identified as a potential epigenetic cancer therapy treatment due to its ability to enhance heterochromatin formation at nanomolar concentrations.^[4]^

Streptonigrin (Figure 1) is composed of a densely functionalized quinoline moiety (A, B rings) connected to an atropisomeric heterobiaryl (C, B rings) system which is a deviation from more common chiral natural products that are composed of stereogenic centers.^[5]^ This latter feature poses particular challenges as the synthetic route must avoid undesired atropisomerizaton that may erode the atropo‐stereointegrity of any intermediates or the final product. The *M*‐stereochemistry of **1** has been assigned on the basis of experimental and theoretical circular dichroism studies.^[6]^

**Figure 1:**
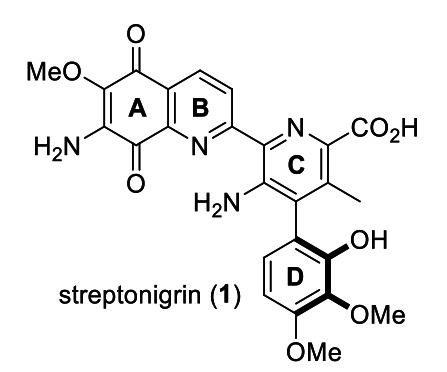
Structure of *M*‐streptonigrin **1**.

Several synthetic studies towards streptonigrin have been reported. In 1980, Weinreb devised a 31 step route where the key pyridine C ring was constructed by an imino Diels–Alder reaction.^[7]^ A year later, Kende's group reported a 19 step route whereby the C ring was assembled by a regioselective condensation of α‐ketoenamine with methyl acetoacetate,^[8]^ while Boger presented a more concise strategy employing an inverse‐electron‐demand Diels–Alder reaction of a heterocyclic azadiene, obtaining an advanced intermediate in Kende's route.^[9]^ More recently, Donohoe and co‐workers applied their de novo pyridine synthesis through ring closing metathesis along with cross‐coupling strategies in two different routes affording streptonigrin in 14 linear steps and 11 % overall yield.^[10]^ Their investigations highlighted an enantioselective Suzuki–Miyaura cross‐coupling that offered the first asymmetric approach to the synthesis of streptonigrin (up to 42 % ee). Herein we report a new synthetic approach for the enantioselective total synthesis of streptonigrin that takes advantage of a boron‐directed cycloaddition strategy developed in our group.^[11]^

As shown in Scheme 1, central to our route design was the synthesis of a difluoroboranyl pyridine intermediate **2** that we planned to access using a boron‐directed cycloaddition reaction of triazine **3** and potassium trifluoroborate salt **4**. This process would offer a convergent means for assembling the A–D ring array while installing functionality that would enable interconversion towards the natural product. We envisaged that employment of the directed cycloaddition strategy would also overcome the low reactivity and poor regiocontrol observed in Boger's classical inverse‐electron‐demand Diels–Alder method.^[9]^

**Scheme 1:**
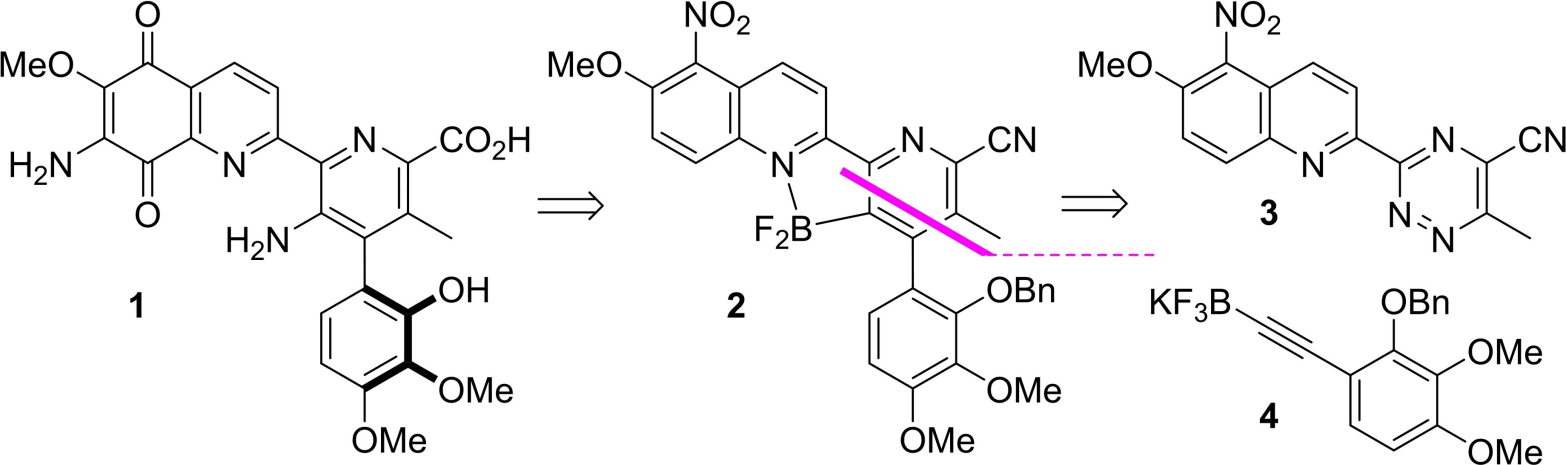
Directed cycloaddition strategy to the streptonigrin tetracyclic ring system.

Our synthesis commenced with the preparation of fragments **3** and **4**. 2‐Cyanoquinoline **5** was converted into triazinone **6**, whereupon successive deoxychlorination and displacement with cyanide provided a triazine intermediate that underwent nitration to provide **3** in 30 % overall yield. Synthesis of potassium trifluoroborate **4** consisted of a regioselective iodination of 2,3‐dimethoxyphenol **7** followed by protection of the free phenol with benzyl bromide to give **8**. Subsequent, Sonogashira cross‐coupling and alkyne borylation provided the key alkyne substrate **4** in 46 % yield over 5 steps. Finally, we were delighted to find that the directed cycloaddition reaction proceeded smoothly in the presence of BF_3_ ⋅ OEt_2_ within 1 hr at 30 °C to provide **2** with complete regiocontrol in 65 % yield (Scheme 2).

**Scheme 2:**
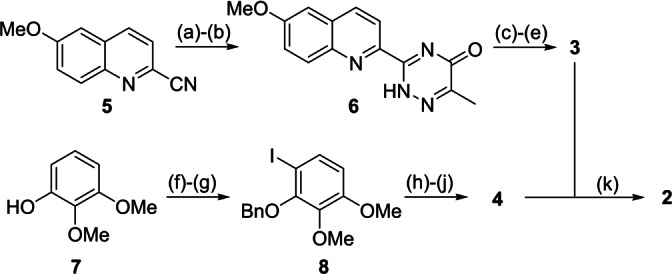
Reagents and conditions: a) N_2_H_4_.H_2_O, EtOH, rt, 95 %; b) MeCOCO_2_H, EtOH, reflux, 95 %; c) [ClC(H)N(Me)_2_]Cl, CH_2_Cl_2_, rt, 74 %; d) Zn(CN)_2_, Pd(PPh_3_)_4_, MeCN, 85 °C, 55 %; e) HNO_3_, H_2_SO_4_, 0 °C, 81 %; f) ICl, CH_2_Cl_2_, rt, 95 %; g) PhCH_2_Br, K_2_CO_3_, acetone, reflux, 80 %; h) Me_3_SiCCH, PdCl_2_(PPh_3_)_2_ (10 mol%), CuI (20 mol%), Et_3_N, 50 °C, 98 %; i) K_2_CO_3_, MeOH, rt, 72 %; j) BuLi, THF, −78 °C; B(OMe)_3_; KHF_2_ −20 °C to rt, 85 %; k) BF_3_.OEt_2_, CH_2_Cl_2_, 30 °C, 1 h, 65 %.

Evidence for atropisomerism in **2** was apparent from the ^19^F NMR spectrum that exhibited two AB doublets indicating the presence of diastereotopic F‐atoms (see the Supporting Information). With a view to separating the racemic mixture, we postulated that we could further exploit the presence of the boron moiety by generating a chiral boronate ester that could resolve the enantiomers by kinetic or classical resolution procedures. To test this hypothesis, we prepared model compounds **9 a**,**b** (synthesis details are in the Supporting Information) and converted these to the corresponding (*R*)‐1,1′‐bi‐2‐naphthol (Binol) esters. Running the esterification of **9 a** produced **10 a** in 69 % yield as an equal mixture of diastereomers. Surprisingly however, the corresponding reaction of **9 b** produced a 4 : 1 mixture of diastereomers **10 b** in an overall yield of 57 % (Scheme 3). Notably, the intermediate boronic acid was completely consumed in this reaction, ruling out the possibility that selective diastereomer formation was the result of a kinetic resolution process. In addition, the reaction could be conducted on 0.8 mmol scale allowing the product to be isolated as a single diastereomer in 66 % yield after slow precipitation from MeCN (see the Supporting Information).

**Scheme 3:**
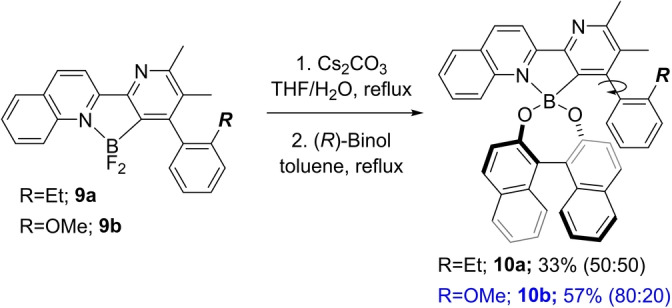
Model studies on resolution of atropisomeric boronates.

In order to better understand the generality of this transformation, we undertook a brief study of the scope of this resolution with respect to triazines and alkyne trifluoroborate salts; our results are summarized in Table 1. Persisting with (*R)‐*Binol as our resolving agent, we found that more hindered triazines (R^1^=Ph) showed similar trends in the resolution of *ortho*‐Et versus −OMe containing arylalkynes, with the latter showing modest levels of diastereocontrol (entries 1,2). Other Lewis basic substituents were explored and we found that bulkier ethers (entry 3) and amines (entry 4) provided marginally better diasteromeric ratios. Interestingly, the corresponding thioether **10 g** was formed as a single diastereomer (entry 5). Overall, a consistent picture emerged with *ortho‐*heteroatom groups providing approximately 80 : 20 diastereomeric ratios and *ortho*‐Et producing the corresponding Binol esters in equal amounts. This study confirmed that the incorporation of a Lewis basic substituent provided an opportunity to convert racemic biaryl difluoroboranes into diastereomeric boronic esters with stereocontrolled resolution at the biaryl bond.

**Table 1:**
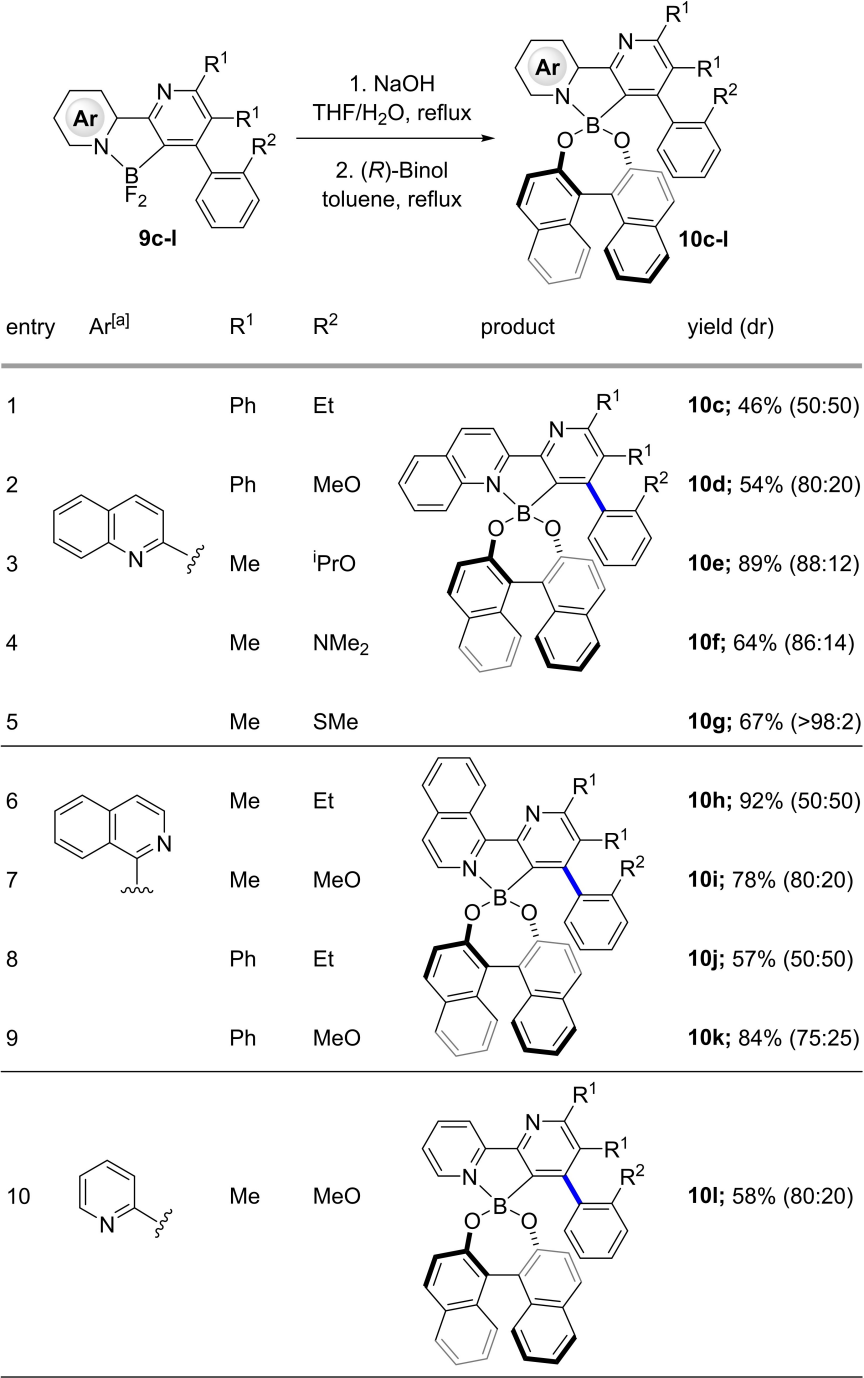
Scope of the resolution process.

[a] Compounds **9 e**, **9 f**, **9 g** were hydrolyzed with Cs_2_CO_3_ instead of NaOH.

We next undertook a study of the dynamic behavior of these systems upon heating. Towards this end, we were able to generate a diastereoenriched sample of **10 a** by chiral preparative HPLC (dr >98 : 2), and an enriched sample of **10 b** by precipitation (66 % yield, dr=95 : 5). Heating these samples in toluene at 120 °C overnight led to no change in diastereomeric ratio in the case of **10 a**. In contrast, however, **10 b** was returned as a 80 : 20 mixture of diastereomers (Scheme 4a). The observation that *only* those products bearing an *ortho*‐Lewis base form diastereomerically enriched boronic esters, together with the stereochemical lability of these compounds under thermolysis (in contrast to *ortho*‐ethyl analog **10 a**) highlights that the presence of an *ortho*‐heteroatom adjacent to the biaryl bond leads to a greater tendency for rotation around the biaryl bond, as compared to their simple hydrocarbon analogs. This in turn provides a pathway for a dynamic thermodynamic resolution process.[^12^, ^13^] A mechanism that is consistent with these observations is shown in Scheme 4b. Diastereomers **A** and **B** are able to interconvert via a planar intermediate that is formed when the Lewis basic atom ‘X′ coordinates to the boron center. This type of isomerization is reminiscent of the Pd‐promoted epimerization reported by Lassaletta and Stoltz which demonstrated that Lewis acid‐base complexation can promote planarization of atropisomers.^[14]^ Finally, X‐ray crystal structures of compounds **10 b**,**g** were obtained which highlight the potential for π‐stacking between Binol and one of the biaryl rings (Scheme 4c). We speculate that the minor diastereomer in these cases engenders an unfavourable steric interaction of the *ortho*‐substituent and the Binol ester oxygen atom. Furthermore, this analysis suggested that the incorporation of substituents at the 3,3′‐positions of Binol could influence diastereomer ratios, assuming that they did not impede atropisomerization. Indeed, preliminary results have highlighted that cyanomethyl‐substituted Binols **10 m**–**10 o** can offer improved diastereomeric ratios, and further studies relating to the generality of this effect are underway in our labs (Scheme 4d).

**Scheme 4:**
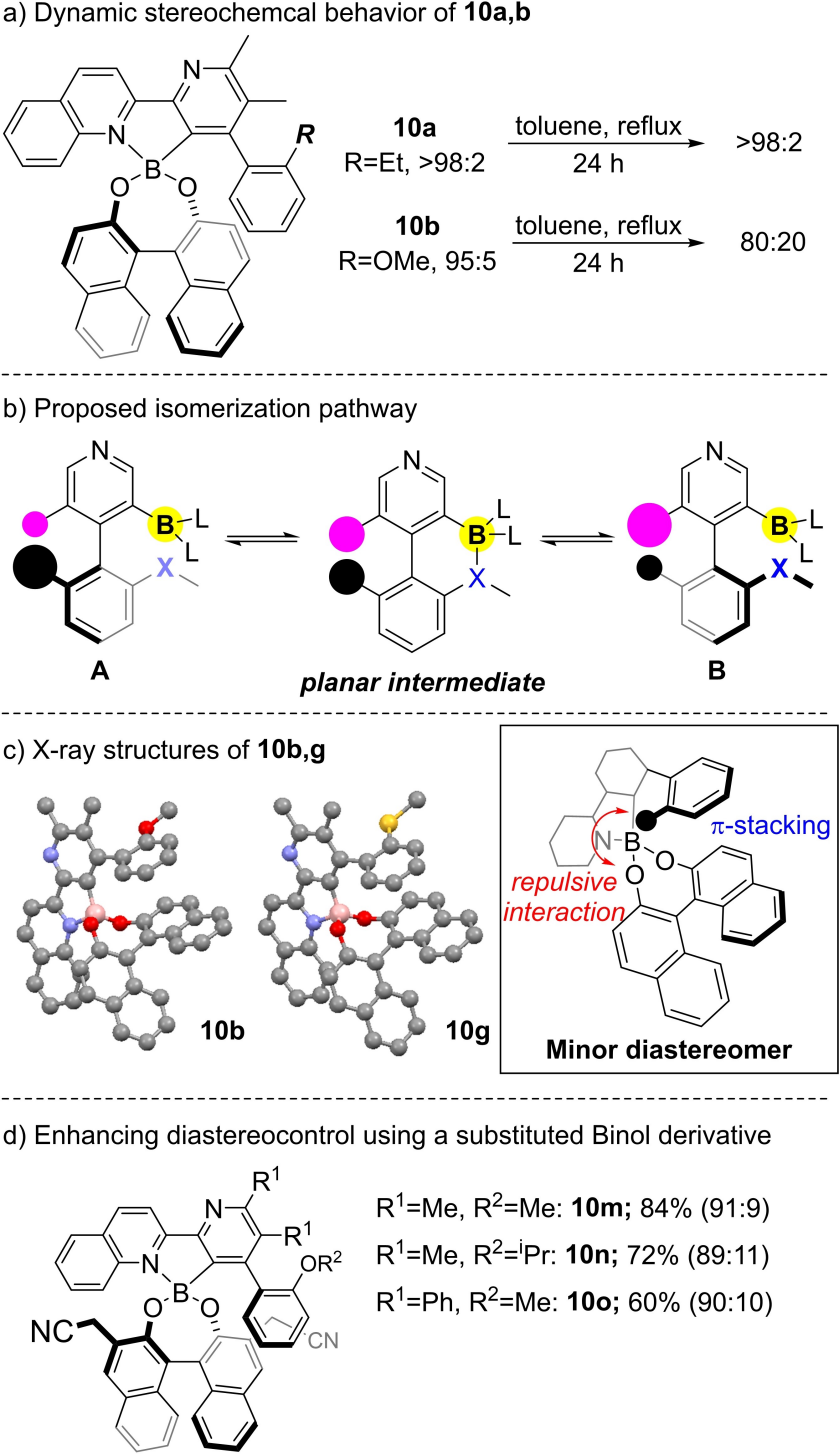
Dynamic behavior of diastereomeric atropisomers.

The stereochemical lability of the Binol esters raised an obvious concern regarding their suitability for stereoretentive functionalization reactions. Therefore, before continuing with our asymmetric synthesis of streptonigrin, we decided to explore the oxidation of boronates **10 b**,**e**,**g** to establish whether it was possible to generate new products with useful levels of enantiocontrol. As shown in Scheme 5, boron oxidation provided the corresponding phenols with high levels of stereoretention and the configuration of **11** was unambiguously confirmed by single crystal X‐ray diffraction analysis. Conversion of **10 g** to **13** showed more pronounced racemization and concomitant oxidation at sulfur.

**Scheme 5:**
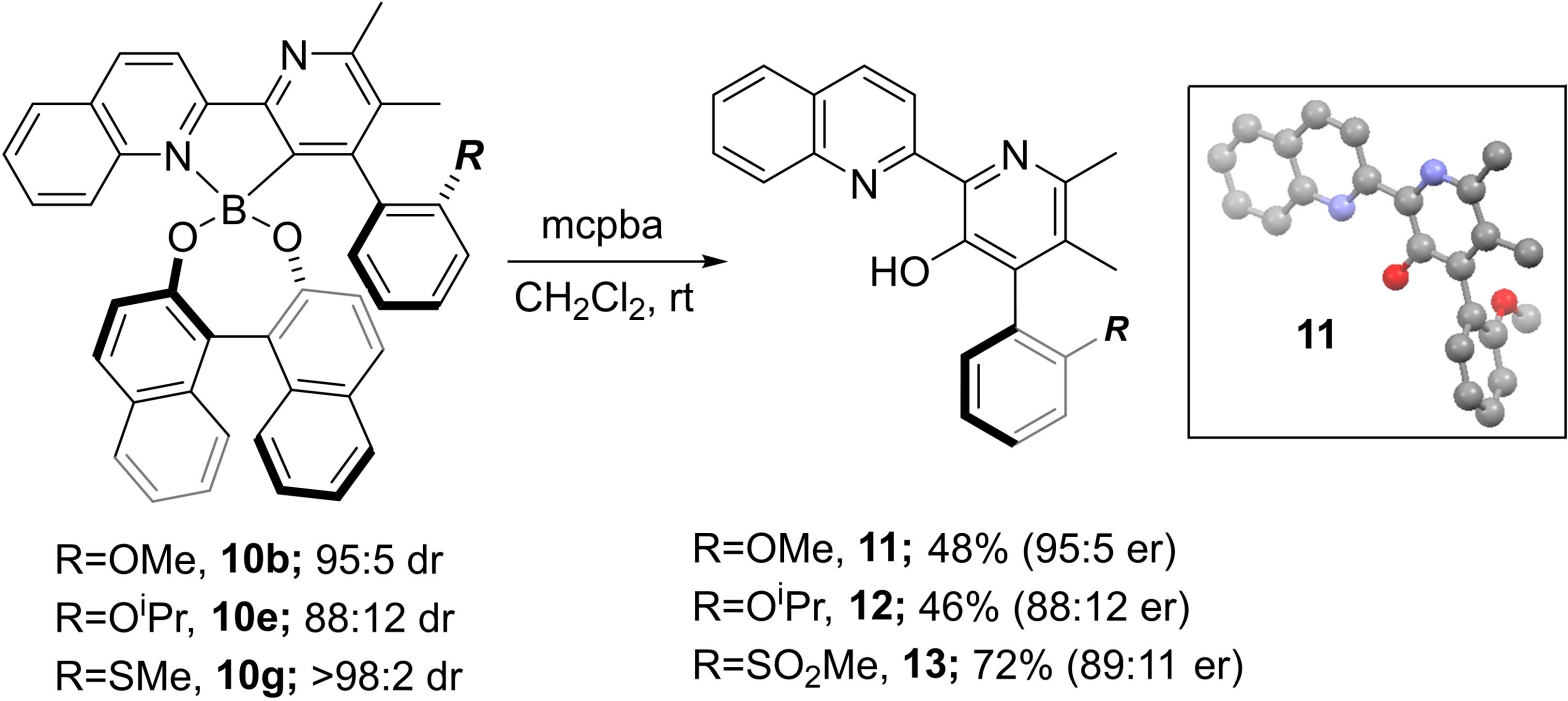
Stereoretentive oxidation reactions. mcpba: 3‐Chloroperbenzoic acid.

Having developed a protocol for the resolution of atropisomeric arylboranes, we continued with our synthesis towards streptonigrin (Scheme 6). To this end, difluoroboranyl pyridine **2** was subjected to hydrolysis, followed by esterification with (*R)‐*Binol to give boronic ester **14** in 56 % as a 86 : 14 mixture of diastereomers (major diastereomer assignment made by analogy to structures **10 b**,**g**). After column chromatography, the ratio could be improved up to 95 : 5, and the minor diastereomer recycled to an 86 : 14 ratio by simply refluxing the sample in toluene, thereby highlighting a practical advantage of the dynamic resolution approach. Finally, amination^[15]^ of diastereoenriched **14** afforded picolinitrile **15** in 50 % yield with a 92 : 8 e.r. confirming that this transformation could proceed with minimal erosion of stereochemical integrity around the biaryl bond.

**Scheme 6:**
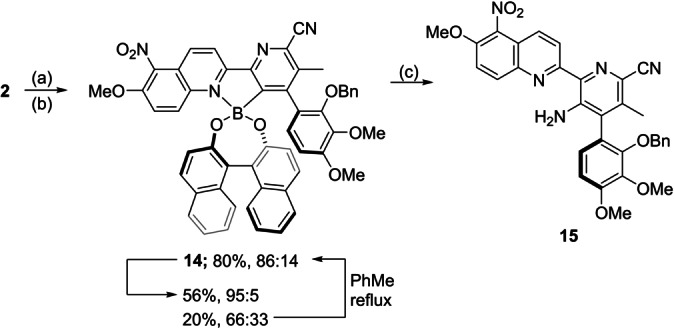
Reagents and conditions: a) NaOH (aq.), THF:CH_2_Cl_2_ 2 : 1, rt, 95 %; b) (*R)‐*Binol, PhMe, 120 °C, *ca*. 18 h, 56 %, 8 : 1 dr; c) NaN_3_, CuI, MeCN:MeOH 1 : 1, rt, 50 %, 92 : 8 e.r.

Our closing steps involved reduction of the cyano group of picolinitrile **15** to the corresponding picolinaldehyde with DIBAL‐H, followed by Pinnick oxidation and methylation with TMSCHN_2_ to obtain the corresponding methyl picolinate **16**. Then, the nitro group of **16** was reduced with Na_2_S_2_O_4_ and the resulting diamine was subjected to a two‐step oxidation protocol (Fremy's salt followed by aq. CAN) to yield quinolone intermediate **17** in 47 % overall yield over 6 steps with a minimal loss of the stereochemical information (**17**; 90 : 10 e.r.). Notably, these steps could be done sequentially and only the last step required chromatographic purification. The A‐ring amino group was introduced by a procedure successfully employed by Donohoe.^[10]^ Accordingly, intermediate **17** was treated with bromine and pyridine, followed by sodium azide, and finally subjected to hydrogenolysis to obtain streptonigrin methyl ester **18** in 55 % yield over 3 steps with a 89 : 11 e.r. Concomitantly, we acquired a commercial sample of streptonigrin and converted this to the corresponding methyl ester,^[16]^ thereby allowing us to confirm that **18** was the expected unnatural *P*‐atropisomer. Finally, hydrolysis of **18** with K_2_CO_3_ afforded streptonigrin with a 3.7 % overall yield over 18 linear steps (Scheme 7).^[17]^

**Scheme 7:**
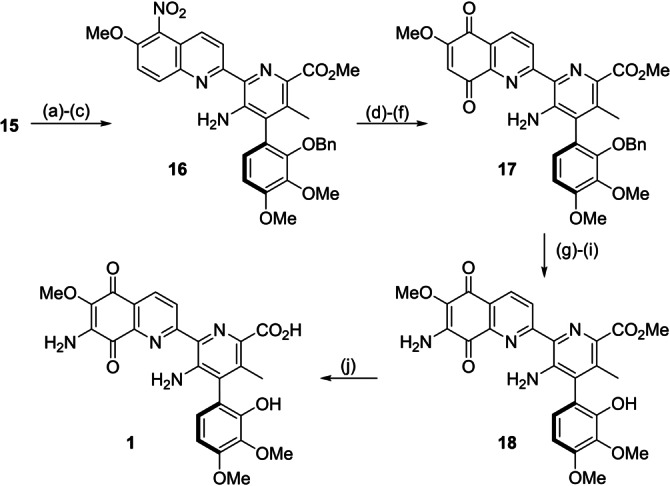
Reagents and conditions: a) DIBAL‐H, CH_2_Cl_2_, −78 °C; b) NaClO_2_, NaH_2_PO_4_⋅2 H_2_O, 2‐Me‐2‐butene, CH_2_Cl_2_:^*t*^BuOH:THF:H_2_O 2 : 1 : 1 : 1, rt; c) TMSCHN_2_, CH_2_Cl_2_:MeOH 1 : 1, 0 °C; d) Na_2_S_2_O_4_, THF:MeOH:H_2_O 2 : 1 : 1, 80 °C; e) Fremy's salt, Na_2_HPO_4_⋅2 H_2_O, acetone, r.t.; f) aq. CAN, MeCN, 0 °C to rt, 47 % over 6 steps, 90 : 10 e.r.; g) Br_2_, pyridine, CHCl_3_, rt; h) NaN_3_, DMF, 0 °C; i) H_2_, Pd/C, MeOH;EtOAc 3 : 1, rt, 55 % over 3 steps, 89 : 11 e.r.; j) K_2_CO_3_, MeOH:H_2_O 2 : 1, rt, 65 %.

In conclusion, we have demonstrated the versatility of organoboron chemistry for the stereocontrolled synthesis of atropisomeric biaryl compounds. Specifically, alkynylboranes allow the mild and regiocontrolled construction of highly functionalized aromatic products, and these can be resolved to deliver diastereoenriched boronate esters through a novel dynamic resolution process. Additionally, the versatility of the C−B bond allows new functionality to be introduced with stereoretention, providing a new way to access useful products in enantiomerically pure form. Finally, we have exploited this strategy for the first highly stereocontrolled synthesis of streptonigrin, thereby addressing a long‐standing synthetic challenge associated with this natural product.

## Conflict of interest

The authors declare no conflict of interest.

## Supporting information

As a service to our authors and readers, this journal provides supporting information supplied by the authors. Such materials are peer reviewed and may be re‐organized for online delivery, but are not copy‐edited or typeset. Technical support issues arising from supporting information (other than missing files) should be addressed to the authors.

Supporting Information

## Data Availability

The data that support the findings of this study are available in the supplementary material of this article.
