# Supplementary material for: A Dynamic Thermodynamic Resolution Strategy for the Stereocontrolled Synthesis of Streptonigrin
Source: Angew Chem Int Ed Engl. 2022 Dec 7;62(5):e202213692. doi: 10.1002/anie.202213692 (PMC10107650; doi:10.1002/anie.202213692)
Supplement: Supplementary file 1 — Supporting Information [file ANIE-62-0-s001.pdf]

Angewandte Chemie
Eine Zeitschrift der Gesellschaft Deutscher Chemiker

Supporting Information

A Dynamic Thermodynamic Resolution Strategy for the Stereocontrolled Synthesis of Streptonigrin

*L. F. Valdez Pérez, S. P. J. T. Bachollet, N. V. Orlov, K. P. M. Kopf, J. P. A. Harrity**

Contents

General Considerations S2

Synthesis of (R)-3,3'-Bis(cyanomethyl)-1,1'-binaphthyl-2,2'-diol (S6) S3

Synthesis of Difluoroboranes S5

General Procedures for the Synthesis of Boronic Esters S17

Total Synthesis of Streptonigrin S36

Comparison of ^{1}H and ^{13}C NMR Data for Natural and Synthetic Streptonigrin S57

NMR Spectra S58

Selected Crystallographic Data S138

Selected HPLC Data S188

References S196

General Considerations

All reactions were carried out in flame-dried glassware under high vacuum, unless stated otherwise. For reactions carried out under an inert atmosphere, solvents were purified using a PureSolv MD purification system and transferred under nitrogen. Infrared (IR) spectra were recorded on a Perkin Elmer Paragon FTIR spectrometer (ν_{max}/cm^{-1}). Samples were recorded neat as thin films. ^{1}H NMR spectra were recorded on a Bruker AVIII HD 400 (400 MHz), Bruker AVI 400 (400 MHz) or Bruker AMX400 (400 MHz). Chemical shifts are reported in parts per million (ppm) from tetramethylsilane, using the residual protic solvent resonance as the internal reference: ($CHCl_3$: δ 7.26 ppm, *d*^{6}–DMSO: δ 2.50 ppm, C_6D_6: δ 7.16 ppm) unless otherwise stated. Data are reported as follows: chemical shift, multiplicity (s = singlet, d = doublet, t = triplet, q = quartet, br = broad, m = multiplet), coupling constant (Hz), integration). ^{13}C NMR spectra were recorded on a Bruker AVIII HD 400 (101 MHz), Bruker AVI 400 (101 MHz) or Bruker AMX-400 (101 MHz) with broadband proton decoupling. Chemical shifts are reported in ppm from tetramethylsilane with the solvent as the internal reference ($CDCl_3$: δ 77.16 ppm, *d*^{6}–DMSO: δ 39.52 ppm, C_6D_6: δ 128.06 ppm). The signal of the carbon atom bonded to the boron atom is not observed due to quadrupolar relaxation broadening. ^{11}B NMR spectra were recorded on a Bruker AVIII HD 400 (128 MHz). ^{19}F NMR spectra were recorded on a Bruker AVIII HD 400 (128 MHz). High-resolution mass spectra (HRMS) recorded for accurate mass analysis, were performed on a Micromass LCT operating in electrospray mode (TOF, ESI$^+$, ESI$^-$). Thin layer chromatography (TLC) was performed on aluminium-backed plates pre-coated with silica (0.2 mm, Merck 60 F254) which were developed using standard visualizing agents: UV light or potassium permanganate. Flash chromatography was performed on silica gel (Merck 40-63 µm) or Florisil® (60-100 mesh). Melting points were recorded on Gallenkamp melting point apparatus and are uncorrected.

Synthesis of (*R*)-3,3'-Bis(cyanomethyl)-1,1'-binaphthyl-2,2'-diol (S6)

3,3'-Cyanomethyl Binol (**S6**) was synthesised according to the published procedures[1] without purification of the intermediate compounds (Scheme S1).

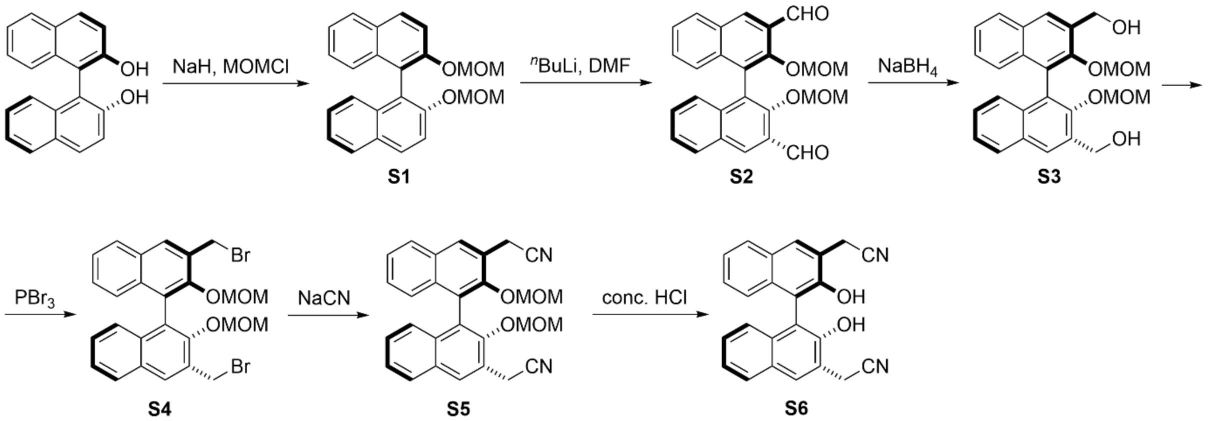

Scheme S1. Synthesis of (*R*)-3,3'-bis(cyanomethyl)-1,1'-binaphthyl-2,2'-diol (S6).

Following a literature procedure[1], (*R*)-(+)-1,1'-bi(2-naphtol) ((*R*)-BINOL, 1 g, 3.5 mmol, 1 equiv.), sodium hydride (NaH 60% dispersion in mineral oil, 252 mg, 10 mmol, 3 equiv.) and chloromethyl methyl ether (MOMCl, 0.58 mL, 7.7 mmol, 2.2 equiv.) were used to afford the crude product **S1**, which was purified by column chromatography on silica gel with a mixture of petrol:EtOAc 1:1 as eluent to afford the pure product as an amorphous white solid (1.085 g, 83%).

^{1}H NMR (400 MHz, $CDCl_3$): δ 7.96 (d, *J* = 9.0 Hz, 2H), 7.88 (d, *J* = 8.0 Hz, 2H), 7.59 (d, *J* = 9.0 Hz, 2H), 7.35 (t, *J* = 8 Hz, 2H), 7.26 – 7.15 (m, 4H), 5.09 (d, *J* = 7.0 Hz, 2H), 4.98 (d, *J* = 7.0 Hz, 2 H), 3.15 (s, 6H).

^{13}C NMR (101 MHz, $CDCl_3$): δ 153.6, 134.8, 130.2, 129.7, 128.7, 126.9, 126.0, 124.6, 121.8, 117.1, 95.2, 56.3.

These data were in accordance with the literature[1].

Following a literature procedure[1], **S1** (1 g, 2.7 mmol, 1 equiv.), a solution of ^{n}BuLi (2.5 M in hexanes, 3.3 mL, 8.3 mmol, 3.1 equiv.) and DMF (0.72 mL, 9.3 mmol, 3.5 equiv.) were used to afford the crude product **S2**, which was used in the next step without further purification.

[1] Y. Loewer, C. Weiss, A. T. Biju, R. Fröhlich, F. Glorius, *J. Org. Chem.* **2008**, *76*, 2324–2327. (S3)

Following a literature procedure[1], crude **S2** (2.7 mmol, 1 equiv.) and sodium borohydride ($NaBH_4$, 212 mg, 5.6 mmol, 2.1 equiv.) were used to afford the crude product **S3**, which was used in the next step without further purification.

Crude **S3** (2.7 mmol, 1 equiv.) was dissolved in $CHCl_3$ (40 mL) and the solution was cooled down to 0 ^oC. Phosphorus tribromide (PBr_3, 0.10 mL, 1.0 mmol, 0.4 equiv.) was added dropwise to the solution at 0 ^oC and the mixture was stirred overnight (*ca*. 18 h). The mixture was then poured into crushed ice and the product was extracted with DCM (3 x 30 mL). The organic extract was dried over Na_2SO_4, filtered, and evaporated to dryness *in vacuo* to afford the crude product **S4**, which was used in the next step without further purification.

Crude **S4** (2.7 mmol, 1 equiv.) was dissolved in 10 mL of dry DMF and then sodium cyanide (NaCN, 327 mg, 6.7 mmol, 2.5 equiv.) was added at room temperature under a nitrogen atmosphere. After stirring overnight (*ca*. 18 h) at room temperature, the solution was poured into crushed ice and left at room temperature for 1 h. The precipitate formed was filtered, washed with deionized water and dried *in vacuo* to afford crude **S5** as an amorphous beige solid.

Crude **S5** (2.7 mmol, 1 equiv.) was dissolved in 30 mL of THF and the solution was cooled down to 0 ^oC. Then, concentrated HCl (3 mL) was added dropwise to the solution at 0 ^oC. After stirring overnight (*ca*. 18 h) at room temperature, deionized water was added to the solution and the product was extracted with DCM (3 x 30 mL). The organic extract was dried over Na_2SO_4, filtered, and evaporated to dryness *in vacuo* to afford the crude product **S6**, which was purified by column chromatography on silica gel with a mixture of petrol:EtOAc 1:1 as eluent to afford the product as an amorphous beige solid (0.311 g, 32% over 5 steps).
$[\alpha]_D^{21}$ = +52 [0.25 *c* in MeOH].
^{1}H NMR (400 MHz, $CDCl_3$): δ 8.16 (s, 2H), 7.95 (d, *J* = 8.0 Hz, 2H), 7.45 (t, *J* = 7.5 Hz, 2H), 7.36 (t, *J* = 7.5 Hz, 2H), 7.09 (d, *J* = 8.0 Hz, 2H), 5.19 (s, 2H), 4.00 (s, 4H).
^{13}C NMR (101 MHz, $CDCl_3$): δ 150.3, 133.0, 130.7, 129.3, 128.6, 128.4, 125.2, 124.0, 119.4, 117.6, 111.1, 19.6.
HRMS: (ESI) $[M+H]^+$ *m/z* calculated for $C_{24}H_{16}N_2O_2$ 364.1285, found 365.1290.
AR–FTIR (neat, cm^{-1}): 3351, 2265, 1624, 1507, 1457, 1360, 1207, 1142, 1012, 755.

Synthesis of Difluoroboranes

Synthesis of 10-(2-Ethylphenyl)-11,11-difluoro-8,9-dimethyl-11*H*-pyrido[3',2':3,4][1,2]azaborolo[1,5-*a*]quinolin-12-ium-11-uide (9a)

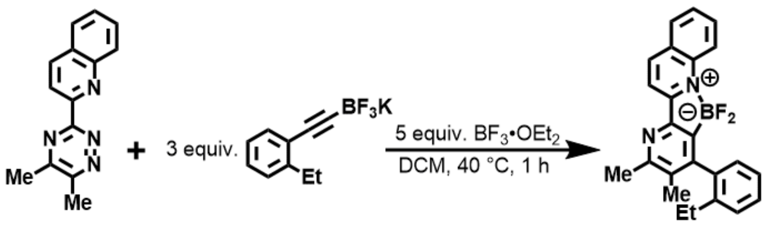

To a suspension of 5,6-dimethyl-3-(2-quinolyl)-1,2,4-triazine (100 mg, 0.423 mmol, 1 equiv.) and potassium (2-ethylphenyl)ethynyl)trifluoroborate (300 mg, 1.27 mmol, 3 equiv.) in DCM (4 mL) was added ***freshly distilled*** boron trifluoride diethyl ethereate (BF_3•OEt_2, 0.26 mL, 2.12 mmol, 5 equiv.). The mixture was stirred for 1 h at reflux. Then, saturated $NaHCO_3$(*aq.*) (15 mL) was added and the mixture was washed with deionized water (20 mL) and extracted with DCM (4 x 15 mL). The organic extract was dried over $MgSO_4$, filtered, and evaporated to dryness *in vacuo*. The residue was purified by column chromatography on silica gel with a mixture of DCM:EtOAc 1:1 as eluent to afford the product as a beige amorphous solid (110 mg, 67%).

^{1}H NMR (400 MHz, $CDCl_3$): δ 8.61 (d, *J* = 8.5 Hz, 1H), 8.50 (d, *J* = 9.0 Hz, 1H), 8.46 (d, *J* = 8.5 Hz, 1H), 7.97 (d, *J* = 7.5 Hz, 1H), 7.85 (ddd, *J* = 8.5, 7.0, 1.5 Hz, 1H), 7.69 – 7.60 (m, 1H), 7.42 – 7.34 (m, 2H), 7.34 – 7.28 (m, 1H), 7.19 (d, *J* = 7.5 Hz, 1H), 2.67 (s, 3H), 2.43 (q, *J* = 7.5 Hz, 2H), 2.09 (s, 3H), 1.06 (t, *J* = 7.5 Hz, 3H).

^{13}C NMR (101 MHz, $CDCl_3$): δ 158.5, 157.1, 154.6, 152.5, 152.3, 144.2, 141.2, 140.2, 138.1, 133.3, 133.2, 129.0, 128.7, 128.5, 128.0, 127.7, 125.6, 122.8, 115.4, 26.1, 23.6, 16.4, 14.6.

^{11}B NMR (128 MHz, $CDCl_3$): δ 8.9 (s, br).

^{19}F NMR (377 MHz, $CDCl_3$): δ –154.9 (d, *J* = 128 Hz), –156.8 (d, *J* = 116 Hz).

HRMS: (ESI) $[M+H]^+$ *m/z* calculated for $C_{24}H_{22}BF_2N_2$ 387.1844, found 387.1841.

AR–FTIR (neat, cm^{-1}): 2961, 2925, 2874, 1597, 1447, 1269, 1087.

Synthesis of 11,11-Difluoro-10-(2-methoxyphenyl)-8,9-dimethyl-11*H*-pyrido[3',2':3,4][1,2]azaborolo[1,5-*a*]quinolin-12-ium-11-uide (9b)

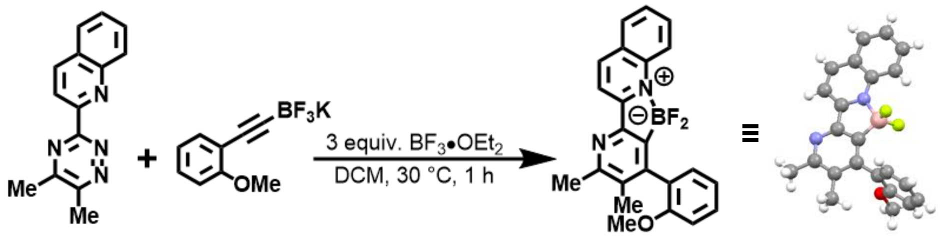

To a suspension of 5,6-dimethyl-3-(2-quinolyl)-1,2,4-triazine (99 mg, 0.42 mmol, 1 equiv.) and potassium (2-methoxyphenyl)ethynyltrifluoroborate salt (100 mg, 0.42 mmol, 1 equiv.) in DCM (4 mL) was added ***freshly distilled*** boron trifluoride diethyl etherate (BF_3•OEt_2, 0.15 mL, 1.26 mmol, 3 equiv.). The mixture was stirred for 1 h at 30 °C. Then, saturated $NaHCO_3$(*aq.*) (15 mL) was added and the mixture was washed with deionized water (20 mL) and extracted with DCM (4 x 15 mL). The organic extract was dried over $MgSO_4$, filtered, and evaporated to dryness *in vacuo*. The reside was purified by column chromatography on silica gel with a mixture of hexane:EtOAc 6:4 as eluent to afford the product as a white solid (132 mg, 81%).

M.P. 263–265 °C recrystallized from DCM.

^{1}H NMR (400 MHz, $CDCl_3$): δ 8.59 (d, *J* = 8.5 Hz, 1H), 8.52 (d, *J* = 9.0 Hz, 1H), 8.44 (d, *J* = 8.5 Hz, 1H), 7.96 (d, *J* = 8.0 Hz, 1H), 7.85 (ddd, *J* = 8.5, 7.0, 1.0 Hz, 1H), 7.67 – 7.62 (m, 1H), 7.42 (td, *J* = 8.0, 1.5 Hz, 1H), 7.38 (d, *J* = 7.5 Hz, 1H), 7.12 (td, *J* = 7.5, 1.0 Hz, 1H), 7.03 (d, *J* = 8.0 Hz, 1H), 3.78 (s, 3H), 2.66 (s, 3H), 2.14 (s, 3H).

^{13}C NMR (101 MHz, $CDCl_3$): δ 158.3, 157.3, 156.5, 152.4, 149.6, 144.2, 140.3, 134.4, 133.2, 130.7, 129.4, 129.1, 128.6, 128.4, 127.7, 122.9, 120.8, 115.5, 111.1, 55.8, 23.7, 16.5.

^{11}B NMR (128 MHz, $CDCl_3$): δ 8.7 (s, br).

^{19}F NMR (377 MHz, $CDCl_3$): δ –152.2 (d, *J* = 91.5 Hz), –158.6 (d, *J* = 91.5 Hz).

HRMS: (ESI) *m/z* $[M+H]^+$ calculated for $C_{23}H_{20}BF_2N_2O$ 389.1637, found 389.1640.

AR–FTIR (neat, cm^{-1}): 1599, 1558, 1381, 1187, 1083.

Synthesis of 10-(2-Ethylphenyl)-11,11-difluoro-8,9-diphenyl-11*H*-pyrido[3',2':3,4][1,2]azaborolo[1,5-*a*]quinolin-12-ium-11-uide (9c)

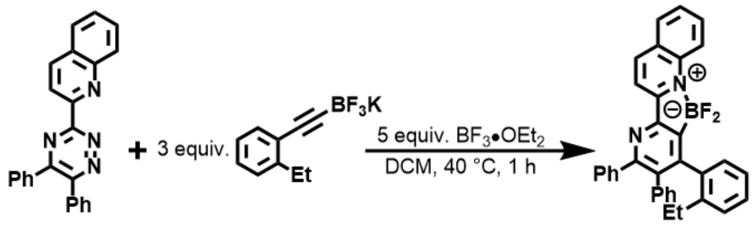

To a suspension of 5,6-diphenyl-3-(2-quinolyl)-1,2,4-triazine (85 mg, 0.24 mmol, 1 equiv.) and potassium ((2-ethylphenyl)ethynyl)trifluoroborate salt (139 mg, 0.59 mmol, 3 equiv.) in DCM (4 mL) was added ***freshly distilled*** boron trifluoride diethyl etherate (BF_3•OEt_2, 0.15 mL, 1.2 mmol, 5 equiv.). The mixture was stirred for 1 h at reflux. Then, saturated $NaHCO_3$ (*aq.*) (15 mL) was added and the mixture was washed with water (20 mL) and extracted with DCM (4 x 10 mL). The organic extract was dried over $MgSO_4$, filtered, and evaporated to dryness *in vacuo*. The product was slowly precipitated from DCM to afford it as an amorphous beige solid (55 mg, 46%).

^{1}H NMR (400 MHz, $CDCl_3$): δ 8.68 (d, *J* = 8.5 Hz, 1H), 8.61 – 8.56 (m, 2H), 8.02 (d, *J* = 8.0 Hz, 1H), 7.91 (t, *J* = 7.5 Hz, 1H), 7.70 (t, *J* = 7.5 Hz, 1H), 7.49 – 7.41 (m, 2H), 7.37 (d, *J* = 8.0 Hz, 1H), 7.30 – 7.21 (m, 5H), 7.18 – 7.10 (m, 1H), 7.08 – 6.90 (m, 5H), 2.51 – 2.35 (m, 1H), 2.23 – 2.09 (m, 1H), 1.02 (t, *J* = 7.5 Hz, 3H).

^{13}C NMR (101 MHz, $CDCl_3$): δ 159.2, 156.6, 154.2, 153.4, 144.5, 140.9, 140.8, 140.3, 138 .0, 137.9, 137.6, 133.5, 130.3 (x 2C), 129.9, 129.5, 128.7, 128.2, 127.9, 127.8 (x 2C), 127.7, 127.4, 127.3 (x 2C), 126.7, 124.8, 123.0, 115.9, 26.0, 14.2.

^{11}B NMR (128 MHz, $CDCl_3$): δ 7.3 (s, br).

^{19}F NMR (377 MHz, $CDCl_3$): δ –153.4 (d, *J* = 139.0 Hz), –155.9 (d, *J* = 139.0 Hz).

HRMS: (ESI) $[M+H]^+$ *m/z* calculated for $C_{34}H_{26}BF_2N_2$ 511.2157, found 511.2166.

AR–FTIR (neat, cm^{-1}): 3273, 2924, 1628, 1553, 1492, 1243, 1089.

Synthesis of 11,11-Difluoro-10-(2-methoxyphenyl)-8,9-diphenyl-11*H*-pyrido[3',2':3,4][1,2]azaborolo[1,5-*a*]quinolin-12-ium-11-uide (9d)

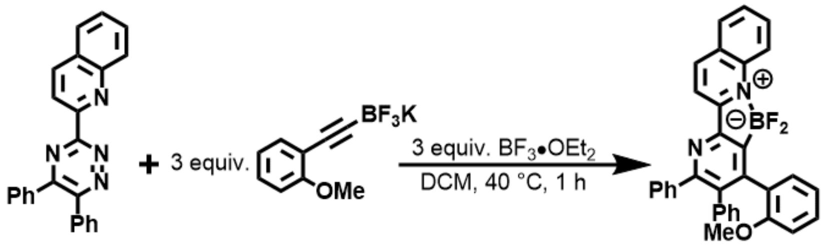

To a suspension of 5,6-diphenyl-3-(2-quinolyl)-1,2,4-triazine (85 mg, 0.24 mmol,1 equiv.) and potassium((2-ethylphenyl)ethynyl)trifluoroborate salt (139 mg, 0.59 mmol, 3 equiv.) in DCM (4 mL) was added ***freshly distilled*** boron trifluoride diethyl etherate (BF_3•OEt_2, 0.15 mL, 1.2 mmol, 3 equiv.). The mixture was stirred for 1 h at reflux. Then, saturated $NaHCO_3$ (*aq*.) (15 mL) was added and the reaction mixture was washed with water (20 mL) and extracted with DCM (4 x 15 mL). The organic extract was dried over $MgSO_4$, filtered, and evaporated to dryness *in vacuo*. The product was slowly precipitated from DCM and washed with Et_2O (15 mL) to afford the product as an amorphous pale grey solid (60 mg, 50%).

^{1}H NMR (400 MHz, $CDCl_3$): δ 8.66 (d, *J* = 8.5 Hz, 1H), 8.62 – 8.55 (m, 2H), 8.01 (d, *J* = 8.3 Hz, 1H), 7.90 (ddd, *J* = 8.5, 7.0, 1.5 Hz, 1H), 7.72 – 7.67 (m, 1H), 7.47 – 7.39 (m, 1H), 7.41 – 7.36 (m, 2H), 7.25 – 7.20 (m, 5H), 7.03 – 6.90 (m, 5H), 6.66 (d, *J* = 7.5 Hz, 1H), 3.39 (s, 3H).

^{13}C NMR (101 MHz, $CDCl_3$): δ 158.8, 156.6, 155.9, 154.3, 151.0, 145.0, 144.5, 140.7, 140.3, 138.9, 138.5, 133.5, 130.9, 130.3 (x2 C), 129.4, 128.7, 128.2, 128.1, 127.8 (x2 C), 127.0, 126.6, 123.1, 120.2, 116.1, 110.5, 55.1.

^{11}B NMR (128 MHz, $CDCl_3$): δ 9.2 (s, br).

^{19}F NMR (377 MHz, $CDCl_3$): δ –151.5 (d, *J* = 102.0 Hz), –158.0 (d, *J* = 102.0 Hz).

HRMS: (ESI) $[M+H]^+$ *m/z* calculated for $C_{33}H_{24}BF_2N_2O$ 513.1950, found 513.1959.

AR–FTIR (neat, cm^{-1}): 2925, 1597, 1240, 1103, 1006.

Synthesis of 11,11-Difluoro-10-(2-*iso*propoxyphenyl)-8,9-dimethyl-11*H*-pyrido[3',2':3,4][1,2]azaborolo[1,5-*a*]quinolin-12-ium-11-uide (9e)

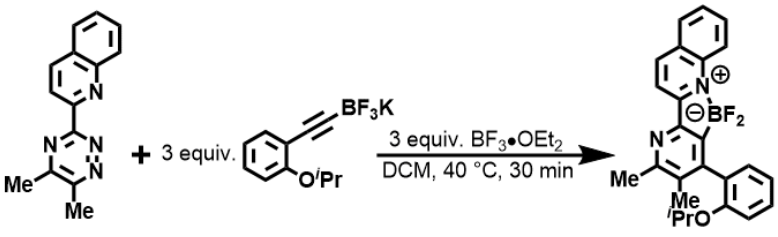

To a suspension of 5,6-dimethyl-3-(2-quinolyl)-1,2,4-triazine (100 mg, 0.4 mmol,1 equiv.) and potassium trifluoro((2-isopropoxyphenyl)ethynyl)borate (338 mg, 1.3 mmol, 3 equiv.) in DCM (4 mL) was added ***freshly distilled*** boron trifluoride diethyl etherate (BF_3•OEt_2, 0.16 mL, 1.3 mmol, 3 equiv.). The mixture was stirred for 30 min at reflux. Then, saturated $NaHCO_3$ (*aq.*) (15 mL) was added and the reaction mixture was washed with water (20 mL) and extracted with DCM (4 x 15 mL). The organic extract was dried over $MgSO_4$, filtered, and evaporated to dryness *in vacuo*. The reside was purified by column chromatography on silica gel with a gradient starting with DCM, ending in Et_2O to afford the product as an amorphous white solid (130 mg, 70%).

^{1}H NMR (400 MHz, CDCl$_3$): δ 8.60 (d, *J* = 8.5 Hz, 1H), 8.53 (d, *J* = 8.5 Hz, 1H), 8.44 (d, *J* = 8.5 Hz, 1H), 7.96 (d, *J* = 8.0 Hz, 1H), 7.88 – 7.81 (m, 1H), 7.64 (t, *J* = 7.5 Hz, 1H), 7.44 (d, *J* = 7.5 Hz, 1H), 7.40 – 7.34 (m, 1H), 7.14 (t, *J* = 7.5 Hz, 1H), 7.02 (d, *J* = 8.0 Hz, 1H), 4.38 – 4.25 (m, 1H), 2.67 (s, 3H), 2.17 (s, 3H), 1.19 (d, *J* = 6.0 Hz, 3H), 0.98 (d, *J* = 6.0 Hz, 3H).

^{13}C NMR (101 MHz, CDCl$_3$): δ 158.0, 157.4, 154.9, 152.3, 150.2, 144.2, 140.3, 134.7, 133.3, 131.1, 130.6, 129.3, 129.1, 128.6, 127.8, 123.0, 121.4, 116.2, 115.5, 71.5, 23.7, 22.2 (x 2C), 16.8.

^{19}F NMR (377 MHz, CDCl$_3$): δ –151.0 (d, *J* = 94.5 Hz), –158.4 (d, *J* = 94.5 Hz).

HRMS: (ESI) *m/z* $[M+H]^+$ calculated for $C_{25}H_{24}BF_2N_2O$ 417.1944, found 417.1944.

AR–FTIR (neat, cm^{-1}): 2915, 1600, 1523, 1227, 1102.

Synthesis of 10-(2-(Dimethylamino)phenyl)-11,11-difluoro-8,9-dimethyl-11*H*-pyrido[3',2':3,4][1,2]azaborolo[1,5-*a*]quinolin-12-ium-11-uide (9f)

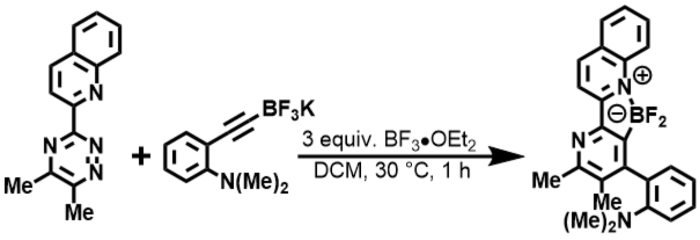

To a suspension of 5,6-dimethyl-3-(2-quinolyl)-1,2,4-triazine (100 mg, 0.42 mmol, 1 equiv.) and potassium trifluoro((2-dimethylaminophenyl)ethynyl)borate (106 mg, 0.42 mmol, 1 equiv.) in DCM (4 mL) was added ***freshly distilled*** boron trifluoride diethyl etherate (BF_3•OEt_2, 0.16 mL, 1.27 mmol, 3 equiv.). The mixture was stirred for 1 h at 30 °C. Then, saturated $NaHCO_3$ (*aq*.) (15 mL) was added and the reaction mixture was washed with water (20 mL) and extracted with DCM (4 x 15 mL). The organic extract was dried over $MgSO_4$, filtered, and evaporated to dryness *in vacuo*. The residue was purified by column chromatography on silica gel with a mixture of petrol:EtOAc 2:1 as eluent to afford the product as an amorphous white solid (124 mg, 73%).

^{1}H NMR (400 MHz, $CDCl_3$): δ 8.61 (d, J = 8.5 Hz, 1H), 8.54 (d, J = 8.5 Hz, 1H), 8.45 (d, J = 8.5 Hz, 1H), 7.96 (d, J = 8.0 Hz, 1H), 7.86 (t, J = 8.0, Hz, 1H), 7.68 – 7.60 (m, 1H), 7.40 – 7.32 (m, 2H), 7.16 – 7.07 (m, 2H), 2.67 (s, 3H), 2.55 (s, 6H), 2.09 (s, 3H).

^{13}C NMR (101 MHz, $CDCl_3$): δ 158.4, 157.4, 151.9, 150.3, 144.2, 142.4, 133.3, 132.8, 131.42, 131.39, 129.2, 128.9, 128.7, 127.8, 123.0, 122.9, 121.7, 117.7, 115.5, 43.7 (x2 C), 23.9, 16.3.

^{11}B NMR (128 MHz, $CDCl_3$): δ 8.9 (s, br).

^{19}F NMR (377 MHz, $CDCl_3$): δ –150.5 (d, *J* = 89.0 Hz), 158.3 (d, *J* = 89.0 Hz).

HRMS: (ESI) $[M+H]^+$ *m/z* calculated for $C_{24}H_{22}BF_2N_3$ 402.1948, found 402.1962.

AR–FTIR (neat, cm^{-1}): 3923, 1596, 1523, 1451, 1382, 1264, 1100, 1082.

Synthesis of 11,11-Difluoro-8,9-dimethyl-10-(2-(methylthio)phenyl)-11*H*-pyrido[3',2':3,4][1,2]azaborolo[1,5-*a*]quinolin-12-ium-11-uide (9g)

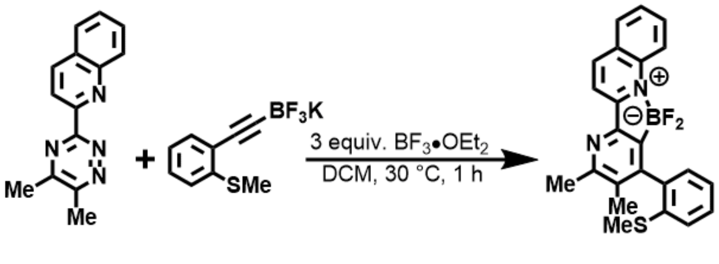

To a suspension of 5,6-dimethyl-3-(2-quinolyl)-1,2,4-triazine (100 mg, 0.4 mmol, 1 equiv.) and potassium (2-thiomethoxyphenyl)ethynyltrifluoroborate salt (109 mg, 0.4 mmol, 1 equiv.) in DCM (4 mL) was added ***freshly distilled*** boron trifluoride diethyl ethereate (BF_3•OEt_2, 0.2 mL, 1.3 mmol, 3 equiv.). The mixture was stirred for 1 h at 30 °C. Then, saturated $NaHCO_3$ (*aq.*) (15 mL) was added and the reaction mixture was washed with water (20 mL) and extracted with DCM (4 x 15 mL). The organic extract was dried over $MgSO_4$, filtered, and evaporated to dryness *in vacuo*. The reside was purified by column chromatography on silica gel with a mixture of DCM:Et_2O 98:2 as eluent to afford the product as a white solid (111 mg, 78%).

M.P. 255–257 °C recrystallized from DCM.

^{1}H NMR (400 MHz, $CDCl_3$): δ 8.59 (d, *J* = 8.0 Hz, 1H), 8.49 (d, *J* = 9.0 Hz, 1H), 8.43 (d, *J* = 9.0 Hz, 1H), 7.95 (d, *J* = 8.0 Hz, 1H), 7.83 (t, *J* = 8.0 Hz, 1H), 7.63 (t, *J* = 7.0 Hz, 1H), 7.44 – 7.38 (m, 2H), 7.35 – 7.24 (m, 2H), 2.68 (s, 3H), 2.36 (s, 3H), 2.14 (s, 3H).

^{13}C NMR (101 MHz, $CDCl_3$): δ 158.5, 157.0, 152.5, 150.9, 144.2, 140.2, 138.7, 136.6, 133.9, 133.1, 129.1, 129.0, 128.5, 128.4, 127.7, 126.5, 125.3, 122.8, 115.4, 23.5, 16.3, 16.2.

^{11}B NMR (128 MHz, $CDCl_3$): δ 8.7 (s, br).

^{19}F NMR (377 MHz, $CDCl_3$): δ –154.1 (d, *J* = 90.0 Hz), –157.5 (d, *J* = 90.0 Hz).

HRMS: (ESI) *m/z* $[M+H]^+$ calculated for $C_{23}H_{20}BF_2N_2S$ 405.1403, found 405.1411.

AR–FTIR (neat, cm^{-1}): 2918, 1618, 1596, 1276, 725.

Synthesis of 9-(2-Ethylphenyl)-8,8-difluoro-10,11-dimethyl-8*H*-pyrido[3',2'3,4][1,2]azaborolo[5,1-*a*]isoquinolin-7-ium-8-uide (9h)

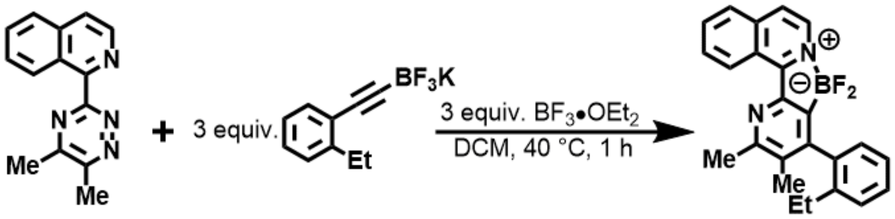

To a suspension of 5,6-dimethyl-3-(2-isoquinolyl)-1,2,4-triazine (100 mg, 0.424 mmol,1 equiv.) and potassium ((2-ethylphenyl)ethynyl)trifluoroborate (290 mg, 1.27 mmol, 3 equiv.) in DCM (4 mL) was added ***freshly distilled*** boron trifluoride diethyl etherate (BF_3•OEt_2, 0.26 mL, 2.14 mmol, 3 equiv.). The mixture was stirred for 1 h at reflux. Then, saturated $NaHCO_3$ (*aq.*) (15 mL) was added and the reaction mixture was washed with water (20 mL) and extracted with DCM (4 x 15 mL). The organic extract was dried over $MgSO_4$, filtered, and evaporated to dryness *in vacuo*. The residue was purified by column chromatography on Fluorisil® with a mixture of hexane:EtOAc 4:1 as eluent to afford the product as an amorphous pale yellow solid (66 mg, 40%).

^{1}H NMR (400 MHz, $CDCl_3$): δ 10.58 (dd, *J* = 8.5, 1.0 Hz, 1H), 8.27 (d, *J* = 6.5 Hz, 1H), 7.98 – 7.94 (m, 2H), 7.93 – 7.87 (m, 1H), 7.84 (d, *J* = 6.0 Hz, 1H), 7.39 – 7.34 (m, 1H), 7.32 – 7.27 (m, 2H), 7.17 (d, *J* = 7.0 Hz, 1H), 2.74 (s, 3H), 2.43 (q, *J* = 7.5 Hz, 2H), 2.09 (s, 3H), 1.07 (t, *J* = 7.5 Hz, 3H).

^{13}C NMR (101 MHz, $CDCl_3$): δ 157.8, 155.2, 154.4, 152.1, 141.2, 139.6, 138.2, 134.0, 132.4, 132.0, 130.1, 130.0, 128.7, 128.0, 127.9, 127.0, 125.5, 125.2, 123.0, 60.4, 26.1, 23.9, 21.1.

^{11}B NMR (128 MHz, $CDCl_3$): δ 7.3 (s, br).

^{19}F NMR (377 MHz, $CDCl_3$): δ –162.46 (d, *J* = 131.0 Hz), –164.74 (d, *J* = 131.0 Hz).

HRMS: (ESI) $[M+H]^+$ *m/z* calculated for $C_{24}H_{22}BF_2N_2$ 387.1844, found 387.1845.

AR–FTIR (neat, cm^{-1}): 2961, 2925, 2874, 1597, 1447, 1269, 1087.

Synthesis of 8,8-Difluoro-9-(2-methoxyphenyl)-10,11-dimethyl-8*H*-pyrido[3',2':3,4][1,2]azaborolo[5,1-*a*]isoquinolin-7-ium-8-uide (9i)

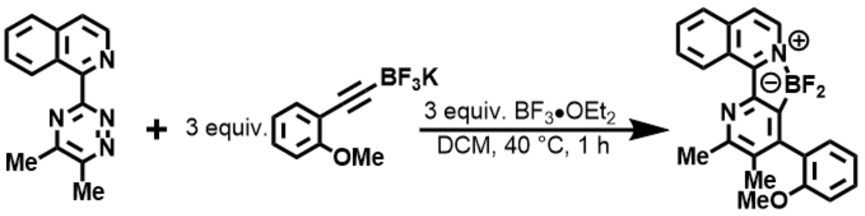

To a suspension of 5,6-dimethyl-3-(2-isoquinolyl)-1,2,4-triazine (100 mg, 0.424 mmol, 1 equiv.) and potassium ((2-methoxyphenyl)ethynyl)trifluoroborate (250 mg, 1.07 mmol, 3 equiv.) in DCM (4 mL) was added ***freshly distilled*** boron trifluoride diethyl etherate (BF_3•OEt_2, 0.13 mL, 1.07 mmol, 3 equiv.). The mixture was stirred for 1 h at reflux. Then, saturated $NaHCO_3$ (*aq.*) (15 mL) was added and the reaction mixture was washed with water (20 mL) and extracted with DCM (4 x 15 mL). The organic extract was dried over $MgSO_4$, filtered, and evaporated to dryness *in vacuo*. The product was slowly precipitated from DCM to afford the product as an amorphous pale brown solid (130 mg, 70%).

^{1}H NMR (400 MHz, $CDCl_3$): δ 10.60 (d, *J* = 8.0 Hz, 1H), 8.30 (d, *J* = 6.0 Hz, 1H), 8.00 – 7.94 (m, 2H), 7.94 – 7.88 (m, 1H), 7.85 (d, *J* = 6.0 Hz, 1H), 7.46 – 7.36 (m, 2H), 7.13 (td, *J* = 7.5, 1.0 Hz, 1H), 7.04 (d, *J* = 8.5 Hz, 1H), 3.80 (s, 3H), 2.75 (s, 3H), 2.17 (s, 3H).

^{13}C NMR (101 MHz, $CDCl_3$): δ 157.4, 156.3, 155.2, 154.6, 149.4, 139.5, 134.0, 133.4, 132.0, 130.6, 130.3, 129.7, 129.2, 128.4, 126.9, 125.2, 122.8, 120.7, 111.0, 55.7, 23.7, 16.3.

^{11}B NMR (128 MHz, $CDCl_3$): δ 7.3 (s, br).

^{19}F NMR (377 MHz, $CDCl_3$): δ –159.7 (d, *J* = 125.0 Hz), –166.8 (d, *J* = 123.0 Hz).

HRMS: (ESI) $[M+H]^+$ *m/z* calculated for $C_{23}H_{20}BF_2N_2O$ 389.1637, found 389.1632.

AR–FTIR (neat, cm^{-1}): 3079, 2953, 2836, 1600, 1553, 1494, 1353, 1243, 1089.

Synthesis of 9-(2-Ethylphenyl)-8,8-difluoro-10,11-diphenyl-8*H*-pyrido[3',2':3,4][1,2]azaborolo[5,1-*a*]isoquinolin-7-ium-8-uide (9j)

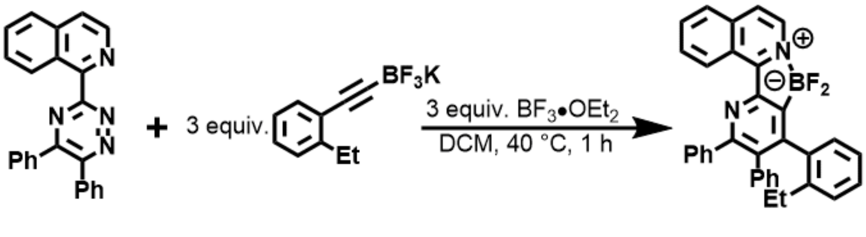

To a suspension of 5,6-diphenyl-3-(2-isoquinolyl)-1,2,4-triazine (85 mg, 0.236 mmol, 1 equiv.) and potassium ((2-ethylphenyl)ethynyl)trifluoroborate (139 mg, 0.590 mmol, 3 equiv.) in DCM (4 mL) was added ***freshly distilled*** boron trifluoride diethyl etherate (BF_3•OEt_2, 0.15 mL, 1.18 mmol, 3 equiv.). The mixture was stirred for 1 h at reflux. Then, saturated $NaHCO_3$ (*aq.*) (15 mL) was added and the reaction mixture was washed with water (20 mL) and extracted with DCM (4 x 15 mL). The organic extract was dried over $MgSO_4$, filtered, and evaporated to dryness *in vacuo*. The product was slowly precipitated from DCM to afford the product as an amorphous pale-yellow solid (55 mg, 46%).

^{1}H NMR (400 MHz, $CDCl_3$): δ 10.60 (d, *J* = 8.5 Hz, 1H), 8.40 (d, *J* = 6.5 Hz, 1H), 8.07 – 7.88 (m, 4H), 7.51 – 7.45 (m, 2H), 7.34 – 7.18 (m, 6H), 7.15 – 7.08 (m, 1H), 7.06 – 6.89 (m, 5H), 2.46 – 2.34 (m, 1H), 2.19 – 2.07 (m, 1H), 0.99 (t, *J* = 7.5 Hz, 3H).

^{13}C NMR (101 MHz, $CDCl_3$): δ 158.4, 157.0, 153.7, 153.2, 141.1, 140.7, 139.7, 137.8, 137.5, 137.1, 134.2, 132.2, 130.2 (x2 C), 130.2, 130.1, 129.8, 127.7 (x2 C), 127.5, 127.2 (x2 C), 127.1, 126.6, 125.4, 124.7, 123.7, 25.8, 14.1.

^{11}B NMR (128 MHz, $CDCl_3$): δ 7.3 (s, br).

^{19}F NMR (377 MHz, $CDCl_3$): δ –161.0 (d, *J* = 111.0 Hz), –163.9 (d, *J* = 111.0 Hz).

HRMS: (ESI) $[M+H]^+$ *m/z* calculated for $C_{34}H_{26}BF_2N_2$ 511.2152, found 511.2167.

AR–FTIR (neat, cm^{-1}): 3272, 2925, 1628, 1552, 1493, 1243, 1089.

Synthesis of 8,8-Difluoro-9-(2-methoxyphenyl)-10,11-diphenyl-8*H*-pyrido[3',2':3,4][1,2]azaborolo[5,1-*a*]isoquinolin-7-ium-8-uide (9k)

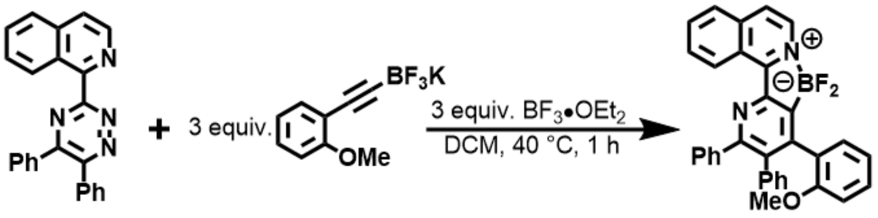

To a suspension of 5,6-diphenyl-3-(2-isoquinolyl)-1,2,4-triazine (121 mg, 0.336 mmol,1 equiv.) and potassium ((2-methoxyphenyl)ethynyl)trifluoroborate (240 mg, 1.00 mmol, 3 equiv.) in DCM (4 mL) was added ***freshly distilled*** boron trifluoride diethyl etherate (BF_3•OEt_2, 0.13 mL, 1.00 mmol, 3 equiv.). The mixture was stirred for 1 h at reflux. Then, saturated $NaHCO_3$ (*aq.*) (15 mL) was added and the reaction mixture was washed with water (20 mL) and extracted with DCM (4 x 15 mL). The organic extract was dried over $MgSO_4$, filtered, and evaporated to dryness *in vacuo*. The product was slowly precipitated from DCM to afford the product as an amorphous pale yellow solid (104 mg, 60%).

^{1}H NMR (400 MHz, $CDCl_3$): δ 10.57 (d, *J* = 8.5 Hz, 1H), 8.40 (d, *J* = 6.0 Hz, 1H), 8.04 – 7.85 (m, 4H), 7.51 – 7.42 (m, 3H), 7.32 – 7.21 (m, 4H), 7.09 – 6.93 (m, 6H), 6.67 (d, *J* = 8.0 Hz, 1H), 3.41 (s, 3H).

^{13}C NMR (101 MHz, $CDCl_3$): δ 158.1, 157.4, 155.9, 154.0, 150.6, 141.3, 139.8, 138.6, 137.9, 134.3, 132.2, 131.0, 130.4 (x2 C), 130.3, 129.2, 128.1, 127.8 (x2 C), 127.5, 127.2, 127.0, 126.6, 125.4, 123.7, 120.2, 110.4, 55.1.

^{11}B NMR (128 MHz, $CDCl_3$): δ 7.8 (s, br).

^{19}F NMR (377 MHz, $CDCl_3$): δ –158.8 (d, *J* = 114.0 Hz), –165.9 (d, *J* = 114.0 Hz).

HRMS: (ESI) $[M+H]^+$ *m/z* calculated for $C_{33}H_{24}BF_2N_2O$ 513.1950, found 513.1961.

AR–FTIR (neat, cm^{-1}): 3057, 2838, 1547, 1243, 1109, 1009.

Synthesis of 5,5-Difluoro-4-(2-methoxyphenyl)-2,3-dimethyl-5*H*-[1,2]azaborolo[1,5-*a*:4,3-*b*']dipyridin-6-ium-5-uide (9l)

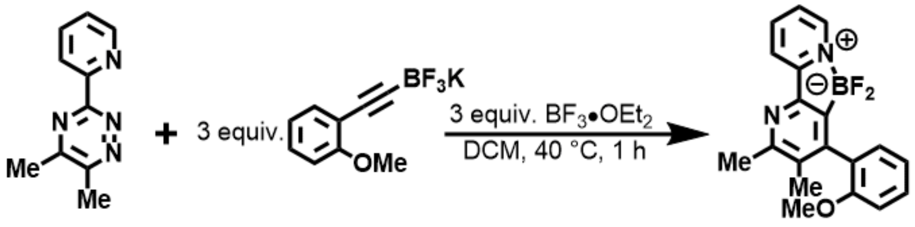

To a suspension of 5,6-diphenyl-3-(2-quinolyl)-1,2,4-triazine (100 mg, 0.424 mmol,1 equiv.) and potassium ((2-methoxyphenyl)ethynyl)trifluoroborate (197 mg, 1.06 mmol, 3 equiv.) in DCM (4 mL) was added freshly distilled boron trifluoride diethyl etherate (BF_3•OEt_2, 0.13 mL, 1.06 mmol, 3 equiv.). The mixture was stirred for 1 h at reflux. Then, saturated $NaHCO_3$ (*aq.*) (15 mL) was added and the reaction mixture was washed with water (20 mL) and extracted with DCM (4 x 15 mL). The organic extract was dried over $MgSO_4$, filtered, and evaporated to dryness *in vacuo*. The residue was purified by column chromatography on silica gel using EtOAc as eluent to afford the product as an amorphous pale yellow solid (60 mg, 30%)

^{1}H NMR (400 MHz, $CDCl_3$): δ 8.44 (d, *J* = 5.5 Hz, 1H), 8.29 (d, *J* = 8.0 Hz, 1H), 8.15 (t, *J* = 7.5 Hz, 1H), 7.56 – 7.46 (m, 1H), 7.39 (t, *J* = 8.5 Hz, 1H), 7.32 (d, *J* = 7.5 Hz, 1H), 7.08 (t, *J* = 7.5 Hz, 1H), 7.00 (d, *J* = 8.5 Hz, 1H), 3.76 (s, 3H), 2.63 (s, 3H), 2.10 (s, 3H).

^{13}C NMR (101 MHz, $CDCl_3$): δ 158.3, 156.4, 156.0, 151.9, 149.9, 143.8, 141.3, 133.9, 130.6, 129.4, 128.4, 124.3, 120.8, 118.6, 111.0, 55.8, 23.7, 16.5.

^{11}B NMR (128 MHz, $CDCl_3$): δ 7.6 (s, br).

^{19}F NMR (377 MHz, $CDCl_3$): δ –156.2 (d, *J* = 90.0 Hz), –163.2 (d, *J* = 90.0 Hz).

HRMS: (ESI) $[M+H]^+$ *m/z* calculated for $C_{19}H_{18}BF_2N_2O$ 339.1480, found 339.1486.

AR–FTIR (neat, cm^{-1}): 2923, 1626, 1560, 1486, 1244, 1077.

General Procedures for the Synthesis of Boronic Esters

Procedure 1

To a solution of aryl difluoroborane (1 equiv.) in THF (0.03 M) was added NaOH (1.0 M in water, 5 equiv.). The reaction was stirred *ca*. 18 h at reflux. Then, the mixture was allowed to cool down to room temperature and concentrated *in vacuo*. The concentrate was washed with water and 1.0 M HCl (*aq*.) (10 mL) and extracted with DCM (3 x 15 mL). The organic extract was dried over $MgSO_4$, filtered, and evaporated to dryness *in vacuo*. The crude material was dissolved in PhMe (0.03 M) and then (*R*)-(+)-1,1'-bi(2-naphtol) ((*R*)-BINOL, 1 equiv.) was added. The reaction was stirred for 1 h at 120 °C. Then, the mixture was allowed to cool down to room temperature and the volatiles were evaporated to dryness *in vacuo* at ***35 °C***. The residue was purified by column chromatography or by recrystallization to afford the title compound.

Procedure 2

Aryl difluoroborane (1 equiv.) and Cs_2CO_3 (3 equiv.) were dissolved in a mixture of THF:H_2O 9:1 (0.1 M). The mixture was stirred at reflux for 12 h. Then, the mixture was allowed to cool down to room temperature and was concentrated *in vacuo*. The concentrate was washed with deionized water (15 mL) and 1 M HCl (*aq*.) and extracted with DCM. The organic extract was dried over $MgSO_4$, filtered, and evaporated to dryness *in vacuo*. The residue was redissolved in PhMe and (*R*)-BINOL (1 equiv.) was added. The mixture was heated at 120 °C for 12 h under Dean-Stark conditions. Then, the mixture was allowed to cool down to room temperature and the volatiles were evaporated *in vacuo* at ***35 °C***. The residue was purified by column chromatography or by recrystallization to afford the title compound.

Synthesis of (4*S*,10'*R*,11b*R*)-10'-(2-Ethylphenyl)-8',9'-dimethylspiro[dinaphtho[2,1-*d*:1',2'-*f*][1,3,2]dioxaborepine-4,11'-pyrido[3',2'3,4][1,2]azaborolo[1,5-*a*]quinolin]-12'-ium-26-uide (10a)

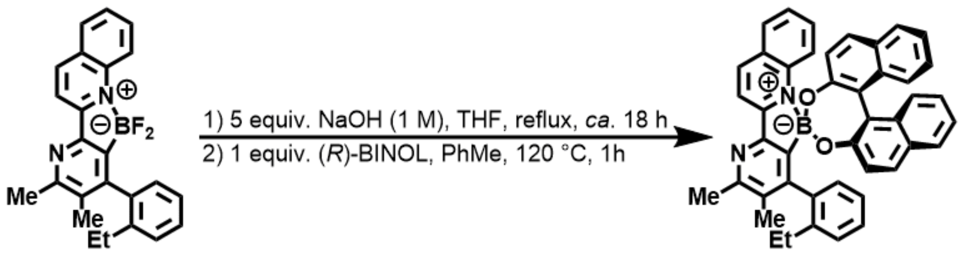

Following general procedure 2, aryl difluoroborane **9a** (90 mg, 0.23 mmol, 1 equiv.) and (*R*)-BINOL (133 mg, 0.47 mmol, 2 equiv.) were used to afford the crude product, which was purified by column chromatography on silica gel with a mixture hexane:Et_2O 1:1 as eluent to afford the product as a 1:1 mixture of diastereomers (101 mg, 69%). The compound could not be generated in pure form due to rapid hydrolysis of the boronic ester, and the sample contains ~25% BINOL. The yield has been corrected against this impurity.

^{1}H NMR (400 MHz, C_6D_6): δ 8.39 (d, *J* = 8.5 Hz, 1H), 8.34 (d, *J* = 8.5 Hz, 1H), 8.12 (d, *J* = 9.0 Hz, 1H), 8.06 (d, *J* = 9.0 Hz, 1H), 7.75 – 7.42 (m, 9H), 7.38 – 7.19 (m, 9H), 7.12 – 6.92 (m, 10H), 6.85 (t, *J* = 8.5 Hz, 2H), 6.68 – 6.56 (m, 4H), 6.47 – 6.31 (m, 4H), 6.28 – 6.22 (m, 1H), 6.19 (t, *J* = 7.5 Hz, 1H), 2.70 – 2.40 (m, 10H), 1.81 (s, 3H), 1.79 (s, 3H), 1.26 (t, *J* = 7.5 Hz, 3H), 0.93 (t, *J* = 7.5 Hz, 3H).

^{13}C NMR (101 MHz, C_6D_6): δ 158.2, 158.2, 158.0, 157.9, 156.2, 155.8, 155.7, 153.3, 153.2, 152.8, 152.5, 143.5, 143.4, 141.6, 141.2, 141.0, 139.5, 138.8, 138.6, 134.5, 134.4, 134.3, 133.9, 133.8, 131.3, 131.0, 130.8, 130.7, 130.5, 130.3, 129.8, 129.7, 129.4, 129.2, 129.1, 127.5, 127.2, 127.0, 126.7, 126.6, 126.5, 126.4, 125.9, 125.7, 125.6, 125.2, 125.1, 125.0, 124.9, 124.1, 124.0, 123.9, 123.8 (x 2C), 123.6, 123.5, 123.3 (x 2C), 122.6, 121.3, 121.1, 115.8, 26.3, 26.1, 23.6, 23.5, 16.9, 16.6, 14.6, 14.0.

^{11}B NMR (128 MHz, C_6D_6): δ 13.6 (s, br).

HRMS: (ESI) *m/z* $[M+H]^+$ calculated for $C_{44}H_{34}BN_2O_2$ 633.2708, found 633.2710.

AR–FTIR (neat, cm^{-1}): 3055, 2963, 2934, 1593, 1505, 1466, 1339, 1254, 1098, 1023.

Synthesis of (4*S*,10'*R*,11b*R*)-10'-(2-Methoxyphenyl)-8',9'-dimethylspiro[dinaphtho[2,1-*d*:1',2'-*f*][1,3,2]dioxaborepine-4,11'-pyrido[3',2':3,4][1,2]azaborolo[1,5-*a*]quinolin]-12'-ium-26-uide (10b)

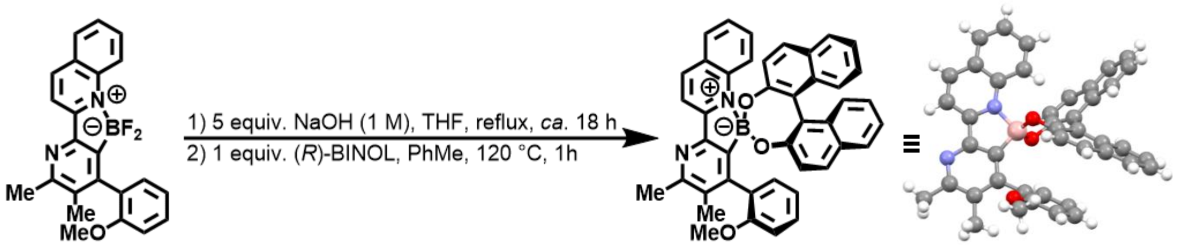

Following general procedure 1, difluoroborane **9b** (53 mg, 0.13 mmol), and (*R*)-BINOL (39 mg, 0.13 mmol) were combined to give the crude boronic ester. The residue was purified by column chromatography on silica gel with a mixture of petrol:EtOAc 7:3 as eluent to afford the product as a 4:1 mixture of diastereomers (49 mg, 57%).

Following general procedure 1, aryl difluoroborane **9b** (100 mg, 0.13 mmol, 1 equiv.) and (*R*)-BINOL (148 mg, 0.13 mmol, 1 equiv.) The residue was purified by column chromatography on silica gel with a mixture of petrol:EtOAc 7:3 as eluent to afford the product as a 4:1 mixture of diastereomers (49 mg, 57%).

<u>Diastereomer enrichment by precipitation</u>: Following general procedure 2 with difluoroborane **9b** (100 mg, 0.26 mmol, 1 equiv.), caesium carbonate (Cs_2CO_3, 252 mg, 0.78 mmol, 3 equiv.) and (*R*)-BINOL (148 mg, 0.52 mmol, 2 equiv.). The product was slowly precipitated from MeCN and was obtained as a yellow solid (108 mg, 66%, ≥98:2 *d.r.*).

$[\alpha]_D^{20}$ = –862 [1.0 *c* in $CHCl_3$].

M.P. 175–177 °C (decomposition) recrystallized from PhMe.

^{1}H NMR (400 MHz, $CDCl_3$, major diastereomer): δ 8.58 (d, *J* = 8.5 Hz, 1H), 8.53 (d, *J* = 8.5 Hz, 1H), 7.80 (dd, *J* = 8.5, 1.5 Hz, 2H), 7.71 (d, *J* = 8.0 Hz, 1H), 7.68 (d, *J* = 9.0 Hz, 1H), 7.53 (d, *J* = 8.5 Hz, 1H), 7.37 (d, *J* = 8.5 Hz, 1H), 7.35 – 7.27 (m, 3H), 7.21 (d, *J* = 7.5 Hz, 1H), 7.17 – 7.09 (m, 4H), 6.88 (d, *J* = 8.5 Hz, 1H), 6.82 (d, *J* = 8.5 Hz, 1H), 6.67 (ddd, *J* = 8.5, 7.0, 1.5 Hz, 1H), 6.30 (dd, *J* = 8.5, 1.0 Hz, 1H), 6.25 (ddd, *J* = 8.5, 7.0, 1.5 Hz, 1H), 5.86 (td, *J* = 7.5, 1.0 Hz, 1H), 3.80 (s, 3H), 2.65 (s, 3H), 1.94 (s, 3H).

^{13}C NMR (101 MHz, $CDCl_3$, major diastereomer): δ 158.2, 157.6, 155.4, 155.1, 155.0, 143.7, 141.1, 135.0, 133.7, 133.5, 131.6, 131.5, 130.4, 130.2, 129.3, 129.2, 129.1, 128.5, 128.2, 127.9, 127.8, 127.7, 127.6, 127.3, 126.9, 125.2, 124.6, 124.4, 124.0, 123.8, 123.7, 123.2, 123.0, 122.6, 122.4, 121.3, 119.0, 115.8, 109.6, 54.9, 23.8, 16.5.

^{11}B NMR (128 MHz, C_6D_6): δ 13.2 (s, br).

HRMS: (ESI) *m/z* $[M+H]^+$ calculated for $C_{43}H_{32}BN_2O_3$ 635.2508, found 635.2511.

AR–FTIR (neat, cm^{-1}): 3059, 2952, 1594, 1523, 1253.

Synthesis of (4*S*,10'*R*,11b*R*)-10'-(2-Ethylphenyl)-8',9'-diphenylspiro[dinaphtho[2,1-*d*:1',2'-*f*][1,3,2]dioxaborepine-4,11'-pyrido[3',2':3,4][1,2]azaborolo[1,5-*a*]quinolin]-12'-ium-26-uide (10c)

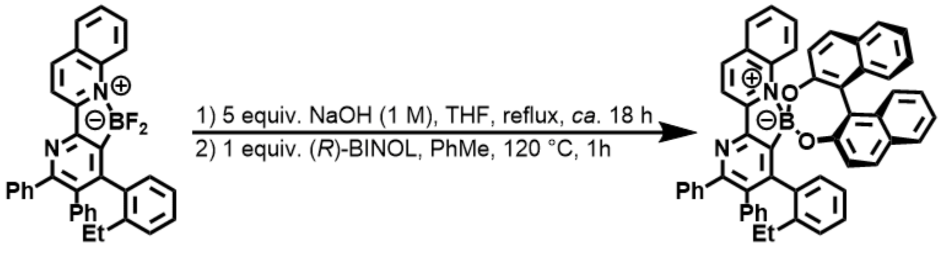

Following general procedure 1, aryl difluoroborane **9c** (50 mg, 0.1 mmol, 1 equiv.), and (*R*)-BINOL (28 mg, 0.1 mmol, 1 equiv.) were combined to give the crude product. The residue was purified by column chromatography on silica gel using a mixture of petrol:EtOAc 7:3 as eluent to afford the title compound as a 1:1 mixture of diastereomers (34 mg, 46%).

^{1}H NMR (400 MHz, C_6D_6): δ 8.43 – 8.36 (m, 2H), 8.18 (d, *J* = 9.0 Hz, 1H), 8.10 (d, *J* = 9.0 Hz, 1H), 7.78 (d, *J* = 7.5 Hz, 1H), 7.74 – 7.62 (m, 7H), 7.62 – 7.39 (m, 9H), 7.38 – 7.19 (m, 8H), 7.13 – 6.96 (m, 17H), 6.89 (d, *J* = 8.5 Hz, 2H), 6.74 – 6.53 (m, 9H), 6.44 – 6.13 (m, 7H), 3.05 – 2.93 (m, 1H), 2.60 – 2.48 (m, 1H), 2.45 – 2.32 (m, 1H), 2.30 – 2.17 (m, 1H), 1.04 – 0.93 (m, 6H).

^{13}C NMR (101 MHz, C_6D_6): δ 159.2, 159.1, 157.6, 157.5, 156.1, 155.8, 155.7, 155.5, 155.3, 154.8, 154.5, 153.3, 143.7, 143.6, 142.1, 141.9, 141.8, 141.4, 140.7, 139.4, 139.3, 139.2, 138.7, 138.4, 138.1, 134.5, 134.4, 134.3, 134.2, 132.3, 131.9, 131.8, 131.5, 131.4, 131.1, 131.0, 130.9 (x2 C), 130.8 (x2 C), 130.7, 130.6, 129.9, 129.8, 129.6, 129.5, 129.4, 128.6 (x2 C), 127.6, 127.4, 127.3, 127.0, 126.9, 126.8, 126.7, 126.6, 126.5, 125.8, 125.7, 125.5, 125.3, 125.1, 124.8, 124.4, 124.2, 124.1, 124.0 (x2 C), 123.7 (x2 C), 123.6, 123.5, 123.4, 123.3, 123.2, 122.9, 121.1, 120.8, 118.3, 116.0, 26.0, 25.9, 13.6, 13.5.

^{11}B NMR (128 MHz, C_6D_6): δ 13.7 (s, br).

HRMS: (ESI) *m/z* $[M+H]^+$ calculated for $C_{54}H_{38}BN_2O_2$ 759.2813, found 759.2781.

AR–FTIR (neat, cm^{-1}): 3057, 2971, 2929, 2924, 1619, 1596, 1544, 1339, 1252, 1098.

Synthesis of (4*S*,10'*R*,11b*R*)-10'-(2-Methoxyphenyl)-8',9'-diphenylspiro[dinaphtho[2,1-*d*:1',2'-*f*][1,3,2]dioxaborepine-4,11'-pyrido[3',2':3,4][1,2]azaborolo[1,5-*a*]quinolin]-12'-ium-26-uide (10d)

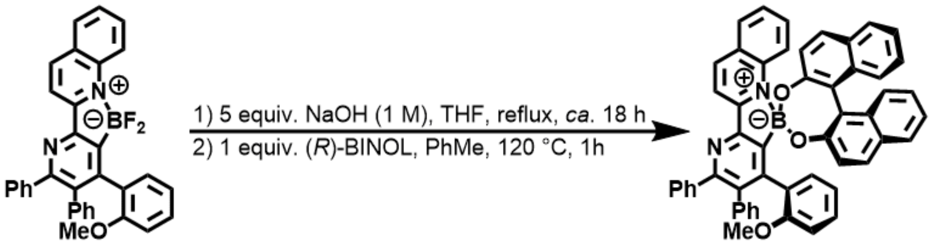

Following general procedure 1, aryl difluoroborane **9d** (140 mg, 0.27 mmol, 1 equiv.), and (*R*)-BINOL (78 mg, 0.27 mmol, 1 equiv.) were combined to give the crude product. The residue was purified by column chromatography on silica gel with DCM as eluent to afford the title compound as a 4:1 mixture of diastereomers (111 mg, 54%)

^{1}H NMR (400 MHz, C_6D_6, major diastereomer): δ 8.31 (d, *J* = 8.5 Hz, 1H), 8.16 (d, *J* = 9.0 Hz, 1H), 7.78 – 7.69 (m, 2H), 7.66 (d, *J* = 8.5 Hz, 1H), 7.60 – 7.55 (m, 3H), 7.53 (d, *J* = 8.5 Hz, 1H), 7.46 (d, *J* = 8.5 Hz, 1H), 7.41 (d, *J* = 8.5 Hz, 1H), 7.33 – 7.18 (m, 5H), 7.14 – 6.97 (m, 8H), 6.84 (t, *J* = 7.0 Hz, 1H), 6.75 (t, *J* = 7.5 Hz, 1H), 6.71 – 6.62 (m, 2H), 6.36 (t, *J* = 8.5 Hz, 1H), 6.18 (t, *J* = 8.0 Hz, 1H), 5.99 (t, *J* = 7.5 Hz, 1H), 5.72 (d, *J* = 8.0 Hz, 1H), 2.96 (s, 3H).

^{13}C NMR (101 MHz, C_6D_6, major diastereomer): δ 158.5, 157.8, 156.2, 156.1, 155.4, 155.1, 151.3, 143.6, 142.3, 141.4, 139.7, 139.6, 134.5, 132.6, 132.2, 131.3, 131.0, 130.9 (x 2C), 130.7, 129.7, 129.5, 127.5, 126.8, 126.6, 126.2, 125.8, 125.0, 124.5, 124.2, 123.6, 123.4, 123.2, 121.6, 118.8, 116.0, 109.7, 53.7. (*A complete assignment could not be achieved due to a large number of overlapping signals*).

^{11}B NMR (128 MHz, C_6D_6): δ 14.3 (s, br).

HRMS: (ESI) *m/z* $[M+H]^+$ calculated for $C_{53}H_{36}BN_2O_2$ 759.2819, found 759.2781.

AR–FTIR (neat, cm^{-1}): 3057, 2971, 2929, 2924, 1619, 1596, 1544, 1339, 1252, 1098.

Synthesis of (4*S*,10'*R*,11b*R*)-10'-(2-*Iso*propoxyphenyl)-8',9'-dimethylspiro[dinaphtho[2,1-*d*:1',2'-*f*][1,3,2]dioxaborepine-4,11'-pyrido[3',2':3,4][1,2]azaborolo[1,5-*a*]quinolin]-12'-ium-26-uide (10e)

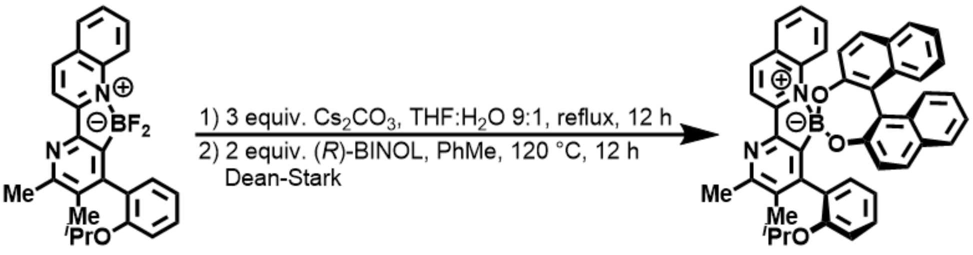

Following general procedure 2 with difluoroborane **9e** (158 mg, 0.38 mmol, 1 equiv.), Cs_2CO_3 (371 mg, 1.14 mmol, 3 equiv.) and (*R*)-BINOL (217 mg, 0.76 mmol, 2 equiv.). The residual crude was purified by column chromatography on silica gel using a mixture of hexane:Et_2O 1:1 as eluent to afford the product as an amorphous yellow solid (225 mg, 89%, 7:1 *d.r.*).

M.P. 176–178 °C (decomposition) recrystallized from EtOAc.

^{1}H NMR (400 MHz, C_6D_6, major diastereomer): δ 8.30 (d, *J* = 8.5 Hz, 1H), 8.11 (d, *J* = 9.0 Hz, 1H), 7.70 – 7.64 (m, 3H), 7.58 (t, *J* = 8.5 Hz, 1H), 7.47 (d, *J* = 8.5 Hz, 1H), 7.39 – 7.18 (m, 7H), 7.13 – 7.02 (m, 2H), 6.97 (d, *J* = 8.5 Hz, 1H), 6.94 (d, *J* = 8.5 Hz, 1H), 6.60 (t, *J* = 7.5 Hz, 1H), 6.37 – 6.30 (m, 1H), 6.27 (t, *J* = 8.0 Hz, 1H), 5.91 (t, *J* = 7.5 Hz, 1H), 4.28 (hept, *J* = 6.0 Hz, 1H), 2.61 (s, 3H), 2.01 (s, 3H), 1.16 (d, *J* = 6.0 Hz, 3H), 1.12 (d, *J* = 6.0 Hz, 3H).

^{13}C NMR (101 MHz, C_6D_6, major diastereomer): δ 158.5, 157.3, 156.3, 156.2, 154.3, 153.0, 150.4, 143.3, 141.4, 134.9, 134.5, 134.3, 132.7, 131.3, 131.2, 131.0, 130.9, 130.7, 129.7, 129.6, 129.4, 129.3, 129.1, 126.7, 126.4, 125.7, 125.1, 124.5, 124.2, 124.1, 123.6, 123.4, 123.2, 119.4, 115.8, 112.2, 69.1, 23.7, 22.8, 21.7, 17.1.

^{11}B NMR (128 MHz, C_6D_6): δ 14.3 (s, br).

HRMS: (ESI) $[M+H]^+$ *m/z* calculated for $C_{45}H_{36}BN_2O_3$ 663.2813, found 663.2809.

AR–FTIR (neat, cm^{-1}): 2975, 2278, 1593, 1226.

Synthesis of (4*S*,10'*R*,11b*R*)-10'-(2-(dimethylamino)phenyl)-8',9'-dimethylspiro[dinaphtho[2,1-*d*:1',2'-*f*][1,3,2]dioxaborepine-4,11'-pyrido[3',2':3,4][1,2]azaborolo[1,5-*a*]quinolin]-12'-ium-26-uide (10f)

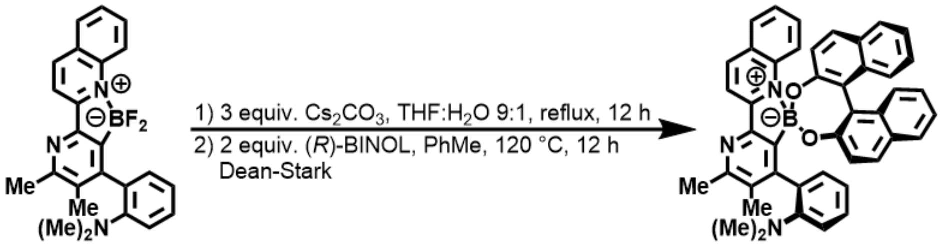

Following general procedure 2 with difluoroborane **9f** (100 mg, 0.25 mmol, 1 equiv.), Cs_2CO_3 (246 mg, 0.75 mmol, 3 equiv.) and (*R*)-BINOL (143 mg, 0.50 mmol). The residual crude was purified by column chromatography on silica gel using a mixture of hexane:Et_2O 1:1 as eluent to afford the product as an amorphous yellow solid (103 mg, 64%, 6:1 *d.r.*).

^{1}H NMR (400 MHz, C_6D_6, major diastereomer): δ 8.44 (d, *J* = 8.5 Hz, 1H), 8.18 (d, *J* = 9.0 Hz, 1H), 7.79 – 7.70 (m, 3H), 7.66 (d, *J* = 8.5 Hz, 1H), 7.61 (d, *J* = 8.0 Hz, 1H), 7.38 (d, *J* = 8.5 Hz, 1H), 7.33 – 7.27 (m, 2H), 7.23 – 7.18 (m, 2H), 7.13 – 7.05 (m, 2H), 7.04 (d, *J* = 8.5 Hz, 1H), 6.95 (dd, *J* = 8.5, 1.0 Hz, 1H), 6.74 (d, *J* = 8.5 Hz, 1H), 6.71 – 6.66 (m, 1H), 6.66 – 6.56 (m, 2H), 6.20 (ddd, *J* = 8.5, 7.0, 1.5 Hz, 1H), 5.75 (td, *J* = 7.5, 1.0 Hz, 1H), 2.62 (s, 3H), 2.56 (s, 6H), 1.97 (s, 3H).

^{13}C NMR (101 MHz, C_6D_6, major diastereomer): δ 158.5, 158.0, 156.4, 156.1, 153.6, 153.2, 152.4, 150.8, 143.4, 141.9, 134.6, 134.5, 134.2, 132.8, 131.3, 130.7, 130.6, 129.8, 129.3, 128.8, 126.3, 125.9, 125.8, 125.3, 124.9, 124.0, 123.8, 123.6, 123.3, 121.1, 118.5, 116.8, 115.8, 43.7, 23.8, 16.7. (*A complete assignment could not be achieved due to a large number of overlapping signals*).

^{11}B NMR (128 MHz, C_6D_6): δ 15.0 (s, br).

HRMS: (ESI) *m/z* $[M+H]^+$ calculated for $C_{44}H_{35}BN_3O_2$ 648.2817, found 648.2823.

AR–FTIR (neat, cm^{-1}): 3062, 2960, 2929, 1593, 1431, 1343, 1253, 1230, 1082.

Synthesis of (4*S*,10'*R*,11b*R*)-8',9'-Dimethyl-10'-(2-(methylthio)phenyl)spiro[dinaphtho[2,1-*d*:1',2'-*f*][1,3,2]dioxaborepine-4,11'-pyrido[3',2':3,4][1,2]azaborolo[1,5-*a*]quinolin]-12'-ium-26-uide (10g)

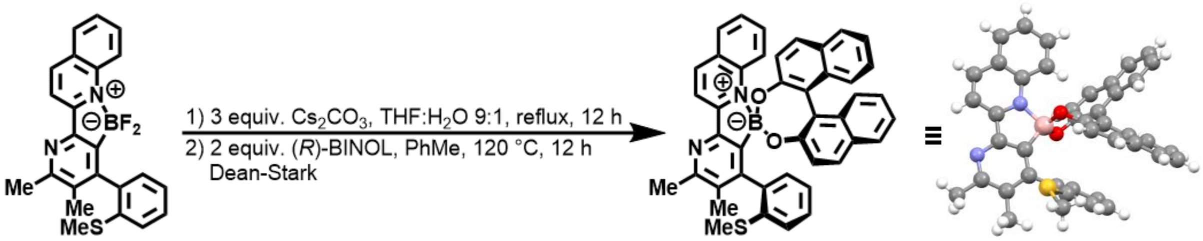

Following general procedure 2 with difluoroborane **9g** (50 mg, 0.12 mmol, 1 equiv.), Cs_2CO_3 (121 mg, 0.37 mmol, 3 equiv.) and (*R*)-BINOL (71 mg, 0.25 mmol, 2 equiv.). The product was slowly precipitated from MeCN and filtered to afford it as a yellow solid (54 mg, 67%, >98:2 *d.r.*). The product was further recrystallized from benzene.

$[\alpha]_D^{25}$ = –570 [1.0 *c* in $CHCl_3$].

M.P. 164–166 °C (decomposition) recrystallized from benzene.

^{1}H NMR (400 MHz, C_6D_6, single diastereomer): δ 8.30 (d, *J* = 8.5 Hz, 1H), 8.08 (d, *J* = 9.0 Hz, 1H), 7.70 – 7.59 (m, 3H), 7.55 (d, *J* = 8.5 Hz, 1H), 7.46 (t, *J* = 8.0 Hz, 1H), 7.33 (m, 3H), 7.27 (t, *J* = 8.0 Hz, 1H), 7.22 – 7.17 (m, 2H), 7.08 (t, *J* = 7.5 Hz, 1H), 7.03 (t, *J* = 7.0 Hz, 1H), 6.93 (m, 2H), 6.60 (t, *J* = 7.5 Hz, 1H), 6.50 (d, *J* = 8.0 Hz, 1H), 6.26 (m, 2H), 6.11 (t, *J* = 7.0 Hz, 1H), 2.58 (s, 3H), 2.06 (s, 3H), 2.04 (s, 3H).

^{13}C NMR (101 MHz, C_6D_6, single diastereomer): δ 158.3, 158.1, 156.2, 155.9, 153.4, 150.8, 143.3, 141.5, 137.8, 136.9, 134.8, 134.4, 134.2, 131.1, 130.8, 129.4, 129.3, 129.2, 127.2, 126.4, 125.7, 125.0, 124.1, 124.0, 123.8, 123.7, 123.6, 123.4, 123.1, 121.4, 115.7, 23.7, 16.6, 14.7 (*Some signals are overlapped with the solvent signal*).

^{11}B NMR (128 MHz, C_6D_6): δ 14.0 (s, br).

HRMS: (ESI) $[M+H]^+$ *m/z* calculated for $C_{43}H_{32}BN_2O_2S$ 651.2272, found 651.2299.

AR–FTIR (neat, cm^{-1}): 2920, 2852, 1590, 1465, 1252.

Synthesis of (4*S*,11b*R*)-9'-(2-Ethylphenyl)-10',11'-dimethylspiro[dinaphtho[2,1-*d*:1',2'-*f*][1,3,2]dioxaborepine-4,8'-pyrido[3',2':3,4][1,2]azaborolo[5,1-*a*]isoquinolin]-7'-ium-23-uide (10h)

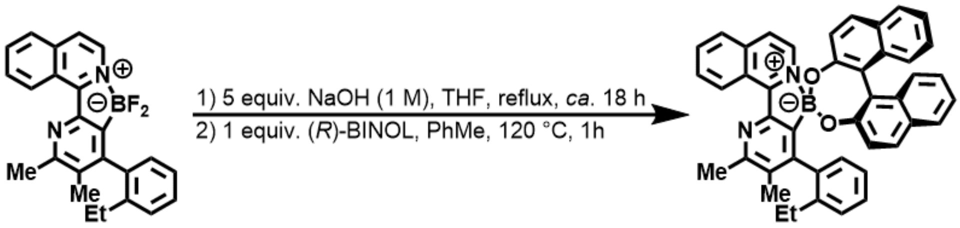

Following general procedure 1, difluoroborane **9h** (80 mg, 0.21 mmol, 1 equiv.), and (*R*)-BINOL (59 mg, 0.21 mmol, 1 equiv.) were combined to give the crude product. The residue was purified by column chromatography on silica gel with a mixture of petrol:EtOAc 7.5:2.5 as eluent to afford the title compound as a 1:1 mixture of diastereomers (120 mg, 92%).

^{1}H NMR (400 MHz, $CDCl_3$): δ 10.72 (d, *J* = 8.0 Hz, 2H), 7.95 – 7.84 (m, 8H), 7.83 – 7.74 (m, 2H), 7.73 – 7.68 (m, 2H), 7.56 – 7.46 (m, 3H), 7.45 – 7.37 (m, 2H), 7.37 – 7.27 (m, 5H), 7.25 – 6.99 (m, 10H), 6.92 (d, *J* = 8.5 Hz, 1H), 6.84 (d, *J* = 8.5 Hz, 2H), 6.73 (d, *J* = 7.5 Hz, 1H), 6.66 (d, *J* = 8.5 Hz, 1H), 6.61 – 6.39 (m, 4H), 5.97 (t, *J* = 7.5 Hz, 1H), 2.74 (s, 3H), 2.74 (s, 3H), 2.56 – 2.39 (m, 2H), 2.28 (q, *J* = 7.5 Hz, 2H), 2.00 (s, 3H), 1.95 (s, 3H), 1.30 (d, *J* = 7.5 Hz, 3H), 0.74 (t, *J* = 7.5 Hz, 3H).

^{13}C NMR (101 MHz, $CDCl_3$): δ 157.5, 157.4, 155.8, 155.3, 154.6 (x 2C), 154.5, 154.4, 154.2, 152.7, 151.9, 141.1, 139.8, 139.6, 139.5, 138.3 (x 2C), 133.8, 133.7, 133.5 (x 2C), 133.2 (x 2C), 133.1 (x 2C), 132.9, 132.8, 130.5, 130.2, 130.1, 130.0, 129.9, 129.6, 129.5, 129.4, 129.1, 128.8, 128.7, 128.4, 128.1, 128.0, 127.8, 127.7, 127.4, 127.3, 127.2, 127.1, 127.0, 126.9, 126.8, 126.7, 126.6 (x 2C), 125.6, 125.5, 125.3, 125.2, 125.0, 124.7, 124.6, 124.5, 123.6, 123.5, 123.4, 123.3, 123.2, 123.1, 122.8, 122.7, 122.6, 122.2, 121.9, 121.6, 120.6, 120.5, 25.8, 25.7, 24.1, 24.0, 16.8, 16.7, 14.2, 14.1.

^{11}B NMR (128 MHz, $CDCl_3$): δ 11.0 (s, br).

HRMS: (ESI) *m/z* $[M+H]^+$ calculated for $C_{44}H_{34}BN_2O_2$ 633.2708, found 633.2710.

AR–FTIR (neat, cm^{-1}): 3055, 2963, 2934, 1593, 1505, 1466, 1339, 1254, 1098, 1023.

Synthesis of (4*S*,9'*R*,11b*R*)-9'-(2-Methoxyphenyl)-10',11'-dimethylspiro[dinaphtho[2,1-*d*:1',2'-*f*][1,3,2]dioxaborepine-4,8'-pyrido[3',2':3,4][1,2]azaborolo[5,1-*a*]isoquinolin]-7'-ium-23-uide (10i)

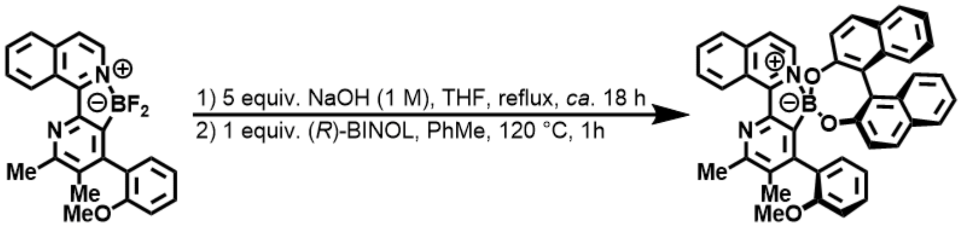

Following general procedure 1, difluoroborane **9i** (70 mg, 0.18 mmol, 1 equiv.), and (*R*)-BINOL (52 mg, 0.18 mmol, 1 equiv.) were combined to give the crude product**.** The residue was purified by column chromatography on silica gel using a mixture of petrol:EtOAc 7.5:2.5 as eluent to afford the title compound as a 4:1 mixture of diastereomers (89 mg, 78%).

^{1}H NMR (400 MHz, $CDCl_3$, major diastereomer): δ 10.72 (d, *J* = 7.5 Hz, 1H), 7.94 – 7.82 (m, 4H), 7.77 (d, *J* = 9.0 Hz, 1H), 7.72 (d, *J* = 8.0 Hz, 1H), 7.52 – 7.42 (m, 2H), 7.42 – 7.27 (m, 3H), 7.24 – 7.07 (m, 5H), 6.98 (d, *J* = 8.5 Hz, 1H), 6.77 (d, *J* = 8.5 Hz, 1H), 6.54 (d, *J* = 8.0 Hz, 1H), 6.43 (t, *J* = 7.5 Hz, 1H), 5.78 (t, *J* = 7.5 Hz, 1H), 3.86 (s, 3H), 2.73 (s, 3H), 2.02 (s, 3H).

^{13}C NMR (101 MHz, $CDCl_3$, major diastereomer): δ 157.0, 155.8, 155.7, 154.8, 154.7, 154.6, 149.4, 139.5, 134.0, 133.7, 133.6, 133.2, 133.1, 132.1, 130.6, 130.2, 130.1, 129.5, 129.4, 128.8, 128.2, 128.1, 127.8, 127.7, 127.4, 127.0, 126.9, 125.4, 125.2, 124.7, 123.6, 123.4, 123.0, 122.8, 122.3, 121.7, 121.0, 119.2, 109.8, 55.1, 24.1, 16.5.

^{11}B NMR (128 MHz, $CDCl_3$): δ 11.3 (s, br).

HRMS: (ESI) *m/z* $[M+H]^+$ calculated for $C_{43}H_{32}BN_2O_3$ 635.2506, found 635.2496.

AR–FTIR (neat, cm^{-1}): 3054, 3002, 2953, 1593, 1551, 1339, 1253, 1078, 1024.

Synthesis of (4*S*,11b*R*)-9'-(2-ethylphenyl)-10',11'-diphenylspiro[dinaphtho[2,1-*d*:1',2'-*f*][1,3,2]dioxaborepine-4,8'-pyrido[3',2':3,4][1,2]azaborolo[5,1-*a*]isoquinolin]-7'-ium-23-uide (10j)

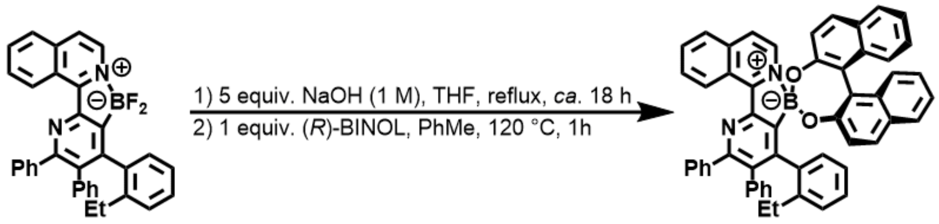

Following general procedure 1, difluoroborane **9j** (70 mg, 0.14 mmol, 1 equiv.), and (*R*)-BINOL (39 mg, 0.14 mmol, 1 equiv.) were combined to give the crude product. The residue was purified by column chromatography on silica gel using a mixture of petrol:EtOAc 7.5:2.5 as eluent to afford the title compound as a 1:1 mixture of diastereomers (59 mg, 57%).

^{1}H NMR (400 MHz, C_6D_6): δ 10.76 (d, *J* = 8.5 Hz, 2H), 7.83 (d, *J* = 7.5 Hz, 1H), 7.81 – 7.58 (m, 11H), 7.57 – 7.48 (m, 4H), 7.47 – 7.39 (m, 2H), 7.33 (d, *J* = 8.5 Hz, 1H), 7.29 – 7.17 (m, 8H), 7.15 – 6.95 (m, 20H), 6.94 – 6.88 (m, 2H), 6.87 – 6.77 (m, 2H), 6.76 – 6.64 (m, 5H), 6.52 (t, *J* = 7.5 Hz, 1H), 6.44 – 6.41 (m, 3H), 6.38 (d, *J* = 6.0 Hz, 1H), 6.24 (t, *J* = 7.5 Hz, 1H), 3.04 – 2.92 (m, 1H), 2.64 – 2.51 (m, 1H), 2.45 – 2.34 (m, 1H), 2.30 – 2.17 (m, 1H), 1.06 – 0.94 (m, 6H).

^{13}C NMR (101 MHz, C_6D_6): δ 158.5, 158.4, 158.2, 157.8, 155.9, 155.8, 155.4, 155.3, 154.2, 153.8, 153.2, 142.4, 142.2, 141.1, 139.6, 139.5 (x 2C), 139.1, 138.6, 138.4, 138.3, 137.6, 134.4, 134.3, 134.1, 133.9, 133.7, 133.6, 133.3, 133.2, 132.3 (x 2C), 132.0, 130.8, 130.7, 130.6 (x 2C), 130.5, 130.4, 130.3, 130.1, 129.9, 129.6, 129.5, 129.2, 129.1, 127.5, 127.4, 127.3, 127.1, 126.9 (x 2C), 126.7, 126.6, 126.1, 126.0, 125.9, 125.8, 125.7, 125.6, 125.3, 125.2, 124.6, 124.4, 124.2, 124.1, 124.0, 123.9, 123.8, 123.7, 123.6, 123.2, 123.1 (x 2C), 122.4, 122.1, 120.9, 120.8, 26.1, 26.0, 13.9, 13.5.

^{11}B NMR (128 MHz, C_6D_6): δ 12.0 (s, br).

HRMS: (ESI) *m/z* $[M+Na]^+$ calculated for $C_{54}H_{38}BN_2NaO_2$ 779.2846, found 779.2846.

AR–FTIR (neat, cm^{-1}): 3055, 2963, 2934, 1593, 1505, 1466, 1339, 1254, 1098, 1023.

Synthesis of (4*S*,9'*R*,11b*R*)-9'-(2-Methoxyphenyl)-10'-11'-diphenylspiro[dinaphto[2,1-*d*:1',2'-*f*][1,3,2]dioxaborepine-4,8'-pyrido[3',2':3,4][1,2]azaborolo[5,1-*a*]isoquinolin]-7'-ium-23-uide (10k)

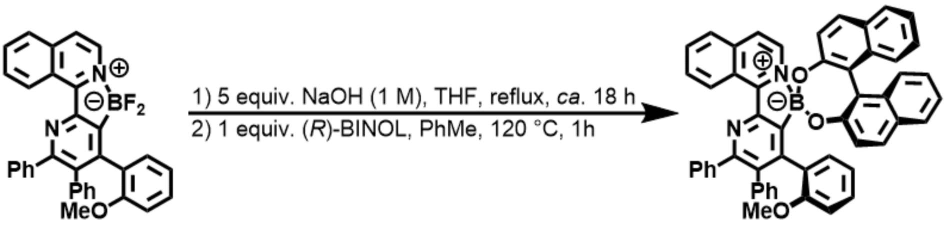

Following general procedure 1, difluoroborane **9k** (100 mg, 0.2 mmol, 1 equiv.), and (*R*)-BINOL (56 mg, 0.2 mmol, 1 equiv.) were combined to give the crude product. The residue was purified by column chromatography on silica gel using a mixture of petrol:EtOAc 7.5:2.5 as eluent to afford the title compound as a 3:1 mixture of diastereomers (124 mg, 84%).

^{1}H NMR (400 MHz, $CDCl_3$, mixture of diastereomers 3:1): δ 10.74 – 10.65 (m, 4H), 7.99 – 7.69 (m, 21H), 7.65 (d, *J* = 8.0 Hz, 3H), 7.58 – 7.46 (m, 9H), 7.45 – 7.23 (m, 36H), 7.23 – 7.10 (m, 15H), 7.05 – 6.78 (m, 20H), 6.77 – 6.60 (m, 7H), 6.54 (dd, *J* = 7.5, 1.5 Hz, 1H), 6.51 – 6.44 (m, 1H), 6.37 – 6.31 (m, 3H), 6.28 (t, *J* = 7.5 Hz, 1H), 6.11 (d, *J* = 8.0 Hz, 3H), 5.73 – 5.66 (m, 4H), 3.27 (s, 9H), 3.24 (s, 3H).

^{13}C NMR (101 MHz, $CDCl_3$, major diastereomer): δ 158.0, 157.8, 155.1, 154.8, 154.5, 154.0, 150.4, 141.7, 139.5, 138.9, 138.3, 133.9, 133.7, 133.3, 133.1, 132.4, 130.5, 130.4 (x 2C), 130.1 (x 2C), 130.0, 129.5, 129.4, 128.9, 128.4, 128.1, 127.7 (x 2C), 127.6 (x 2C), 127.5, 127.4, 127.3 (x 2C), 127.1, 126.9, 126.5, 126.2, 125.5, 125.3, 124.7, 123.5 (x 2C), 123.3, 122.8, 122.4, 122.1, 120.8, 118.8, 109.5, 54.4.

^{11}B NMR (128 MHz, $CDCl_3$): δ 11.4 (s, br).

HRMS: (ESI) *m/z* $[M+H]^+$ calculated for $C_{53}H_{36}BN_2O_3$ 759.2819, found 759.2844.

AR–FTIR (neat, cm^{-1}): 3056, 2966, 2932, 1593, 1540, 1505, 1339, 1253, 1099.

Synthesis of (4*S*,4'*R*,11b'*R*)-4-(2-Methoxyphenyl)-2,3-dimethylspiro[[1,2]azaborolo[1,5-*a*:4,3-*b*']dipyridine-5,4'-dinaphto[2,1-*d*:1',2'-*f*][1,3,2]dioxaborepin]-6-ium-14-uide (10l)

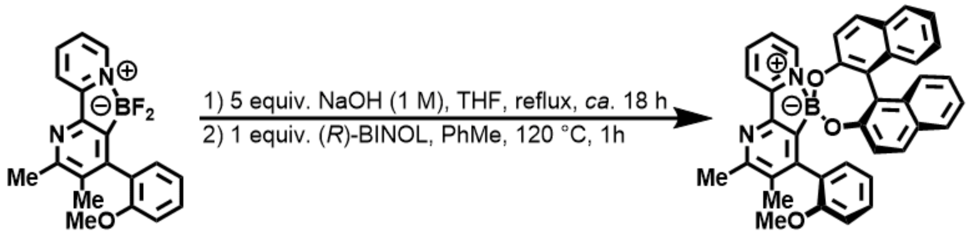

Following general procedure 1, difluoroborane **9l** (60 mg, 0.18 mmol, 1 equiv.), and (*R*)-BINOL (51 mg, 0.18 mmol, 1 equiv.) were combined to give the crude product. The residue was purified by column chromatography on silica gel using a mixture of petrol:EtOAc 6:4 as eluent to afford the title compound as a 5.6:1 mixture of diastereomers (60 mg, 58%).

^{1}H NMR (400 MHz, C_6D_6, major diastereomer): δ 7.98 – 7.92 (m, 1H), 7.79 – 7.72 (m, 2H), 7.69 – 7.62 (m, 2H), 7.61 – 7.57 (m, 2H), 7.38 – 7.33 (m, 1H), 7.33 – 7.26 (m, 2H), 7.22 – 7.18 (m, 1H), 7.13 – 7.07 (m, 3H), 7.04 – 6.98 (m, 1H), 6.79 – 6.73 (m, 1H), 6.45 (t, *J* = 8.0 Hz, 1H), 6.31 (d, *J* = 8.0 Hz, 1H), 5.94 (t, *J* = 7.5 Hz, 1H), 5.87 (t, *J* = 7.5 Hz, 1H), 3.43 (s, 3H), 2.56 (s, 3H), 1.98 (s, 3H).

^{13}C NMR (101 MHz, C_6D_6, major diastereomer): δ 157.4, 156.3, 156.2, 155.8, 155.3, 153.0, 150.4, 142.5, 141.7, 134.4, 134.2, 133.9, 132.6, 130.7, 130.6, 129.8, 129.0, 128.6, 128.5, 128.4, 127.5, 125.9, 125.2, 124.0, 123.9, 123.8, 123.1, 123.0, 122.5, 121.5, 119.8, 118.1, 110.2, 54.7, 23.6, 16.5 (*A complete assignment could not be achieved due to a large number of overlapping signals*).

^{11}B NMR (128 MHz, C_6D_6): δ 12.2 (s, br).

HRMS: (ESI) *m/z* $[M+H]^+$ calculated for $C_{39}H_{30}BN_2O_3$ 585.2344, found 585.2358.

AR–FTIR (neat, cm^{-1}): 3056, 2995, 2924, 2832, 1624, 1492, 1466, 1339, 1252, 1076.

Synthesis of (10'*R*,11b*R*)-2,6-Bis(cyanomethyl)-10'-(2-methoxyphenyl)-8',9'-dimethylspiro[dinaphtho[2,1-*d*:1',2'-*f*][1,3,2]dioxaborepine-4,11'-pyrido[3',2':3,4][1,2]azaborolo[1,5-*a*]quinolin]-12'-ium-26-uide (10m)

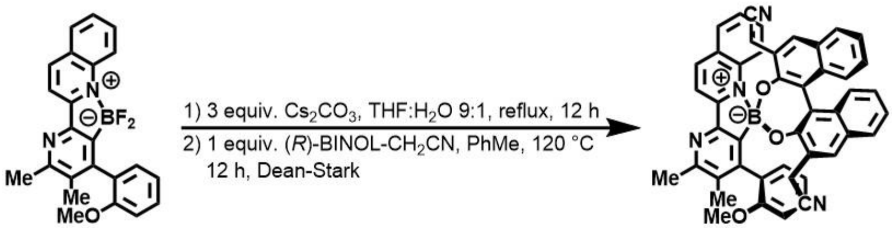

Following general procedure 2 with difluoroborane **9b** (53 mg, 0.14 mmol, 1 equiv.), Cs_2CO_3 (133 mg, 0.40 mmol, 3 equiv.) and (*R*)-BINOL-CH_2CN (50 mg, 0.13 mmol, 1 equiv.). The residue was purified by column chromatography on silica gel with a mixture of petrol:EtOAc 7:3 as eluent to afford the product as an amorphous yellow solid (82 mg, 84%, >98:2 *d.r.*).

^{1}H NMR (400 MHz, $CDCl_3$): δ 8.69–8.59 (m, 2H), 7.88 (d, *J* = 8.0 Hz, 1H), 7.83 (d, *J* = 8.0 Hz, 1H), 7.76 (d, *J* = 7.5 Hz, 1H), 7.72 (s, 1H), 7.65–7.54 (m, 2H), 7.42–7.33 (m, 3H), 7.23–7.12 (m, 3H), 7.10–7.01 (m, 2H), 6.70 (t, *J* = 8.0 Hz, 1H), 6.40 (d, *J* = 7.5 Hz, 1H), 6.26 (t, *J* = 8.0 Hz, 1H), 5.77 (t, *J* = 7.0 Hz, 1H), 3.89 (s, 3H), 3.67 (d, *J* = 19.0 Hz, 1H), 3.58–3.42 (m, 2H), 2.85 (d, *J* = 19.0 Hz, 1H), 2.69 (s, 3H), 1.94 (s, 3H).

^{13}C NMR (101 MHz, $CDCl_3$): δ 158.2, 158.0, 155.3, 152.3, 152.1, 149.5, 144.5, 140.7, 135.4, 133.4, 133.1, 131.9, 131.8, 130.1, 129.6, 129.4, 128.8, 128.1, 128.0, 127.9, 127.8, 127.4, 127.1, 127.0, 126.9, 126.8, 125.9, 125.2, 124.2, 123.9, 123.6, 123.3, 122.5, 121.1, 119.1, 118.9, 117.7, 115.9, 109.6, 55.5, 23.9, 20.3, 19.2, 16.6.

^{11}B NMR (128 MHz, $CDCl_3$): δ 12.7.

HRMS: (ESI) $[M+H]^+$ calculated for $C_{47}H_{33}BN_4O_3$ 713.2718, found 713.2239.

AR–FTIR (neat, cm^{-1}): 3062, 2999, 2920, 2836, 2253, 1598, 1522, 1428, 1342, 1260, 1243, 1066, 950, 749.

Synthesis of (10'*R*,11b*R*)-2,6-Bis(cyanomethyl)-10'-(2-isopropoxyphenyl)-8',9'-dimethylspiro[dinaphtho[2,1-*d*:1',2'-*f*][1,3,2]dioxaborepine-4,11'-pyrido[3',2':3,4][1,2]azaborolo[1,5-*a*]quinolin]-12'-ium-26-uide (10n)

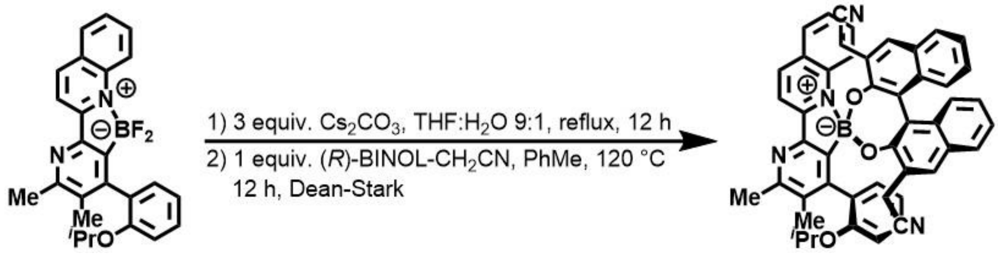

Following general procedure 2 with difluoroborane **9e** (158 mg, 0.38 mmol, 1 equiv.), Cs_2CO_3 (371 mg, 1.14 mmol, 3 equiv.) and (*R*)-BINOL-CH_2CN (138 mg, 0.38 mmol, 1 equiv.). The residual crude was purified by column chromatography on silica gel using a mixture of petrol:EtOAc 7:3 as eluent to afford the product as an amorphous yellow solid (202 mg, 72%, >98:2 *d.r.*).

^{1}H NMR (400 MHz, $CDCl_3$): δ 8.66–8.58 (m, 2H), 7.85 (d, *J* = 8.0 Hz, 1H), 7.83–7.72 (m, 2H), 7.64 (s, 1H), 7.60 (d, *J* = 9.0 Hz, 1H), 7.55 (s, 1H), 7.42–7.35 (m, 2H), 7.31 (t, *J* = 7.5 Hz, 1H), 7.22–7.15 (m, 3H), 7.15–7.09 (m, 2H), 6.60–6.48 (m, 2H), 6.33 (t, *J* = 8.0 Hz, 1H), 5.55 (t, *J* = 7.5 Hz, 1H), 4.80–4.70 (m, 1H), 3.73 (d, *J* = 19.0 Hz, 1H), 3.57–3.38 (m, 2H), 2.77–2.63 (m, 4H), 2.01 (s, 3H), 1.41 (d, *J* = 6.0 Hz, 3H), 1.32 (d, *J* = 6.0 Hz, 3H).
^{13}C NMR (101 MHz, $CDCl_3$): δ 158.1, 158.0, 153.4, 152.4, 152.3, 152.1, 149.5, 144.4, 140.9, 135.3, 133.5, 133.3, 132.7, 131.6, 130.0, 129.8, 129.3, 128.6, 128.4, 128.0, 127.9, 127.8, 127.6, 127.5, 127.3, 127.0, 126.9, 126.0, 125.3, 124.3, 123.9, 123.5, 123.2, 122.9, 122.4, 120.7, 118.9, 118.6, 117.7, 115.8, 111.1, 68.8, 23.9, 23.5, 20.8, 20.0, 18.9, 17.7.
^{11}B NMR (128 MHz, $CDCl_3$): δ 12.9.
HRMS: (ESI) $[M+H]^+$ calculated for $C_{49}H_{37}BN_4O_3$ 741.3031, found 741.3037.
AR–FTIR (neat, cm^{-1}): 3061, 2978, 2923, 2252, 1597, 1429, 1360, 1343, 1260, 1220, 1108, 1065, 949, 749.

Synthesis of (9'*R*,11b*R*)-2,6-Bis(cyanomethyl)-9'-(2-methoxyphenyl)-10',11'-diphenylspiro[dinaphtho[2,1-*d*:1',2'-*f*][1,3,2]dioxaborepine-4,8'-pyrido[3',2':3,4][1,2]azaborolo[5,1-*a*]isoquinolin]-7'-ium-23-uide (10o)

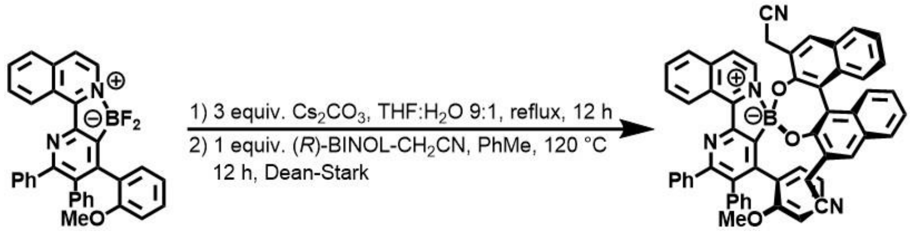

Following general procedure 2 with difluoroborane **9k** (102 mg, 0.2 mmol, 1 equiv.), Cs_2CO_3 (194 mg, 0.6 mmol, 3 equiv.) and (*R*)-BINOL-CH_2CN (73 mg, 0.2 mmol, 1 equiv.) were combined to give the crude product. The residue was purified chromatographically over silica gel using a mixture of petrol:EtOAc 7.5:2.5 as eluent to afford the product as an amorphous yellow solid (100 mg, 60%, >98:2 *d.r.*).

^{1}H NMR (400 MHz, $CDCl_3$): δ 10.69 (d, *J* = 8.0 Hz, 1H), 8.04–7.89 (m, 5H), 7.70 (d, *J* = 7.0 Hz, 1H), 7.65 (d, *J* = 6.5 Hz, 1H), 7.53 (d, *J* = 6.5 Hz, 1H), 7.46–7.39 (m, 4H), 7.34 (t, *J* = 7.5 Hz, 2H), 7.32–7.27 (m, 3H), 7.24–7.15 (m, 3H), 7.12 (d, *J* = 7.5 Hz, 1H), 7.06–6.78 (m, 4H), 6.71–6.57 (m, 1H), 6.37 (t, *J* = 8.0 Hz, 1H), 6.23 (d, *J* = 7.5 Hz, 1H), 5.55 (t, *J* = 7.0 Hz, 1H), 3.72 (d, *J* = 19.5 Hz, 1H), 3.61 (s, 2H), 3.22 (s, 3H), 2.99 (d, *J* = 19.5 Hz, 1H).
^{13}C NMR (101 MHz, $CDCl_3$): δ 158.2, 157.6, 154.7, 153.8, 151.9, 151.6, 150.1, 141.4, 139.8, 138.6, 138.4, 134.6, 133.3, 132.9, 132.8, 131.9, 130.6, 130.4, 130.3 (x 2C), 129.8, 129.7, 129.1, 128.2, 127.8 (x 3C), 127.6, 127.4, 127.2, 127.1, 127.0, 126.7 (x 2C), 126.5, 126.4, 126.0, 125.5, 125.3, 124.5, 123.7, 123.5, 123.4, 123.1, 122.9, 120.6, 119.1, 118.9, 118.1, 109.9, 55.2, 20.4, 19.4.
^{11}B NMR (128 MHz, $CDCl_3$): δ 11.1.
HRMS: (ESI) $[M+H]^+$ calculated for $C_{57}H_{37}BN_4O_3$ 837.3031, found 837.3049.
AR–FTIR (neat, cm^{-1}): 3058, 2923, 2853, 2254, 1710, 1454, 1430, 1360, 1261, 1106, 1094, 1022, 985, 750, 701.

Synthesis of 4-(2-Methoxyphenyl)-5,6-dimethyl-2-(quinolin-2-yl)pyridin-3-ol (11)

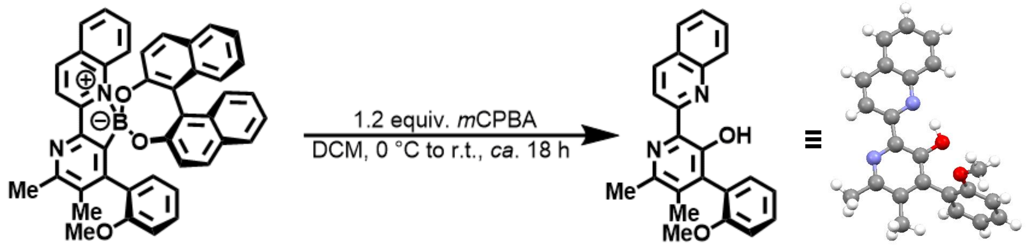

(4*S*,10'*R*,11b*R*)-10'-(2-Methoxyphenyl)-8',9'-dimethylspiro[dinaphtho[2,1-*d*:1',2'-*f*][1,3,2]dioxaborepine-4,11'-pyrido[3',2':3,4][1,2]azaborolo[1,5-*a*]quinolin]-12'-ium-26-uide (**10b**) (40 mg, 0.06 mmol, 1 equiv., 5:1 *d.r.*), was dissolved in DCM (8 mL) at 0 °C, then 3-chloroperbenzoic acid (*m*CPBA, 17 mg, 0.10 mmol, 1.2 equiv.) was added portion-wise. The mixture was stirred under a nitrogen atmosphere at room temperature overnight (*ca*. 18 h). Then, saturated $NaHCO_3$ (*aq.*) (15 mL) was added and the mixture was extracted with DCM (3 x 15 mL). The organic extract was dried over $MgSO_4$, filtered, and evaporated to dryness *in vacuo*. The residue was purified by column chromatography on silica gel with a gradient of elution of petrol:EtOAc 19:1 → 2:1 to afford the product as a yellow solid (10.8 mg, 48% yield, 84% *ee*, 92:8 *e.r.*).

$[\alpha]_D^{21.5}$ = +65 [1.0 *c* in $CHCl_3$].

M.P. 182 – 184 °C recrystallized from DCM.

^{1}H NMR (400 MHz, $CDCl_3$): δ 15.11 (br, 1H), 8.85 (d, *J* = 9.0 Hz, 1H), 8.29 (d, *J* = 9.0 Hz, 1H), 7.92 (d, *J* = 8.5 Hz, 1H), 7.83 (d, *J* = 8.0 Hz, 1H), 7.67 (t, *J* = 7.5, 1H), 7.52 (t, *J* = 8.0 Hz, 1H), 7.44 (dt, *J* = 8.0, 2.0 Hz, 1H), 7.23 (dd, *J* = 7.5, 2.0 Hz, 1H), 7.13 (t, *J* = 7.5, Hz, 1H), 7.08 (d, *J* = 8.0, Hz, 1H), 3.80 (s, 3H), 2.62 (s, 3H), 2.07 (s, 3H).

^{13}C NMR (101 MHz, $CDCl_3$): δ 159.3, 156.8, 154.0, 147.1, 144.6, 137.3, 135.7, 134.3, 132.8, 130.9, 130.2, 129.5, 127.8, 127.5, 127.4, 126.6, 124.7, 120.9, 118.9, 111.4, 55.8, 23.2, 16.8.

HRMS: (ESI) *m/z* $[M+H]^+$ calculated for $C_{23}H_{21}N_2O_2$ 357.1598, found 357.1600.

AR–FTIR (neat, cm^{-1}): 3014, 2934, 1594, 1496, 1459, 1420, 1243, 1184, 1113, 842, 761.

Chiral HPLC: The *ee* was determined by HPLC using a Chiralpak IA column [*n*-hexane/^{i}PrOH (99:1)]; τ_{major} = 20.67 min, τ_{minor} = 24.35 min (91.7:8.3).

Synthesis of 4-(2-Isopropoxyphenyl)-5,6-dimethyl-2-(quinolin-2-yl)pyridin-3-ol (12)

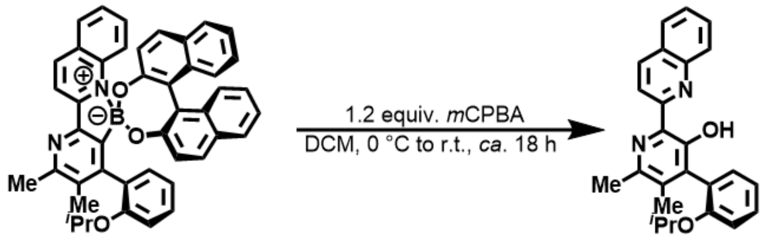

(4*S*,10'*R*,11b*R*)-10'-(2-*Iso*propoxyphenyl)-8',9'-dimethylspiro[dinaphtho[2,1-*d*:1',2'-*f*][1,3,2]dioxaborepine-4,11'-pyrido[3',2':3,4][1,2]azaborolo[1,5-*a*]quinolin]-12'-ium-26-uide (**10e**) (20 mg, 0.03 mmol, 7:1 *d.r.*), was dissolved in DCM (8 mL) at 0 °C, then 3-chloroperbenzoic acid (*m*CPBA, 7 mg, 0.04 mmol, 1.2 equiv.) was added portion-wise. The mixture was stirred under a nitrogen atmosphere at room temperature (*ca*. 18 h). Then, saturated $NaHCO_3$ (*aq*.) (15 mL) was added and the mixture was extracted with DCM (3 x 15 mL). The organic extract was dried over $MgSO_4$, filtered, and evaporated to dryness *in vacuo*. The residue was purified by column chromatography on silica gel with a gradient of elution of petrol:EtOAc 19:1 → 2:1 to afford the product as a yellow solid (5.4 mg, 46% yield, 76% *ee*, 88:12 *e.r.*).

^{1}H NMR (400 MHz, $CDCl_3$): δ 15.03 (br. s, 1H), 8.85 (d, *J* = 9.0 Hz, 1H), 8.30 (d, *J* = 9.0 Hz, 1H), 7.93 (d, *J* = 9.0 Hz, 1H), 7.83 (d, *J* = 8.0 Hz, 1H), 7.68 (dt, *J* = 7.5, 1 Hz, 1H), 7.52 (t, *J* = 7.5 Hz, 1H), 7.38 (dt, *J* = 8.0, 2.0 Hz, 1H), 7.23 (dd, *J* = 7.5, 1.5 Hz, 1H), 7.10 (t, *J* = 7.5 Hz, 1H), 7.06 (d, *J* = 8.0, Hz, 1H), 4.48–4.37 (m, 1H), 2.61 (s, 3H), 2.08 (s, 3H), 1.20 (d, *J* = 6.0 Hz, 3H), 1.13 (d, *J* = 6.0 Hz, 3H).

^{13}C NMR (101 MHz, $CDCl_3$): δ 159.4, 155.4, 154.1, 147.0, 144.7, 137.3, 136.1, 134.3, 132.6, 131.3, 130.2, 129.3, 127.8, 127.5, 127.4, 126.6, 126.4, 121.0, 118.8, 115.3, 71.2, 23.2, 22.3, 22.2, 16.9.

HRMS: (ESI) *m/z* $[M+H]^+$ calculated for $C_{25}H_{25}N_2O_2$ 385.1911, found 385.1915.

AR–FTIR (neat, cm^{-1}): 2970, 2919, 1600, 1443, 1372, 1232, 1114, 947, 833.

Chiral HPLC: The *ee* was determined by HPLC using a Cellulose-1 column [*n*-hexane/^{i}PrOH (95:5)]; flow rate 1.0 mL/min; t_{major} = 5.37 min, t_{minor} = 6.36 min (87.6:12.4).

Synthesis of 5,6-Dimethyl-4-(2-(methylsulfonyl)phenyl)-2-(quinolin-2-yl)pyridin-3-ol (13)

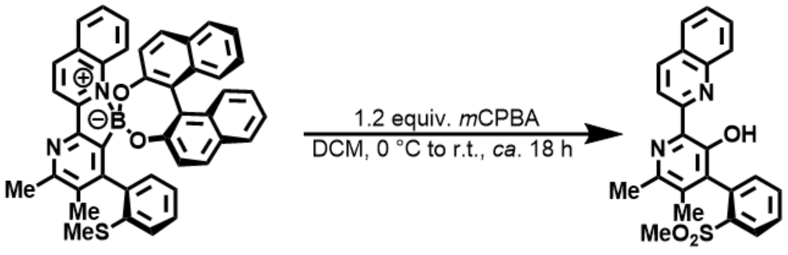

(4*S*,10'*R*,11b*R*)-8',9'-Dimethyl-10'-(2-(methylthio)phenyl)spiro[dinaphtho[2,1-*d*:1',2'-*f*][1,3,2]dioxaborepine-4,11'-pyrido[3',2':3,4][1,2]azaborolo[1,5-*a*]quinolin]-12'-ium-26-uide (**10g**) (64.9 mg, 0.1 mmol, 49:1 *d.r.*), was dissolved in DCM (5 mL) at 0 °C, then 3-chloroperbenzoic acid (*m*CPBA, 51.8 mg, 0.3 mmol, 1.2 equiv.) was added portion-wise. The mixture was stirred under a nitrogen atmosphere at room temperature (*ca*. 18 h). Then, saturated $NaHCO_3$ (*aq*.) (20 mL) was added and the mixture was extracted with DCM (3 x 30 mL). The organic extract was dried over $MgSO_4$, filtered, and evaporated to dryness *in vacuo*. The residue was purified by column chromatography on silica gel with a mixture of petrol:EtOAc 4:1 as eluent to afford the product as an amorphous yellow solid (29.1 mg, 72% yield, 78% *ee*, 89:11 *e.r.*).

^{1}H NMR (400 MHz, $CDCl_3$): δ 15.40 (br. s, 1H), 8.89 (d, *J* = 8.0 Hz, 1H), 8.35 (d, *J* = 9.0 Hz, 1H), 8.29 (d, *J* = 8.0 Hz, 1H), 7.90 (d, *J* = 8.5 Hz, 1H), 7.87 (d, *J* = 8.0 Hz, 1H), 7.78 (t, *J* = 7.5 Hz, 1H), 7.70 (t, *J* = 8.0 Hz, 1H), 7.67 (t, *J* = 7.5 Hz, 1H), 7.56 (t, *J* = 7.5 Hz, 1H), 7.32 (t, *J* = 7.5 Hz, 1H), 3.01 (s, 3H), 2.63 (s, 3H), 2.04 (s, 3H).

^{13}C NMR (101 MHz, $CDCl_3$): δ 152.9, 147.8, 144.4, 139.5, 137.8, 136.4, 135.4, 134.0, 132.7, 132.5, 131.8, 130.5, 129.8, 129.0, 128.0, 127.7, 127.3, 127.0, 119.0, 43.9, 22.9, 17.8.

HRMS: (ESI) *m/z* $[M+H]^+$ calculated for $C_{23}H_{21}N_2O_3S$ 405.1267, found 405.1281.

AR–FTIR (neat, cm^{-1}): 2971, 2920, 1738, 1466, 1301, 1259, 1149, 953, 845, 758.

Chiral HPLC: The *ee* was determined by HPLC using a Cellulose-1 column [*n*-hexane/^{i}PrOH (90:10)]; flow rate 1.0 mL/min; t_{major} = 14.67 min, t_{minor} = 21.42 min (88.6:11.4).

Total Synthesis of Streptonigrin

Synthesis of 6-iodo-2,3-dimethoxyphenol

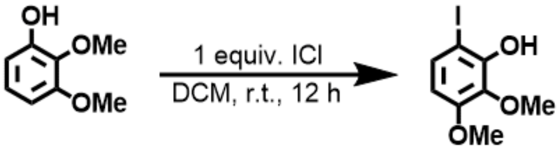

To a stirred solution of 2,3-dimethoxyphenol (5 mL, 38.4 mmol, 1 equiv.) in DCM (100 mL) at room temperature was added dropwise a commercial solution of iodine monochloride (1 M ICl in DCM, 38.4 mL, 38.4 mmol, 1 equiv.). The mixture was stirred for 12 h at room temperature in the dark. The reaction mixture was quenched with $Na_2S_2O_3$ (*aq.*) (100 mL) and washed with water (40 mL). The aqueous layer was extracted with DCM (2 x 50 mL). The organic extract was dried over $MgSO_4$, filtered, and evaporated to dryness *in vacuo*. The reaction crude was purified by column chromatography on silica gel with a mixture of DCM:petrol 1:1 as eluent to provide the product as a yellow solid (10.22 g, 95%).

M.P. 50–52 °C recrystallized from DCM.

^{1}H NMR (400 MHz, $CDCl_3$): δ 7.30 (d, *J* = 9.0 Hz, 1H), 6.35 (s, 1H), 6.30 (d, *J* = 9.0 Hz, 1H), 3.86 (s, 3H), 3.81 (s, 3H).

^{13}C NMR (101 MHz, $CDCl_3$): δ 152.9, 149.3, 135.5, 132.7, 106.5, 71.8, 61.1, 56.1.

HRMS: (ESI) *m/z* $[M+H]^+$ calculated for $C_8H_{10}IO_3$ 280.9675, found 280.9679.

AR–FTIR (neat, cm^{-1}): 1201, 1292, 1583, 2833, 2937, 3401.

Synthesis of 2-benzyloxy-1-iodo-2,3-dimethoxybenzene

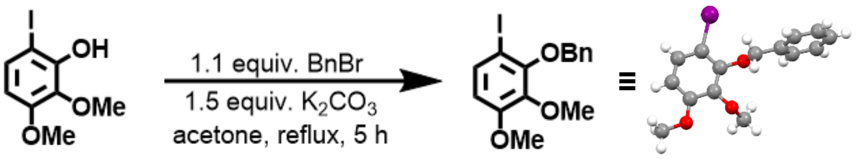

To a stirred solution of 6-iodo-2,3-dimethoxybenzene (10.2 g, 36.5 mmol, 1 equiv.) in acetone (170 mL) was added benzyl bromide (BnBr, 4.8 mL, 40.1 mmol, 1.1 equiv.) and potassium carbonate (K_2CO_3, 7.6 g, 54.8 mmol, 1.5 equiv.). Then, the mixture was stirred for 5 h at reflux. After cooling down to room temperature, the reaction mixture was filtered, and volatiles were evaporated *in vacuo*. The residue was redissolved in DCM (50 mL) and washed with water (50 mL) and brine (50 mL). The organic extract was dried over $MgSO_4$, filtered, and evaporated to

dryness *in vacuo*. Purification by column chromatography on silica gel with a mixture of petrol:EtOAc 95:5 as eluent afforded the product as a white solid (10.82 g, 80%).

M.P. 71–73 °C recrystallized from DCM.

^{1}H NMR (400 MHz, $CDCl_3$): δ 7.63 – 7.56 (m, 2H), 7.47 – 7.31 (m, 4H), 6.53 (d, *J* = 9.0 Hz, 1H), 5.06 (s, 2H), 3.89 (s, 3H), 3.86 (s, 3H).

^{13}C NMR (101 MHz, $CDCl_3$): δ 154.5, 152.3, 143.0, 137.0, 132.8, 128.7, 128.4, 128.2, 110.0, 81.9, 75.2, 61.2, 56.2.

HRMS: (ESI) *m/z* $[M+H]^+$ calculated for $C_{15}H_{16}IO_3$ 371.0144, found 371.0159.

AR–FTIR (neat, cm^{-1}): 1217, 1289, 1418, 1475, 1571, 2933.

Synthesis of (2-benzyloxy-2,3-dimethoxyphenylethynyl)trimethylsilane

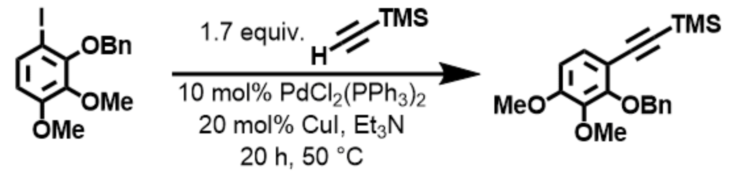

To a stirred suspension of 2-(benzyloxy)-1-iodo-2,3-dimethoxybenzene (10.3 g, 27.9 mmol, 1 equiv.), bis(triphenylphosphine)palladium(II) dichloride ($PdCl_2(PPh_3)_2$, 1.96 g, 2.79 mmol, 0.1 equiv.), copper(I) iodide (CuI, 1.1 g, 5.6 mmol, 0.2 equiv.) in ***freshly distilled*** trimethylamine (Et_3N, 0.4 M) under a nitrogen atmosphere at 50 °C, trimethylsilylacetylene (4.8 mL, 33.5 mmol, 1.2 equiv.) was added dropwise. After 10 h, an extra 0.5 equiv. of trimethylsilylacetylene was added and the reaction mixture was stirred further for 10 h. Then, the mixture was cooled down to room temperature and filtered thorugh a Celite™ pad and washed with DCM (50 mL). The filtrate was washed with saturated NH_4Cl (*aq.*) (50 mL) and extracted with DCM (3 x 40 mL). The organic extract was dried over $MgSO_4$, filtered, and evaporated to dryness *in vacuo*. The resulting crude was triturated with petrol (100 mL) and filtered out. The filtrate was evaporated to dryness *in vacuo* and was purified by column chromatography on silica gel with a mixture of petrol:EtOAc 95:5 as eluent to afford the product as a yellow oil (9.28 g, 98%).

<u>*Note:*</u> Triethylamine was stirred at room temperature with KOH pellets under a nitrogen atmosphere for 2 h, followed by distillation over 4 Å MS under a nitrogen atmosphere before use.

¹H NMR (400 MHz, CDCl₃): δ 7.60 – 7.55 (m, 2H), 7.40 – 7.31 (m, 3H), 7.18 (d, *J* = 9.0 Hz, 1H), 6.62 (d, *J* = 9.0 Hz, 1H), 5.17 (s, 2H), 3.87 (s, 3H), 3.86 (s, 3H), 0.24 (s, 9H).

¹³C NMR (101 MHz, CDCl₃): δ 154.6, 154.1, 137.5, 128.6, 128.3, 127.9, 110.9, 107.4, 101.3, 97.0, 75.4, 61.1, 56.1, 0.1.

HRMS: (ESI) *m/z* $[M+H]^+$ calculated for $C_{20}H_{25}O_3Si$ 341.1573, found 341.1581.

AR–FTIR (neat, cm⁻¹): 2959, 2151, 1592, 1492, 1427, 1295.

Synthesis of 2-Benzyloxy-1-ethynyl-2,3-dimethoxybenzene

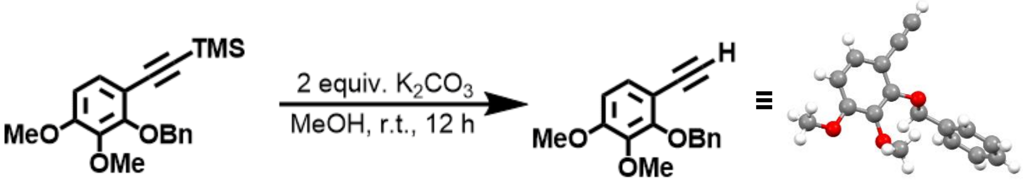

To a stirred solution of (2-benzyloxy-2,3-dimethoxyphenylethynyl)trimethylsilane (9.20 g, 27.1 mmol, 1 equiv.) in MeOH (100 mL), potassium carbonate (K_2CO_3, 7.49 g, 54.2 mmol, 2 equiv.) was added. The mixture was stirred for 12 h at room temperature. Then, volatiles were evaporated *in vacuo*, and the resulting crude was washed with water (20 mL), brine (20 mL), and extracted with DCM (3 x 30 mL). The organic extract was dried over $MgSO_4$, filtered, and evaporated to dryness *in vacuo* to afford the product as a brown solid (5.22 g, 72%).

M.P. 69–71 °C recrystallized from DCM.

¹H NMR (400 MHz, CDCl₃): δ 7.60 – 7.55 (m, 2H), 7.42 – 7.33 (m, 3H), 7.19 (d, *J* = 9.0 Hz, 1H), 6.64 (d, *J* = 9.0 Hz, 1H), 5.17 (s, 2H), 3.88 (s, 3H), 3.87 (s, 3H), 3.21 (s, 1H).

¹³C NMR (101 MHz, CDCl₃): δ 154.8, 154.3, 142.5, 137.4, 128.7, 128.5, 128.3, 128.0, 107.6, 80.0, 76.7, 71.2, 71.1, 61.1, 56.1.

HRMS: (ESI) *m/z* $[M+H]^+$ calculated for $C_{17}H_{17}O_3$ 269.1178, found 269.1174.

AR–FTIR (neat, cm⁻¹): 3283, 2939, 2104, 1037, 1594, 1491, 1453, 1294, 1098.

Synthesis of Potassium ((2-(benzyloxy)-3,4-dimethoxyphenyl)ethynyl)trifluoroborate

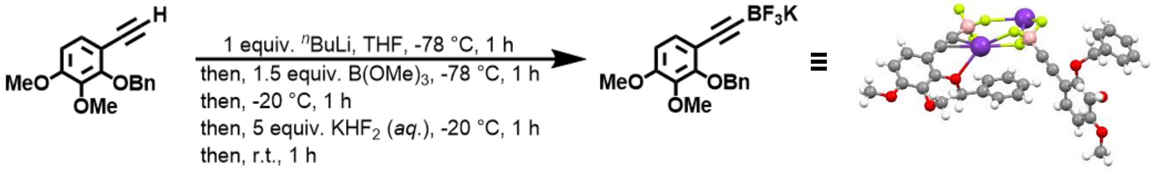

To a stirred solution of 2-benzyloxy-1-ethynyl-2,3-dimethoxybenzene (5.00 g, 18.7 mmol) in dry THF (0.15 M) under a nitrogen atmosphere at –78 °C, a solution of ^{n}BuLi (2.5 M in hexanes, 7.5 mL, 18.7 mmol, 1 equiv.) was added dropwise. After 1 h, trimethyl borate ($B(OMe)_3$, 3.1 mL, 28.1 mmol, 1.5 equiv.) was added dropwise and the reaction mixture was stirred at –78 °C for 1 h. Then, the reaction mixture was warmed up to –20 °C and stirred for 1 h. A solution of potassium hydrogen fluoride (KHF_2, 7.3 g, 93.5 mmol, 5 equiv.) in deionized water (3.5 M) was added and the resulting mixture was stirred for 1 h at –20 °C. Then, the reaction was warmed up to room temperature and stirred for 1 h. Volatiles were evaporated *in vacuo* and the residual solid was suspended in acetone (75 mL) and heated at reflux for 1 h. Then, the suspension was filtered while still warm and volatiles were evaporated *in vacuo*. The residual solid was washed with cold Et_2O (50 – 100 mL) and filtered to afford the product as a white solid (5.28 g, 85%).

M.P. 210–212 °C (decomposition) recrystallized from acetone.

^{1}H NMR (400 MHz, *d*^{6}-DMSO): δ 7.63 – 7.61 (m, 2H), 7.38 – 7.32 (m, 3H), 7.01 (d, *J* = 8.5 Hz, 1H), 6.73 (d, *J* = 8.5 Hz, 1H), 5.07 (s, 2H), 3.78 (s, 3H), 3.70 (s, 3H).

^{13}C NMR (101 MHz, *d*^{6}-DMSO): δ 153.4, 153.1, 142.4, 138.0, 129.1, 128.6, 128.3, 127.9, 113.3, 108.4, 74.8, 65.4, 61.0, 56.3.

^{11}B NMR (128 MHz, *d*^{6}-DMSO): δ –1.5 (s, br).

^{19}F NMR (377 MHz, *d*6-DMSO): δ –131.7 (s).

HRMS: (ESI) *m/z* $[M–K]^-$ calculated for $C_{17}H_{15}BF_3O_3$ 335.1072, found 335.1060.

AR–FTIR (neat, cm^{-1}): 2942, 2183, 1594, 1490, 1295, 1051.

Synthesis of 6-methoxyquinoline-2-carbohydrazonamide

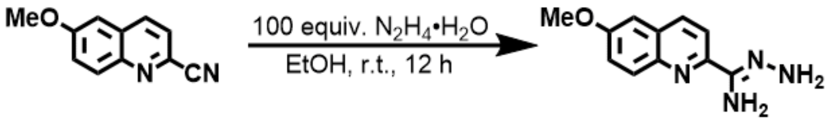

6-Methoxyquinoline-2-carbonitrile (1.00 g, 5.43 mmol, 1 equiv.) and hydrazine monohydrate (N_2H_4•H_2O, N_2H_4 64–65%, 26.4 mL, 543 mmol, 100 equiv.) in EtOH (20 mL) were stirred for 12 h at room temperature. The product was filtered and washed with deionized water (150 mL) to afford it as an amorphous yellow solid (1.12 g, 95%).

Note: Hydrazine monohydrate (N_2H_4•H_2O) is a highly toxic, carcinogenic, explosive and volatile liquid. It must be handled with care and always inside a well-ventilated fume hood.

M.P. 219 – 221 °C (decomposition) not recrystallized.
^{1}H NMR (400 MHz, *d*^{6}-DMSO): δ 8.14 (d, *J* = 8.5 Hz, 1H), 8.02 (d, *J* = 8.5 Hz, 1H), 7.90 (d, *J* = 9.0 Hz, 1H), 7.39 – 7.34 (m, 2H), 5.83 (s, 2H), 5.42 (s, 2H), 3.89 (s, 3H).
^{13}C NMR (101 MHz, *d*^{6}-DMSO): δ 157.2, 149.5, 143.2, 142.0, 134.6, 130.0, 128.6, 121.6, 117.8, 106.0, 55.5.
HRMS: (ESI) *m/z* $[M+H]^+$ calculated for $C_{11}H_{13}N_4O$ 217.1084, found 217.1087.
AR–FTIR (neat, cm^{-1}): 1023, 1164, 1230, 1390, 1501, 1635, 3220.

Synthesis of 3-(6-methoxyquinolin-2-yl)-6-methyl-1,2,4-triazin-5(2*H*)-one

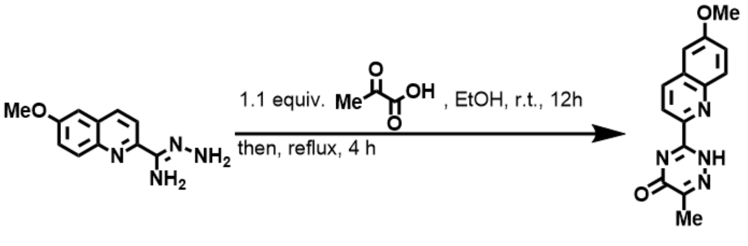

To a stirred suspension of 6-methoxyquinoline-2-carbohydrazonamide (2.00 g, 9.5 mmol, 1 equiv.) in EtOH (100 mL) was added pyruvic acid (0.72 mL, 10.2 mmol, 1.1 equiv.) and the mixture was stirred at room temperature for 12 h. Then, the mixture was heated to 98 °C and stirred further for 4 h. After cooling down to room temperature, the resulting precipitate was

filtered and washed with Et_2O (50 mL) to afford the product as an amorphous white solid (2.36 g, 95%).

^{1}H NMR (400 MHz, *d*^{6}-DMSO): δ 14.03 (s, br, 1H), 8.48 (d, *J* = 8.0 Hz, 1H), 8.28 (d, *J* = 8.0 Hz, 1H), 8.08 (d, *J* = 9.0 Hz, 1H), 7.54 (d, *J* = 9.0 Hz, 1H), 7.50 (s, 1H), 3.94 (s, 3H), 2.25 (s, 3H).

^{13}C NMR (101 MHz, *d*^{6}-DMSO): δ 159.4, 143.1, 137.1, 131.3, 131.1, 124.7, 119.7, 106.3, 56.3, 17.8. (*4 signals missing; weak spectrum due to low solubility*).

HRMS: (ESI) *m/z* $[M+H]^+$ calculated for $C_{14}H_{13}N_4O_2$ 269.1033, found 269.1039.

AR–FTIR (neat, cm^{-1}): 1024, 1232, 1383, 1477, 1535, 1652, 3293.

Synthesis of 2-(5-chloro-6-methyl-1,2,4-triazin-3-yl)-6-methoxyquinoline

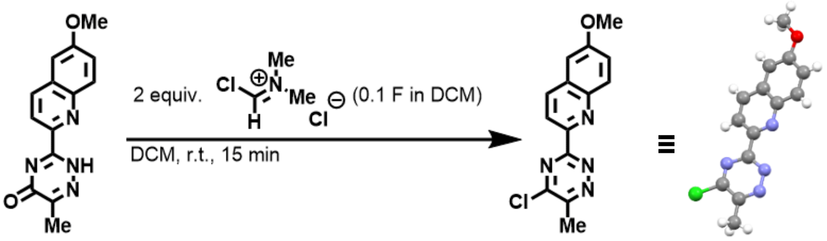

To a suspension of 3-(6-methoxyquinolin-2-yl)-6-methyl-1,2,4-triazin-5(2*H*)-one (2.00 g, 7.5 mmol, 1 equiv.) in dry DCM (200 mL) was slowly added a solution of Vilsmeier reagent ((chloromethylene)dimethyliminium chloride, 150 mL, 0.1 F in DCM, 2 equiv.) via a dropping funnel. After the addition, the mixture was stirred at room temperature for 15 min. Then, the reaction was quenched with saturated $NaHCO_3$ (*aq.*) (40 mL) and the mixture was washed with 1 M NaOH (*aq.*) (120 mL). The mixture was extracted with DCM (3 x 50 mL) and the organic extract was dried over $MgSO_4$, filtered, and evaporated to dryness *in vacuo*. The reaction crude was purified by column chromatography on silica gel with a mixture of DCM:EtOAc 7:3 as eluent to afford the product as a yellow solid (1.58 g, 74%).

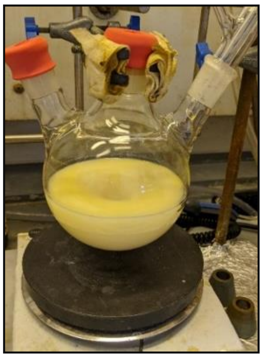

Initial suspension of triazinone in DCM.

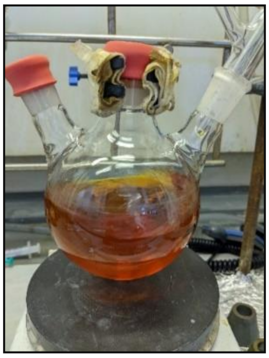

While adding the Vilsmeier regaent solution.

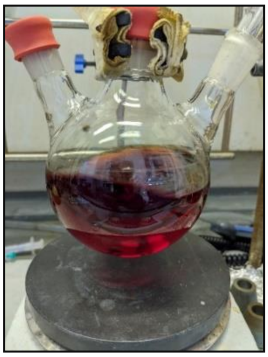

After addition of the Vilsmeier reagent solution (t = 0 min).

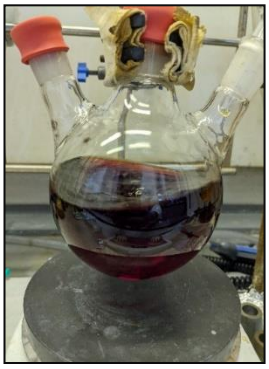

After stirring for 15 min.

<u>*Note:*</u> The (chloromethylene)dimethyliminium chloride solution was prepared by adding 2.3 mL of dry DMF dropwise to a solution of 2.5 mL of oxalyl chloride $(COCl)_2$ in 300 mL of dry DCM under a nitrogen atmosphere. The mixture was stirred overnight and left to stand at room temperature under a nitrogen atmosphere until use.

M.P. 152–154 °C (decomposition) recrystallized from DCM.

^{1}H NMR (400 MHz, $CDCl_3$): δ 8.61 (d, J = 8.5 Hz, 1H), 8.25 (d, J = 9.5 Hz, 1H), 8.23 (d, J = 9.0 Hz, 1H), 7.42 (dd, J = 9.0, 3.0 Hz, 1H), 7.12 (d, J = 3.0 Hz, 1H), 3.96 (s, 3H), 2.89 (s, 3H).

^{13}C NMR (101 MHz, $CDCl_3$): δ 161.9, 159.2, 158.5, 157.3, 148.9, 144.5, 135.9, 132.3, 130.3, 123.2, 121.1, 104.8, 55.7, 20.0.

HRMS: (ESI) *m/z* $[M+H]^+$ calculated for $C_{14}H_{12}ClN_4O$ 287.0694, found 287.0699.

AR–FTIR (neat, cm^{-1}): 1028, 1111, 1227, 1399, 1478, 1619, 3005.

Synthesis of 3-(6-methoxyquinolin-2-yl)-6-methyl-1,2,4-triazine-5-carbonitrile

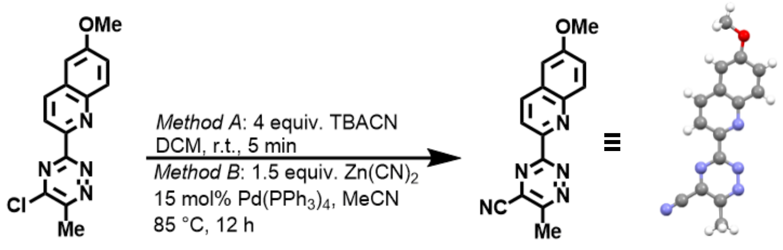

<u>*Method A:*</u> 2-(5-Chloro-6-methyl-1,2,4-triazin-3-yl)-6-methoxyquinoline (1.00 g, 3.50 mmol, 1 equiv.) was added to a flame-dried three necked 250 mL flask equipped via a dropping funnel under a nitrogen atmposphere. Then, the dropping funnel was filled with 100 mL of dry DCM. Tetrabutylammonium cyanide (TBACN, 3.85 g, 14 mmol, 4 equiv.) was added and the tap of the funnel was quickly opened. The mixture was stirred for 5 min at room temperature and then ***immediately*** washed with brine (80 mL). The organic extract was dried over $MgSO_4$, filtered, and evaporated to dryness *in vacuo*. The reaction crude was purfied by column chromatography on silica gel with a mixture of DCM:EtOAc 9:1 as eluent to afford the product as an orange solid (523 mg, 54%).

<u>*Method B:*</u> A mixture of 2-(5-chloro-6-methyl-1,2,4-triazin-3-yl)-6-methoxyquinoline (500 mg, 1.75 mmol, 1 equiv.), zinc cyanide ($Zn(CN)_2$, 308 mg, 2.62 mmol, 1.5 equiv.), and tetrakis(triphenylphosphine)palladium(0) ($Pd(PPh_3)_4$, 303 mg, 0.26 mmol, 15 mol%) in dry MeCN (24 mL) was heated to 85 °C and stirred for 12 h. Then, the mixture was cooled down to room temperature and filtered through a Celite^{TM} pad eluting with DCM (200 mL). Volatiles were evaporated *in vacuo* and the resulting crude was purfied by column chromatography on silica gel with a mixture of DCM:EtOAc 9:1 as eluent to afford the product as a yellow solid (266 mg, 55%).

M.P. 167–169 °C (decomposition) recrystallized from DCM.

^{1}H NMR (400 MHz, $CDCl_3$): δ 8.62 (d, J = 8.5 Hz, 1H), 8.27 (d, J = 3.0 Hz, 1H), 8.25 (d, J = 4.5 Hz, 1H), 7.45 (dd, J = 9.0, 3.0 Hz, 1H), 7.14 (d, J = 3.0 Hz, 1H), 3.97 (s, 3H), 3.05 (s, 3H).

^{13}C NMR (101 MHz, $CDCl_3$): δ 161.7, 159.5, 157.9, 148.3, 144.7, 136.2, 135.7, 132.3, 130.5, 123.6, 121.0, 113.6, 104.8, 55.7, 19.5.

HRMS: (ESI) m/z $[M+H]^+$ calculated for $C_{15}H_{12}N_5O$ 278.1036, found 278.1041.

AR–FTIR (neat, cm^{-1}): 1022, 1092, 1163, 1223, 1615, 2008, 2999.

Synthesis of 3-(6-methoxy-5-nitroquinolin-2-yl)-6-methyl-1,2,4-triazine-5-carbonitrile

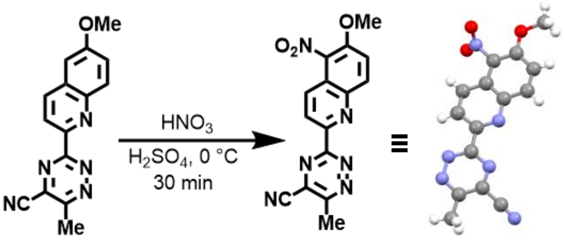

To a stirred suspension of 3-(6-methoxyquinolin-2-yl)-6-methyl-1,2,4-triazine-5-carbonitrile (500 mg, 2.19 mmol) in sulfuric acid (H_2SO_4, 5 mL) at 0 °C was added nitric acid ($HNO_3 \geq$ 65%, 0.9 mL). The mixture was ***vigorously*** stirred for 30 min at 0 °C. Then, the mixture was poured into crushed ice and carefully neutralized with saturated $NaHCO_3$ (*aq*.). The mixture was washed with water (20 mL) and extracted with DCM (3 x 25 mL). The organic extract was dried over $MgSO_4$, filtered, and evaporated to dryness *in vacuo* to afford the product as a yellow solid (570 mg, 81%).

M.P. 170–172 °C (decomposition) recrystallized from DCM.

^{1}H NMR (400 MHz, CDCl$_3$): δ 8.79 (d, *J* = 9.0 Hz, 1H), 8.55 (dd, *J* = 9.5, 1.0 Hz, 1H), 8.33 (dd, *J* = 9.0, 1.0 Hz, 1H), 7.70 (d, *J* = 9.5 Hz, 1H), 4,14 (s, 3H), 3.09 (s, 3H).

^{13}C NMR (101 MHz, CDCl$_3$): δ 160.9, 158.6, 150.8, 150.1, 142.4, 135.8, 135.5, 131.0, 123.1, 122.3, 117.3, 113.4, 57.3, 19.6. (*1C missing*).

HRMS: (ESI) *m/z* $[M+H]^+$ calculated for $C_{15}H_{11}N_6O_3$ 323.0887, found 323.0983.

AR–FTIR (neat, cm^{-1}): 1117, 1270, 1524, 1627, 2159, 2996.

Synthesis of 10-(2-(benzyloxy)-3,4-dimethoxyphenyl)-8-cyano-11,11-difluoro-3-methoxy-9-methyl-4-nitro-11*H*-pyrido[3',2':3,4][1,2]azaborolo[1,5-*a*]quinolin-12-ium-11-uide

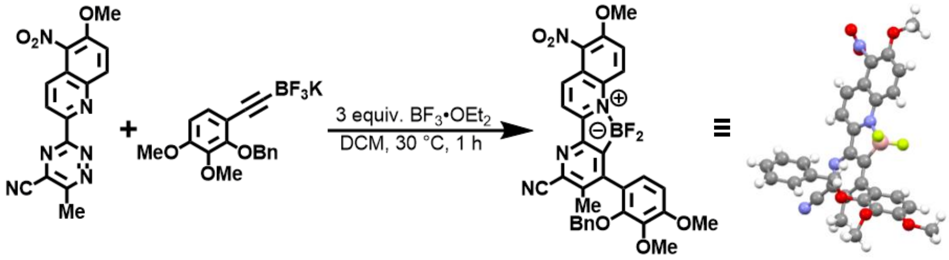

To a stirred suspension of 3-(6-methoxy-5-nitroquinolin-2-yl)-6-methyl-1,2,4-triazine-5-carbonitrile (1.10 g, 3.42 mmol, 1 equiv.) and potassium ((2-(benzyloxy)-3,4-

dimethoxyphenyl)ethynyl)trifluoroborate salt (1.14 g, 3.42 mmol, 1 equiv.) in dry DCM (34.2 mL, 0.1 mol/L) at ***30 °C*** under a nitrogen atmosphere was added ***freshly distilled*** boron trifluoride diethyl etherate (BF_3•OEt_2, 1.3 mL, 10.3 mmol, 3 equiv.) dropwise and the mixture was stirred at ***30 °C*** for 1 h. Then, saturated $NaHCO_3$ (*aq.*) (15 mL) was added, and the reaction mixture was cooled down to room temperature. The reaction mixture was washed with deionized water (20 mL) and extracted with DCM (3 x 20 mL). The organic extract was dried over $MgSO_4$, filtered, and evaporated to dryness *in vacuo*. The reaction crude was purfied by column chromatography on silica gel with a mixture of DCM:Et_2O 99:1 as eluent to afford the product as a pale yellow solid (1.35 g, 65%).

M.P. 258–260 °C recrystallized from DCM.

^{1}H NMR (400 MHz, $CDCl_3$): δ 8.71 (d, *J* = 9.5 Hz, 1H), 8.63 – 8.52 (m, 2H), 7.78 (d, *J* = 9.5 Hz, 1H), 7.16 – 7.09 (m, 4H), 6.97 – 6.84 (m, 3H), 5.02 (d, *J* = 11.0 Hz, 1H), 4.51 (d, *J* = 11.0 Hz, 1H), 4.14 (s, 3H), 3.97 (s, 3H), 3.94 (s, 3H), 2.44 (s, 3H).

^{13}C NMR (101 MHz, $CDCl_3$): δ 154.5, 154.0, 153.5, 151.6, 150.6, 150.1, 142.6, 141.9, 137.9, 137.3, 135.0, 134.4, 128.1, 127.7, 127.5, 127.4, 124.4, 124.3, 124.2, 123.0, 120.3, 118.5, 116.8, 107.9, 75.8, 61.3, 57.6, 56.0, 17.4.

^{11}B NMR (128 MHz, $CDCl_3$): δ 8.79 (s, br).

^{19}F NMR (377 MHz, $CDCl_3$): δ –147.2 (d, *J* = 140 Hz), –154.7 (d, *J* = 140 Hz).

HRMS: (ESI) *m/z* $[M+H]^+$ calculated for $C_{32}H_{26}BF_2N_4O_6$ 611.1908, found 611.1930.

AR–FTIR (neat, cm^{-1}): 1093, 1126, 1273, 1344, 1529, 1598.

Synthesis of (4*S*,11b*R*)-10'-(2-(benzyloxy)-3,4-dimethoxyphenyl)-8'-cyano-3'-methoxy-9'-methyl-4'-nitrospiro[dinaphtho[2,1-*d*:1',2'-*f*][1,3,2]dioxaborepine-4,11'-pyrido[3',2':3,4][1,2]azaborolo[1,5-*a*]quinolin]-12'-ium-26-uide

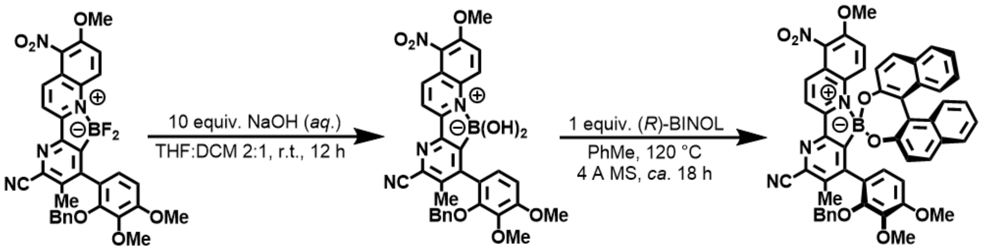

To a stirred solution of 10-(2-(benzyloxy)-3,4-dimethoxyphenyl)-8-cyano-11,11-difluoro-3-methoxy-9-methyl-4-nitro-11*H*-pyrido[3',2':3,4][1,2]azaborolo[1,5-*a*]quinolin-12-ium-11-uide (1.10 g, 1.8 mmol, 1 equiv.) in a mixture of THF:DCM 2:1 (18 mL in total, 0.1 M) under a nitrogen atmosphere was added 1 M NaOH (*aq.*) (10.8 mL, 10.8 mmol, 10 equiv.). The

mixture stirred for 12 h at room temperature under a nitrogen atmosphere. Then, the mixture was concentrated *in vacuo*, extracted with DCM (5 x 25 mL), washed with water (30 mL) and 1 M HCl (*aq.*) (25 mL in total, adding 5 mL per DCM extraction). The organic extract was dried over $MgSO_4$, filtered, and evaporated to dryness *in vacuo* to afford the corresponding aryl boronic acid as an amorphous beige solid which was used without further purification.

Note 1: Protodeboration and oxidation side reactions occur if the reaction is carried out the in the absence of an inert atmosphere.

Characterization of the picolinitrile boronic acid intermediate:
^{1}H NMR (400 MHz, $CDCl_3$): δ 8.96 (d, *J* = 9.5 Hz, 1H), 8.57 (d, *J* = 9.0 Hz, 1 H), 8.47 (dd, *J* = 9.0, 0.5 Hz, 1H), 7.69 (d, *J* = 9.5 Hz, 1H), 7.16 – 7.11 (m, 3H), 7.10 – 6.83 (m, 4H), 5.03 (d, *J* = 12.0 Hz, 1H), 4.91 (d, *J* = 12.0 Hz, 1H), 4.11 (s, 3H), 4.01 (s, 3H), 3.96 (s, 3H), 2.08 (s, 3H). (*Wet acetone was added to the sample to obtain a cleaner spectrum. Presumably, water prevents the formation of boroxines which yield complex NMR spectra*).
^{13}C NMR (101 MHz, $CDCl_3$): δ 154.2, 153.4, 152.3, 150.2, 150.0, 149.7, 148.6, 142.8, 140.0, 136.6, 135.3, 135.0, 133.9, 129.0, 128.33 (x 2C), 128.27 (x 2C), 128.2, 124.5, 124.0, 122.9, 119.0, 118.7, 117.0, 108.0, 75.5, 61.1, 57.5, 56.1, 17.2.
^{11}B NMR (128 MHz, $CDCl_3$): δ 12.9 (s, br).
HRMS: (ESI) *m/z* $[M+H]^+$ calculated for $C_{32}H_{28}BN_4O_8$ 607.1995, found 607.2014.
AR–FTIR (neat, cm^{-1}): 1003, 1093, 1274, 1354, 1496, 1530, 1597, 1976, 2159.

The aryl boronic acid (500 mg, 0.83 mmol, 1 equiv.) obtained in the previous step was added to a flame-dried two-neck flask (connected to a condenser) charged with activated 4 Å MS under a nitrogen atmosphere and dissolved in PhMe (15 mL), then (*R*)-(+)-1,1’-bi(2-naphtol) ((*R*)-BINOL, 237 mg, 0.83 mmol, 1 equiv.). The mixture was then heated to 120 °C and left to stir overnight (*ca.* 18 h). The reaction mixture was cooled down to room temperature, filtered and volatiles were evaporated to dryness *in vacuo*. ^{1}H NMR spectrum of the crude sample showed that the corresponding boronic ester was obtained as a 4.6:1 mixture of diastereomers. The reaction crude was purified by column chromatography on silica gel with a mixture of DCM:Et_2O 99.5:0.5 as eluent to obtain the product as a 8:1 mixture of diastereomers (amorphous orange solid, 396 mg, 56%).

Note 2: A second fraction of product was also isolated (240 mg, 34%, 2:1 *d.r.*) which could be equilibrated back to the thermodynamic ratio of 4.6:1 by subjecting it to the conditions just described, hence, allowing the recycle of material.

$[\alpha]_D^{21.5}$ = –310 [1.0 *c* in $CHCl_3$].

^{1}H NMR (400 MHz, $CDCl_3$, major diastereomer): δ 8.73 (d, *J* = 9.0 Hz, 1H), 8.54 (dd, *J* = 9.0, 1.0 Hz, 1H), 8.04 –7.88 (m, 3H), 7.80 (d, *J* = 8.0 Hz, 1H), 7.53 (d, *J* = 8.5 Hz, 1H), 7.46 – 7.22 (m, 9H), 7.17 – 7.12 (m, 3H), 7.04 (d, *J* = 8.7 Hz, 1H), 6.71 (d, *J* = 8.7 Hz, 1H), 6.47 (d, *J* = 8.7 Hz, 1H), 5.94 (d, *J* = 10.0 Hz, 1H), 5.31 (d, *J* = 11.0 Hz, 1H), 4.82 (d, *J* = 11.0 Hz, 1H), 3.97 (s, 3H), 3.68 (s, 3H), 3.25 (s, 3H), 2.42 (s, 3H). (*The compound partially hydrolyses to its corresponding boronic acid in $CDCl_3$ due to traces of HCl).*

^{1}H NMR (400 MHz, C_6D_6, major diastereomer): δ 8.00 (d, *J* = 10.0 Hz, 1H), 7.95 (d, *J* = 9.0 Hz, 1H), 7.75 (d, *J* = 8.0 Hz, 1H), 7.67 – 7.53 (m, 5H), 7.50 (d, *J* = 8.0 Hz, 1H), 7.24 (d, *J* = 8.5 Hz, 1H), 7.20 – 6.92 (m, 10H), 6.55 (d, *J* = 8.5 Hz, 1H), 5.47 – 5.38 (m, 2H), 5.16 (d, *J* = 8.5 Hz, 1H), 5.08 (d, *J* = 11.0 Hz, 1H), 3.83 (s, 3H), 2.90 (s, 3H), 2.66 (s, 3H), 2.34 (s, 3H).

^{13}C NMR (101 MHz, C_6D_6, major diastereomer): δ 155.2, 155.0, 154.5, 154.2, 153.8, 150.9, 149.7, 148.7, 142.0, 141.8, 137.8, 136.1, 135.1, 134.7, 134.3, 134.0, 133.6, 131.1, 130.7, 130.0, 126.6, 129.3, 128.4, 127.7, 127.5, 127.22, 127.15, 127.1, 126.8, 125.8, 125.7, 125.2, 124.5, 123.8, 123.63, 123.56, 123.2, 123.0, 122.3, 120.3, 118.3, 118.0, 116.4, 106.4, 75.6, 60.4, 55.7, 54.5, 17.5.

^{11}B NMR (128 MHz, C_6D_6): δ 14.8 (s, br).

HRMS: (ESI) *m/z* $[M+H]^+$ calculated for $C_{52}H_{38}BN_4O_8$ 857.2777, found 857.2783.

AR–FTIR (neat, cm^{-}1): 1094, 1251, 1279, 1534, 1597, 2387, 2939.

Synthesis of 5-amino-4-(2-(benzyloxy)-3,4-dimethoxyphenyl)-6-(6-methoxy-5-nitroquinolin-2-yl)-3-methylpicolinonitrile

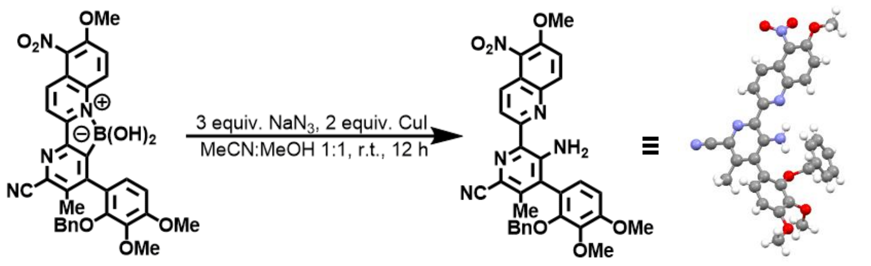

Racemic synthesis:

A suspension of 10-(2-(benzyloxy)-3,4-dimethoxyphenyl)-8-cyano-11,11-dihydroxy-3-methoxy-9-methyl-4-nitro-11*H*-pyrido[3',2':3,4][1,2]azaborolo[1,5-a]quinolin-12-ium-11-uide (500 mg, 0.83 mmol, 1 equiv.), sodium azide (NaN_3, 161 mg, 2.48 mmol, 3 equiv.), copper(I) iodide (CuI, 316 mg, 1.66 mmol, 2 equiv.) in a mixture of MeCN:MeOH 1:1 (40 mL in total) was stirred at room temperature for 12 h. Then, the reaction mixture was filtered through a Celite™ pad eluting with EtOAc (25 mL). The filtrate was evaporated to dryness *in vacuo* and the resulting crude was purified by column chromatography on silica gel with a mixture of DCM:EtOAc 99:1 as eluent to afford the product as a yellow solid (352 mg, 74%).

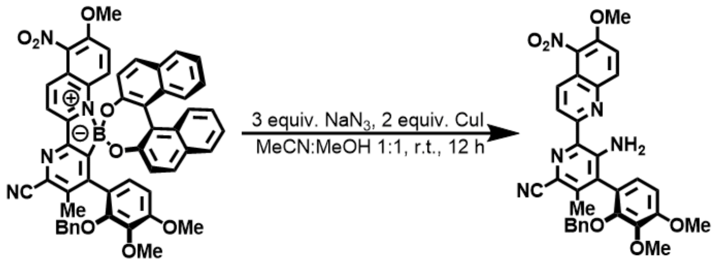

Enantioenriched synthesis:

A suspension of (4*S*,11b*R*)-10'-(2-(benzyloxy)-3,4-dimethoxyphenyl)-8'-cyano-3'-methoxy-9'-methyl-4'-nitrospiro[dinaphtho[2,1-*d*:1',2'-*f*][1,3,2]dioxaborepine-4,11'-pyrido[3',2':3,4][1,2]-azaborolo[1,5-*a*]quinolin]-12'-ium-26-uide (300 mg, 0.35 mmol, 1 equiv.), sodium azide (NaN_3, 68.3 mg, 1.05 mmol, 3 equiv.), copper(I) iodide (CuI, 133.3 mg, 0.7 mmol, 2 equiv.) in a mixture of MeCN:MeOH 1:1 (24 mL in total) was stirred at room temperature for 12 h. Then, the reaction mixture was filtered through Celite™ eluting with EtOAc (25 mL). The

filtrate was evaporated to dryness *in vacuo* at ***35 °C*** and the resulting crude was purified by column chromatography on silica gel with a mixture of DCM:EtOAc 99:1 as eluent to afford the product as a yellow solid (101 mg, 50%, 92:8 *e.r.*, 84% *ee*).

$[\alpha]_D^{18.5}$ = +62.5 [1.0 *c* in $CHCl_3$].

M.P. 230–232 °C recrystallized from DCM.

^{1}H NMR (400 MHz, $CDCl_3$): δ 8.89 (d, *J* = 9.0 Hz, 1H), 8.13 (d, *J* = 9.0 Hz, 1H), 8.06 (d, *J* = 9.5 Hz, 1H), 7.51 (d, *J* = 9.5 Hz, 1 H), 7.13 – 7.04 (m, 3H), 6.97 (m, 2H), 6.88 (d, *J* = 8.5 Hz, 1H), 6.84 (d, *J* = 8.5 Hz, 1H), 4.99 (d, *J* = 11.5 Hz, 1H), 4.92 (d, *J* = 11.5 Hz, 1H), 4.06 (s, 3H), 3.97 (*overlapping singlets*, 6H), 2.10 (s, 3H).

^{13}C NMR (101 MHz, $CDCl_3$): δ 157.3, 154.7, 150.3, 149.3, 145.9, 143.5, 139.9, 138.9, 136.9, 135.0, 134.0, 132.9, 132.0, 129.3, 128.3 (x 2C), 128.1, 128.0 (x 2C), 124.7, 123.1, 120.7, 120.3, 120.2, 118.1, 116.1, 108.7, 75.4, 61.1, 57.2, 56.2, 17.2.

HRMS: (ESI) *m/z* $[M+H]^+$ calculated for $C_{32}H_{28}N_5O_6$ 578.2034, found 578.2060.

AR–FTIR (neat, cm^{-1}): 3450, 2527, 2218, 2014, 1583, 1524, 1450, 1357, 1266, 1076.

Chiral HPLC: The *ee* was determined by HPLC using a Chiral Art amylose-SA S-5 µm column [*n*-hexane/*i*PrOH (50:50), flow rate = 1.0 mL/min]; t_{major} = 10.1 min, t_{minor} = 11.7 min (91.8:8.2).

Synthesis of methyl 5-amino-4-(2-(benzyloxy)-3,4-dimethoxyphenyl)-6-(6-methoxy-5,8-dioxo-5,8-dihydroquinolin-2-yl)-3-methylpicolinate

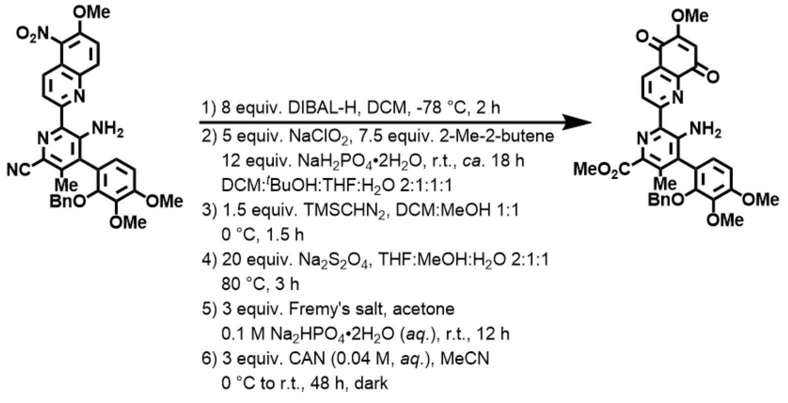

<u>Racemic synthesis:</u>

To a stirred solution of 5-amino-4-(2-(benzyloxy)-3,4-dimethoxyphenyl)-6-(6-methoxy-5-nitroquinolin-2-yl)-3-methylpicolinonitrile (200 mg, 0.35 mmol, 1 equiv.) in DCM (50 mL) at –78 °C was added a commercial solution of diisobutylalumnium hydride (1 M DIBAL-H in DCM, 2.1 mL, 2.1 mmol, 8 equiv.). The mixture was stirred for 2 h at –78 °C and then 2 mL of EtOAc was added, followed by Rochelle’s salt (*aq.*) (20 mL), and the mixture was stirred

further for 1 h at room temperature. Then, the mixture was washed with deionized water (20 mL) and the aqueous phase was extracted with DCM (2 x 20 mL). The organic extract was dried over $MgSO_4$, filtered, and evaporated to dryness *in vacuo*. The crude was used without further purification in the next step.

Characterization of the picolinaldehyde intermediate:

For characterization purposes, the aldehyde was purified by column chromatography on Florisil® with an elution gradient of DCM → DCM:EtOAc 98:2 to afford the product as a yellow solid (37.2 mg, 74%). The product was found to degrade on both silica gel and Florisil®.

M.P. 196 – 198 °C recrystallized from EtOAc.

^{1}H NMR (400 MHz, $CDCl_3$): δ 10.15 (s, 1H), 9.09 (d, *J* = 9.0 Hz, 1H), 8.18 (d, *J* = 9.0 Hz, 1H), 8.09 (d, *J* = 9.5 Hz, 1H), 7.53 (d, *J* = 9.5 Hz, 1H), 7.10 – 7.01 (m, 3H), 6.97 (m, 2H), 6.88 (d, *J* = 8.5 Hz, 1H), 6.84 (d, *J* = 8.5 Hz, 1H), 4.93 (s, 2H), 4.08 (s, 3H), 3.97 (s, 3H), 3.96 (s, 3H), 2.33 (s, 3H).

^{13}C NMR (101 MHz, $CDCl_3$): δ 194.3, 158.0, 154.4, 150.6, 149.2, 146.2, 143.5, 140.2, 139.8, 137.7, 137.1, 135.1, 132.9, 132.5, 129.3, 128.2, 127.8 (x 2C), 125.0, 123.1, 120.9, 120.2, 119.8, 116.1, 108.6, 75.3, 61.2, 57.2, 56.2, 16.1.

HRMS: (ESI) *m/z* $[M+H]^+$ calculated for $C_{32}H_{29}N_4O_7$ 581.2031, found 581.2059.

The aldehyde obtained in the previous step was dissolved in DCM (8 mL). Then, *tert*-butanol (^{t}BuOH, 4 mL) was added, followed by a solution of 2-methyl-2-butene (0.28 mL, 2.6 mmol, 7.5 equiv.) in THF (4 mL) and finally a solution of sodium chlorite ($NaClO_2$ technical ≥80%, 196 mg, 1.73 mmol, 5 equiv.) and sodium dihydrogen phosphate dihydrate (NaH_2PO_4•$2H_2O$, 649 mg, 4.16 mmol, 12 equiv.) in H_2O (4 mL). The mixture was stirred ***vigorously*** overnight (*ca*. 18 h) at room temperature. Then, the mixture was diluted with DCM (15 mL), and washed with water (15 mL). The aqueous phase was extracted two more times with DCM (15 mL). The organic extract was dried over $MgSO_4$, filtered, and evaporated to dryness *in vacuo* to afford the corresponding carboxylic acid, which was used without further purification.

The carboxylic acid obtained in the previous reaction was dissolved in a mixture of DCM:MeOH 1:1 (16 mL in total) and cooled down to 0 °C. Trimethylsilyldiazomethane (2 M $TMSCHN_2$ in hexanes, 0.26 mL, 0.52 mmol, 1.5 equiv.) was added and the mixture was stirred for 1.5 h at 0 °C. Then, AcOH (2 mL) was added, and the mixture was stirred for 10 min at room temperature. The mixture was washed with brine (25 mL) and extracted with DCM (3 x

15 mL). The organic phase was dried over $MgSO_4$, filtered, and evaporated to dryness *in vacuo* to afford the corresponding methyl ester, which was used without further purification.

Characterization of the methyl picolinate intermediate:

For characterization purposes, the methyl ester was purified by column chromatography on silica gel with an elution gradient of DCM → DCM:EtOAc 95:5 to afford the product as a yellow solid (24.8 mg, 47%). The product was found to degrade on silica gel.

^{1}H NMR (400 MHz, $CDCl_3$): δ 9.01 (d, *J* = 9.0 Hz, 1H), 8.16 (d, *J* = 9.0 Hz, 1H), 8.08 (d, *J* = 9.5 Hz, 1H), 7.51 (d, *J* = 9.5 Hz, 1H), 7.11 – 7.05 (m, 3H), 7.03 – 6.97 (m, 2H), 6.87 (d, *J* = 8.5 Hz, 1H), 6.84 (d, *J* = 8.5 Hz, 1H), 4.93 (s, 2H), 4.06 (s, 3H), 3.99 (s, 3H), 3.96 (s, 3H), 3.95 (s, 3H), 2.25 (s, 3H).

^{13}C NMR (101 MHz, $CDCl_3$): δ 167.0, 158.2, 154.3, 150.5, 149.2, 145.3, 143.5, 140.3, 137.1, 137.1, 135.4, 135.1, 133.5, 132.9, 131.9, 129.2, 128.2, 128.0, 127.9, 125.0, 123.6, 121.6, 120.2, 116.0, 108.6, 75.2, 61.2, 57.2, 56.2, 52.2, 17.5.

These data were in accordance with the literature[1].

The crude methyl ester obtained in the previous step was dissolved in a mixture of THF:MeOH:H_2O 2:1:1 (16 mL in total) and sodium dithionite ($Na_2S_2O_4$ technical ≥85%, 1.42 g, 6.93 mmol, 20 equiv.) was added. The suspension was stirred for 3 h at 80 °C. The mixture was then cooled down to room temperature, diluted with EtOAc (20 mL) and washed with saturated $NaHCO_3$ (*aq.*) (30 mL). The aqueous phase was extracted two more times with EtOAc (2 x 20 mL). The organic extract was dried over $MgSO_4$, filtered, and evaporated to dryness *in vacuo* to afford the corresponding diamine, which was used without further purification.

The crude diamine obtained in the previous step was dissolved in a mixture of acetone:H_2O 1:1 (8 mL in total), then di-sodium hydrogen phosphate dihydrate (Na_2HPO_4•$2H_2O$, 185 mg, 1.04 mmol, 3 equiv.) and Frémy´s salt ($K_2NO(SO_3)_2$, 279 mg, 1.04 mmol, 3 equiv.) were added. The mixture was stirred at room temperature for 12 h. The mixture was then diluted with DCM (15 mL) and washed with water (15 mL) and brine (15 mL). The aqueous phase was extracted again with DCM (2 x 15 mL). The organic phase was dried over $MgSO_4$, filtered, and evaporated to dryness *in vacuo* to afford the corresponding dihydroxyquinoline, which was used without further purification.

The crude dihydroxyquinoline obtained in the previous step was dissolved in MeCN (80 mL), cooled down to 0 °C, and protected from light with aluminium foil. Then, aqueous cerium

ammonium nitrate (0.04 M CAN, 26 mL, 1.04 mmol, 3 equiv.) was added dropwise, and the mixture was stirred at room temperature for 48 h in the dark. The mixture was then diluted with EtOAc (20 mL), washed with water (25 mL), and brine (25 mL). The organic extract was dried over $MgSO_4$, filtered, and evaporated to dryness *in vacuo*. The reaction crude was purified by column chromatography on silica gel with a mixture of petrol:EtOAc 1:1 as eluent to afford the product as a red solid (97 mg, 47% over 6 steps).

Note 5: The aqueous CAN solution was prepared 24 h in advance, and left to stand at room temperature until use.

Enantioenriched synthesis:

Enantioenriched 5-amino-4-(2-(benzyloxy)-3,4-dimethoxyphenyl)-6-(6-methoxy-5-nitroquinolin-2-yl)-3-methylpicolinonitrile (100 mg, 0.17 mmol, 1 equiv.) was used as the starting material and subjected to the six steps previously described. The final product was obtained as a red amorphous solid (28 mg, 47% over 6 steps, 90:10 *e.r.*, 80% *ee*).

$[\alpha]_D^{20}$ = +58 [1.0 *c* in $CHCl_3$].

^{1}H NMR (400 MHz, $CDCl_3$): δ 9.01 (d, *J* = 8.5 Hz, 1H), 8.48 (d, *J* = 8.5 Hz, 1H), 7.09 (m, 3H), 7.00 (m, 2H), 6.86 (d, *J* = 8.5 Hz, 1H), 6.83 (d, *J* = 8.5 Hz, 1H), 6.27 (s, 1H), 4.94 (d, *J* = 11.0 Hz, 1H), 4.88 (d, *J* = 11.0 Hz, 1H), 3.98 (s, 3H), 3.95 (s, 3H), 3.93 (*overlapping singlets*, 6H), 2.23 (s, 3H).

^{13}C NMR (101 MHz, $CDCl_3$): δ 182.8, 179.4, 166.9, 163.0, 160.5, 154.4, 150.5, 146.2, 145.5, 143.5, 137.9, 137.1, 135.6, 134.3, 133.9, 130.5, 128.2, 127.9, 127.8, 125.5, 125.4, 124.9, 121.4, 109.9, 108.6, 75.2, 61.1, 56.7, 56.2, 52.2, 17.6.

Chiral HPLC: The *ee* was determined by HPLC using a Chiral Art amylose-SA S-5 µm column [*n*-hexane/*i*PrOH (70:30), flow rate = 1.25 mL/min]; t_{major} = 12.2 min, t_{minor} = 40.7 min (89.7:10.2).

These data were in accordance with the literature.[2]

Synthesis of methyl 5-amino-6-(7-amino-6-methoxy-5,8-dioxo-5,8-dihydroquinolin-2-yl)-4-(2-hydroxy-3,4-dimethoxyphenyl)-3-methylpicolinate

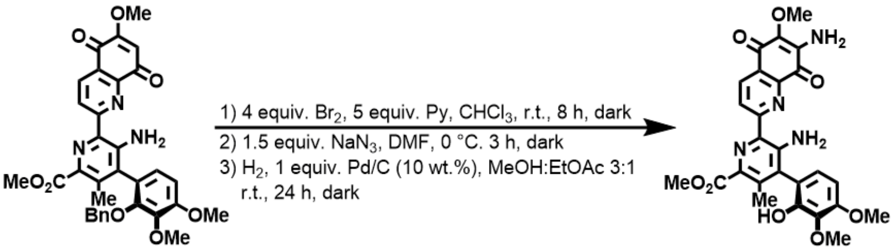

Methyl 5-amino-4-(2-(benzyloxy)-3,4-dimethoxyphenyl)-6-(6-methoxy-5,8-dioxo-5,8-dihydroquinolin-2-yl)-3-methylpicolinate (50 mg, 0.084 mmol, 1 equiv.) was dissolved in a solution of bromine (Br_2) and pyridine (Py) in dry $CHCl_3$ (4 mL, 0.1 M Br_2, 0.12 M Py). The mixture was stirred for 8 h at room temperature in the ***dark***. Then, the mixture was diluted with $CHCl_3$ (10 mL), washed with water (10 mL), 1 M HCl (*aq.*) (10 mL) and saturated $NaHCO_3$ (*aq.*) (10 mL). The organic extract was dried over Na_2SO_4, filtered, and evaporated to dryness *in vacuo* ***at room temperature in the dark***. The reaction crude was used directly in the following step without further purification.

The crude from the previous step was dissolved in dry DMF (4 mL), cooled down to 0 °C and protected from light. Sodium azide (NaN_3, 8.2 mg, 0.13 mmol, 1.5 equiv.) was added and the mixture was stirred for 3 h at 0 °C in the ***dark***. Then, the mixture was diluted with EtOAc (15 mL), and washed with deionized water (5 x 15 mL). The organic layer was dried over Na_2SO_4, filtered, and evaporated to dryness *in vacuo* ***at room temperature in the dark***. The reaction crude was used directly in the following step without further purification.

The crude from the previous step was dissolved in a degassed mixture of MeOH:EtOAc 3:1 (20 mL). Then palladium on activated charcoal (10 wt% Pd/C, 135 mg, 0.084 mmol, 1 equiv.) was added and the system was connected to a balloon of hydrogen and protected from light. The mixture was stirred for 24 h at room temperature in the ***dark***. The mixture was then filtered through Celite® and washed with EtOAc (20 mL). The filtrate was then evaporated to dryness *in vacuo* and the resulting crude was purified by column chromatography on silica gel with a mixture of DCM:MeOH 98:2 as eluent to afford the product as a brown solid (24 mg, 55%).

<u>*Note:*</u> The bromide and azide intermediates are sensitive to light, heat, and silica gel. It is crucial to do the reaction set up and work up in the dark and at room temperature to prevent decomposition/degradation and achieve reproducible results. Nevertheless, these intermediates can be stored for the next day if necessary by protecting them from light and keeping them in a freezer.

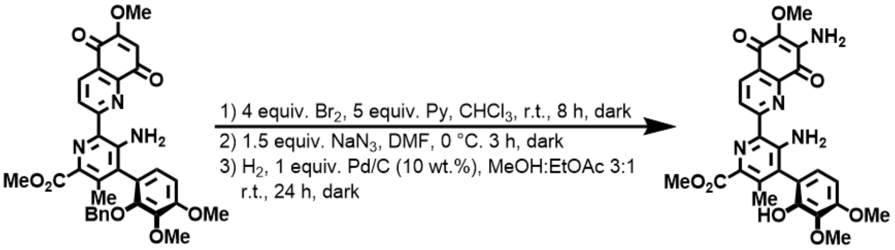

<u>Enantioenriched synthesis:</u>

Enantioenriched methyl 5-amino-4-(2-(benzyloxy)-3,4-dimethoxyphenyl)-6-(6-methoxy-5,8-dioxo-5,8-dihydroquinolin-2-yl)-3-methylpicolinate (25 mg, 0.042 mmol, 1 equiv.) was used as the starting material and subjected to the three steps just described. The final product was obtained as a brown solid (12 mg, 55% over 3 steps, 89.5:10.5 *e.r.*, 79% *ee*).

$[\alpha]_D^{19}$ = +35 [5 x 10^{-3} *c* in DCM].

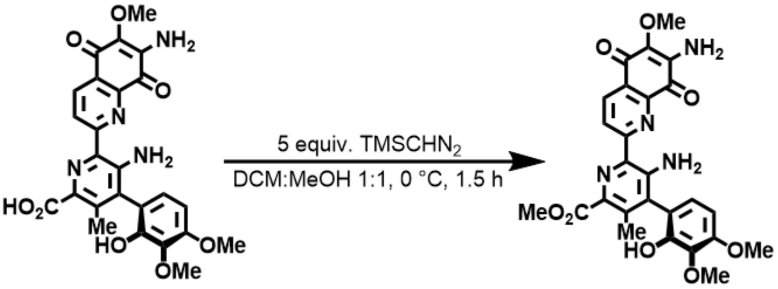

<u>Methylation of natural streptonigrin:</u>

Commercial streptonigrin (5 mg, 0.01 mmol, 1 equiv.) was dissolved in a mixture of DCM:MeOH 1:1 (4 mL in total) and cooled down to 0 °C. Trimethylsilyldiazomethane (2 M $TMSCHN_2$ in hexanes, 0.03 mL, 0.05 mmol, 5 equiv.) was added and the mixture was stirred for 1.5 h at 0 °C. Then, AcOH (1 mL) was added, and the mixture was stirred for 10 min at room temperature. The mixture was washed with brine (10 mL) and extracted with DCM (3 x 5 mL). The organic phase was dried over $MgSO_4$, filtered, and evaporated to dryness *in vacuo*.

The crude was purified by column chromatography on silica gel to afford the product as a brown solid (4 mg, 78% yield, 92:8 *e.r.*, 84% *ee*).

^{1}H NMR (400 MHz, CDCl$_3$): δ 8.98 (d, *J* = 8.5 Hz, 1H), 8.41 (d, *J* = 8.5 Hz, 1H), 6.81 (d, *J* = 8.5 Hz, 1H), 6.66 (d, *J* = 8.5 Hz, 1H), 5.87 (s, 1H), 5.07 (s, 2H), 4.08 (s, 3H), 3.98 (*overlapping singlets*, 6H), 3.95 (s, 3H), 2.32 (s, 3H).
^{13}C NMR (101 MHz, CDCl$_3$): δ 180.1, 177.6, 167.2, 161.5, 152.8, 147.2, 145.8, 144.1, 139.1, 137.8, 137.4, 136.5, 135.6, 133.9, 132.8, 131.2, 126.8, 126.5, 125.2, 114.0, 105.2, 61.3, 60.7, 56.1, 52.3, 17.5.
HRMS: (ESI) *m/z* $[M+H]^+$ calculated for $C_{26}H_{25}N_4O_8$ 521.1667, found 521.1675.
Chiral HPLC: The *ee* was determined by HPLC using a Chiral Art amylose-SC S-5 µm column [*n*-hexane/*i*PrOH (60:40), flow rate = 0.7 mL/min, 40 °C]; t_{major} = 27.8 min, t_{minor} = 25 min (89.2:10.8).
These data were in accordance with the literature.[2]

Synthesis of Streptonigrin

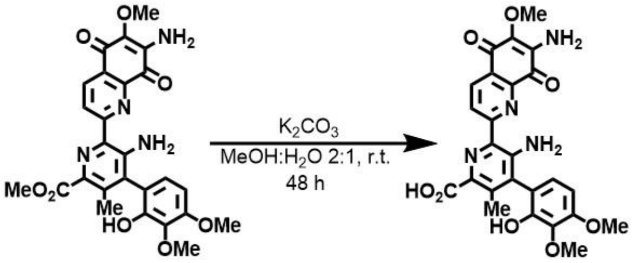

Methyl 5-amino-6-(7-amino-6-methoxy-5,8-dioxo-5,8-dihydroquinolin-2-yl)-4-(2-hydroxy-3,4-dimethoxyphenyl)-3-methylpicolinate (35 mg, 0.067 mmol, 1 equiv) and potassium carbonate (K_2CO_3, 200 mg, 1.45 mmol, 22 equiv.) were dissolved in a mixture of MeOH:H_2O 2:1 (12 mL) and the mixture was stirred for 48 h at room temperature under an argon atmosphere. Then, MeOH was evaporated *in vacuo* at ***35 °C***. The residue was neutralized with 1 M HCl (*the aqueueos phase will turn yellow as it is acidified*) and extracted with DCM (3 x 15 mL). The organic extract was dried over Na_2SO_4, filtered, and evaporated to dryness *in vacuo*. The reaction crude was purified by column chromatography on pH (6.8) buffered silica gel with a mixture of DCM:MeOH 98:2 as eluent to afford the product as a dark-brown solid (14 mg, 65%).

<u>*Note 1:*</u> It is important to evaporate the methanol at a temperature ***no higher than 35 °C***.

<u>*Note 2:*</u> The silica gel used for the purification was stirred for 5 min with a commercial phosphate buffer solution (pH = 6.8) and filtered. Then, the buffered silica gel was kept in an oven until use.

<u>*Note 3:*</u> Our attempts to obtain reproducible results for the optical rotation measurement of streptonigrin failed, likely because of its limited solubility.

^{1}H NMR (400 MHz, *d^6*-DMSO): δ 12.28 (s, 1H), 9.01 (d, *J* = 8.4 Hz, 1H), 8.92 (s, 1H), 8.36 (d, *J* = 8.4 Hz, 1H), 6.91 (s, 2H), 6.74 (d, *J* = 8.5 Hz, 1H), 6.70 (d, *J* = 8.6 Hz, 1H), 3.85 (s, 3H), 3.82 (s, 3H), 3.76 (s, 3H), 2.18 (s, 3H).
^{13}C NMR (101 MHz, *d^6*-DMSO): δ 180.3, 175.9, 167.0, 159.9, 153.1, 148.1, 145.7, 144.1, 141.6, 136.9, 136.2, 135.7, 134.6, 134.0, 133.4, 129.5, 126.71 126.0, 124.6, 114.9, 104.4, 60.3, 59.7, 55.7, 17.0.
HRMS: (ESI) *m/z* $[M+H]^+$ calculated for $C_{25}H_{23}N_4O_8$ 507.1510, found 507.1523.

Comparison of ^{1}H and ^{13}C NMR data for natural and synthetic streptonigrin

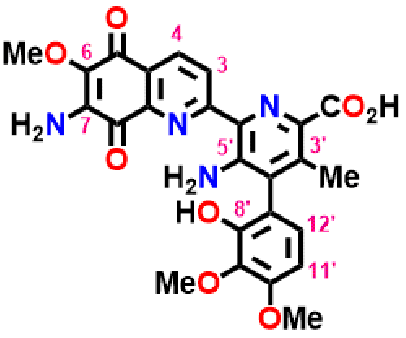

Atom Number	^{1}H NMR (d^{6} – DMSO)			^{13}C NMR (d^{6} – DMSO)		
	Harrity	*Donohoe*	*Literature*	*Harrity*	*Donohoe*	*Literature*
**1**	–	–	–	–	–	–
**2**	9.01 (d, 8.4)	9.01 (d, 8.4)	9.01 (d)	144.1	144.1	144.1
**3**	8.36 (d, 8.4)	8.36 (d, 8.5)	8.36 (d)	126.0	125.9	125.9
**4**	–	–	–	133.4	133.4	133.4
**4a**	–	–	–	126.7	126.7	126.7
**5**	–	–	–	175.9	175.9	175.9
**6**	–	–	–	135.7	135.7	135.7
**6–OMe**	3.82 (s)	3.81 (s)	3.81 (s)	59.7	59.7	59.7
**7**	–	–	–	141.6	141.6	141.6
**7–NH_2**	6.91 (s, br)	6.92 (s, br)	6.93 (s, br)	–	–	–
**8**	–	–	–	180.3	180.3	180.3
**8a**	–	–	–	159.9	159.8	159.8
**1’**	–	–	–	–	–	–
**2’**	–	–	–	136.2	136.2	136.2
**2’–CO_2H**	12.28 (s, br)	12.32 (s, br)	12.22 (s, br)	167.0	167.0	167.1
**3’**	–	–	–	134.6	134.5	134.8
**3’–Me**	2.18 (s)	2.18 (s)	2.17 (s)	17.0	17.0	17.0
**4’**	–	–	–	134.0	134.0	133.9
**5’**	–	–	–	145.7	145.7	145.7
**6’**	–	–	–	129.5	129.5	129.5
**7’**	–	–	–	114.9	114.9	114.8
**8’**	–	–	–	148.1	148.1	148.1
**8’–OH**	8.92 (s)	8.92 (s)	8.94 (s, br)	–	–	–
**9’**	–	–	–	136.9	136.9	136.9
**9’–OMe**	3.76 (s)	3.76 (s)	3.76 (s)	60.3	60.3	60.3
**10’**	–	–	–	153.1	153.1	153.1
**10’–OMe**	3.85 (s)	3.85 (s)	3.85 (s)	55.7	55.7	55.7
**11’**	6.70 (d, 8.5)	6.70 (d, 8.6)	6.70 (d)	104.4	104.4	104.4
**12’**	6.74 (d, 8.5)	6.73 (d, 8.5)	6.73 (d)	124.6	124.6	124.6

NMR Spectra

^{1}H and ^{13}C NMR spectra of (R)-3,3'-bis(cyanomethyl)-1,1'-binaphthyl-2,2'-diol (S6)

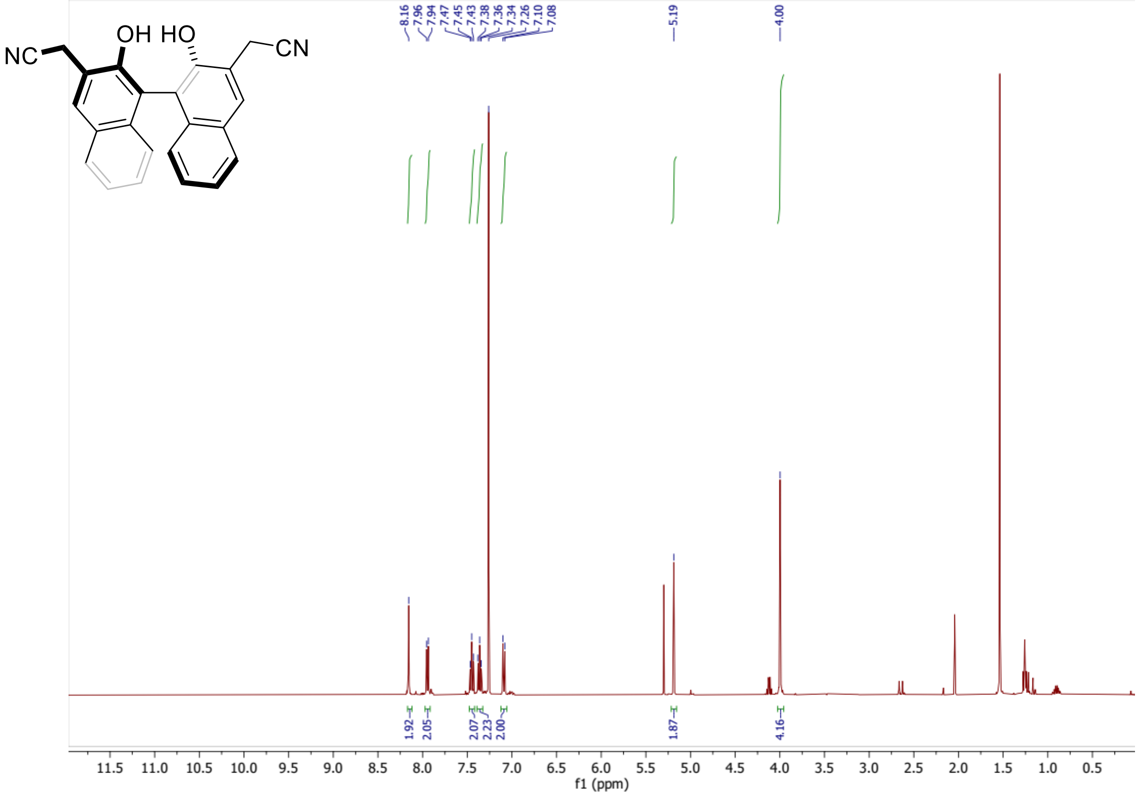

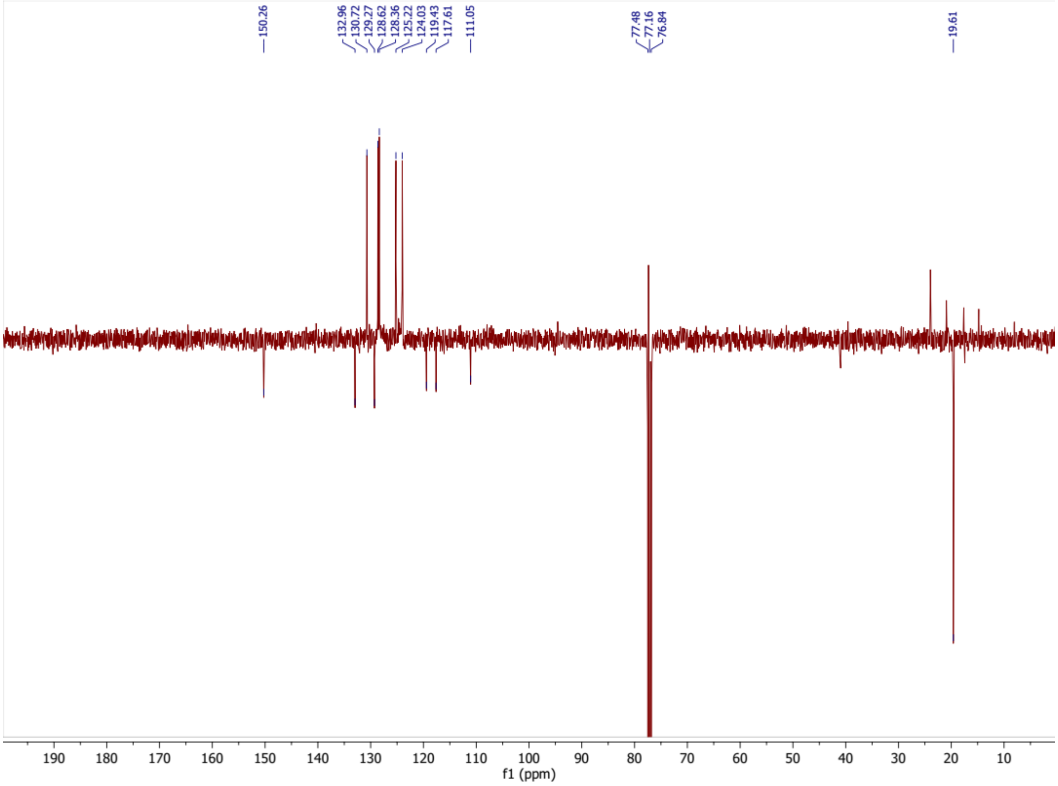

^{1}H, ^{13}C, ^{11}B, and ^{19}F NMR spectra of 10-(2-ethylphenyl)-11,11-difluoro-8,9-dimethyl-11H-pyrido[3',2',3,4][1,2]azaborolo[1,5-a]quinolin-12-ium-11-uide (9a)

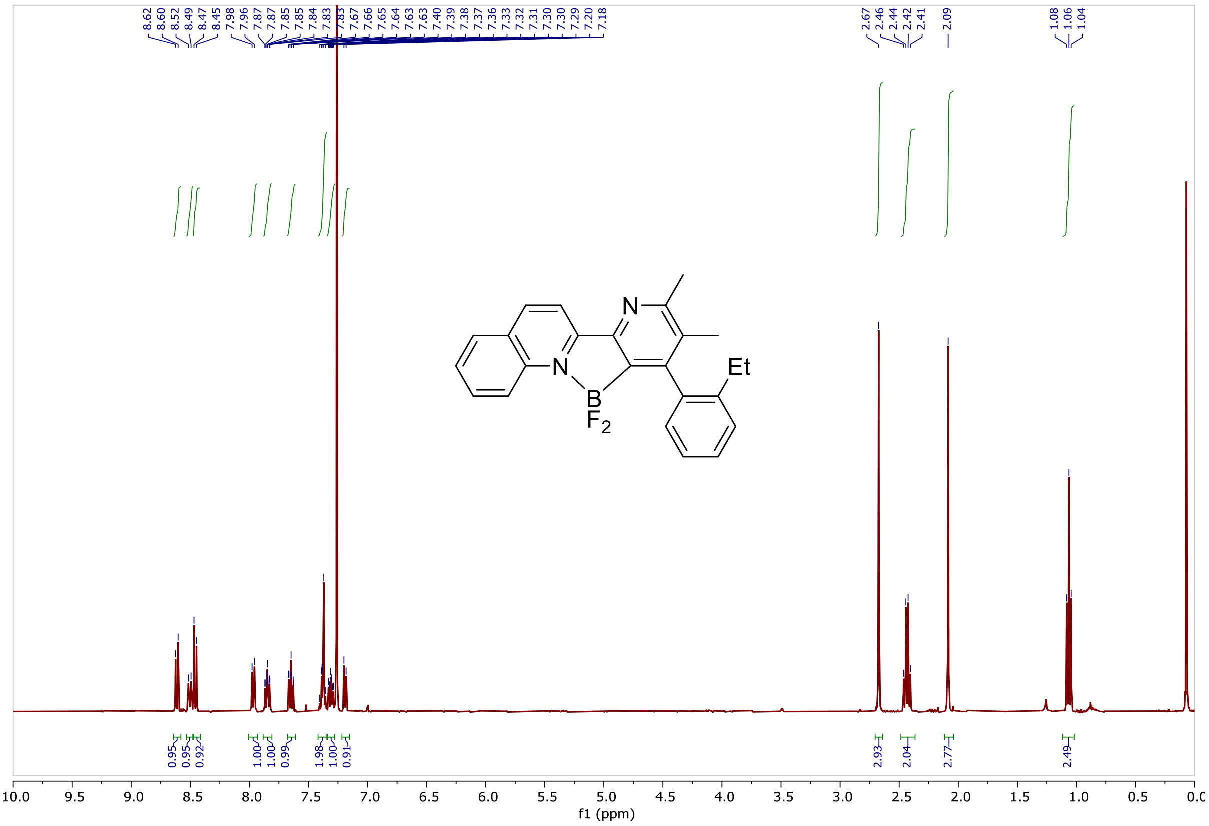

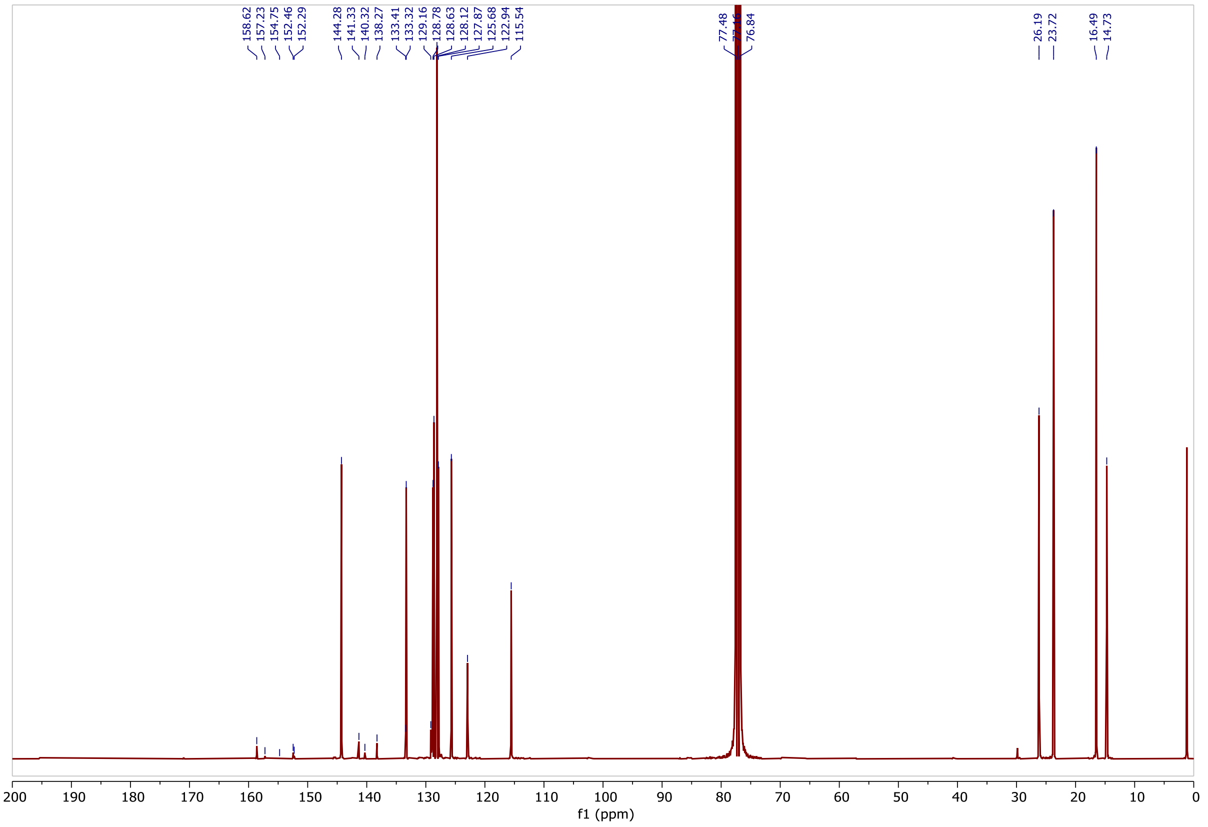

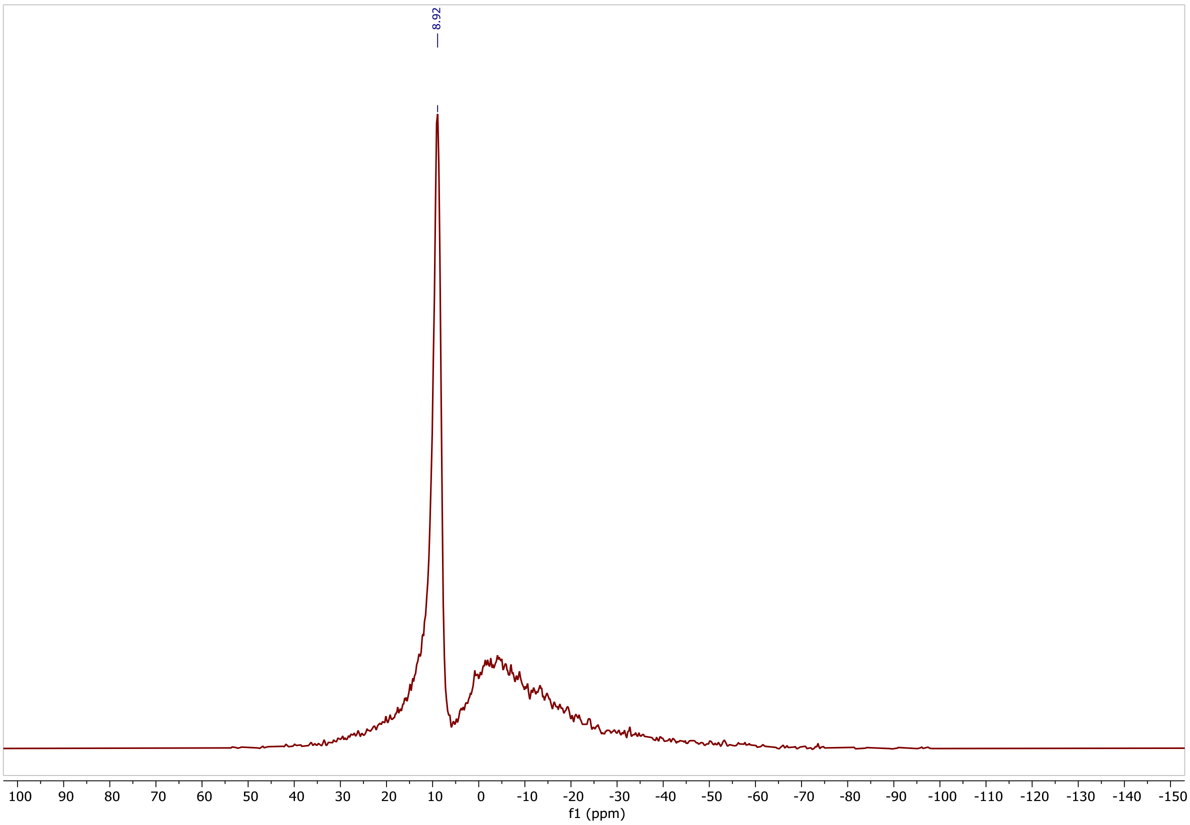
8.92
100
90
80
70
60
50
40
30
20
10
0
-10
-20
-30
-40
-50
-60
-70
-80
-90
-100
-110
-120
-130
-140
-150
f1 (ppm)

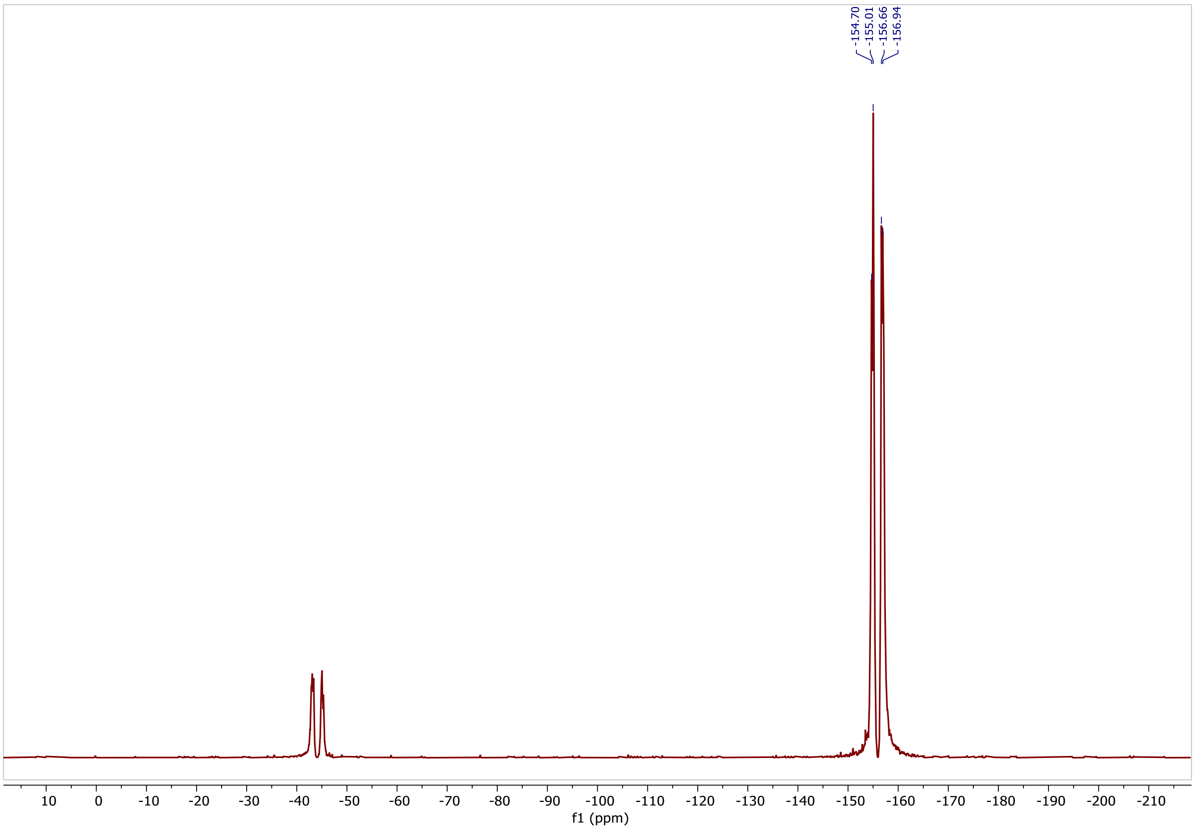
-154.70
-155.01
-156.66
-156.94
10
0
-10
-20
-30
-40
-50
-60
-70
-80
-90
-100
-110
-120
-130
-140
-150
-160
-170
-180
-190
-200
-210
f1 (ppm)

^{1}H, ^{13}C, ^{11}B, and ^{19}F NMR spectra of 11,11-difluoro-10-(2-methoxyphenyl)-8,9-dimethyl-11H-pyrido[3',2':3,4][1,2]azaborolo[1,5-a]quinolin-12-ium-11-uide (9b)

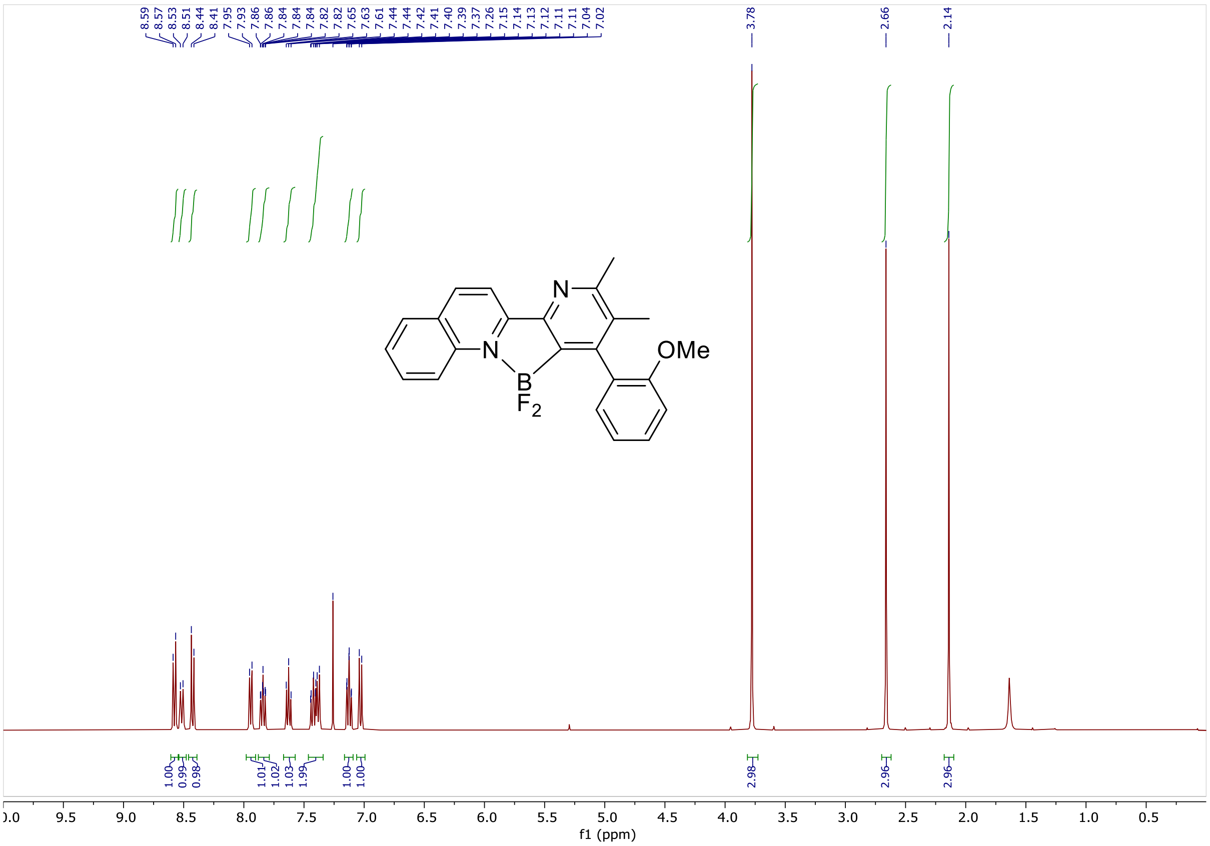

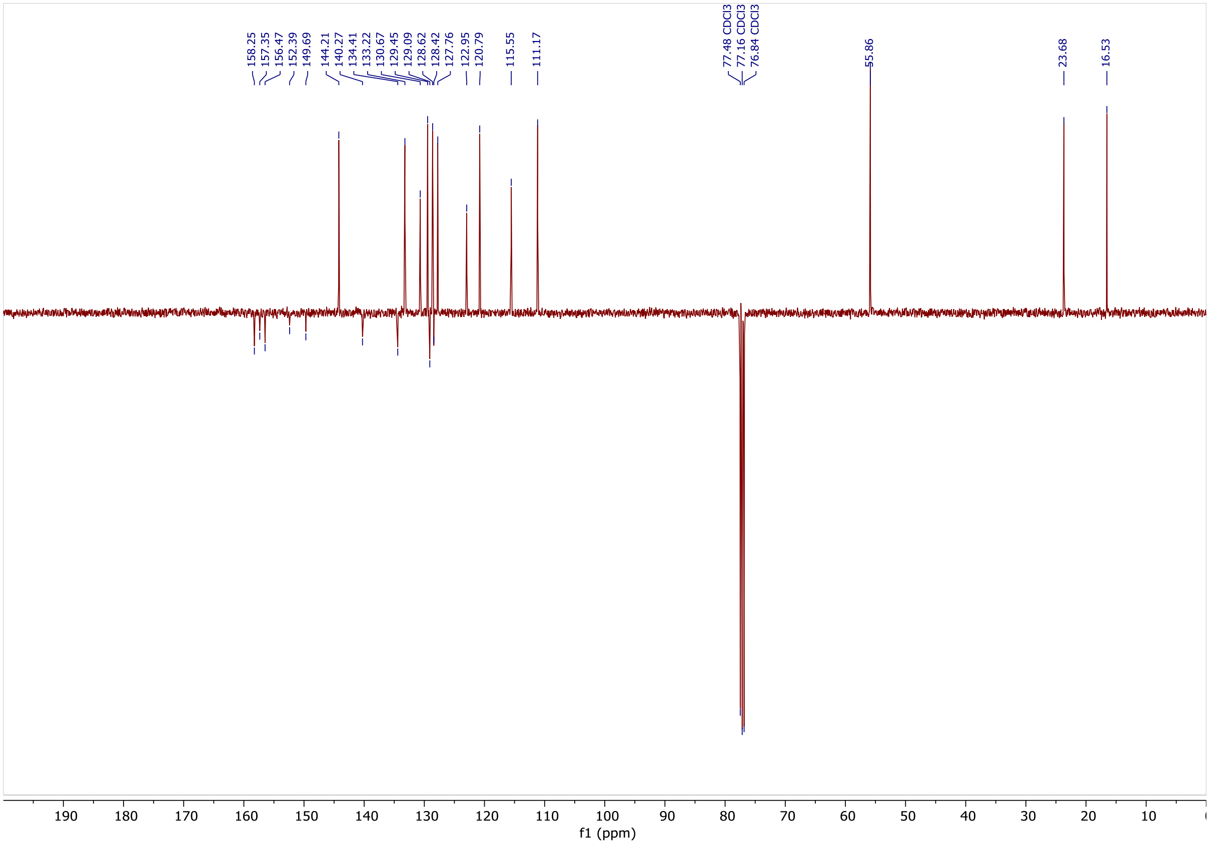

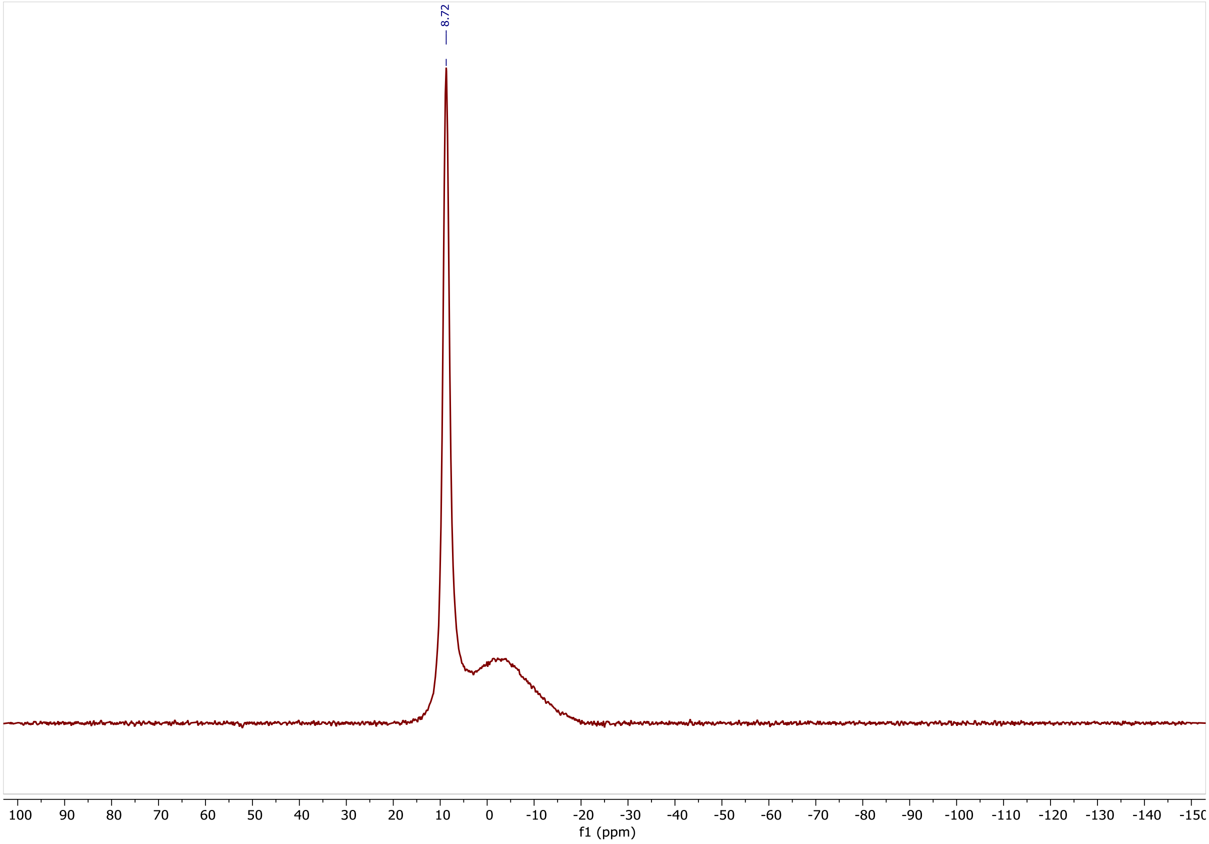
8.72
100 90 80 70 60 50 40 30 20 10 0 -10 -20 -30 -40 -50 -60 -70 -80 -90 -100 -110 -120 -130 -140 -150
f1 (ppm)

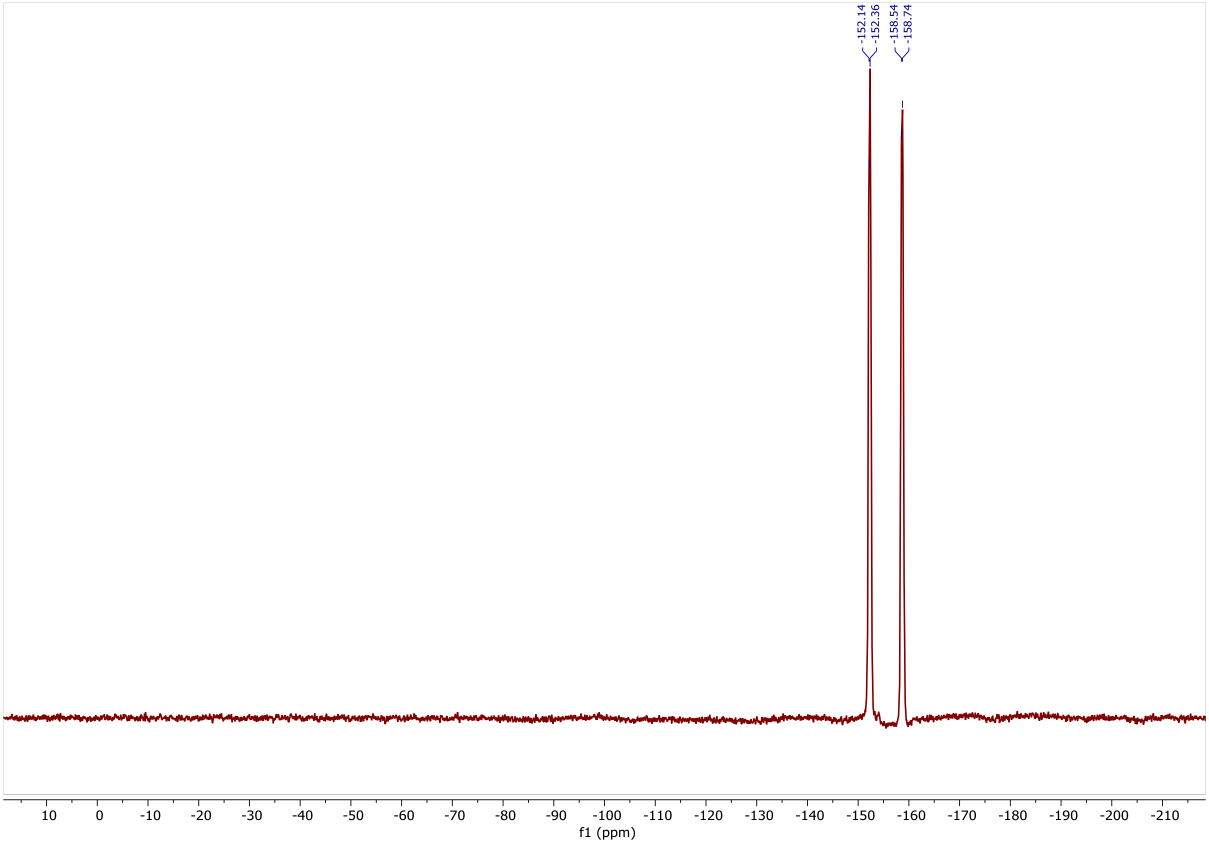
-152.14
-152.36
-158.54
-158.74
10 0 -10 -20 -30 -40 -50 -60 -70 -80 -90 -100 -110 -120 -130 -140 -150 -160 -170 -180 -190 -200 -210
f1 (ppm)

^{1}H, ^{13}C, ^{11}B, and ^{19}F NMR spectra of 10-(2-ethylphenyl)-11,11-difluoro-8,9-diphenyl-11H-pyrido[3',2':3,4][1,2]azaborolo[1,5-a]quinolin-12-ium-11-uide (9c)

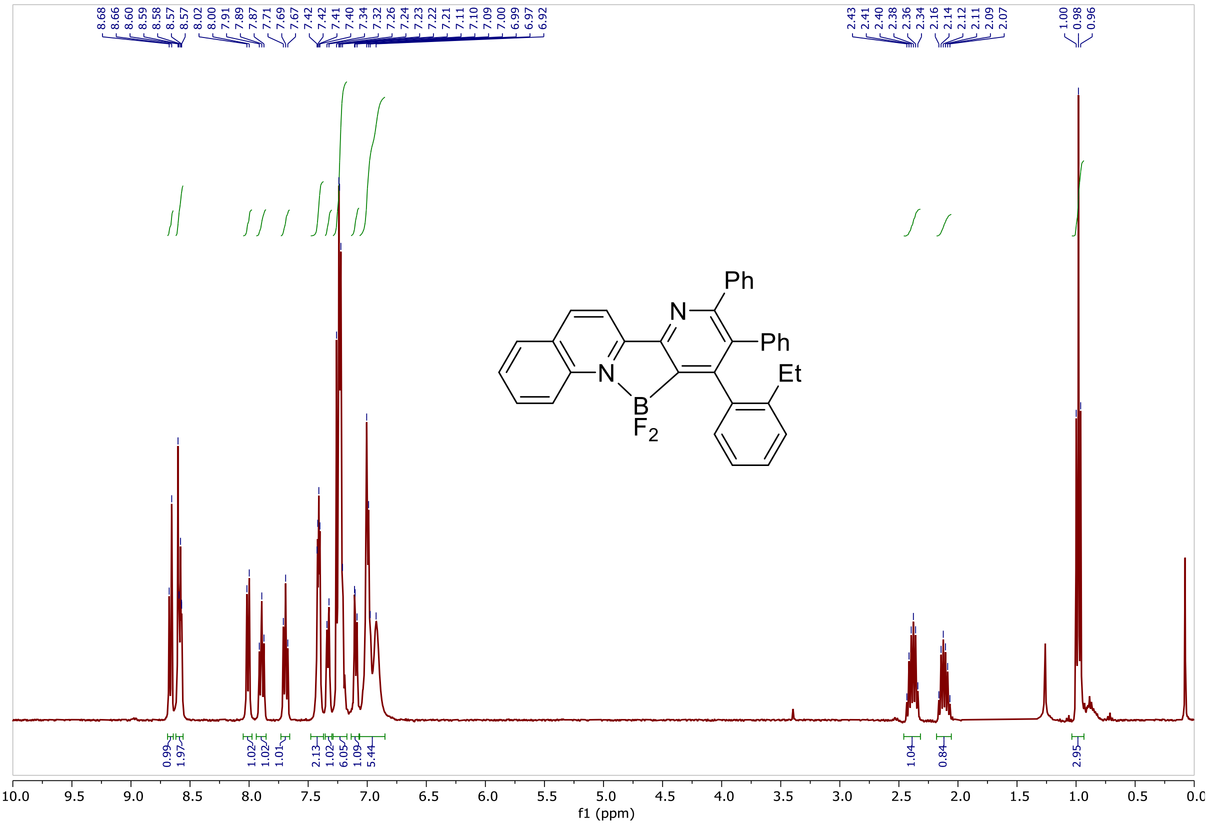

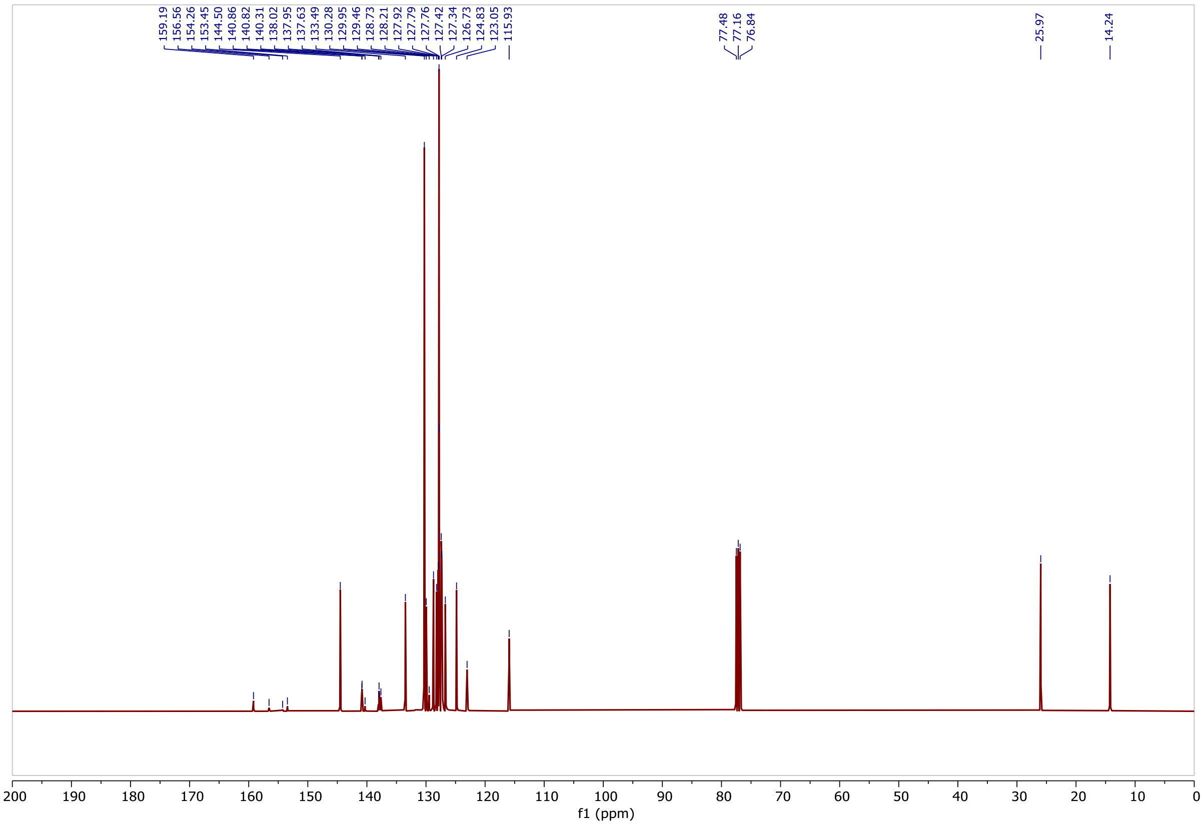

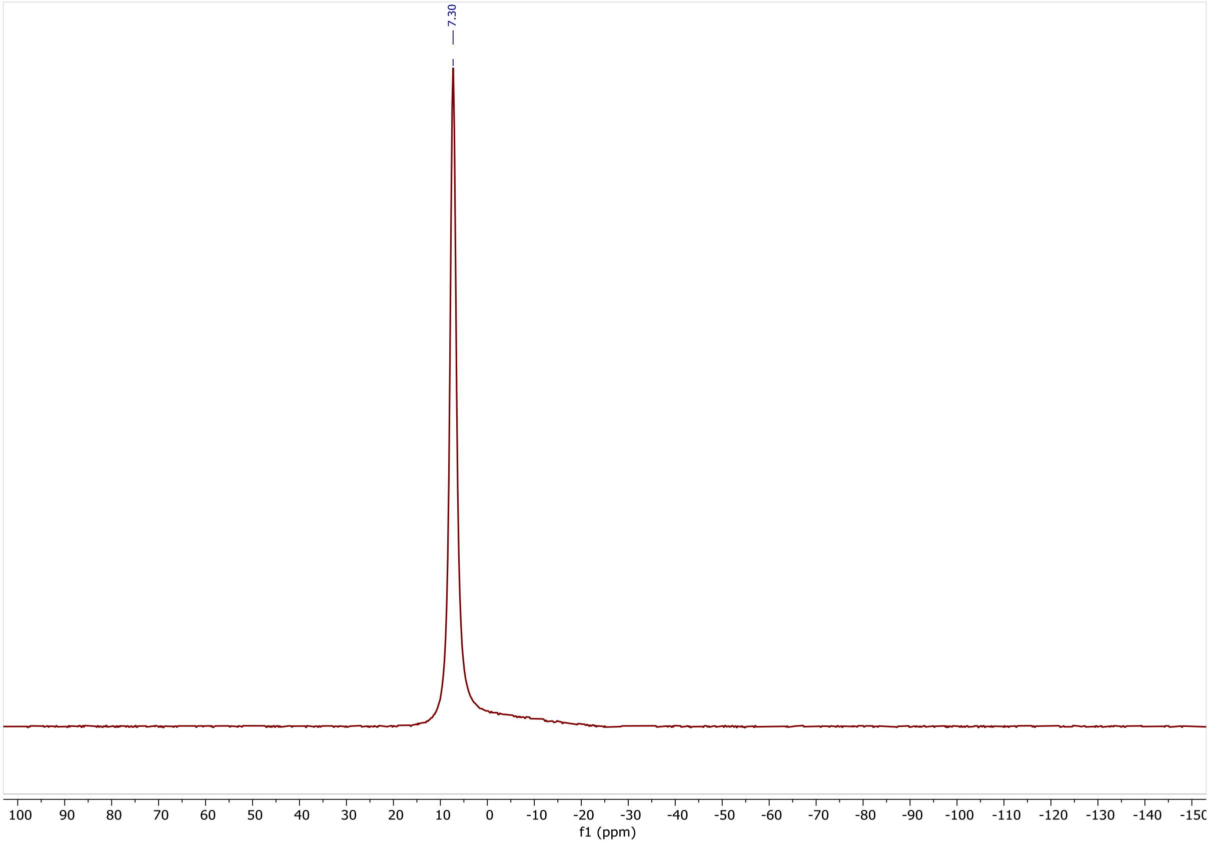
7.30
100 90 80 70 60 50 40 30 20 10 0 -10 -20 -30 -40 -50 -60 -70 -80 -90 -100 -110 -120 -130 -140 -150
f1 (ppm)

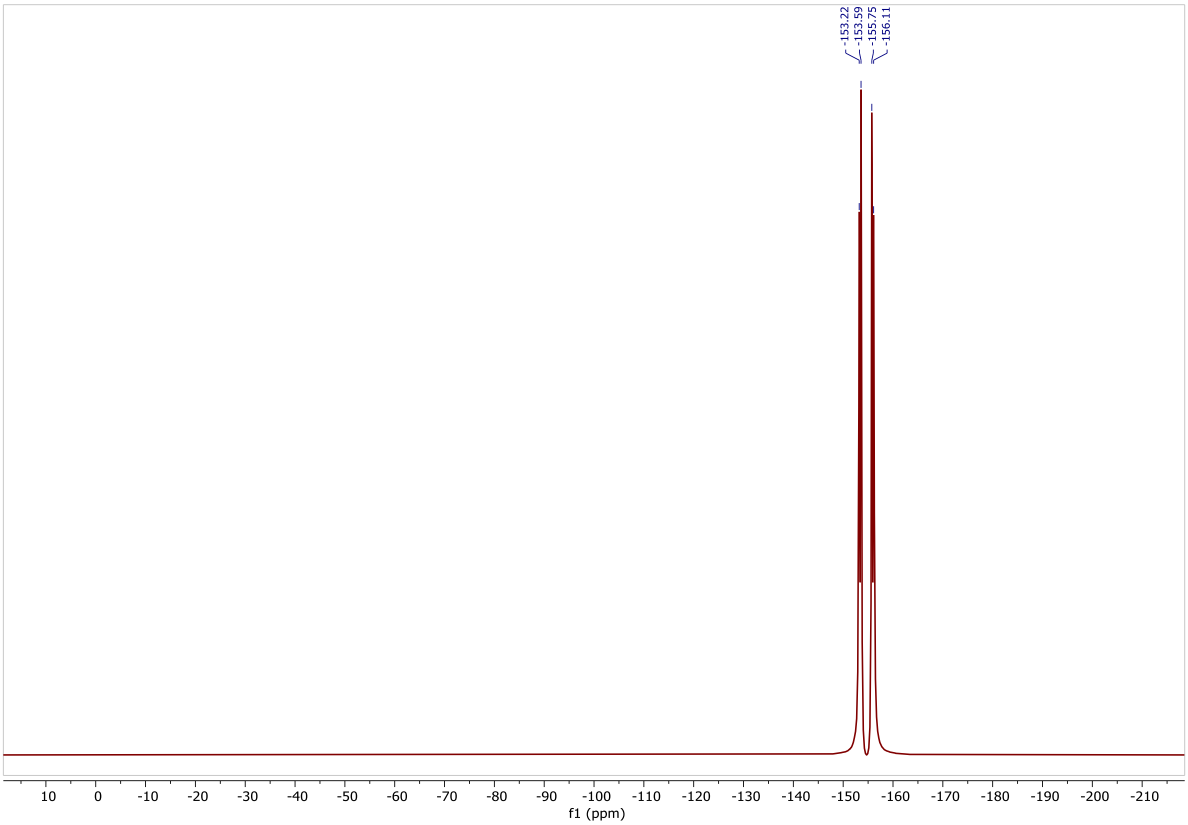
-153.22
-153.59
-155.75
-156.11
10 0 -10 -20 -30 -40 -50 -60 -70 -80 -90 -100 -110 -120 -130 -140 -150 -160 -170 -180 -190 -200 -210
f1 (ppm)

^{1}H, ^{13}C, ^{11}B, and ^{19}F NMR spectra of 11,11-difluoro-10-(2-methoxyphenyl)-8,9-diphenyl-11H-pyrido[3',2':3,4][1,2]azaborolo[1,5-a]quinolin-12-ium-11-uide (9d)

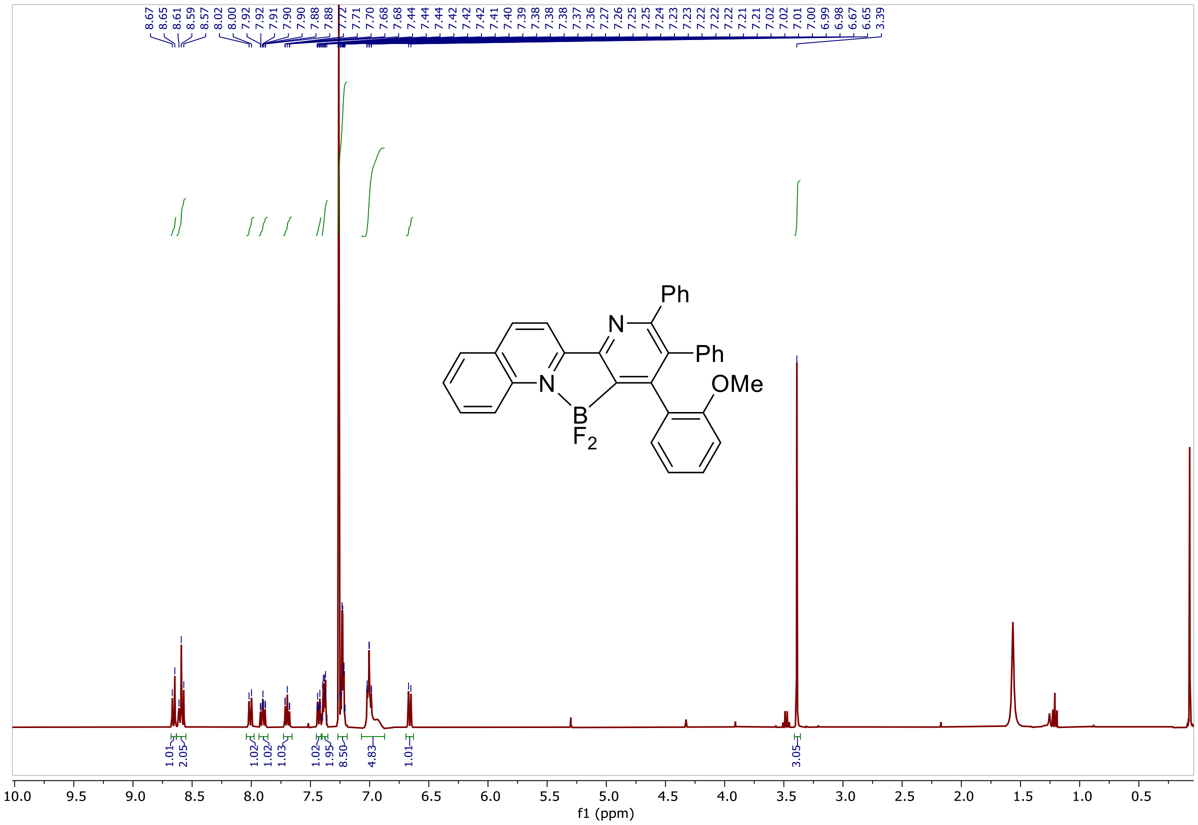

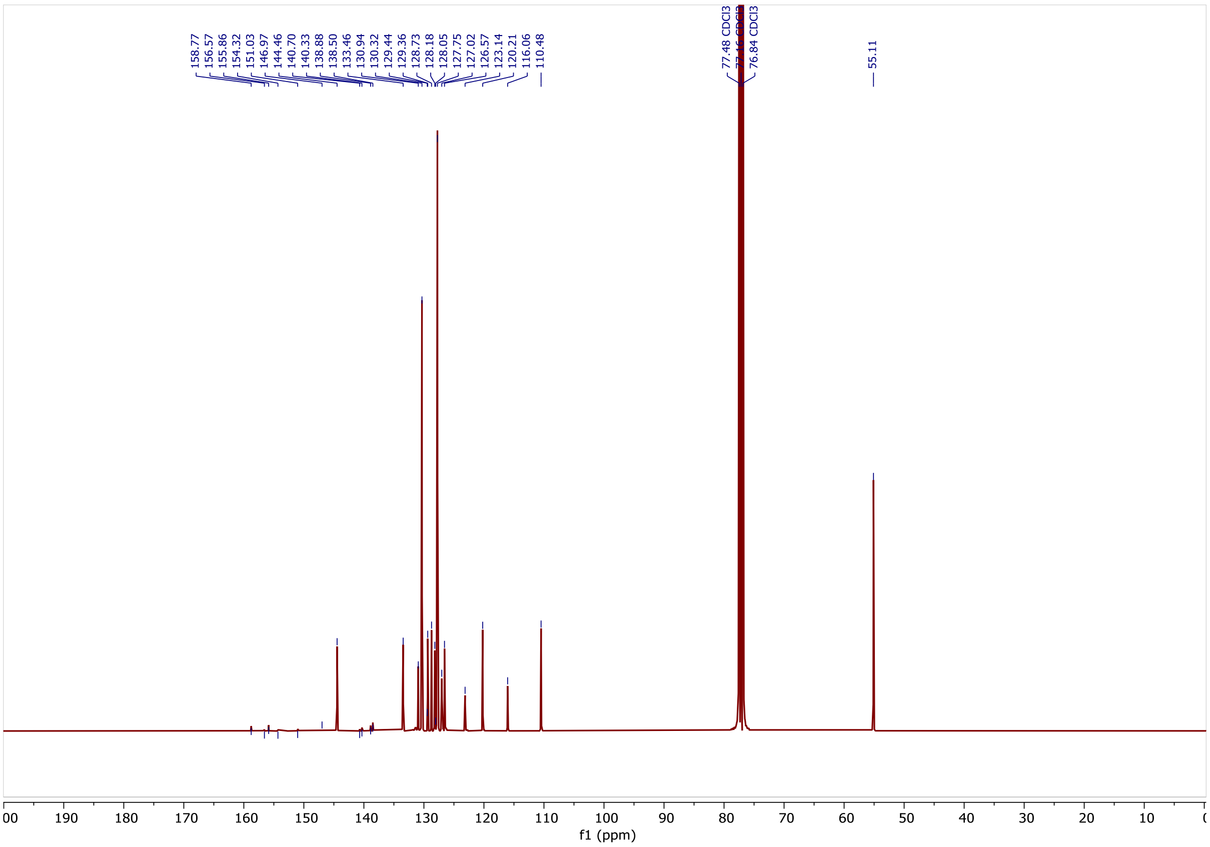

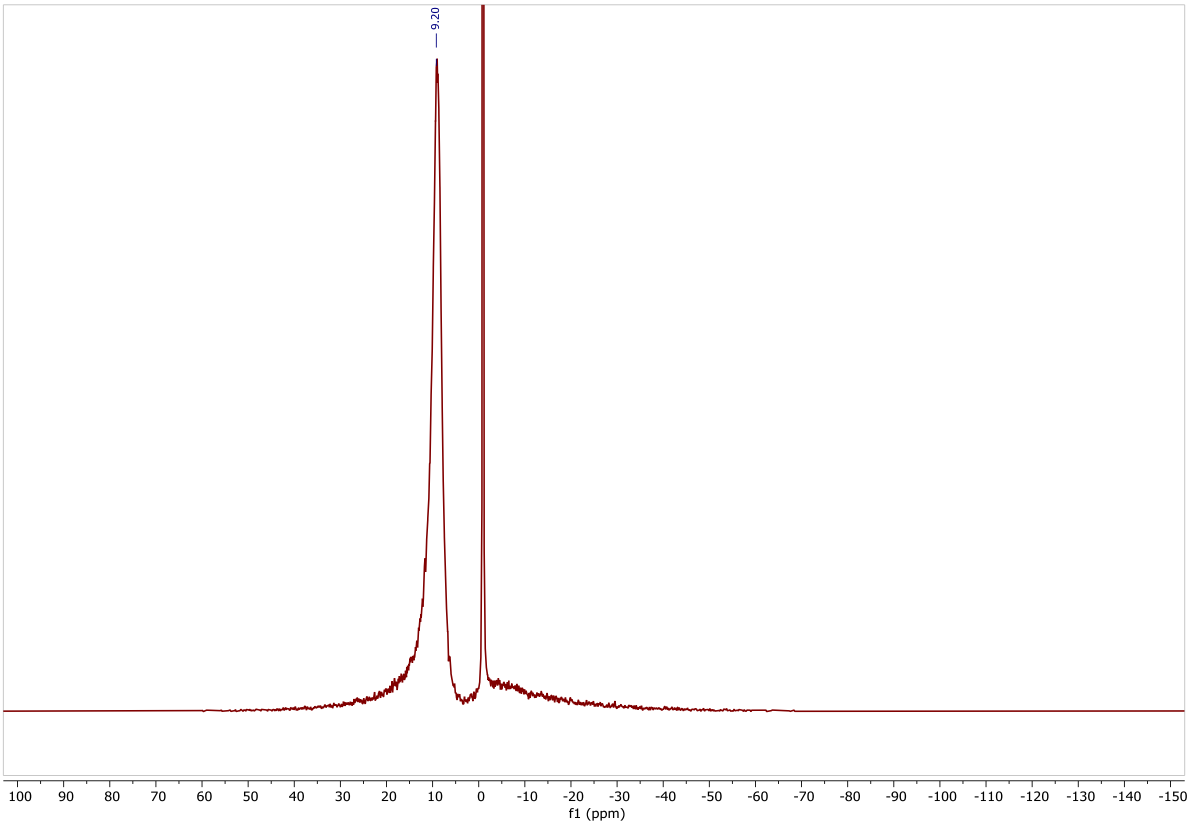
9.20
100 90 80 70 60 50 40 30 20 10 0 -10 -20 -30 -40 -50 -60 -70 -80 -90 -100 -110 -120 -130 -140 -150
f1 (ppm)

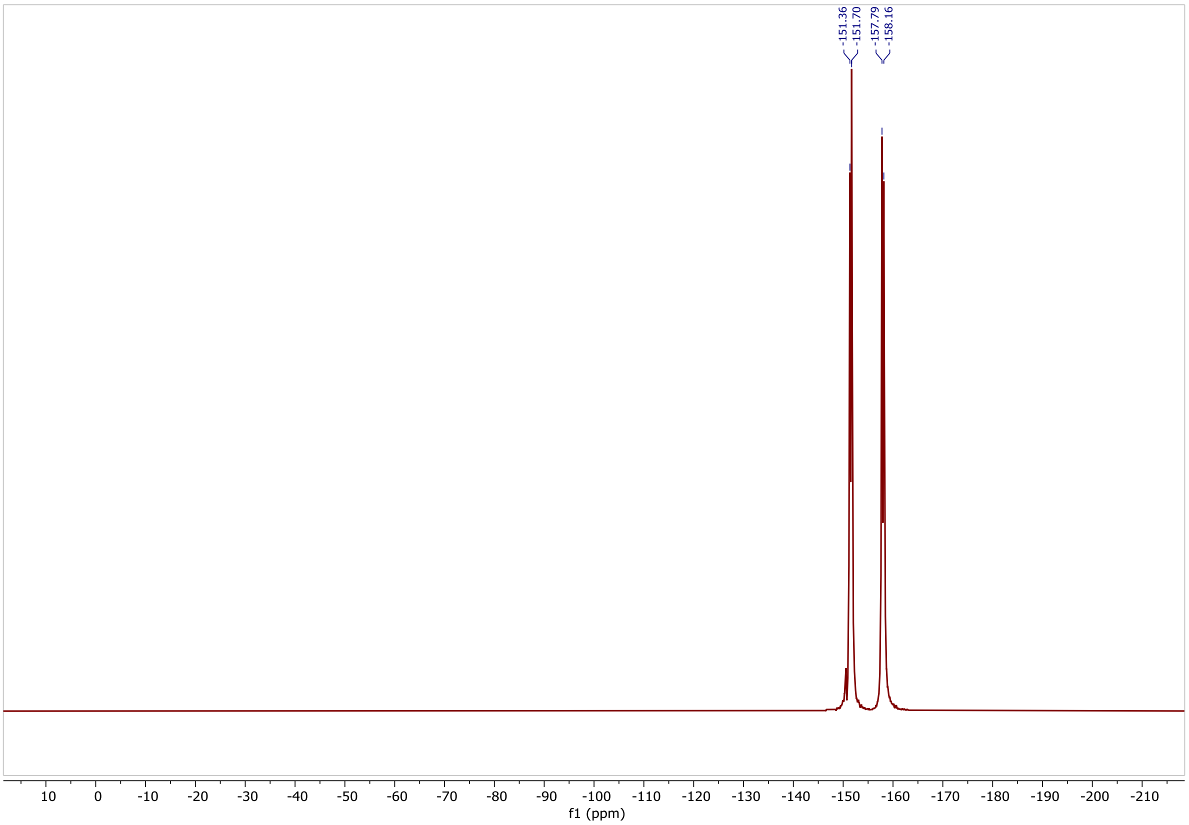
-151.36
-151.70
-157.79
-158.16
10 0 -10 -20 -30 -40 -50 -60 -70 -80 -90 -100 -110 -120 -130 -140 -150 -160 -170 -180 -190 -200 -210
f1 (ppm)

^{1}H, ^{13}C, ^{11}B, and ^{19}F NMR spectra of 11,11-difluoro-10-(2-isopropoxyphenyl)-8,9-dimethyl-11H-pyrido[3',2':3,4][1,2]azaborolo[1,5-a]quinolin-12-ium-11-uide (9e)

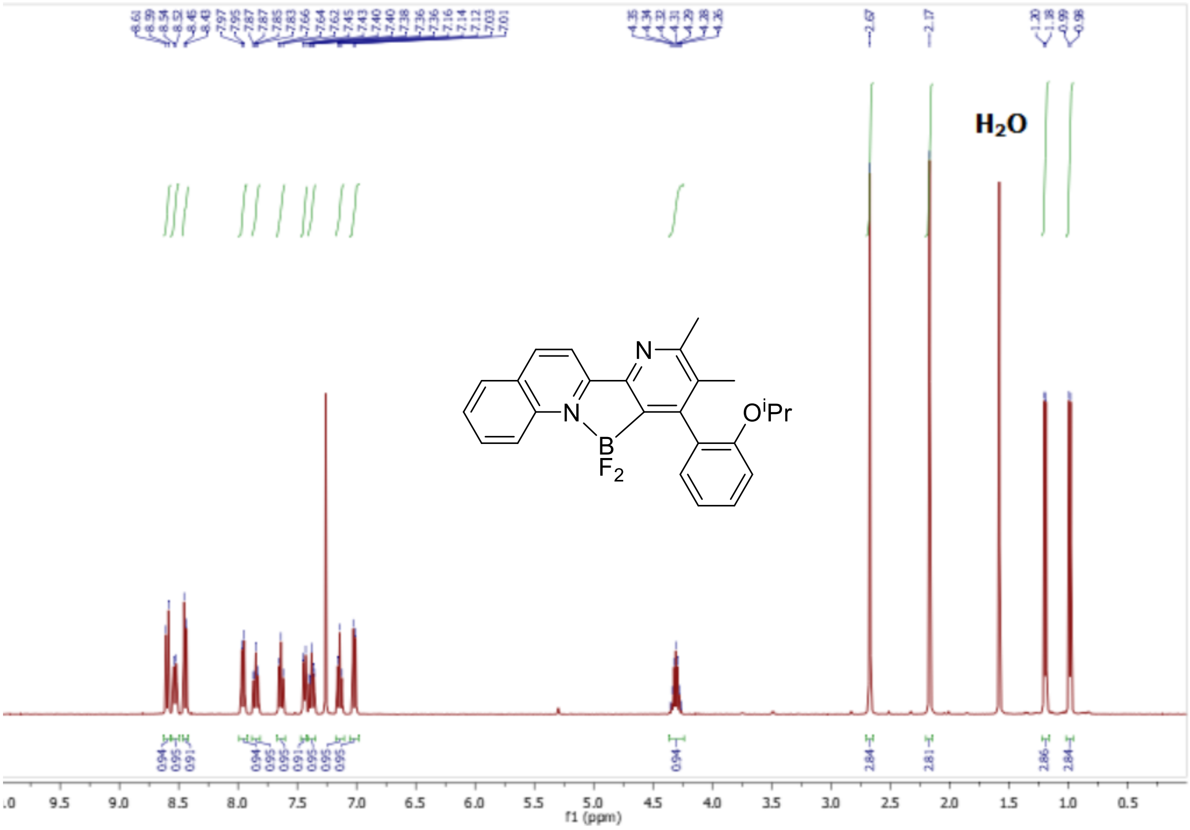

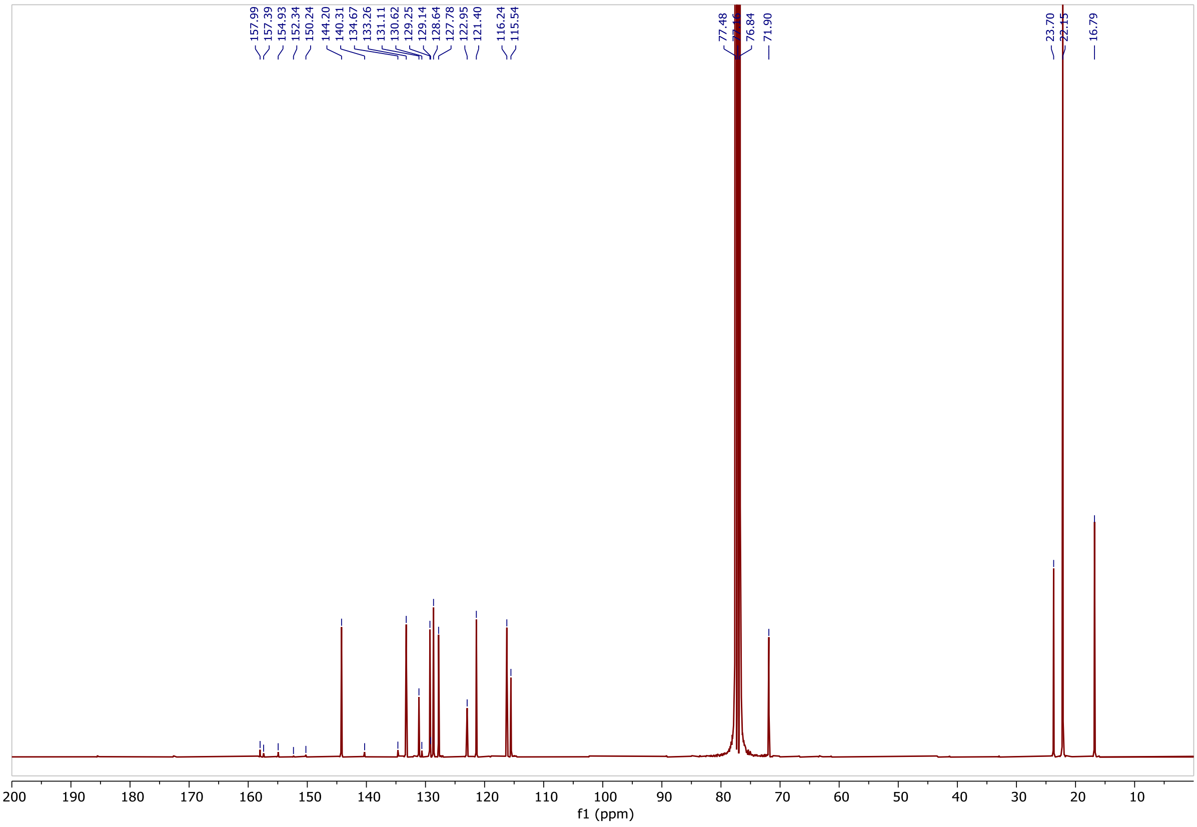

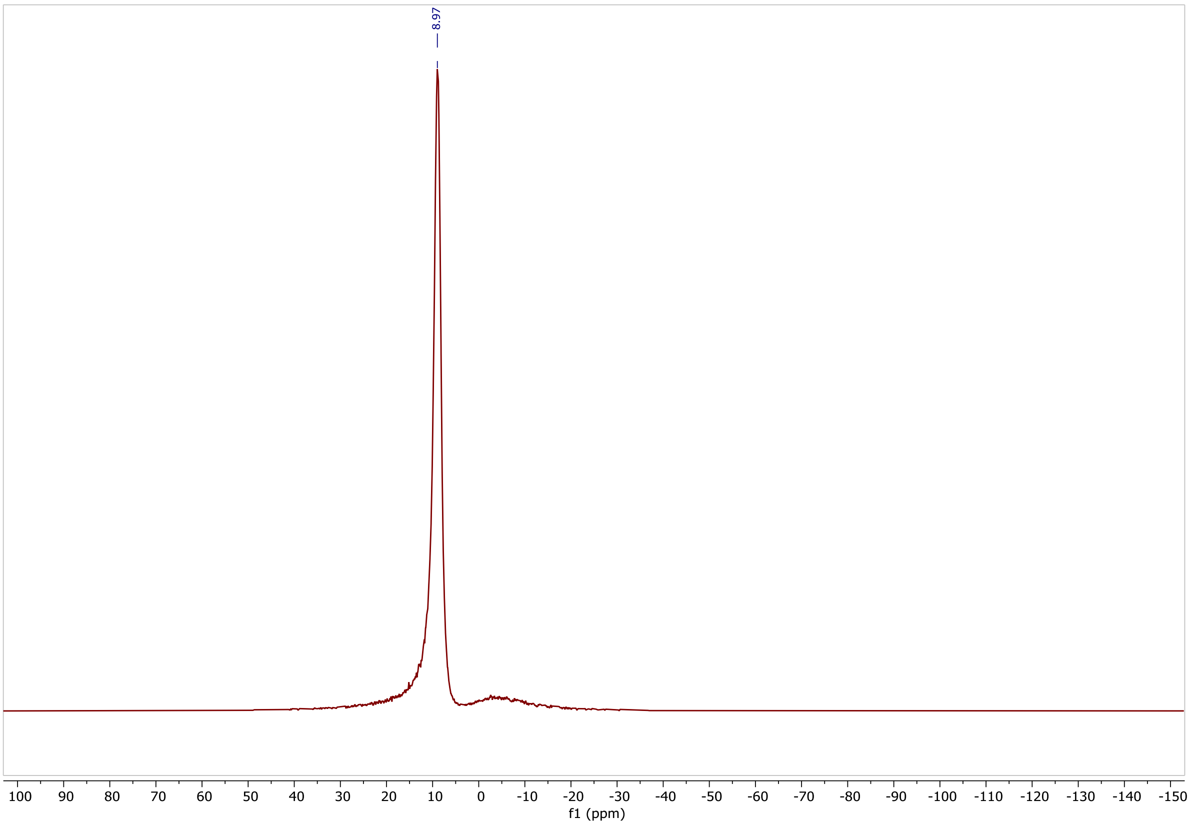
8.97
100
90
80
70
60
50
40
30
20
10
0
-10
-20
-30
-40
-50
-60
-70
-80
-90
-100
-110
-120
-130
-140
-150
f1 (ppm)

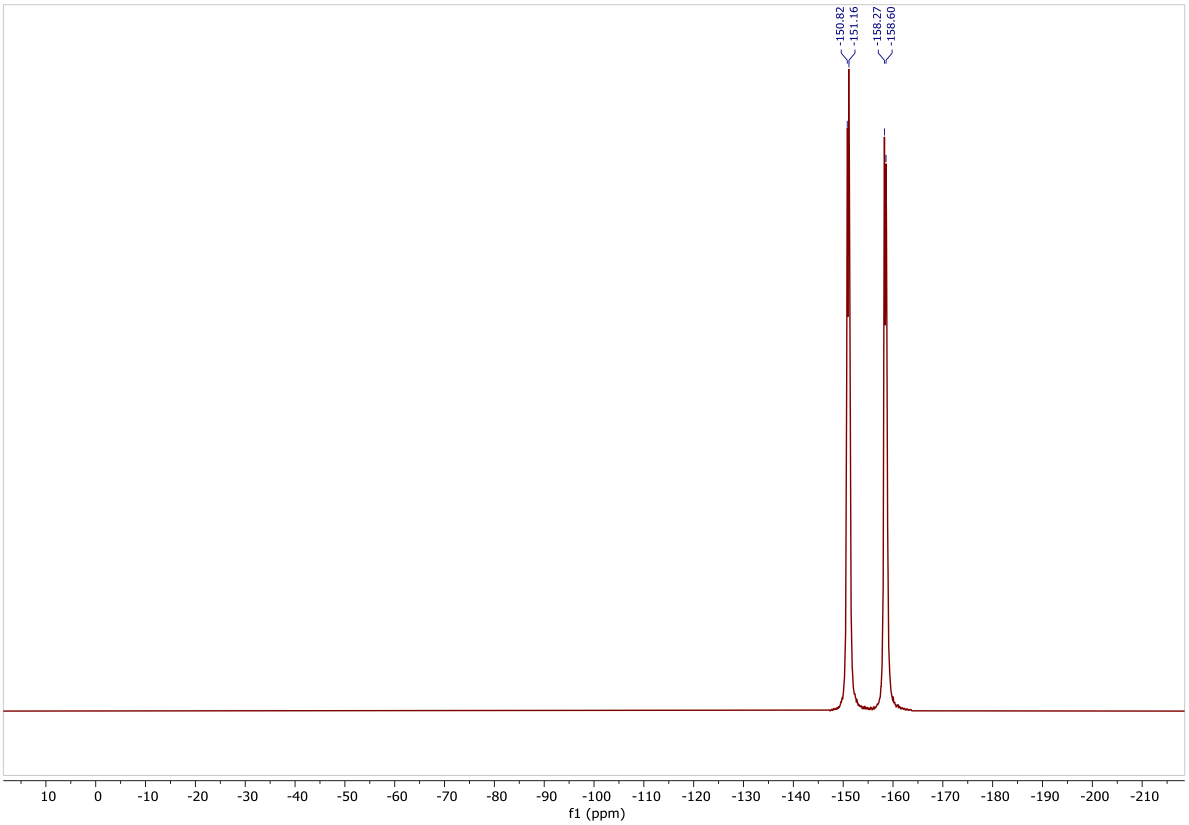
-150.82
-151.16
-158.27
-158.60
10
0
-10
-20
-30
-40
-50
-60
-70
-80
-90
-100
-110
-120
-130
-140
-150
-160
-170
-180
-190
-200
-210
f1 (ppm)

^{1}H, ^{13}C, ^{11}B, and ^{19}F NMR spectra of 10-(2-(dimethylamino)phenyl)-11,11-difluoro-8,9-dimethyl-11H-pyrido[3',2':3,4][1,2]azaborolo[1,5-a]quinolin-12-ium-11-uide (9f)

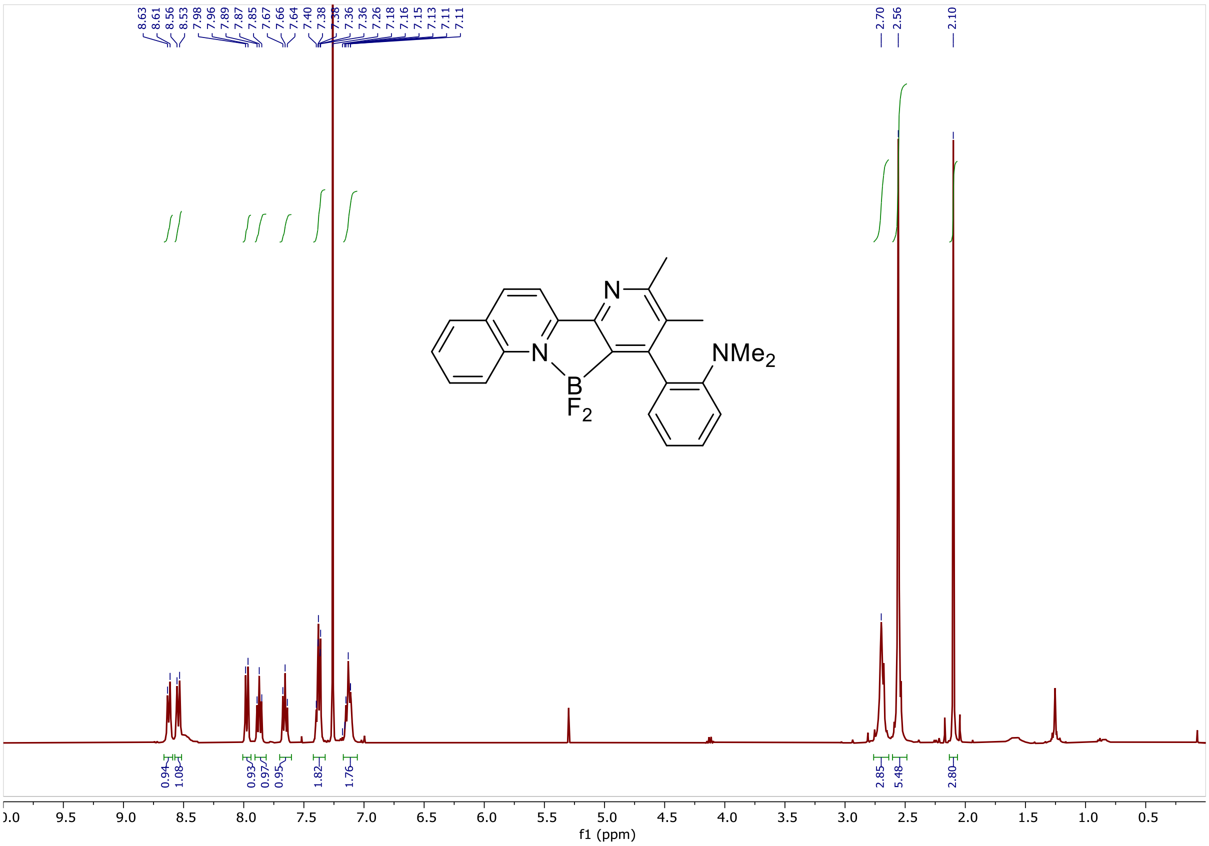

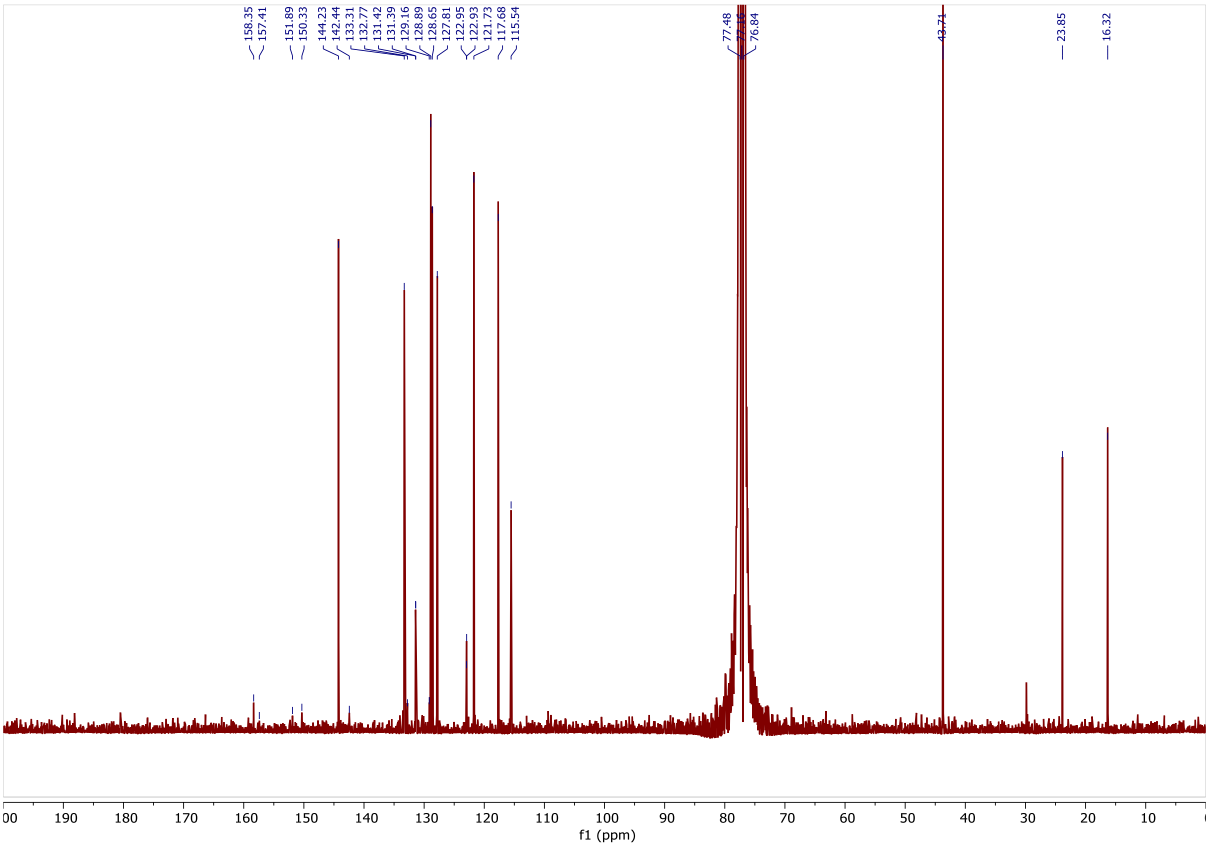

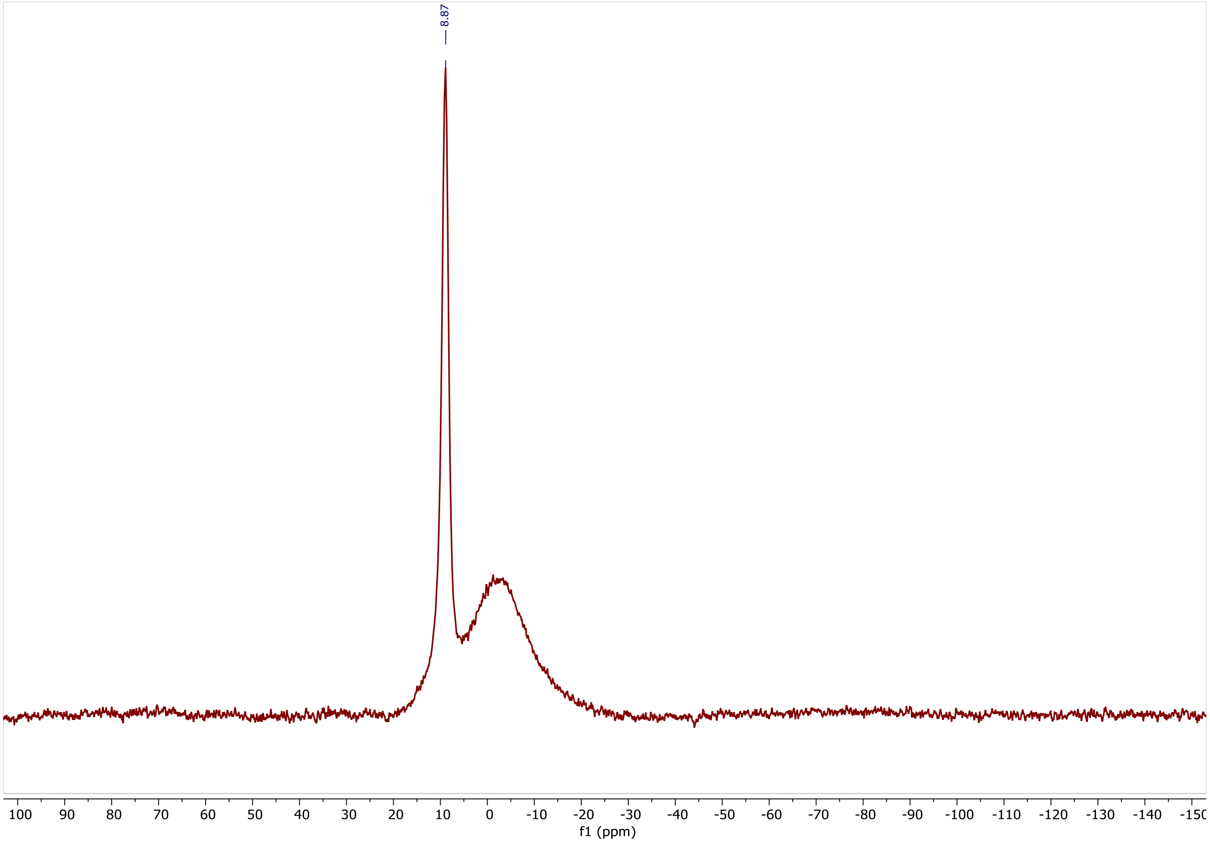
8.87
100
90
80
70
60
50
40
30
20
10
0
-10
-20
-30
-40
-50
-60
-70
-80
-90
-100
-110
-120
-130
-140
-150
f1 (ppm)

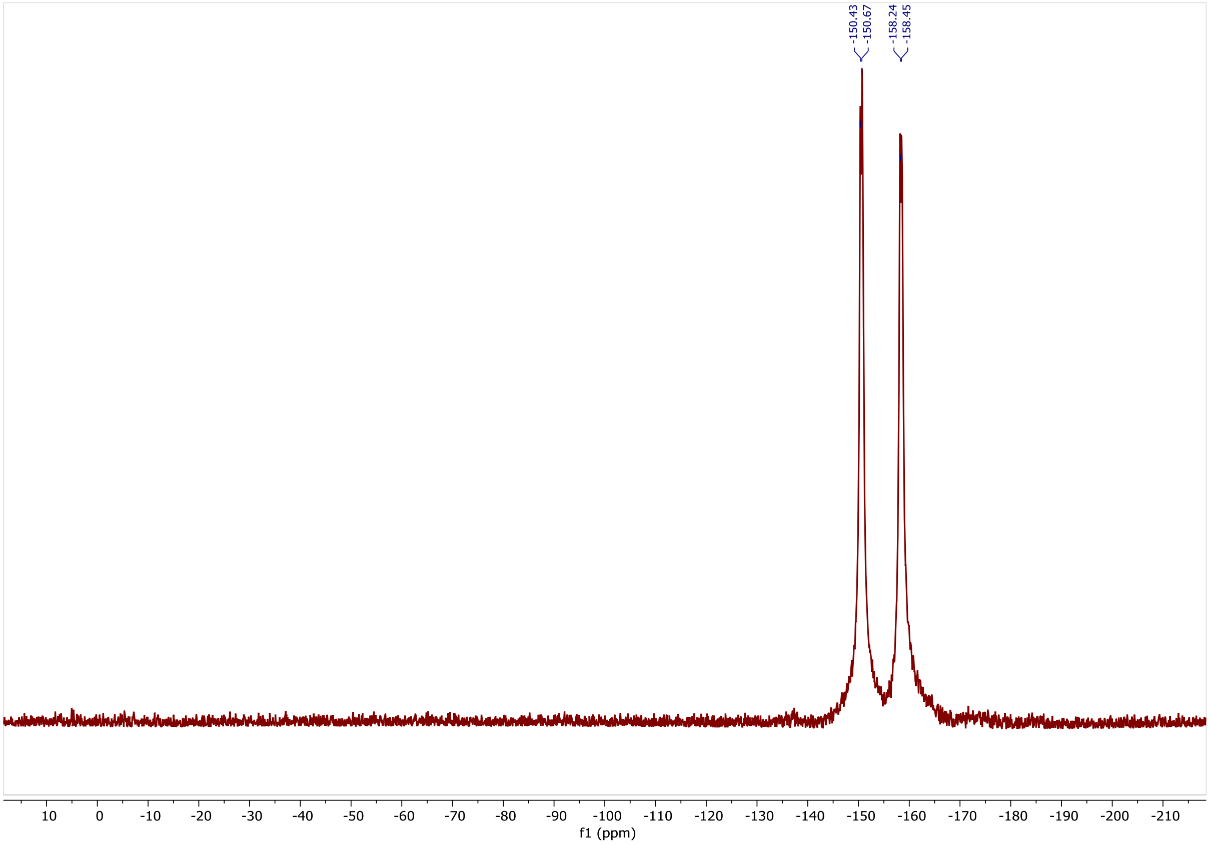
-150.43
-150.67
-158.24
-158.45
10
0
-10
-20
-30
-40
-50
-60
-70
-80
-90
-100
-110
-120
-130
-140
-150
-160
-170
-180
-190
-200
-210
f1 (ppm)

^{1}H, ^{13}C, ^{11}B, and ^{19}F NMR spectra of 11,11-difluoro-8,9-dimethyl-10-(2-(methylthio)phenyl)-11H-pyrido[3',2':3,4][1,2]azaborolo[1,5-a]quinolin-12-ium-11-uide (9g)

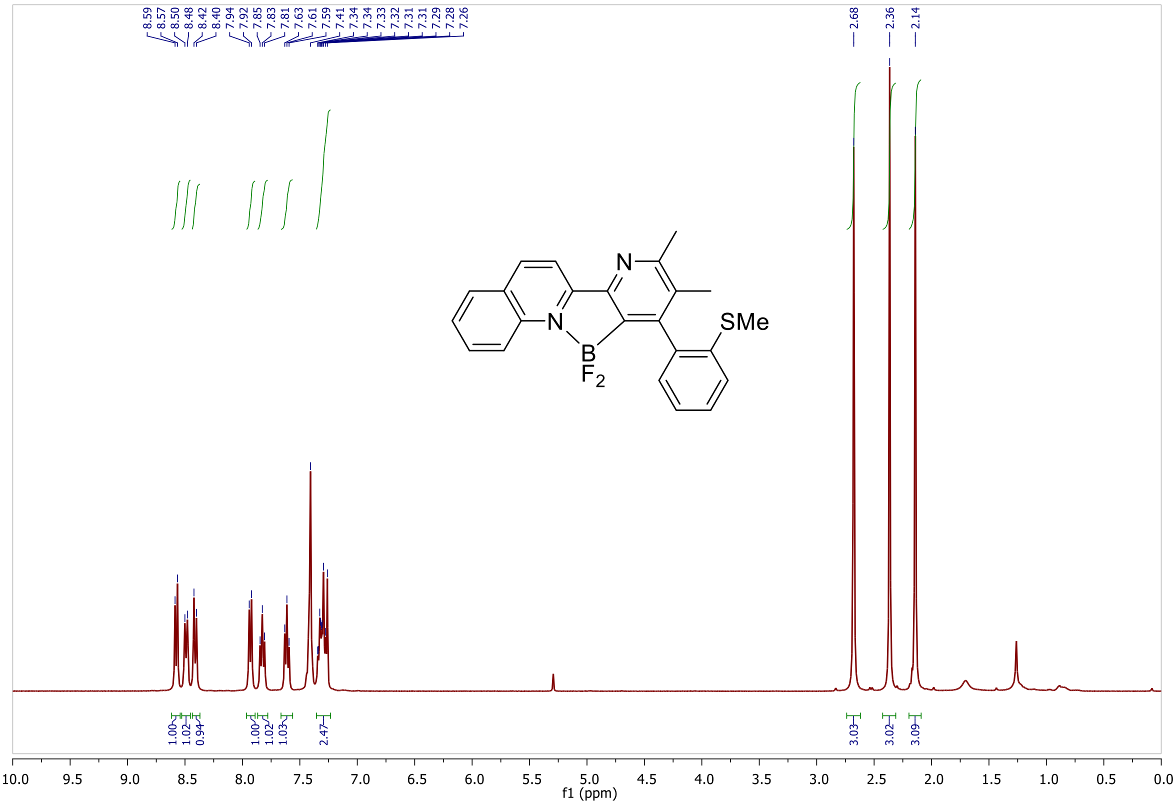

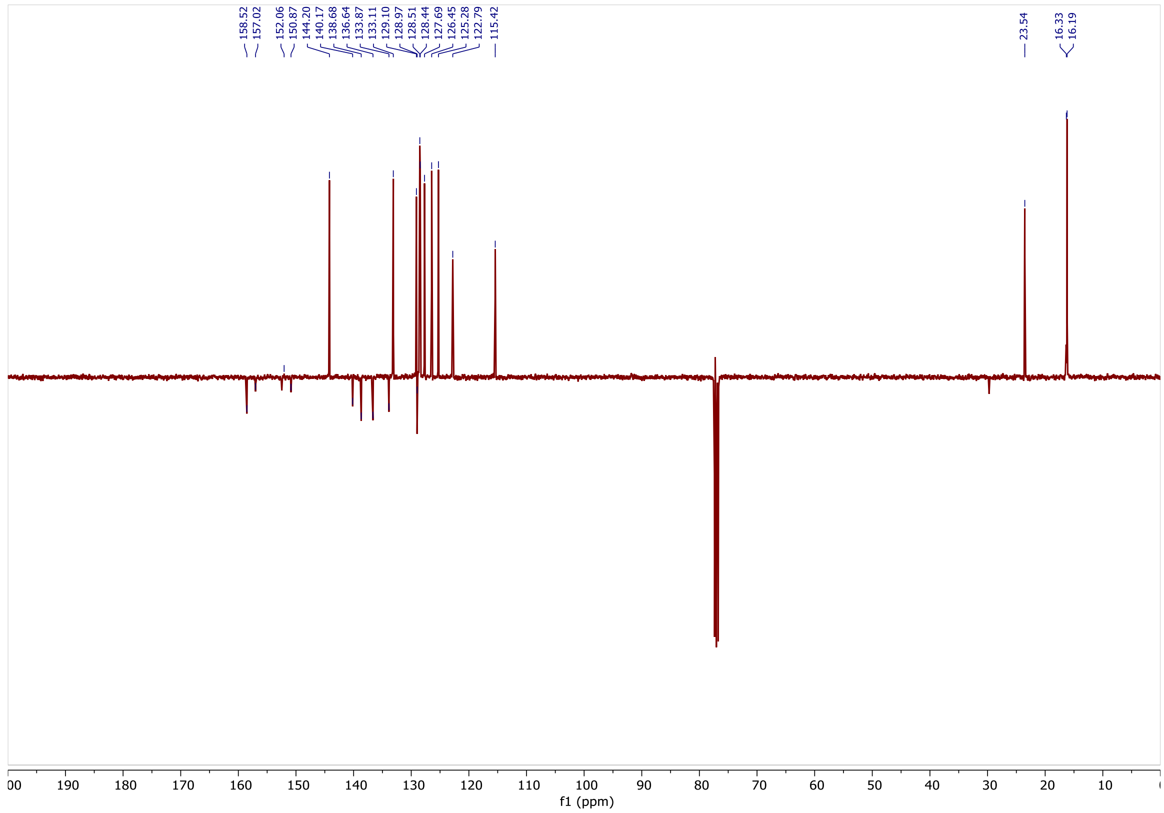

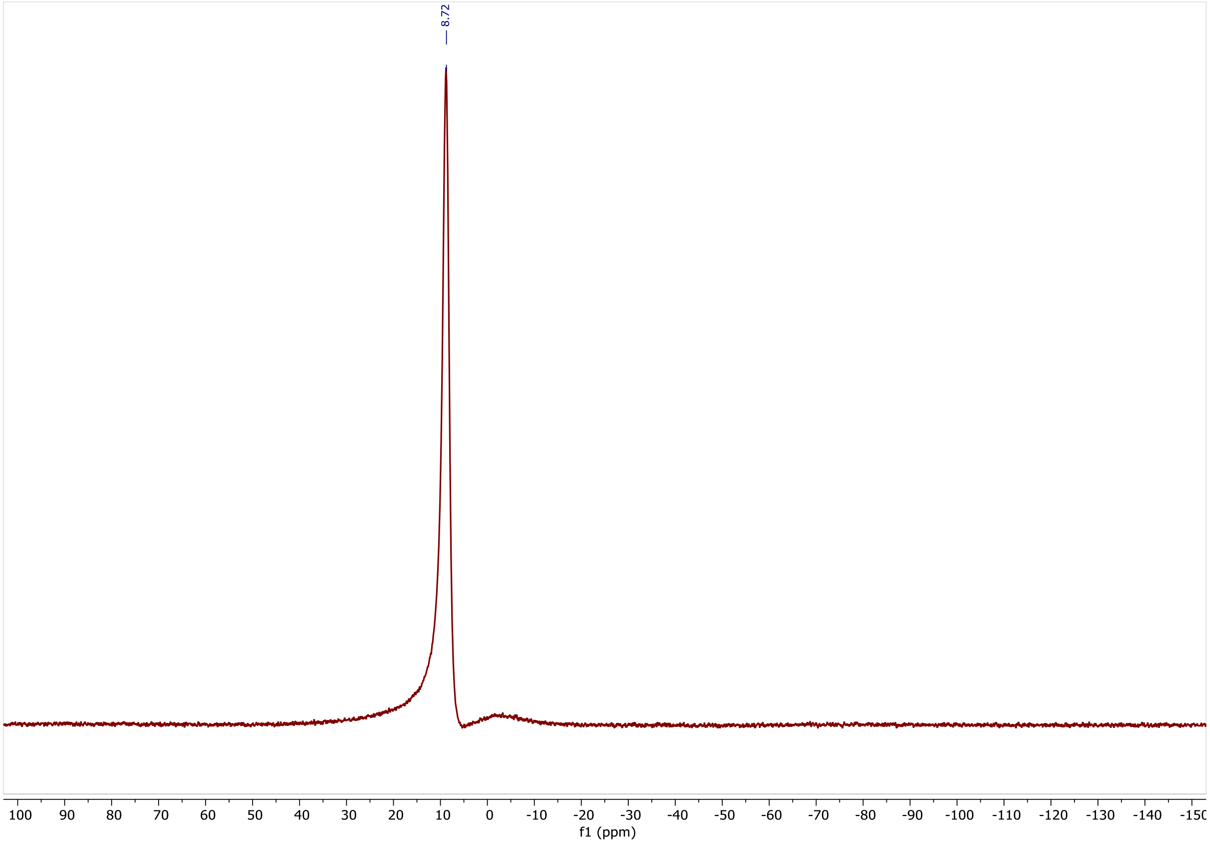
8.72
100
90
80
70
60
50
40
30
20
10
0
-10
-20
-30
-40
-50
-60
-70
-80
-90
-100
-110
-120
-130
-140
-150
f1 (ppm)

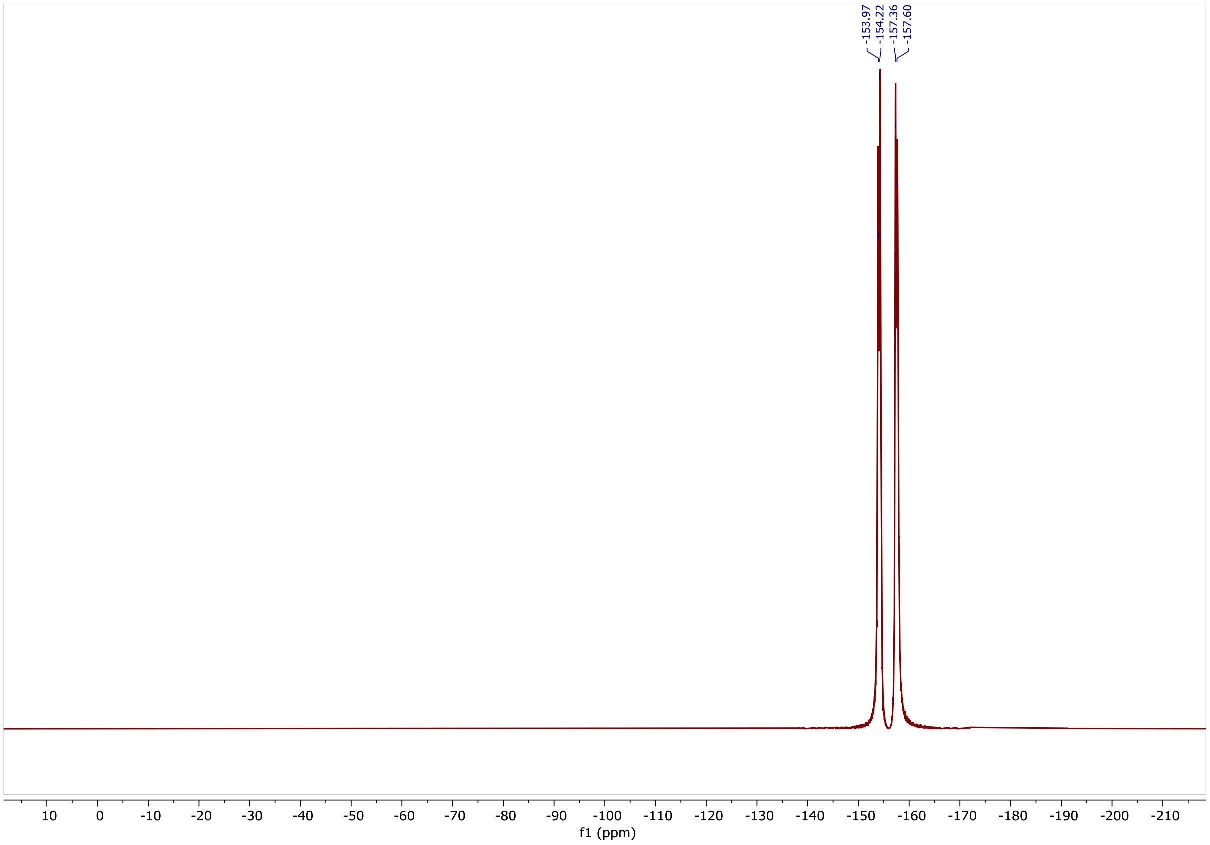
-153.97
-154.22
-157.36
-157.60
10
0
-10
-20
-30
-40
-50
-60
-70
-80
-90
-100
-110
-120
-130
-140
-150
-160
-170
-180
-190
-200
-210
f1 (ppm)

^{1}H, ^{13}C, ^{11}B, and ^{19}F NMR spectra of 9-(2-ethylphenyl)-8,8-difluoro-10,11-dimethyl-8H-pyrido[3',2'3,4][1,2]azaborolo[5,1-a]isoquinolin-7-ium-8-uide (9h)

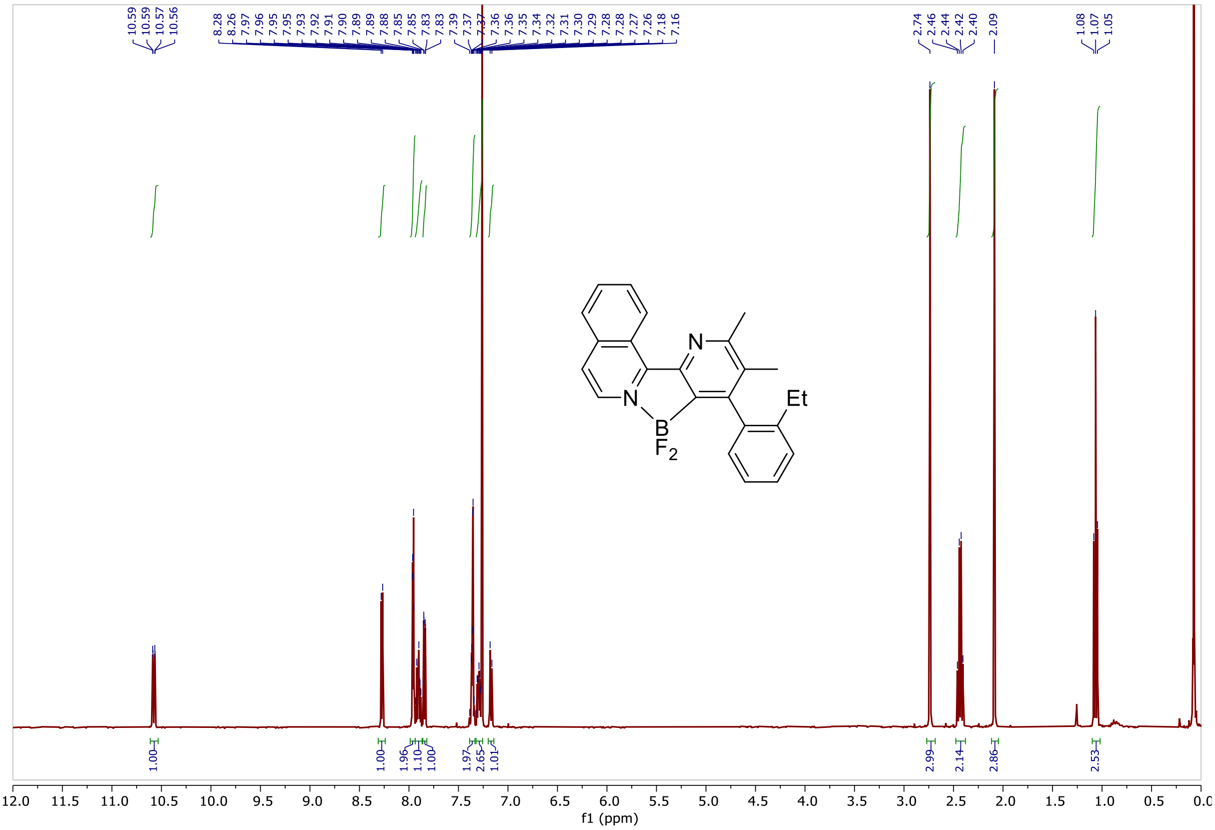

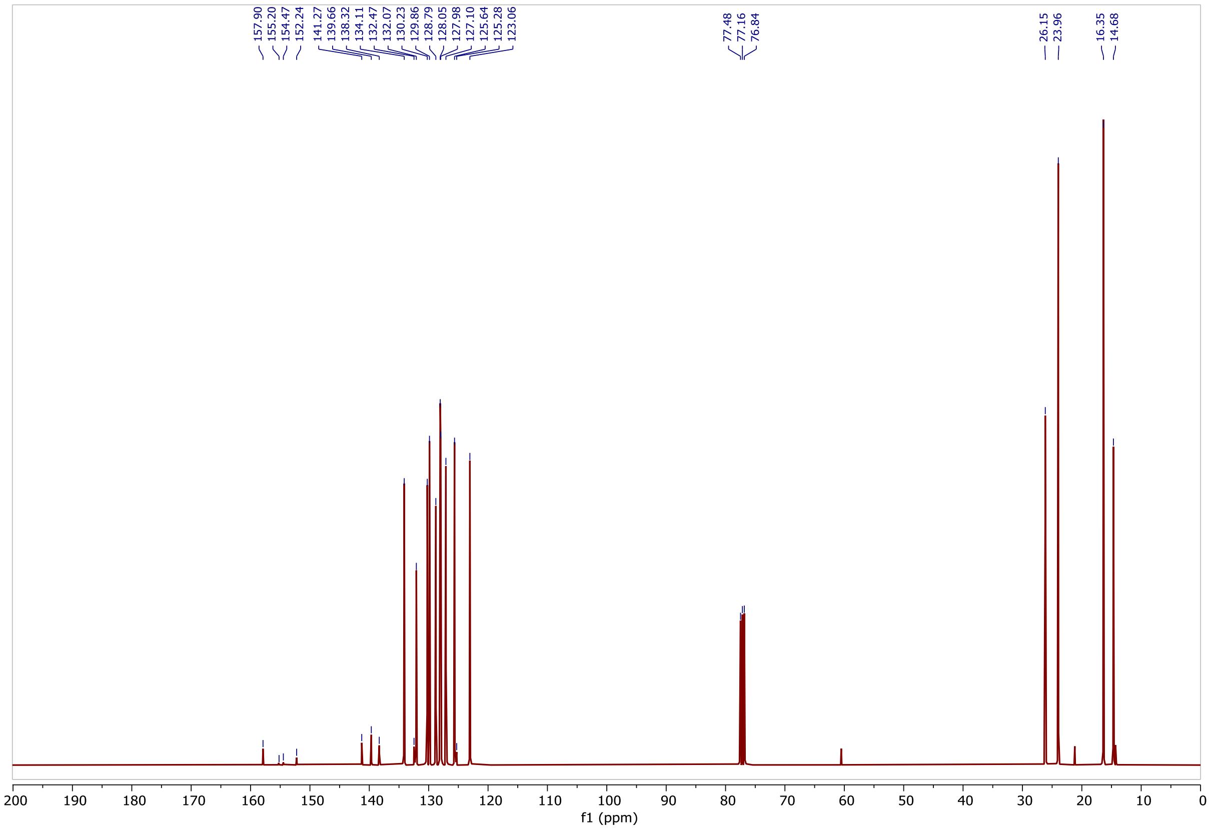

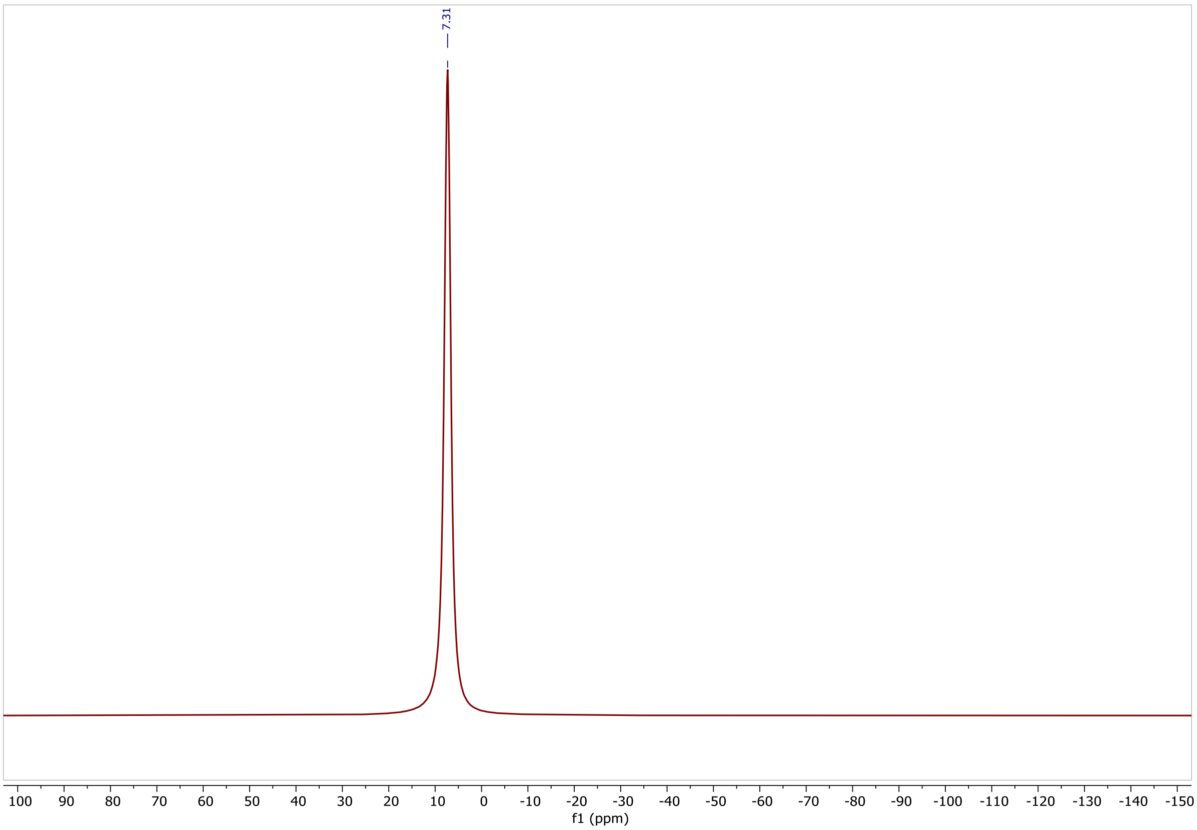

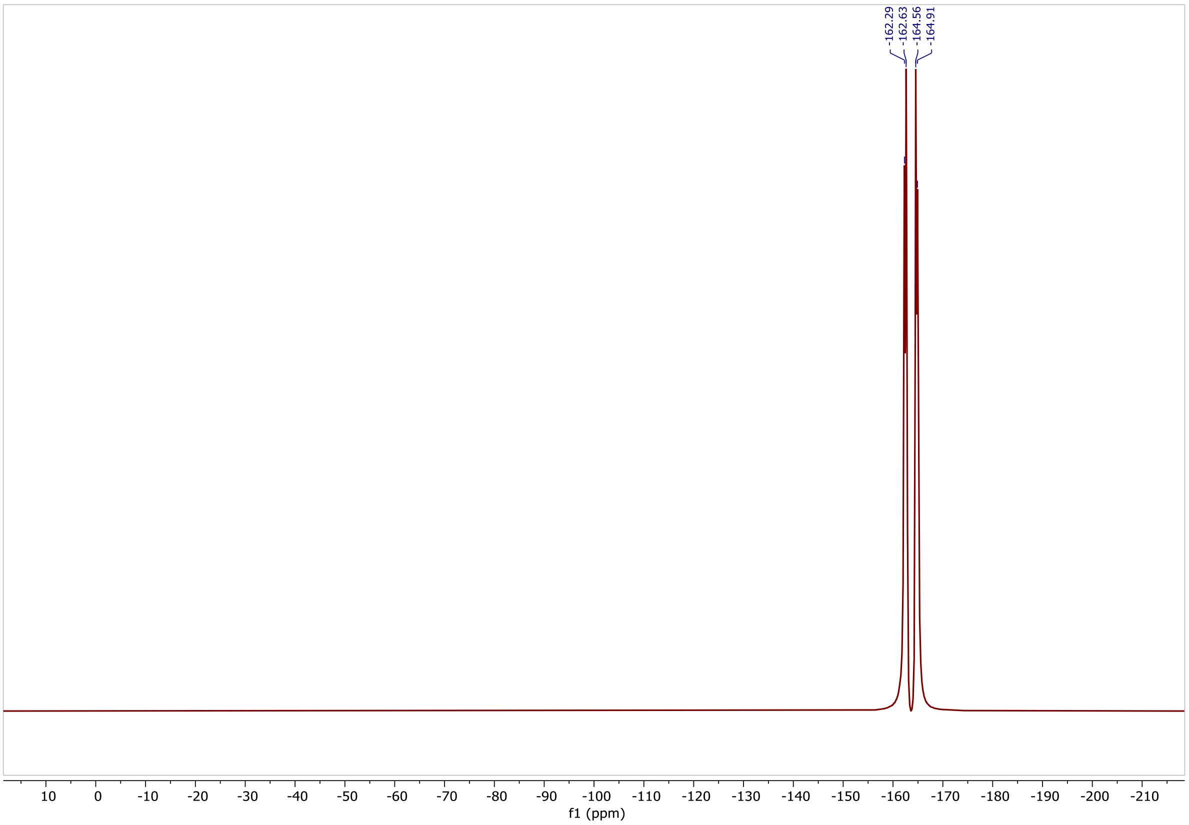

^{1}H, ^{13}C, ^{11}B, and ^{19}F NMR spectra of 8,8-difluoro-9-(2-methoxyphenyl)-10,11-dimethyl-8H-pyrido[3',2':3,4][1,2]azaborolo[5,1-a]isoquinolin-7-ium-8-uide (9i)

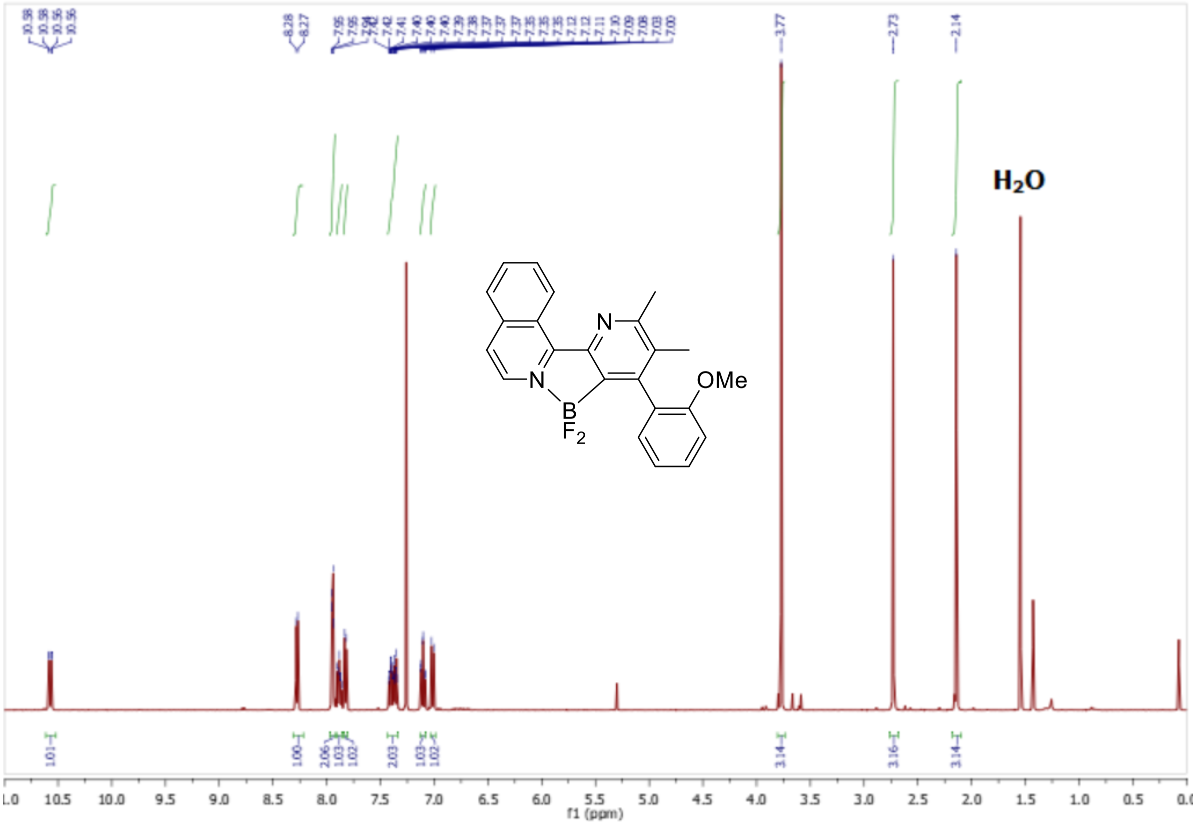
H2O
N
N
B
F2
OMe
f1 (ppm)

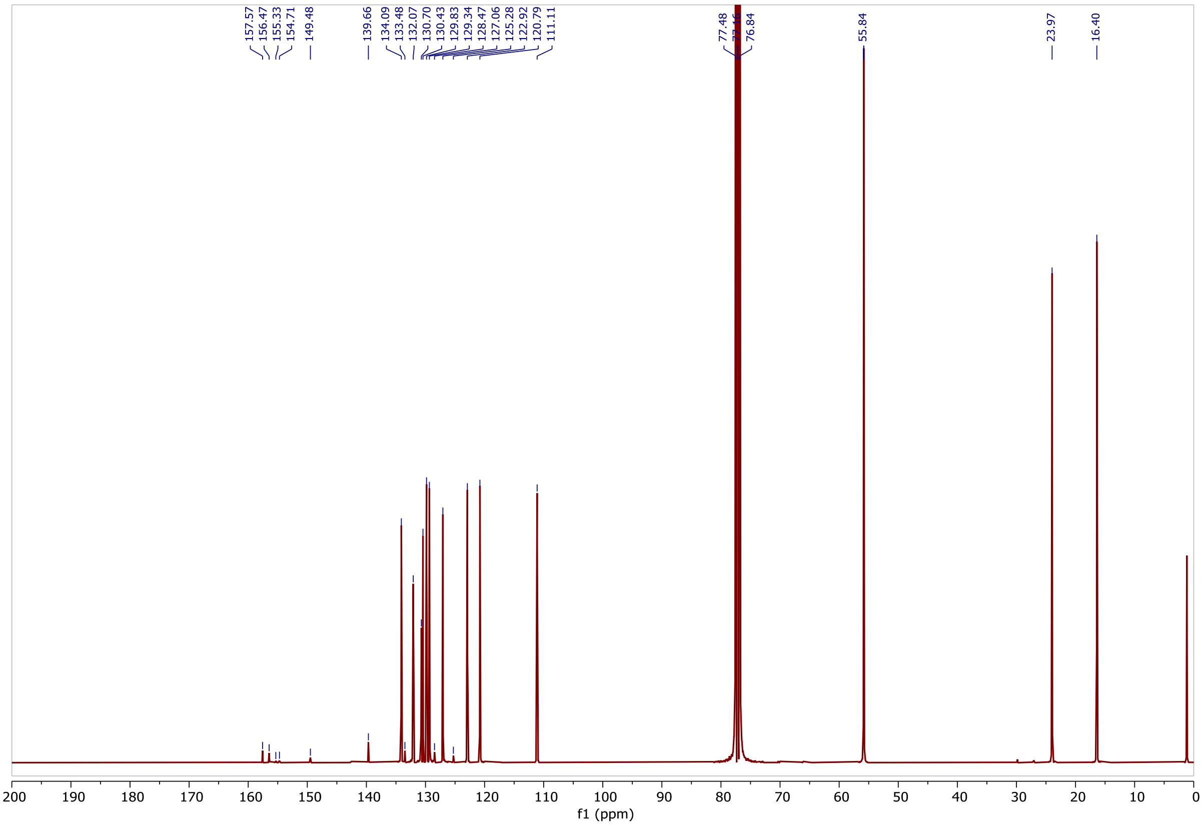
f1 (ppm)

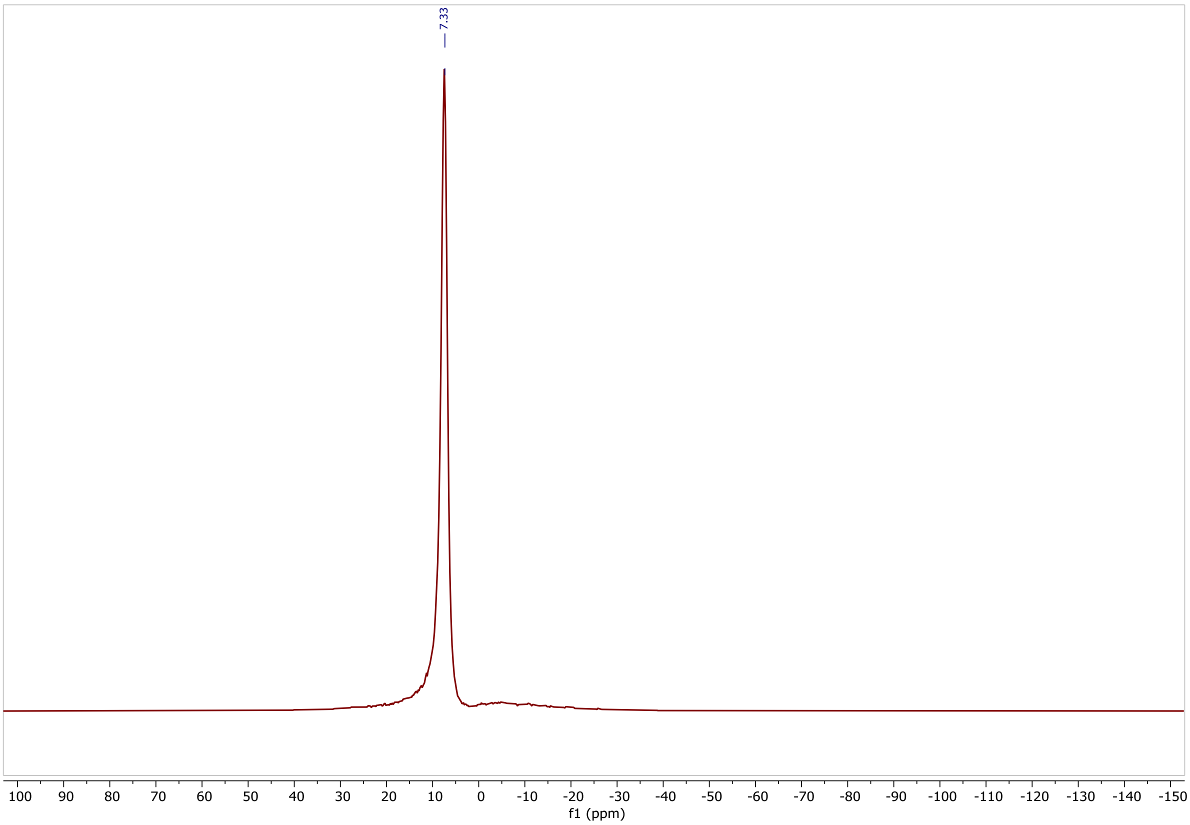
7.33
100 90 80 70 60 50 40 30 20 10 0 -10 -20 -30 -40 -50 -60 -70 -80 -90 -100 -110 -120 -130 -140 -150
f1 (ppm)

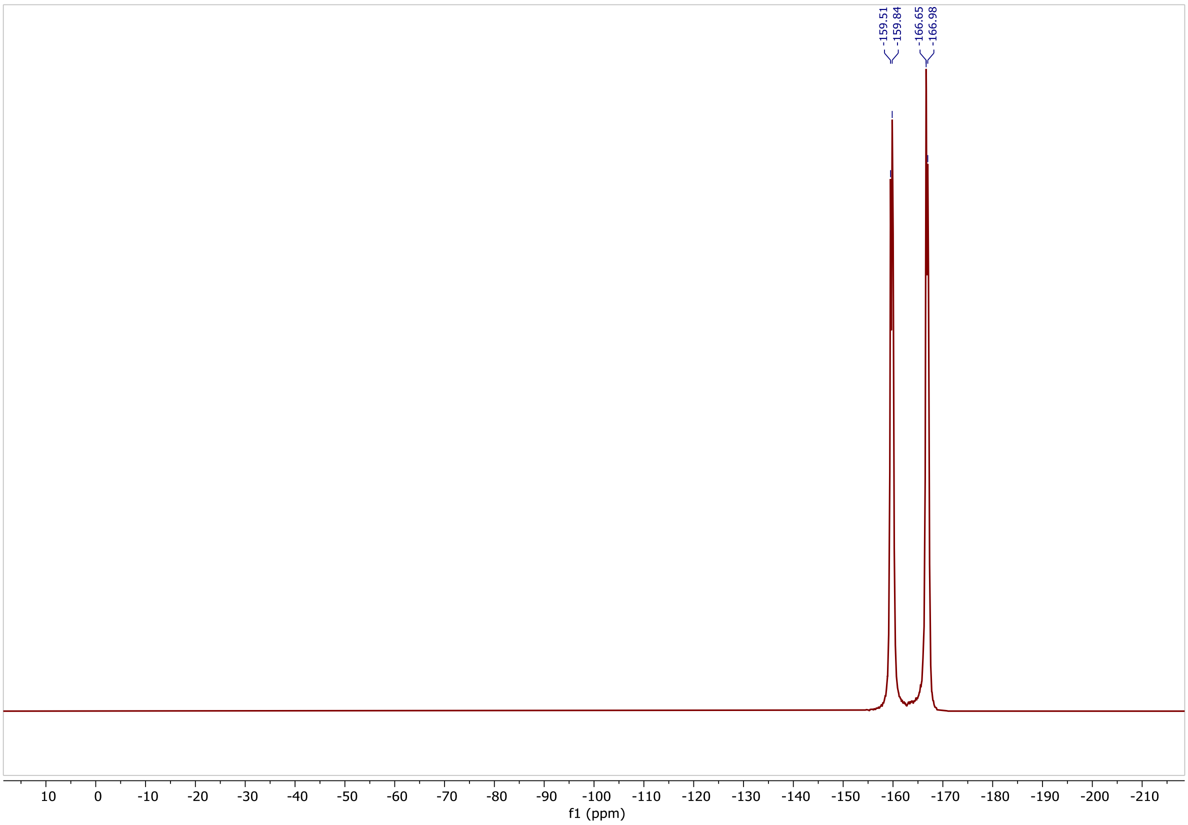
-159.51
-159.84
-166.65
-166.98
10 0 -10 -20 -30 -40 -50 -60 -70 -80 -90 -100 -110 -120 -130 -140 -150 -160 -170 -180 -190 -200 -210
f1 (ppm)

^{1}H, ^{13}C, ^{11}B, and ^{19}F NMR spectra of 9-(2-ethylphenyl)-8,8-difluoro-10,11-diphenyl-8H-pyrido[3',2':3,4][1,2]azaborolo[5,1-a]isoquinolin-7-ium-8-uide (9j)

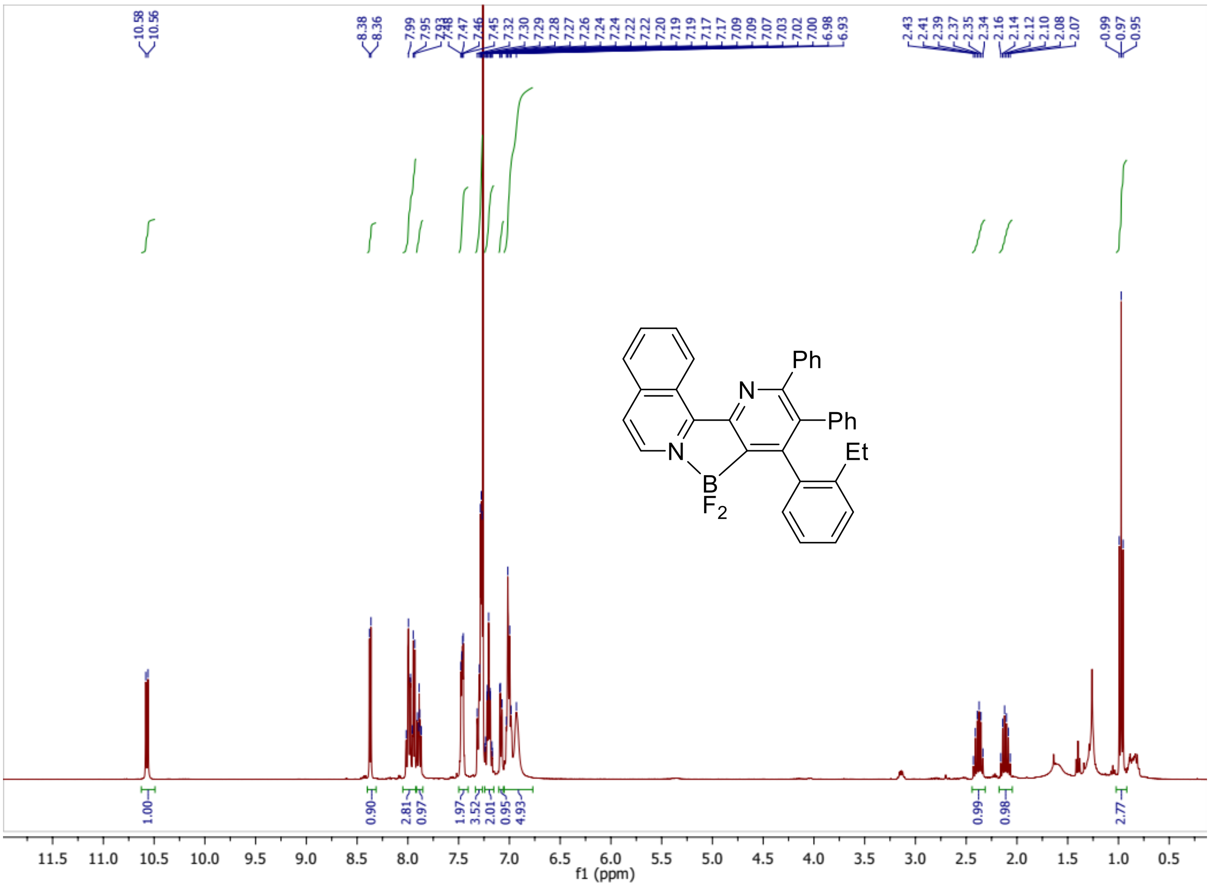

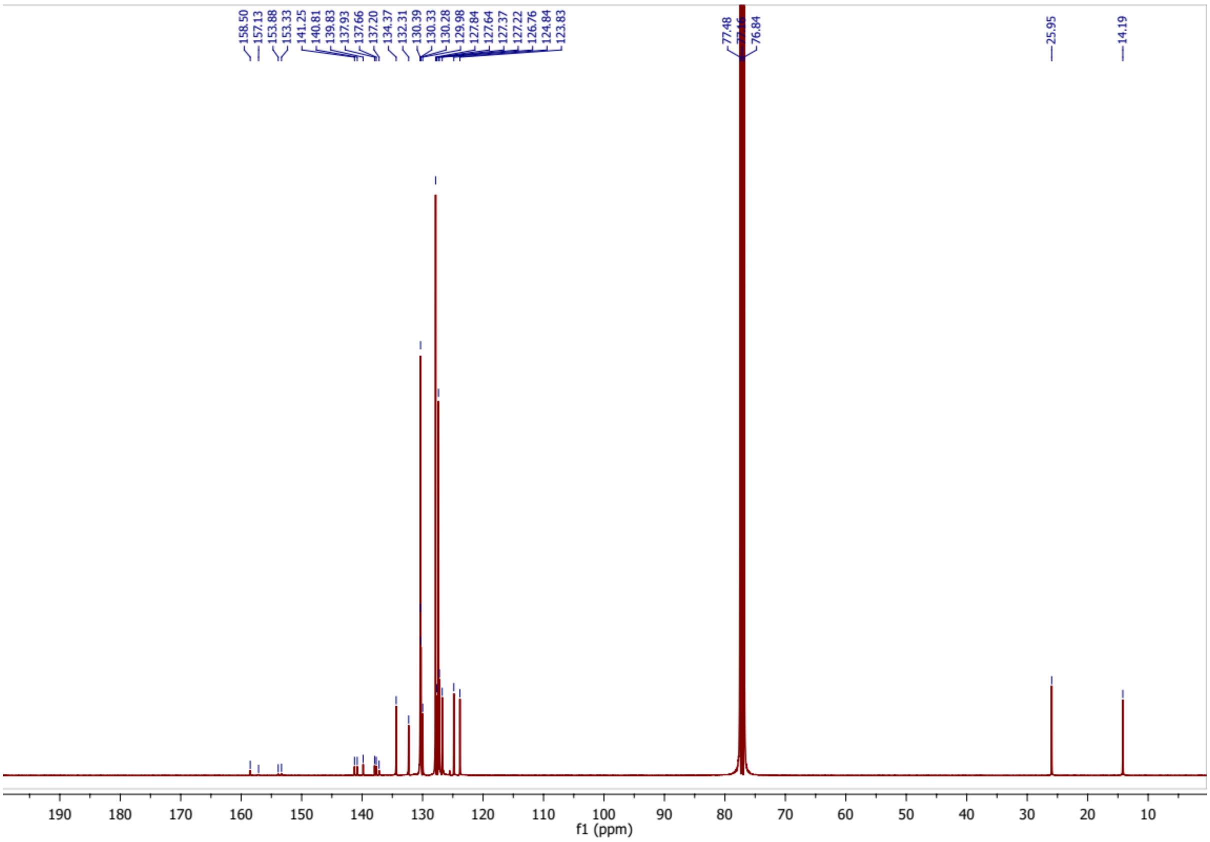

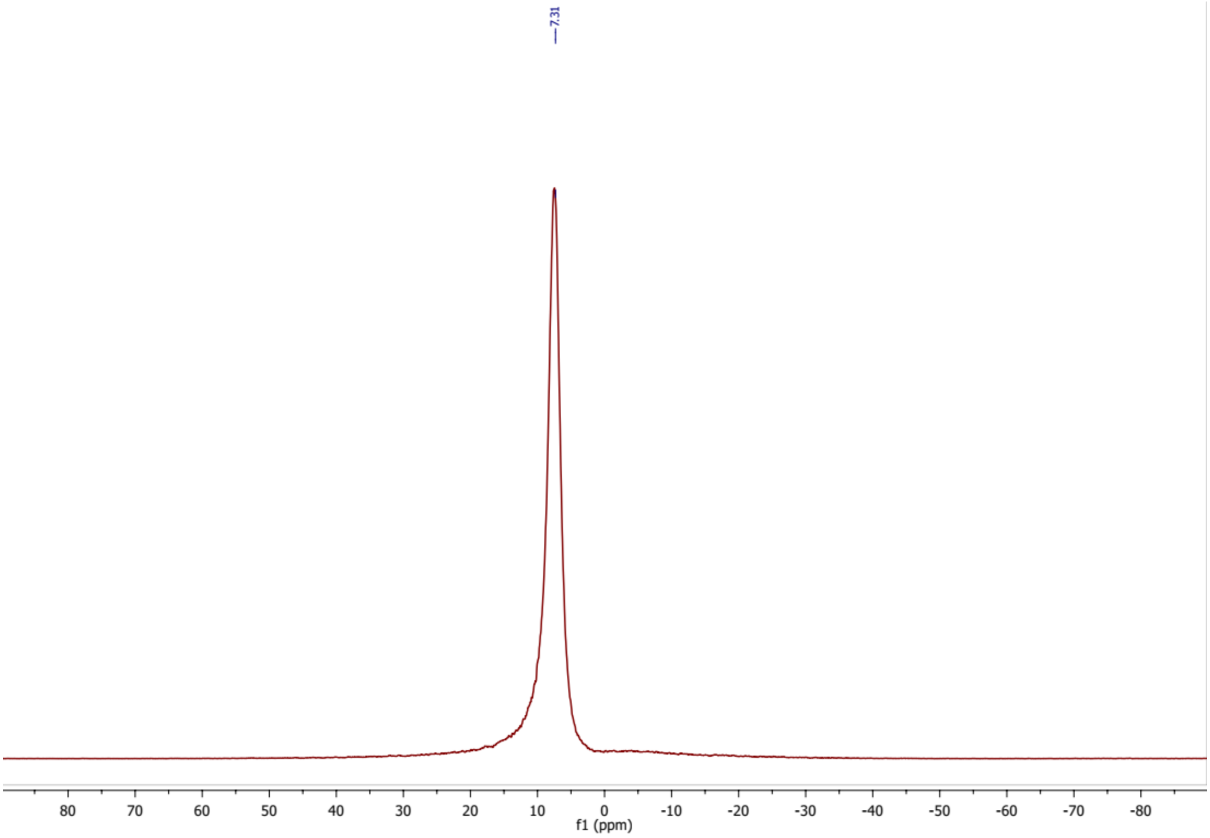
7.31
80
70
60
50
40
30
20
10
0
-10
-20
-30
-40
-50
-60
-70
-80
f1 (ppm)

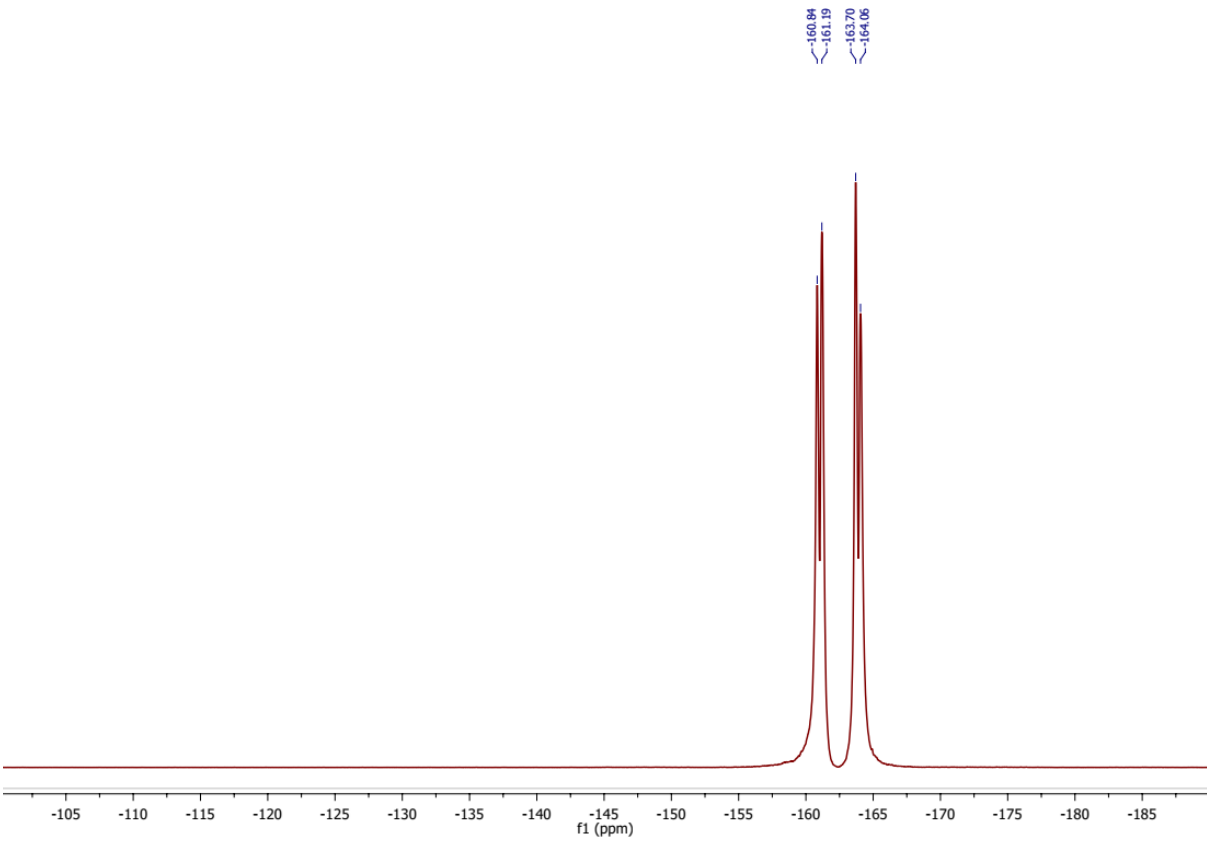
-160.84
-161.19
-163.70
-164.06
-105
-110
-115
-120
-125
-130
-135
-140
-145
-150
-155
-160
-165
-170
-175
-180
-185
f1 (ppm)

^{1}H, ^{13}C, ^{11}B, and ^{19}F NMR spectra of 8,8-difluoro-9-(2-methoxyphenyl)-10,11-diphenyl-8H-pyrido[3',2':3,4][1,2]azaborolo[5,1-a]isoquinolin-7-ium-8-uide (9k)

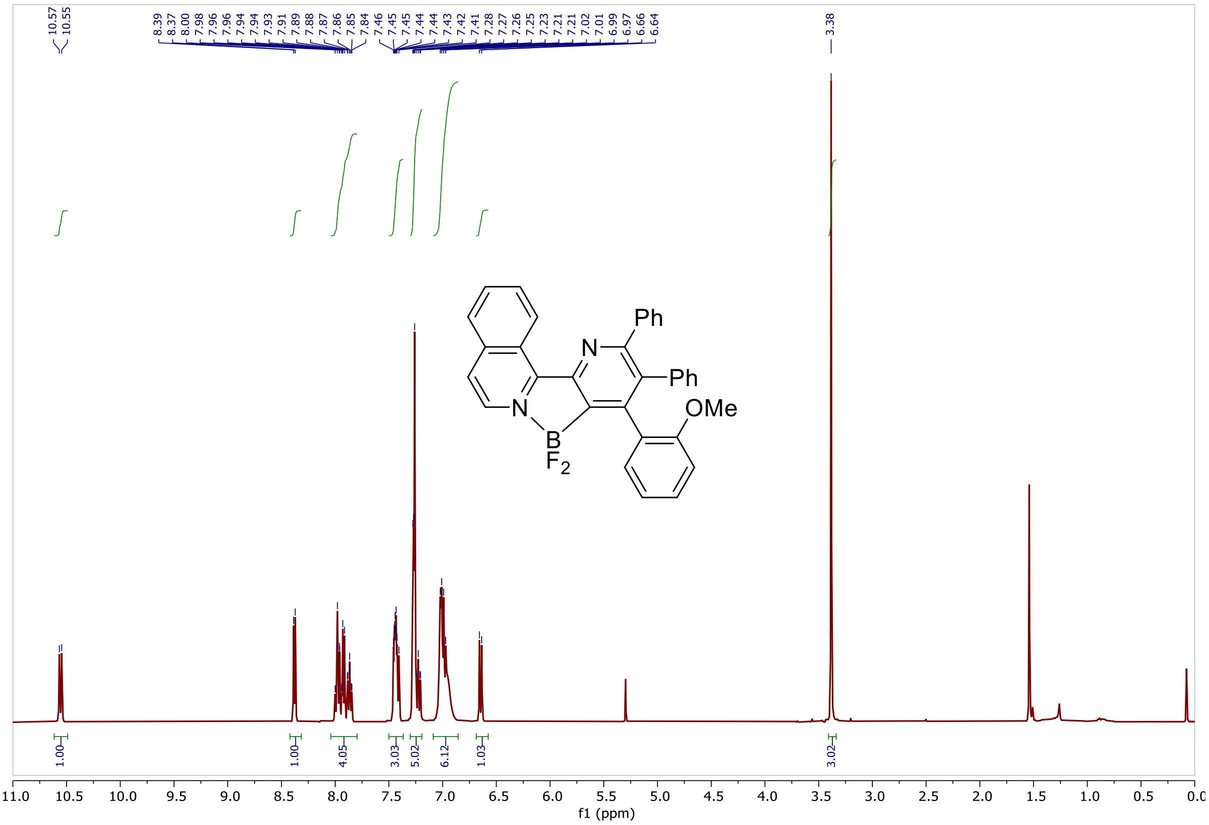

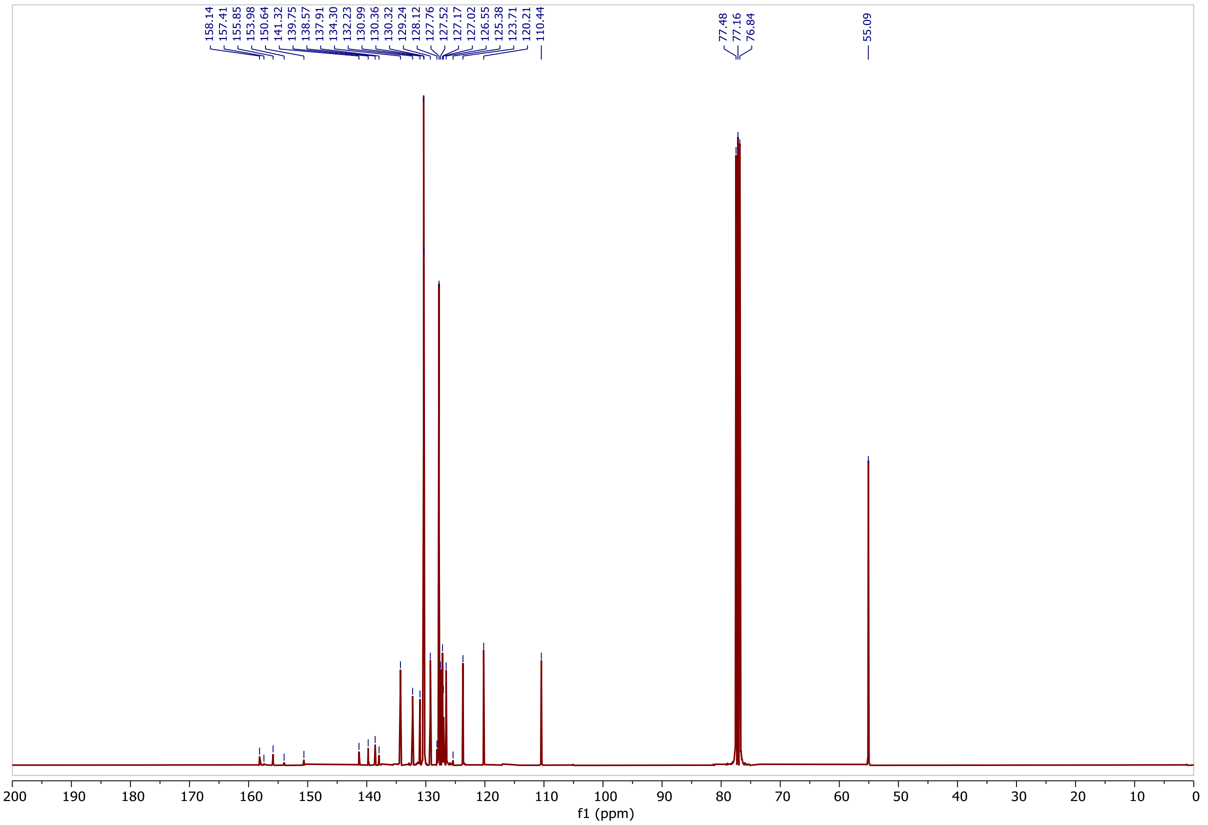

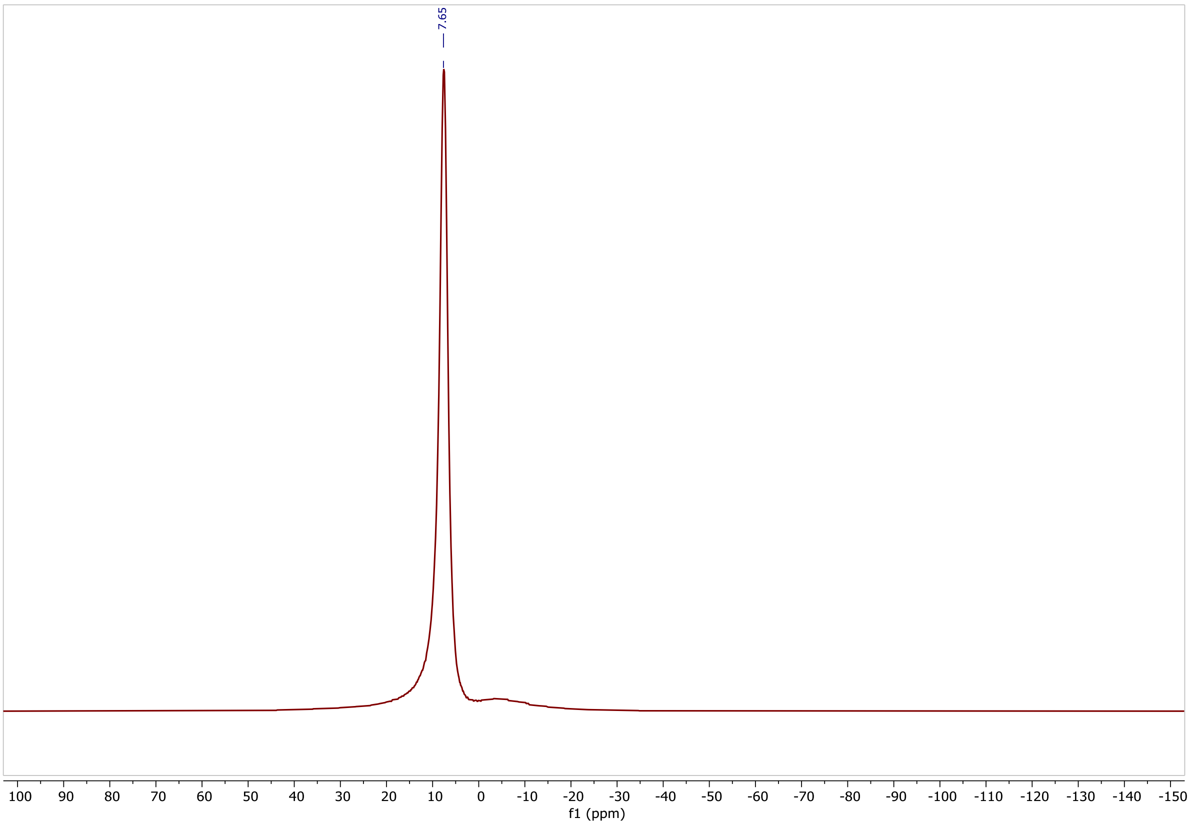
7.65
100
90
80
70
60
50
40
30
20
10
0
-10
-20
-30
-40
-50
-60
-70
-80
-90
-100
-110
-120
-130
-140
-150
f1 (ppm)

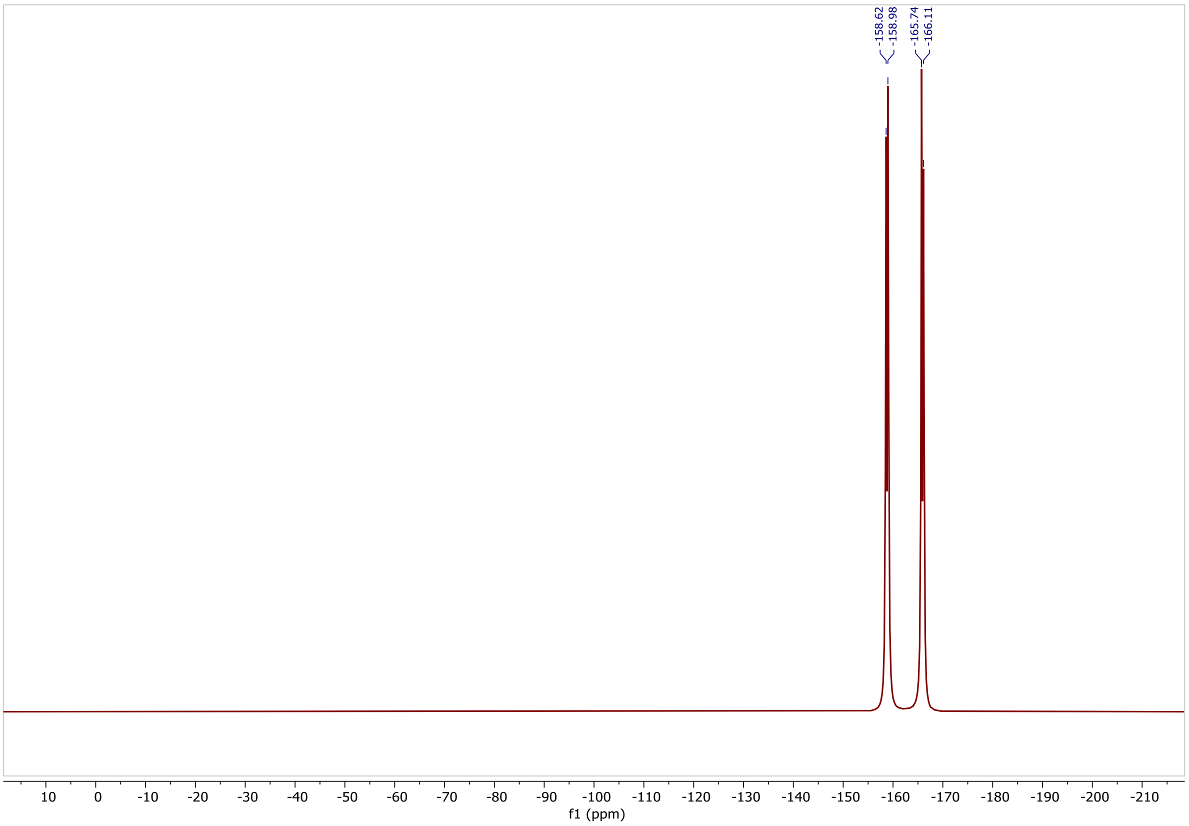
-158.62
-158.98
-165.74
-166.11
10
0
-10
-20
-30
-40
-50
-60
-70
-80
-90
-100
-110
-120
-130
-140
-150
-160
-170
-180
-190
-200
-210
f1 (ppm)

^{1}H, ^{13}C, ^{11}B, and ^{19}F NMR spectra of 5,5-difluoro-4-(2-methoxyphenyl)-2,3-dimethyl-5H-[1,2]azaborolo[1,5-a:4,3-b']dipyridin-6-ium-5-uide (9l)

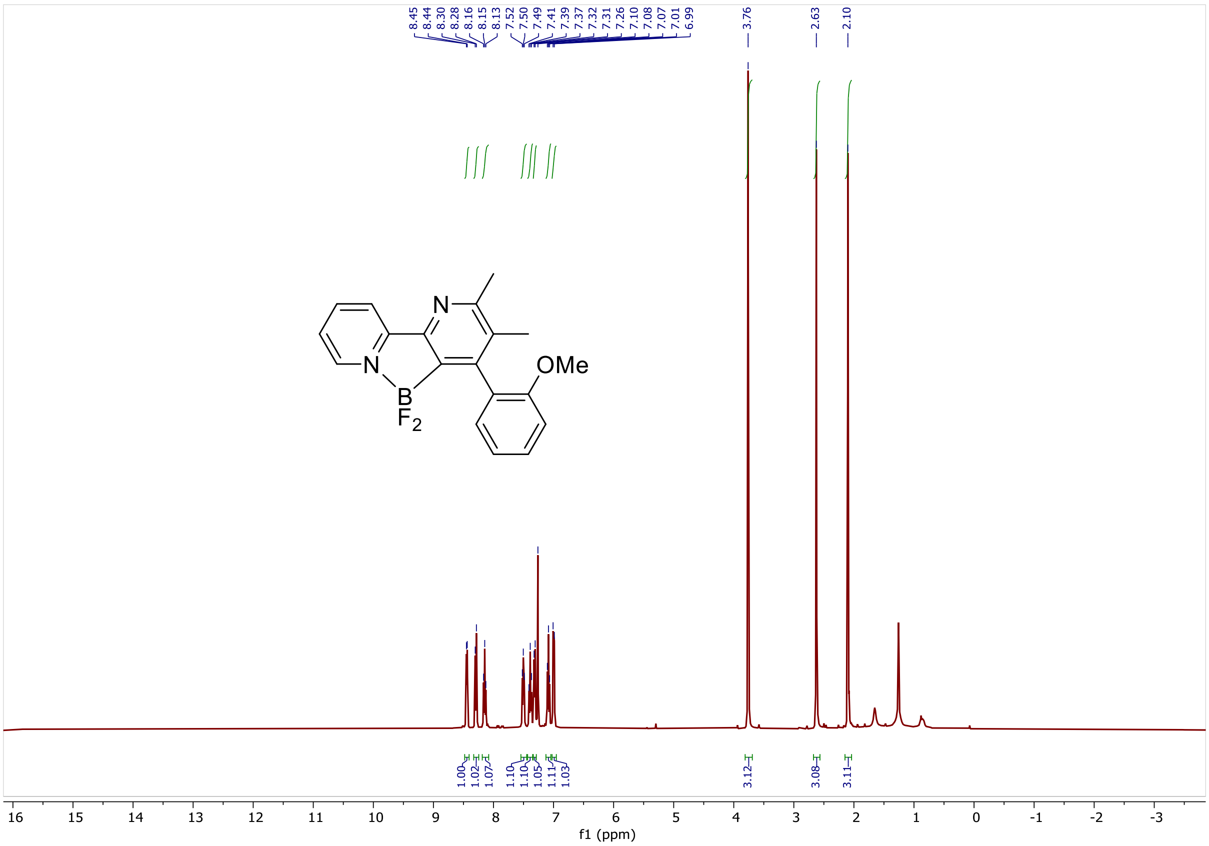

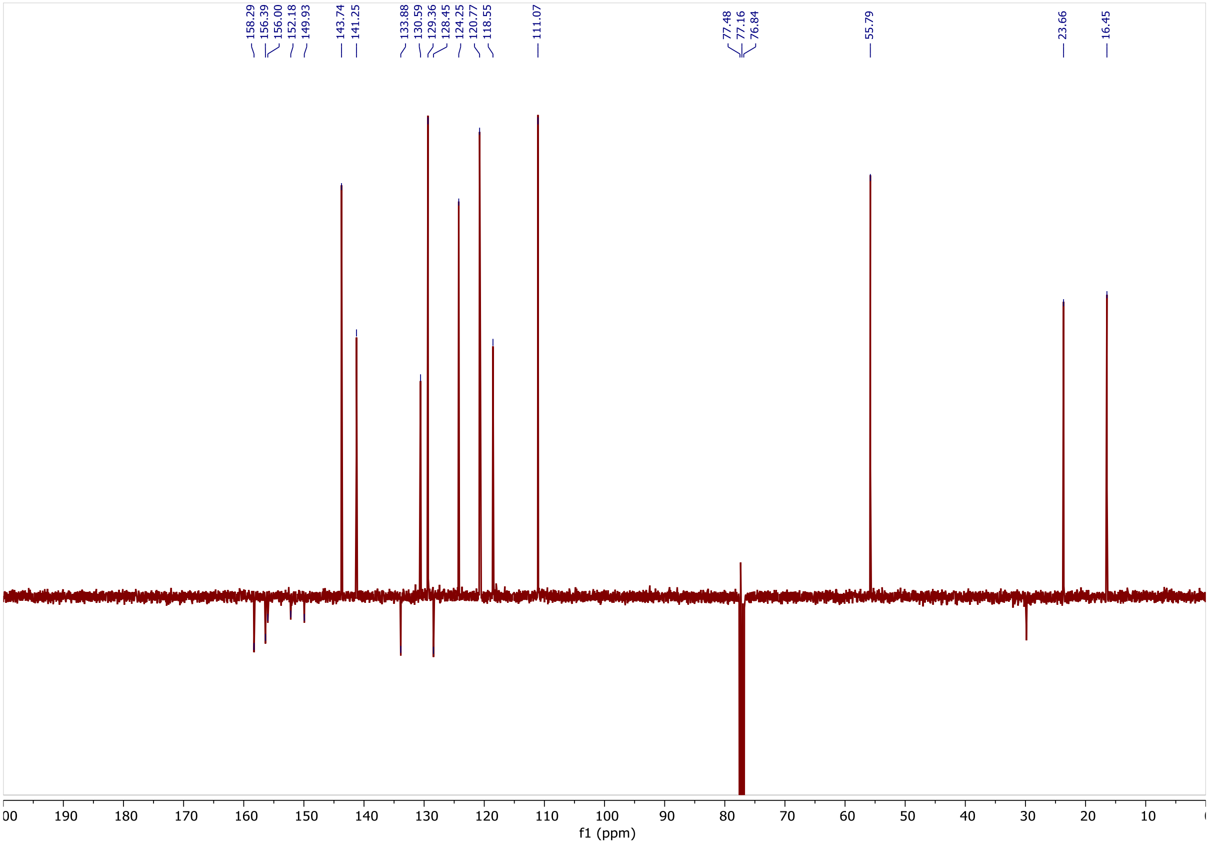

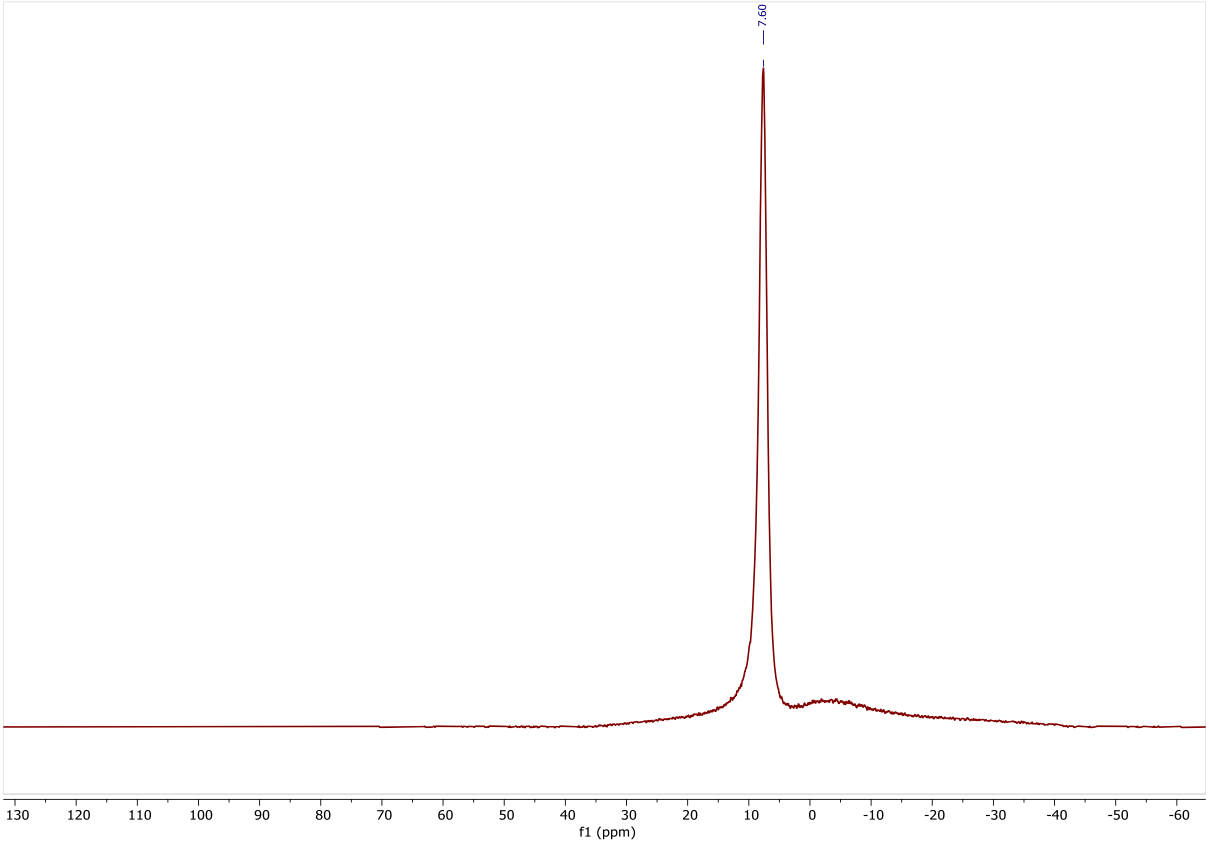
7.60
130
120
110
100
90
80
70
60
50
40
30
20
10
0
-10
-20
-30
-40
-50
-60
f1 (ppm)

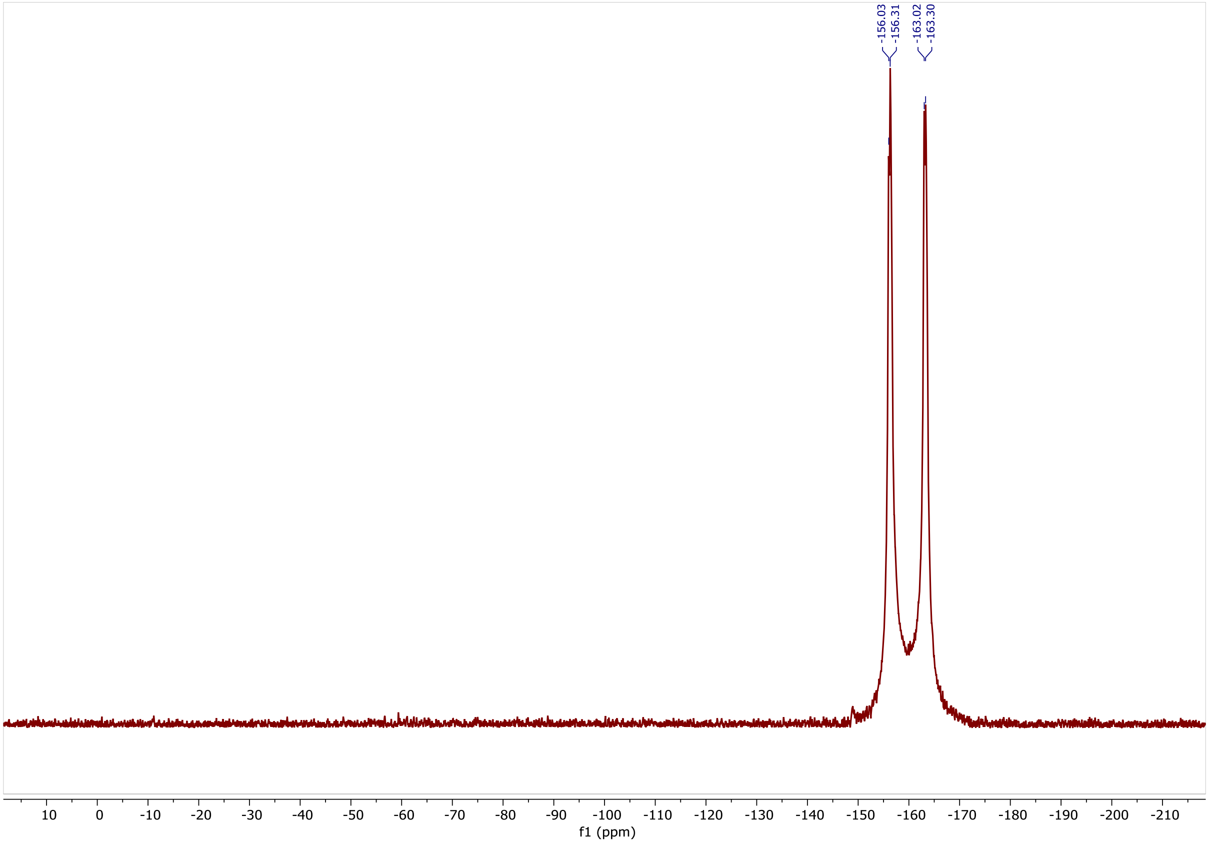
-156.03
-156.31
-163.02
-163.30
10
0
-10
-20
-30
-40
-50
-60
-70
-80
-90
-100
-110
-120
-130
-140
-150
-160
-170
-180
-190
-200
-210
f1 (ppm)

^{1}H, ^{13}C, and ^{11}B NMR spectra of (4S,10'R,11bR)-10'-(2-ethylphenyl)-8',9'-dimethylspiro[dinaphtho[2,1-d:1',2'-f][1,3,2]dioxaborepine-4,11'-pyrido[3',2'3,4][1,2]azaborolo[1,5-a]quinolin]-12'-ium-26-uide (10a)

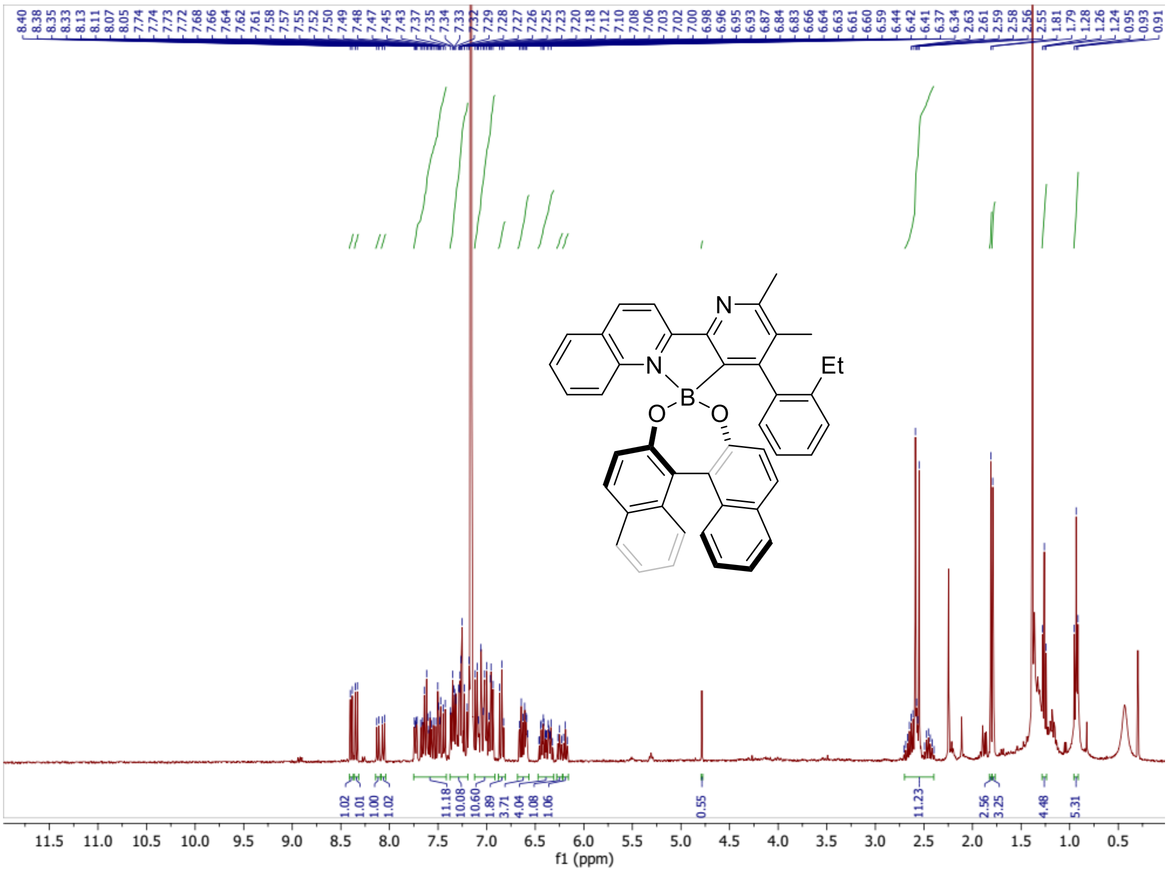

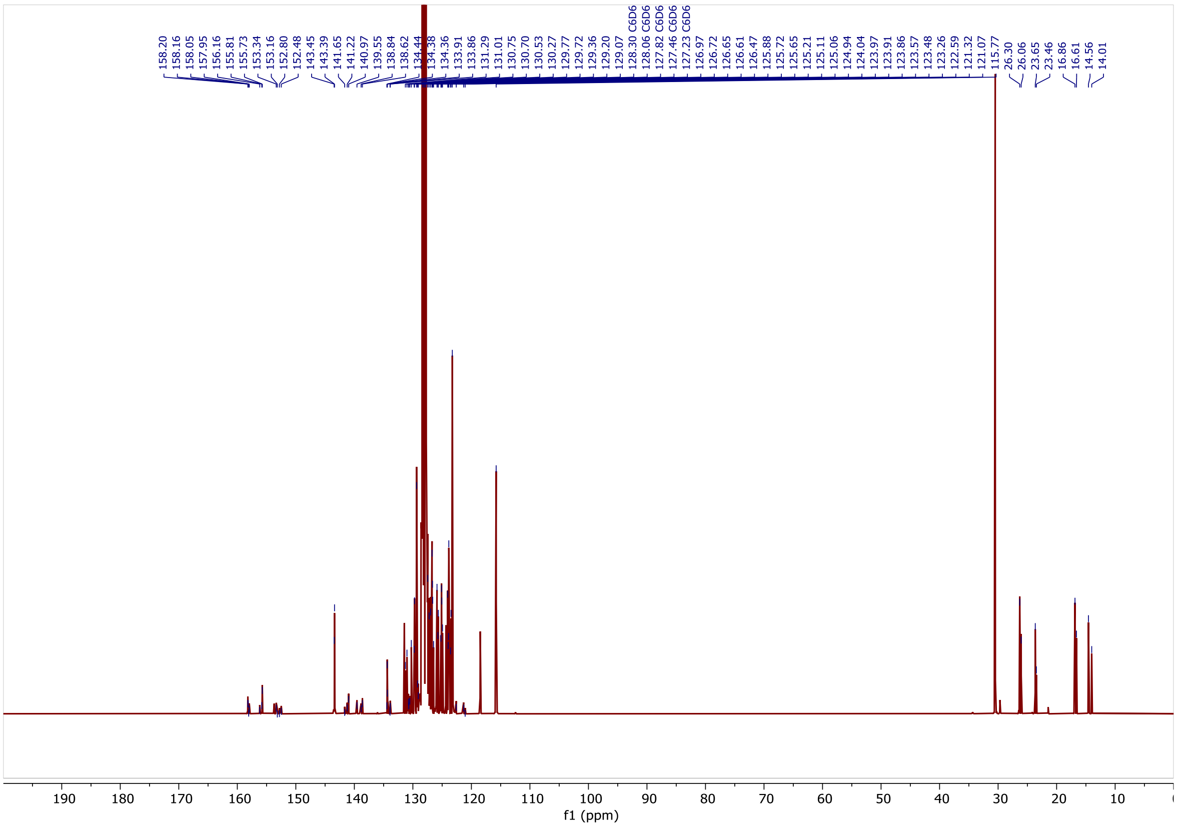

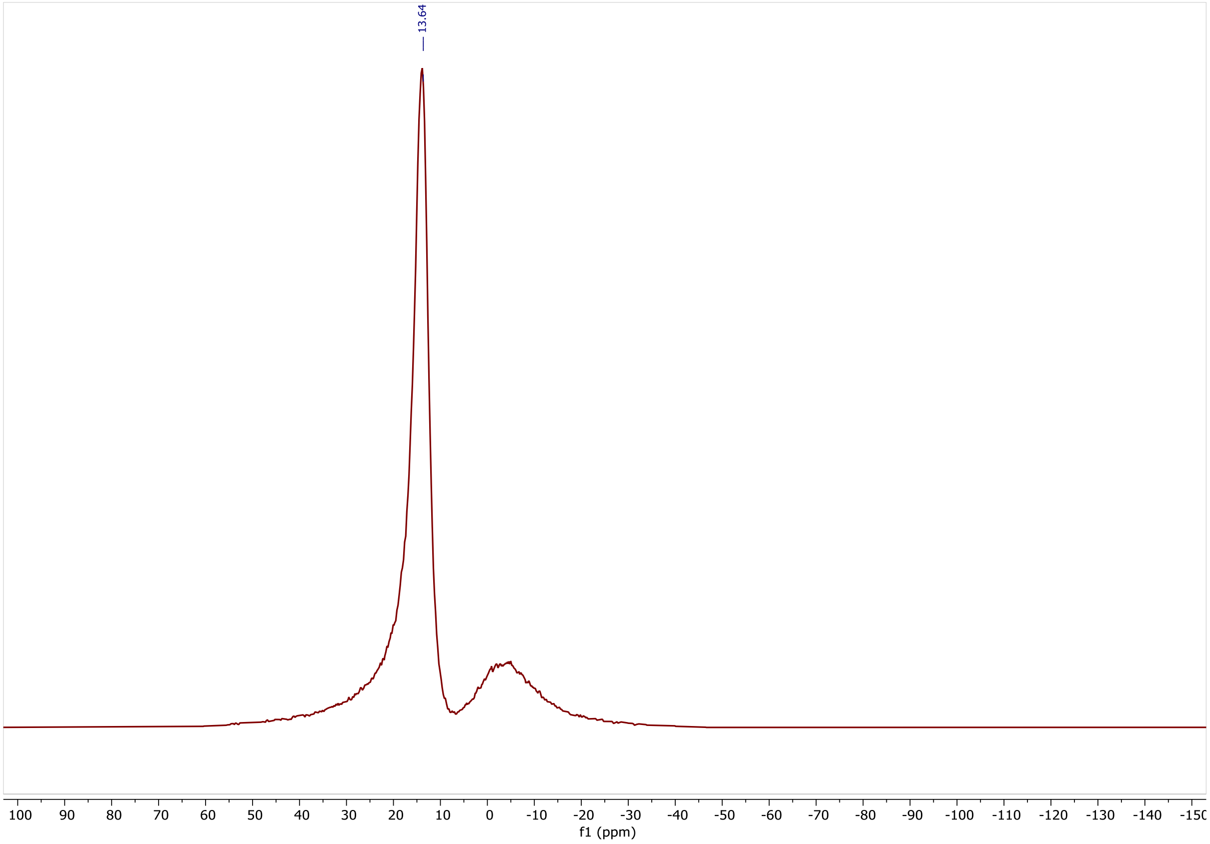
13.64
100
90
80
70
60
50
40
30
20
10
0
-10
-20
-30
-40
-50
-60
-70
-80
-90
-100
-110
-120
-130
-140
-150
f1 (ppm)

^{1}H, ^{13}C, and ^{11}B NMR spectra of (4S,10'R,11bR)-10'-(2-methoxyphenyl)-8',9'-dimethylspiro[dinaphtho[2,1-d:1',2'-f][1,3,2]dioxaborepine-4,11'-pyrido[3',2':3,4][1,2]azaborolo[1,5-a]quinolin]-12'-ium-26-uide (10b)

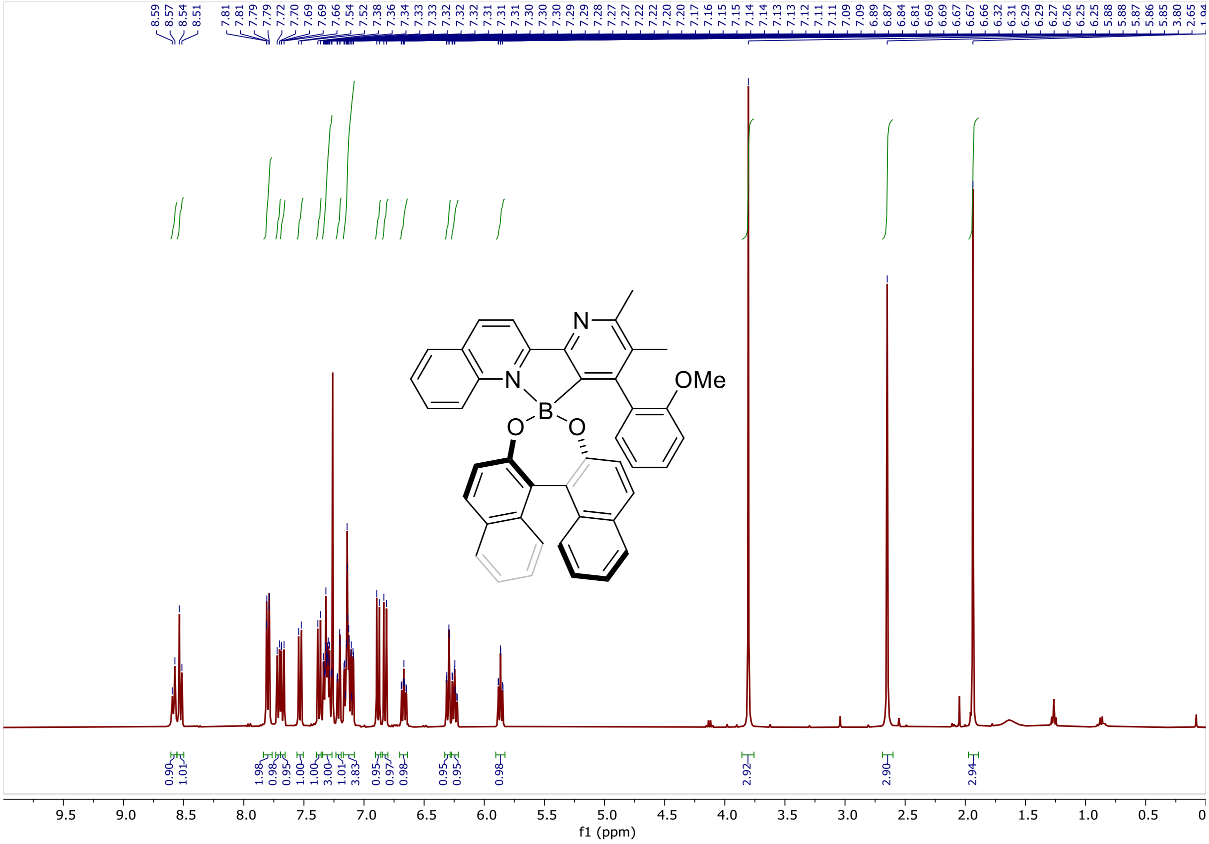

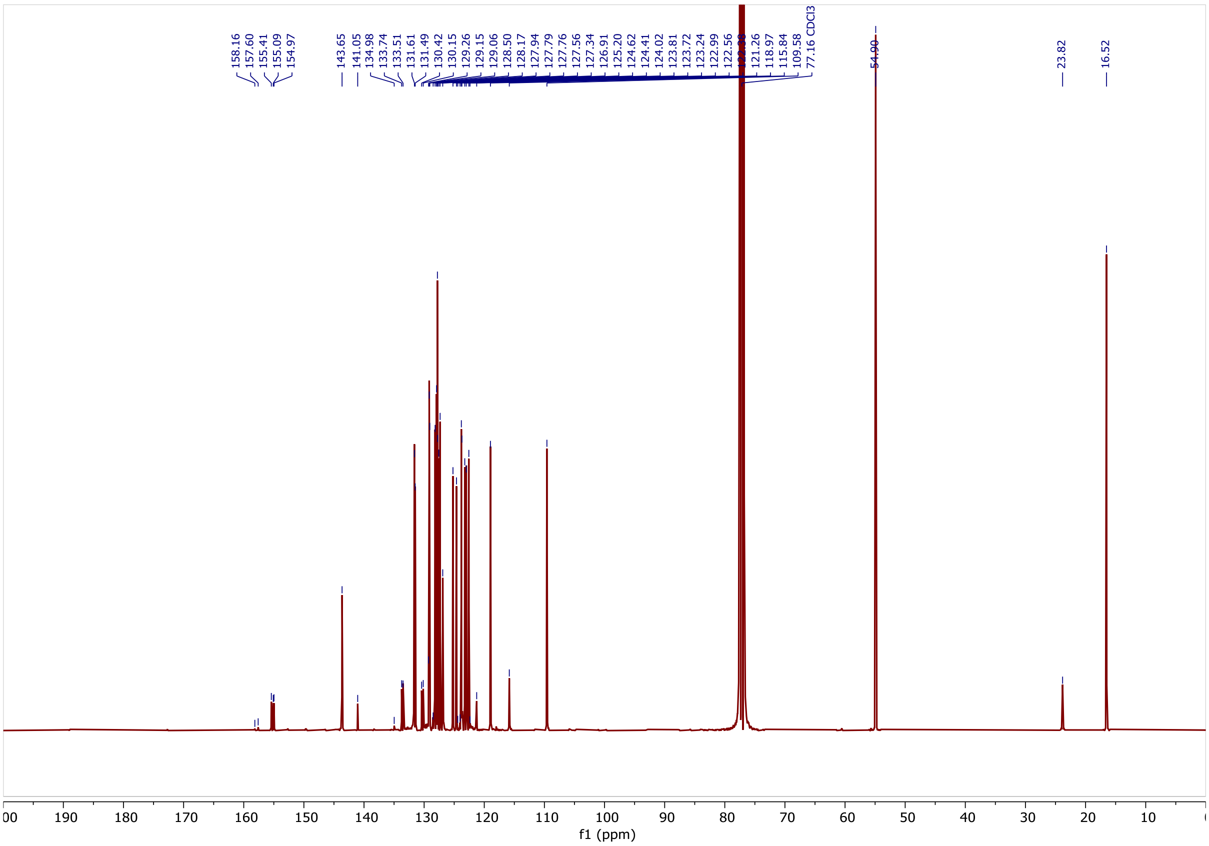

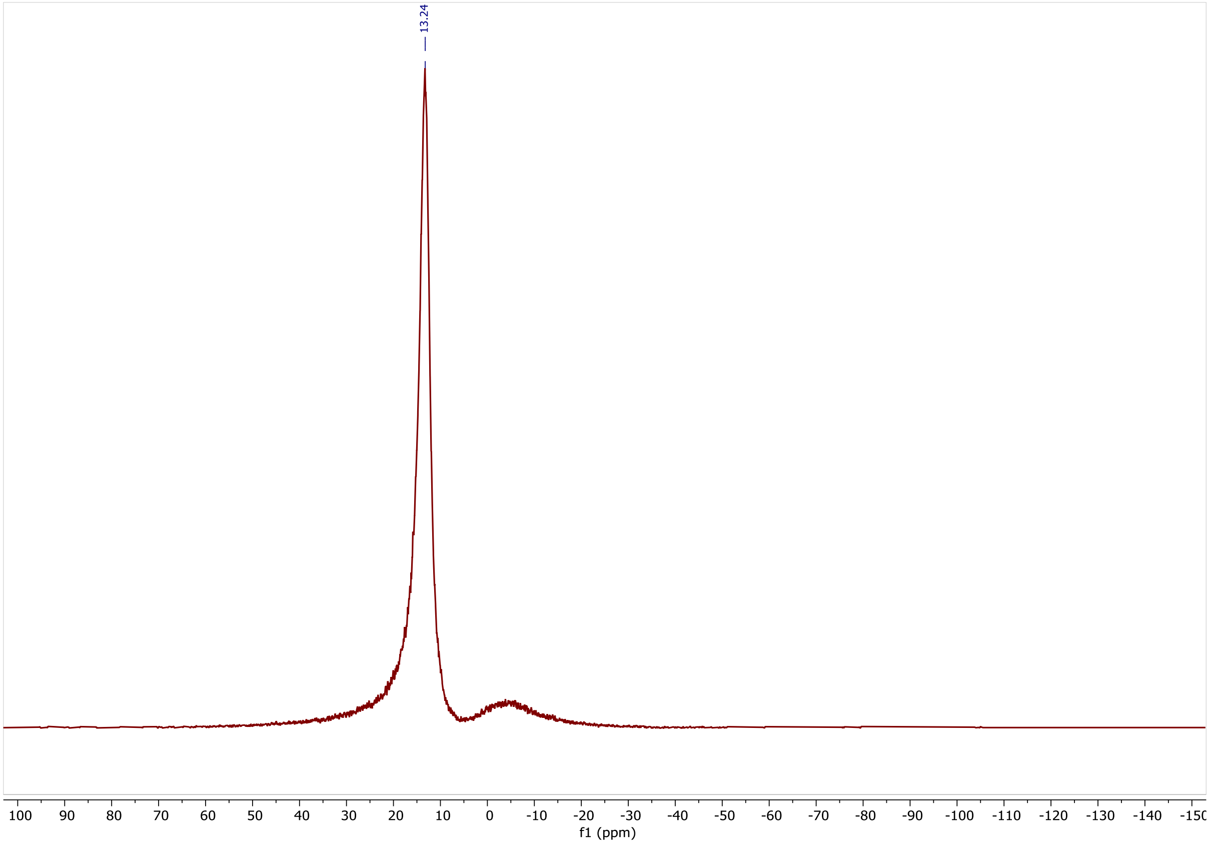
13.24
100
90
80
70
60
50
40
30
20
10
0
-10
-20
-30
-40
-50
-60
-70
-80
-90
-100
-110
-120
-130
-140
-150
f1 (ppm)

^{1}H, ^{13}C, and ^{11}B NMR spectra of2-(3-((R)-dinaphtho[2,1-d:1',2'-f][1,3,2]dioxaborepin-4-yl)-4-(2-ethylphenyl)-5,6-diphenylpyridin-2-yl)quinoline (10c)

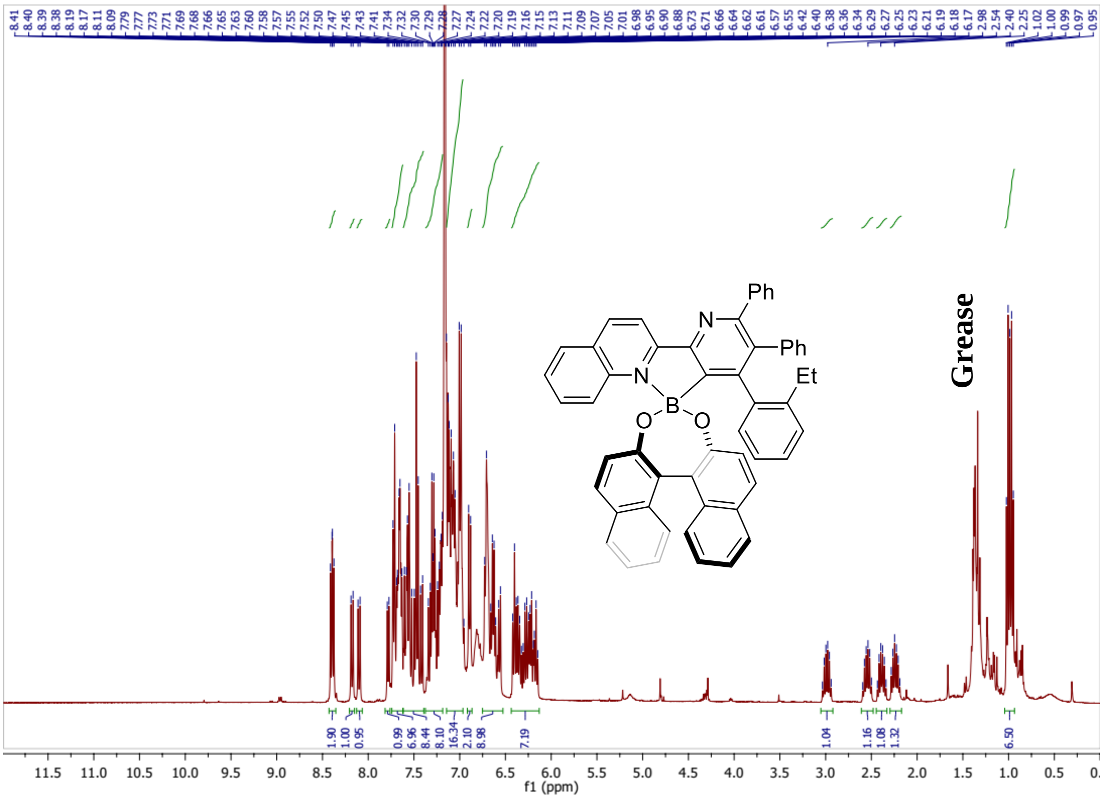

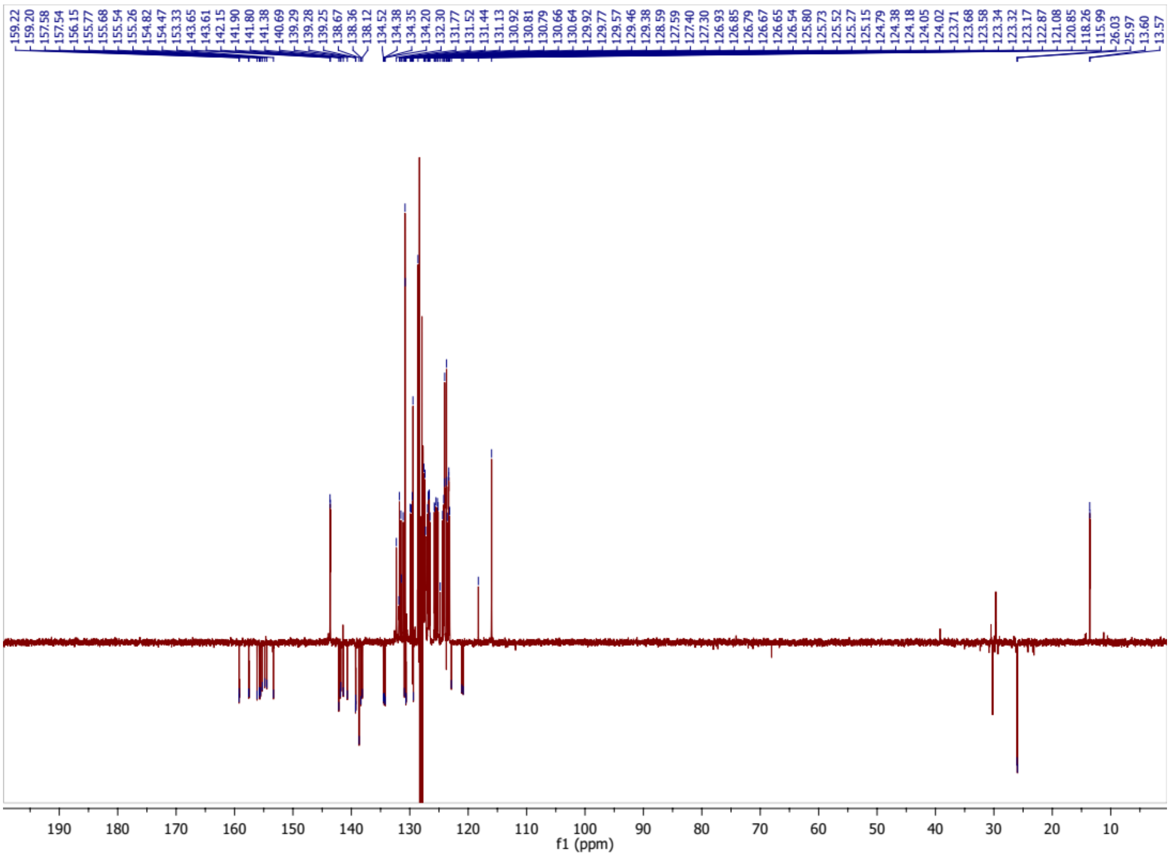

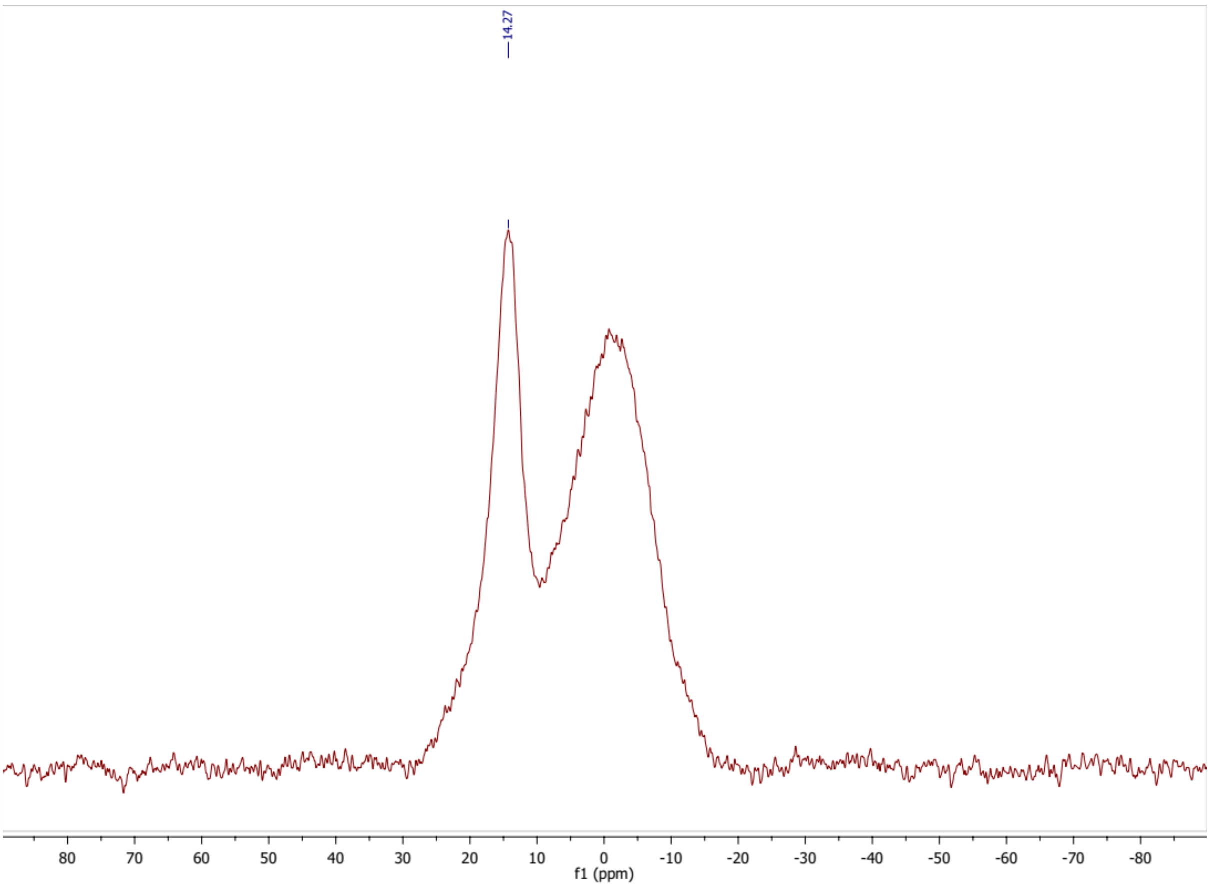
14.27
80
70
60
50
40
30
20
10
0
-10
-20
-30
-40
-50
-60
-70
-80
f1 (ppm)

^{1}H, ^{13}C, and ^{11}B NMR spectra of (4S,10'R,11bR)-10'-(2-methoxyphenyl)-8',9'-diphenylspiro[dinaphtho[2,1-d:1',2'-f][1,3,2]dioxaborepine-4,11'-pyrido[3',2':3,4][1,2]azaborolo[1,5-a]quinolin]-12'-ium-26-uide (10d)

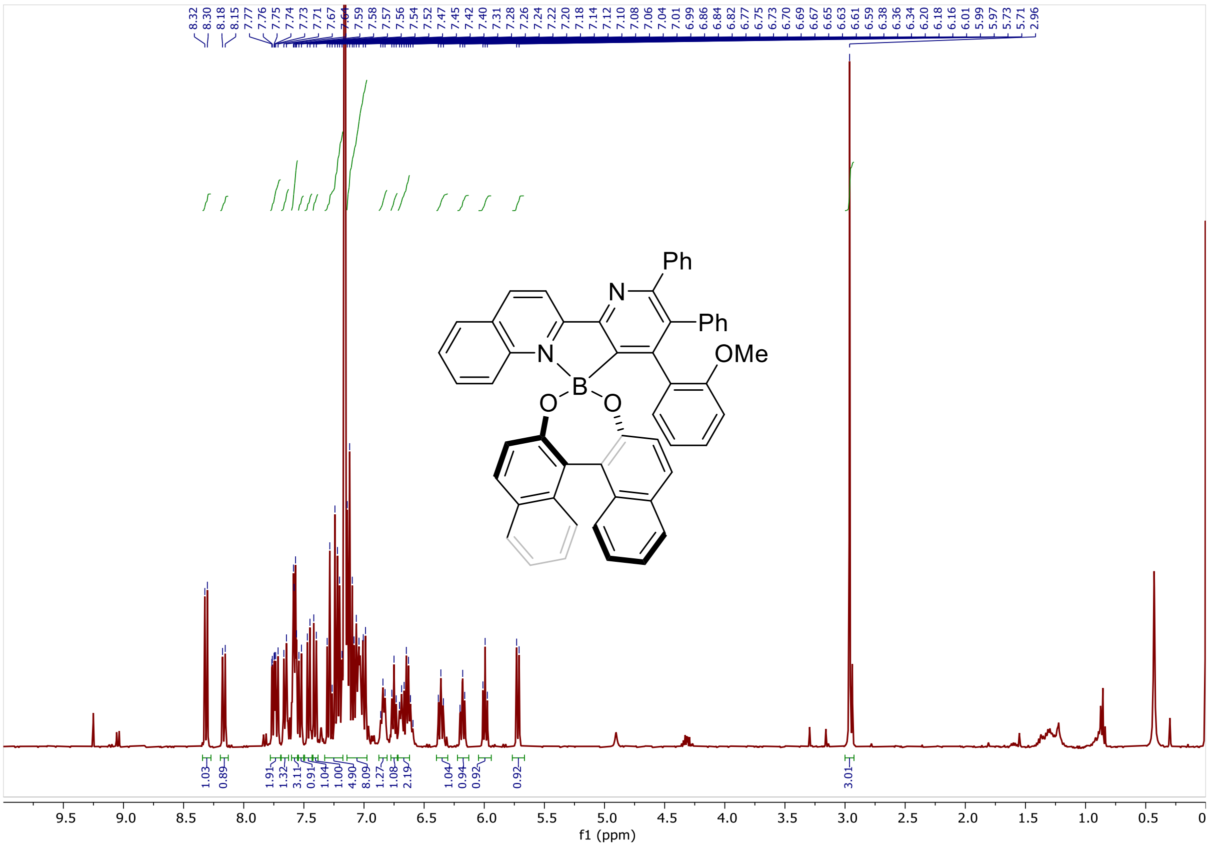

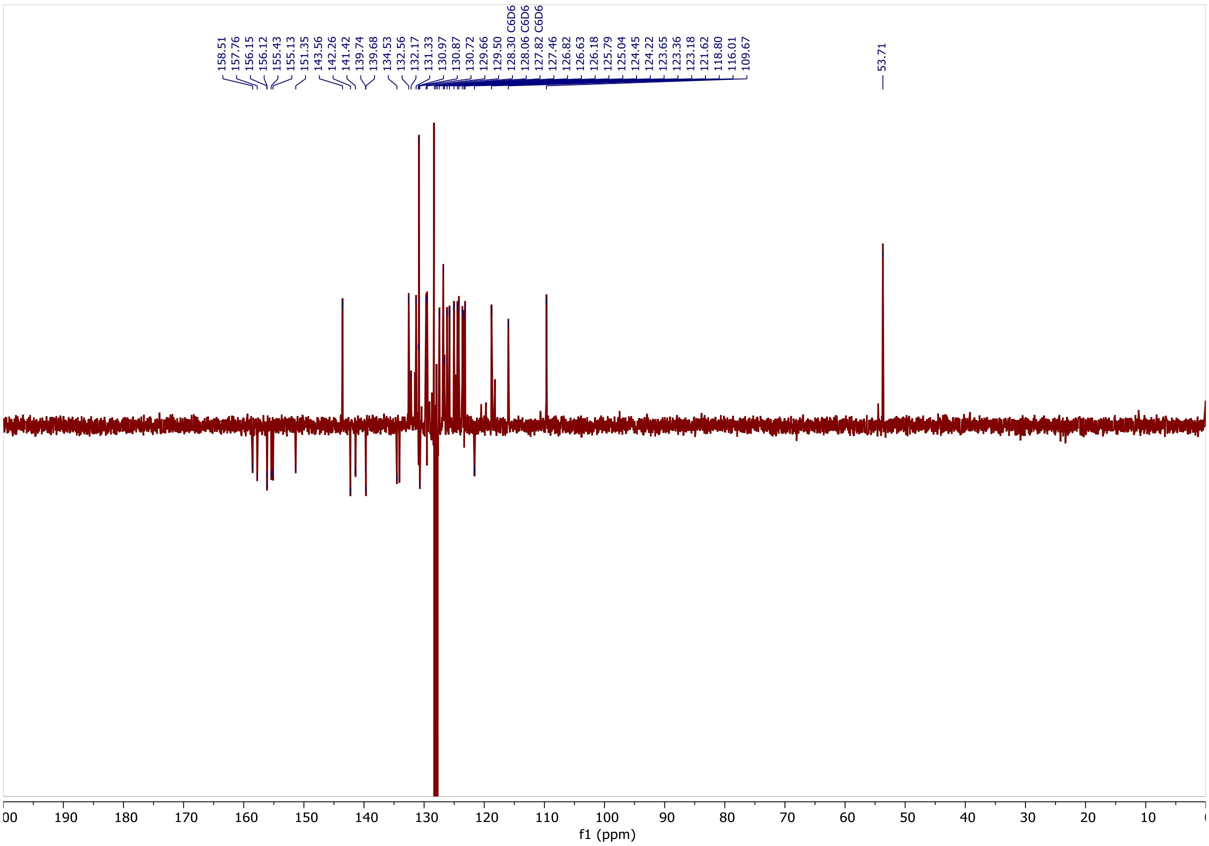

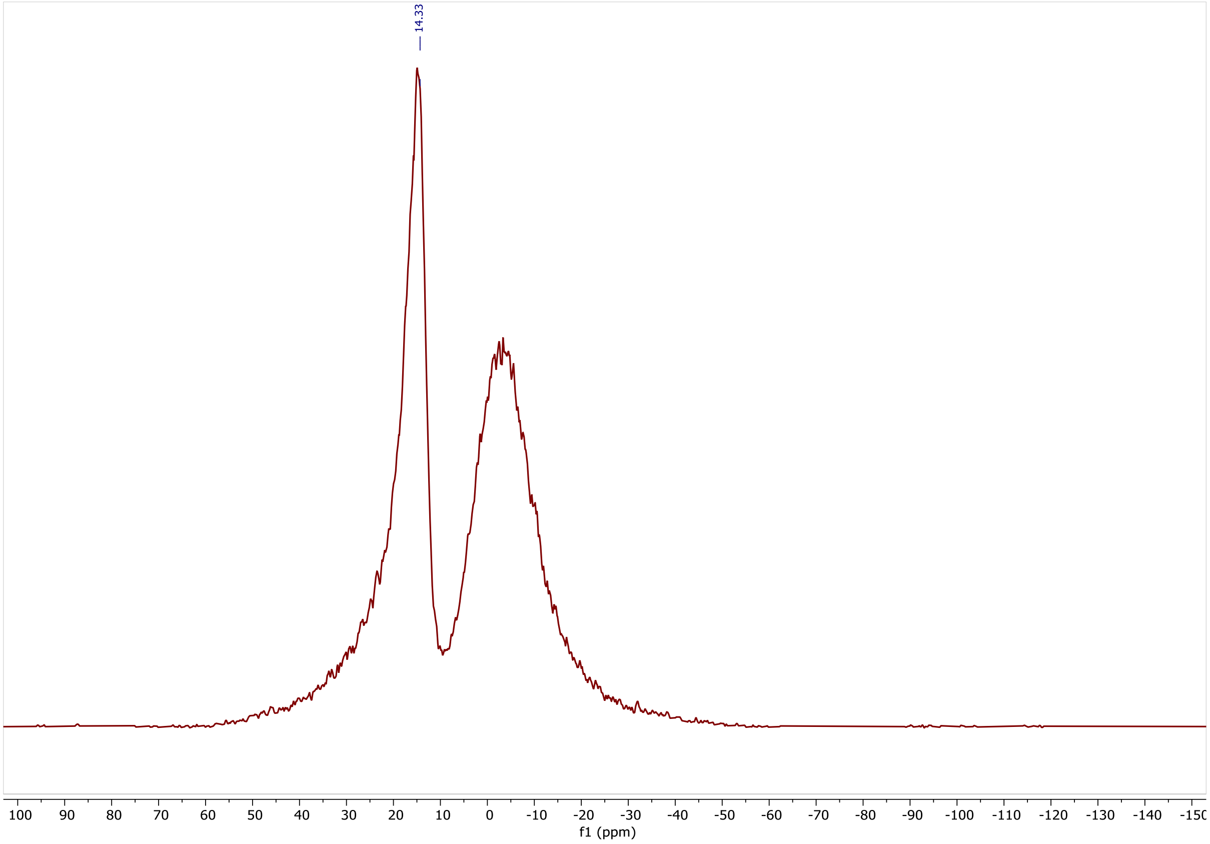
14.33
100 90 80 70 60 50 40 30 20 10 0 -10 -20 -30 -40 -50 -60 -70 -80 -90 -100 -110 -120 -130 -140 -150
f1 (ppm)

^{1}H, ^{13}C, and ^{11}B NMR spectra of (4S,10'R,11bR)-10'-(2-isopropoxyphenyl)-8',9'-dimethylspiro[dinaphtho[2,1-d:1',2'-f][1,3,2]dioxaborepine-4,11'-pyrido[3',2':3,4][1,2]azaborolo[1,5-a]quinolin]-12'-ium-26-uide (10e)

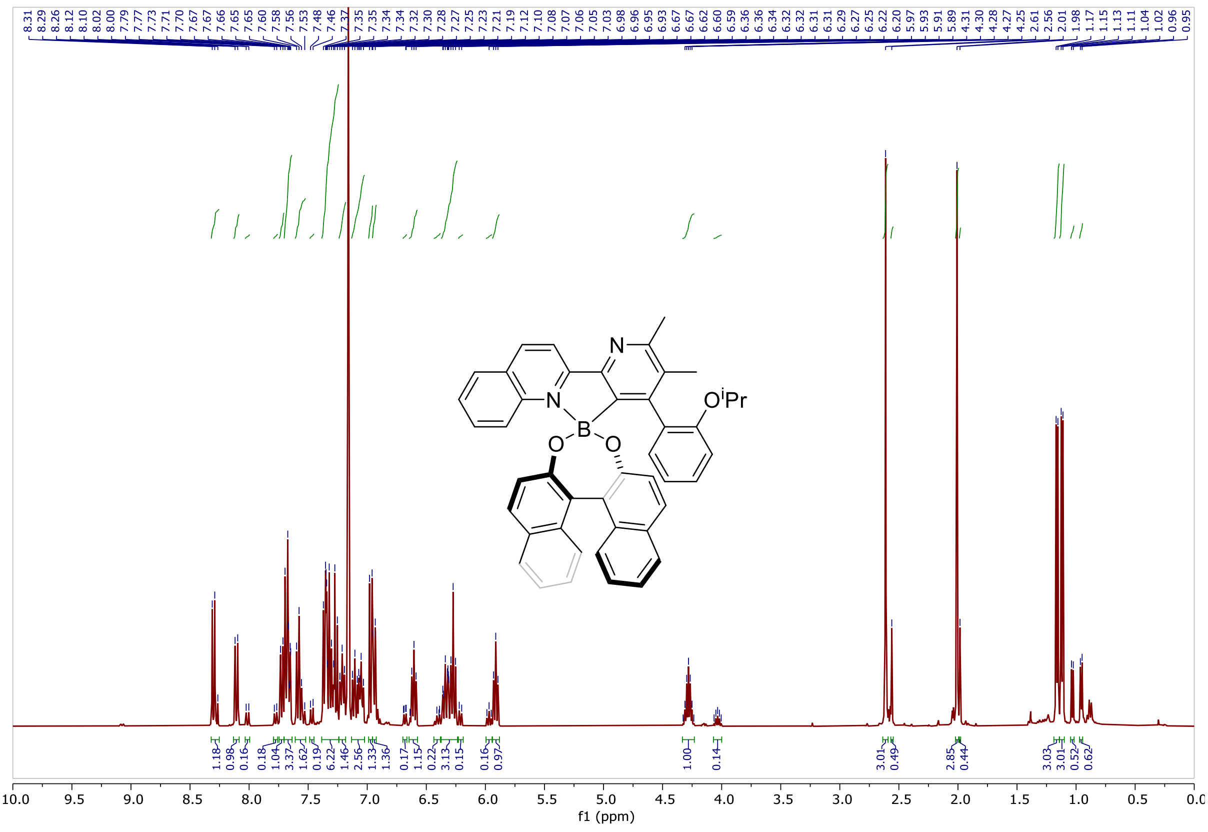

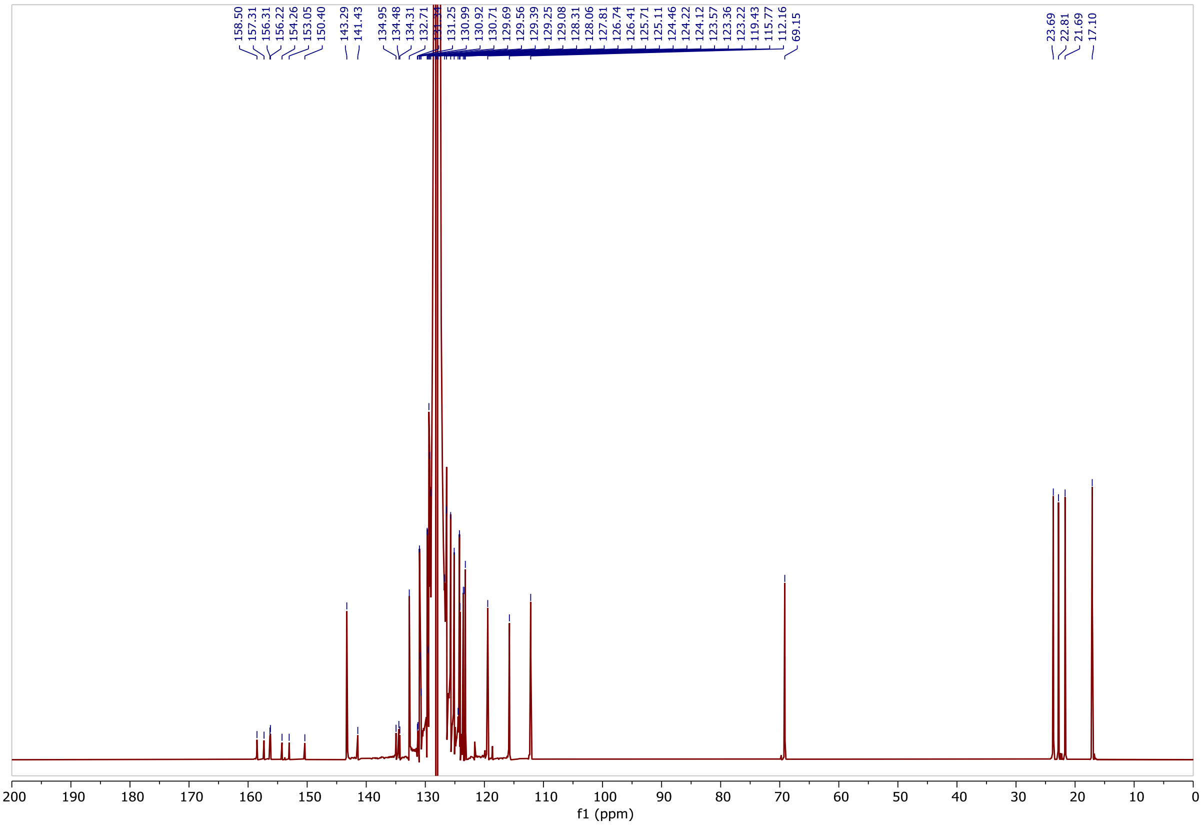

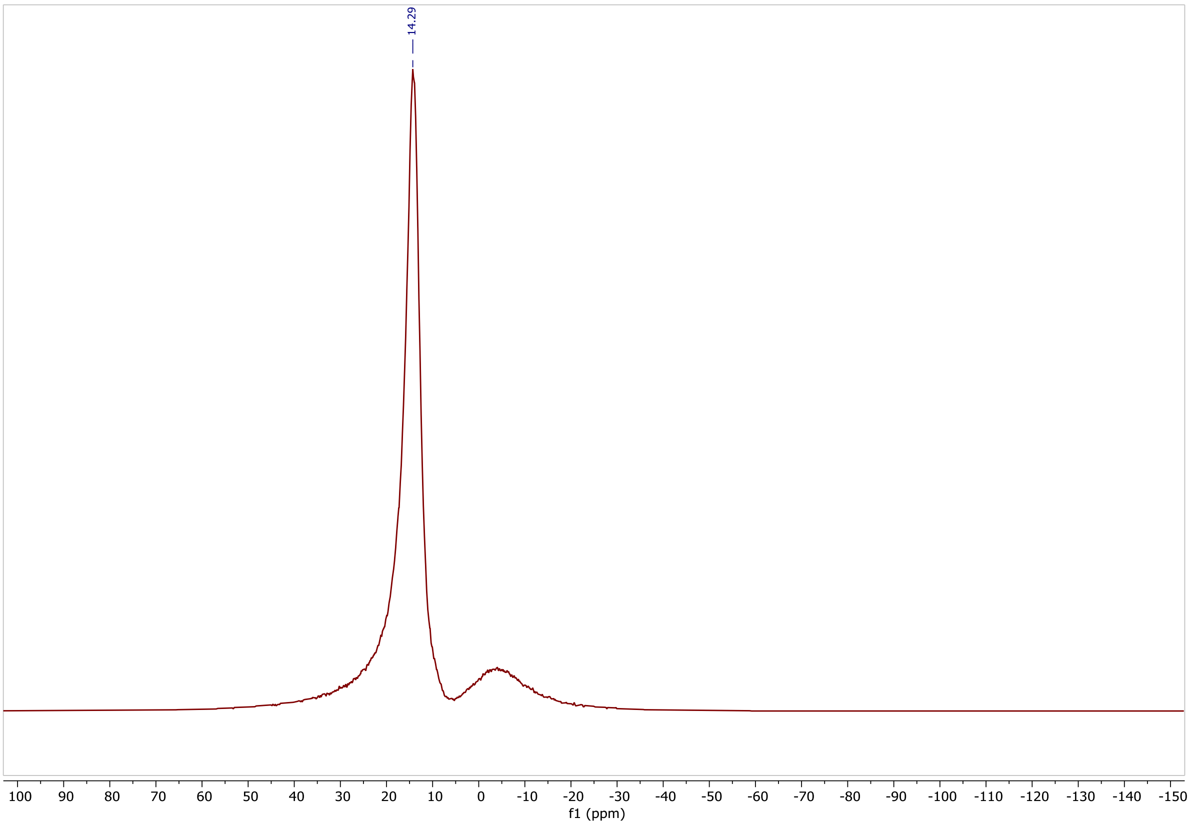
14.29
100 90 80 70 60 50 40 30 20 10 0 -10 -20 -30 -40 -50 -60 -70 -80 -90 -100 -110 -120 -130 -140 -150
f1 (ppm)

^{1}H, ^{13}C, and ^{11}B NMR spectra of (4S,10'R,11bR)-10'-(2-(dimethylamino)phenyl)-8',9'-dimethylspiro[dinaphtho[2,1-d:1',2'-f][1,3,2]dioxaborepine-4,11'-pyrido[3',2':3,4][1,2]azaborolo[1,5-a]quinolin]-12'-ium-26-uide (10f)

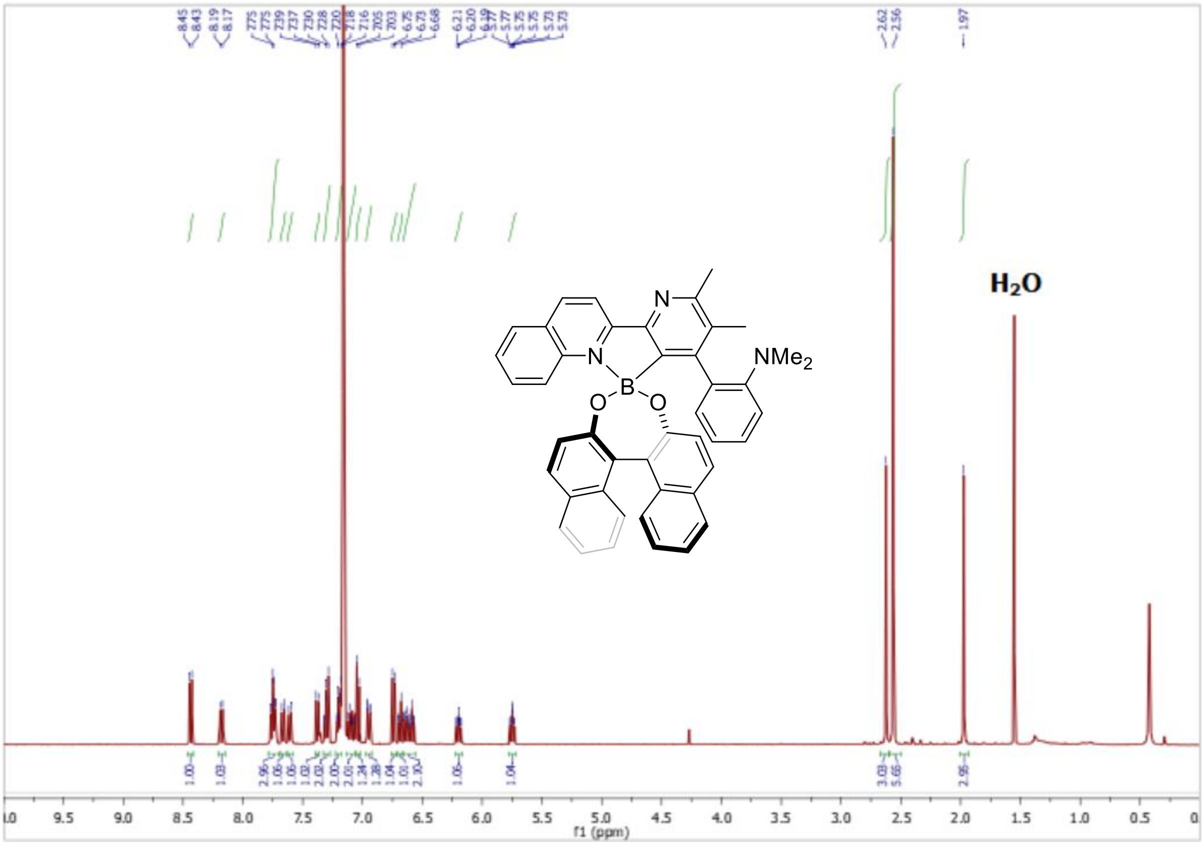

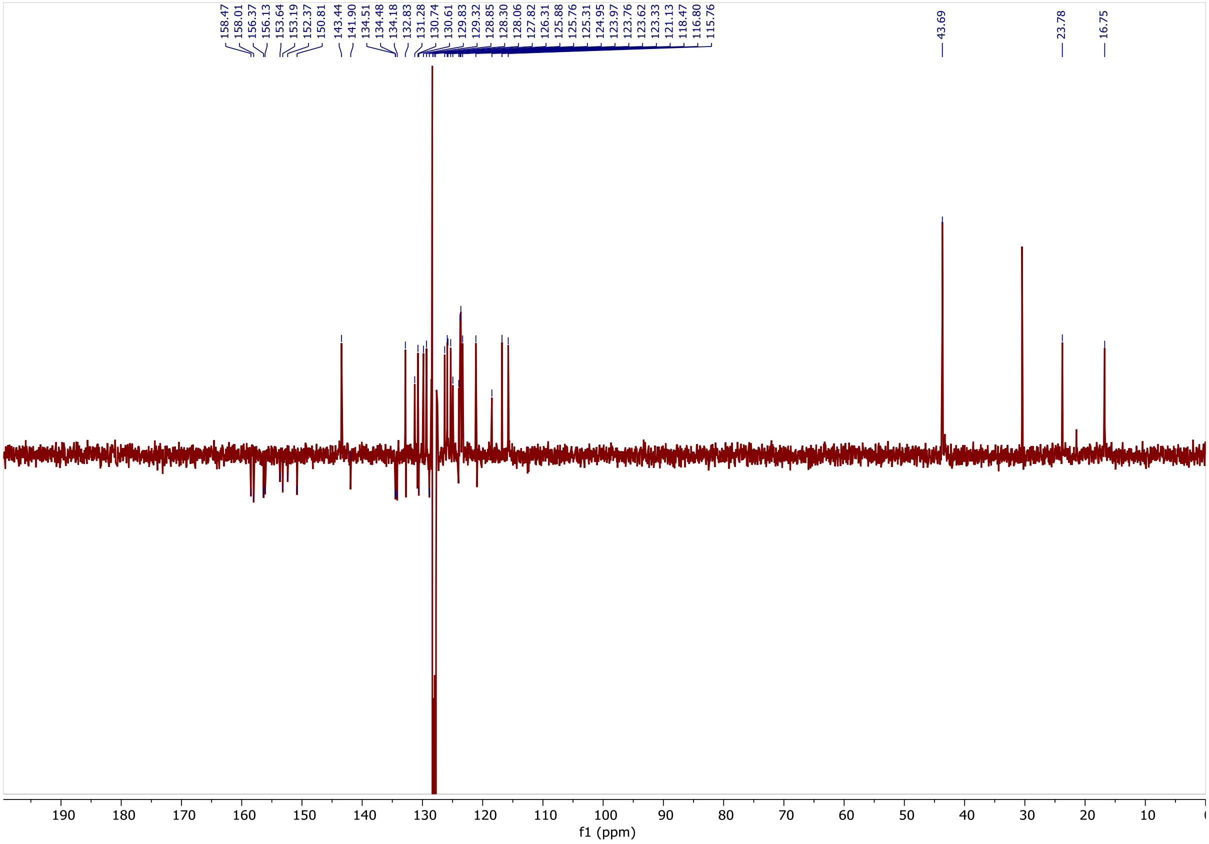

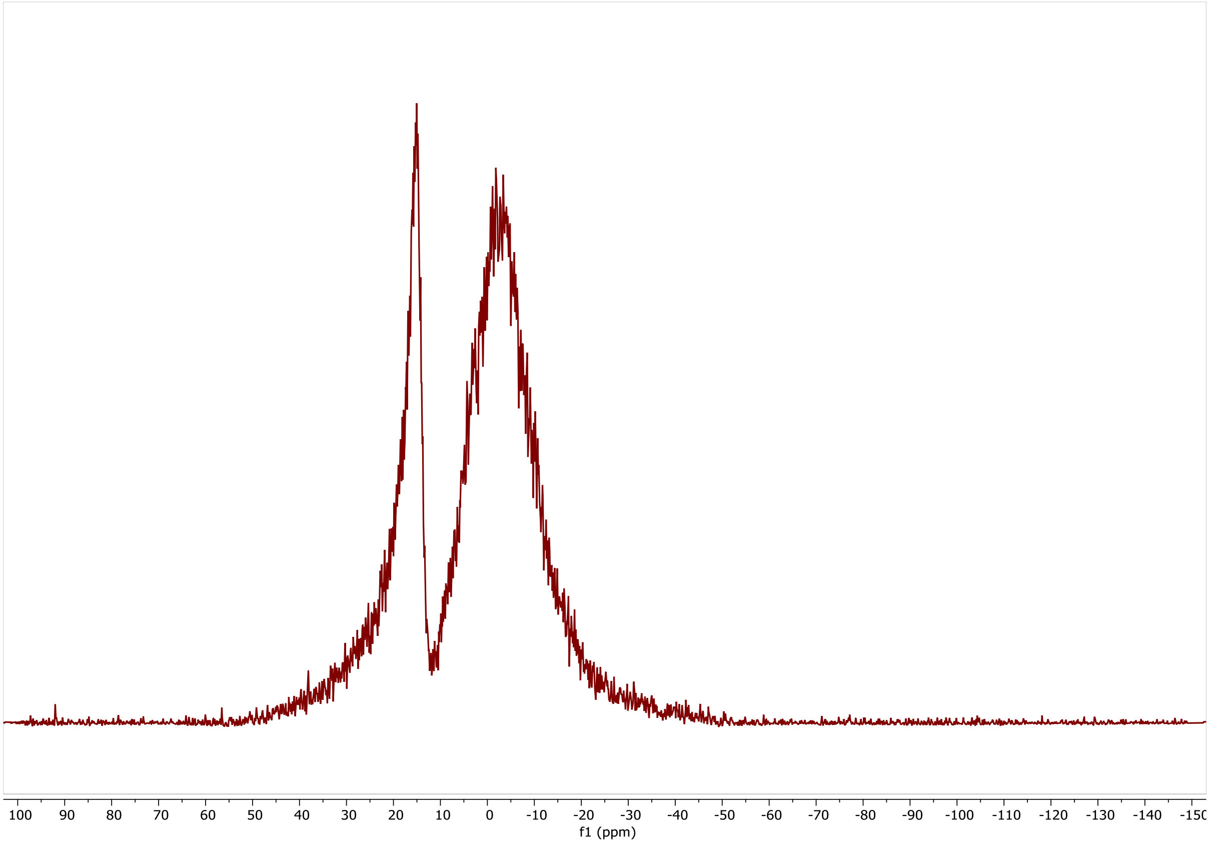
100
90
80
70
60
50
40
30
20
10
0
-10
-20
-30
-40
-50
-60
-70
-80
-90
-100
-110
-120
-130
-140
-150
f1 (ppm)

¹H, ¹³C, and ¹¹B NMR spectra of (4S,10'R,11bR)-8',9'-dimethyl-10'-(2-(methylthio)phenyl)spiro[dinaphtho[2,1-d:1',2'-f][1,3,2]dioxaborepine-4,11'-pyrido[3',2':3,4][1,2]azaborolo[1,5-a]quinolin]-12'-ium-26-uide (10g)

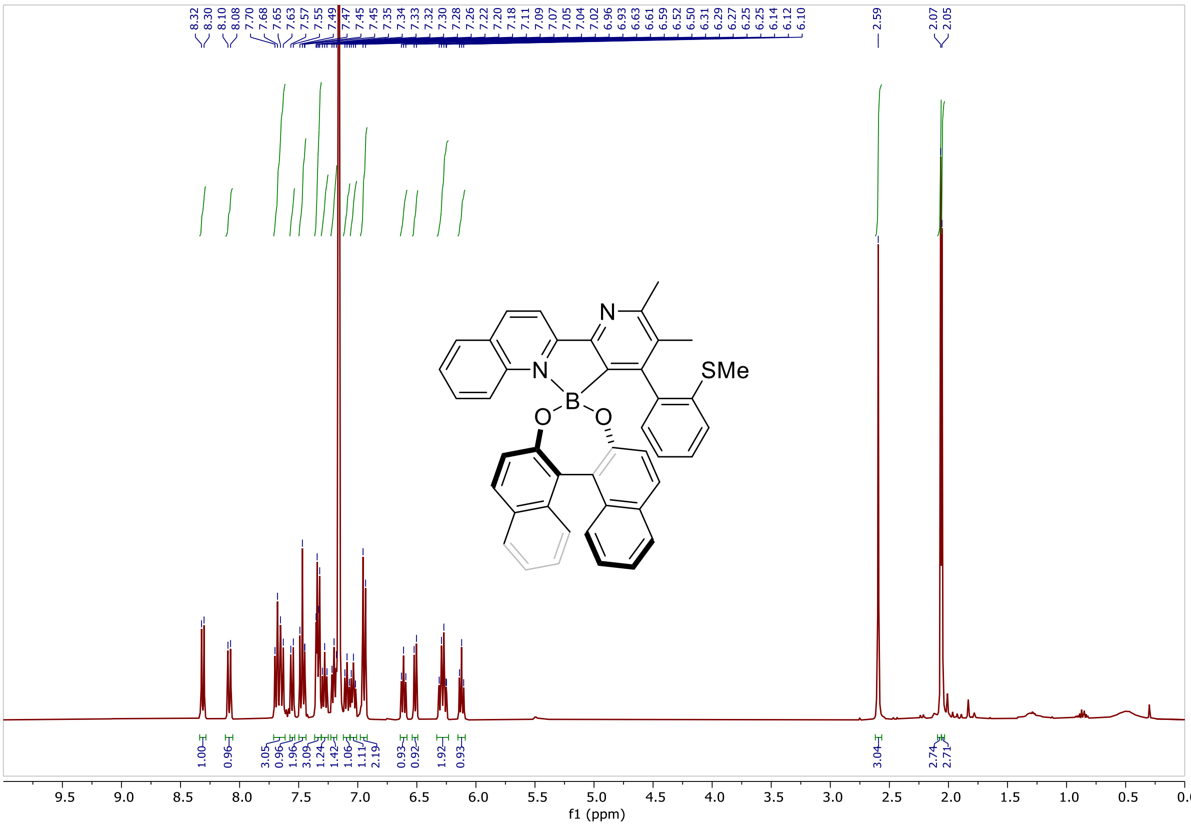

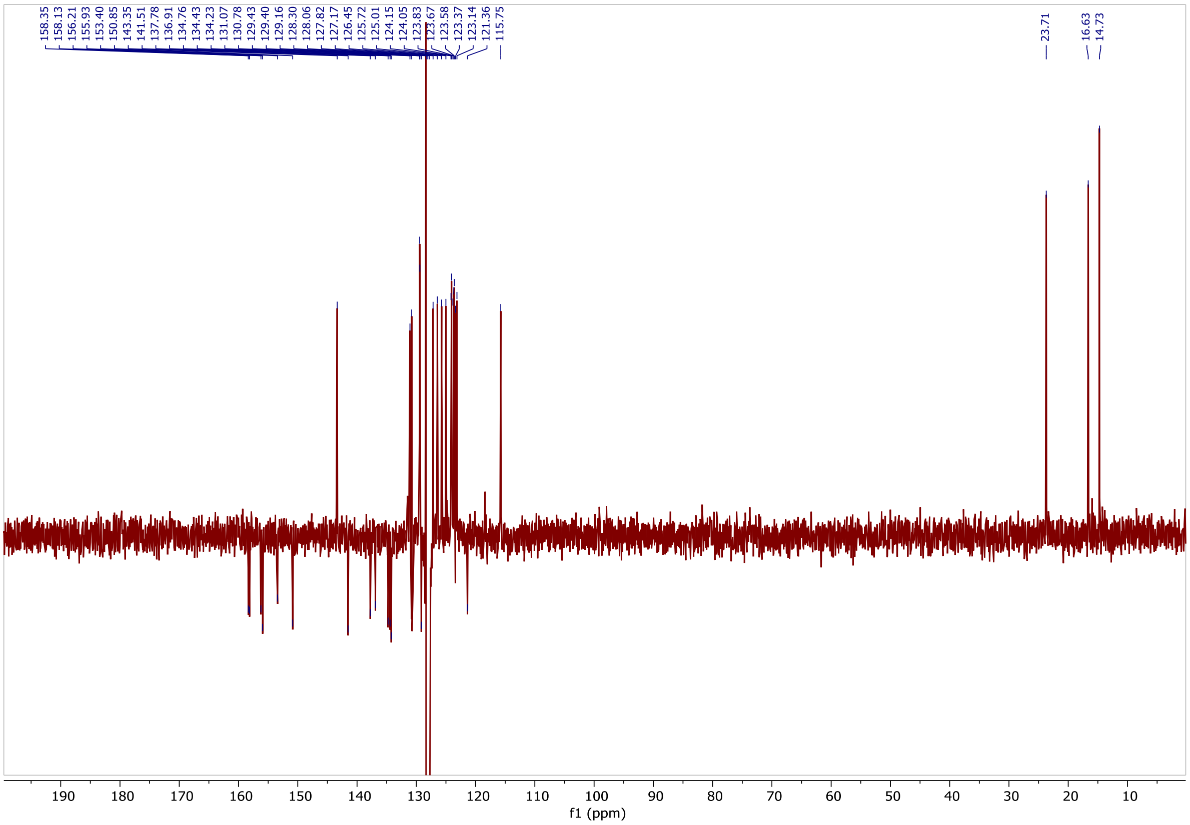

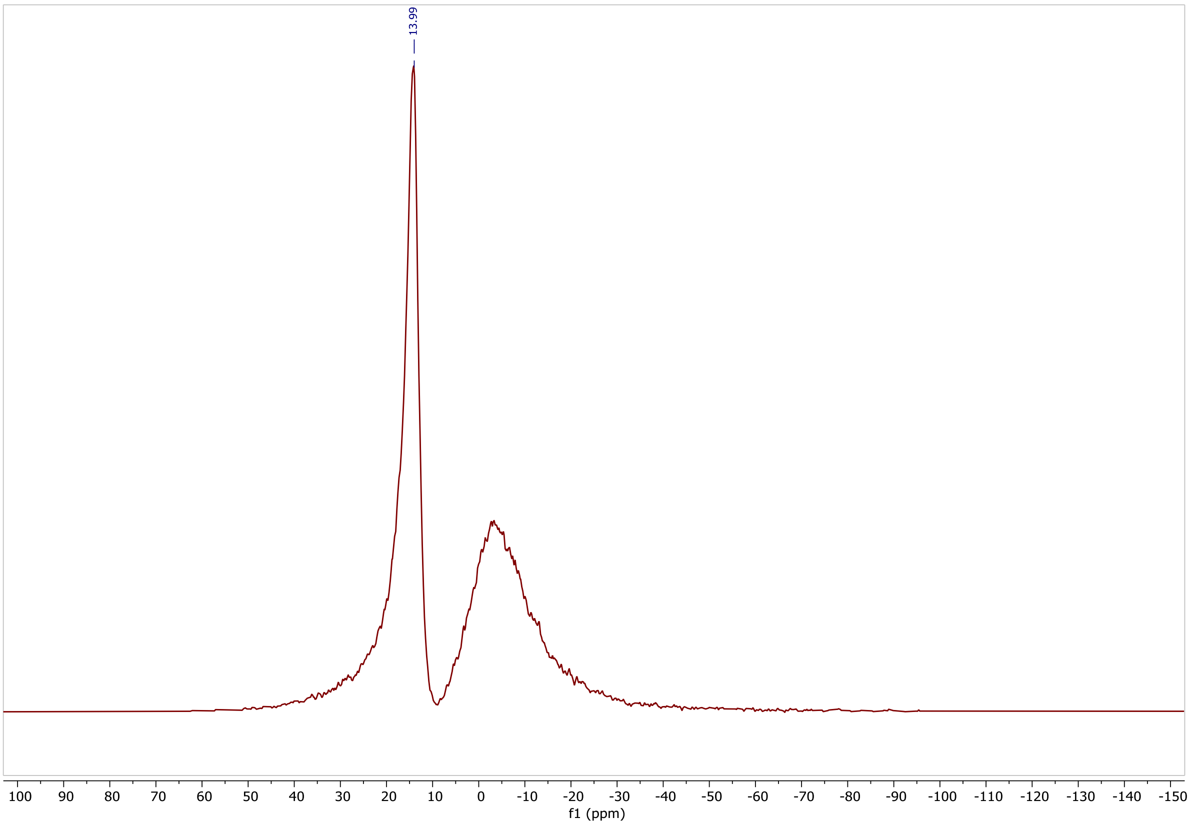

13.99
100 90 80 70 60 50 40 30 20 10 0 -10 -20 -30 -40 -50 -60 -70 -80 -90 -100 -110 -120 -130 -140 -150
f1 (ppm)

^{1}H, ^{13}C, and ^{11}B NMR spectra of (4S,11bR)-9'-(2-ethylphenyl)-10',11'-dimethylspiro[dinaphtho[2,1-d:1',2'-f][1,3,2]dioxaborepine-4,8'-pyrido[3',2':3,4][1,2]azaborolo[5,1-a]isoquinolin]-7'-ium-23-uide (10h)

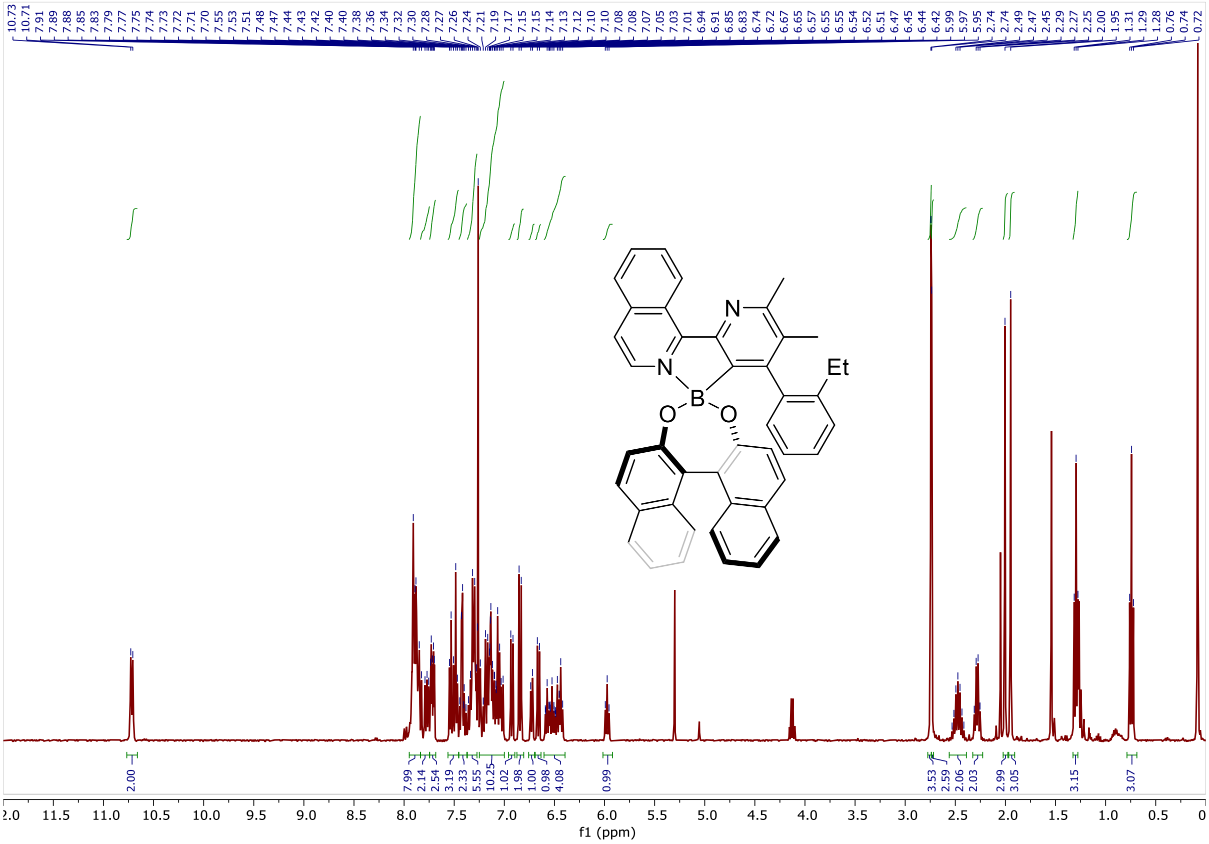

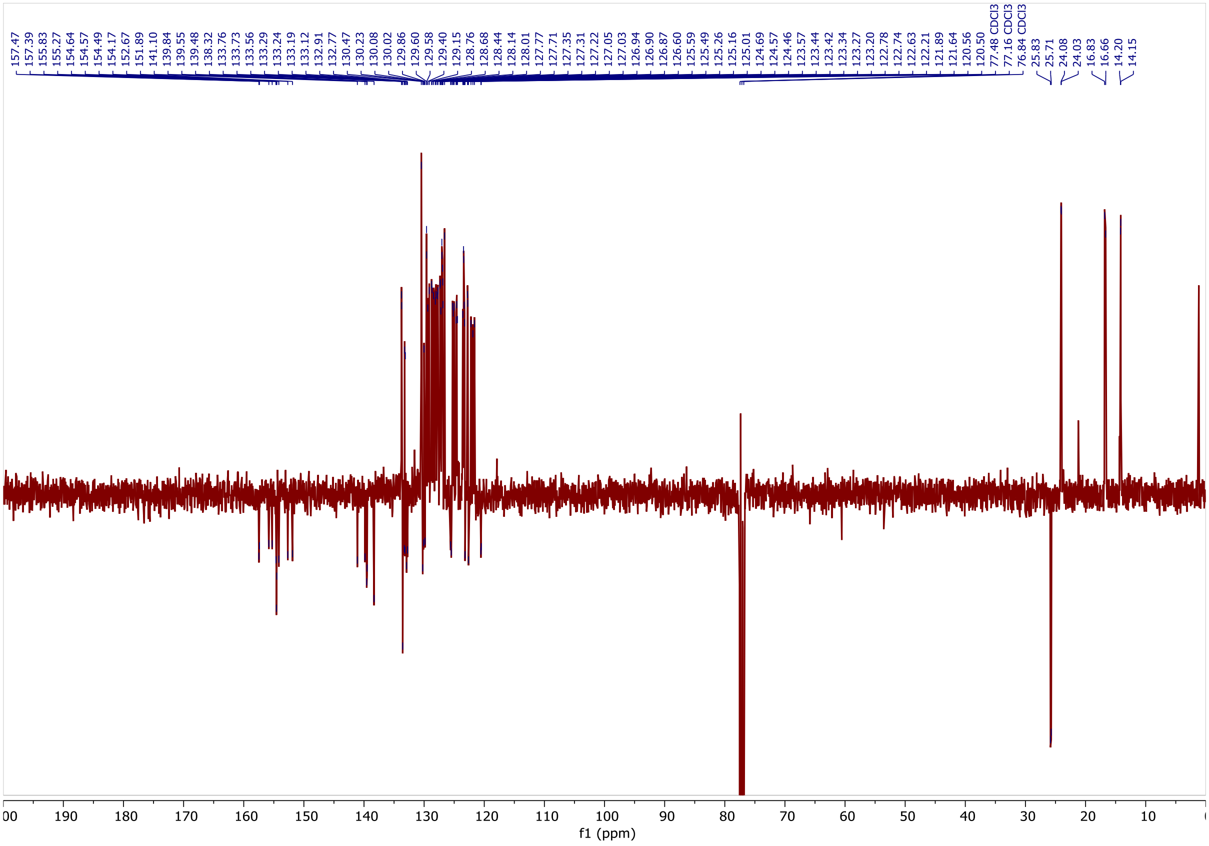

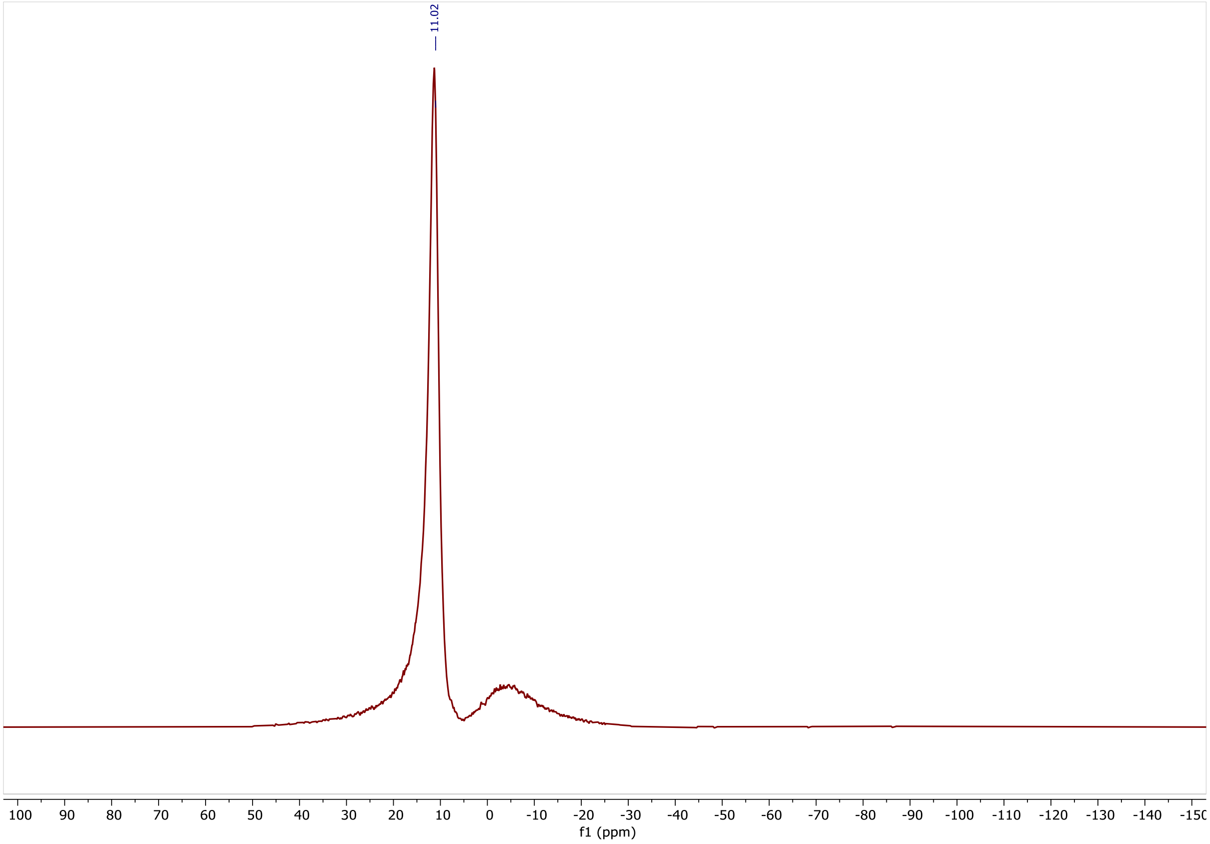
11.02
100
90
80
70
60
50
40
30
20
10
0
-10
-20
-30
-40
-50
-60
-70
-80
-90
-100
-110
-120
-130
-140
-150
f1 (ppm)

^{1}H, ^{13}C, and ^{11}B NMR spectra of (4S,9'R,11bR)-9'-(2-methoxyphenyl)-10',11'-dimethylspiro[dinaphtho[2,1-d:1',2'-f][1,3,2]dioxaborepine-4,8'-pyrido[3',2':3,4][1,2]azaborolo[5,1-a]isoquinolin]-7'-ium-23-uide (10i)

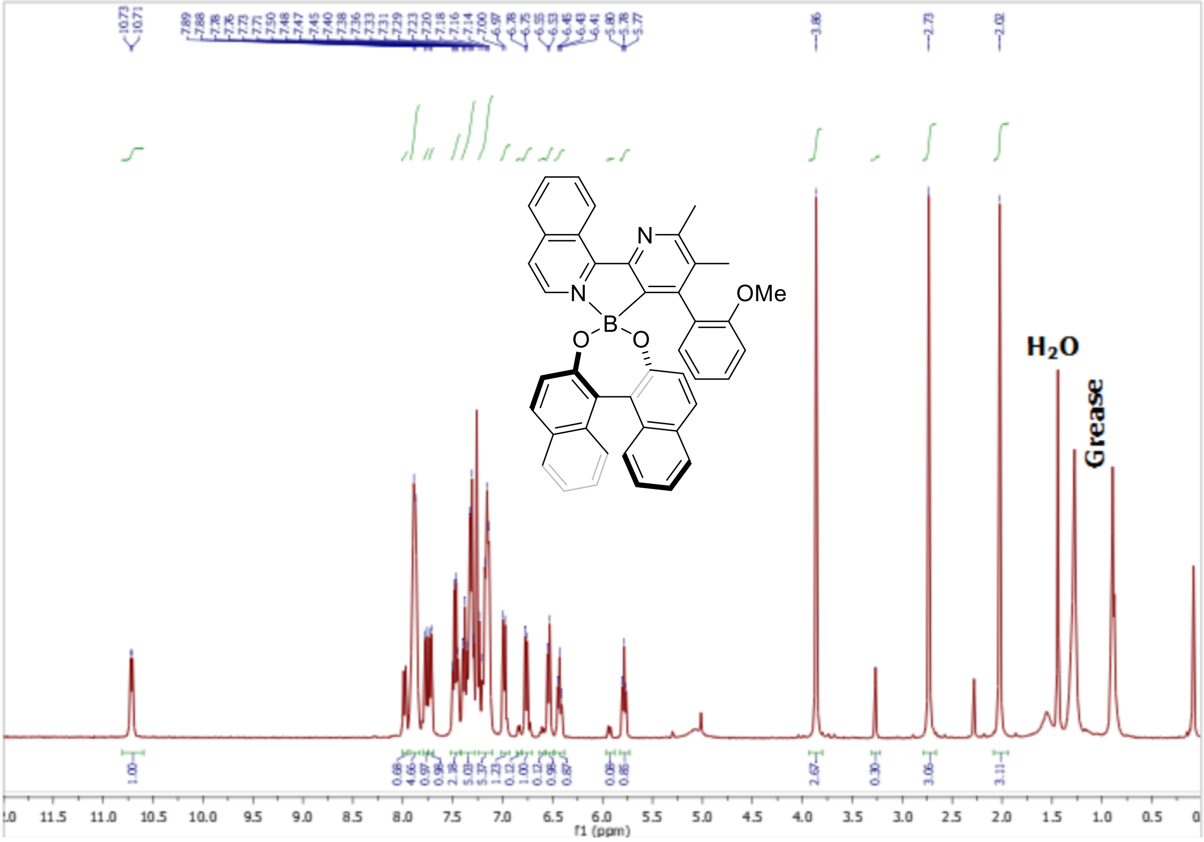

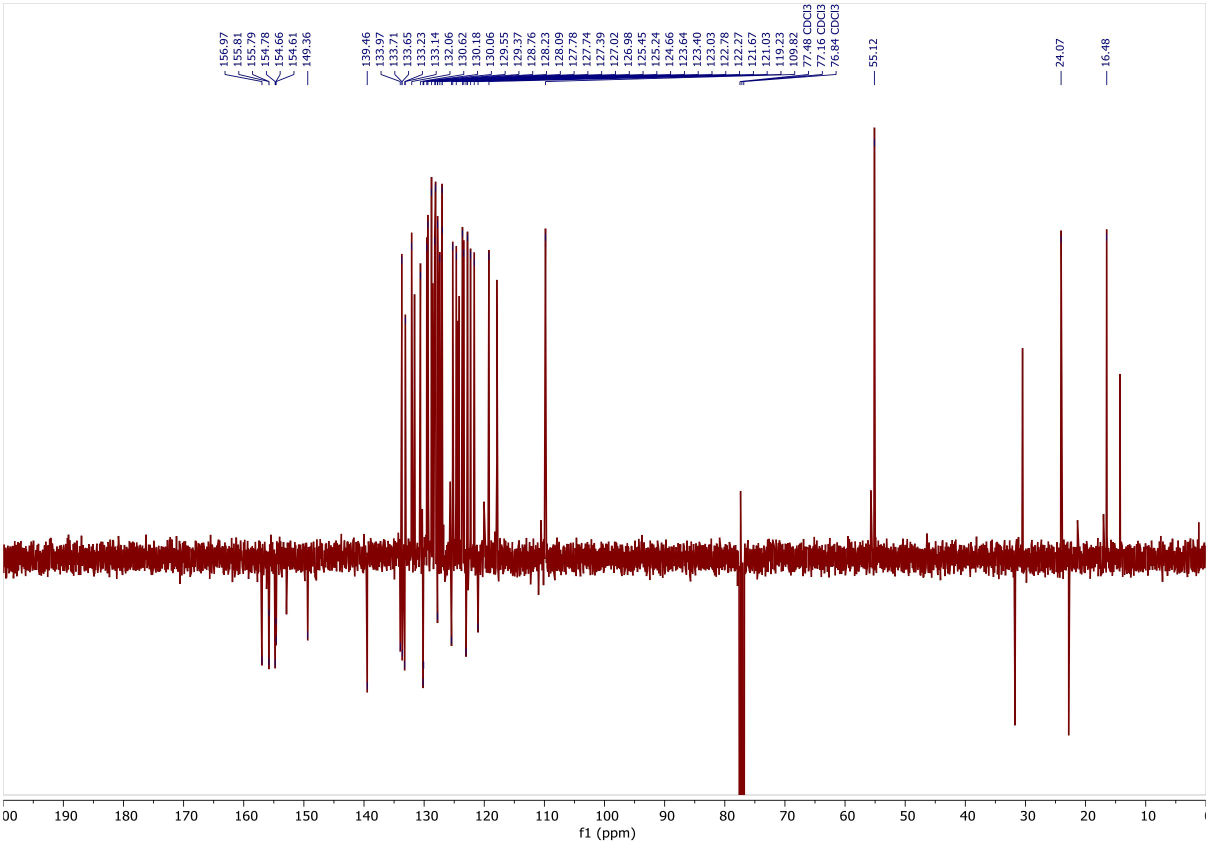

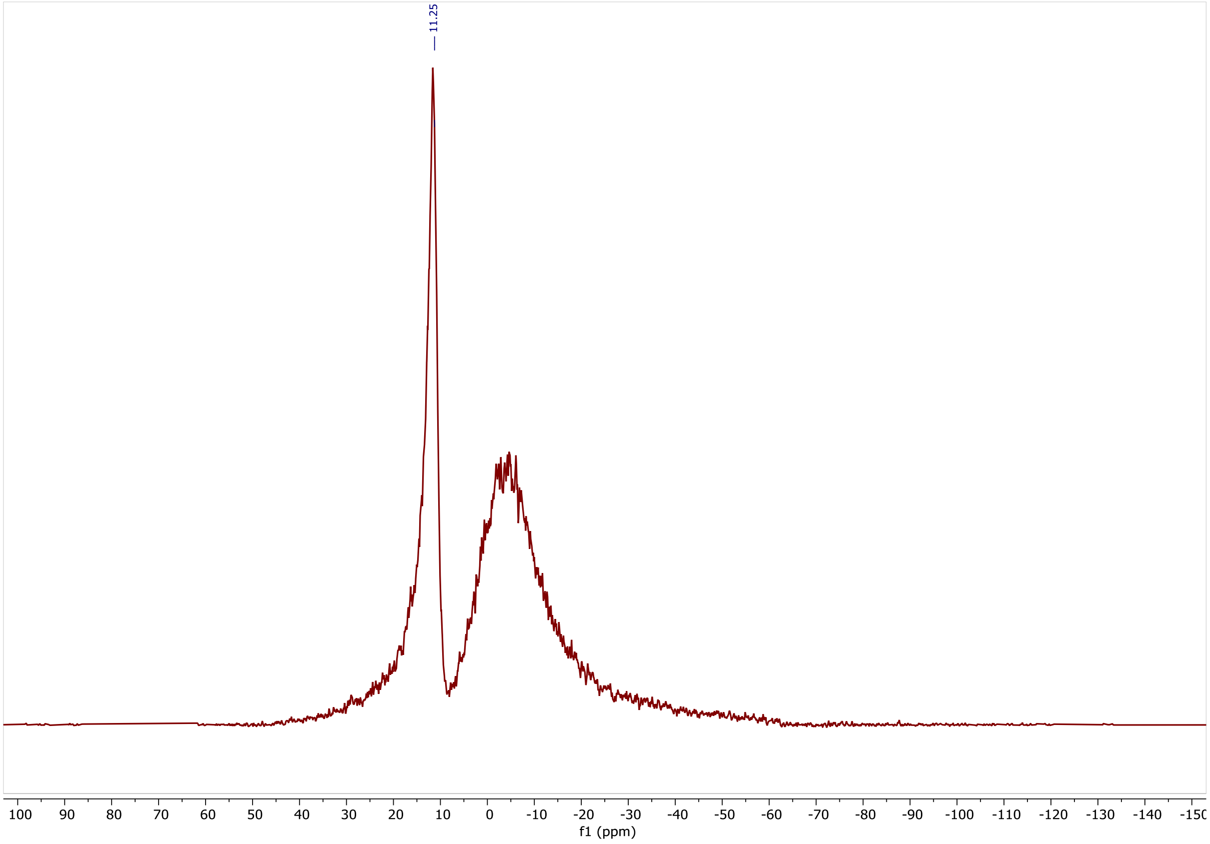
11.25
100
90
80
70
60
50
40
30
20
10
0
-10
-20
-30
-40
-50
-60
-70
-80
-90
-100
-110
-120
-130
-140
-150
f1 (ppm)

^{1}H, ^{13}C, and ^{11}B NMR spectra of (4S,11bR)-9'-(2-ethylphenyl)-10',11'-diphenylspiro[dinaphtho[2,1-d:1',2'-f][1,3,2]dioxaborepine-4,8'-pyrido[3',2':3,4][1,2]azaborolo[5,1-a]isoquinolin]-7'-ium-23-uide (10j)

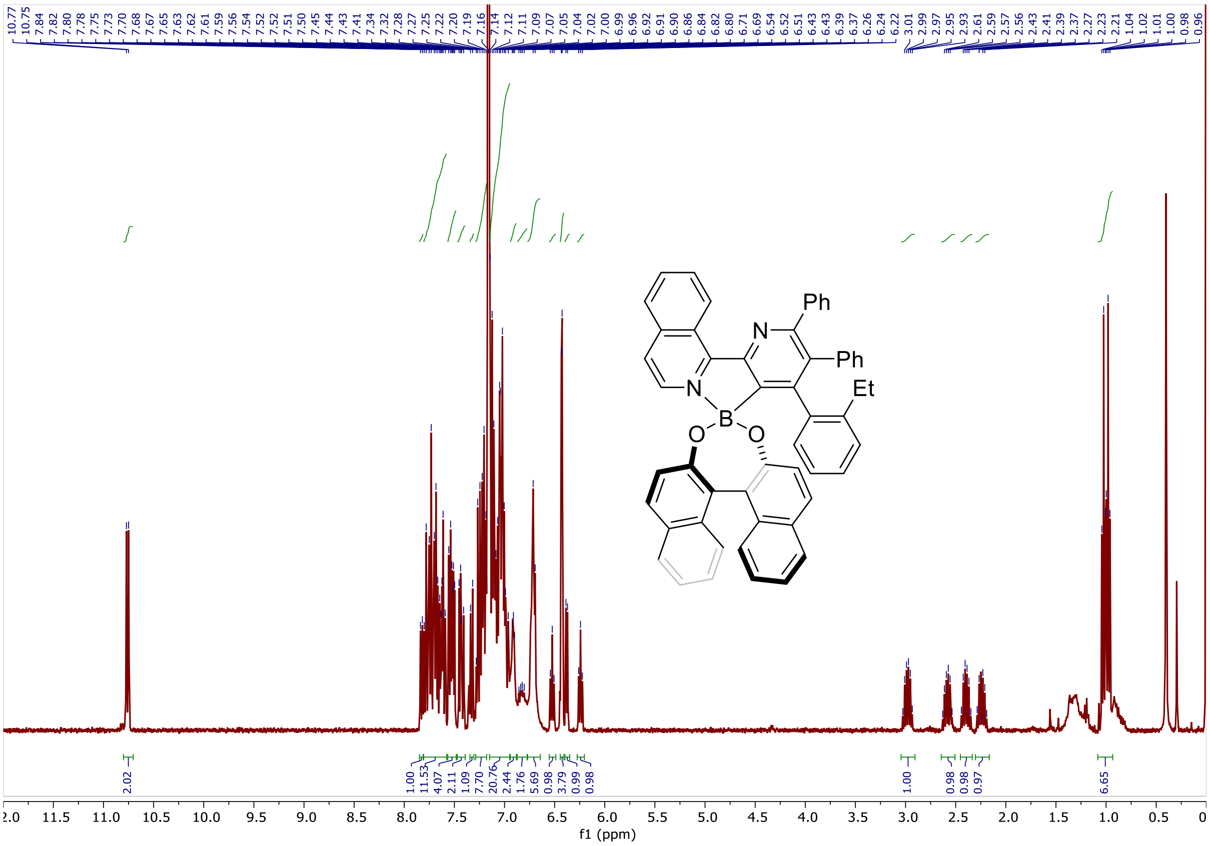

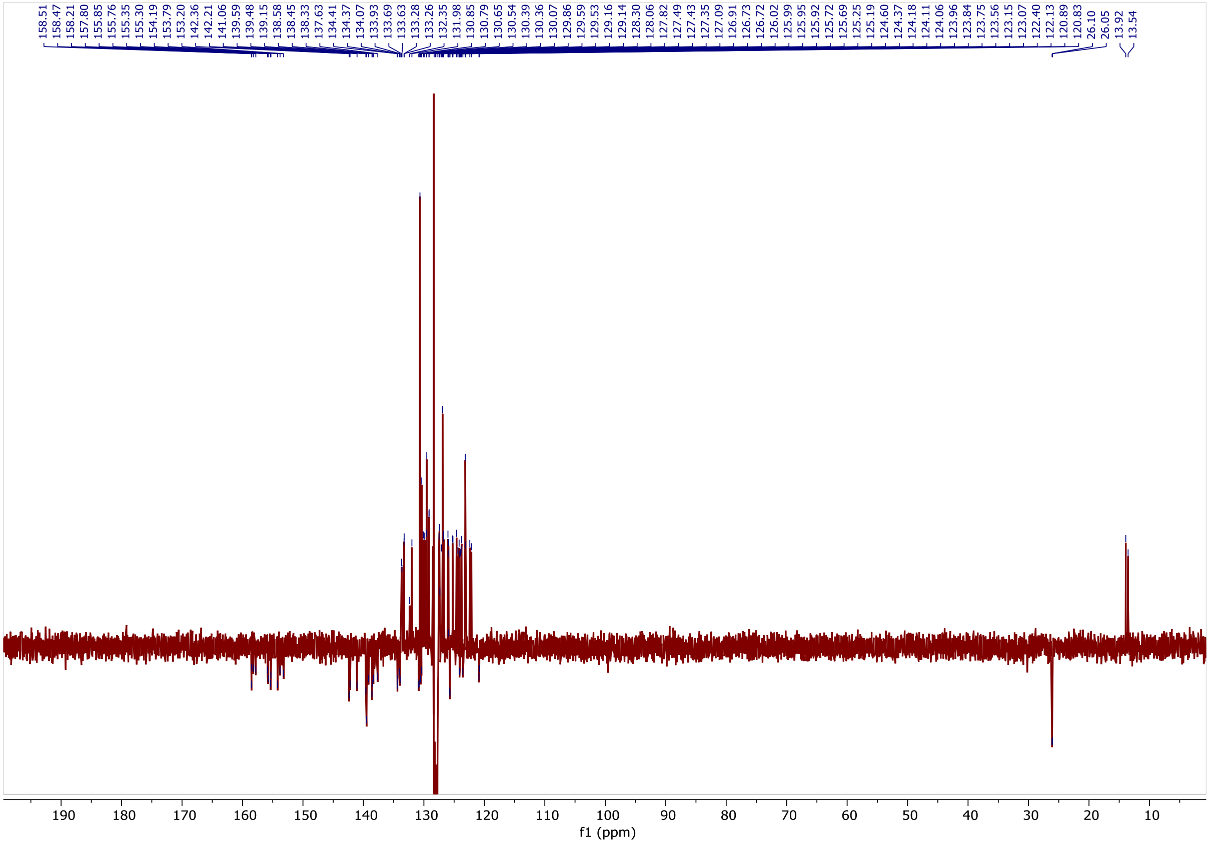

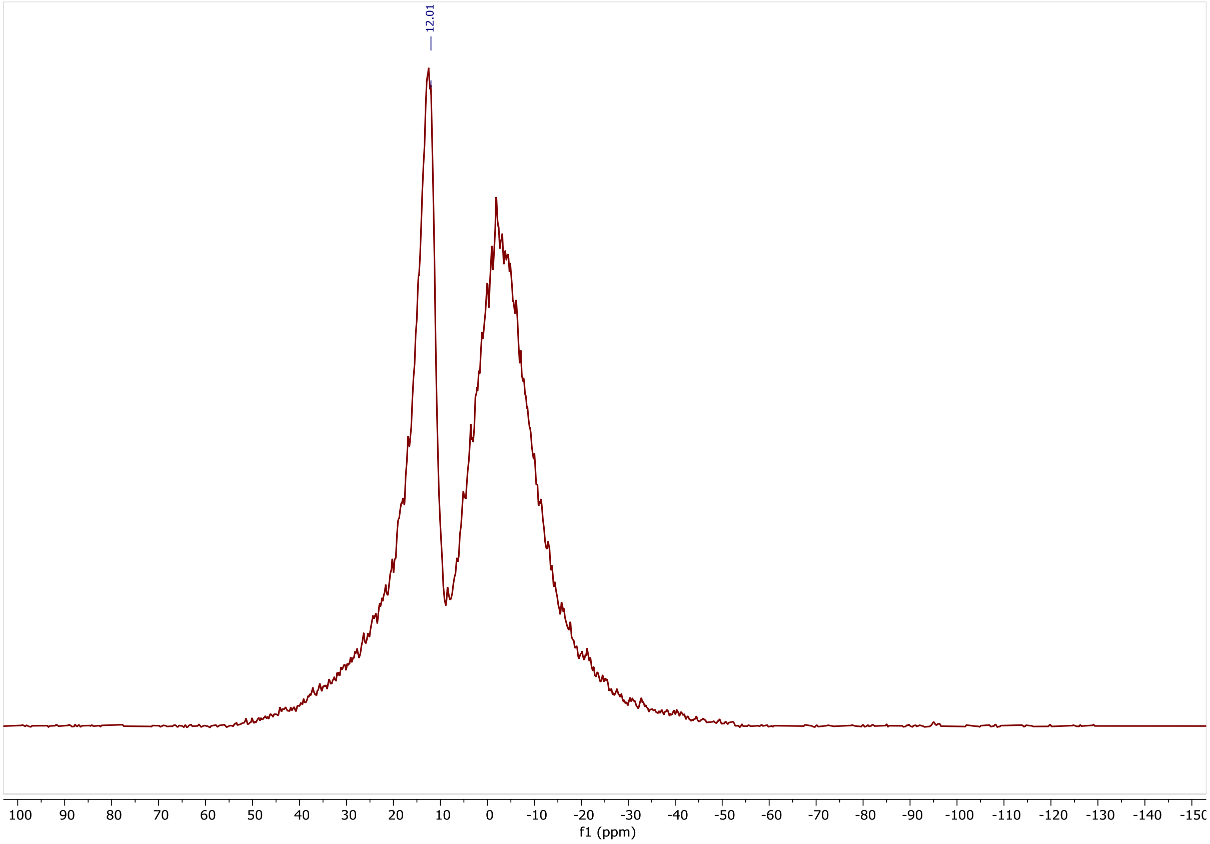
12.01
100
90
80
70
60
50
40
30
20
10
0
-10
-20
-30
-40
-50
-60
-70
-80
-90
-100
-110
-120
-130
-140
-150
f1 (ppm)

^{1}H, ^{13}C, and ^{11}B NMR spectra of (4S,9’R,11bR)-9’-(2-methoxyphenyl)-10’-11’-diphenylspiro[dinaphto[2,1-d:1’,2’-f][1,3,2]dioxaborepine-4,8’-pyrido[3’,2’:3,4][1,2]azaborolo[5,1-a]isoquinolin]-7’-ium-23-uide (10k)

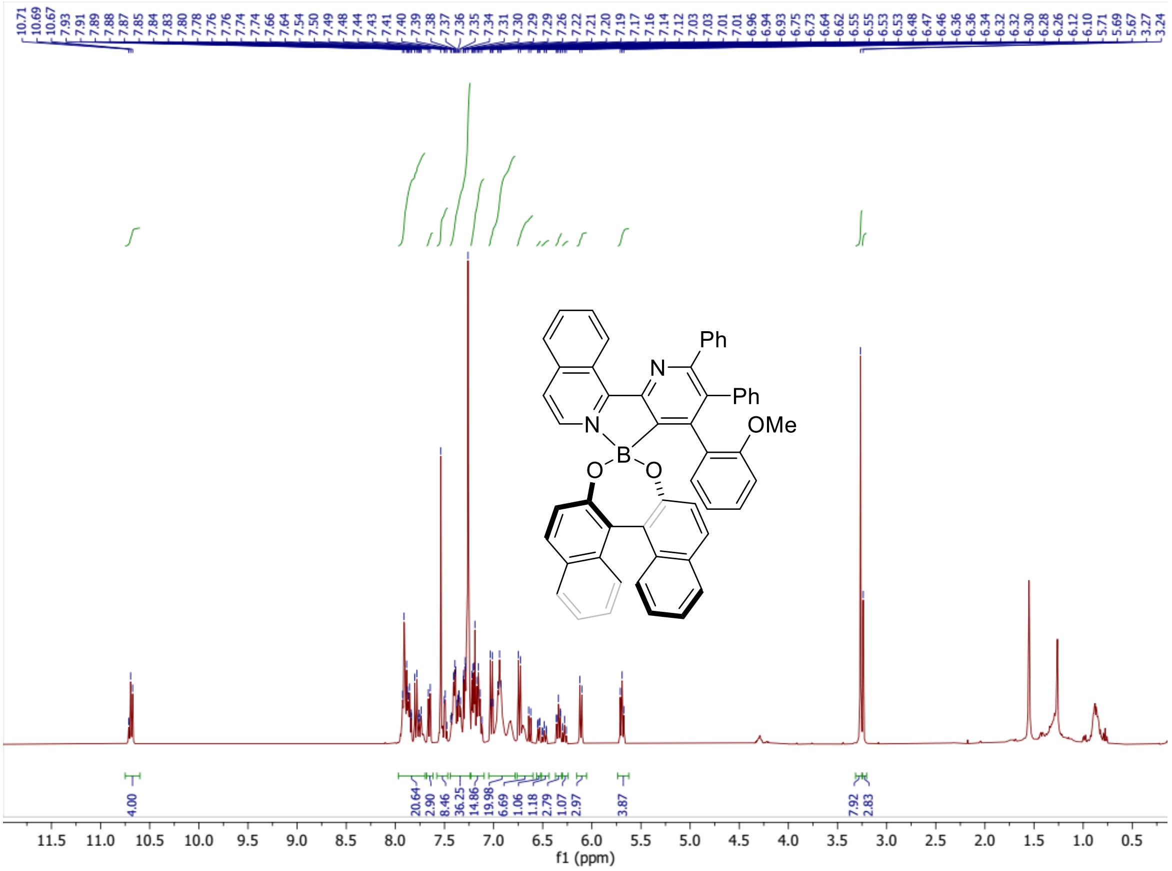

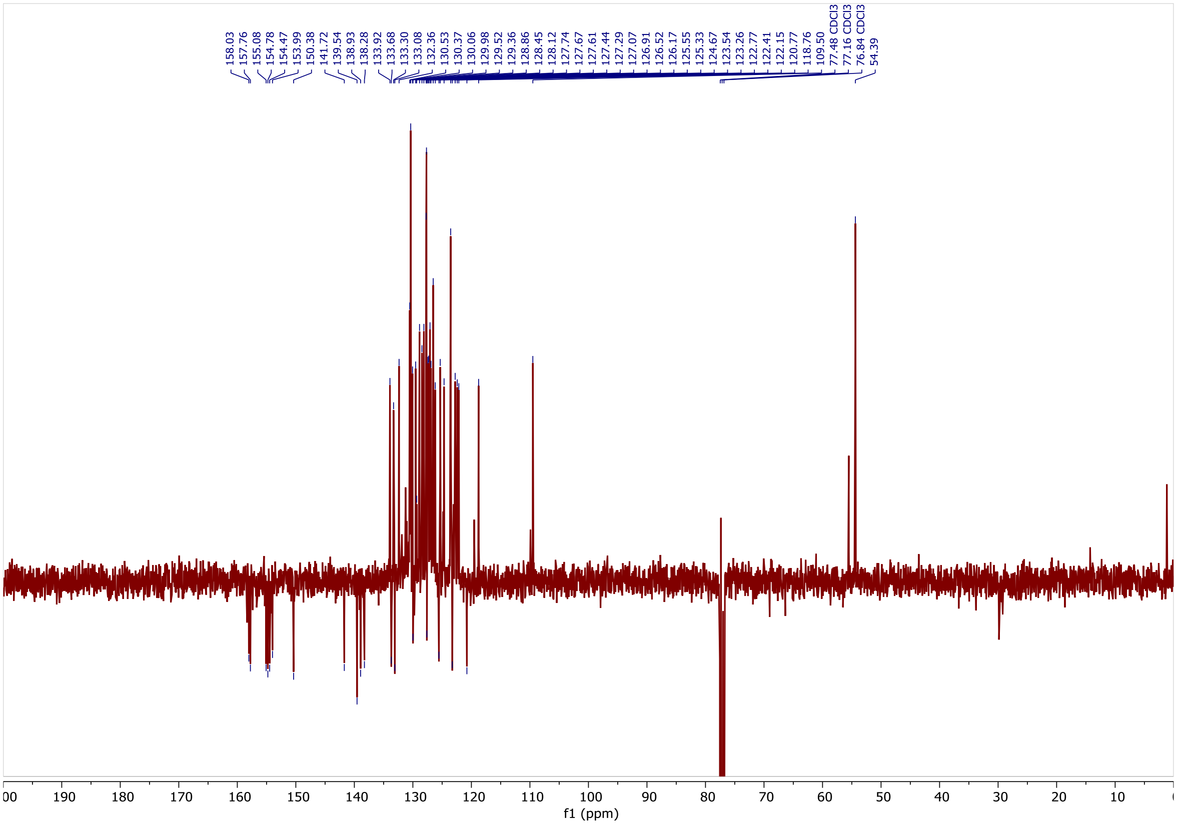

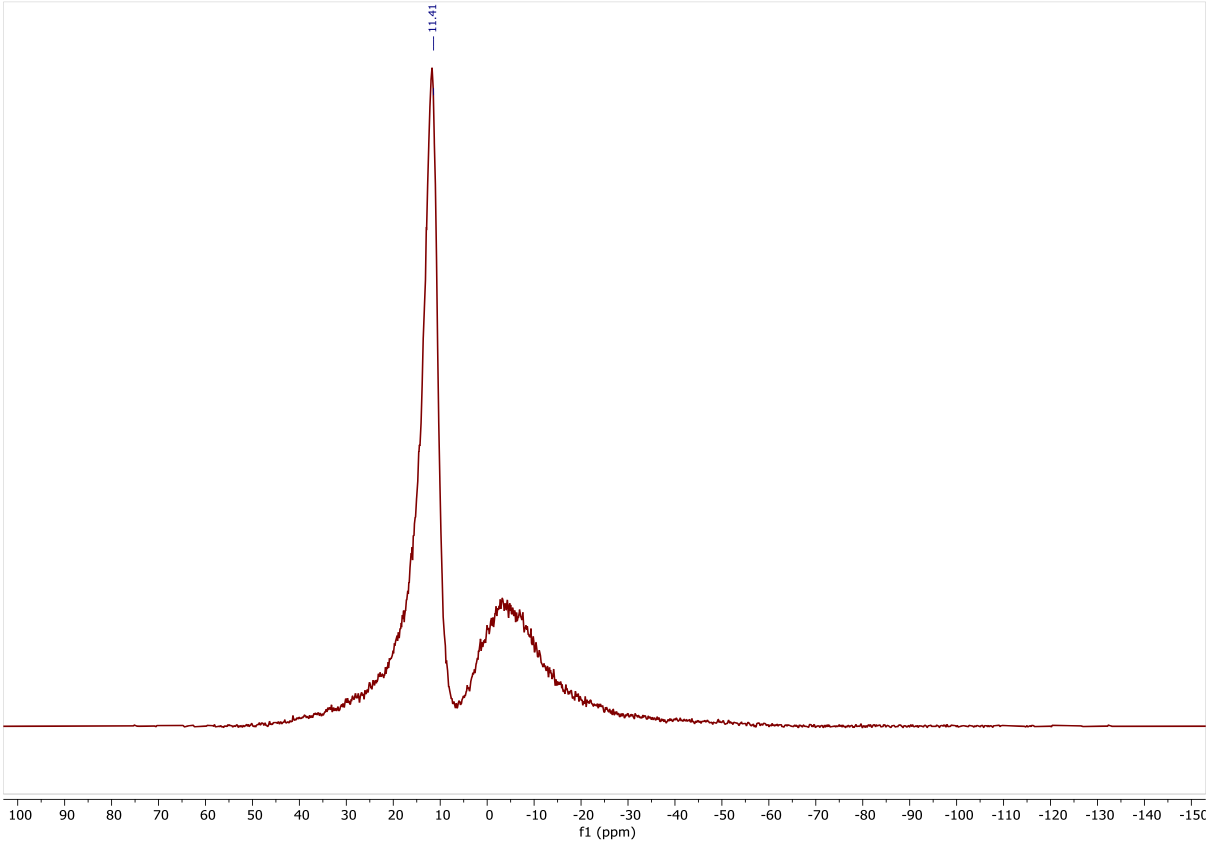
11.41
100
90
80
70
60
50
40
30
20
10
0
-10
-20
-30
-40
-50
-60
-70
-80
-90
-100
-110
-120
-130
-140
-150
f1 (ppm)

^{1}H, ^{13}C, and ^{11}B NMR spectra of (4S,4'R,11b'R)-4-(2-methoxyphenyl)-2,3-dimethylspiro[[1,2]azaborolo[1,5-a:4,3-b']dipyridine-5,4'-dinaphto[2,1-d:1',2'-f][1,3,2]dioxaborepin]-6-ium-14-uide (10l)

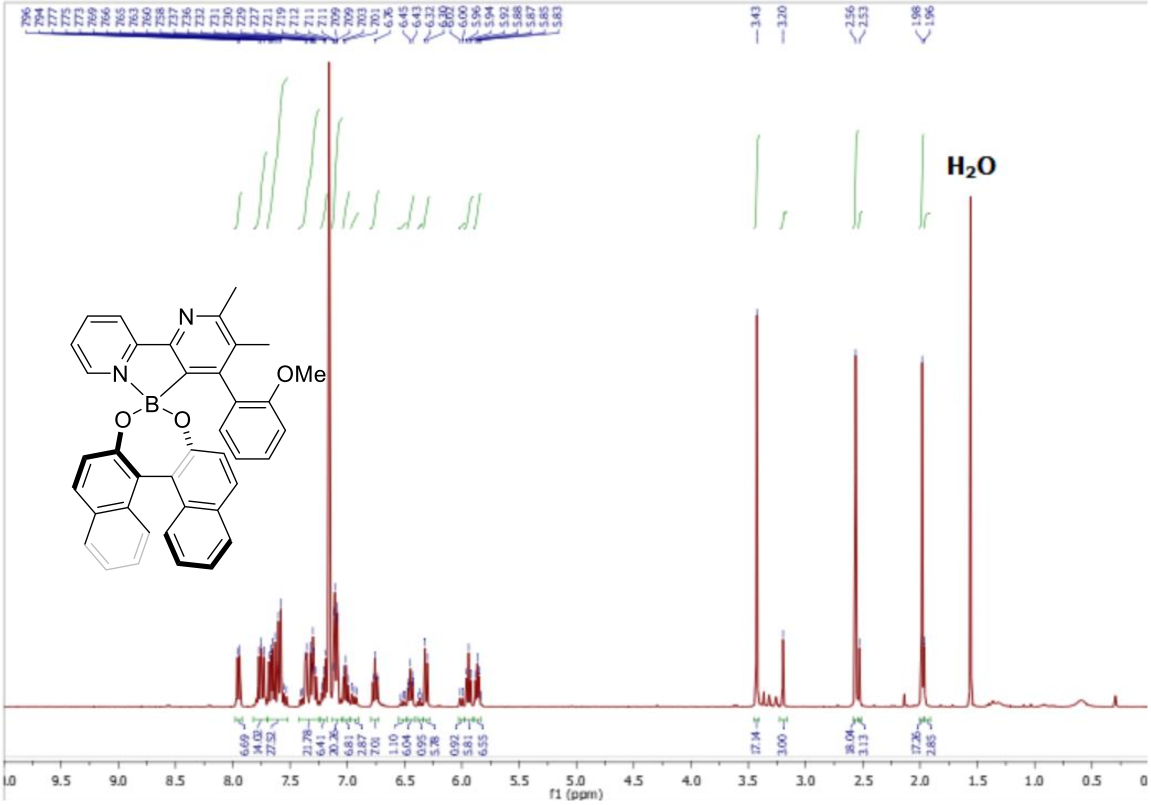

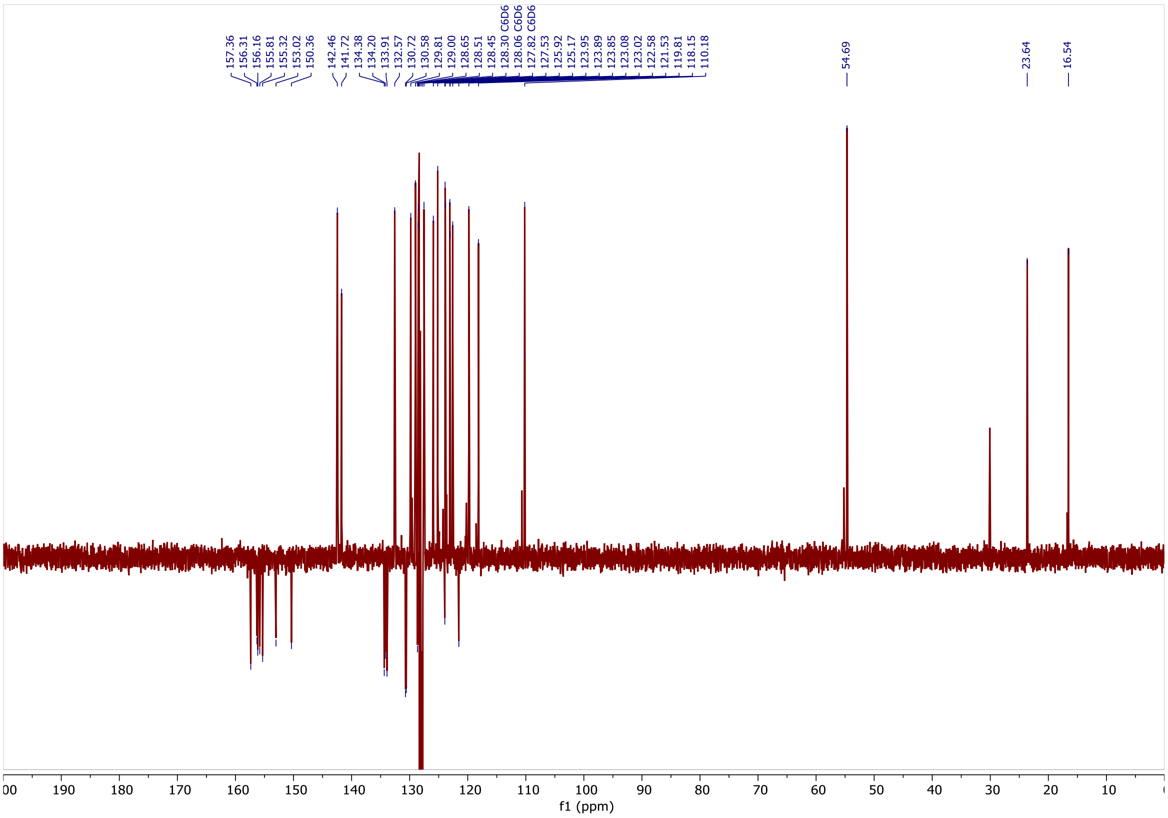

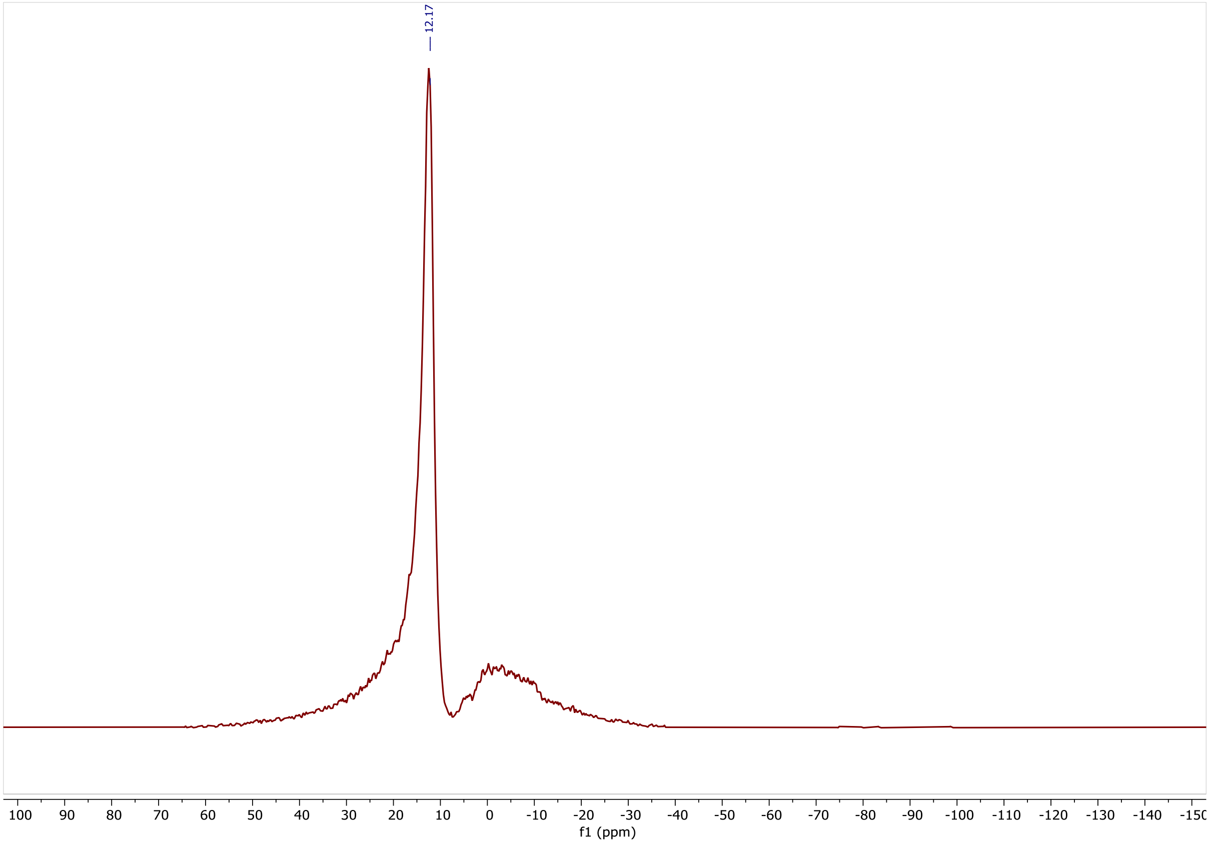
12.17
100
90
80
70
60
50
40
30
20
10
0
-10
-20
-30
-40
-50
-60
-70
-80
-90
-100
-110
-120
-130
-140
-150
f1 (ppm)

^{1}H, ^{13}C, and ^{11}B NMR spectra of 2-(3-((R)-Dinaphtho[2,1-d:1',2'-f][1,3,2]dioxaborepin-4-yl)-4-(2-methoxyphenyl)-5,6-dimethylpyridin-2-yl)quinoline (10m)

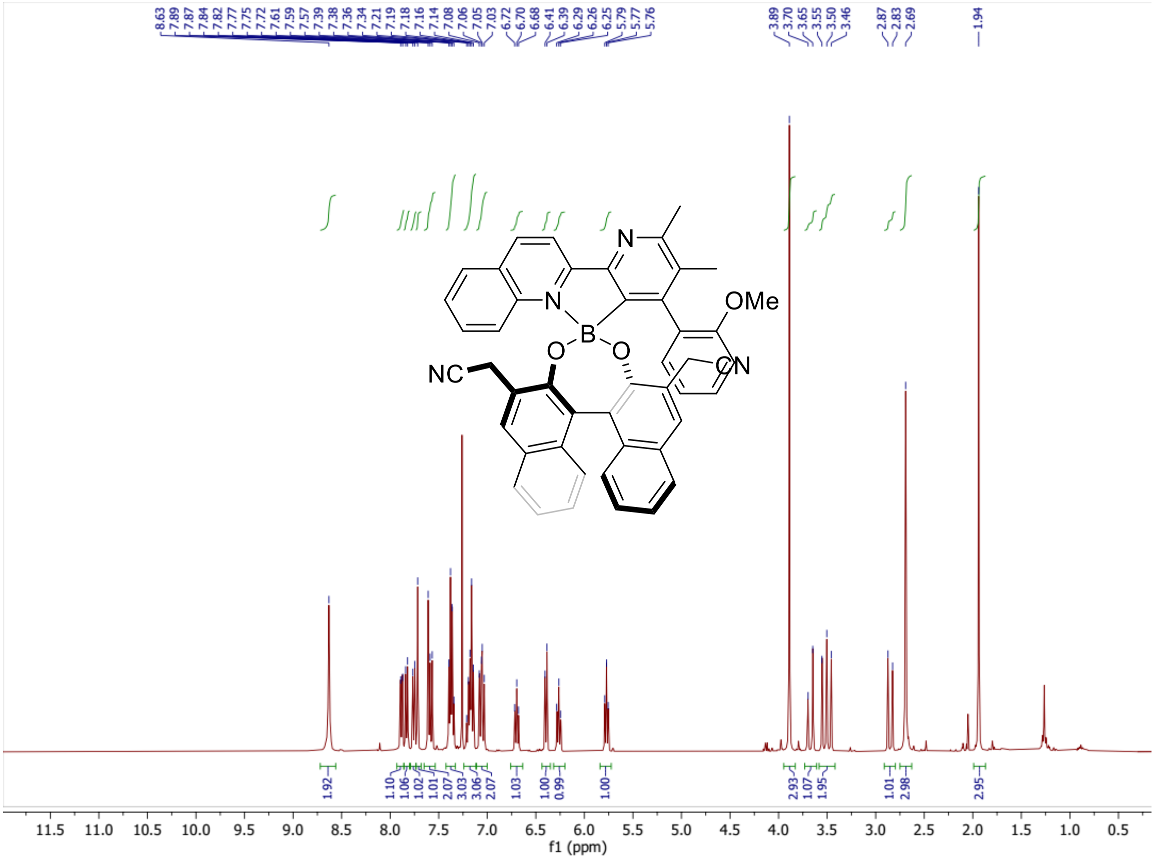

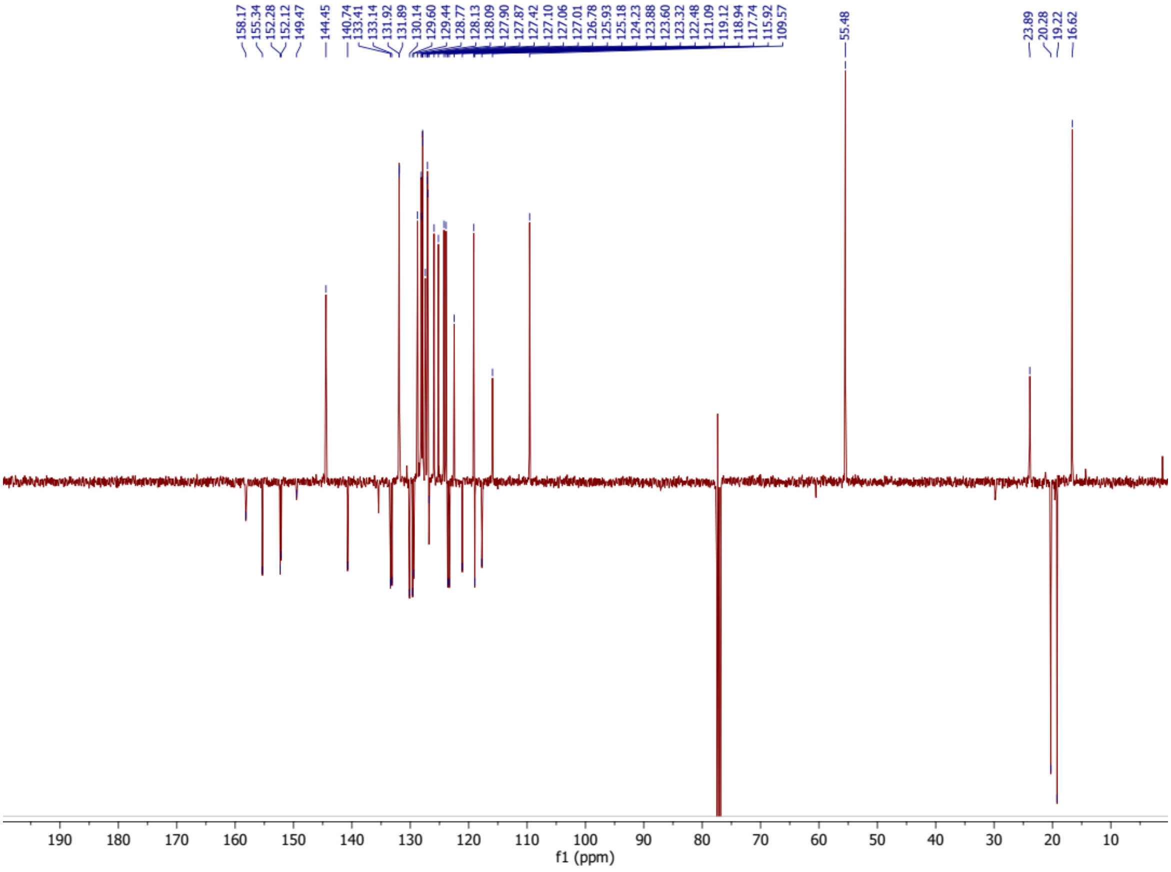

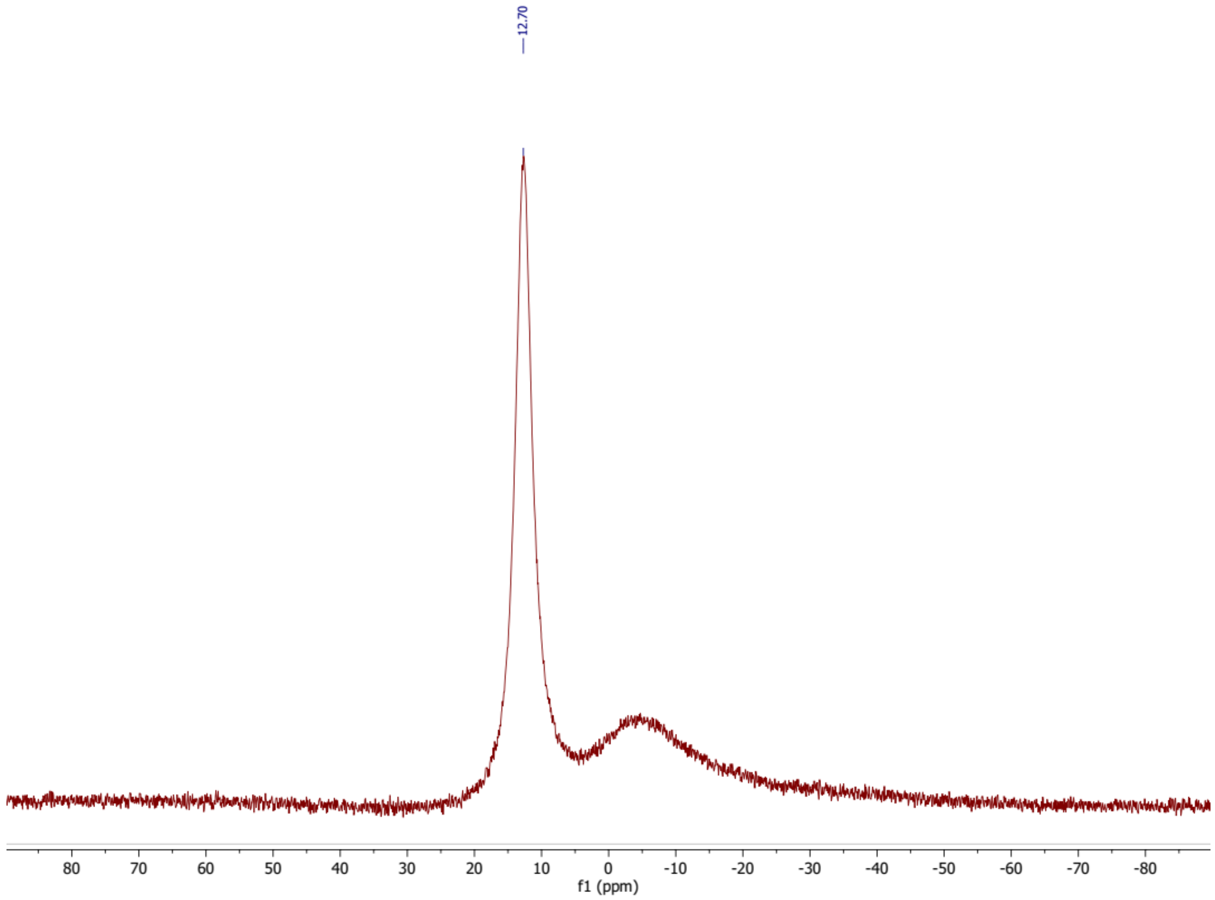
12.70
80
70
60
50
40
30
20
10
0
-10
-20
-30
-40
-50
-60
-70
-80
f1 (ppm)

^{1}H, ^{13}C, and ^{11}B NMR spectra of 2-(3-((R)-Dinaphtho[2,1-d:1',2;-f][1,3,2]dioxaborepin-4-yl)-4-(2-isopropoxyphenyl)-5,6-dimethylpyridin-2-yl)quinolone (10n)

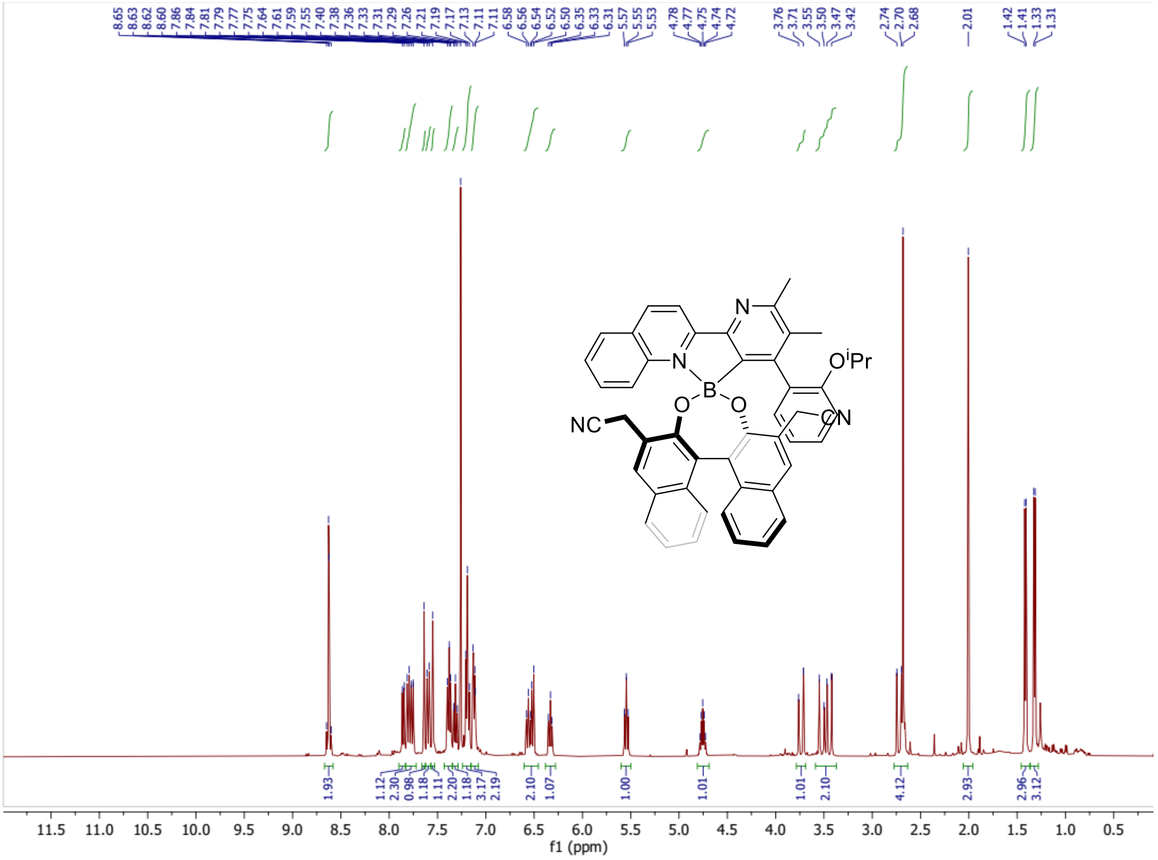

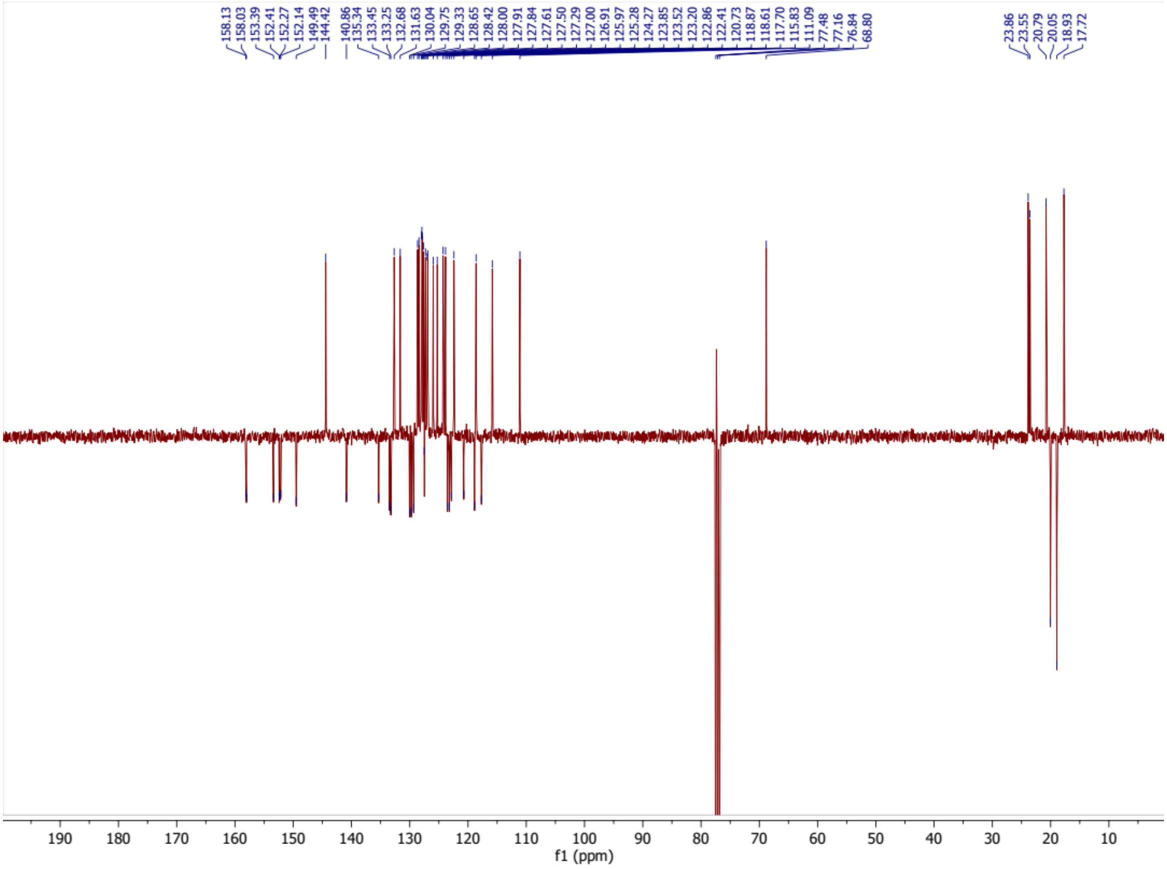

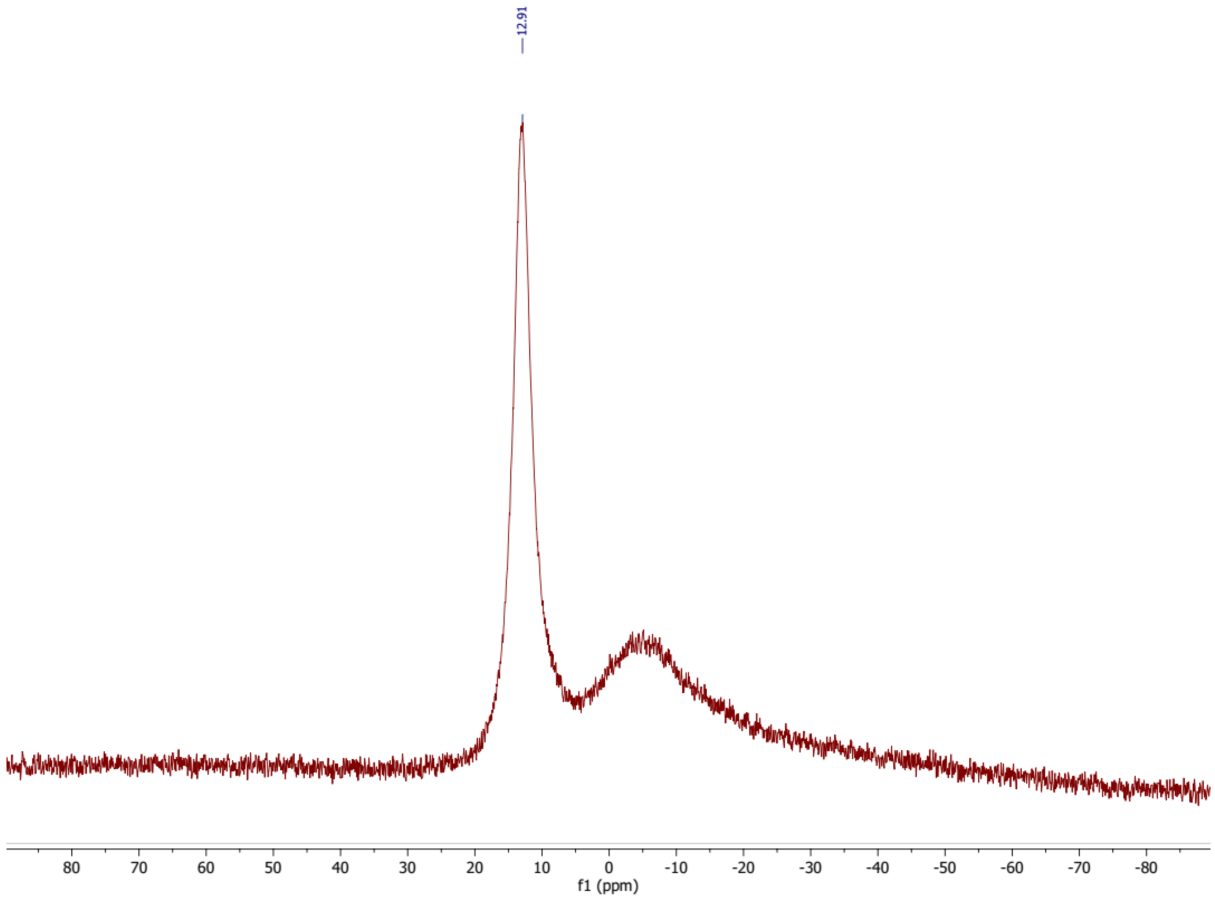
12.91
80
70
60
50
40
30
20
10
0
-10
-20
-30
-40
-50
-60
-70
-80
f1 (ppm)

^{1}H, ^{13}C, and ^{11}B NMR spectra of 1-(3-((R)-dinaphtho[2,1-d:1',2'-f][1,3,2]dioxaborepin-4-yl)-4-(2-methoxyphenyl)-5,6-diphenylpyridin-2-yl)isoquinoline (10o)

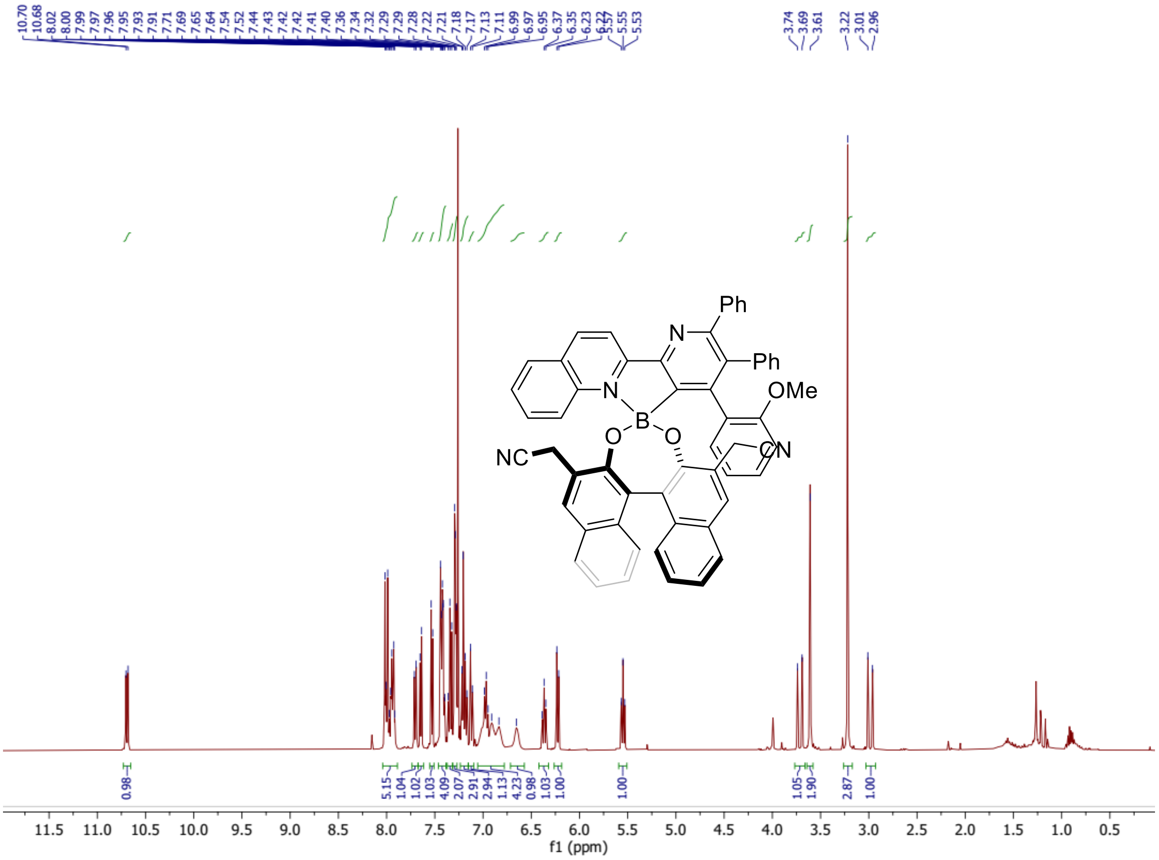

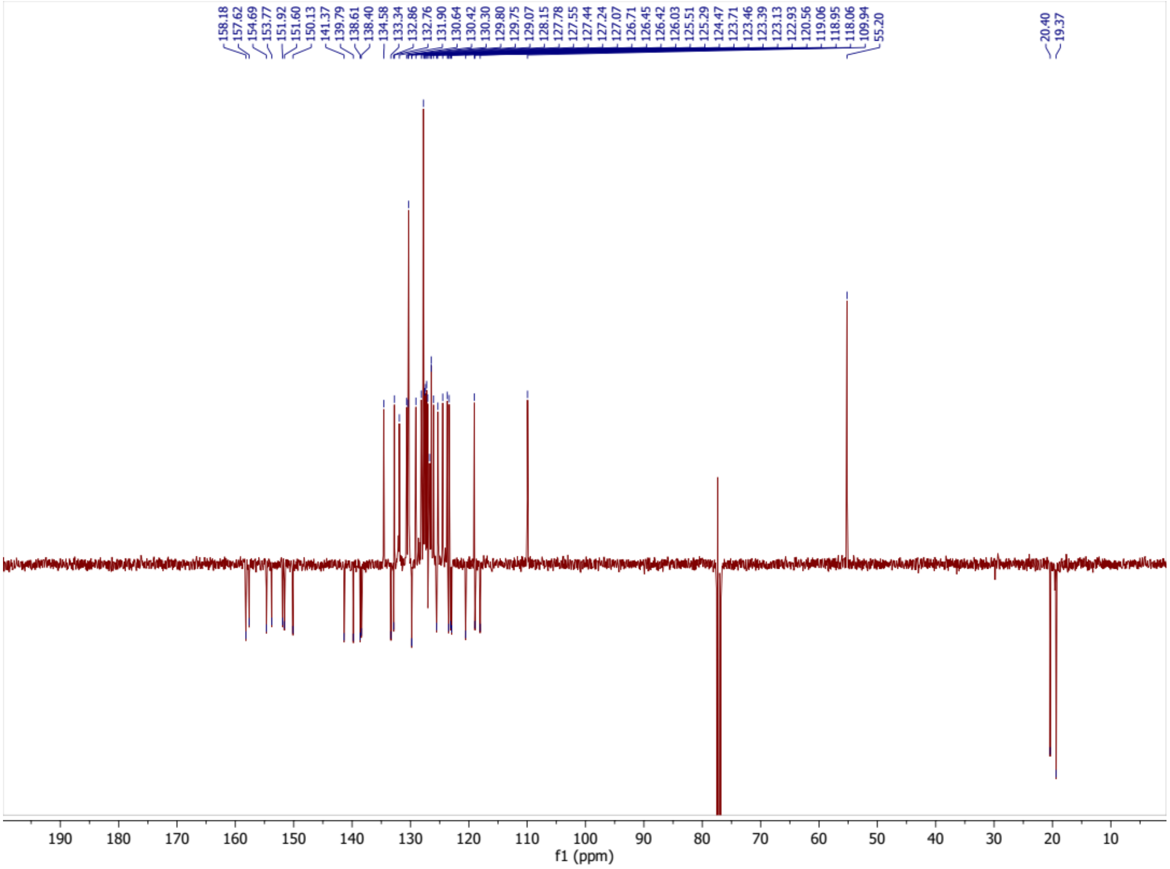

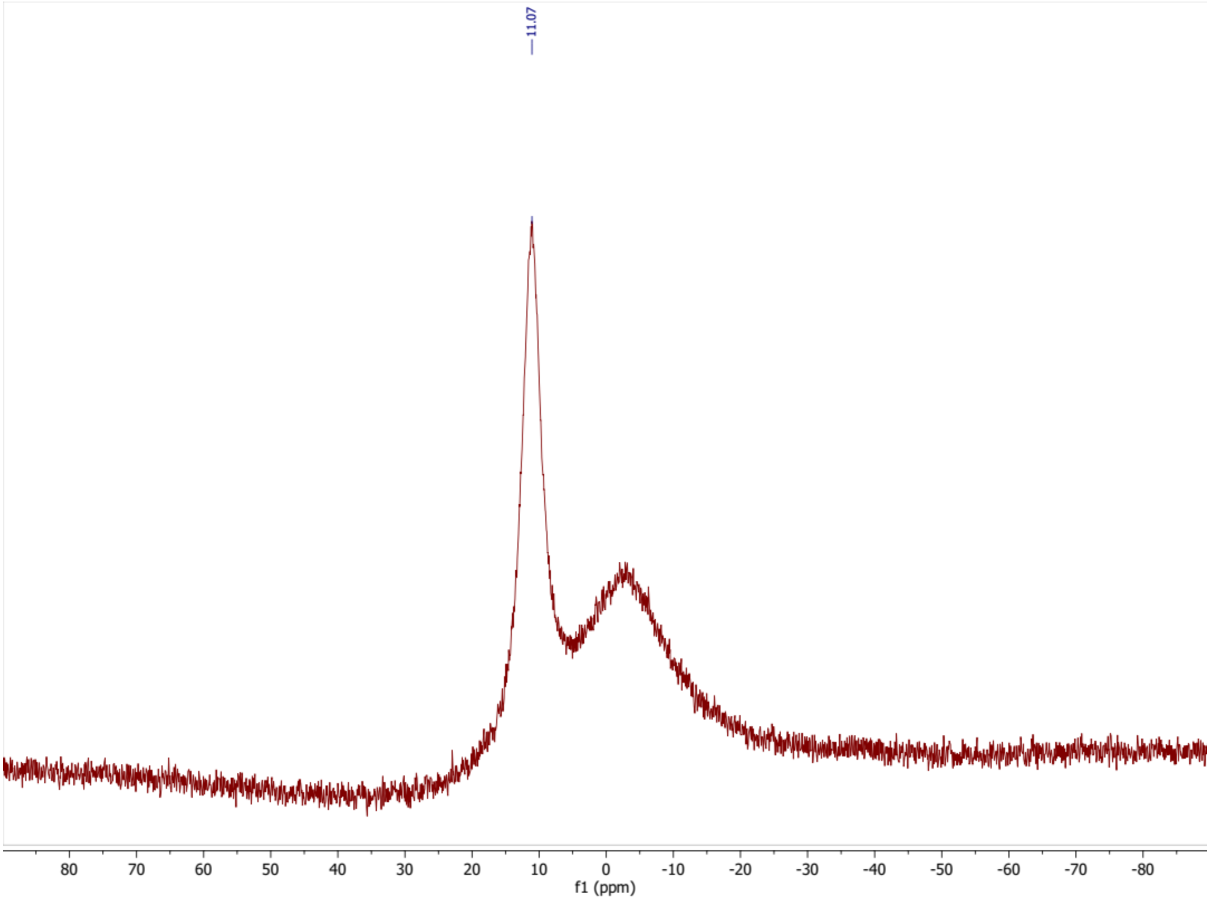

11.07
80
70
60
50
40
30
20
10
0
-10
-20
-30
-40
-50
-60
-70
-80
f1 (ppm)

^{1}H and ^{13}C spectra of 4-(2-methoxyphenyl)-5,6-dimethyl-2-(quinolin-2-yl)pyridin-3-ol (11)

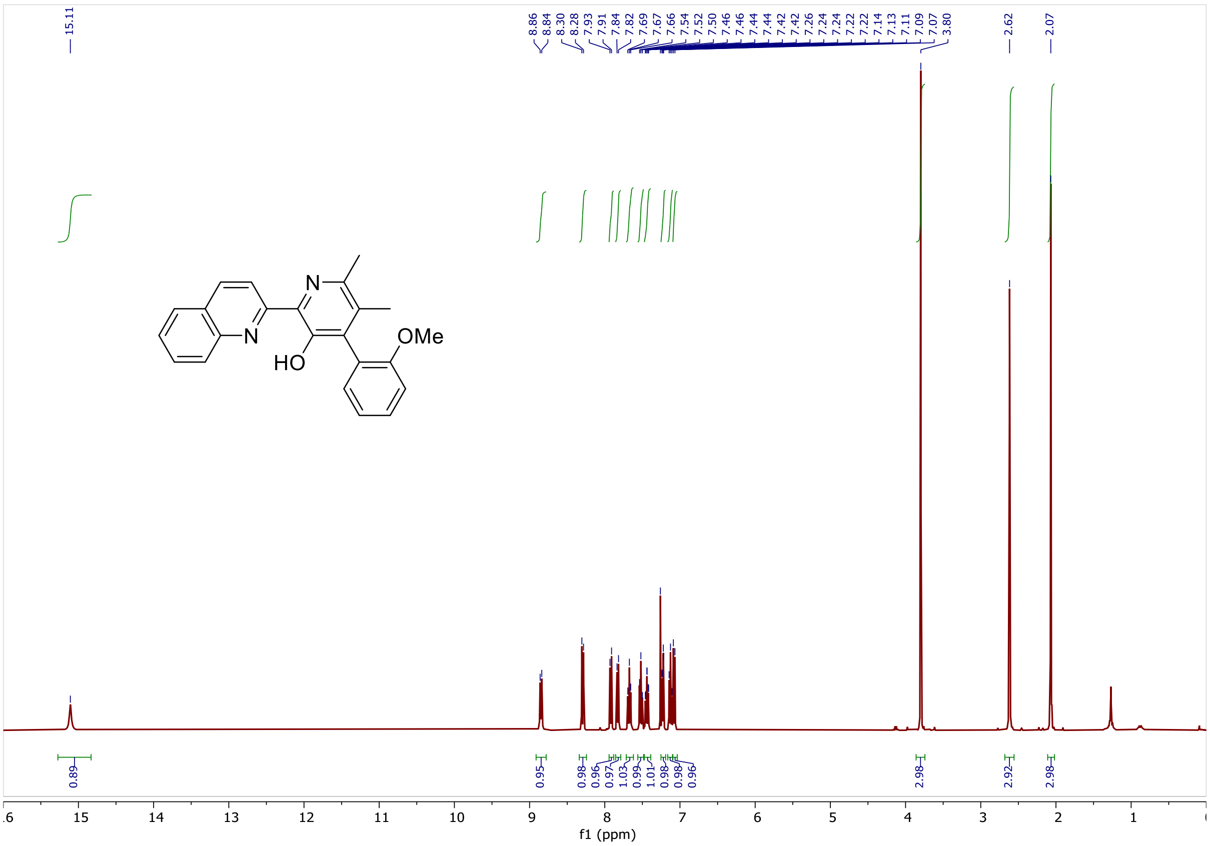

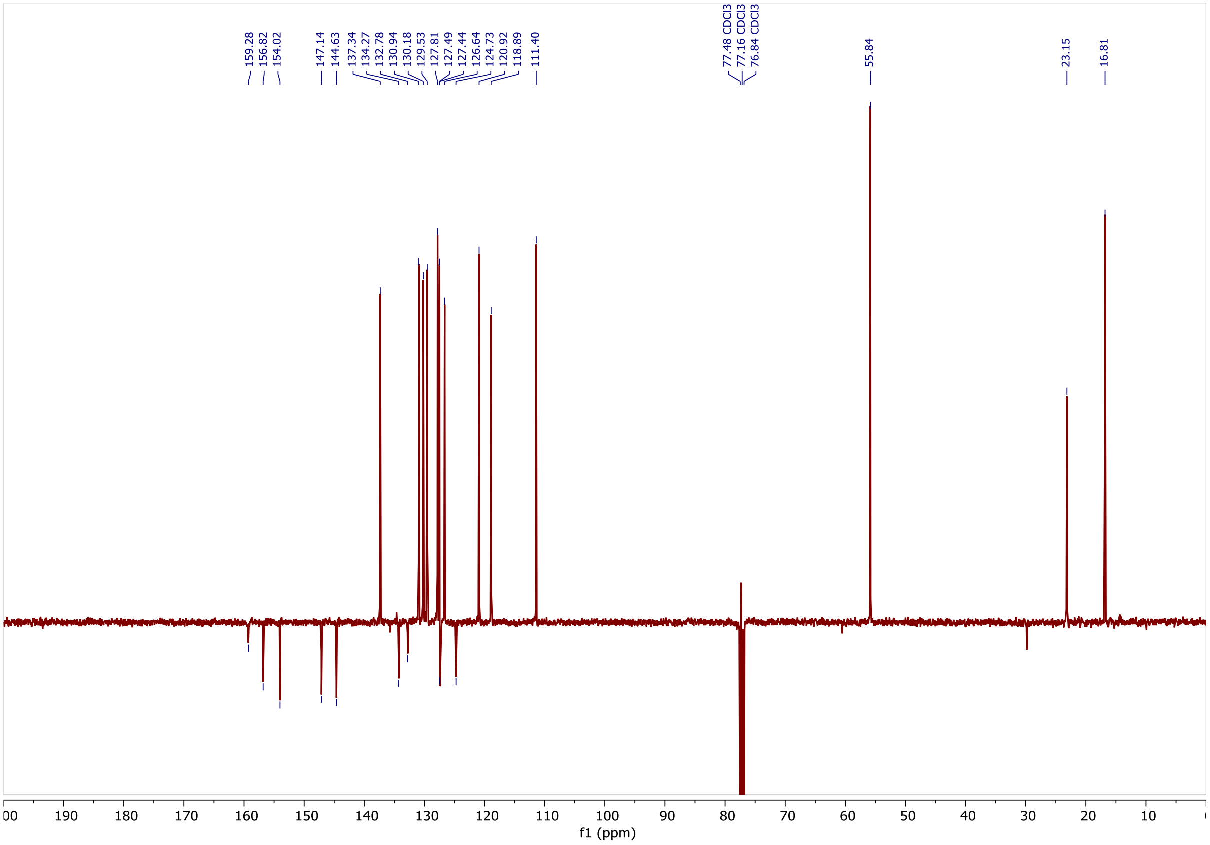

^{1}H and ^{13}C spectra of 4-(2-isopropoxyphenyl)-5,6-dimethyl-2-(quinolin-2-yl)pyridin-3-ol (12)

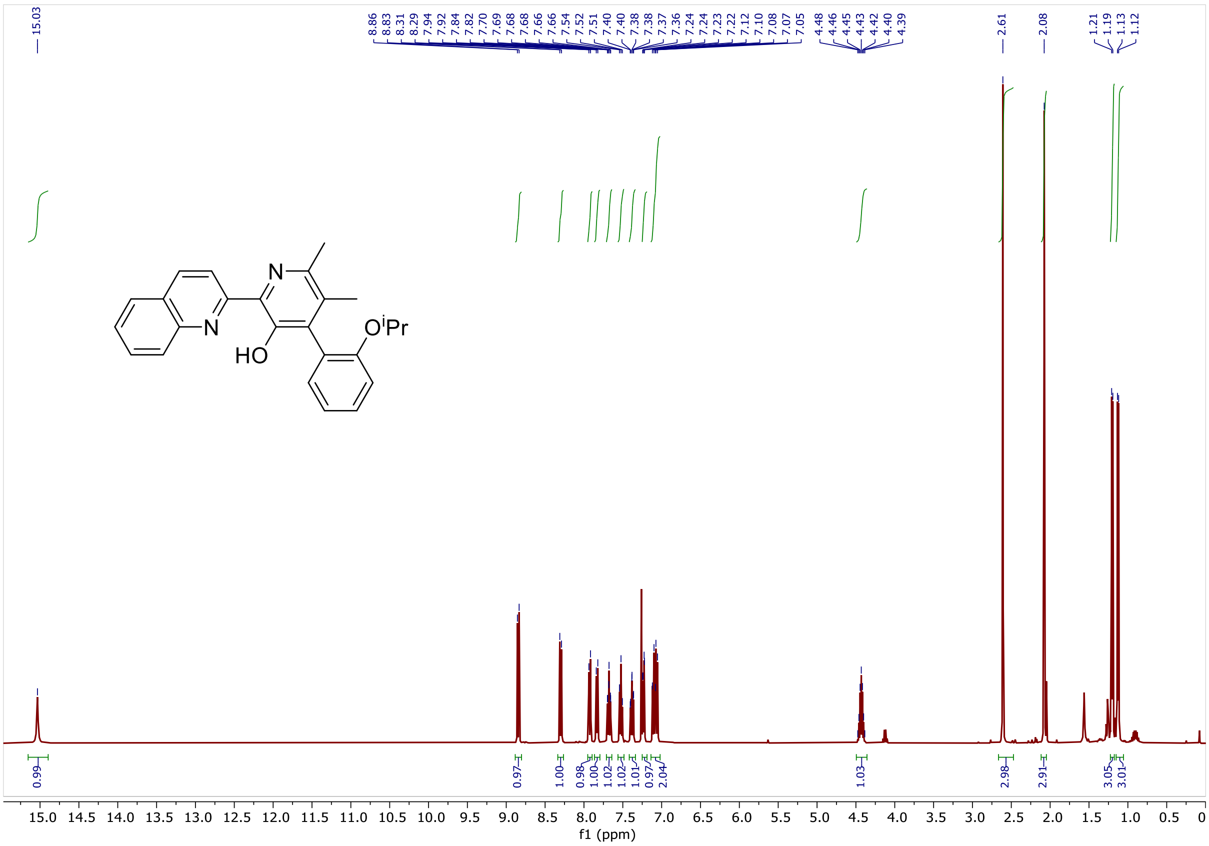

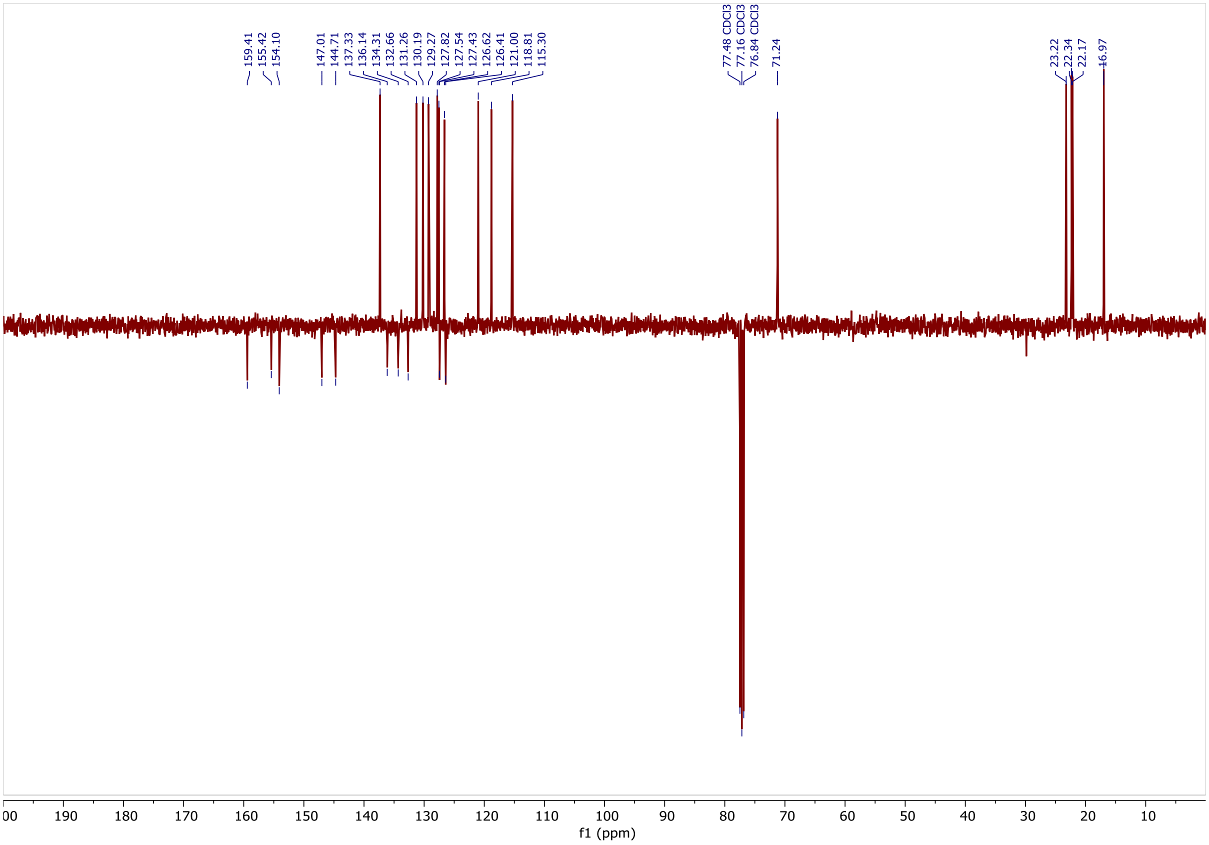

^{1}H and ^{13}C spectra of 5,6-Dimethyl-4-(2-(methylsulfonyl)phenyl)-2-(quinolin-2-yl)pyridin-3-ol (13)

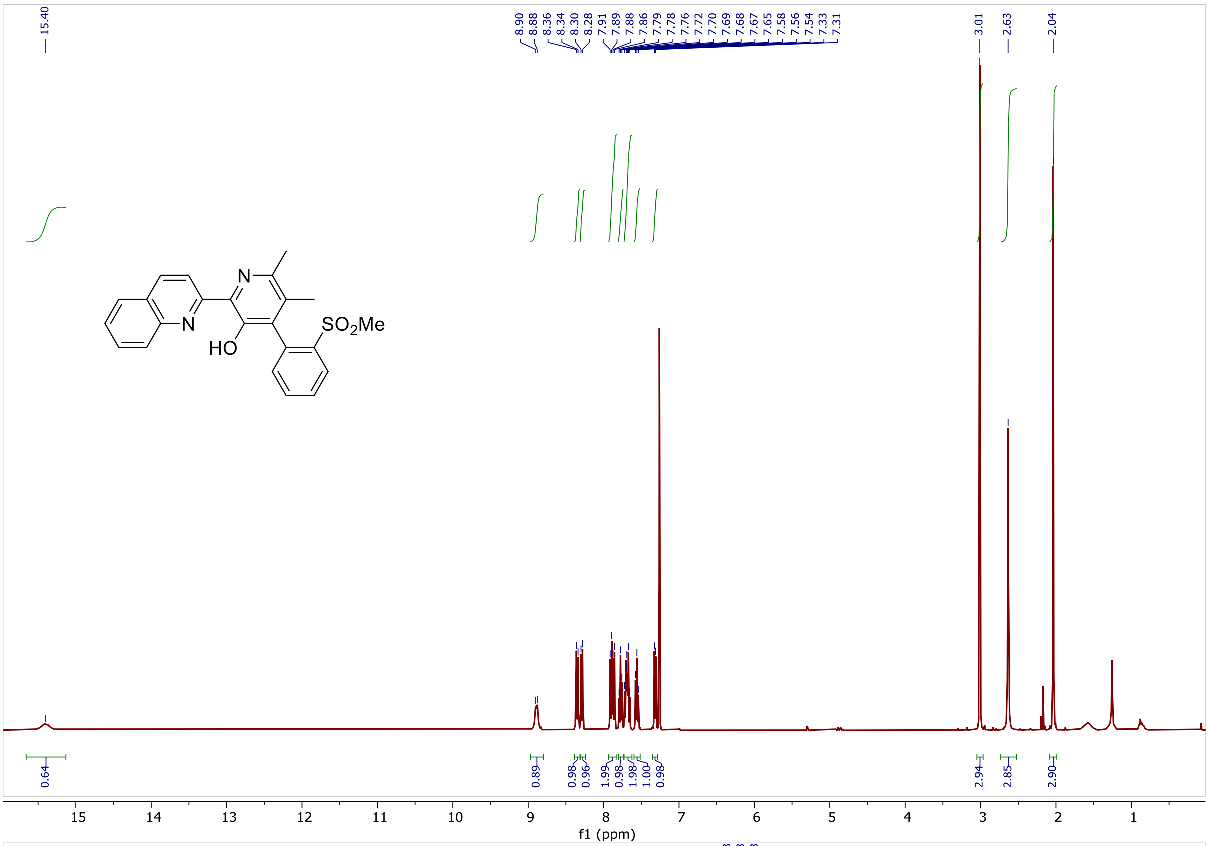

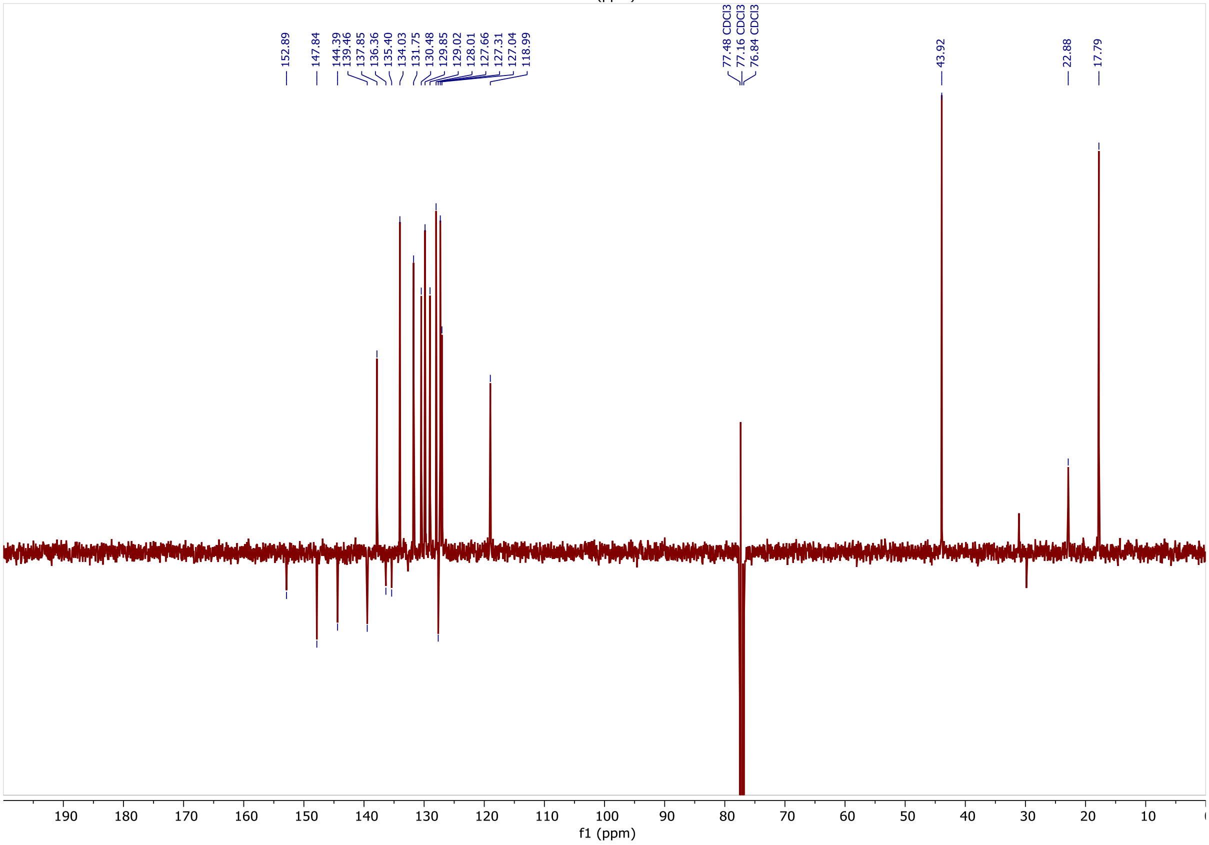

^{1}H and ^{13}C spectra of 6-iodo-2,3-dimethoxyphenol

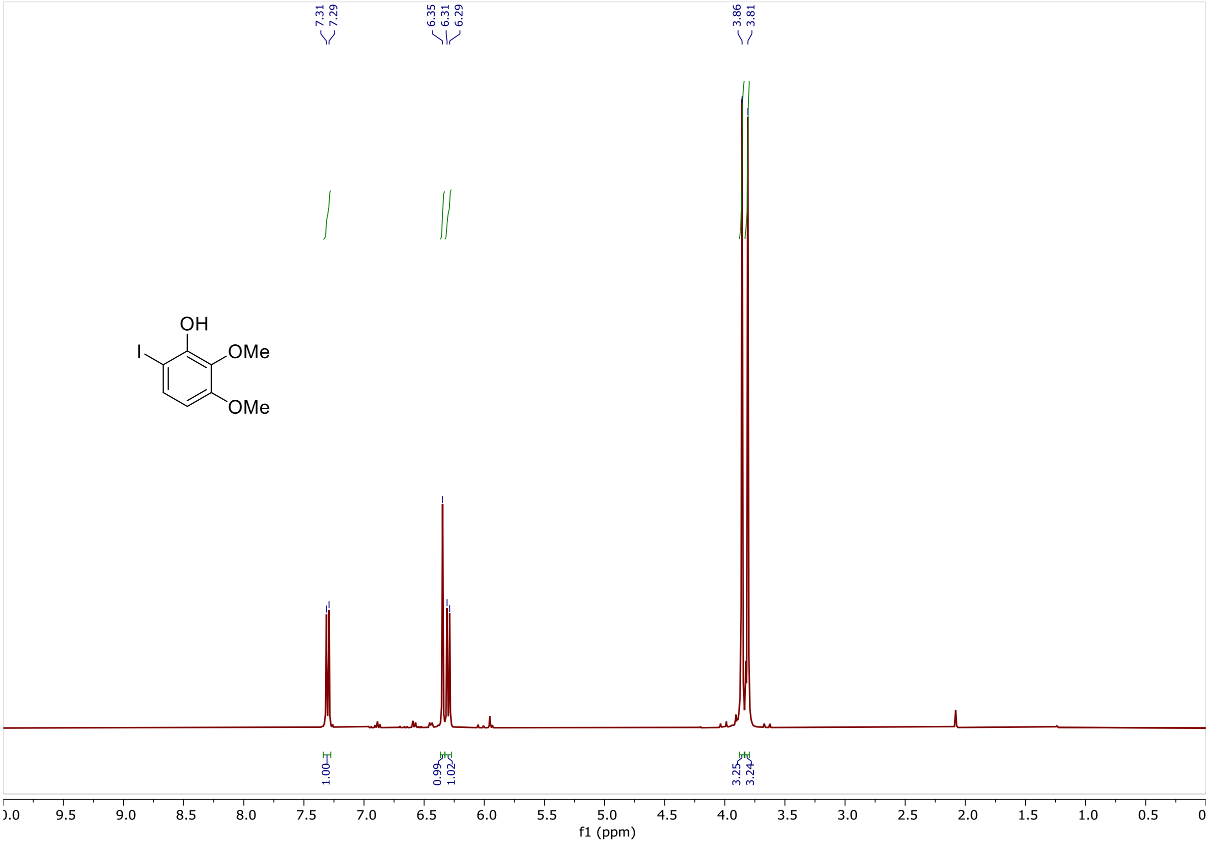

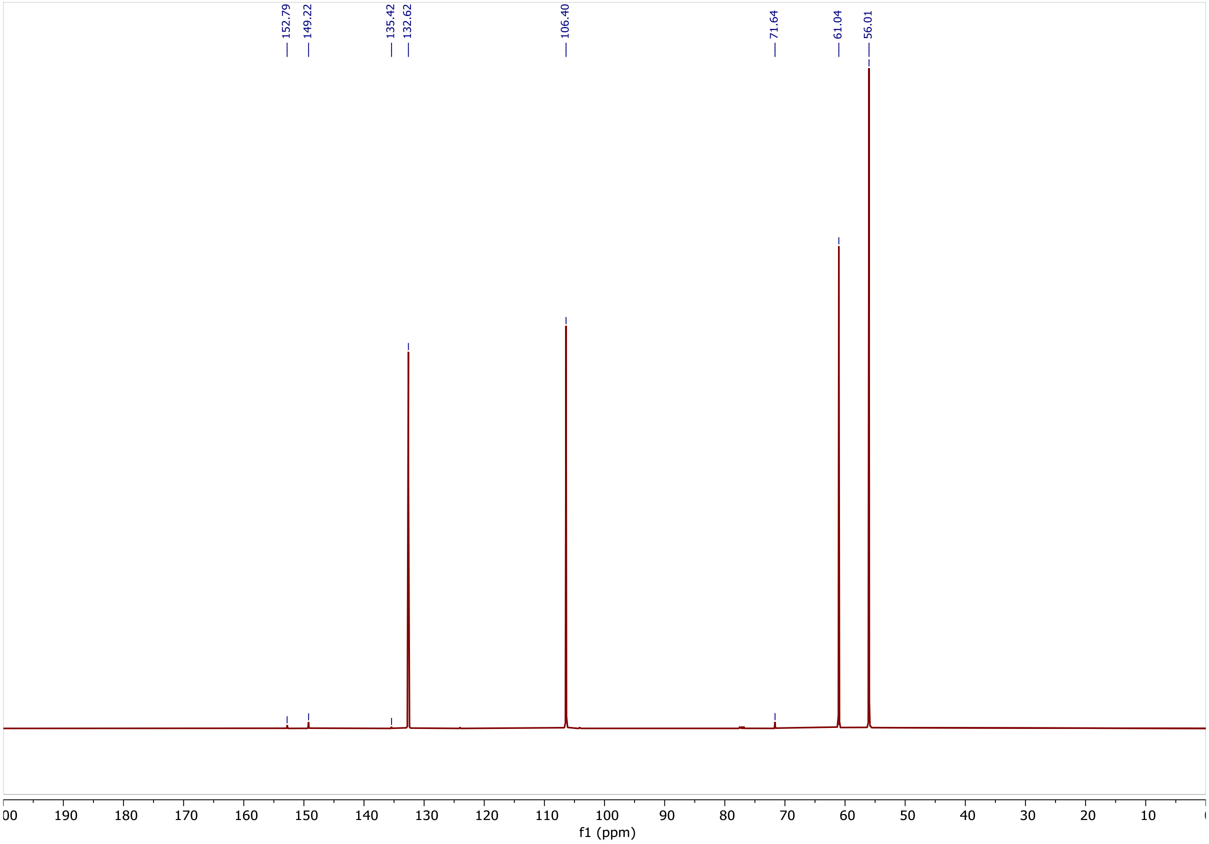

^1H and ^{13}C spectra of 2-benzyloxy-1-iodo-2,3-dimethoxybenzene

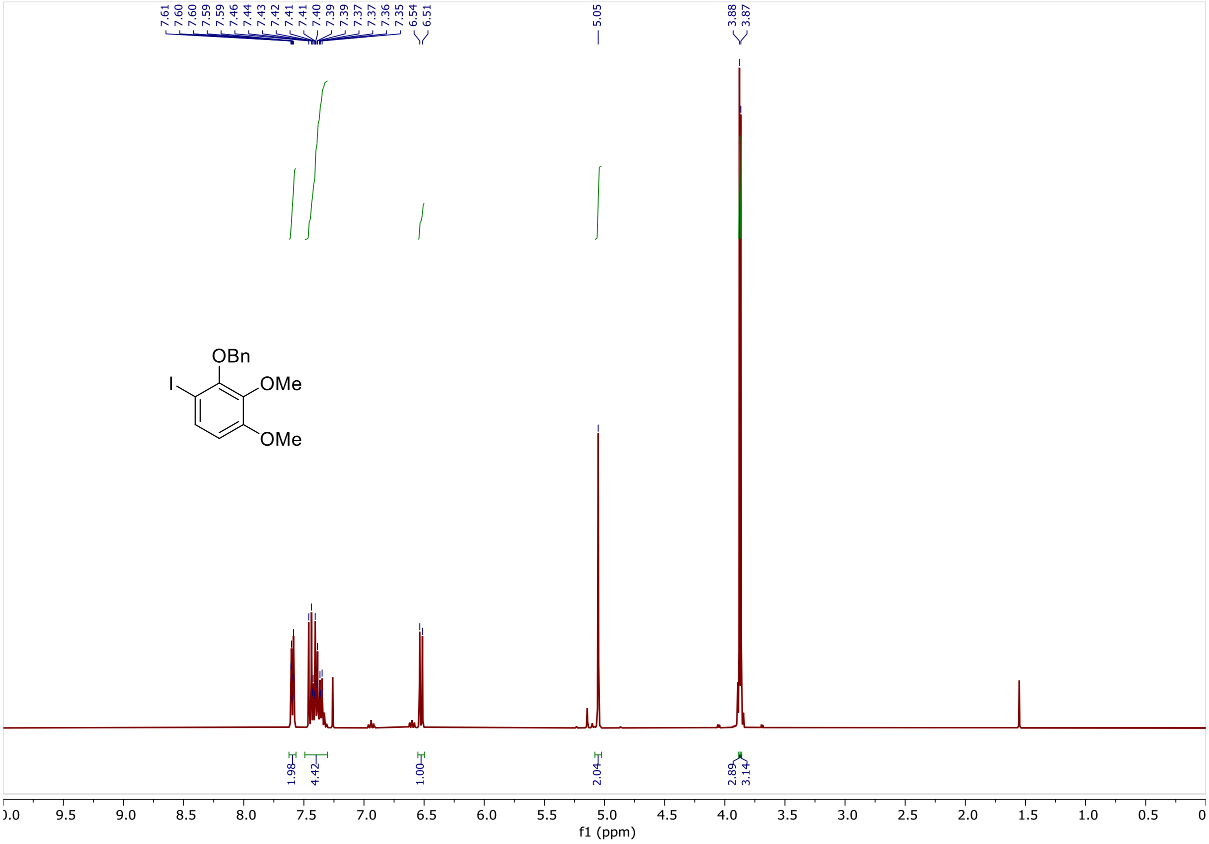

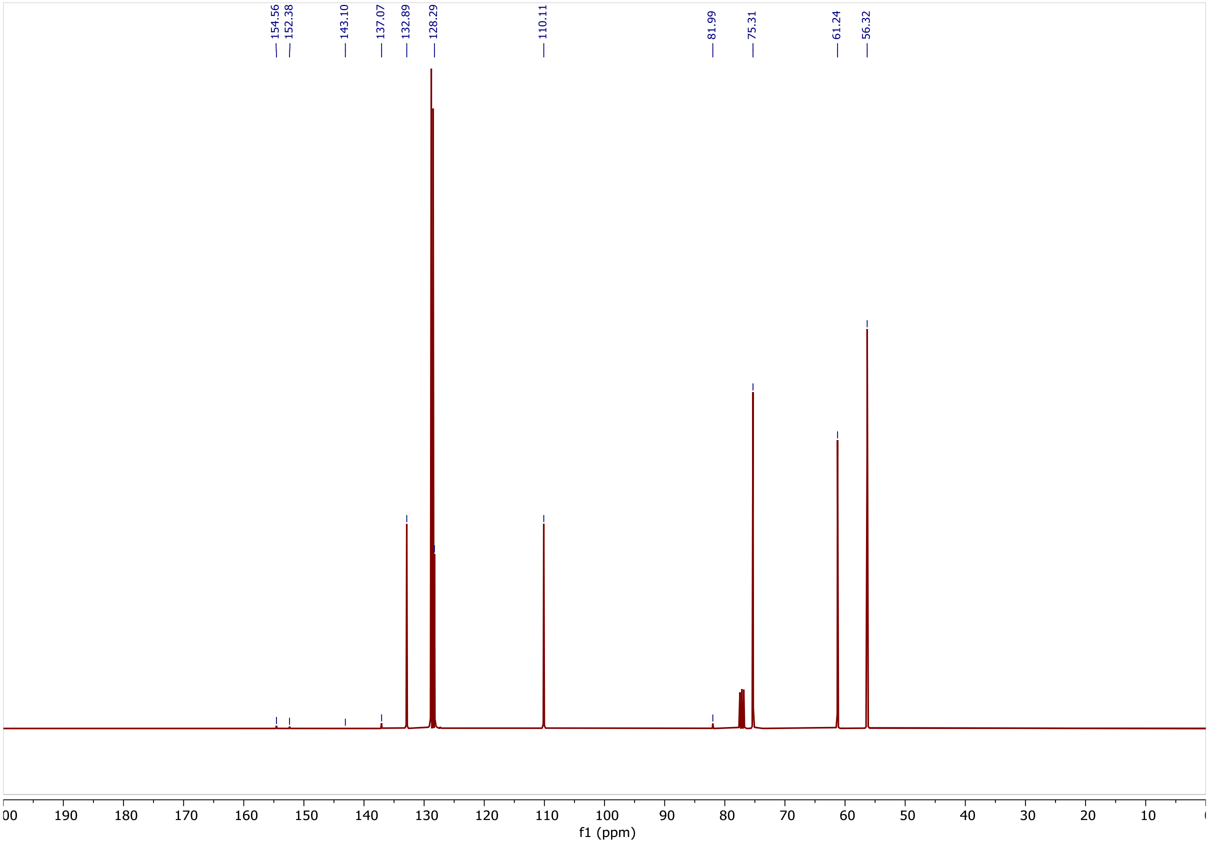

^{1}H and ^{13}C spectra of (2-benzyloxy-2,3-dimethoxyphenylethynyl)trimethylsilane

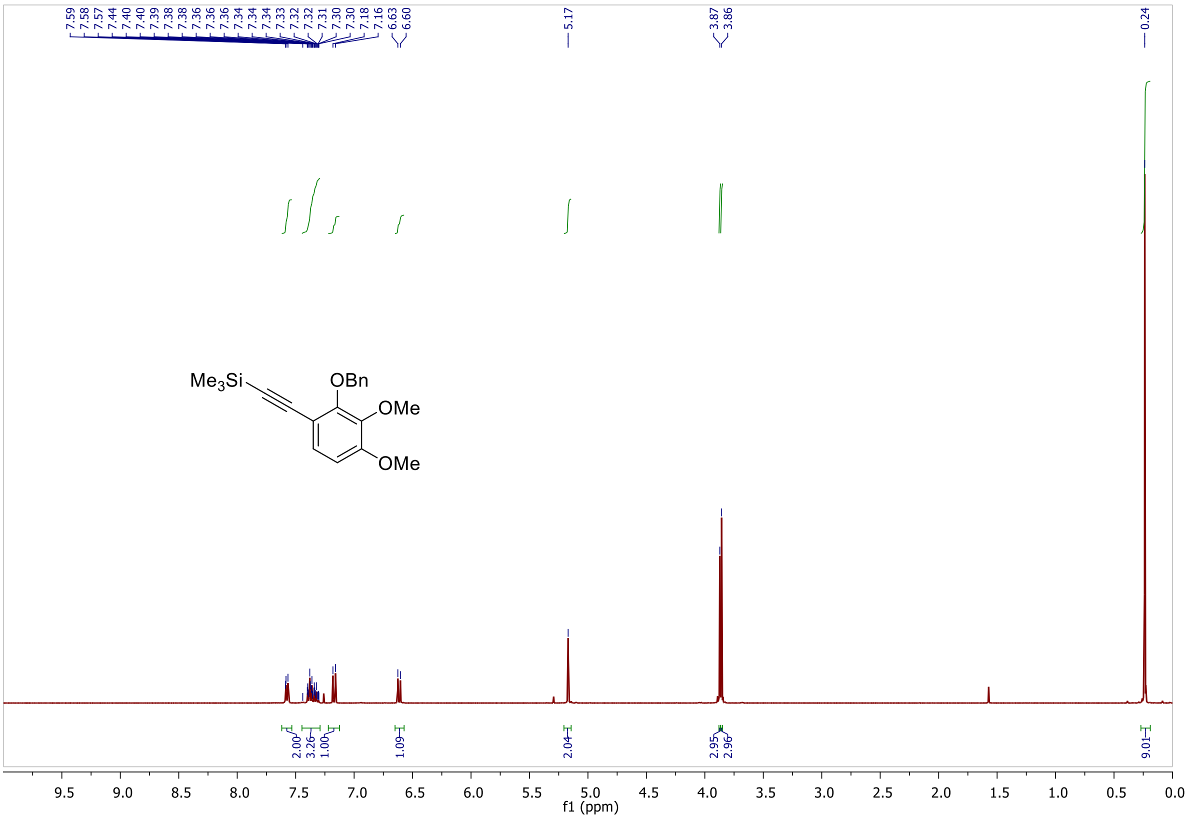

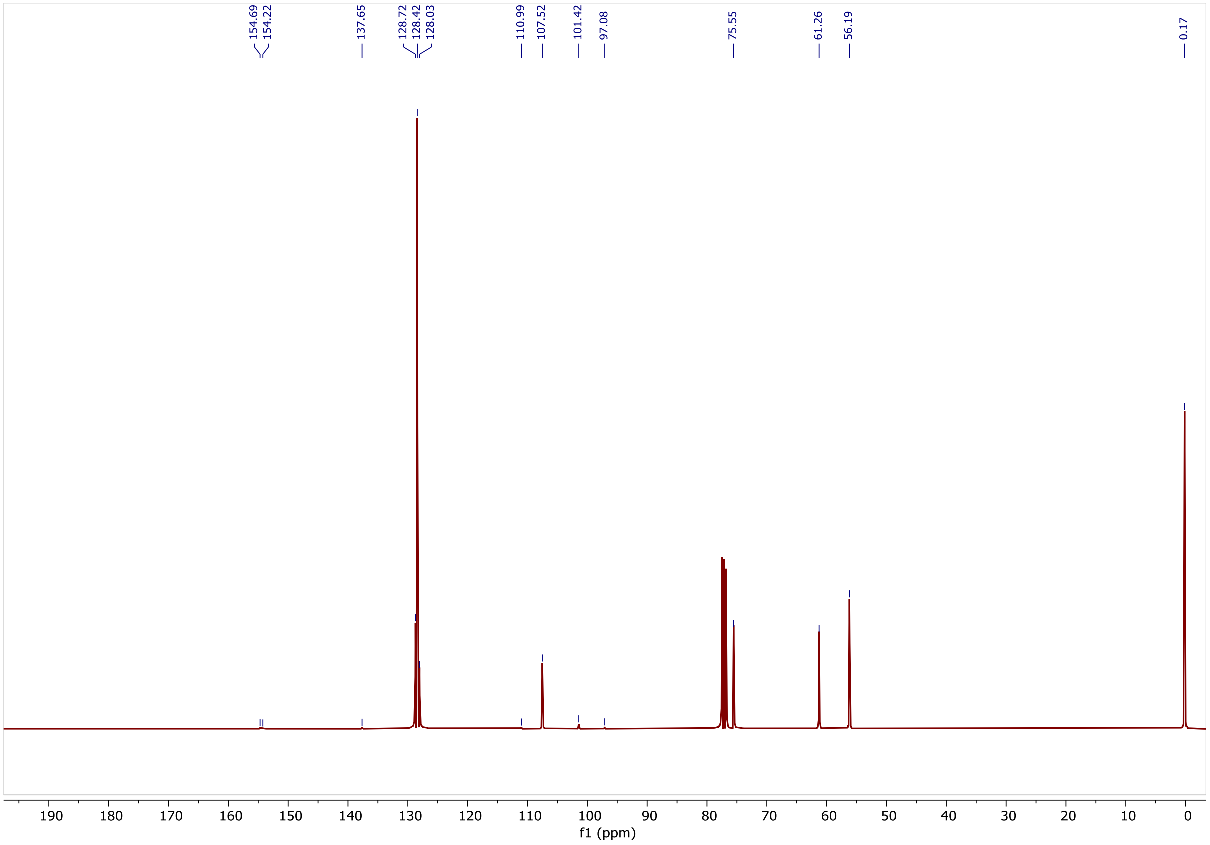

^{1}H and ^{13}C spectra of 2-benzyloxy-1-ethynyl-2,3-dimethoxybenzene

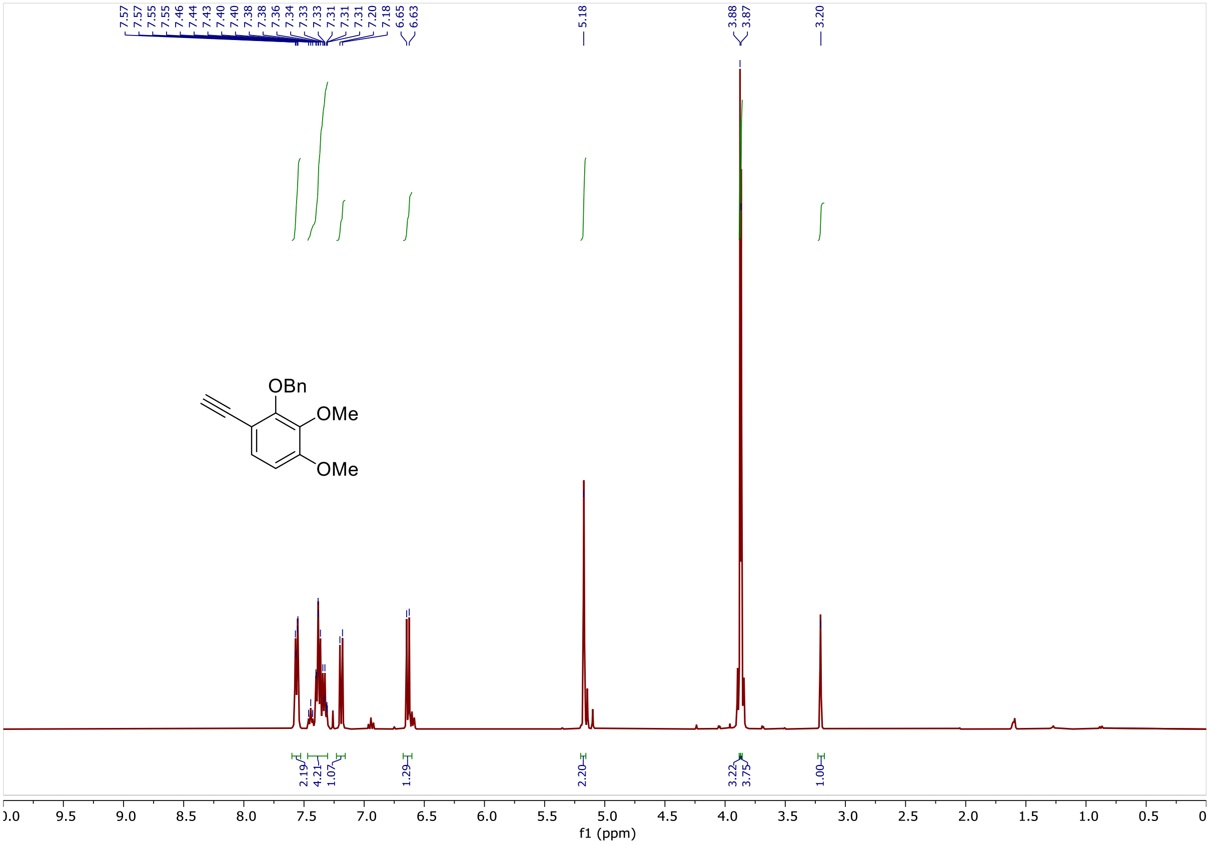

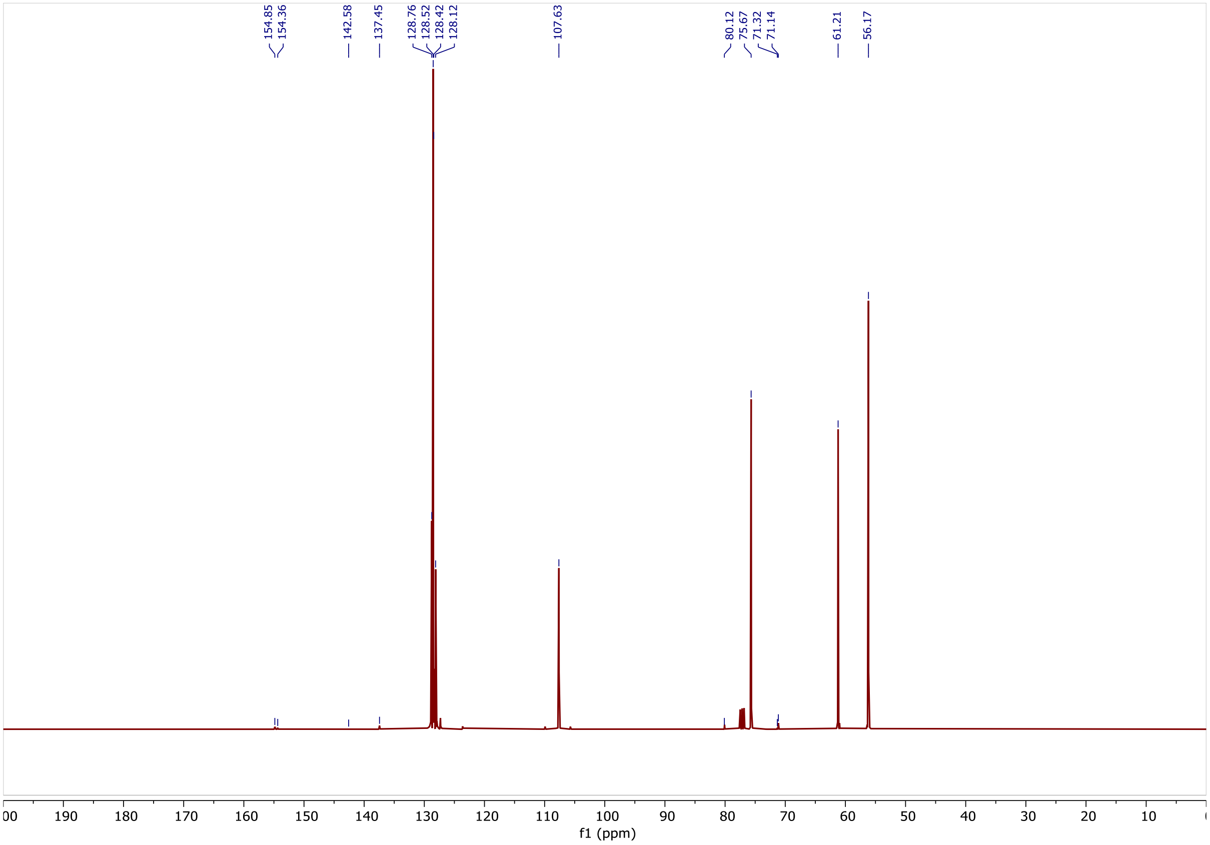

^{1}H, ^{13}C, ^{11}B, and ^{19}F NMR spectra of potassium ((2-(benzyloxy)-3,4-dimethoxyphenyl)ethynyl)trifluoroborate

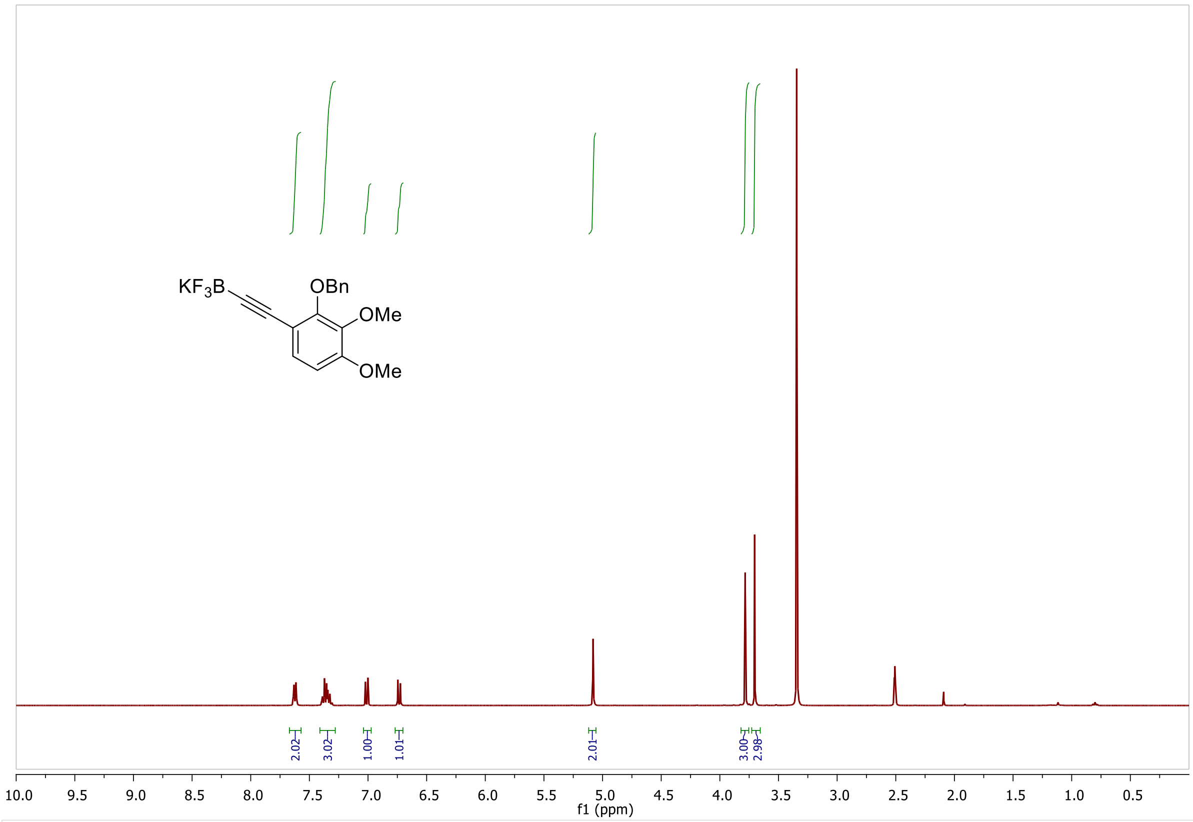

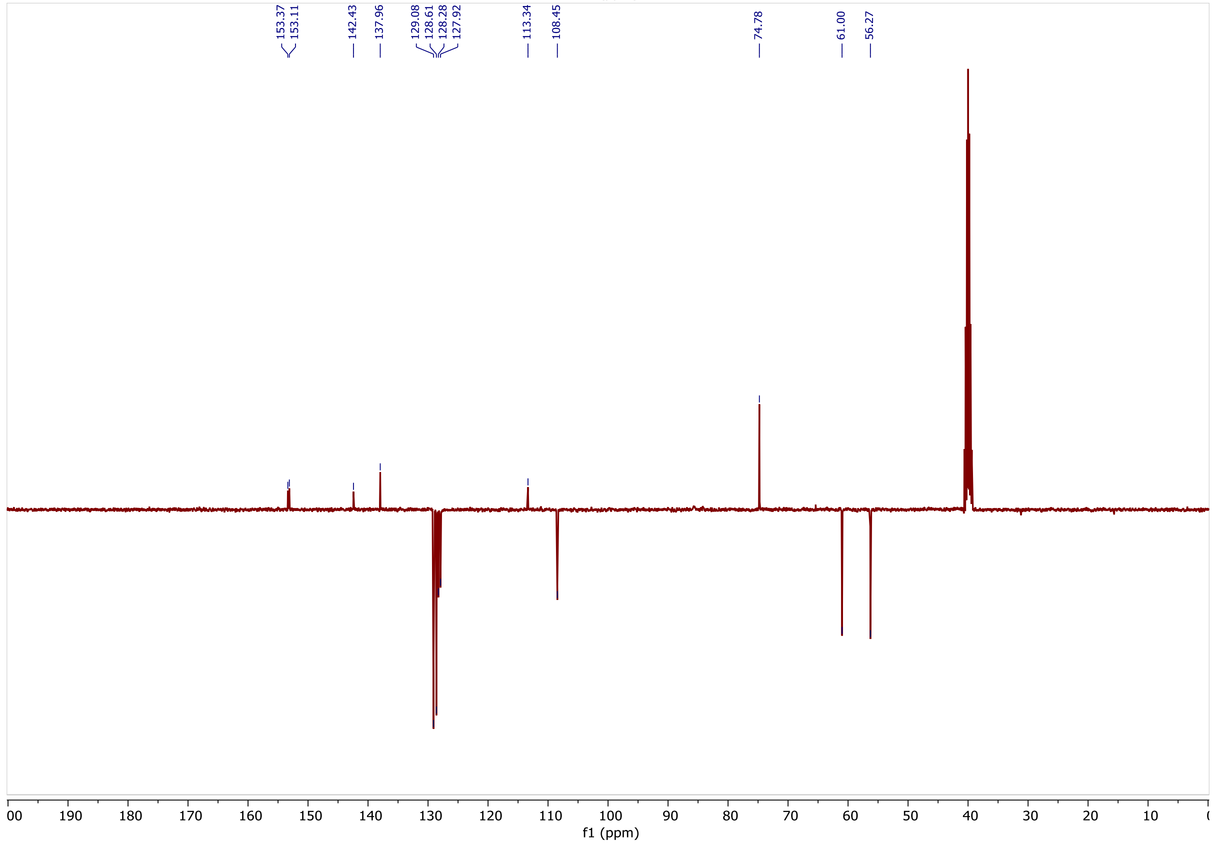

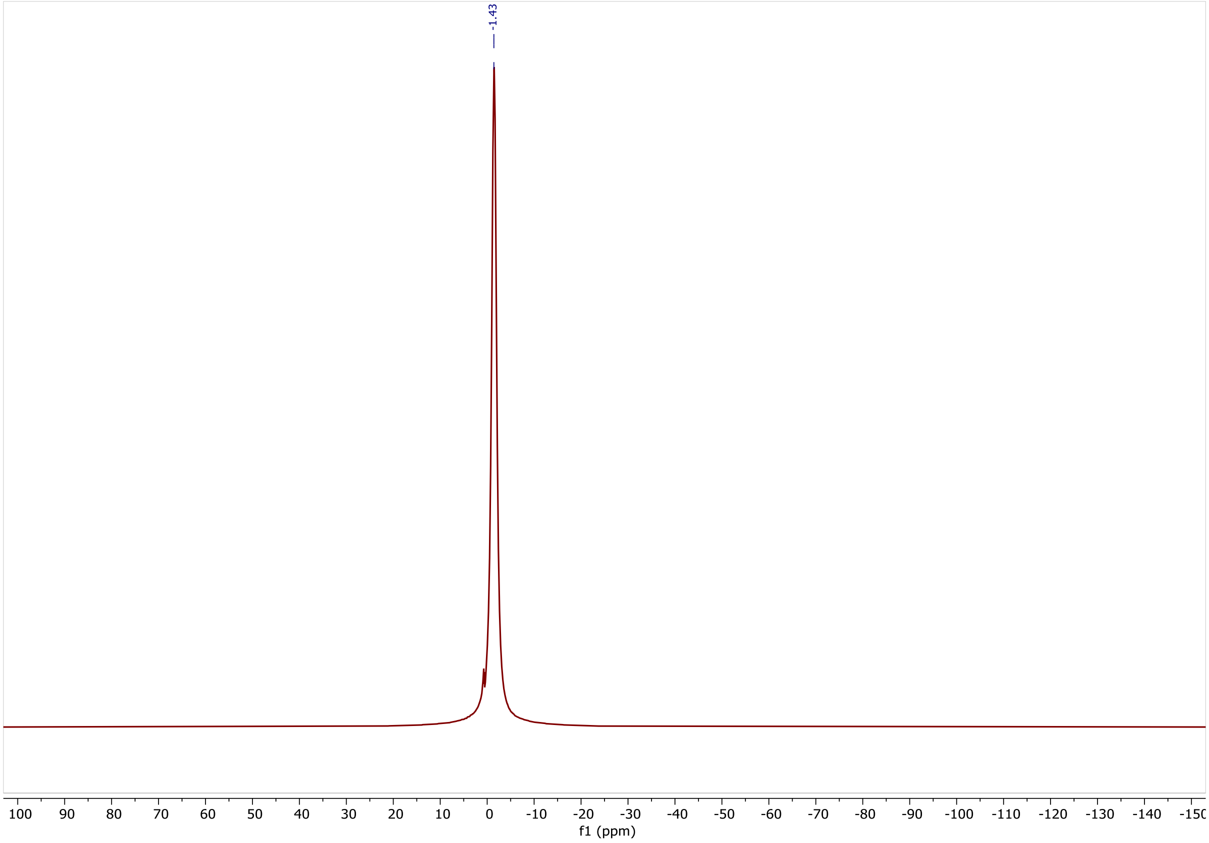
-1.43
100
90
80
70
60
50
40
30
20
10
0
-10
-20
-30
-40
-50
-60
-70
-80
-90
-100
-110
-120
-130
-140
f1 (ppm)

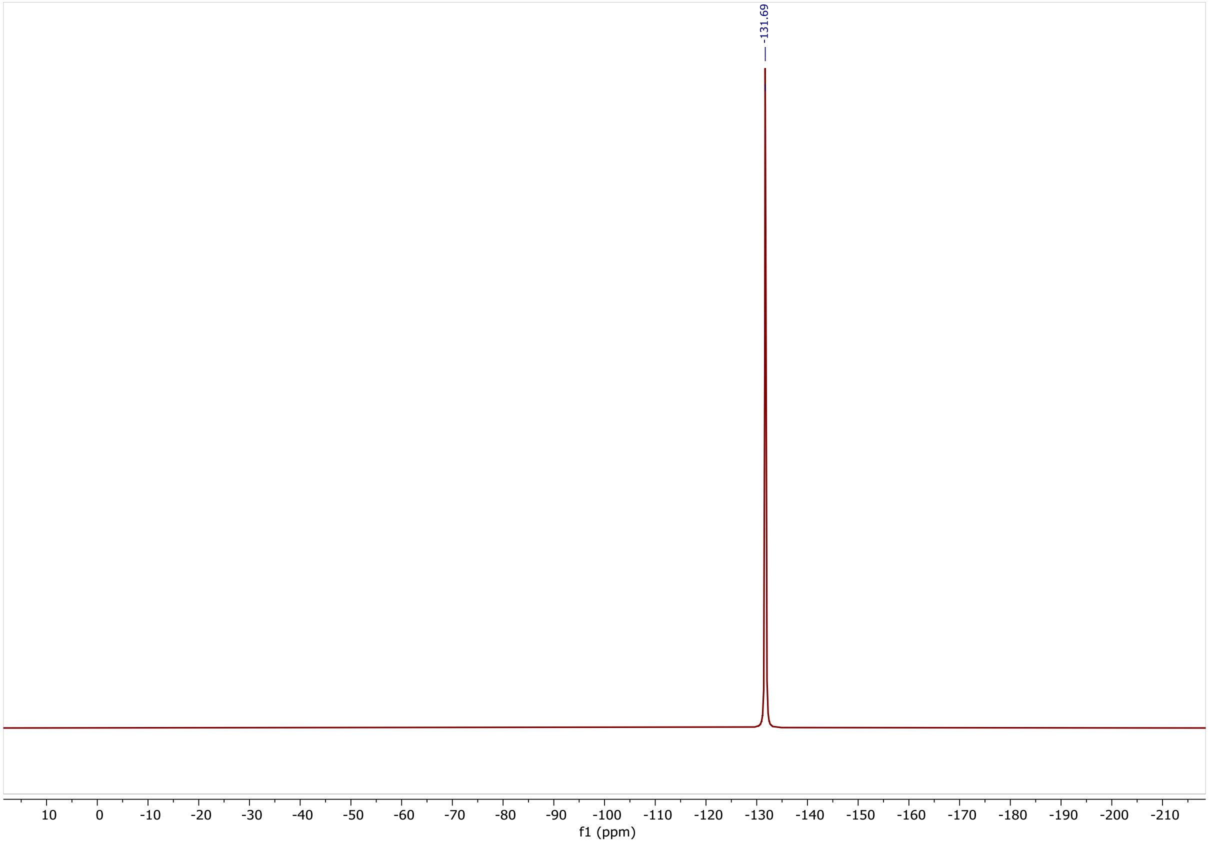
-131.69
10
0
-10
-20
-30
-40
-50
-60
-70
-80
-90
-100
-110
-120
-130
-140
-150
-160
-170
-180
-190
-200
-210
f1 (ppm)

^{1}H and ^{13}C spectra of 6-methoxyquinoline-2-carbohydrazonamide

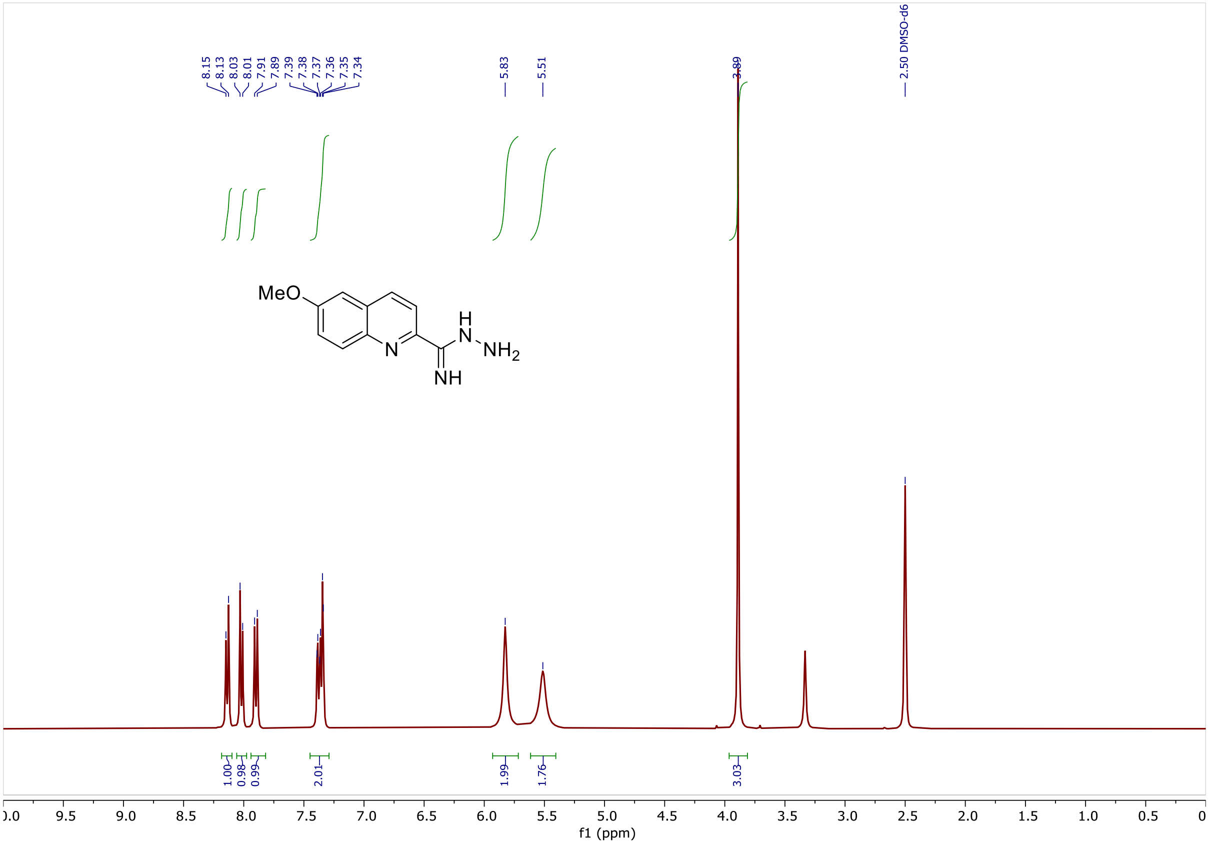

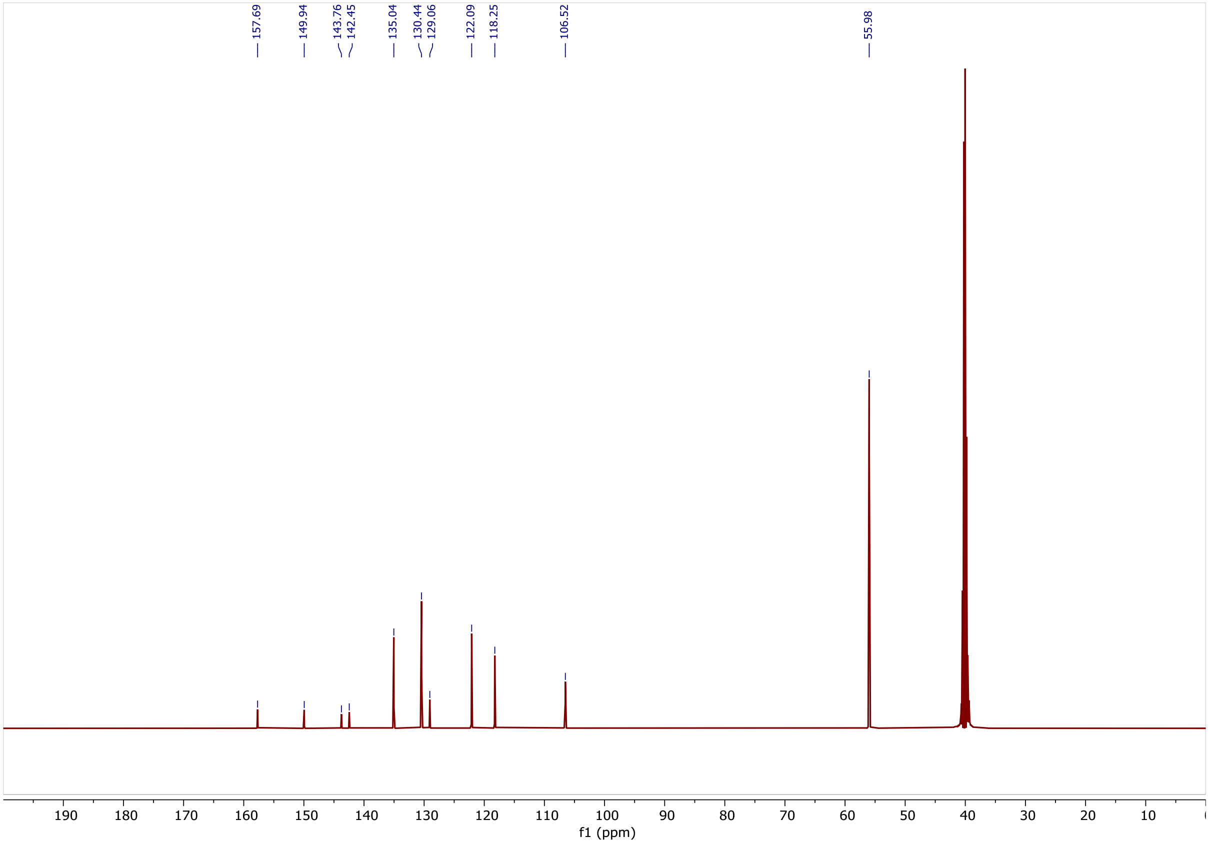

^{1}H and ^{13}C spectra of 3-(6-methoxyquinolin-2-yl)-6-methyl-1,2,4-triazin-5(2H)-one

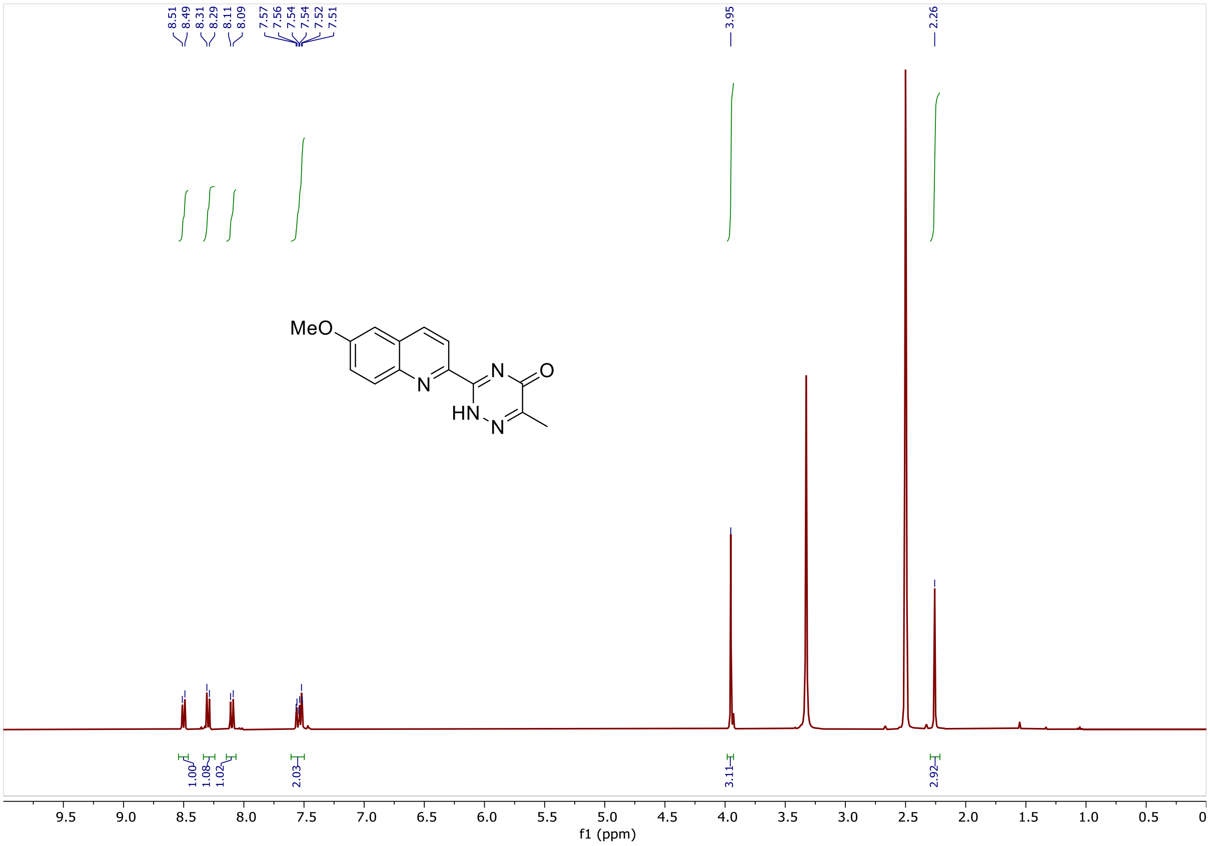

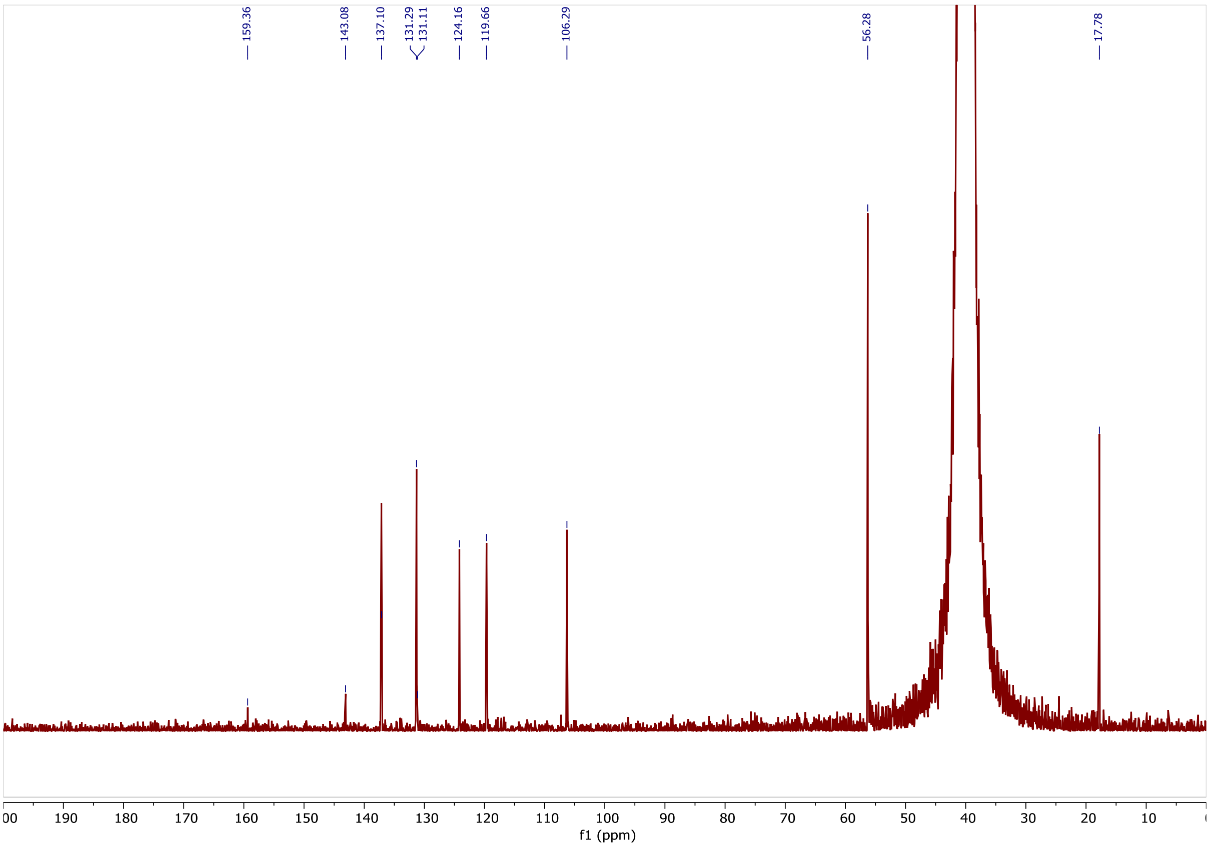

^{1}H and ^{13}C spectra of 2-(5-chloro-6-methyl-1,2,4-triazin-3-yl)-6-methoxyquinoline

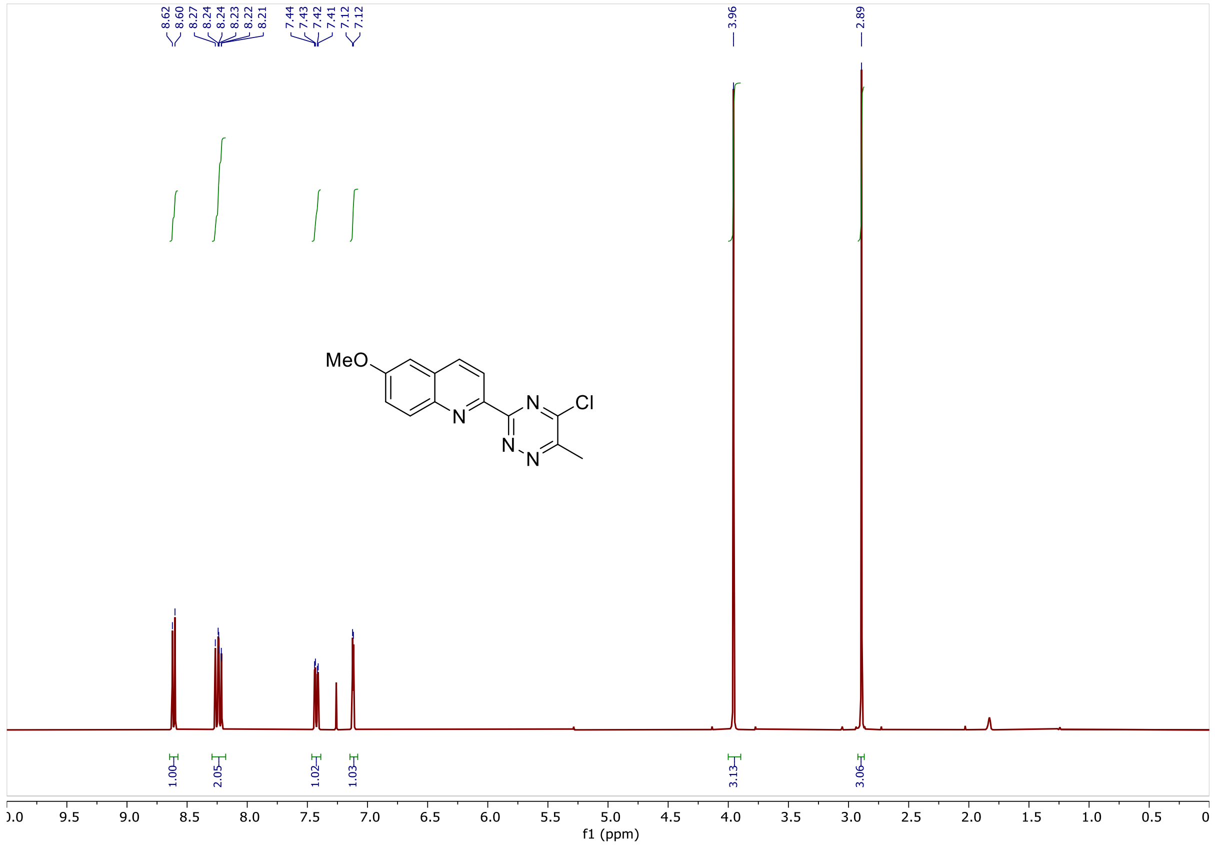

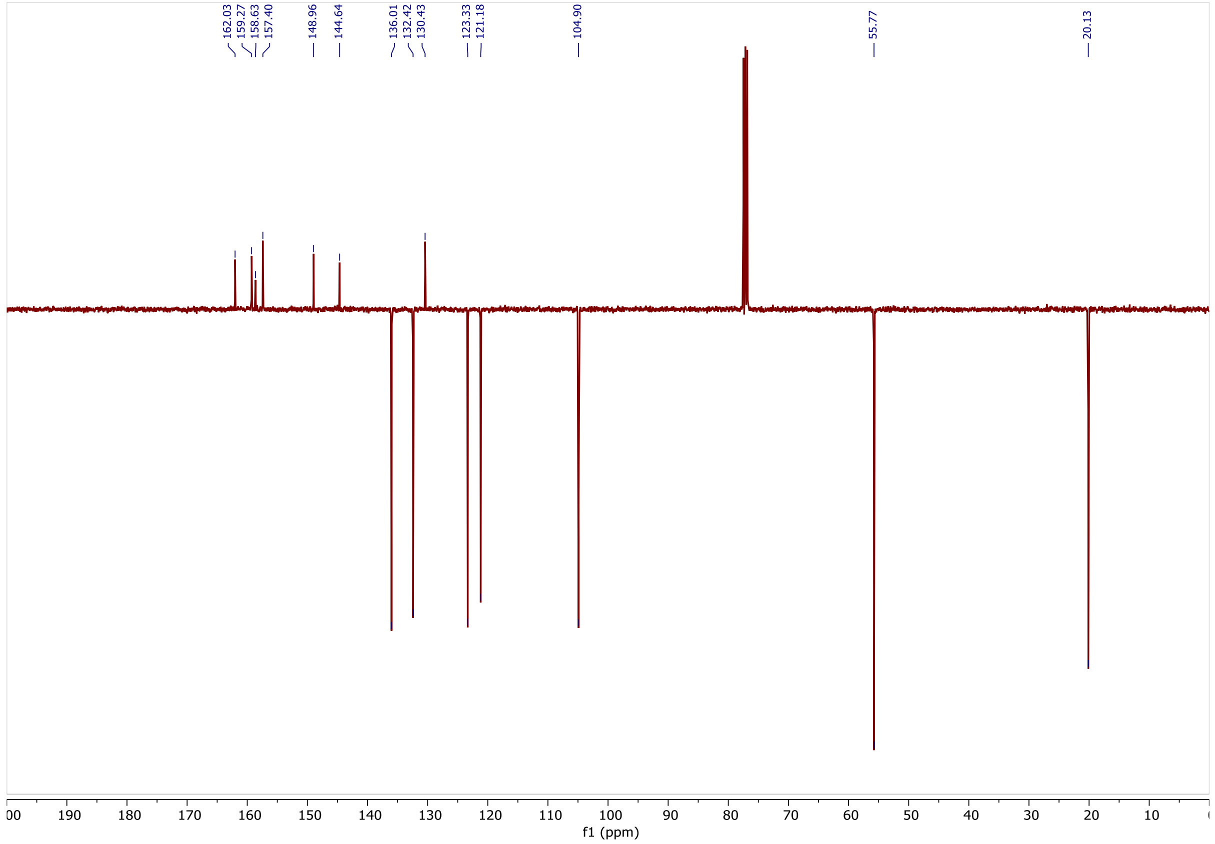

^{1}H and ^{13}C spectra of 3-(6-methoxyquinolin-2-yl)-6-methyl-1,2,4-triazine-5-carbonitrile

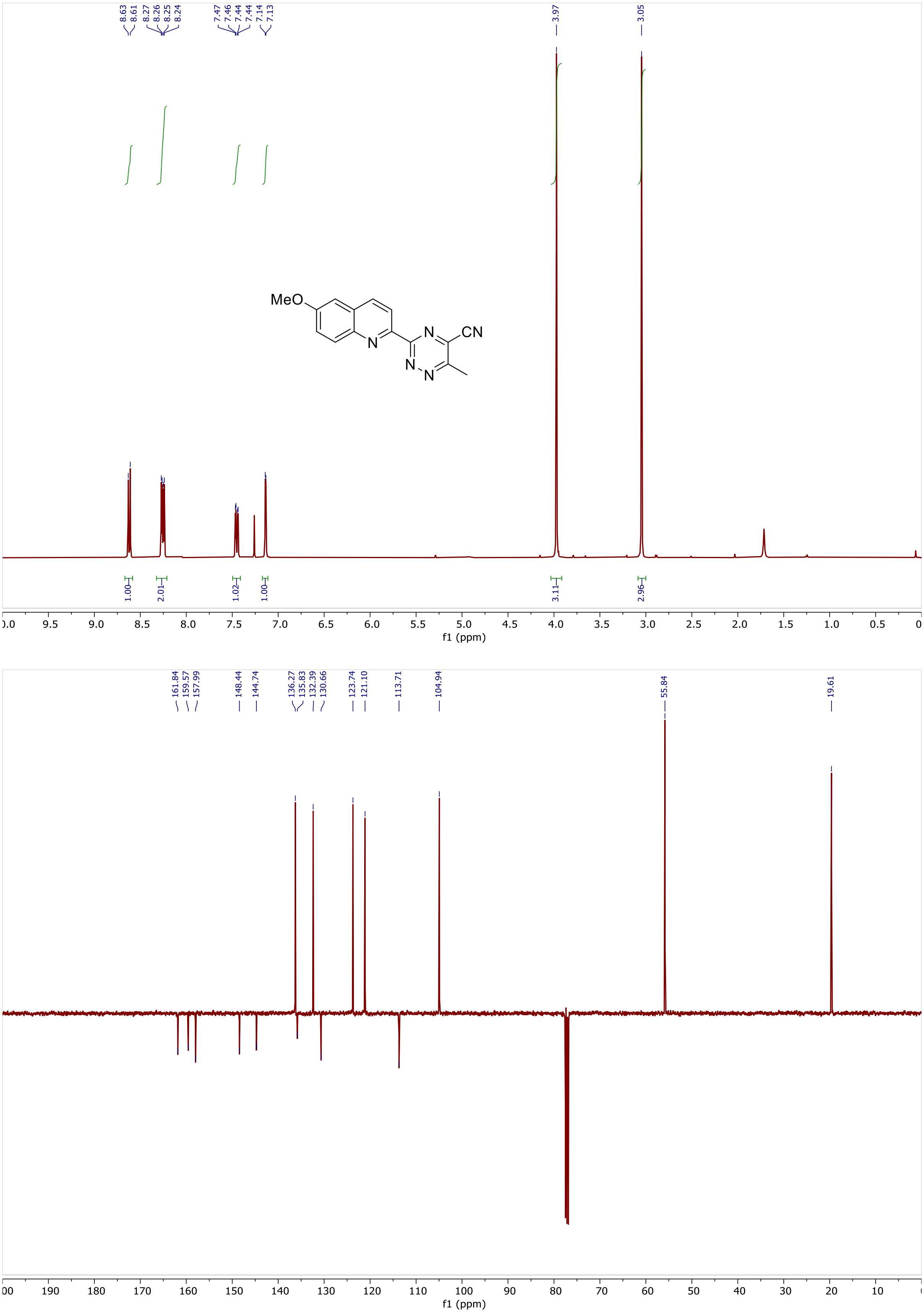

^{1}H and ^{13}C spectra of 3-(6-methoxy-5-nitroquinolin-2-yl)-6-methyl-1,2,4-triazine-5-carbonitrile

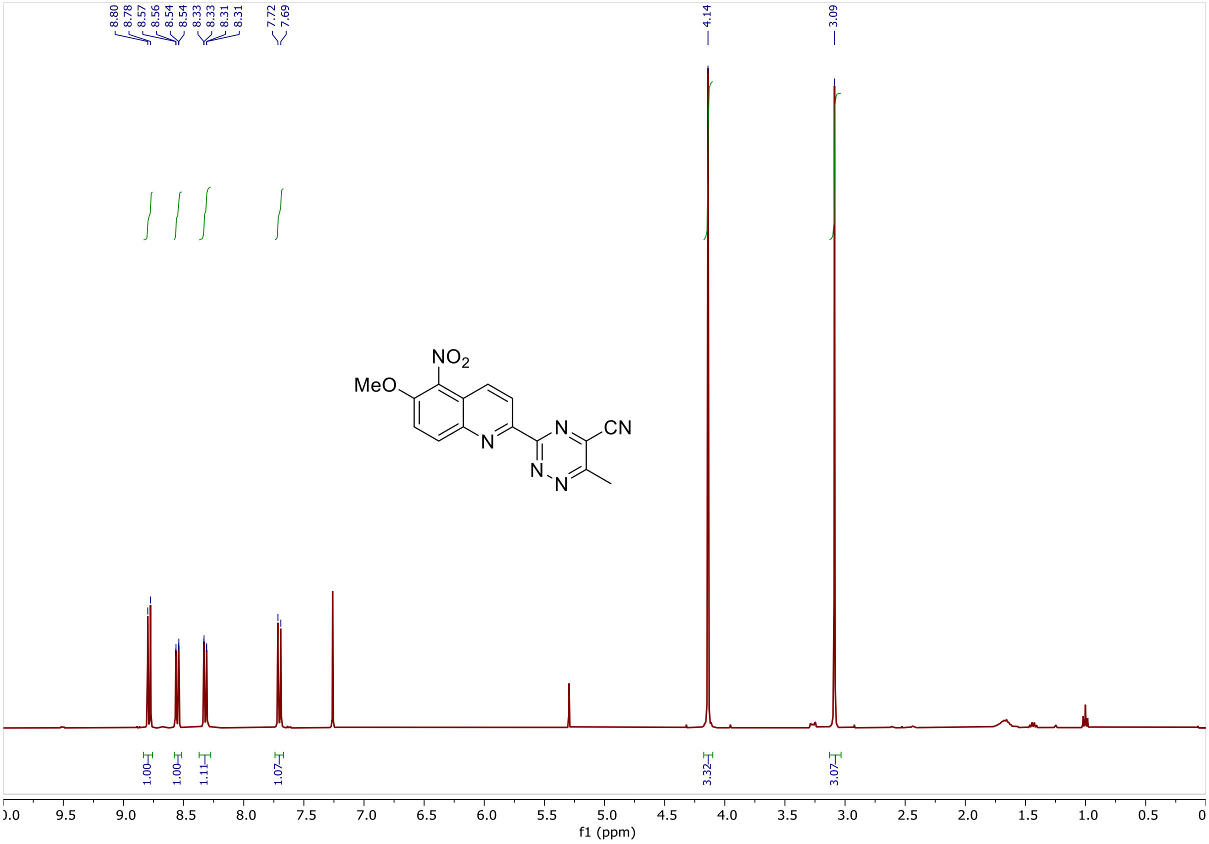

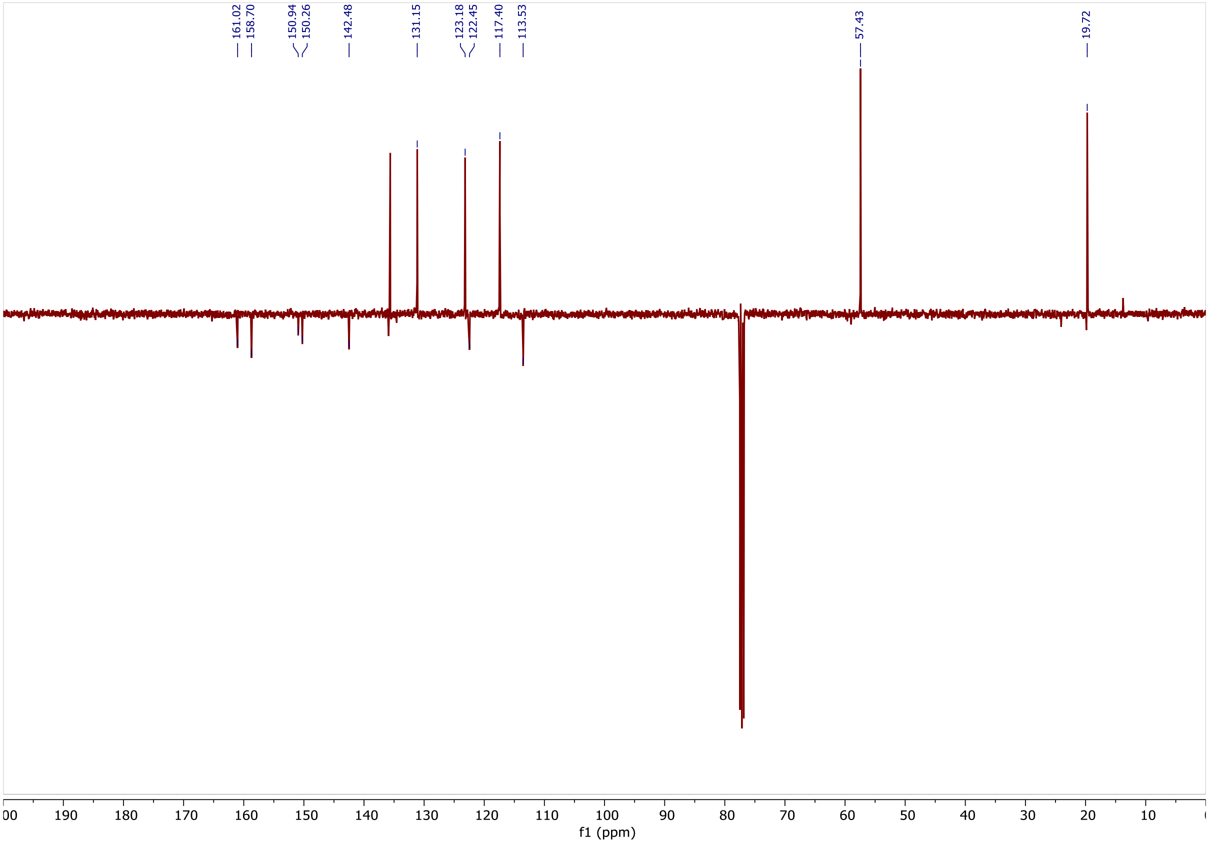

^{1}H, ^{13}C, ^{11}B, and ^{19}F spectra of 10-(2-(benzyloxy)-3,4-dimethoxyphenyl)-8-cyano-11,11-difluoro-3-methoxy-9-methyl-4-nitro-11H-pyrido[3',2':3,4][1,2]azaborolo[1,5-a]quinolin-12-ium-11-uide

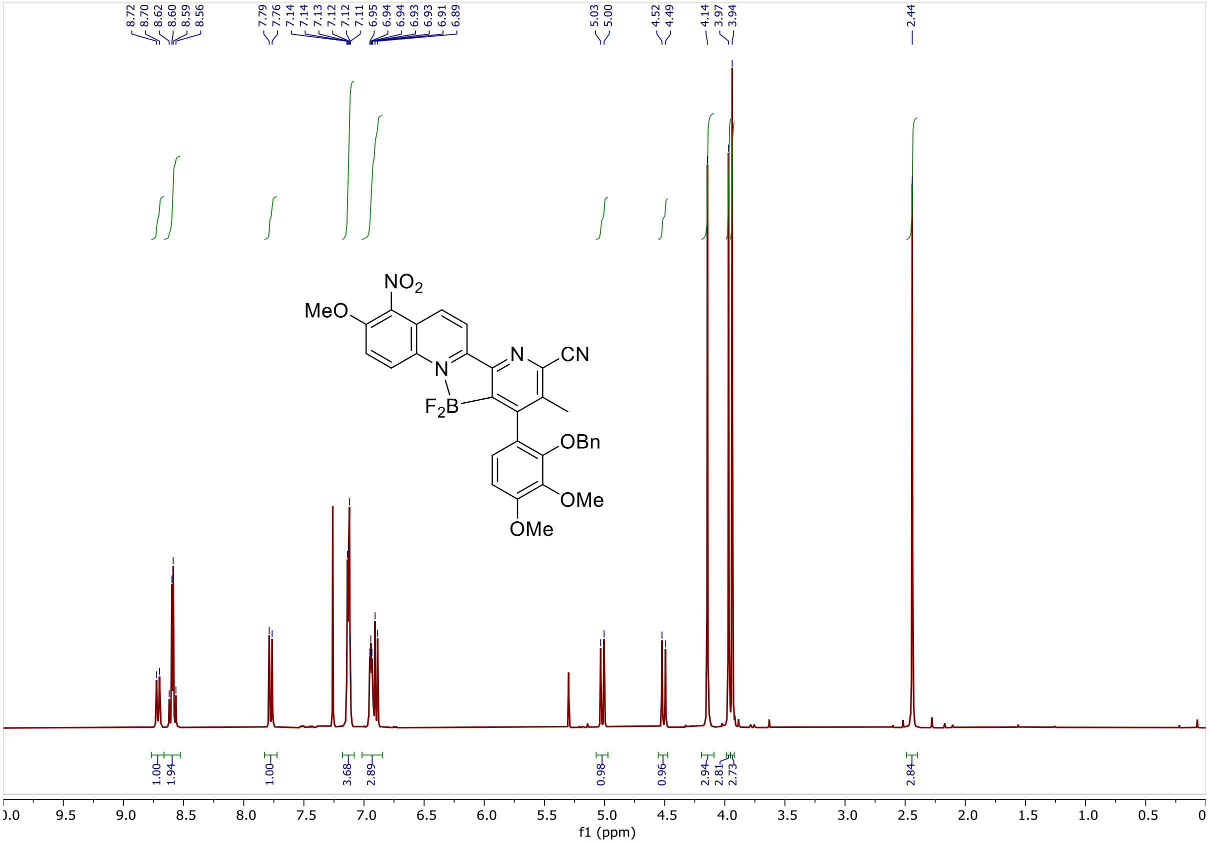

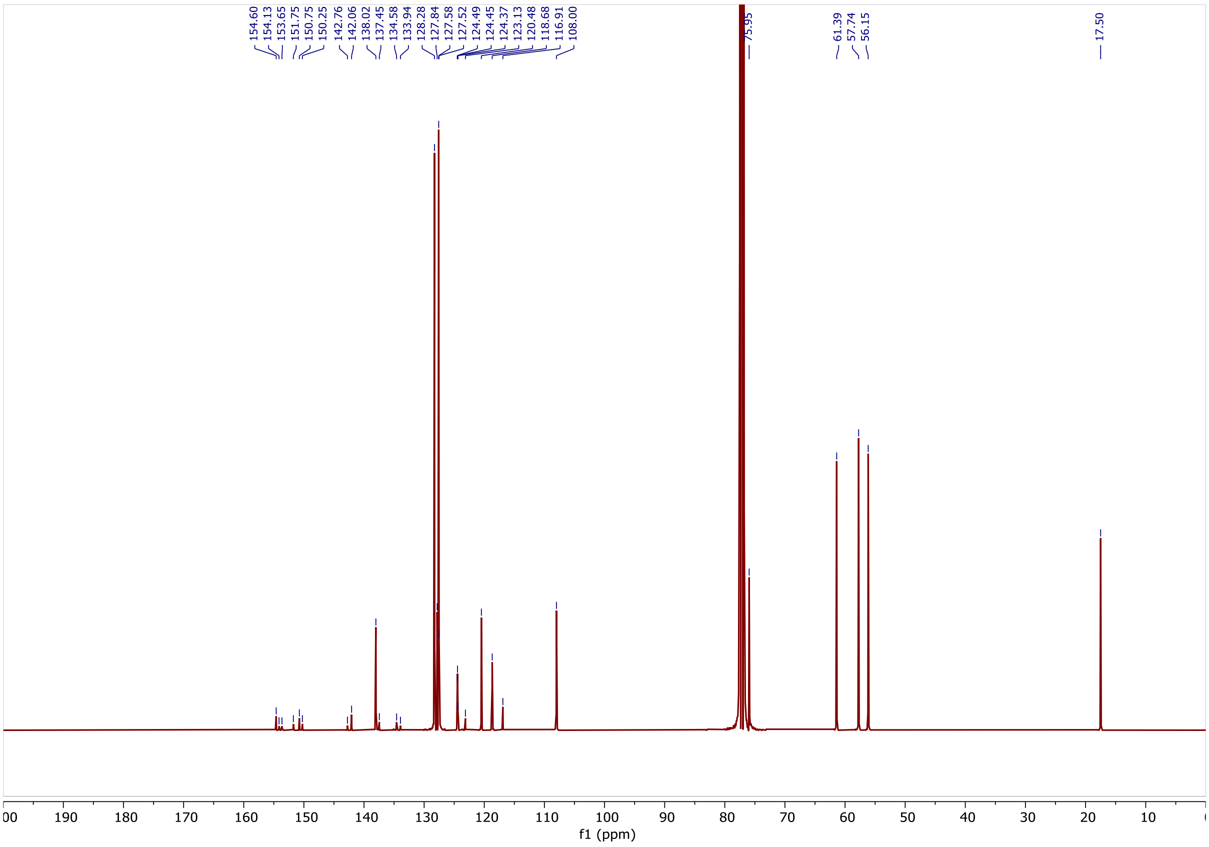

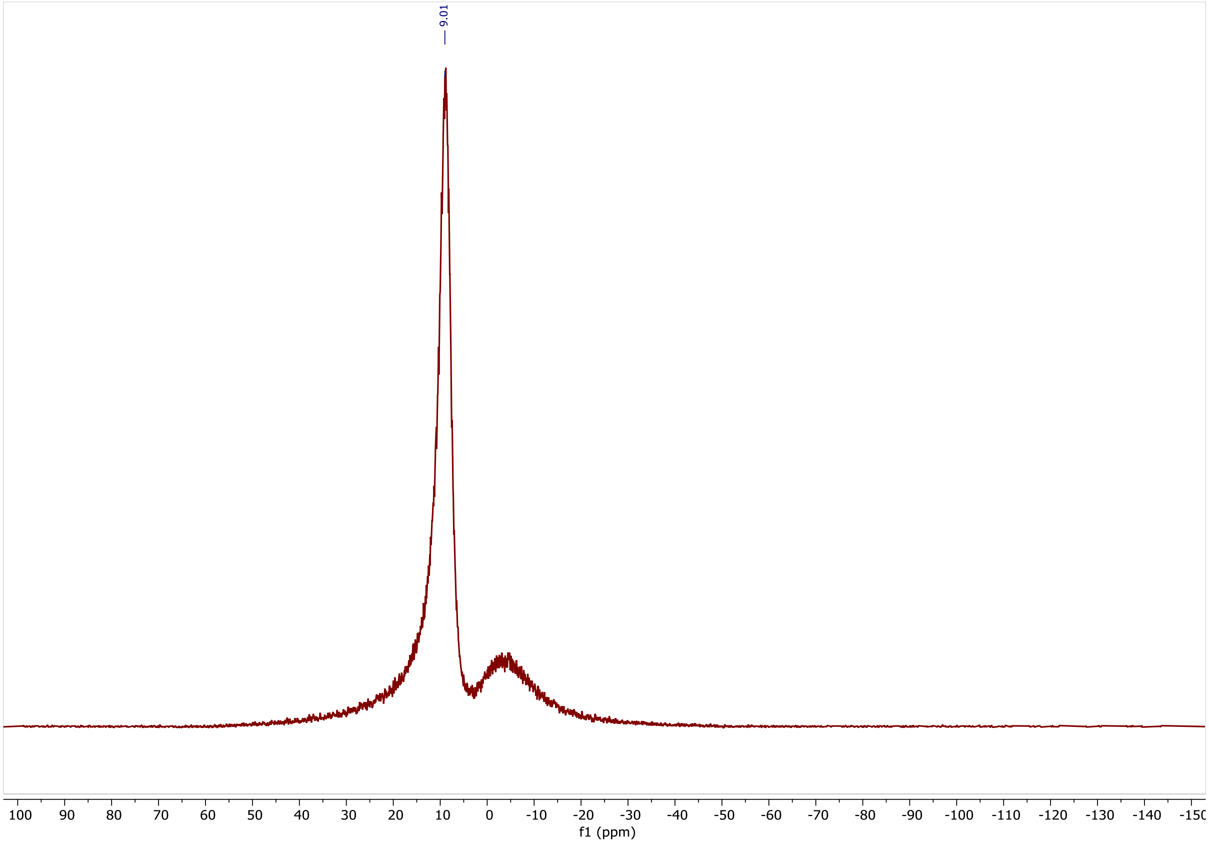
9.01
100
90
80
70
60
50
40
30
20
10
0
-10
-20
-30
-40
-50
-60
-70
-80
-90
-100
-110
-120
-130
-140
-150
f1 (ppm)

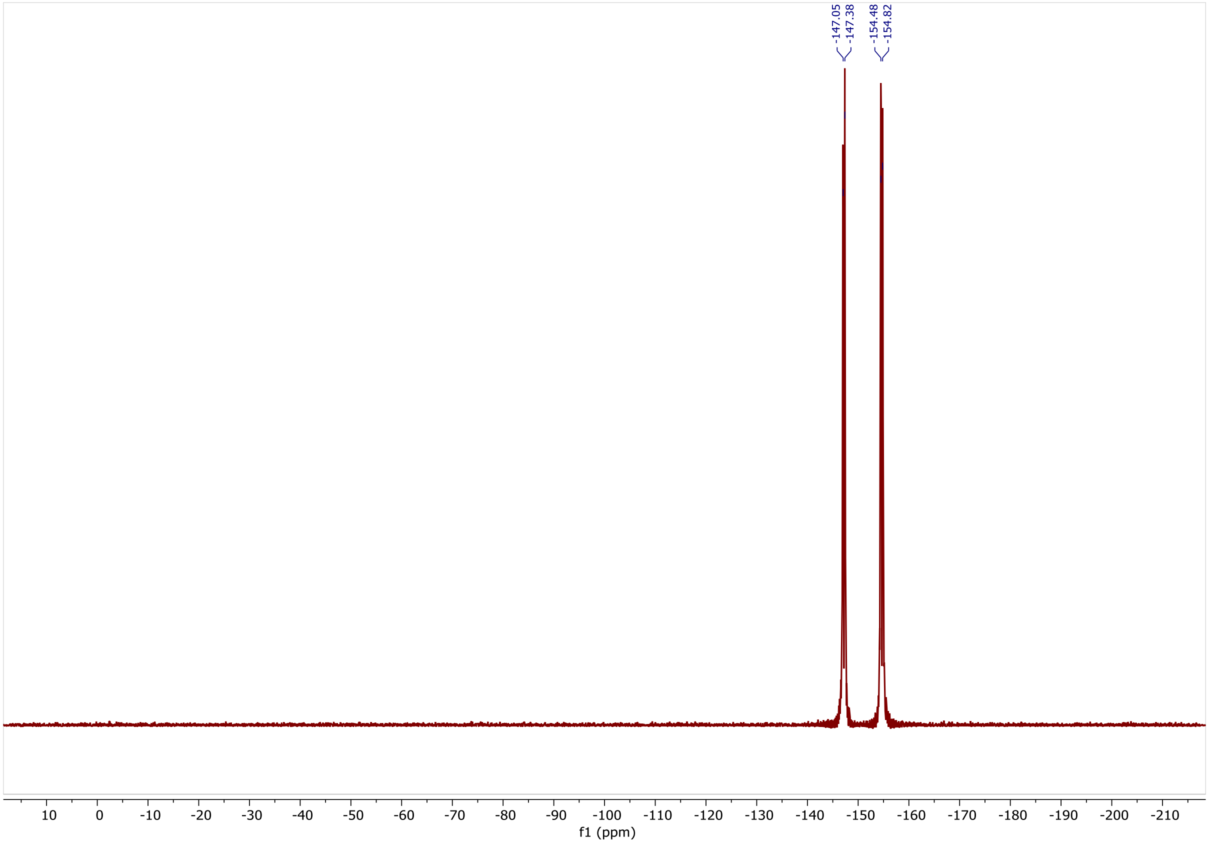
-147.05
-147.38
-154.48
-154.82
10
0
-10
-20
-30
-40
-50
-60
-70
-80
-90
-100
-110
-120
-130
-140
-150
-160
-170
-180
-190
-200
-210
f1 (ppm)

Crude ^{1}H and ^{11}B spectra of (4S,11bR)-10'-(2-(benzyloxy)-3,4-dimethoxyphenyl)-8'-cyano-3'-methoxy-9'-methyl-4'-nitrospiro[dinaphtho[2,1-d:1',2'-f][1,3,2]dioxaborepine-4,11'-pyrido[3',2':3,4][1,2]azaborolo[1,5-a]quinolin]-12'-ium-26-uide

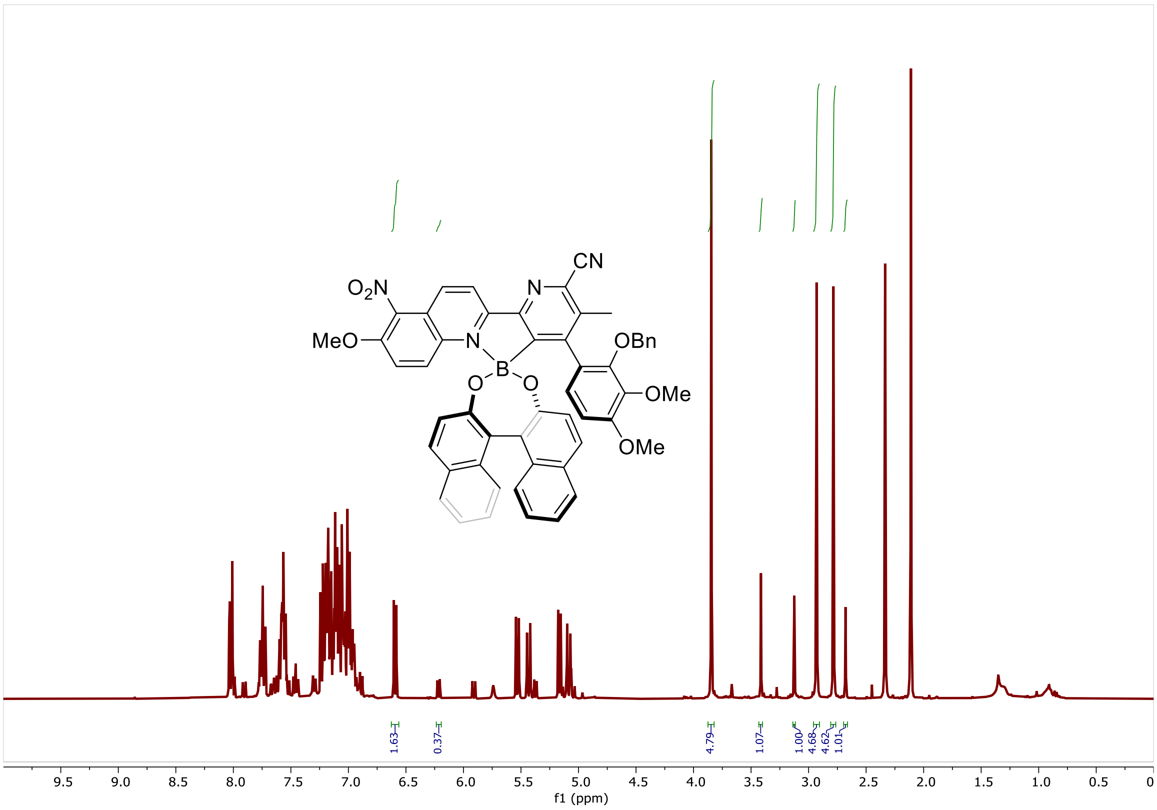

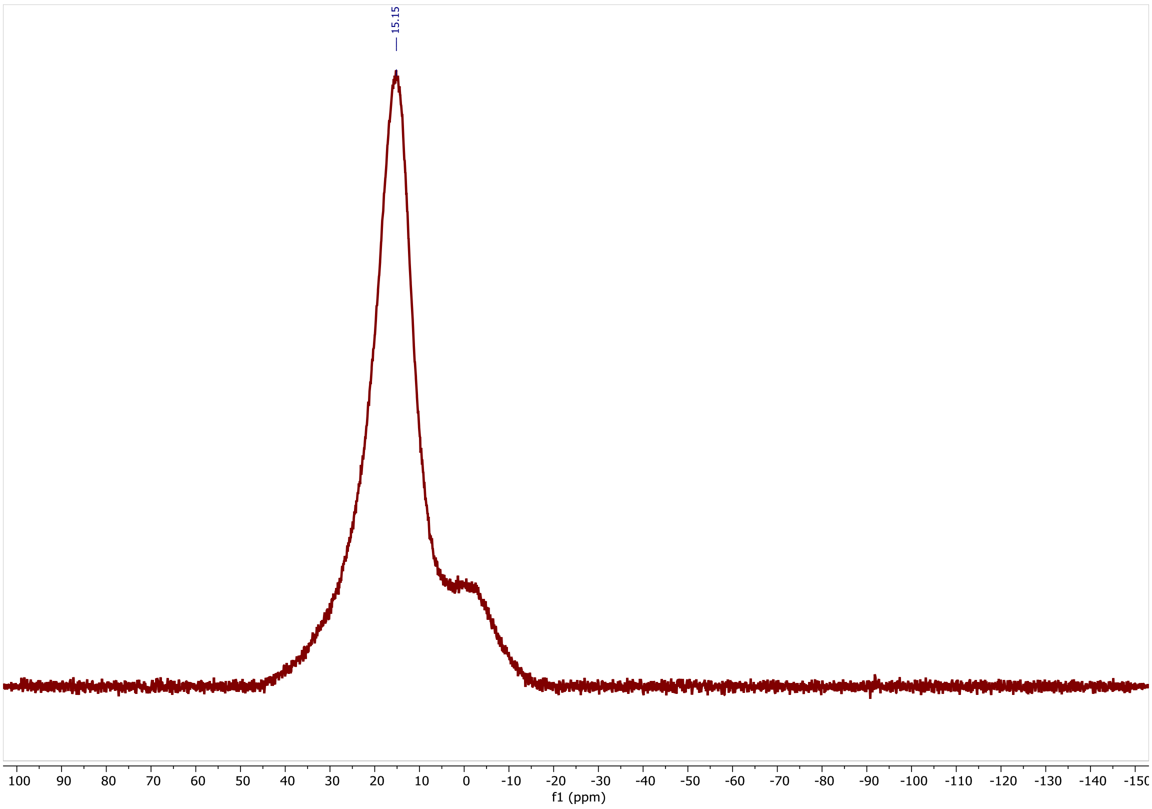

^{1}H, ^{13}C, and ^{11}B spectra of (4S,11bR)-10'-(2-(benzyloxy)-3,4-dimethoxyphenyl)-8'-cyano-3'-methoxy-9'-methyl-4'-nitrospiro[dinaphtho[2,1-d:1',2'-f][1,3,2]dioxaborepine-4,11'-pyrido[3',2':3,4][1,2]azaborolo[1,5-a]quinolin]-12'-ium-26-uide

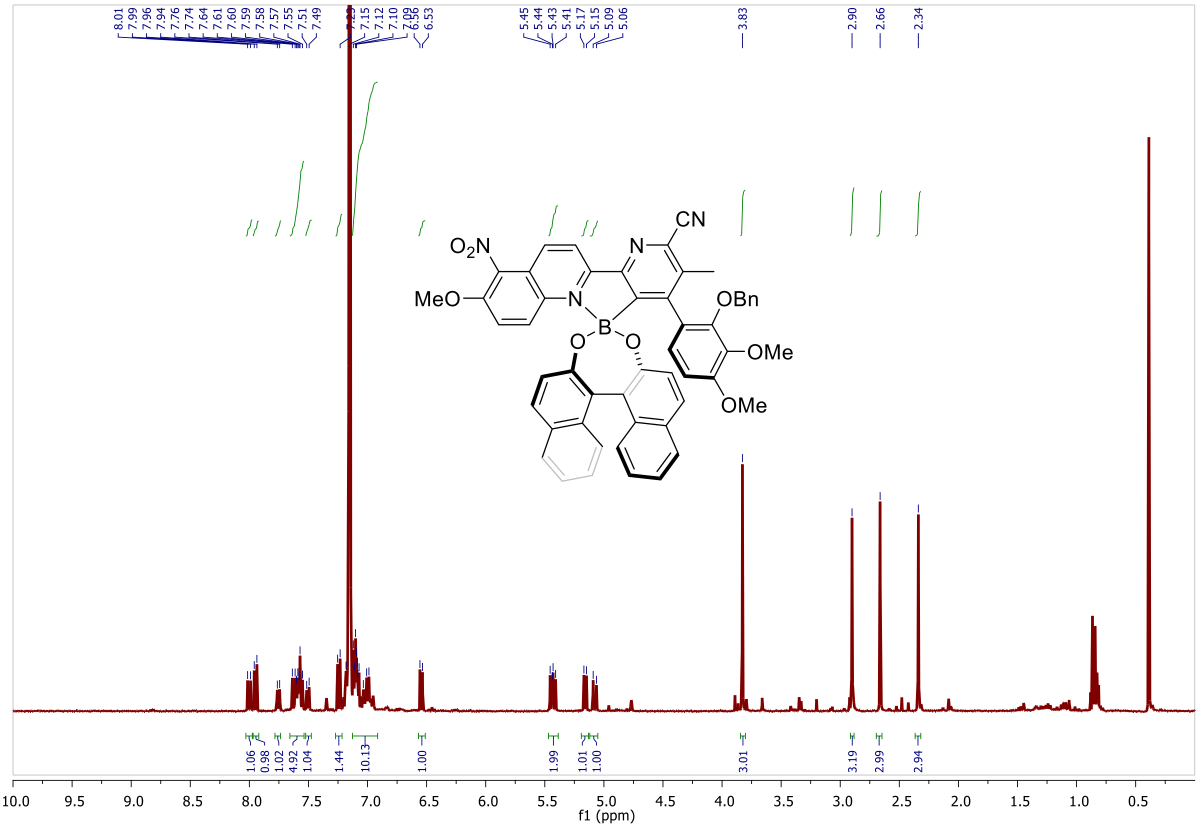

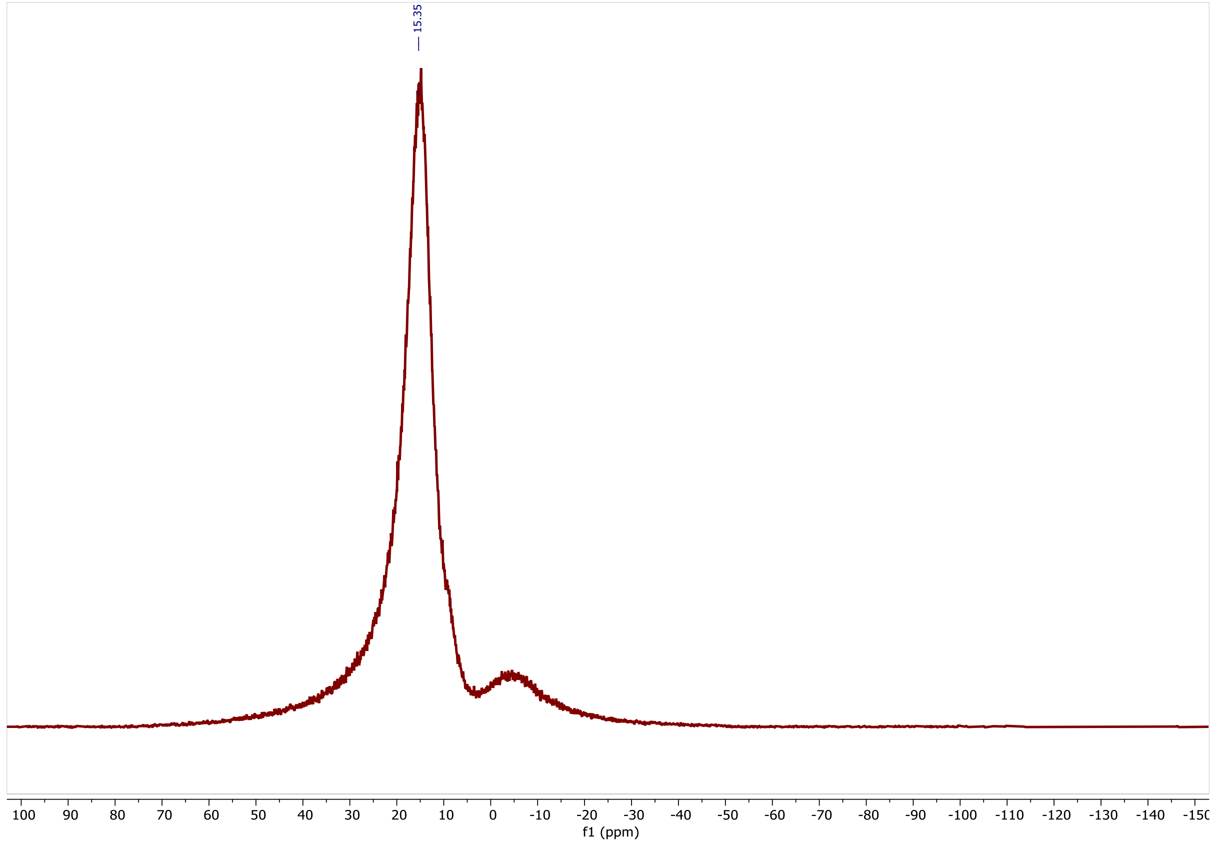

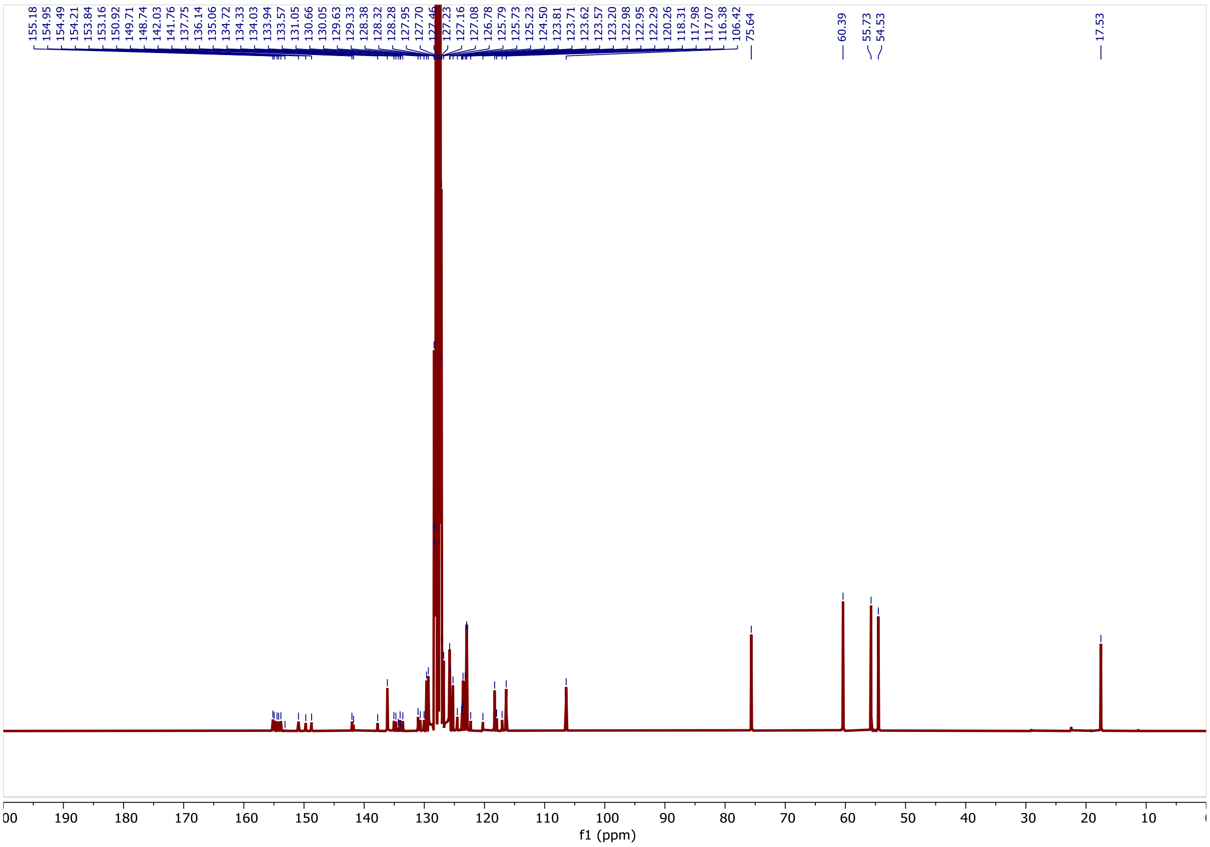
155.18
154.95
154.49
154.21
153.84
153.16
150.92
149.71
148.74
142.03
141.76
137.75
136.14
135.06
134.72
134.33
134.03
133.94
133.57
131.05
130.66
130.05
129.63
129.33
128.38
128.32
128.28
127.95
127.70
127.46
127.23
127.16
127.08
126.78
125.79
125.73
125.23
124.50
123.81
123.71
123.62
123.57
123.20
122.98
122.95
122.29
120.26
118.31
117.98
117.07
116.38
106.42
75.64
60.39
55.73
54.53
17.53
f1 (ppm)

^{1}H and ^{13}C spectra of 5-amino-4-(2-(benzyloxy)-3,4-dimethoxyphenyl)-6-(6-methoxy-5-nitroquinolin-2-yl)-3-methylpicolinonitrile

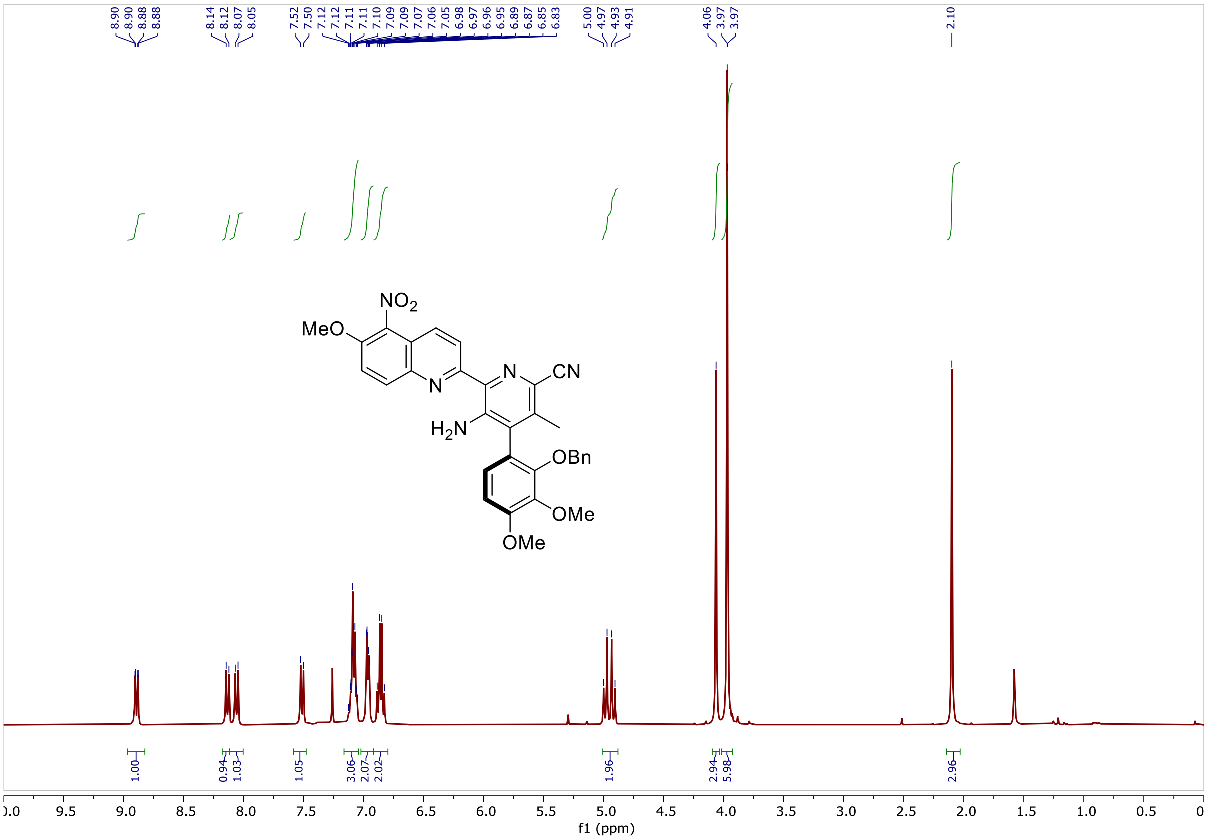

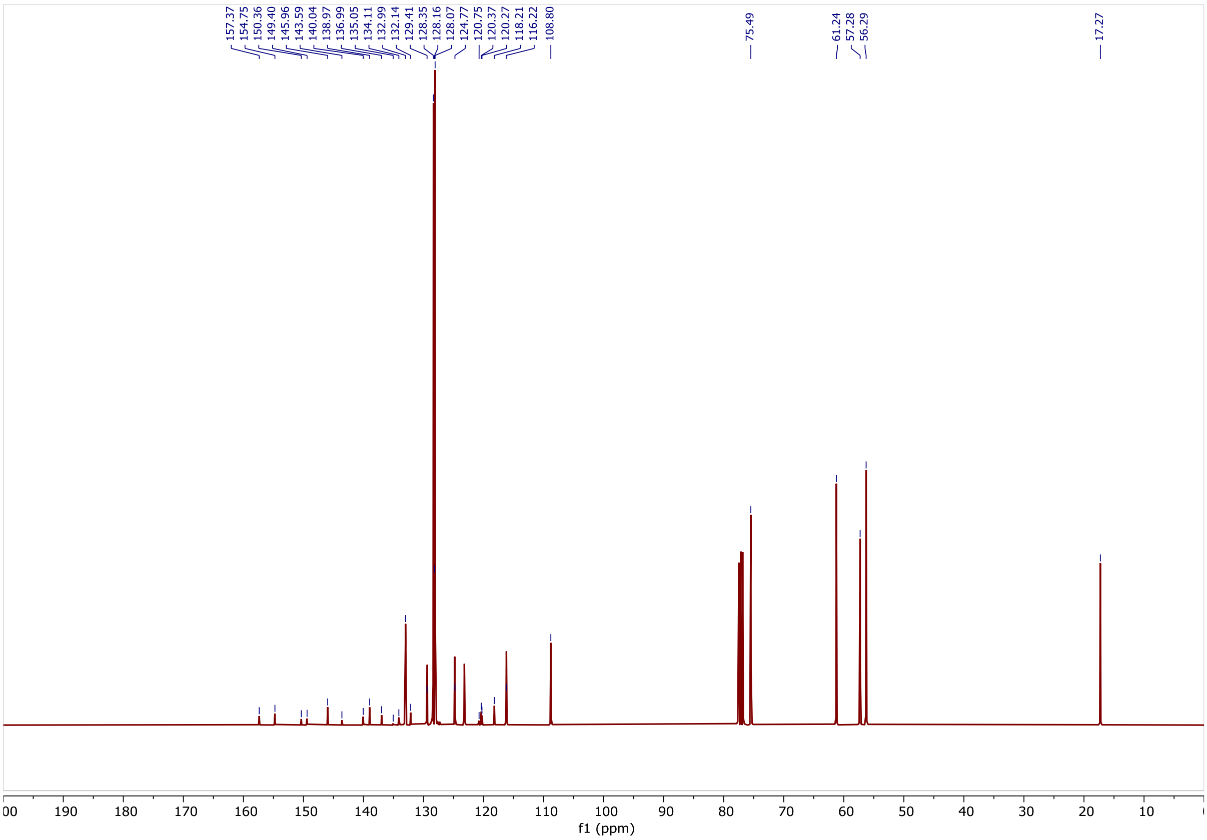

^{1}H and ^{13}C spectra of 5-amino-4-(2-(benzyloxy)-3,4-dimethoxyphenyl)-6-(6-methoxy-5-nitroquinolin-2-yl)-3-methylpicolinaldehyde

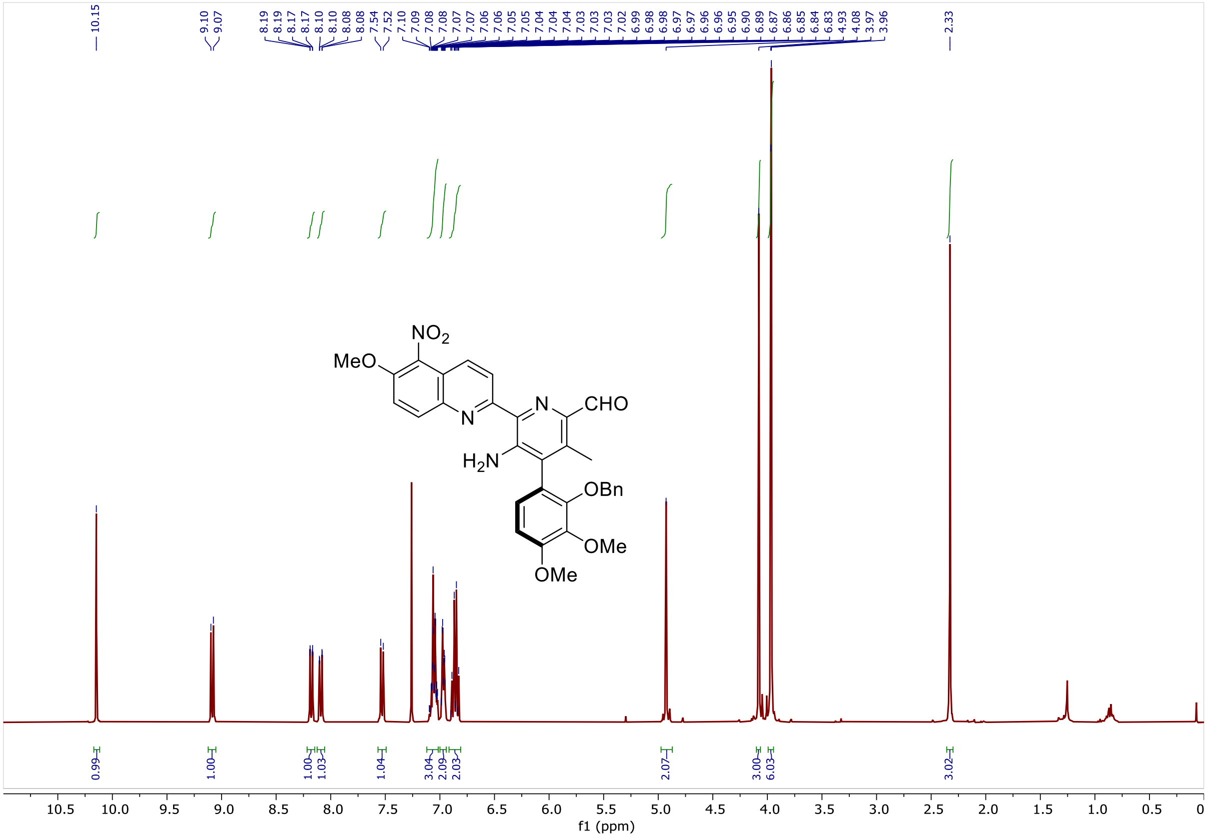

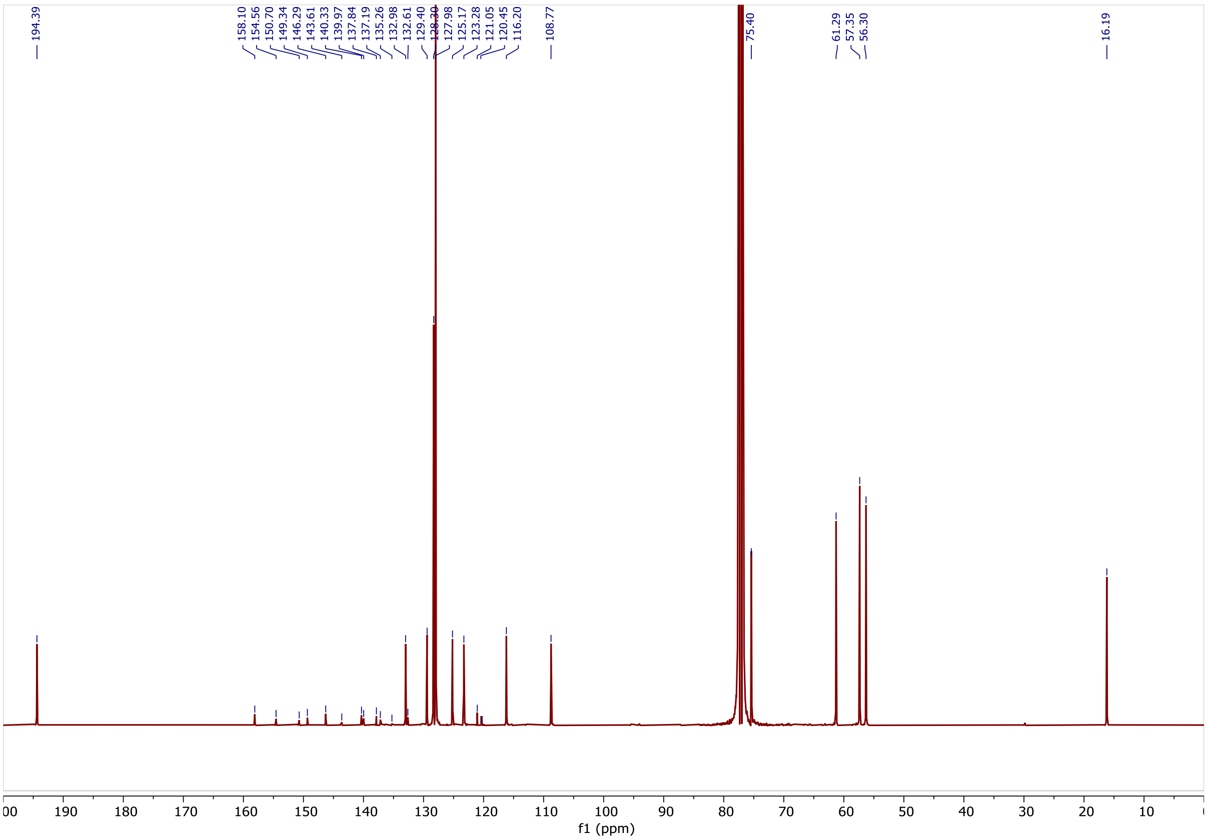

^{1}H and ^{13}C spectra of methyl 5-amino-4-(2-(benzyloxy)-3,4-dimethoxyphenyl)-6-(6-methoxy-5-nitroquinolin-2-yl)-3-methylpicolinate

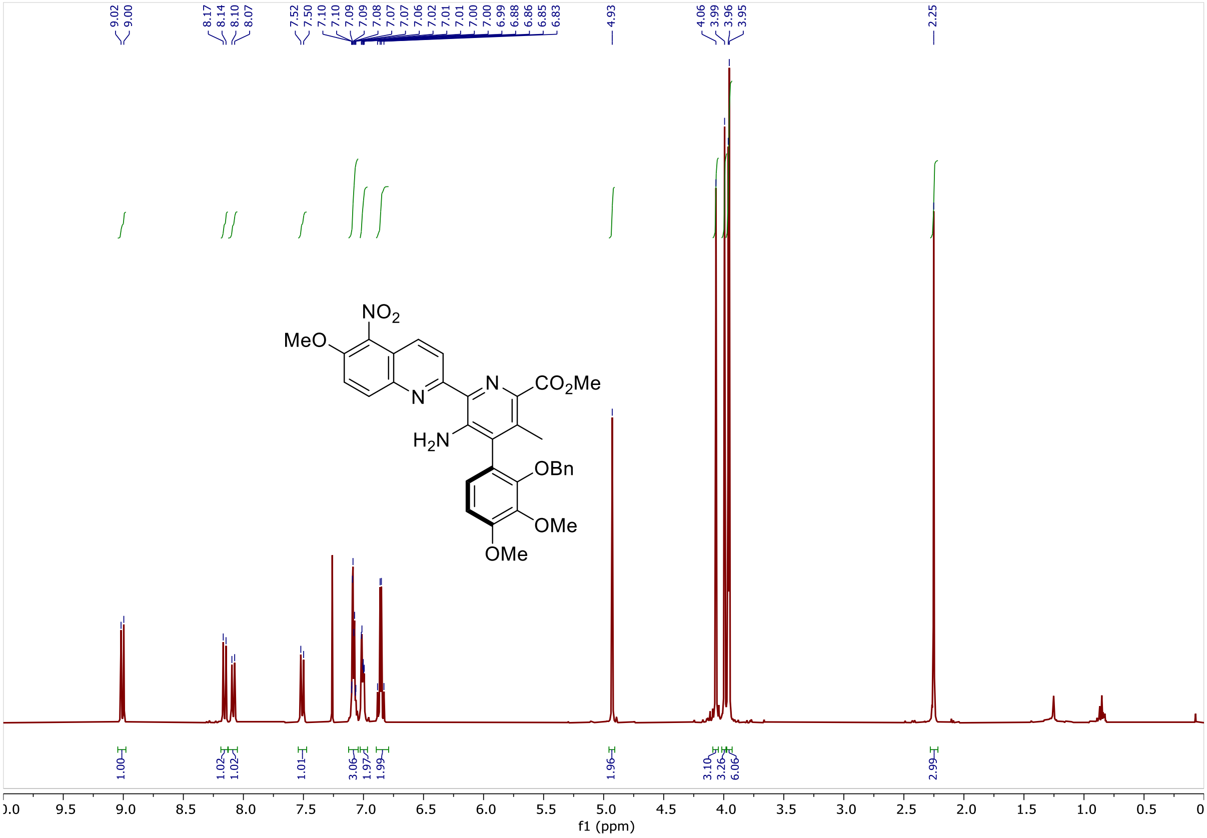

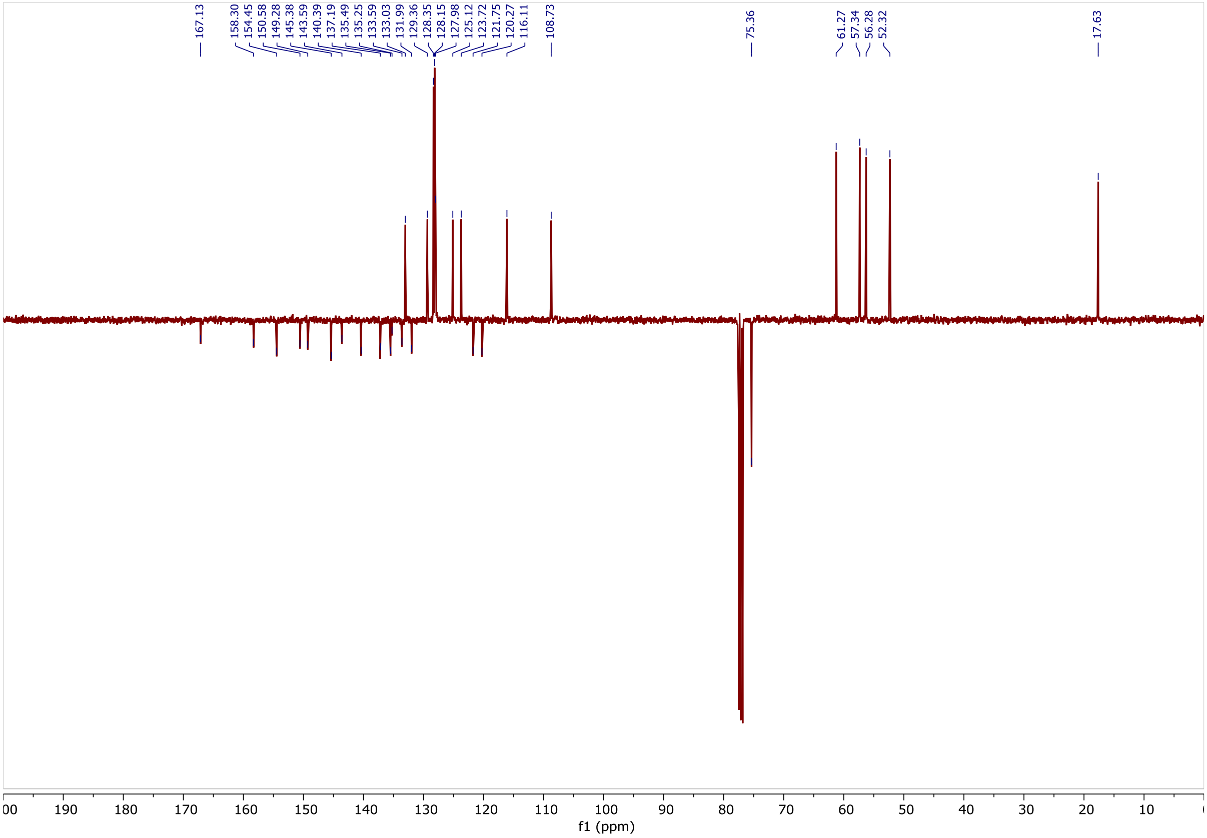

^{1}H and ^{13}C spectra of methyl 5-amino-4-(2-(benzyloxy)-3,4-dimethoxyphenyl)-6-(6-methoxy-5,8-dioxo-5,8-dihydroquinolin-2-yl)-3-methylpicolinate

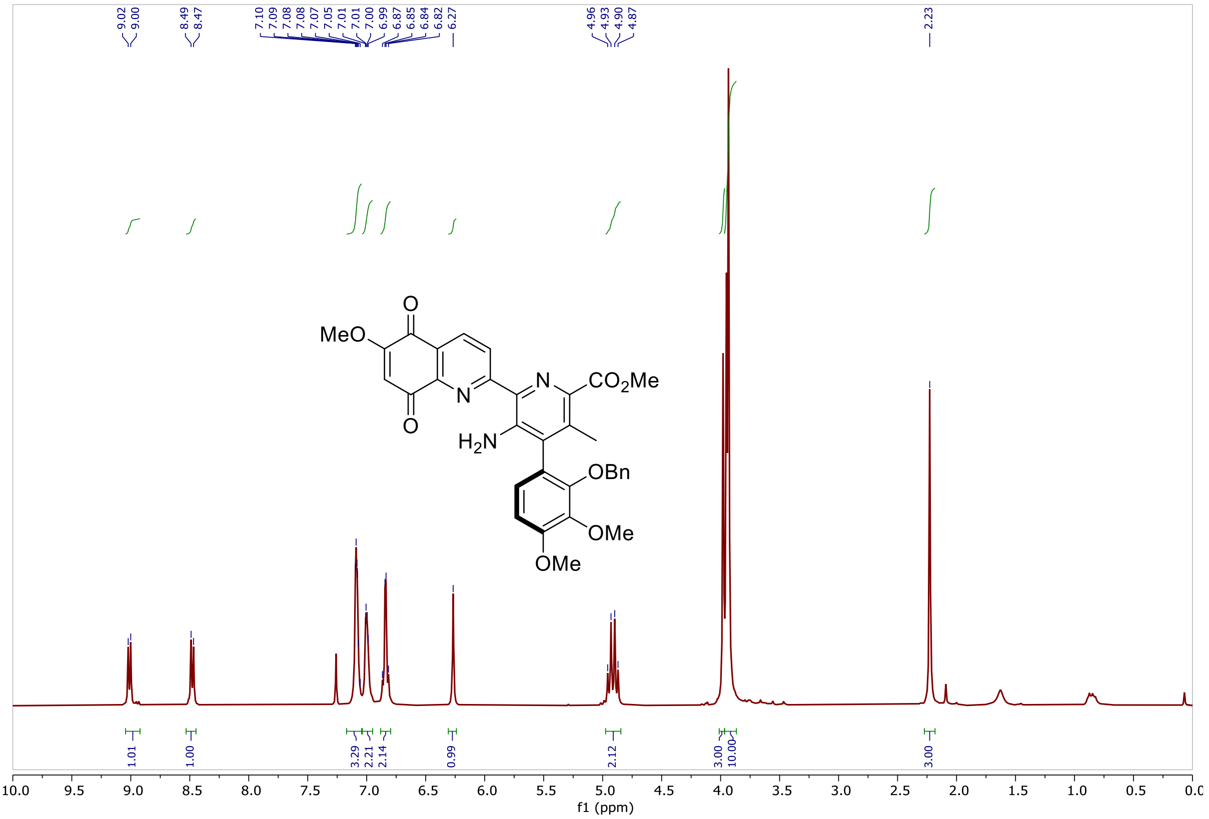

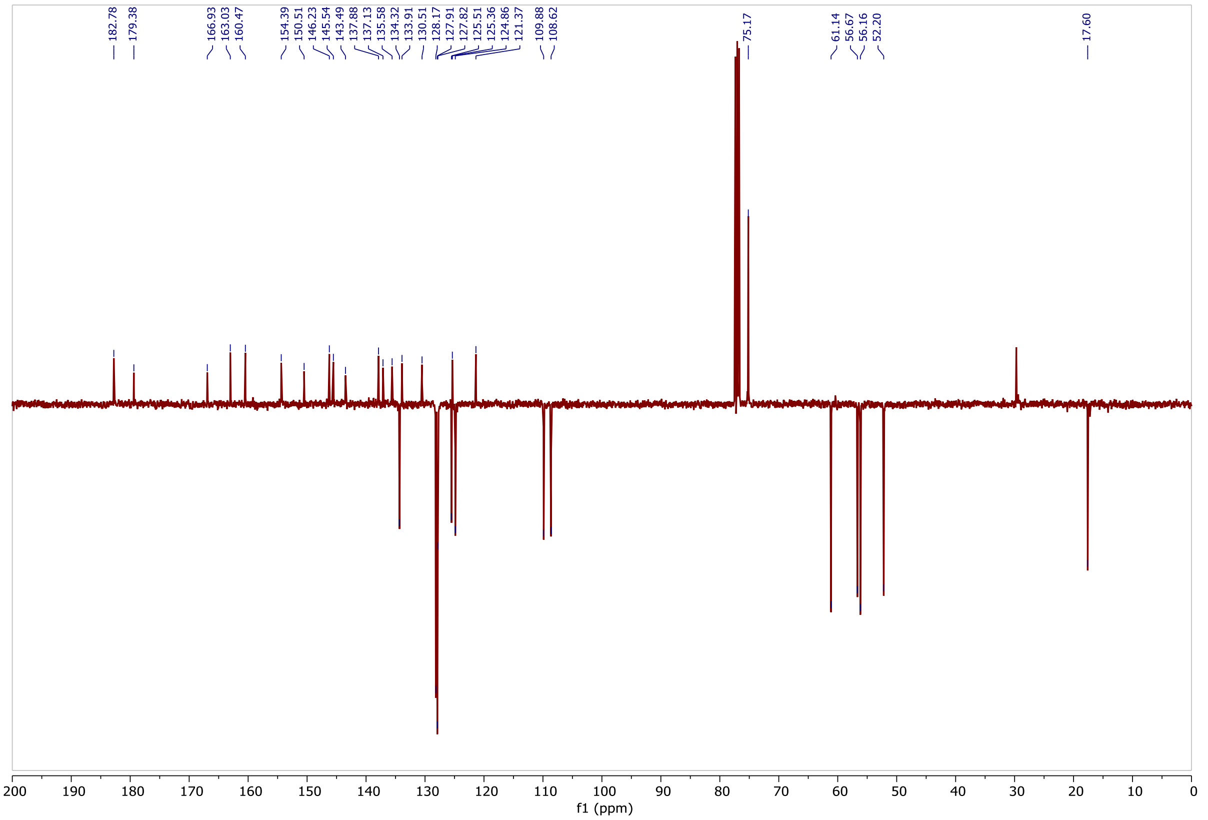

^{1}H and ^{13}C spectra of methyl 5-amino-6-(7-amino-6-methoxy-5,8-dioxo-5,8-dihydroquinolin-2-yl)-4-(2-hydroxy-3,4-dimethoxyphenyl)-3-methylpicolinate

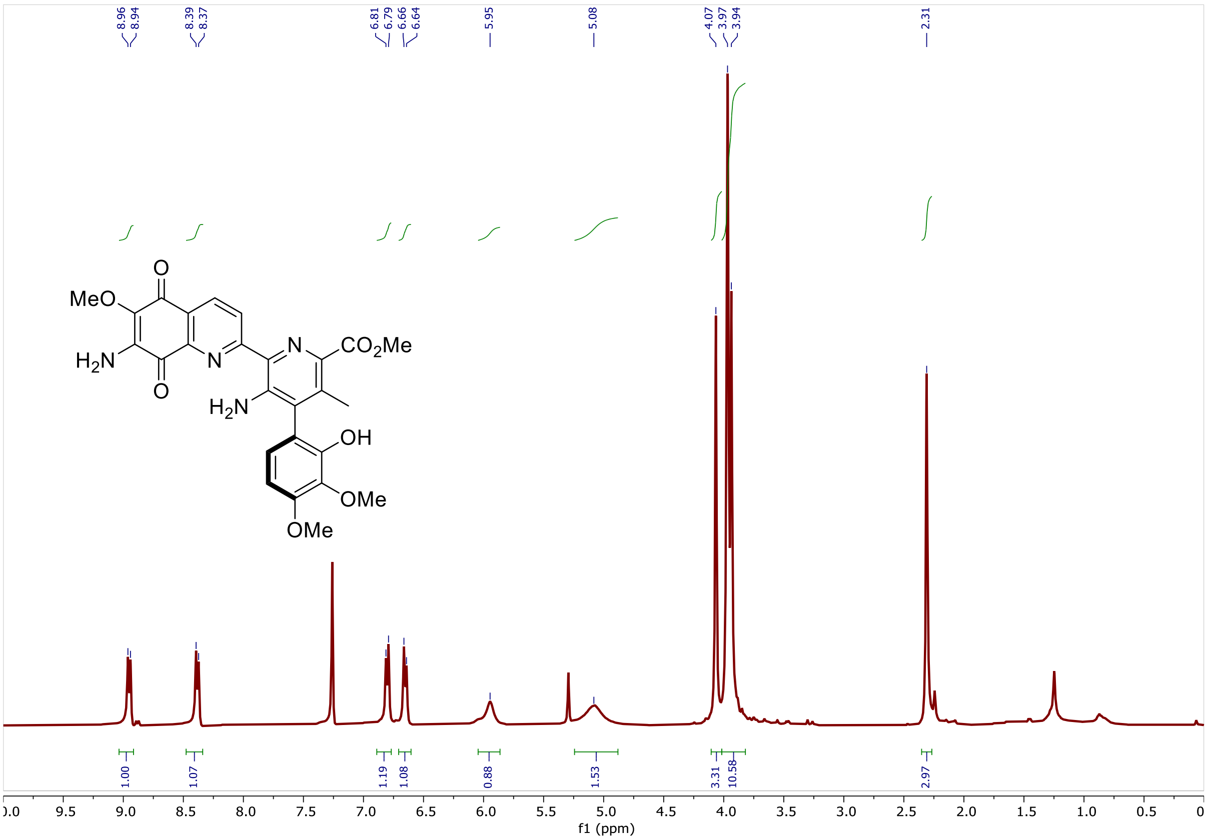

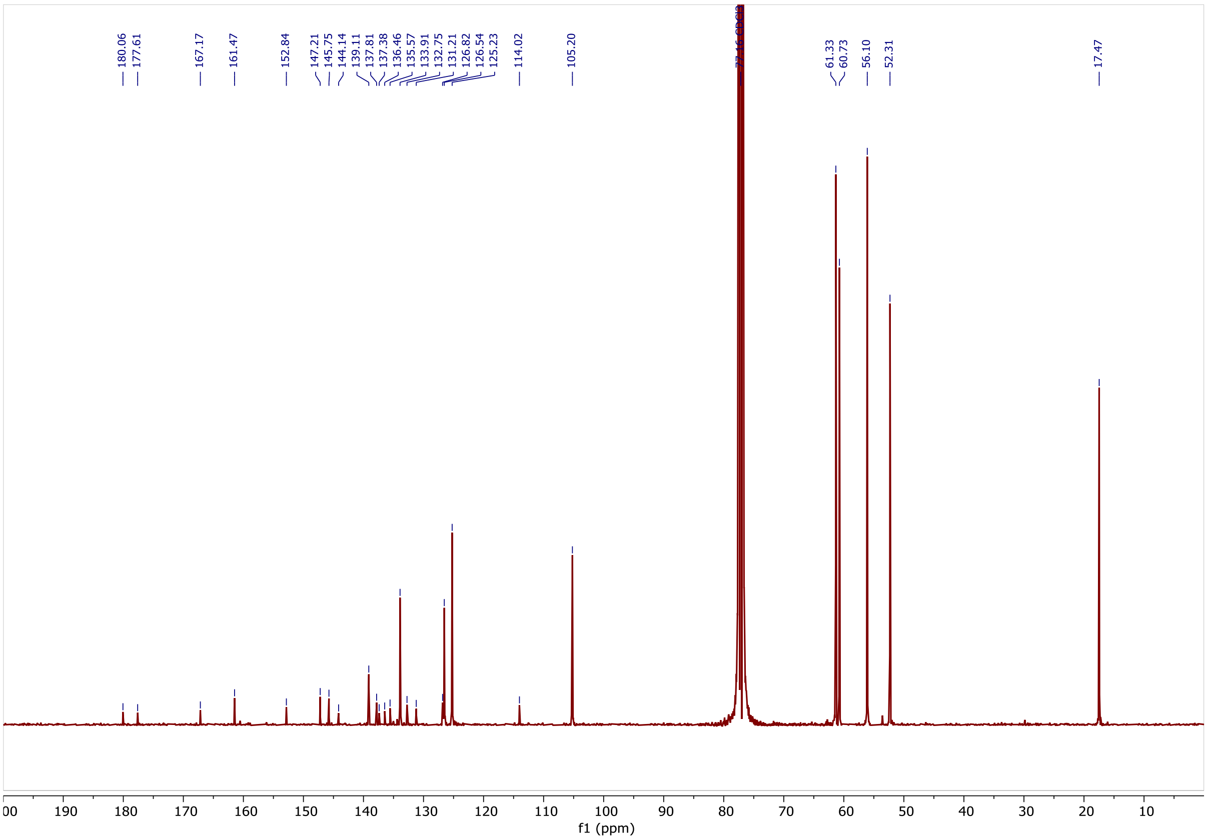

^{1}H and ^{13}C spectra of streptonigrin

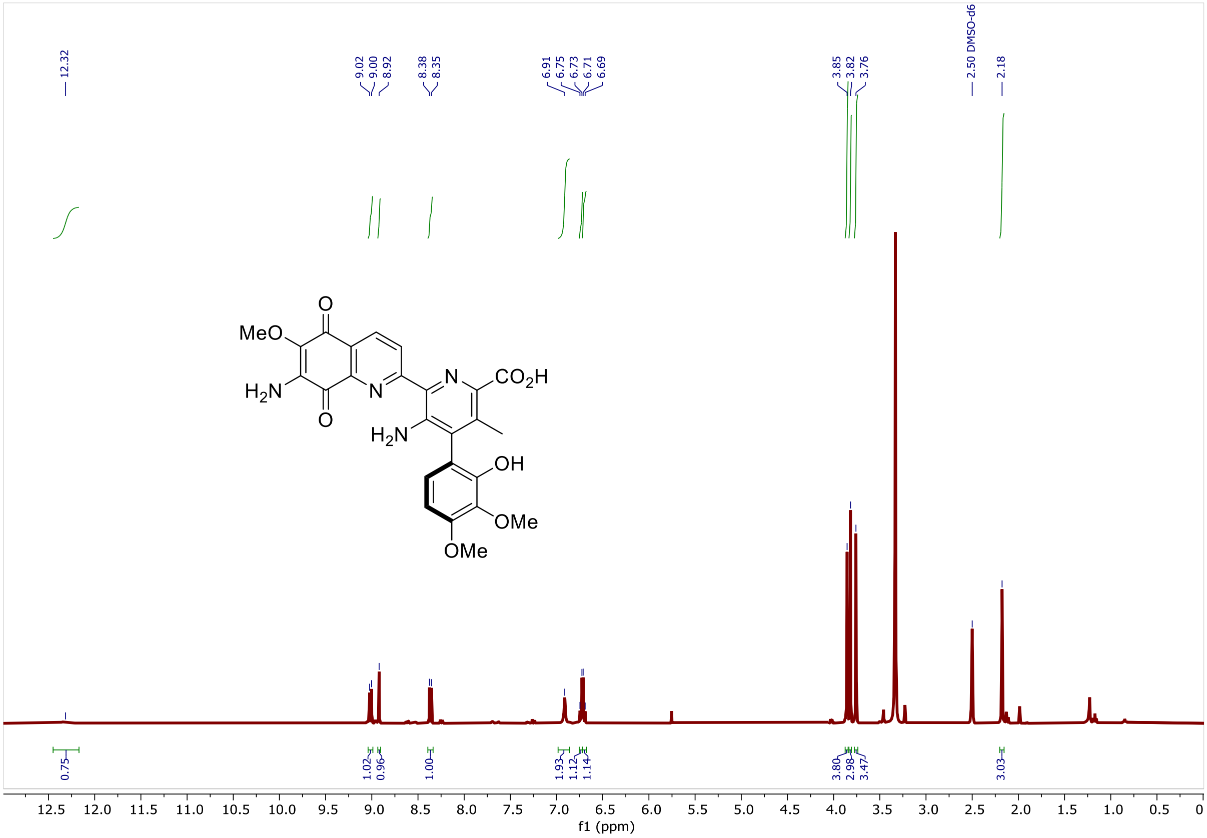

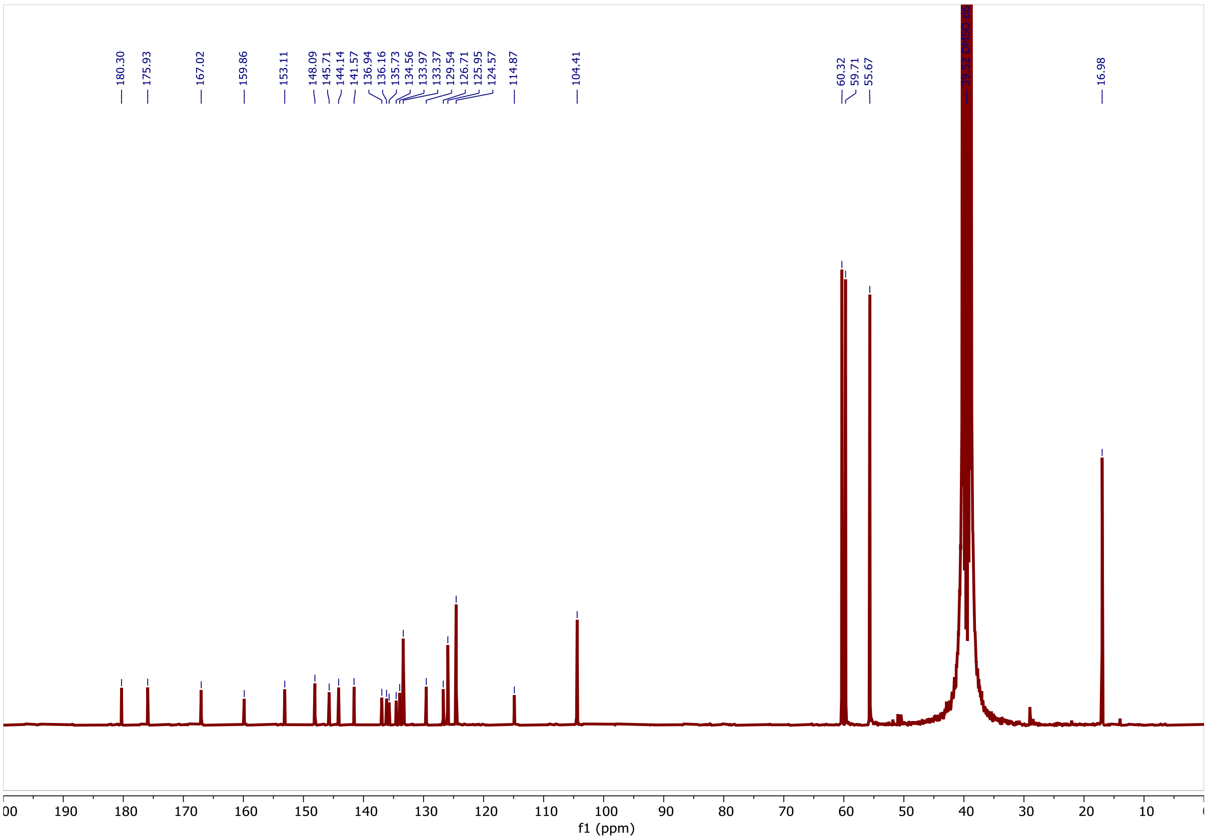

Crystallographic Data

X-ray crystallographic analysis of 3-(6-methoxy-5-nitroquinolin-2-yl)-6-methyl-1,2,4-triazine-5-carbonitrile 3

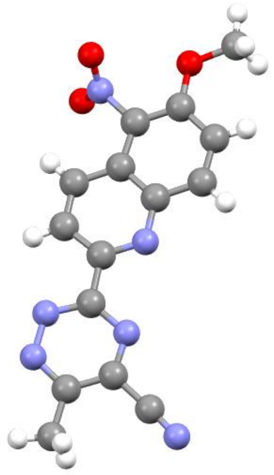

Figure S1. X-ray crystal structure of 3-(6-methoxy-5-nitroquinolin-2-yl)-6-methyl-1,2,4-triazine-5-carbonitrile.

Crystals of 3-(6-methoxy-5-nitroquinolin-2-yl)-6-methyl-1,2,4-triazine-5-carbonitrile were grown from a saturated DCM solution, allowing slow evaporation.

Crystal Data for $C_{15}H_{10}N_6O_3$ (M =322.29 g/mol): monoclinic, space group $P2_1/c$ (no. 14), a = 9.711(4) Å, b = 7.752(3) Å, c = 19.014(7) Å, β = 91.177(10)°, V = 1430.9(9) $Å^3$, Z = 4, T = 100 K, μ(MoKα) = 0.110 mm^{-1}, $Dcalc$ = 1.496 g/cm^3, 28844 reflections measured (4.196° ≤ 2Θ ≤ 55.242°), 3305 unique (R_{int} = 0.1614, R_{sigma} = 0.0900) which were used in all calculations. The final R_1 was 0.0729 (I > 2σ(I)) and wR_2 was 0.2204 (all data).

Table 1 Crystal data and structure refinement for ojh417S.

Identification code	ojh417S
Empirical formula	$C_{15}H_{10}N_6O_3$
Formula weight	322.29
Temperature/K	100
Crystal system	monoclinic
Space group	$P2_1/c$
a/Å	9.711(4)
b/Å	7.752(3)
c/Å	19.014(7)
α/°	90
β/°	91.177(10)
γ/°	90
Volume/$Å^3$	1430.9(9)
Z	4
$\rho_{calc}g/cm^3$	1.496

μ/mm^{-1}	0.110
F(000)	664.0
Crystal size/mm^3	0.322 × 0.173 × 0.093
Radiation	MoKα (λ = 0.71073)
2Θ range for data collection/°	4.196 to 55.242
Index ranges	-12 ≤ h ≤ 12, -10 ≤ k ≤ 10, -24 ≤ l ≤ 24
Reflections collected	28844
Independent reflections	3305 [R_{int} = 0.1614, R_{sigma} = 0.0900]
Data/restraints/parameters	3305/0/219
Goodness-of-fit on F^2	1.063
Final R indexes [I>=2σ (I)]	R_1 = 0.0729, wR_2 = 0.1851
Final R indexes [all data]	R_1 = 0.1300, wR_2 = 0.2204
Largest diff. peak/hole / e Å^{-3}	0.36/-0.44

Table 2 Fractional Atomic Coordinates (×10^4) and Equivalent Isotropic Displacement Parameters (Å^2×10^3) for ojh417S. U_{eq} is defined as 1/3 of the trace of the orthogonalised U_{IJ} tensor.

**Atom**	***x***	***y***	***z***	**U(eq)**
O1	-183(2)	6922(3)	6267.9(12)	25.6(6)
O2	1572(3)	4719(3)	7108.4(12)	35.4(7)
O3	740(2)	2566(3)	6501.2(12)	28.5(6)
N1	3518(3)	4712(3)	4276.7(13)	20.2(6)
N2	6155(3)	1554(4)	4061.4(14)	22.6(6)
N3	7040(3)	926(4)	3586.6(14)	22.2(6)
N4	5157(3)	3497(4)	3217.9(13)	21.5(6)
N5	1253(3)	4021(4)	6547.9(14)	23.5(6)
N6	6113(3)	4199(4)	1511.8(15)	29.5(7)
C1	2616(3)	5230(4)	4772.7(16)	19.2(7)
C2	1817(3)	6734(4)	4647.5(17)	21.6(7)
C3	898(3)	7329(4)	5123.6(17)	23.3(7)
C4	715(3)	6444(4)	5767.4(17)	21.6(7)
C5	1475(3)	4974(4)	5892.6(16)	19.6(7)
C6	2439(3)	4307(4)	5414.7(16)	18.6(7)
C7	3272(3)	2848(4)	5540.5(17)	23.5(7)
C8	4209(3)	2372(4)	5049.5(17)	23.2(7)
C9	4280(3)	3327(4)	4414.9(16)	19.9(7)
C10	5250(3)	2768(4)	3864.1(16)	19.2(7)
C11	6992(3)	1534(4)	2927.3(16)	21.3(7)
C12	6044(3)	2859(4)	2764.9(16)	21.3(7)
C13	-927(4)	8513(5)	6174.8(18)	29.3(8)
C14	7967(4)	825(5)	2414.2(18)	26.4(8)
C15	6040(3)	3619(4)	2062.9(18)	22.4(7)

Table 3 Anisotropic Displacement Parameters (Å^2×10^3) for ojh417S. The Anisotropic displacement factor exponent takes the form: -2π^2[h^{2}a*^{2}U$_{11}$+2hka*b*U$_{12}$+…].

Atom	U$_{11}$	U$_{22}$	U$_{33}$	U$_{23}$	U$_{13}$	U$_{12}$
O1	22.1(13)	25.3(13)	29.8(13)	0.1(10)	7.2(10)	3.4(10)
O2	43.5(17)	37.9(15)	24.7(13)	1.0(11)	-0.1(11)	-11.0(13)
O3	29.3(14)	20.6(13)	35.8(14)	2.6(10)	6.1(11)	-3.7(10)
N1	16.7(14)	18.9(14)	25.0(14)	0.8(11)	0.3(11)	-0.9(11)
N2	19.7(15)	20.5(14)	27.6(15)	-1.7(12)	1.8(11)	-0.6(12)
N3	16.4(14)	20.0(15)	30.4(15)	1.2(11)	2.6(11)	0.9(11)
N4	17.5(15)	23.2(15)	23.9(14)	0.6(11)	1.0(11)	0.0(12)
N5	16.3(14)	25.3(16)	29.1(15)	1.0(12)	4.6(11)	0.2(12)
N6	27.7(17)	31.5(17)	29.5(16)	2.8(13)	5.3(13)	6.7(13)
C1	15.2(16)	19.2(16)	23.1(16)	0.2(12)	1.8(12)	-1.7(13)
C2	18.9(17)	20.0(17)	25.8(17)	3.2(13)	0.7(13)	-0.5(13)
C3	21.2(17)	21.1(17)	27.6(18)	2.0(13)	-0.6(14)	0.6(14)
C4	15.3(16)	24.7(17)	25.0(17)	-2.8(13)	2.3(13)	-1.8(14)
C5	18.1(17)	18.6(16)	22.1(16)	1.7(12)	1.5(13)	-2.2(13)
C6	15.3(16)	18.0(15)	22.3(16)	-0.4(12)	-0.2(12)	-2.9(13)
C7	25.4(18)	20.1(17)	24.9(17)	2.4(13)	0.0(14)	0.3(14)
C8	21.5(18)	19.0(17)	29.0(18)	1.2(13)	1.2(14)	1.4(13)
C9	15.0(16)	20.8(16)	23.9(17)	-2.5(13)	-0.6(13)	-1.5(13)
C10	13.8(15)	16.6(16)	27.3(17)	-2.9(13)	0.3(13)	-3.7(12)
C11	17.9(17)	19.6(16)	26.5(17)	0.2(13)	0.9(13)	0.0(13)
C12	17.1(16)	22.8(17)	24.2(17)	-0.2(13)	1.7(13)	-1.2(14)
C13	26.1(19)	28.0(19)	34(2)	-3.7(15)	3.9(15)	8.6(16)
C14	22.3(18)	26.6(19)	30.3(18)	0.1(14)	4.1(14)	3.7(15)
C15	14.3(16)	23.7(17)	29.2(18)	-2.7(14)	3.1(13)	4.2(13)

Table 4 Bond Lengths for ojh417S.

Atom	Atom	Length/Å	Atom	Atom	Length/Å
O1	C4	1.355(4)	C1	C2	1.418(4)
O1	C13	1.439(4)	C1	C6	1.428(4)
O2	N5	1.229(4)	C2	C3	1.364(5)
O3	N5	1.235(3)	C3	C4	1.418(4)
N1	C1	1.361(4)	C4	C5	1.376(5)
N1	C9	1.327(4)	C5	C6	1.415(4)
N2	N3	1.350(4)	C6	C7	1.408(4)
N2	C10	1.336(4)	C7	C8	1.368(5)
N3	C11	1.339(4)	C8	C9	1.418(4)
N4	C10	1.354(4)	C9	C10	1.487(4)
N4	C12	1.326(4)	C11	C12	1.410(5)
N5	C5	1.468(4)	C11	C14	1.479(5)
N6	C15	1.144(4)	C12	C15	1.459(5)

Table 5 Bond Angles for ojh417S.

Atom	Atom	Atom	Angle/°	Atom	Atom	Atom	Angle/°
C4	O1	C13	118.5(3)	C5	C6	C1	117.3(3)
C9	N1	C1	117.7(3)	C7	C6	C1	117.9(3)
C10	N2	N3	119.4(3)	C7	C6	C5	124.7(3)
C11	N3	N2	119.3(3)	C8	C7	C6	119.3(3)
C12	N4	C10	113.7(3)	C7	C8	C9	119.0(3)
O2	N5	O3	123.9(3)	N1	C9	C8	123.7(3)
O2	N5	C5	118.4(3)	N1	C9	C10	117.0(3)
O3	N5	C5	117.7(3)	C8	C9	C10	119.3(3)
N1	C1	C2	118.9(3)	N2	C10	N4	125.4(3)
N1	C1	C6	122.4(3)	N2	C10	C9	115.5(3)
C2	C1	C6	118.7(3)	N4	C10	C9	119.1(3)
C3	C2	C1	122.0(3)	N3	C11	C12	118.1(3)
C2	C3	C4	120.3(3)	N3	C11	C14	118.5(3)
O1	C4	C3	124.6(3)	C12	C11	C14	123.4(3)
O1	C4	C5	117.1(3)	N4	C12	C11	124.0(3)
C5	C4	C3	118.3(3)	N4	C12	C15	117.0(3)
C4	C5	N5	118.4(3)	C11	C12	C15	118.9(3)
C4	C5	C6	123.4(3)	N6	C15	C12	176.3(3)
C6	C5	N5	118.2(3)				

Table 6 Torsion Angles for ojh417S.

A	B	C	D	Angle/°	A	B	C	D	Angle/°
O1	C4	C5	N5	0.8(4)	C2	C3	C4	O1	-179.2(3)
O1	C4	C5	C6	179.2(3)	C2	C3	C4	C5	-0.6(5)
O2	N5	C5	C4	-65.9(4)	C3	C4	C5	N5	-177.9(3)
O2	N5	C5	C6	115.6(3)	C3	C4	C5	C6	0.5(5)
O3	N5	C5	C4	113.3(3)	C4	C5	C6	C1	0.1(5)
O3	N5	C5	C6	-65.2(4)	C4	C5	C6	C7	177.2(3)
N1	C1	C2	C3	-179.7(3)	C5	C6	C7	C8	-177.2(3)
N1	C1	C6	C5	179.7(3)	C6	C1	C2	C3	0.6(5)
N1	C1	C6	C7	2.3(5)	C6	C7	C8	C9	-2.3(5)
N1	C9	C10	N2	169.5(3)	C7	C8	C9	N1	2.7(5)
N1	C9	C10	N4	-10.6(4)	C7	C8	C9	C10	-177.1(3)
N2	N3	C11	C12	2.3(4)	C8	C9	C10	N2	-10.7(4)
N2	N3	C11	C14	-179.8(3)	C8	C9	C10	N4	169.2(3)
N3	N2	C10	N4	-2.3(5)	C9	N1	C1	C2	178.2(3)
N3	N2	C10	C9	177.5(3)	C9	N1	C1	C6	-2.1(4)
N3	C11	C12	N4	-2.7(5)	C10	N2	N3	C11	0.0(4)
N3	C11	C12	C15	175.9(3)	C10	N4	C12	C11	0.7(5)

Table 6 Torsion Angles for ojh417S.

A	B	C	D	Angle/°	A	B	C	D	Angle/°
N5	C5	C6	C1	178.5(3)	C10	N4	C12	C15	-178.0(3)
N5	C5	C6	C7	-4.3(5)	C12	N4	C10	N2	1.9(5)
C1	N1	C9	C8	-0.4(5)	C12	N4	C10	C9	-178.0(3)
C1	N1	C9	C10	179.4(3)	C13	O1	C4	C3	-5.4(5)
C1	C2	C3	C4	0.0(5)	C13	O1	C4	C5	176.0(3)
C1	C6	C7	C8	0.0(5)	C14	C11	C12	N4	179.4(3)
C2	C1	C6	C5	-0.6(4)	C14	C11	C12	C15	-1.9(5)
C2	C1	C6	C7	-178.0(3)					

Table 7 Hydrogen Atom Coordinates (Å×10^4) and Isotropic Displacement Parameters (Å^2×10^3) for ojh417S.

Atom	*x*	*y*	*z*	U(eq)
H2	1924.66	7346.2	4219.33	26
H3	378.15	8341.02	5023.24	28
H7	3184.14	2202.01	5961.72	28
H8	4803.99	1417.35	5132.29	28
H13A	-1498.98	8447.75	5744.88	44
H13B	-273.48	9469.5	6136.32	44
H13C	-1516.28	8704.35	6579.79	44
H14A	8624.36	1722.55	2283.25	40
H14B	7456.26	434.47	1993.67	40
H14C	8464.96	-150.98	2625.67	40

X-ray crystallographic analysis of potassium ((2-(benzyloxy)-3,4-dimethoxy-phenyl)ethynyl)trifluoroborate 4

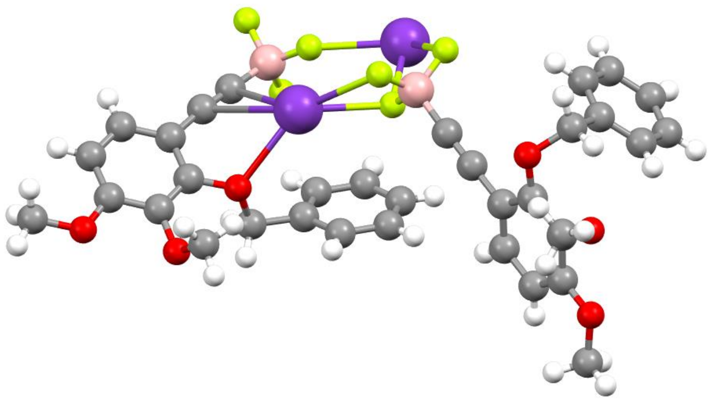

Figure S2. X-ray crystal structure of potassium ((2-(benzyloxy)-3,4-dimethoxy-phenyl)e-thynyl)trifluoroborate.

Crystals of potassium ((2-(benzyloxy)-3,4-dimethoxy-phenyl)ethynyl)trifluoroborate were grown from a saturated acetone solution, allowing slow evaporation.

Empirical formula	$C_{17}H_{15}BF_3KO_3$
Formula weight	374.20
Temperature/K	100.0
Crystal system	triclinic
Space group	P-1
a/Å	7.3233(3)
b/Å	14.6433(6)
c/Å	16.5239(6)
α/°	94.618(2)
β/°	90.430(2)
γ/°	102.959(2)
Volume/Å^3	1720.64(12)
Z	4
ρ_{calc}g/cm^3	1.445
μ/mm^{-1}	3.112
F(000)	768.0
Crystal size/mm^3	0.44 × 0.21 × 0.177
Radiation	CuKα (λ = 1.54178)
2Θ range for data collection/°	5.368 to 133.172
Index ranges	$-8 \leq h \leq 8$, $-17 \leq k \leq 17$, $-19 \leq l \leq 19$
Reflections collected	23433
Independent reflections	5999 [R_{int} = 0.0696, R_{sigma} = 0.0555]
Data/restraints/parameters	5999/0/455
Goodness-of-fit on F^2	1.038

Final R indexes [I>=2σ (I)] R_1 = 0.0432, wR_2 = 0.1054
Final R indexes [all data] R_1 = 0.0554, wR_2 = 0.1140
Largest diff. peak/hole / e $Å^{-3}$ 0.44/-0.59

Table 2 Fractional Atomic Coordinates (×10⁴) and Equivalent Isotropic Displacement Parameters ($Å^2×10^3$) for OJH404v_0m. U_{eq} is defined as 1/3 of of the trace of the orthogonalised U_{IJ} tensor.

Atom	*x*	*y*	*z*	U(eq)
K1	1050.9(7)	9267.5(3)	4107.4(3)	16.18(13)
K2	4403.8(7)	8402.2(4)	5949.3(3)	18.90(14)
F1	6731.5(19)	9474.4(10)	4816.8(8)	21.1(3)
F2	5233(2)	9764.7(10)	3674.9(9)	23.8(3)
F3	3980.4(18)	8502.3(10)	4363.4(8)	20.0(3)
F4	-2258.6(19)	7740.2(10)	5845.9(9)	21.5(3)
F5	-1955(2)	9118.9(10)	6611.1(9)	22.3(3)
F6	183(2)	8971.0(11)	5640.4(9)	26.2(3)
O1	10974(2)	8678.3(11)	2430.4(9)	15.5(3)
O2	12646(2)	7546.3(12)	1313.7(9)	15.3(3)
O3	11140(2)	5723.1(12)	1039.1(10)	18.7(4)
O4	3599(2)	6750.9(11)	6733.6(9)	15.5(3)
O5	5418(2)	5765.1(11)	7728.8(10)	17.1(3)
O6	5458(2)	6109.1(12)	9337.9(10)	18.8(4)
C1	8470(3)	7366.0(16)	2585.5(13)	13.5(4)
C2	10150(3)	7751.5(16)	2203.3(13)	12.4(4)
C3	10992(3)	7186.4(17)	1681.4(13)	14.0(5)
C4	10167(3)	6226.2(16)	1525.0(13)	13.9(5)
C5	8488(3)	5843.3(16)	1887.3(14)	15.1(5)
C6	7664(3)	6410.9(17)	2410.1(14)	15.7(5)
C7	7602(3)	7947.6(16)	3125.0(14)	14.5(5)
C8	6811(3)	8425.7(17)	3563.9(14)	16.1(5)
C9	11441(3)	9308.7(17)	1779.2(14)	17.1(5)
C10	9765(3)	9284.5(16)	1239.3(15)	19.0(5)
C11	8396(4)	9753.7(18)	1501.8(17)	24.8(6)
C12	6858(4)	9737(2)	1002(2)	36.3(7)
C13	6689(5)	9246(2)	240(2)	40.2(8)
C14	8038(5)	8786(2)	-25.6(18)	37.4(7)
C15	9586(4)	8801.8(19)	474.9(16)	26.8(6)
C16	14224(3)	7692(2)	1877.2(16)	23.4(5)
C17	10446(4)	4722.1(17)	946.4(17)	24.0(5)
C18	2417(3)	7482.3(16)	7897.5(14)	15.9(5)
C19	3496(3)	6871.0(16)	7569.0(13)	13.8(4)
C20	4485(3)	6417.7(16)	8058.1(14)	13.6(4)
C21	4461(3)	6591.7(16)	8911.5(14)	14.6(5)

Table 2 Fractional Atomic Coordinates (×10^4) and Equivalent Isotropic Displacement Parameters (Å^2×10^3) for OJH404v_0m. U_{eq} is defined as 1/3 of of the trace of the orthogonalised U_{IJ} tensor.

Atom	*x*	*y*	*z*	U(eq)
C22	3422(3)	7210.8(17)	9246.1(14)	16.8(5)
C23	2411(3)	7645.5(17)	8744.6(14)	16.9(5)
C24	1317(3)	7882.1(16)	7359.3(14)	16.1(5)
C25	352(3)	8149.9(16)	6876.7(14)	16.8(5)
C26	2380(4)	5892.7(18)	6359.9(16)	28.5(6)
C27	2115(4)	6036.7(16)	5481.8(15)	20.4(5)
C28	491(4)	6279.3(18)	5227.1(17)	25.0(5)
C29	223(4)	6430(2)	4422.6(18)	30.0(6)
C30	1567(5)	6332(2)	3868.7(17)	34.6(7)
C31	3203(5)	6107(2)	4115.7(18)	37.8(7)
C32	3483(4)	5954(2)	4922.5(18)	30.1(6)
C33	7183(4)	6181(2)	7397.8(17)	26.0(6)
C34	5511(4)	6280.9(19)	10209.8(14)	21.6(5)
B1	5692(4)	9055.3(19)	4107.7(16)	15.3(5)
B2	-934(4)	8488.1(19)	6234.2(16)	16.3(5)

Table 3 Anisotropic Displacement Parameters (Å^2×10^3) for OJH404v_0m. The Anisotropic displacement factor exponent takes the form: -2π^2[h^{2}a*^{2}U$_{11}$+2hka*b*U$_{12}$+…].

Atom	U_{11}	U_{22}	U_{33}	U_{23}	U_{13}	U_{12}
K1	14.0(2)	17.6(3)	15.0(2)	0.72(18)	-0.26(18)	-0.08(19)
K2	16.4(3)	18.1(3)	21.2(3)	6.54(19)	4.99(19)	-0.10(19)
F1	18.3(7)	23.9(7)	17.1(7)	-4.3(6)	1.1(5)	-1.9(6)
F2	28.0(8)	22.2(7)	23.7(7)	4.2(6)	4.8(6)	10.2(6)
F3	11.7(6)	21.8(7)	24.1(7)	-0.2(6)	6.4(5)	-0.5(5)
F4	17.6(7)	18.8(7)	26.5(7)	2.0(6)	-3.8(6)	0.9(6)
F5	23.5(7)	23.1(7)	21.9(7)	2.3(6)	-0.3(6)	8.5(6)
F6	21.5(7)	34.1(8)	21.4(7)	13.8(6)	4.6(6)	-1.0(6)
O1	14.5(8)	15.3(8)	13.6(8)	-0.8(6)	0.2(6)	-2.4(6)
O2	7.0(7)	22.1(8)	14.5(8)	1.7(6)	2.3(6)	-1.5(6)
O3	15.2(8)	18.1(8)	21.1(9)	-3.1(7)	5.8(7)	2.1(7)
O4	15.0(8)	17.1(8)	11.4(8)	3.0(6)	1.5(6)	-3.0(6)
O5	14.1(8)	18.1(8)	18.5(8)	4.0(6)	6.4(6)	1.3(7)
O6	18.8(8)	24.8(9)	13.9(8)	4.9(7)	1.6(6)	6.4(7)
C1	8.4(10)	18.9(11)	12.4(10)	2.5(9)	1.2(8)	1.1(9)
C2	9.2(10)	16.5(11)	10.0(10)	0.3(8)	-0.1(8)	0.2(9)
C3	8.3(10)	18.9(11)	13.4(11)	4.1(9)	0.6(8)	-0.6(9)
C4	10.5(10)	19.0(12)	12.4(10)	0.0(9)	0.5(8)	3.9(9)
C5	12.7(11)	14.0(11)	16.5(11)	3.1(9)	1.5(9)	-2.0(9)
C6	10.4(10)	20.5(12)	16.3(11)	6.3(9)	3.6(9)	1.8(9)

Table 3 Anisotropic Displacement Parameters (Å^2×10^3) for OJH404v_0m. The Anisotropic displacement factor exponent takes the form: -2π^2[h^{2}a*^{2}U$_{11}$+2hka*b*U$_{12}$+…].

Atom	U11	U22	U33	U23	U13	U12
C7	6.3(10)	18.9(11)	16.8(11)	4.2(9)	-0.1(9)	-1.2(9)
C8	9.6(10)	20.3(12)	16.5(11)	3.2(9)	0.4(9)	-1.0(9)
C9	14.5(11)	16.1(11)	17.8(11)	3.9(9)	4.3(9)	-3.7(9)
C10	21.2(12)	13.9(11)	19.7(12)	6.4(9)	2.2(10)	-2.4(9)
C11	20.7(13)	20.0(13)	32.4(14)	7.1(11)	3.4(11)	0.2(10)
C12	22.2(14)	27.4(15)	60(2)	17.9(14)	-1.6(13)	1.7(12)
C13	34.8(16)	30.1(16)	49.8(19)	17.4(14)	-20.3(14)	-9.7(13)
C14	52.4(19)	25.9(15)	27.0(14)	7.0(12)	-16.3(14)	-6.7(14)
C15	35.9(15)	20.2(13)	21.9(13)	3.4(10)	2.6(11)	0.5(11)
C16	9.5(11)	36.1(15)	23.0(13)	3.9(11)	-0.9(9)	1.6(10)
C17	26.1(13)	16.7(12)	28.7(14)	-0.3(10)	11.6(11)	4.2(10)
C18	10.6(10)	15.6(11)	18.9(11)	4.1(9)	0.3(9)	-3.6(9)
C19	9.4(10)	15.2(11)	13.6(11)	1.8(9)	4.0(8)	-4.2(9)
C20	7.8(10)	14.9(11)	16.6(11)	3.8(9)	3.7(8)	-2.0(8)
C21	9.6(10)	17.0(11)	15.2(11)	4.6(9)	1.2(8)	-2.4(9)
C22	13.0(11)	20.1(12)	15.5(11)	1.5(9)	2.8(9)	-0.2(9)
C23	13.9(11)	18.8(12)	16.7(11)	1.8(9)	4.1(9)	1.0(9)
C24	12.4(11)	15.3(11)	18.3(11)	1.6(9)	4.5(9)	-2.2(9)
C25	12.7(11)	16.9(11)	18.6(11)	2.8(9)	4.7(9)	-1.9(9)
C26	43.8(16)	16.6(12)	17.6(12)	0.7(10)	-6.6(11)	-8.6(12)
C27	28.1(13)	12.5(11)	16.1(12)	-0.4(9)	0.3(10)	-4.5(10)
C28	24.8(13)	19.8(12)	27.8(13)	2.0(10)	1.6(11)	-0.7(10)
C29	28.3(14)	26.5(14)	32.1(15)	4.3(11)	-8.8(12)	-0.9(11)
C30	47.6(18)	32.0(15)	17.8(13)	5.8(11)	-8.4(12)	-5.7(13)
C31	41.3(17)	44.9(18)	23.2(14)	0.3(13)	15.2(13)	2.0(14)
C32	25.3(14)	33.2(15)	31.9(15)	2.0(12)	0.9(11)	7.0(12)
C33	15.5(12)	34.3(15)	30.7(14)	10.6(12)	11.7(10)	7.3(11)
C34	22.5(12)	29.2(13)	13.6(11)	6.5(10)	1.7(9)	5.6(11)
B1	10.1(11)	18.4(13)	16.1(12)	0.1(10)	3.4(10)	1.0(10)
B2	12.8(12)	17.5(13)	17.8(13)	3.8(10)	4.6(10)	0.7(10)

Table 4 Bond Lengths for OJH404v_0m.

Atom	Atom	Length/Å	Atom	Atom	Length/Å
K1	K2[1]	4.2065(7)	C1	C2	1.410(3)
K1	F1[1]	2.6978(14)	C1	C6	1.397(3)
K1	F2	3.0885(15)	C1	C7	1.433(3)
K1	F3	2.6792(15)	C2	C3	1.390(3)
K1	F5[2]	2.6820(15)	C3	C4	1.403(3)
K1	F6	2.6620(14)	C4	C5	1.393(3)

Table 4 Bond Lengths for OJH404v_0m.

Atom	Atom	Length/Å	Atom	Atom	Length/Å
K1	F6^{2}	2.9191(17)	C5	C6	1.385(3)
K1	O1^{3}	2.8316(16)	C7	C8	1.207(3)
K2	F1	2.8625(15)	C8	B1	1.595(3)
K2	F2^{1}	2.6591(15)	C9	C10	1.504(3)
K2	F3	2.6561(15)	C10	C11	1.390(4)
K2	F4^{4}	2.8259(15)	C10	C15	1.387(4)
K2	F5^{4}	2.8151(16)	C11	C12	1.386(4)
K2	O4	2.7839(16)	C12	C13	1.389(5)
F1	B1	1.411(3)	C13	C14	1.371(5)
F2	B1	1.406(3)	C14	C15	1.393(4)
F3	B1	1.419(3)	C18	C19	1.402(3)
F4	B2	1.397(3)	C18	C23	1.401(3)
F5	B2	1.423(3)	C18	C24	1.437(3)
F6	B2	1.413(3)	C19	C20	1.383(3)
O1	C2	1.376(3)	C20	C21	1.414(3)
O1	C9	1.467(3)	C21	C22	1.394(3)
O2	C3	1.375(3)	C22	C23	1.388(3)
O2	C16	1.445(3)	C24	C25	1.207(4)
O3	C4	1.358(3)	C25	B2	1.593(4)
O3	C17	1.435(3)	C26	C27	1.500(3)
O4	C19	1.382(3)	C27	C28	1.387(4)
O4	C26	1.456(3)	C27	C32	1.387(4)
O5	C20	1.375(3)	C28	C29	1.385(4)
O5	C33	1.433(3)	C29	C30	1.372(5)
O6	C21	1.354(3)	C30	C31	1.379(5)
O6	C34	1.441(3)	C31	C32	1.390(4)

^{1}1-X,2-Y,1-Z; ^{2}-X,2-Y,1-Z; ^{3}-1+X,+Y,+Z; ^{4}1+X,+Y,+Z

Table 5 Bond Angles for OJH404v_0m.

Atom	Atom	Atom	Angle/°	Atom	Atom	Atom	Angle/°
F1^{1}	K1	K2^{1}	42.33(3)	O1	C2	C1	116.69(19)
F1^{1}	K1	F2	65.28(4)	O1	C2	C3	122.55(19)
F1^{1}	K1	F6^{2}	67.33(5)	C3	C2	C1	120.5(2)
F1^{1}	K1	O1^{3}	138.27(5)	O2	C3	C2	121.3(2)
F2	K1	K2^{1}	39.11(3)	O2	C3	C4	118.5(2)
F3	K1	K2^{1}	78.02(3)	C2	C3	C4	120.1(2)
F3	K1	F1^{1}	75.93(5)	O3	C4	C3	115.81(19)
F3	K1	F2	45.64(4)	O3	C4	C5	124.4(2)
F3	K1	F5^{2}	113.55(4)	C5	C4	C3	119.8(2)
F3	K1	F6^{2}	142.14(5)	C6	C5	C4	119.7(2)
F3	K1	O1^{3}	90.77(5)	C5	C6	C1	121.7(2)

Table 5 Bond Angles for OJH404v_0m.

Atom	Atom	Atom	Angle/°	Atom	Atom	Atom	Angle/°
F5^{2}	K1	K2^{1}	41.25(3)	C1	C7	K1^{4}	103.03(13)
F5^{2}	K1	F1^{1}	73.73(5)	C8	C7	K1^{4}	79.26(15)
F5^{2}	K1	F2	68.01(4)	C8	C7	C1	177.7(2)
F5^{2}	K1	F6^{2}	47.84(4)	C7	C8	K1^{4}	78.74(15)
F5^{2}	K1	O1^{3}	76.06(5)	C7	C8	B1	177.1(2)
F6^{2}	K1	K2^{1}	68.93(3)	B1	C8	K1^{4}	103.59(14)
F6	K1	K2^{1}	109.77(4)	O1	C9	C10	111.82(18)
F6	K1	F1^{1}	67.44(5)	C11	C10	C9	120.1(2)
F6	K1	F2	118.37(5)	C15	C10	C9	120.2(2)
F6^{2}	K1	F2	107.44(4)	C15	C10	C11	119.7(3)
F6	K1	F3	86.40(5)	C12	C11	C10	120.1(3)
F6	K1	F5^{2}	130.06(5)	C11	C12	C13	119.7(3)
F6	K1	F6^{2}	87.81(5)	C14	C13	C12	120.6(3)
F6	K1	O1^{3}	152.24(5)	C13	C14	C15	119.9(3)
O1^{3}	K1	K2^{1}	96.56(3)	C10	C15	C14	120.1(3)
O1^{3}	K1	F2	77.07(4)	C19	C18	C24	119.1(2)
O1^{3}	K1	F6^{2}	110.43(5)	C23	C18	C19	117.8(2)
F2^{1}	K2	F1	69.16(4)	C23	C18	C24	123.0(2)
F2^{1}	K2	F4^{4}	116.89(5)	O4	C19	C18	118.1(2)
F2^{1}	K2	F5^{4}	72.64(5)	O4	C19	C20	120.1(2)
F2^{1}	K2	O4	138.02(5)	C20	C19	C18	121.7(2)
F3	K2	F1	48.47(4)	O5	C20	C19	121.0(2)
F3	K2	F2^{1}	94.65(5)	O5	C20	C21	119.4(2)
F3	K2	F4^{4}	95.77(4)	C19	C20	C21	119.6(2)
F3	K2	F5^{4}	116.10(4)	O6	C21	C20	115.2(2)
F3	K2	O4	125.08(5)	O6	C21	C22	125.5(2)
F4^{4}	K2	F1	72.84(4)	C22	C21	C20	119.3(2)
F5^{4}	K2	F1	69.28(4)	C23	C22	C21	120.2(2)
F5^{4}	K2	F4^{4}	47.24(4)	C22	C23	C18	121.4(2)
O4	K2	F1	146.53(5)	C25	C24	C18	175.0(2)
O4	K2	F4^{4}	75.86(4)	C24	C25	B2	179.2(3)
O4	K2	F5^{4}	97.52(5)	O4	C26	C27	107.16(19)
K1^{1}	F1	K2	98.27(4)	C28	C27	C26	119.5(2)
B1	F1	K1^{1}	162.28(14)	C28	C27	C32	119.3(2)
B1	F1	K2	97.03(12)	C32	C27	C26	121.3(3)
K2^{1}	F2	K1	93.79(4)	C29	C28	C27	120.7(3)
B1	F2	K1	94.01(12)	C30	C29	C28	119.8(3)
B1	F2	K2^{1}	132.00(14)	C29	C30	C31	120.1(3)
K2	F3	K1	108.72(5)	C30	C31	C32	120.4(3)
B1	F3	K1	112.67(13)	C27	C32	C31	119.7(3)
B1	F3	K2	106.21(13)	F1	B1	F3	106.88(18)
B2	F4	K2^{3}	102.64(13)	F1	B1	C8	111.39(19)
K1^{2}	F5	K2^{3}	99.83(5)	F2	B1	F1	108.9(2)

Table 5 Bond Angles for OJH404v_0m.

Atom	Atom	Atom	Angle/°	Atom	Atom	Atom	Angle/°
B2	F5	K1[2]	107.82(13)	F2	B1	F3	106.88(19)
B2	F5	K2[3]	102.39(13)	F2	B1	C8	111.63(19)
K1	F6	K1[2]	92.19(5)	F3	B1	C8	110.9(2)
B2	F6	K1	152.32(15)	K1[2]	B2	K2[3]	76.24(6)
B2	F6	K1[2]	97.20(13)	F4	B2	K1[2]	119.59(15)
C2	O1	K1[4]	117.01(12)	F4	B2	K2[3]	53.83(11)
C2	O1	C9	117.12(17)	F4	B2	F5	106.53(19)
C9	O1	K1[4]	125.00(13)	F4	B2	F6	108.8(2)
C3	O2	C16	111.23(17)	F4	B2	C25	112.4(2)
C4	O3	C17	117.01(18)	F5	B2	K1[2]	48.70(10)
C19	O4	K2	115.35(13)	F5	B2	K2[3]	53.61(11)
C19	O4	C26	114.50(17)	F5	B2	C25	111.5(2)
C26	O4	K2	121.71(13)	F6	B2	K1[2]	58.44(12)
C20	O5	C33	113.18(18)	F6	B2	K2[3]	112.68(15)
C21	O6	C34	117.14(18)	F6	B2	F5	107.13(19)
C2	C1	C7	120.6(2)	F6	B2	C25	110.24(19)
C6	C1	C2	118.2(2)	C25	B2	K1[2]	127.68(16)
C6	C1	C7	121.3(2)	C25	B2	K2[3]	137.05(15)

[1]1-X,2-Y,1-Z; [2]-X,2-Y,1-Z; [3]-1+X,+Y,+Z; [4]1+X,+Y,+Z

Table 6 Torsion Angles for OJH404v_0m.

A	B	C	D	Angle/°	A	B	C	D	Angle/°
K1[1]	F1	B1	F2	-23.8(5)	O5	C20	C21	O6	2.6(3)
K1[1]	F1	B1	F3	-138.9(4)	O5	C20	C21	C22	-176.13(19)
K1[1]	F1	B1	C8	99.8(4)	O6	C21	C22	C23	-178.2(2)
K1	F2	B1	F1	-107.55(15)	C1	C2	C3	O2	-178.9(2)
K1	F2	B1	F3	7.58(17)	C1	C2	C3	C4	0.5(3)
K1	F2	B1	C8	129.04(17)	C2	O1	C9	C10	54.6(3)
K1	F3	B1	F1	107.04(16)	C2	C1	C6	C5	1.1(3)
K1	F3	B1	F2	-9.5(2)	C2	C1	C7	K1[4]	-40.3(2)
K1	F3	B1	C8	-131.36(16)	C2	C3	C4	O3	-176.9(2)
K1[2]	F5	B2	K2[3]	-104.72(9)	C2	C3	C4	C5	0.8(3)
K1[2]	F5	B2	F4	-115.14(15)	C3	C4	C5	C6	-1.1(3)
K1[2]	F5	B2	F6	1.2(2)	C4	C5	C6	C1	0.2(3)
K1[2]	F5	B2	C25	121.89(16)	C6	C1	C2	O1	-175.76(19)
K1	F6	B2	K1[2]	-108.9(3)	C6	C1	C2	C3	-1.4(3)

Table 6 Torsion Angles for OJH404v_0m.

A	B	C	D	Angle/°	A	B	C	D	Angle/°
K1^{2}	F6	B2	K2^{3}	55.99(12)	C6	C1	C7	K1^{4}	141.04(19)
K1	F6	B2	K2^{3}	-52.9(4)	C7	C1	C2	O1	5.5(3)
K1	F6	B2	F4	4.9(4)	C7	C1	C2	C3	179.9(2)
K1^{2}	F6	B2	F4	113.76(17)	C7	C1	C6	C5	179.8(2)
K1	F6	B2	F5	-109.9(3)	C9	O1	C2	C1	-127.8(2)
K1^{2}	F6	B2	F5	-1.04(19)	C9	O1	C2	C3	58.0(3)
K1^{2}	F6	B2	C25	-122.55(17)	C9	C10	C11	C12	179.5(2)
K1	F6	B2	C25	128.6(3)	C9	C10	C15	C14	-179.6(2)
K1^{4}	O1	C2	C1	42.0(2)	C10	C11	C12	C13	0.3(4)
K1^{4}	O1	C2	C3	-132.18(18)	C11	C10	C15	C14	-0.2(4)
K1^{4}	O1	C9	C10	-114.26(18)	C11	C12	C13	C14	-0.6(4)
K1^{4}	C8	B1	F1	-22.9(2)	C12	C13	C14	C15	0.6(4)
K1^{4}	C8	B1	F2	99.08(18)	C13	C14	C15	C10	-0.1(4)
K1^{4}	C8	B1	F3	-141.84(15)	C15	C10	C11	C12	0.1(4)
K2	F1	B1	F2	125.80(15)	C16	O2	C3	C2	76.3(3)
K2	F1	B1	F3	10.66(18)	C16	O2	C3	C4	-103.2(2)
K2	F1	B1	C8	-110.66(17)	C17	O3	C4	C3	172.8(2)
K2^{1}	F2	B1	F1	-8.8(3)	C17	O3	C4	C5	-4.7(3)
K2^{1}	F2	B1	F3	106.33(19)	C18	C19	C20	O5	174.88(19)
K2^{1}	F2	B1	C8	-132.20(16)	C18	C19	C20	C21	-1.9(3)
K2	F3	B1	F1	-11.9(2)	C19	O4	C26	C27	159.4(2)
K2	F3	B1	F2	-128.39(15)	C19	C18	C23	C22	-0.7(3)
K2	F3	B1	C8	109.71(17)	C19	C20	C21	O6	179.47(19)
K2^{3}	F4	B2	K1^{2}	-41.06(15)	C19	C20	C21	C22	0.7(3)
K2^{3}	F4	B2	F5	10.39(19)	C20	C21	C22	C23	0.5(3)
K2^{3}	F4	B2	F6	-104.81(17)	C21	C22	C23	C18	-0.5(3)
K2^{3}	F4	B2	C25	132.80(16)	C23	C18	C19	O4	-176.17(19)
K2^{3}	F5	B2	K1^{2}	104.72(9)	C23	C18	C19	C20	1.9(3)
K2^{3}	F5	B2	F4	-10.42(19)	C24	C18	C19	O4	6.2(3)
K2^{3}	F5	B2	F6	105.91(17)	C24	C18	C19	C20	-175.7(2)
K2^{3}	F5	B2	C25	-133.39(16)	C24	C18	C23	C22	176.9(2)
K2	O4	C19	C18	48.3(2)	C26	O4	C19	C18	-100.5(3)
K2	O4	C19	C20	-129.81(18)	C26	O4	C19	C20	81.4(3)

Table 6 Torsion Angles for OJH404v_0m.

A	B	C	D	Angle/°	A	B	C	D	Angle/°
K2	O4	C26	C27	12.8(3)	C26	C27	C28	C29	179.0(2)
O1	C2	C3	O2	-4.9(3)	C26	C27	C32	C31	-179.0(3)
O1	C2	C3	C4	174.5(2)	C27	C28	C29	C30	0.5(4)
O1	C9	C10	C11	76.5(3)	C28	C27	C32	C31	-0.6(4)
O1	C9	C10	C15	-104.1(2)	C28	C29	C30	C31	-1.6(4)
O2	C3	C4	O3	2.5(3)	C29	C30	C31	C32	1.6(5)
O2	C3	C4	C5	-179.8(2)	C30	C31	C32	C27	-0.5(5)
O3	C4	C5	C6	176.3(2)	C32	C27	C28	C29	0.6(4)
O4	C19	C20	O5	-7.1(3)	C33	O5	C20	C19	79.3(3)
O4	C19	C20	C21	176.09(19)	C33	O5	C20	C21	-103.9(2)
O4	C26	C27	C28	-99.8(3)	C34	O6	C21	C20	178.57(19)
O4	C26	C27	C32	78.6(3)	C34	O6	C21	C22	-2.8(3)

[1]1-X,2-Y,1-Z; [2]-X,2-Y,1-Z; [3]-1+X,+Y,+Z; [4]1+X,+Y,+Z

Table 7 Hydrogen Atom Coordinates (Å×10^4) and Isotropic Displacement Parameters (Å^2×10^3) for OJH404v_0m.

Atom	*x*	*y*	*z*	U(eq)
H5	7910.01	5195.8	1776.24	18
H6	6521.14	6143.52	2655.77	19
H9A	12439.06	9121.39	1448.49	21
H9B	11931.2	9959.38	2021.55	21
H11	8514.85	10086.16	2024.41	30
H12	5924.71	10059.39	1179.5	44
H13	5629.26	9229.31	-100.83	48
H14	7916.48	8456.16	-549.51	45
H15	10520.15	8481.87	292.55	32
H16A	15384.97	7931.73	1595.84	35
H16B	14279.29	7093.49	2092.11	35
H16C	14078.59	8147.9	2325.56	35
H17A	11351.44	4430.71	649.68	36
H17B	9246.38	4571.03	642.83	36
H17C	10265.65	4480.92	1483.22	36
H22	3405.14	7335.66	9818.83	20
H23	1700.18	8062.27	8980.71	20
H26A	1156.35	5768.1	6629.57	34
H26B	2959.47	5350.13	6410.27	34
H28	-446.39	6342.51	5608.4	30
H29	-889.21	6601.64	4254.5	36
H30	1371.12	6419.97	3313.99	42
H31	4142.63	6055.21	3732.66	45
H32	4607.32	5794.71	5089.87	36

Table 7 Hydrogen Atom Coordinates (Å×10^4) and Isotropic Displacement Parameters (Å^2×10^3) for OJH404v_0m.

Atom	*x*	*y*	*z*	U(eq)
H33A	6975.56	6542.43	6945.47	39
H33B	7813.3	5684.56	7201.69	39
H33C	7969.22	6599.68	7819.78	39
H34A	6134.36	6937.83	10363.95	32
H34B	6205.4	5865.42	10449.54	32
H34C	4228.89	6156.42	10408.77	32

X-Ray crystallographic analysis of (4*S*,10'*R*,11b*R*)-10'-(2-methoxyphenyl)-8',9'-dimethylspiro[dinaphtho[2,1-*d*:1',2'-*f*][1,3,2]dioxaborepine-4,11'-pyrido[3',2':3,4][1,2]azaborolo[1,5-*a*]quinolin]-12'-ium-26-uide 10b

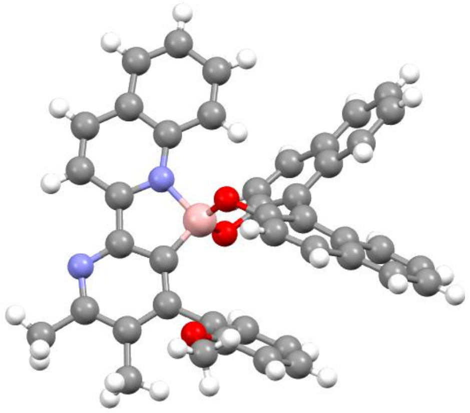

Figure S3. X-ray crystal structure of (4*S*,10'*R*,11b*R*)-10'-(2-methoxyphenyl)-8',9'-dimethylspiro[dinaphtho[2,1-*d*:1',2'-*f*][1,3,2]dioxaborepine-4,11'-pyrido[3',2':3,4][1,2]azaborolo[1,5-a]quinolin]-12'-ium-26-uide.

Crystals of (4*S*,10'*R*,11b*R*)-10'-(2-methoxyphenyl)-8',9'-dimethylspiro[dinaphtho[2,1-*d*:1',2'-*f*][1,3,2]dioxaborepine-4,11'-pyrido[3',2':3,4][1,2]azaborolo[1,5-a]quinolin]-12'-ium-26-uide were grown from a saturated toluene solution, allowing slow evaporation.

Crystal Data for $C_{50}H_{39}BN_2O_3$ (M =726.64 g/mol): monoclinic, space group P2$_1$ (no. 4), a = 6.8740(4) Å, b = 32.7563(18) Å, c = 16.3196(11) Å, β = 92.276(6)°, V = 3671.7(4) Å^3, Z = 4, T = 100.15 K, μ(MoKα) = 0.081 mm^{-1}, $Dcalc$ = 1.314 g/cm^3, 22769 reflections measured (3.524° ≤ 2Θ ≤ 55.054°), 22769 unique (R_{int} = ?, R_{sigma} = 0.0466) which were used in all calculations. The final R_1 was 0.0587 (I > 2σ(I)) and wR_2 was 0.1515 (all data).

Table 1 Crystal data and structure refinement for 2018ncs0765t.

Identification code	2018ncs0765t
Empirical formula	$C_{50}H_{39}BN_2O_3$
Formula weight	726.64
Temperature/K	100.15
Crystal system	monoclinic
Space group	P2$_1$
a/Å	6.8740(4)
b/Å	32.7563(18)
c/Å	16.3196(11)
α/°	90
β/°	92.276(6)
γ/°	90
Volume/Å^3	3671.7(4)
Z	4
ρ_{calc}g/cm^3	1.314
μ/mm^{-1}	0.081
F(000)	1528.0

Crystal size/mm^3	0.12 × 0.025 × 0.02
Radiation	MoKα (λ = 0.71075)
2Θ range for data collection/°	3.524 to 55.054
Index ranges	-8 ≤ h ≤ 8, -42 ≤ k ≤ 42, -21 ≤ l ≤ 21
Reflections collected	22769
Independent reflections	22769 [R_{int} = Merged, R_{sigma} = 0.0466]
Data/restraints/parameters	22769/1/1018
Goodness-of-fit on F^2	1.006
Final R indexes [I>=2σ (I)]	R_1 = 0.0587, wR_2 = 0.1417
Final R indexes [all data]	R_1 = 0.0794, wR_2 = 0.1515
Largest diff. peak/hole / e Å^{-3}	0.35/-0.36
Flack parameter	1.3(8)

Table 2 Fractional Atomic Coordinates (×10^4) and Equivalent Isotropic Displacement Parameters (Å^2×10^3) for 2018ncs0765t. U_{eq} is defined as 1/3 of of the trace of the orthogonalised U_{IJ} tensor.

Atom	*x*	*y*	*z*	U(eq)
O24A	5742(5)	3933.1(10)	7857.8(18)	27.7(7)
O28A	4052(4)	4938.0(9)	5895.2(16)	19.7(7)
O39A	505(4)	4860.7(10)	5977.5(16)	20.3(7)
N2A	2339(5)	5493.8(12)	6562(2)	17.8(8)
N17A	2277(5)	5184.0(12)	8646(2)	19.8(8)
C3A	2364(6)	5817.0(14)	6029(2)	17.1(9)
C4A	2444(7)	5758.7(15)	5174(3)	21.4(10)
C5A	2360(7)	6087.6(16)	4659(3)	24.3(10)
C6A	2275(7)	6488.8(16)	4968(3)	25.0(11)
C7A	2257(7)	6549.9(16)	5783(3)	24.7(10)
C8A	2281(6)	6221.8(15)	6343(3)	19.7(9)
C9A	2214(6)	6279.1(15)	7198(3)	21.3(10)
C10A	2212(6)	5950.7(15)	7708(3)	20.3(10)
C11A	2280(6)	5558.9(14)	7367(2)	18.2(9)
C12A	2311(6)	5177.6(15)	7829(2)	17.9(9)
C13A	2415(6)	4838.7(15)	7320(2)	18.4(9)
C14A	2523(6)	4459.9(15)	7717(2)	17.6(9)
C15A	2449(6)	4450.0(15)	8574(3)	19.0(9)
C16A	2311(6)	4815.3(16)	9014(2)	18.9(9)
C18A	2676(7)	4071.9(15)	7230(3)	23.0(10)
C19A	1197(8)	3965.1(16)	6671(3)	30.7(11)
C20A	1295(8)	3604.6(17)	6218(3)	40.3(14)
C21A	2860(9)	3355.2(17)	6324(3)	38.2(13)
C22A	4373(7)	3452.2(15)	6872(3)	27.0(11)

C23A	4271(7)	3812.5(14)	7322(2)	21.6(10)
C25A	7297(9)	3654(2)	8021(4)	43.3(14)
C26A	2224(7)	4820.7(17)	9928(2)	24.7(10)
C27A	2443(7)	4055.9(16)	9048(3)	25.7(10)
C29A	4285(6)	4586.2(14)	5464(2)	19.2(9)
C30A	5961(7)	4354.6(16)	5645(3)	25.5(10)
C31A	6277(8)	4006.2(17)	5217(3)	32.4(12)
C32A	4934(8)	3861.2(16)	4618(3)	31.4(12)
C33A	5217(9)	3490.6(18)	4187(3)	40.9(14)
C34A	3860(10)	3348.9(18)	3626(3)	44.8(15)
C35A	2137(9)	3566.5(17)	3469(3)	39.9(14)
C36A	1818(8)	3934.0(16)	3861(3)	29.3(11)
C37A	3232(7)	4094.8(15)	4431(3)	25.0(10)
C38A	2965(7)	4480.1(14)	4833(2)	19.9(9)
C40A	152(7)	4920.5(15)	5156(2)	21.1(10)
C41A	-1519(7)	5149.1(15)	4910(3)	25.0(10)
C42A	-1897(7)	5237.8(16)	4108(3)	25.6(11)
C43A	-555(7)	5132.3(15)	3509(3)	24.5(10)
C44A	-791(7)	5259.8(16)	2679(3)	27.1(11)
C45A	594(8)	5176.0(16)	2130(3)	29.3(11)
C46A	2276(8)	4963.7(17)	2376(3)	30.8(12)
C47A	2528(7)	4827.1(16)	3168(3)	24.9(10)
C48A	1103(7)	4897.1(14)	3748(3)	22.8(10)
C49A	1361(7)	4761.6(14)	4589(2)	20.7(9)
B1A	2329(7)	4988.1(16)	6372(3)	18.5(10)
O24B	10584(5)	7294.8(11)	7326.5(18)	27.5(7)
O28B	9126(4)	6250.6(9)	9261.2(16)	20.5(7)
O39B	5579(4)	6328.6(10)	9100.1(16)	18.5(7)
N2B	7363(5)	5703.1(12)	8540(2)	17.3(8)
N17B	7356(5)	6020.5(13)	6461(2)	19.0(8)
C1S	7407(8)	3988.5(17)	1940(3)	31.9(12)
C2S	6220(8)	3732.0(18)	1440(3)	37.2(13)
C3B	7349(6)	5376.3(15)	9071(2)	18.0(9)
C3S	6961(9)	3554.8(17)	755(3)	40.1(13)
C4B	7390(6)	5432.1(16)	9926(3)	22.2(10)
C4S	8848(9)	3620.6(17)	557(3)	37.8(13)
C5B	7236(7)	5098.2(16)	10430(3)	23.7(10)
C5S	10037(8)	3871.9(17)	1052(3)	33.5(12)
C6B	7099(6)	4700.2(16)	10113(3)	23.7(10)
C6S	9303(8)	4047.3(16)	1733(3)	30.8(11)
C7B	7128(7)	4641.5(15)	9288(3)	22.8(10)
C7S	6592(10)	4194(2)	2668(4)	53.9(17)
C8B	7242(6)	4976.3(15)	8742(3)	20.4(10)
C8S	2344(8)	7280.8(17)	3217(3)	32.6(12)
C9B	7225(6)	4921.3(15)	7887(3)	20.1(9)

C9S	3792(8)	7428.5(17)	3755(3)	35.5(12)
C10B	7277(6)	5252.1(15)	7377(3)	19.9(9)
C10S	3338(10)	7600.5(19)	4498(3)	42.6(15)
C11B	7324(6)	5641.1(15)	7730(2)	17.5(9)
C11S	1415(10)	7621.0(18)	4715(3)	42.1(14)
C12B	7349(6)	6023.9(15)	7278(2)	17.0(9)
C12S	-10(9)	7473.0(18)	4191(3)	40.1(14)
C13B	7419(6)	6363.5(15)	7797(2)	17.5(9)
C13S	452(8)	7307.5(17)	3449(3)	33.5(12)
C14B	7485(6)	6742.6(15)	7408(3)	20.4(10)
C14S	2858(10)	7095(2)	2423(3)	55.2(18)
C15B	7408(6)	6755.5(16)	6546(3)	21.8(10)
C16B	7371(6)	6387.2(16)	6097(2)	20.0(10)
C18B	7586(7)	7130.2(14)	7893(2)	21.0(10)
C19B	6120(7)	7225.0(14)	8420(3)	26.2(10)
C20B	6133(8)	7591.1(16)	8852(3)	35.0(13)
C21B	7625(8)	7862.1(16)	8761(3)	31.2(12)
C22B	9134(7)	7775.4(16)	8256(3)	26.7(10)
C23B	9116(7)	7409.6(14)	7826(3)	21.6(10)
C25B	12046(8)	7592.4(18)	7169(3)	35.1(13)
C26B	7351(7)	6383.3(16)	5180(3)	24.3(10)
C27B	7291(8)	7148.2(16)	6076(3)	28.7(11)
C29B	9397(6)	6612.8(14)	9666(2)	19.8(9)
C30B	11060(7)	6840.6(15)	9495(3)	23.8(10)
C31B	11436(7)	7198.9(16)	9900(3)	28.1(11)
C32B	10103(7)	7354.3(16)	10455(3)	28.3(11)
C33B	10413(8)	7738.0(17)	10854(3)	35.6(13)
C34B	9077(9)	7891.9(17)	11362(3)	39.1(13)
C35B	7364(9)	7675.3(17)	11489(3)	35.2(13)
C36B	7038(8)	7306.4(16)	11130(3)	26.9(10)
C37B	8429(7)	7127.1(15)	10622(2)	23.9(10)
C38B	8143(7)	6731.4(15)	10258(2)	21.1(9)
C40B	5311(6)	6284.9(14)	9919(2)	19.6(10)
C41B	3668(7)	6057.2(15)	10149(3)	22.3(10)
C42B	3331(7)	5990.9(16)	10949(3)	23.7(10)
C43B	4682(7)	6122.5(15)	11569(3)	23.4(10)
C44B	4452(7)	6029.4(16)	12412(3)	26.9(11)
C45B	5792(8)	6146.3(16)	12998(3)	29.6(11)
C46B	7464(7)	6354.8(16)	12780(3)	27.7(11)
C47B	7731(7)	6455.6(15)	11977(3)	23.8(10)
C48B	6332(7)	6353.1(15)	11346(2)	21.3(10)
C49B	6541(6)	6457.7(14)	10503(2)	18.4(9)
B1B	7383(7)	6206.0(16)	8746(3)	19.5(11)

Table 3 Anisotropic Displacement Parameters (Å²×10³) for 2018ncs0765t. The Anisotropic displacement factor exponent takes the form: -2π²[h²a*²U11+2hka*b*U12+…].

Atom	U_{11}	U_{22}	U_{33}	U_{23}	U_{13}	U_{12}
O24A	27.7(18)	13.9(19)	40.8(19)	-3.2(14)	-7.0(13)	1.0(15)
O28A	25.5(16)	11.2(17)	22.3(15)	-3.7(12)	1.1(11)	-1.2(13)
O39A	25.6(17)	15.4(18)	19.9(15)	-0.7(12)	-0.1(11)	-0.6(14)
N2A	21(2)	10(2)	22.5(19)	0.0(14)	-0.1(13)	2.0(16)
N17A	20(2)	15(2)	24(2)	-1.0(15)	-1.0(14)	0.3(17)
C3A	17(2)	10(2)	24(2)	-1.2(16)	-0.6(15)	-3.7(18)
C4A	24(3)	12(3)	29(2)	-0.3(17)	4.2(17)	-3(2)
C5A	27(2)	23(3)	23(2)	4.2(18)	2.1(17)	0(2)
C6A	22(2)	16(3)	36(3)	7(2)	1.4(18)	-3(2)
C7A	23(3)	9(3)	42(3)	2.9(19)	-0.5(18)	-1(2)
C8A	12(2)	15(3)	31(2)	0.8(18)	-1.9(16)	-0.5(18)
C9A	19(2)	10(2)	35(3)	-3.8(18)	-1.1(17)	1.4(19)
C10A	20(2)	13(3)	27(2)	-4.1(17)	0.5(16)	3.1(19)
C11A	16(2)	14(3)	24(2)	-2.1(17)	-2.2(15)	-0.5(19)
C12A	15(2)	14(3)	24(2)	-0.7(17)	-1.1(15)	1.7(19)
C13A	17(2)	15(3)	23(2)	-1.9(17)	1.6(15)	1.2(19)
C14A	16(2)	12(2)	25(2)	0.6(17)	-2.0(15)	-1.6(19)
C15A	14(2)	14(2)	29(2)	4.2(18)	0.2(15)	0.0(19)
C16A	10(2)	21(3)	25(2)	0.6(18)	-0.8(15)	-0.1(19)
C18A	35(3)	10(2)	24(2)	3.8(17)	4.1(18)	-1(2)
C19A	40(3)	14(3)	38(3)	1(2)	-10(2)	2(2)
C20A	43(3)	22(3)	54(3)	-8(2)	-25(2)	1(3)
C21A	54(4)	12(3)	47(3)	-8(2)	-9(2)	3(3)
C22A	36(3)	10(3)	35(3)	0.9(18)	-0.8(19)	2(2)
C23A	26(2)	14(2)	25(2)	6.5(18)	2.0(17)	-1(2)
C25A	37(3)	29(3)	62(4)	-12(3)	-15(2)	8(3)
C26A	21(2)	27(3)	26(2)	1.0(19)	2.3(17)	-1(2)
C27A	29(3)	21(3)	27(2)	6.1(19)	-0.2(17)	-2(2)
C29A	23(2)	13(2)	22(2)	1.9(16)	6.6(16)	-0.1(19)
C30A	29(3)	22(3)	26(2)	1.1(18)	0.9(17)	4(2)
C31A	37(3)	28(3)	32(3)	3(2)	0(2)	16(2)
C32A	50(3)	17(3)	28(2)	-1.2(19)	6(2)	7(2)
C33A	63(4)	22(3)	38(3)	-1(2)	2(2)	18(3)
C34A	75(4)	12(3)	47(3)	-7(2)	3(3)	10(3)
C35A	68(4)	18(3)	33(3)	-7(2)	0(2)	-5(3)
C36A	42(3)	15(3)	31(3)	-2.8(19)	1.4(19)	-3(2)
C37A	38(3)	16(3)	22(2)	-0.5(18)	2.1(18)	2(2)
C38A	25(2)	12(2)	22(2)	1.5(17)	3.8(16)	1(2)
C40A	27(2)	16(3)	21(2)	-1.6(17)	0.8(16)	-5(2)
C41A	29(3)	17(3)	29(3)	-1.7(18)	1.5(17)	-2(2)
C42A	28(3)	18(3)	31(3)	1.2(19)	-2.7(18)	2(2)

C43A	30(3)	18(3)	25(2)	-2.1(18)	-4.1(17)	-7(2)
C44A	34(3)	17(3)	29(3)	2.2(18)	-4.2(19)	-6(2)
C45A	44(3)	21(3)	22(2)	3.1(19)	-4.3(19)	-9(2)
C46A	42(3)	28(3)	23(2)	-5(2)	5.5(19)	-11(3)
C47A	26(3)	19(3)	29(2)	-2.9(19)	-0.6(17)	-4(2)
C48A	33(3)	11(2)	24(2)	-3.1(17)	-1.2(17)	-7(2)
C49A	28(2)	12(2)	22(2)	-2.4(17)	-1.8(16)	-3(2)
B1A	22(3)	11(3)	23(2)	0.1(19)	-0.1(18)	2(2)
O24B	29.6(18)	15.9(18)	37.3(19)	-2.4(14)	5.5(13)	-1.0(15)
O28B	27.7(17)	12.9(18)	20.9(15)	-2.0(12)	0.5(11)	-0.8(14)
O39B	25.0(16)	11.3(17)	19.3(15)	-2.2(11)	1.1(11)	-1.9(14)
N2B	17.8(19)	10(2)	24.3(19)	-1.2(14)	1.0(13)	-0.5(15)
N17B	15.3(19)	19(2)	22.9(19)	-0.9(15)	-0.1(13)	-1.5(16)
C1S	43(3)	17(3)	36(3)	0(2)	4(2)	2(2)
C2S	39(3)	26(3)	48(3)	3(2)	3(2)	-6(3)
C3B	14(2)	13(3)	26(2)	2.2(17)	0.0(16)	0.2(18)
C3S	63(4)	20(3)	37(3)	3(2)	-1(2)	-4(3)
C4B	24(2)	14(3)	28(2)	0.8(18)	-3.6(17)	3(2)
C4S	58(4)	19(3)	37(3)	2(2)	12(2)	8(3)
C5B	25(2)	23(3)	23(2)	0.1(18)	-2.2(17)	4(2)
C5S	41(3)	20(3)	40(3)	12(2)	4(2)	4(2)
C6B	16(2)	21(3)	33(3)	9.1(19)	-2.5(17)	-2(2)
C6S	38(3)	22(3)	32(3)	9(2)	-4(2)	-2(2)
C7B	22(2)	9(2)	38(3)	-0.5(18)	-4.4(18)	0(2)
C7S	60(4)	45(4)	58(4)	-12(3)	21(3)	-3(3)
C8B	14(2)	18(3)	29(2)	-0.4(18)	-0.4(16)	0.1(19)
C8S	45(3)	16(3)	36(3)	9(2)	-1(2)	0(2)
C9B	17(2)	11(2)	33(2)	-3.7(17)	2.1(16)	-1.7(19)
C9S	37(3)	22(3)	47(3)	10(2)	0(2)	-4(2)
C10B	20(2)	17(3)	23(2)	-5.2(17)	0.7(16)	-4.1(19)
C10S	60(4)	26(3)	41(3)	4(2)	-12(3)	-8(3)
C11B	15(2)	15(2)	23(2)	0.3(17)	1.2(15)	-0.7(19)
C11S	69(4)	20(3)	38(3)	2(2)	0(3)	6(3)
C12B	10(2)	14(3)	27(2)	-1.3(17)	2.9(15)	-1.1(18)
C12S	45(3)	30(3)	45(3)	11(2)	5(2)	3(3)
C13B	16(2)	16(3)	21(2)	-4.5(17)	2.7(15)	-0.1(19)
C13S	41(3)	20(3)	38(3)	8(2)	-7(2)	-2(2)
C14B	18(2)	19(3)	24(2)	0.5(18)	0.5(16)	-1.3(19)
C14S	64(4)	65(5)	37(3)	-9(3)	0(3)	5(4)
C15B	18(2)	21(3)	26(2)	2.5(18)	1.8(16)	-3(2)
C16B	15(2)	22(3)	23(2)	-1.7(18)	1.5(16)	0(2)
C18B	30(3)	9(2)	23(2)	3.9(17)	-2.4(17)	3(2)
C19B	32(3)	10(3)	37(3)	1.9(18)	7.6(19)	2(2)
C20B	54(3)	16(3)	37(3)	-1(2)	22(2)	4(3)
C21B	52(3)	12(3)	30(3)	-0.4(19)	5(2)	0(2)

C22B	37(3)	16(3)	27(2)	2.6(19)	-1.7(18)	-2(2)
C23B	28(2)	14(3)	22(2)	3.7(17)	-0.8(17)	4(2)
C25B	30(3)	29(3)	47(3)	-5(2)	10(2)	-9(2)
C26B	23(2)	24(3)	26(2)	-0.3(19)	0.3(16)	0(2)
C27B	41(3)	21(3)	24(2)	4.5(19)	1.4(19)	-2(2)
C29B	27(2)	13(2)	19(2)	2.9(16)	-3.5(16)	0(2)
C30B	24(2)	21(3)	26(2)	5.4(18)	-0.2(17)	1(2)
C31B	30(3)	20(3)	34(3)	5(2)	-3.0(19)	-9(2)
C32B	37(3)	20(3)	28(2)	1.8(19)	-10.0(19)	-3(2)
C33B	48(3)	15(3)	42(3)	4(2)	-12(2)	-6(3)
C34B	57(4)	14(3)	45(3)	-4(2)	-12(2)	-3(3)
C35B	52(3)	19(3)	34(3)	-6(2)	-2(2)	8(3)
C36B	38(3)	15(2)	28(2)	-1.9(18)	-1.3(18)	1(2)
C37B	33(3)	17(3)	22(2)	-0.3(18)	-5.6(17)	2(2)
C38B	25(2)	17(2)	20(2)	3.0(17)	-5.7(16)	-2(2)
C40B	24(2)	12(2)	22(2)	-3.0(17)	2.1(16)	4(2)
C41B	28(3)	14(3)	24(2)	-2.8(18)	-3.2(17)	0(2)
C42B	24(2)	18(3)	29(2)	1.1(19)	2.6(17)	-3(2)
C43B	28(3)	16(3)	26(2)	-3.9(18)	3.4(17)	2(2)
C44B	35(3)	20(3)	25(2)	2.7(19)	7.1(18)	-3(2)
C45B	42(3)	22(3)	25(2)	2.4(19)	0.8(19)	3(2)
C46B	35(3)	24(3)	24(2)	-7.1(19)	-5.0(18)	4(2)
C47B	28(3)	15(3)	28(2)	-0.4(18)	0.1(17)	0(2)
C48B	27(2)	14(2)	23(2)	-2.4(17)	1.2(16)	6(2)
C49B	20(2)	9(2)	26(2)	-3.0(16)	4.5(16)	3.3(18)
B1B	26(3)	7(3)	25(3)	-3.5(19)	2.5(19)	-4(2)

Table 4 Bond Lengths for 2018ncs0765t.

Atom	Atom	Length/Å	Atom	Atom	Length/Å
O24A	C23A	1.369(6)	N2B	C11B	1.336(5)
O24A	C25A	1.424(7)	N2B	B1B	1.681(6)
O28A	C29A	1.363(5)	N17B	C12B	1.335(5)
O28A	B1A	1.452(6)	N17B	C16B	1.340(6)
O39A	C40A	1.368(5)	C1S	C2S	1.410(8)
O39A	B1A	1.448(6)	C1S	C6S	1.373(7)
N2A	C3A	1.371(6)	C1S	C7S	1.493(8)
N2A	C11A	1.333(5)	C2S	C3S	1.375(8)
N2A	B1A	1.685(7)	C3B	C4B	1.406(6)
N17A	C12A	1.335(5)	C3B	C8B	1.417(7)
N17A	C16A	1.348(6)	C3S	C4S	1.367(8)
C3A	C4A	1.411(6)	C4B	C5B	1.376(7)
C3A	C8A	1.423(6)	C4S	C5S	1.395(8)
C4A	C5A	1.366(7)	C5B	C6B	1.404(7)

C5A	C6A	1.409(7)	C5S	C6S	1.366(7)
C6A	C7A	1.346(6)	C6B	C7B	1.362(6)
C7A	C8A	1.410(6)	C7B	C8B	1.417(7)
C8A	C9A	1.411(6)	C8B	C9B	1.406(6)
C9A	C10A	1.360(6)	C8S	C9S	1.388(8)
C10A	C11A	1.401(6)	C8S	C13S	1.372(8)
C11A	C12A	1.459(6)	C8S	C14S	1.488(8)
C12A	C13A	1.390(6)	C9B	C10B	1.368(7)
C13A	C14A	1.400(6)	C9S	C10S	1.383(8)
C13A	B1A	1.621(6)	C10B	C11B	1.398(6)
C14A	C15A	1.403(6)	C10S	C11S	1.384(9)
C14A	C18A	1.505(6)	C11B	C12B	1.455(6)
C15A	C16A	1.400(7)	C11S	C12S	1.364(8)
C15A	C27A	1.505(6)	C12B	C13B	1.397(6)
C16A	C26A	1.495(6)	C12S	C13S	1.376(7)
C18A	C19A	1.384(7)	C13B	C14B	1.396(6)
C18A	C23A	1.391(7)	C13B	B1B	1.633(6)
C19A	C20A	1.396(7)	C14B	C15B	1.407(6)
C20A	C21A	1.356(8)	C14B	C18B	1.496(6)
C21A	C22A	1.381(7)	C15B	C16B	1.411(7)
C22A	C23A	1.394(6)	C15B	C27B	1.498(7)
C29A	C30A	1.401(6)	C16B	C26B	1.496(6)
C29A	C38A	1.389(6)	C18B	C19B	1.386(7)
C30A	C31A	1.360(7)	C18B	C23B	1.402(7)
C31A	C32A	1.401(8)	C19B	C20B	1.391(7)
C32A	C33A	1.420(7)	C20B	C21B	1.369(8)
C32A	C37A	1.421(7)	C21B	C22B	1.380(7)
C33A	C34A	1.362(9)	C22B	C23B	1.388(7)
C34A	C35A	1.397(9)	C29B	C30B	1.402(6)
C35A	C36A	1.385(7)	C29B	C38B	1.376(6)
C36A	C37A	1.420(7)	C30B	C31B	1.367(7)
C37A	C38A	1.437(6)	C31B	C32B	1.409(7)
C38A	C49A	1.480(6)	C32B	C33B	1.428(7)
C40A	C41A	1.416(7)	C32B	C37B	1.406(7)
C40A	C49A	1.371(6)	C33B	C34B	1.359(8)
C41A	C42A	1.356(6)	C34B	C35B	1.397(8)
C42A	C43A	1.414(7)	C35B	C36B	1.358(7)
C43A	C44A	1.421(6)	C36B	C37B	1.417(7)
C43A	C48A	1.418(7)	C37B	C38B	1.436(7)
C44A	C45A	1.361(7)	C38B	C49B	1.486(6)
C45A	C46A	1.394(8)	C40B	C41B	1.417(7)
C46A	C47A	1.371(7)	C40B	C49B	1.371(6)
C47A	C48A	1.407(7)	C41B	C42B	1.353(6)
C48A	C49A	1.447(6)	C42B	C43B	1.413(7)
O24B	C23B	1.374(6)	C43B	C44B	1.423(6)

O24B	C25B	1.431(6)	C43B	C48B	1.422(7)
O28B	C29B	1.367(5)	C44B	C45B	1.356(7)
O28B	B1B	1.444(6)	C45B	C46B	1.395(7)
O39B	C40B	1.363(5)	C46B	C47B	1.371(6)
O39B	B1B	1.446(6)	C47B	C48B	1.421(6)
N2B	C3B	1.378(6)	C48B	C49B	1.431(6)

Table 5 Bond Angles for 2018ncs0765t.

Atom	Atom	Atom	Angle/°	Atom	Atom	Atom	Angle/°
C23A	O24A	C25A	117.6(4)	C6S	C1S	C2S	118.3(5)
C29A	O28A	B1A	119.2(3)	C6S	C1S	C7S	121.4(5)
C40A	O39A	B1A	120.8(3)	C3S	C2S	C1S	119.9(5)
C3A	N2A	B1A	130.0(3)	N2B	C3B	C4B	121.5(4)
C11A	N2A	C3A	120.2(4)	N2B	C3B	C8B	118.8(4)
C11A	N2A	B1A	109.8(3)	C4B	C3B	C8B	119.7(4)
C12A	N17A	C16A	115.4(4)	C4S	C3S	C2S	120.8(5)
N2A	C3A	C4A	121.7(4)	C5B	C4B	C3B	119.4(4)
N2A	C3A	C8A	119.3(4)	C3S	C4S	C5S	119.7(5)
C4A	C3A	C8A	119.0(4)	C4B	C5B	C6B	121.5(4)
C5A	C4A	C3A	119.9(4)	C6S	C5S	C4S	119.6(5)
C4A	C5A	C6A	121.1(4)	C7B	C6B	C5B	119.5(4)
C7A	C6A	C5A	119.6(4)	C5S	C6S	C1S	121.7(5)
C6A	C7A	C8A	121.8(5)	C6B	C7B	C8B	121.0(4)
C7A	C8A	C3A	118.4(4)	C7B	C8B	C3B	118.7(4)
C7A	C8A	C9A	122.6(4)	C9B	C8B	C3B	119.5(4)
C9A	C8A	C3A	118.9(4)	C9B	C8B	C7B	121.7(4)
C10A	C9A	C8A	120.1(4)	C9S	C8S	C14S	120.3(5)
C9A	C10A	C11A	118.7(4)	C13S	C8S	C9S	117.9(5)
N2A	C11A	C10A	122.8(4)	C13S	C8S	C14S	121.8(5)
N2A	C11A	C12A	111.8(4)	C10B	C9B	C8B	120.2(4)
C10A	C11A	C12A	125.4(4)	C10S	C9S	C8S	121.0(5)
N17A	C12A	C11A	120.2(4)	C9B	C10B	C11B	118.1(4)
N17A	C12A	C13A	127.8(4)	C9S	C10S	C11S	119.7(5)
C13A	C12A	C11A	112.0(4)	N2B	C11B	C10B	123.0(4)
C12A	C13A	C14A	115.8(4)	N2B	C11B	C12B	111.7(4)
C12A	C13A	B1A	109.1(4)	C10B	C11B	C12B	125.2(4)
C14A	C13A	B1A	135.0(4)	C12S	C11S	C10S	119.4(5)
C13A	C14A	C15A	118.6(4)	N17B	C12B	C11B	120.0(4)
C13A	C14A	C18A	120.6(4)	N17B	C12B	C13B	127.6(4)
C15A	C14A	C18A	120.9(4)	C13B	C12B	C11B	112.3(4)
C14A	C15A	C27A	122.2(4)	C11S	C12S	C13S	120.5(5)
C16A	C15A	C14A	119.8(4)	C12B	C13B	B1B	108.7(4)
C16A	C15A	C27A	117.9(4)	C14B	C13B	C12B	115.7(4)

N17A C16A C15A	122.6(4)	C14B C13B B1B	135.5(4)
N17A C16A C26A	115.6(4)	C8S C13S C12S	121.5(5)
C15A C16A C26A	121.8(4)	C13B C14B C15B	118.7(4)
C19A C18A C14A	119.8(4)	C13B C14B C18B	121.1(4)
C19A C18A C23A	118.2(4)	C15B C14B C18B	120.2(4)
C23A C18A C14A	122.0(4)	C14B C15B C16B	119.5(4)
C18A C19A C20A	120.9(5)	C14B C15B C27B	122.4(4)
C21A C20A C19A	119.7(5)	C16B C15B C27B	118.0(4)
C20A C21A C22A	121.2(5)	N17B C16B C15B	122.5(4)
C21A C22A C23A	118.9(5)	N17B C16B C26B	115.8(4)
O24A C23A C18A	116.9(4)	C15B C16B C26B	121.7(4)
O24A C23A C22A	122.0(4)	C19B C18B C14B	119.9(4)
C18A C23A C22A	121.1(4)	C19B C18B C23B	118.0(4)
O28A C29A C30A	117.5(4)	C23B C18B C14B	122.1(4)
O28A C29A C38A	120.5(4)	C18B C19B C20B	121.2(5)
C38A C29A C30A	121.9(4)	C21B C20B C19B	119.6(5)
C31A C30A C29A	119.5(4)	C20B C21B C22B	121.0(5)
C30A C31A C32A	121.9(5)	C21B C22B C23B	119.2(5)
C31A C32A C33A	122.4(5)	O24B C23B C18B	116.0(4)
C31A C32A C37A	118.8(4)	O24B C23B C22B	123.0(4)
C33A C32A C37A	118.8(5)	C22B C23B C18B	121.0(4)
C34A C33A C32A	121.4(5)	O28B C29B C30B	117.5(4)
C33A C34A C35A	120.1(5)	O28B C29B C38B	120.5(4)
C36A C35A C34A	120.5(5)	C38B C29B C30B	121.9(4)
C35A C36A C37A	120.6(5)	C31B C30B C29B	120.0(4)
C32A C37A C38A	119.6(4)	C30B C31B C32B	120.4(5)
C36A C37A C32A	118.5(4)	C31B C32B C33B	121.5(5)
C36A C37A C38A	121.9(4)	C37B C32B C31B	119.5(5)
C29A C38A C37A	117.8(4)	C37B C32B C33B	119.1(5)
C29A C38A C49A	120.1(4)	C34B C33B C32B	120.8(5)
C37A C38A C49A	122.1(4)	C33B C34B C35B	119.8(5)
O39A C40A C41A	117.6(4)	C36B C35B C34B	120.9(5)
O39A C40A C49A	121.3(4)	C35B C36B C37B	121.2(5)
C49A C40A C41A	121.1(4)	C32B C37B C36B	118.1(5)
C42A C41A C40A	120.6(4)	C32B C37B C38B	119.8(4)
C41A C42A C43A	120.7(5)	C36B C37B C38B	122.1(5)
C42A C43A C44A	122.3(5)	C29B C38B C37B	117.7(4)
C42A C43A C48A	118.8(4)	C29B C38B C49B	120.7(4)
C48A C43A C44A	118.8(4)	C37B C38B C49B	121.6(4)
C45A C44A C43A	120.8(5)	O39B C40B C41B	117.1(4)
C44A C45A C46A	120.3(4)	O39B C40B C49B	122.3(4)
C47A C46A C45A	120.4(4)	C49B C40B C41B	120.6(4)
C46A C47A C48A	121.0(5)	C42B C41B C40B	120.7(4)
C43A C48A C49A	119.6(4)	C41B C42B C43B	120.6(4)
C47A C48A C43A	118.5(4)	C42B C43B C44B	122.1(4)

C47A	C48A	C49A	121.7(4)	C42B	C43B	C48B	119.2(4)
C40A	C49A	C38A	121.3(4)	C48B	C43B	C44B	118.8(4)
C40A	C49A	C48A	118.0(4)	C45B	C44B	C43B	121.5(5)
C48A	C49A	C38A	120.6(4)	C44B	C45B	C46B	120.1(4)
O28A	B1A	N2A	102.2(3)	C47B	C46B	C45B	120.3(4)
O28A	B1A	C13A	118.6(4)	C46B	C47B	C48B	121.5(5)
O39A	B1A	O28A	116.0(4)	C43B	C48B	C49B	119.0(4)
O39A	B1A	N2A	111.2(4)	C47B	C48B	C43B	117.6(4)
O39A	B1A	C13A	109.6(4)	C47B	C48B	C49B	123.3(4)
C13A	B1A	N2A	97.0(3)	C40B	C49B	C38B	120.3(4)
C23B	O24B	C25B	117.2(4)	C40B	C49B	C48B	119.2(4)
C29B	O28B	B1B	117.6(3)	C48B	C49B	C38B	120.4(4)
C40B	O39B	B1B	121.0(3)	O28B	B1B	O39B	116.3(4)
C3B	N2B	B1B	129.4(3)	O28B	B1B	N2B	102.5(4)
C11B	N2B	C3B	120.3(4)	O28B	B1B	C13B	118.5(4)
C11B	N2B	B1B	110.3(3)	O39B	B1B	N2B	110.6(4)
C12B	N17B	C16B	115.8(4)	O39B	B1B	C13B	109.7(4)
C2S	C1S	C7S	120.2(5)	C13B	B1B	N2B	96.9(3)

Table 6 Hydrogen Atom Coordinates (Å×10^4) and Isotropic Displacement Parameters (Å^2×10^3) for 2018ncs0765t.

Atom	*x*	*y*	*z*	U(eq)
H4A	2555.45	5491	4957.28	26
H5A	2359.3	6045.1	4083.36	29
H6A	2231.15	6714.69	4602.22	30
H7A	2225.97	6821.36	5986.58	30
H9A	2169.77	6547.34	7417.66	26
H10A	2165.73	5986.44	8284.74	24
H19A	100.58	4139.41	6593.86	37
H20A	269.62	3534.25	5836.2	48
H21A	2915.88	3109.52	6016.77	46
H22A	5465.67	3276.21	6939.89	32
H25A	8193.45	3767.34	8443.35	65
H25B	7996.08	3606.79	7517.63	65
H25C	6770.49	3394.31	8213.82	65
H	989.86	4701.43	10089.55	37
HA	2316.26	5103.04	10124.31	37
HB	3310.01	4661.36	10168.23	37
H27A	1343.2	4054.65	9412.57	39
H27B	3663.27	4030.35	9375.52	39
H27C	2317.77	3826.05	8665.43	39
H30A	6870.22	4440.31	6063.66	31
H31A	7442.04	3856.71	5327.73	39

H33A	6375.74	3338.23	4292.64	49
H34A	4085.22	3101.55	3341.46	54
H35A	1177.73	3461.61	3090.79	48
H36A	643.43	4079.68	3747.94	35
HC	-2382.63	5241.23	5311.28	30
HD	-3076.4	5372.15	3947.02	31
HE	-1929.15	5405.34	2504.53	33
H45A	415.08	5262.53	1576.72	35
H46A	3253.23	4913.36	1993.46	37
H47A	3681.06	4683.02	3326.64	30
H2S	4909.56	3681.72	1574.84	45
H3S	6151.37	3384.56	415.51	48
HF	7523.27	5698.12	10152.88	27
H4S	9348.76	3495.91	83.45	45
HG	7221.41	5137.74	11006.81	28
HH	11347.87	3920.59	916.8	40
HI	6987.9	4473.67	10471.79	28
HJ	10126.4	4214.51	2073.13	37
H7B	7070.44	4371.75	9074.4	27
H7SA	6700.95	4491.01	2605.59	81
H7SB	7321.63	4108	3165.84	81
H7SC	5219.36	4119.36	2708.51	81
H9B	7176.92	4653.56	7663.62	24
H9S	5115.66	7411.13	3611.72	43
H10B	7281.92	5218.5	6798.54	24
HK	4341.27	7704.2	4856.54	51
HL	1090.9	7737.29	5225.31	51
HM	-1331.05	7484.14	4340.02	48
HN	-562.93	7209.59	3088.53	40
H14A	4247.18	7026.2	2440.21	83
H14B	2576.66	7288.94	1977.34	83
H14C	2089.27	6845.9	2328.01	83
H19B	5088.18	7036.59	8487.67	31
HO	5111.95	7652.76	9207.81	42
HP	7621.65	8113.65	9050.78	37
HQ	10171.47	7963.85	8202.64	32
HR	12963.21	7479.95	6783.86	53
HS	12746.65	7663.62	7684.35	53
HT	11430.18	7837.48	6932.37	53
H26A	6069.22	6470.57	4961.52	36
H26B	7622.13	6106.35	4987.86	36
H26C	8349.82	6570.44	4989.29	36
H27D	6246.66	7130.35	5651.43	43
H27E	8533.28	7198.44	5819.08	43
H27F	7018.05	7372.92	6451.19	43

HU	11924.49	6745.9	9096.54	29
HV	12603.17	7344.03	9806.55	34
HW	11569.15	7887.6	10763.11	43
HX	9305.9	8145.83	11630.15	47
HY	6414.21	7787.67	11831.27	42
HZ	5855.92	7166.05	11220	32
H41B	2794.5	5949.99	9737.88	27
H0AA	2174.9	5854.69	11095.99	28
H1AA	3334.58	5882.12	12566.92	32
H2AA	5591.48	6086.06	13557.31	35
H3AA	8421.13	6427.3	13189.75	33
H4AA	8879.11	6597.15	11838.65	29

X-ray crystallographic analysis of (4*S*,10'*R*,11b*R*)-8',9'-Dimethyl-10'-(2-(methylthio)phenyl)spiro[dinaphtho[2,1-*d*:1',2'-*f*][1,3,2]dioxaborepine-4,11'-pyrido[3',2':3,4][1,2]azaborolo[1,5-*a*]quinolin]-12'-ium-26-uide 10g

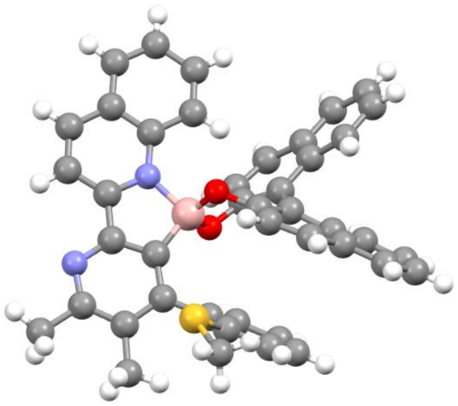

Figure S4. X-ray crystal structure of (4*S*,10'*R*,11b*R*)-8',9'-dimethyl-10'-(2-(methylthio)phenyl)spiro[dinaphtho[2,1-*d*:1',2'-*f*][1,3,2]dioxaborepine-4,11'-pyrido[3',2':3,4][1,2]azaborolo[1,5-*a*]quinolin]-12'-ium-26-uide.

Crystals of (4*S*,10'*R*,11b*R*)-8',9'-dimethyl-10'-(2-(methylthio)phenyl)spiro[dinaphtho[2,1-*d*:1',2'-*f*][1,3,2]dioxaborepine-4,11'-pyrido[3',2':3,4][1,2]azaborolo[1,5-*a*]quinolin]-12'-ium-26-uide were grown from a saturated C_6D_6 solution, allowing slow evaporation.

Crystal Data for $C_{49}H_{37}BN_2O_2S$ (M =728.67 g/mol): orthorhombic, space group $P2_12_12_1$ (no. 19), a = 6.88960(10) Å, b = 15.9833(3) Å, c = 33.7437(6) Å, V = 3715.81(11) Å^3, Z = 4, T = 100.03 K, μ(CuKα) = 1.119 mm^{-1}, $Dcalc$ = 1.303 g/cm^3, 29132 reflections measured (5.238° ≤ 2Θ ≤ 133.212°), 6558 unique (R_{int} = 0.0885, R_{sigma} = 0.0576) which were used in all calculations. The final R_1 was 0.0494 (I > 2σ(I)) and wR_2 was 0.1083 (all data).

Experimental

A suitable single crystal of $C_{49}H_{37}BN_2O_2S$ OJH381v_0m was selected and mounted on a Mitigen microloop in fomblin oil on a Bruker Venture CMOS Photon 100 diffractometer. The crystal was kept at 100 K during data collection. Using Olex2 [1], the structure was solved with the XT [2] structure solution program using Intrinsic Phasing and refined with the XL [3] refinement package using Least Squares minimisation.

1. Dolomanov, O.V., Bourhis, L.J., Gildea, R.J, Howard, J.A.K. & Puschmann, H. (2009), J. Appl. Cryst. 42, 339-341.
2. Sheldrick, G.M. (2015). Acta Cryst. A71, 3-8.
3. Sheldrick, G.M. (2008). Acta Cryst. A64, 112-122.

Table 1 Crystal data and structure refinement for OJH381v_0m.

Identification code	OJH381v_0m
Empirical formula	$C_{49}H_{37}BN_2O_2S$
Formula weight	728.67
Temperature/K	100.03
Crystal system	orthorhombic

Space group	$P2_12_12_1$
a/Å	6.88960(10)
b/Å	15.9833(3)
c/Å	33.7437(6)
α/°	90
β/°	90
γ/°	90
Volume/Å^3	3715.81(11)
Z	4
ρ_{calc}g/cm^3	1.303
μ/mm^{-1}	1.119
F(000)	1528.0
Crystal size/mm^3	0.14 × 0.05 × 0.03
Radiation	CuKα (λ = 1.54178)
2Θ range for data collection/°	5.238 to 133.212
Index ranges	$-8 \leq h \leq 7$, $-19 \leq k \leq 18$, $-40 \leq l \leq 40$
Reflections collected	29132
Independent reflections	6558 [R_{int} = 0.0885, R_{sigma} = 0.0576]
Data/restraints/parameters	6558/0/487
Goodness-of-fit on F^2	1.047
Final R indexes [I>=2σ (I)]	R_1 = 0.0494, wR_2 = 0.1015
Final R indexes [all data]	R_1 = 0.0628, wR_2 = 0.1083
Largest diff. peak/hole / e Å^{-3}	0.53/-0.35
Flack parameter	0.027(14)

Table 2 Fractional Atomic Coordinates (×10^4) and Equivalent Isotropic Displacement Parameters (Å^2×10^3) for OJH381v_0m. U_{eq} is defined as 1/3 of of the trace of the orthogonalised U_{IJ} tensor.

Atom	*x*	*y*	*z*	U(eq)
S1	1507.6(16)	7738.8(7)	3311.7(3)	26.9(2)
O1	7171(4)	5906.0(15)	4222.8(8)	15.1(6)
O2	3616(4)	5785.7(15)	4298.3(8)	16.6(5)
N1	5355(5)	6532.1(18)	4822.6(9)	15.2(6)
N2	5347(5)	8639.8(19)	4482.5(10)	20.9(7)
C1	5338(6)	6011(2)	5148.3(11)	16.8(8)
C2	5307(6)	5133(2)	5105.7(12)	20.6(8)
C3	5416(6)	4634(2)	5435.8(12)	24.2(9)
C4	5475(6)	4975(3)	5819.5(13)	26.3(9)
C5	5424(6)	5821(3)	5868.2(12)	25.2(9)
C6	5368(6)	6361(2)	5536.5(11)	20.1(8)
C7	5375(6)	7247(2)	5575.6(11)	22.5(8)

C8	5376(6)	7739(2)	5247.6(12)	21.5(8)
C9	5357(6)	7365(2)	4873.9(12)	17.9(8)
C10	5340(6)	7796(2)	4491.9(12)	18.0(8)
C11	5277(5)	7253(2)	4172.5(11)	16.3(8)
C12	5244(6)	7621(2)	3794.7(12)	17.9(8)
C13	5322(6)	8503(2)	3769.2(13)	23.1(9)
C14	5347(6)	8974(2)	4120.7(13)	22.1(9)
C15	5451(7)	8958(3)	3380.9(13)	30.9(10)
C16	5370(7)	9920(2)	4109.6(14)	28.9(10)
C17	5120(6)	7089(2)	3427.3(11)	19.2(8)
C18	3512(6)	7117(2)	3171.8(11)	19.9(8)
C19	3468(7)	6625(3)	2830.7(12)	24.9(9)
C20	5023(7)	6106(3)	2743.4(13)	31.5(11)
C21	6602(8)	6068(3)	2992.0(13)	34.1(11)
C22	6652(7)	6555(3)	3332.3(13)	28.6(9)
C23	160(7)	7849(3)	2862.9(14)	42.4(13)
C24	7498(6)	5066(2)	4286.2(11)	14.8(8)
C25	6245(5)	4474(2)	4134.0(11)	15.3(8)
C26	6464(6)	3623(2)	4262.9(11)	17.7(8)
C27	5028(6)	3004(2)	4188.5(12)	22.0(9)
C28	5263(7)	2199(2)	4320.8(12)	24.8(9)
C29	6939(6)	1963(2)	4532.1(13)	25.6(9)
C30	8316(6)	2548(2)	4619.1(12)	23.9(9)
C31	8110(6)	3391(2)	4492.1(11)	19.1(8)
C32	9484(6)	4015(2)	4594.9(12)	22.2(9)
C33	9145(5)	4837(2)	4504.1(12)	18.6(8)
C34	3366(6)	5328(2)	3958.5(11)	16.0(8)
C35	4647(6)	4705(2)	3858.8(11)	16.3(8)
C36	4401(6)	4286(2)	3486.1(12)	19.4(8)
C37	5834(6)	3731(2)	3332.8(12)	24.0(9)
C38	5547(8)	3327(3)	2978.4(13)	32.4(11)
C39	3821(8)	3456(3)	2761.6(13)	38.1(12)
C40	2454(8)	3999(3)	2896.1(14)	34.8(11)
C41	2714(7)	4436(3)	3258.9(13)	26.7(9)
C42	1345(7)	5029(3)	3396.9(13)	28.5(10)
C43	1682(6)	5486(2)	3732.1(13)	23.0(9)
B1	5343(7)	6295(2)	4339.1(12)	15.6(9)
C1S	11273(9)	1980(2)	3434.3(12)	75(2)
C6S	9303(10)	1958(3)	3523.1(13)	94(3)
C5S	8125(5)	1348(4)	3355.0(18)	103(4)
C4S	8916(8)	760(3)	3098.0(15)	97(4)
C3S	10885(9)	782(2)	3009.2(11)	81(3)
C2S	12064(5)	1392(3)	3177.4(14)	67.7(19)

Table 3 Anisotropic Displacement Parameters (Å^2×10^3) for OJH381v_0m. The Anisotropic displacement factor exponent takes the form: -2π^2[h^{2}a*^{2}U$_{11}$+2hka*b*U$_{12}$+…].

Atom	U_{11}	U_{22}	U_{33}	U_{23}	U_{13}	U_{12}
S1	22.0(5)	34.9(5)	23.8(5)	-1.1(4)	-2.6(5)	9.0(4)
O1	11.5(13)	10.0(11)	23.9(14)	-2.6(10)	-0.2(11)	-0.1(10)
O2	14.3(13)	14.2(12)	21.2(13)	-4.9(10)	0.5(12)	-3.4(11)
N1	12.2(15)	13.8(15)	19.6(16)	-1.6(13)	0.2(14)	0.7(13)
N2	14.9(16)	13.6(15)	34.3(19)	-2.7(14)	-5.0(15)	1.5(13)
C1	11.3(18)	18.7(18)	20.5(19)	-0.1(15)	-1.4(16)	3.0(16)
C2	19(2)	19.6(19)	23(2)	-2.4(16)	3.6(18)	-1.4(16)
C3	21(2)	21.2(19)	30(2)	3.0(17)	8(2)	-4.0(18)
C4	22(2)	30(2)	26(2)	8.5(18)	1.4(19)	-3(2)
C5	20(2)	35(2)	21(2)	-3.9(18)	-0.4(19)	-1.8(19)
C6	13.3(18)	27(2)	20.2(19)	-4.9(16)	0.6(17)	0.1(17)
C7	19(2)	27(2)	22(2)	-8.9(17)	-0.5(17)	1.2(17)
C8	17.5(19)	18.9(18)	28(2)	-6.4(17)	-3.5(18)	1.4(17)
C9	9.5(18)	18.0(18)	26(2)	-4.1(16)	-2.2(16)	-0.7(15)
C10	11.9(18)	14.7(17)	27(2)	-2.7(16)	-3.1(17)	4.1(15)
C11	8.2(17)	15.3(17)	25.4(19)	-0.3(16)	-1.8(15)	1.1(15)
C12	10.8(19)	14.4(17)	29(2)	0.3(16)	1.4(17)	1.1(15)
C13	15(2)	16.3(18)	38(2)	4.1(17)	-0.1(19)	-0.5(17)
C14	12.8(19)	14.3(18)	39(2)	1.5(17)	-1.3(19)	1.5(16)
C15	32(2)	23(2)	38(3)	6.3(19)	7(2)	-1(2)
C16	26(2)	13.4(19)	47(3)	1.9(18)	-5(2)	1.9(18)
C17	21(2)	18.3(18)	17.8(19)	4.6(15)	3.9(16)	1.3(16)
C18	22(2)	16.0(18)	22.0(19)	3.7(15)	1.4(18)	2.8(17)
C19	26(2)	30(2)	18(2)	3.0(16)	0.1(19)	-1.4(19)
C20	42(3)	31(2)	22(2)	-2.7(18)	5(2)	8(2)
C21	38(3)	38(2)	27(2)	-3.7(19)	2(2)	17(2)
C22	27(2)	33(2)	26(2)	2.1(19)	-1(2)	11.1(19)
C23	33(3)	66(3)	28(2)	2(2)	-4(2)	23(3)
C24	16.1(19)	12.5(16)	15.9(18)	-4.2(15)	3.7(16)	1.4(15)
C25	12.3(19)	16.8(18)	16.7(18)	-4.0(15)	2.0(16)	1.9(15)
C26	20.5(19)	14.6(17)	18.0(18)	-6.9(14)	1.9(17)	2.3(17)
C27	23(2)	20.3(19)	23(2)	-3.1(16)	1.2(17)	-1.5(17)
C28	32(2)	14.5(18)	28(2)	-5.2(16)	3.0(19)	-5.1(18)
C29	34(3)	11.4(17)	31(2)	-1.4(16)	4(2)	1.0(17)
C30	24(2)	18.8(19)	29(2)	-0.4(16)	-3.5(19)	4.1(17)
C31	21(2)	18.7(18)	17.7(18)	-3.6(15)	0.3(16)	0.9(15)
C32	21(2)	17.8(18)	28(2)	-3.6(16)	-6.1(18)	5.0(17)
C33	15(2)	15.7(18)	25(2)	-1.4(16)	-3.7(16)	-2.0(14)
C34	16.2(19)	13.7(17)	18.0(19)	-0.3(14)	-0.8(17)	-3.6(15)
C35	15.4(19)	13.6(17)	19.8(19)	-1.9(15)	-0.4(17)	-4.2(15)
C36	23(2)	15.8(18)	19.2(19)	0.6(15)	-1.0(17)	-4.2(16)

C37	30(2)	22(2)	20(2)	-3.9(17)	1.8(18)	-1.5(16)
C38	45(3)	30(2)	22(2)	-5.1(19)	3(2)	1(2)
C39	56(3)	39(3)	20(2)	-10(2)	-6(2)	0(2)
C40	45(3)	31(2)	28(2)	-4(2)	-17(2)	-1(2)
C41	34(2)	23(2)	24(2)	-0.2(18)	-8.4(19)	-3.9(18)
C42	25(2)	27(2)	33(2)	1.4(19)	-16(2)	-1.9(19)
C43	17(2)	17.4(18)	34(2)	2.6(16)	-3.5(19)	-0.5(17)
B1	16(2)	15(2)	15(2)	-2.3(16)	-0.1(18)	1.2(17)
C1S	127(7)	55(4)	43(4)	3(3)	-20(4)	-35(4)
C6S	162(9)	59(4)	60(4)	38(4)	59(6)	70(5)
C5S	42(4)	155(9)	112(8)	112(7)	18(5)	25(5)
C4S	94(7)	123(7)	73(5)	77(5)	-41(5)	-69(6)
C3S	149(9)	53(4)	42(3)	-5(3)	2(4)	-16(5)
C2S	41(4)	102(5)	61(4)	-6(4)	12(3)	-5(4)

Table 4 Bond Lengths for OJH381v_0m.

Atom	Atom	Length/Å	Atom	Atom	Length/Å
S1	C18	1.765(4)	C19	C20	1.386(6)
S1	C23	1.785(5)	C20	C21	1.375(7)
O1	C24	1.377(4)	C21	C22	1.388(6)
O1	B1	1.458(5)	C24	C25	1.381(5)
O2	C34	1.371(4)	C24	C33	1.401(5)
O2	B1	1.448(5)	C25	C26	1.435(5)
N1	C1	1.378(5)	C25	C35	1.487(5)
N1	C9	1.342(5)	C26	C27	1.421(5)
N1	B1	1.675(5)	C26	C31	1.422(6)
N2	C10	1.349(5)	C27	C28	1.372(5)
N2	C14	1.333(5)	C28	C29	1.408(6)
C1	C2	1.412(5)	C29	C30	1.364(6)
C1	C6	1.425(5)	C30	C31	1.421(5)
C2	C3	1.372(6)	C31	C32	1.418(5)
C3	C4	1.405(6)	C32	C33	1.369(5)
C4	C5	1.362(6)	C34	C35	1.372(5)
C5	C6	1.415(6)	C34	C43	1.412(5)
C6	C7	1.421(6)	C35	C36	1.434(5)
C7	C8	1.358(6)	C36	C37	1.424(6)
C8	C9	1.396(5)	C36	C41	1.413(6)
C9	C10	1.462(5)	C37	C38	1.373(6)
C10	C11	1.385(5)	C38	C39	1.411(7)
C11	C12	1.404(5)	C39	C40	1.358(7)
C11	B1	1.632(5)	C40	C41	1.421(6)
C12	C13	1.413(5)	C41	C42	1.415(6)
C12	C17	1.505(5)	C42	C43	1.366(6)

C13	C14	1.405(6)	C1S	C6S	1.3900
C13	C15	1.501(6)	C1S	C2S	1.3900
C14	C16	1.512(5)	C6S	C5S	1.3900
C17	C18	1.404(6)	C5S	C4S	1.3900
C17	C22	1.395(6)	C4S	C3S	1.3900
C18	C19	1.395(5)	C3S	C2S	1.3900

Table 5 Bond Angles for OJH381v_0m.

Atom	Atom	Atom	Angle/˚	Atom	Atom	Atom	Angle/˚
C18	S1	C23	103.6(2)	O1	C24	C33	118.0(3)
C24	O1	B1	121.0(3)	C25	C24	C33	121.5(3)
C34	O2	B1	118.8(3)	C24	C25	C26	118.1(3)
C1	N1	B1	129.8(3)	C24	C25	C35	121.7(3)
C9	N1	C1	119.7(3)	C26	C25	C35	120.1(3)
C9	N1	B1	110.5(3)	C27	C26	C25	122.2(4)
C14	N2	C10	115.0(3)	C27	C26	C31	118.0(3)
N1	C1	C2	121.3(3)	C31	C26	C25	119.7(3)
N1	C1	C6	119.7(3)	C28	C27	C26	120.9(4)
C2	C1	C6	119.0(4)	C27	C28	C29	120.8(4)
C3	C2	C1	119.6(4)	C30	C29	C28	119.8(4)
C2	C3	C4	121.6(4)	C29	C30	C31	121.0(4)
C5	C4	C3	119.7(4)	C30	C31	C26	119.4(4)
C4	C5	C6	120.8(4)	C32	C31	C26	118.8(3)
C5	C6	C1	119.2(4)	C32	C31	C30	121.8(4)
C5	C6	C7	122.3(4)	C33	C32	C31	120.4(4)
C7	C6	C1	118.5(4)	C32	C33	C24	120.4(4)
C8	C7	C6	120.1(4)	O2	C34	C35	120.8(3)
C7	C8	C9	119.2(4)	O2	C34	C43	117.4(3)
N1	C9	C8	122.8(4)	C35	C34	C43	121.7(3)
N1	C9	C10	110.8(3)	C34	C35	C25	120.2(3)
C8	C9	C10	126.5(3)	C34	C35	C36	118.5(4)
N2	C10	C9	119.5(3)	C36	C35	C25	121.3(3)
N2	C10	C11	127.5(4)	C37	C36	C35	121.8(4)
C11	C10	C9	113.0(3)	C41	C36	C35	119.6(4)
C10	C11	C12	116.4(3)	C41	C36	C37	118.6(4)
C10	C11	B1	108.7(3)	C38	C37	C36	120.6(4)
C12	C11	B1	134.9(3)	C37	C38	C39	120.2(4)
C11	C12	C13	118.2(4)	C40	C39	C38	120.3(4)
C11	C12	C17	120.8(3)	C39	C40	C41	121.0(4)
C13	C12	C17	121.0(4)	C36	C41	C40	119.2(4)
C12	C13	C15	122.6(4)	C36	C41	C42	118.9(4)
C14	C13	C12	119.0(4)	C42	C41	C40	121.9(4)
C14	C13	C15	118.4(3)	C43	C42	C41	121.1(4)

N2	C14	C13	123.9(3)	C42	C43	C34	119.5(4)
N2	C14	C16	115.0(4)	O1	B1	N1	110.8(3)
C13	C14	C16	121.0(4)	O1	B1	C11	109.3(3)
C18	C17	C12	122.2(3)	O2	B1	O1	116.4(3)
C22	C17	C12	119.5(4)	O2	B1	N1	102.9(3)
C22	C17	C18	118.4(4)	O2	B1	C11	118.1(3)
C17	C18	S1	118.1(3)	C11	B1	N1	97.1(3)
C19	C18	S1	121.4(3)	C6S	C1S	C2S	120.0
C19	C18	C17	120.4(4)	C1S	C6S	C5S	120.0
C20	C19	C18	119.7(4)	C4S	C5S	C6S	120.0
C21	C20	C19	120.6(4)	C5S	C4S	C3S	120.0
C20	C21	C22	119.9(4)	C4S	C3S	C2S	120.0
C21	C22	C17	121.0(4)	C3S	C2S	C1S	120.0
O1	C24	C25	120.5(3)				

Table 6 Hydrogen Atom Coordinates (Å×10[4]) and Isotropic Displacement Parameters (Å[2]×10[3]) for OJH381v_0m.

Atom	*x*	*y*	*z*	U(eq)
H2	5211.29	4888.05	4849.85	25
H3	5451.63	4043.49	5404.33	29
H4	5550.63	4617.69	6043.81	32
H5	5426.46	6049.92	6127.98	30
H7	5379.71	7494.78	5831.47	27
H8	5389.15	8331.09	5272.1	26
H15A	5591.12	8552.11	3165.12	46
H15B	6577.49	9332.07	3383.92	46
H15C	4266.92	9286.98	3340.22	46
H16A	5137.71	10139.55	4376.69	43
H16B	4349.58	10119.87	3930.66	43
H16C	6635.83	10114.17	4014.45	43
H19	2375.99	6644.66	2658.93	30
H20	4997.31	5774.7	2509.78	38
H21	7656.84	5707.29	2931.01	41
H22	7746.5	6524.7	3502.95	34
H23A	1000.62	8082.71	2656.39	64
H23B	-941.91	8225.56	2907.47	64
H23C	-319.46	7300.19	2778.44	64
H27	3890.69	3150	4045.25	26
H28	4282.91	1794.87	4269.2	30
H29	7109.73	1399.3	4613.71	31
H30	9430.84	2389.56	4766.85	29
H32	10646.69	3861.77	4727.69	27
H33	10033.03	5254.69	4589.39	22

H37	6998.22	3639.35	3477.07	29
H38	6514.21	2959.42	2878.87	39
H39	3615.45	3162.88	2520.49	46
H40	1306.78	4086.33	2745.53	42
H42	171.42	5110.11	3254.02	34
H43	790.42	5906.15	3812.32	28
H1S	12077.92	2397.22	3549.24	90
H6S	8762.46	2359.99	3698.7	113
H5S	6778.74	1332.82	3415.64	124
H4S	8110.46	342.88	2983.1	116
H3S	11425.91	380.1	2833.63	98
H2S	13409.66	1407.26	3116.69	81

X-ray crystallographic analysis of 4-(2-methoxyphenyl)-5,6-dimethyl-2-(quinolin-2-yl)pyridin-3-ol 11

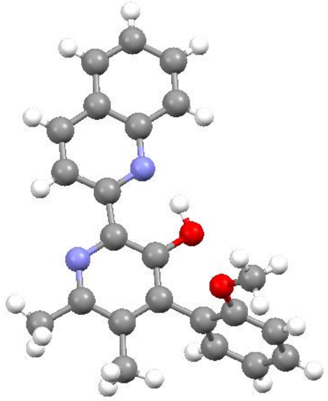

Figure S5. X-ray structure of 4-(2-methoxyphenyl)-5,6-dimethyl-2-(quinolin-2-yl)pyridin-3-ol.

Crystals of 4-(2-methoxyphenyl)-5,6-dimethyl-2-(quinolin-2-yl)pyridin-3-ol were grown from a saturated DCM solution, allowing slow evaporation.

Crystal Data for $C_{23}H_{20}N_2O_2$ (M =356.41 g/mol): triclinic, space group P-1 (no. 2), a = 9.5725(3) Å, b = 10.0172(3) Å, c = 10.6531(3) Å, α = 111.2820(10)°, β = 107.0500(10)°, γ = 91.981(2)°, V = 898.55(5) Å^3, Z = 2, T = 99.99 K, μ(CuKα) = 0.675 mm^{-1}, $Dcalc$ = 1.317 g/cm^3, 27885 reflections measured (9.432° ≤ 2Θ ≤ 133.268°), 3144 unique (R_{int} = 0.0587, R_{sigma} = 0.0269) which were used in all calculations. The final R_1 was 0.0447 (I > 2σ(I)) and wR_2 was 0.1220 (all data).

Table 1 Crystal data and structure refinement for ojh400v_0m.

Identification code	ojh400v_0m
Empirical formula	$C_{23}H_{20}N_2O_2$
Formula weight	356.41
Temperature/K	99.99
Crystal system	triclinic
Space group	P-1
a/Å	9.5725(3)
b/Å	10.0172(3)
c/Å	10.6531(3)
α/°	111.2820(10)
β/°	107.0500(10)
γ/°	91.981(2)
Volume/Å^3	898.55(5)
Z	2
ρ_{calc}g/cm^3	1.317
μ/mm^{-1}	0.675
F(000)	376.0
Crystal size/mm^3	0.25 × 0.24 × 0.06
Radiation	CuKα (λ = 1.54178)

2Θ range for data collection/°	9.432 to 133.268
Index ranges	$-11 \leq h \leq 11$, $-11 \leq k \leq 11$, $-12 \leq l \leq 12$
Reflections collected	27885
Independent reflections	3144 [R_{int} = 0.0587, R_{sigma} = 0.0269]
Data/restraints/parameters	3144/0/251
Goodness-of-fit on F^2	1.035
Final R indexes [I>=2σ (I)]	R_1 = 0.0447, wR_2 = 0.1122
Final R indexes [all data]	R_1 = 0.0586, wR_2 = 0.1220
Largest diff. peak/hole / e Å^{-3}	0.57/-0.24

Table 2 Fractional Atomic Coordinates (×10^4) and Equivalent Isotropic Displacement Parameters (Å^2×10^3) for ojh400v_0m. U_{eq} is defined as 1/3 of of the trace of the orthogonalised U_{IJ} tensor.

Atom	*x*	*y*	*z*	U(eq)
O1	6668.4(13)	3612.2(13)	7743.4(12)	23.8(3)
O2	10038.7(13)	3793.4(12)	7553.2(13)	24.0(3)
N1	5059.7(15)	2594.1(15)	3907.2(14)	19.8(3)
N2	4445.4(15)	1596.2(14)	6645.3(14)	19.5(3)
C1	5285.6(18)	2608.7(17)	5226.4(17)	17.9(4)
C2	6443.4(18)	3564.5(17)	6417.1(17)	19.5(4)
C3	7379.1(18)	4531.5(17)	6228.5(18)	19.9(4)
C4	7135.6(18)	4506.6(17)	4869.9(18)	19.5(4)
C5	5945.8(18)	3508.1(18)	3728.0(18)	20.0(4)
C6	4244.4(18)	1578.9(17)	5348.0(17)	18.6(4)
C7	3077.7(18)	606.2(17)	4133.2(18)	20.1(4)
C8	2138.2(19)	-312.6(18)	4302.2(19)	23.5(4)
C9	2307.5(18)	-303.8(18)	5670.6(18)	21.5(4)
C10	1372(2)	-1209.0(19)	5941(2)	25.9(4)
C11	1613(2)	-1142.3(19)	7292(2)	27.3(4)
C12	2824(2)	-184.3(19)	8443(2)	26.5(4)
C13	3757.8(19)	704.3(19)	8215.8(18)	24.0(4)
C14	3513.2(18)	672.0(17)	6832.0(18)	20.2(4)
C15	8554.2(18)	5606.5(18)	7515.5(17)	20.2(4)
C16	9872.1(19)	5210.6(18)	8178.4(18)	20.7(4)
C17	10927(2)	6229(2)	9391.5(18)	24.9(4)
C18	10667(2)	7641(2)	9952.0(18)	27.1(4)
C19	9366(2)	8049.8(19)	9315.3(19)	26.0(4)
C20	8324.8(19)	7036.7(18)	8102.1(18)	22.1(4)
C21	11372(2)	3357(2)	8191(2)	28.8(4)
C22	8097.6(19)	5509.3(19)	4585.3(18)	23.2(4)
C23	5619.7(19)	3444(2)	2233.8(18)	24.8(4)

Table 3 Anisotropic Displacement Parameters (Å^2×10^3) for ojh400v_0m. The Anisotropic displacement factor exponent takes the form: -2π^2[h^{2}a*^{2}U$_{11}$+2hka*b*U$_{12}$+…].

Atom	U_{11}	U_{22}	U_{33}	U_{23}	U_{13}	U_{12}
O1	23.7(7)	25.7(6)	17.9(6)	8.7(5)	1.5(5)	-3.1(5)
O2	21.2(6)	22.5(6)	26.8(7)	9.6(5)	5.4(5)	6.1(5)
N1	18.0(7)	20.1(7)	19.6(7)	6.6(6)	4.9(6)	6.2(6)
N2	18.1(7)	18.0(7)	21.5(7)	6.9(6)	6.2(6)	3.9(6)
C1	17.1(8)	17.0(8)	18.8(8)	5.9(7)	5.8(6)	5.4(6)
C2	20.2(9)	19.6(8)	17.7(8)	6.6(7)	4.8(6)	7.4(7)
C3	17.5(9)	18.7(8)	21.8(9)	6.8(7)	5.4(7)	5.4(7)
C4	17.7(8)	19.6(8)	22.1(9)	8.0(7)	7.5(7)	6.6(7)
C5	17.4(8)	20.2(8)	22.2(9)	7.5(7)	7.2(7)	5.5(6)
C6	17.0(8)	17.4(8)	20.2(8)	6.0(7)	5.5(6)	7.1(6)
C7	19.9(9)	18.3(8)	18.4(8)	5.2(7)	3.6(7)	2.4(7)
C8	20.1(9)	19.8(8)	23.3(9)	4.6(7)	2.0(7)	1.9(7)
C9	19.8(9)	18.4(8)	25.8(9)	6.3(7)	9.3(7)	5.7(7)
C10	22.4(9)	20.6(9)	31.7(10)	7.8(7)	8.1(7)	1.4(7)
C11	27.3(10)	25.5(9)	33.3(10)	12.1(8)	15.3(8)	3.2(7)
C12	31.5(10)	26.6(9)	26.7(9)	11.4(8)	15.5(8)	8.5(8)
C13	23.0(9)	24.3(9)	22.4(9)	6.9(7)	7.0(7)	4.7(7)
C14	19.5(9)	17.1(8)	24.2(9)	6.7(7)	8.7(7)	6.3(6)
C15	20.3(9)	20.9(8)	20.7(9)	9.0(7)	7.8(7)	1.5(7)
C16	23.2(9)	21.2(8)	19.7(8)	8.3(7)	9.7(7)	2.6(7)
C17	21.9(9)	31.3(9)	20.4(9)	11.1(7)	4.6(7)	-0.8(7)
C18	26.4(10)	28.3(9)	20.2(9)	4.8(7)	5.9(7)	-7.2(7)
C19	30.1(10)	20.0(8)	28.8(10)	6.8(7)	15.0(8)	0.4(7)
C20	21.0(9)	21.0(9)	26.4(9)	10.3(7)	9.4(7)	2.6(7)
C21	20.9(10)	33.0(10)	39.8(11)	21.0(9)	11.3(8)	9.4(8)
C22	21.2(9)	25.4(9)	22.7(9)	9.4(7)	7.0(7)	1.6(7)
C23	21.4(9)	30.0(9)	20.5(9)	8.6(7)	5.3(7)	2.6(7)

Table 4 Bond Lengths for ojh400v_0m.

Atom	Atom	Length/Å	Atom	Atom	Length/Å
O1	C2	1.348(2)	C6	C7	1.426(2)
O2	C16	1.370(2)	C7	C8	1.359(2)
O2	C21	1.424(2)	C8	C9	1.416(2)
N1	C1	1.352(2)	C9	C10	1.412(2)
N1	C5	1.328(2)	C9	C14	1.423(2)
N2	C6	1.332(2)	C10	C11	1.365(3)
N2	C14	1.369(2)	C11	C12	1.415(3)
C1	C2	1.405(2)	C12	C13	1.370(3)

Table 4 Bond Lengths for ojh400v_0m.

Atom	Atom	Length/Å	Atom	Atom	Length/Å
C1	C6	1.475(2)	C13	C14	1.411(2)
C2	C3	1.403(2)	C15	C16	1.401(2)
C3	C4	1.388(2)	C15	C20	1.394(2)
C3	C15	1.498(2)	C16	C17	1.392(2)
C4	C5	1.415(2)	C17	C18	1.385(3)
C4	C22	1.506(2)	C18	C19	1.386(3)
C5	C23	1.506(2)	C19	C20	1.384(2)

Table 5 Bond Angles for ojh400v_0m.

Atom	Atom	Atom	Angle/˚	Atom	Atom	Atom	Angle/˚
C16	O2	C21	117.44(14)	C7	C8	C9	120.51(16)
C5	N1	C1	119.44(14)	C8	C9	C14	117.18(15)
C6	N2	C14	119.85(14)	C10	C9	C8	124.12(16)
N1	C1	C2	121.62(15)	C10	C9	C14	118.70(16)
N1	C1	C6	116.42(14)	C11	C10	C9	120.65(17)
C2	C1	C6	121.95(15)	C10	C11	C12	120.55(16)
O1	C2	C1	122.42(15)	C13	C12	C11	120.28(17)
O1	C2	C3	118.60(15)	C12	C13	C14	120.10(17)
C3	C2	C1	118.96(15)	N2	C14	C9	121.58(15)
C2	C3	C15	118.63(15)	N2	C14	C13	118.71(15)
C4	C3	C2	118.88(15)	C13	C14	C9	119.70(16)
C4	C3	C15	122.42(15)	C16	C15	C3	121.92(15)
C3	C4	C5	118.57(15)	C20	C15	C3	119.55(15)
C3	C4	C22	121.85(15)	C20	C15	C16	118.51(16)
C5	C4	C22	119.58(15)	O2	C16	C15	115.93(15)
N1	C5	C4	122.53(16)	O2	C16	C17	123.57(16)
N1	C5	C23	116.59(15)	C17	C16	C15	120.49(16)
C4	C5	C23	120.88(15)	C18	C17	C16	119.70(17)
N2	C6	C1	116.94(15)	C17	C18	C19	120.60(17)
N2	C6	C7	121.44(15)	C20	C19	C18	119.50(16)
C7	C6	C1	121.62(15)	C19	C20	C15	121.20(16)
C8	C7	C6	119.41(16)				

Table 6 Hydrogen Bonds for ojh400v_0m.

D	H	A	d(D-H)/Å	d(H-A)/Å	d(D-A)/Å	D-H-A/°
O1	H1	N2	1.00(3)	1.60(3)	2.5502(18)	156(2)

Table 7 Torsion Angles for ojh400v_0m.

A	B	C	D	Angle/°	A	B	C	D	Angle/°
O1	C2	C3	C4	-178.79(14)	C6	C1	C2	C3	-179.00(14)
O1	C2	C3	C15	-2.0(2)	C6	C7	C8	C9	0.3(2)
O2	C16	C17	C18	179.95(15)	C7	C8	C9	C10	179.25(16)
N1	C1	C2	O1	178.77(14)	C7	C8	C9	C14	-1.6(2)
N1	C1	C2	C3	0.4(2)	C8	C9	C10	C11	179.61(16)
N1	C1	C6	N2	-179.20(13)	C8	C9	C14	N2	2.0(2)
N1	C1	C6	C7	0.9(2)	C8	C9	C14	C13	-178.52(15)
N2	C6	C7	C8	0.8(2)	C9	C10	C11	C12	-1.3(3)
C1	N1	C5	C4	0.2(2)	C10	C9	C14	N2	-178.83(14)
C1	N1	C5	C23	-179.34(13)	C10	C9	C14	C13	0.7(2)
C1	C2	C3	C4	-0.4(2)	C10	C11	C12	C13	1.0(3)
C1	C2	C3	C15	176.42(14)	C11	C12	C13	C14	0.2(3)
C1	C6	C7	C8	-179.37(14)	C12	C13	C14	N2	178.52(15)
C2	C1	C6	N2	0.2(2)	C12	C13	C14	C9	-1.0(2)
C2	C1	C6	C7	-179.61(14)	C14	N2	C6	C1	179.72(13)
C2	C3	C4	C5	0.3(2)	C14	N2	C6	C7	-0.4(2)
C2	C3	C4	C22	-179.81(14)	C14	C9	C10	C11	0.5(2)
C2	C3	C15	C16	77.7(2)	C15	C3	C4	C5	-176.39(14)
C2	C3	C15	C20	-100.58(18)	C15	C3	C4	C22	3.5(2)
C3	C4	C5	N1	-0.2(2)	C15	C16	C17	C18	0.4(2)
C3	C4	C5	C23	179.36(15)	C16	C15	C20	C19	-0.3(2)
C3	C15	C16	O2	1.9(2)	C16	C17	C18	C19	0.0(3)
C3	C15	C16	C17	-178.57(15)	C17	C18	C19	C20	-0.5(3)
C3	C15	C20	C19	178.05(15)	C18	C19	C20	C15	0.7(3)
C4	C3	C15	C16	-105.65(19)	C20	C15	C16	O2	-179.82(14)
C4	C3	C15	C20	76.1(2)	C20	C15	C16	C17	-0.3(2)
C5	N1	C1	C2	-0.3(2)	C21	O2	C16	C15	179.42(14)
C5	N1	C1	C6	179.10(13)	C21	O2	C16	C17	-0.1(2)
C6	N2	C14	C9	-1.0(2)	C22	C4	C5	N1	179.86(14)
C6	N2	C14	C13	179.50(14)	C22	C4	C5	C23	-0.6(2)
C6	C1	C2	O1	-0.6(2)					

Table 8 Hydrogen Atom Coordinates (Å×10^4) and Isotropic Displacement Parameters (Å^2×10^3) for ojh400v_0m.

Atom	*x*	*y*	*z*	U(eq)
H1	5880(30)	2820(30)	7560(30)	68(8)
H7	2958.28	600.52	3213.87	24
H8	1361.83	-965.85	3495.72	28
H10	566.39	-1871.11	5175.93	31
H11	961.94	-1743.84	7461.76	33
H12	2990.26	-157.19	9376.52	32
H13	4571.04	1342.96	8991.08	29
H17	11822.3	5956.21	9832.92	30
H18	11387.24	8335.76	10780.4	33
H19	9189.11	9017.92	9708.69	31
H20	7438.1	7321.2	7661.05	27
H21A	11344.79	2320.96	7661.21	43
H21B	11470.11	3532.07	9182.37	43
H21C	12217.97	3916.76	8169.48	43
H22A	8527.5	4935.57	3871.55	35
H22B	8892.83	6094.83	5471.32	35
H22C	7498.18	6149.66	4227.78	35
H23A	4802.27	2654.5	1579.79	37
H23B	6501.92	3267.86	1946.61	37
H23C	5342.85	4366.74	2202.63	37

X-ray crystallographic analysis of 5-amino-4-(2-(benzyloxy)-3,4-dimethoxy-phenyl)-6-(6-methoxy-5-nitroquinolin-2-yl)-3-methylpicolinonitrile *rac*-15

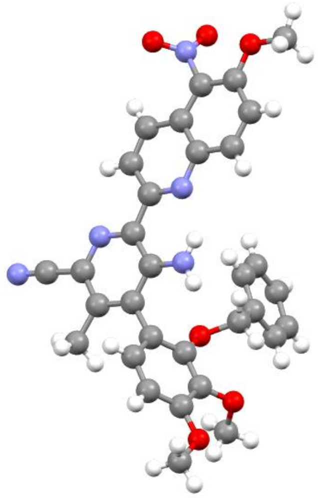

Figure S6. X-ray crystal structure of 5-amino-4-(2-(benzyloxy)-3,4-dimethoxyphenyl)-6-(6-methoxy-5-nitroquinolin-2-yl)-3-methylpicolinonitrile.

Crystals of 5-amino-4-(2-(benzyloxy)-3,4-dimethoxy-phenyl)-6-(6-methoxy-5-nitroquino-lin-2-yl)-3-methylpicolinonitrile were grown from a saturated DCM solution, allowing slow evaporation.

Crystal Data for $C_{32}H_{27}N_5O_6$ (M =577.58 g/mol): triclinic, space group P-1 (no. 2), a = 9.6677(3) Å, b = 12.4797(4) Å, c = 13.6173(4) Å, α = 110.1880(10)°, β = 107.3970(10)°, γ = 102.303(2)°, V = 1376.66(8) Å^3, Z = 2, T = 99.98 K, μ(CuKα) = 0.811 mm^{-1}, $Dcalc$ = 1.393 g/cm^3, 42804 reflections measured (7.57° ≤ 2Θ ≤ 133.184°), 4834 unique (R_{int} = 0.0356, R_{sigma} = 0.0182) which were used in all calculations. The final R_1 was 0.0369 (I > 2σ(I)) and wR_2 was 0.1047 (all data).

Table 1 Crystal data and structure refinement for OJH393v_0m.

Identification code	OJH393v_0m
Empirical formula	$C_{32}H_{27}N_5O_6$
Formula weight	577.58
Temperature/K	99.98
Crystal system	triclinic
Space group	P-1
a/Å	9.6677(3)
b/Å	12.4797(4)
c/Å	13.6173(4)
α/°	110.1880(10)
β/°	107.3970(10)
γ/°	102.303(2)
Volume/Å^3	1376.66(8)
Z	2
ρ_{calc}g/cm^3	1.393

μ/mm^{-1}	0.811
F(000)	604.0
Crystal size/mm^3	0.35 × 0.35 × 0.22
Radiation	CuKα (λ = 1.54178)
2Θ range for data collection/°	7.57 to 133.184
Index ranges	-11 ≤ h ≤ 11, -14 ≤ k ≤ 14, -16 ≤ l ≤ 16
Reflections collected	42804
Independent reflections	4834 [R_{int} = 0.0356, R_{sigma} = 0.0182]
Data/restraints/parameters	4834/0/393
Goodness-of-fit on F^2	1.061
Final R indexes [I>=2σ (I)]	R_1 = 0.0369, wR_2 = 0.1006
Final R indexes [all data]	R_1 = 0.0401, wR_2 = 0.1047
Largest diff. peak/hole / e Å^{-3}	0.26/-0.20

Table 2 Fractional Atomic Coordinates (×10^4) and Equivalent Isotropic Displacement Parameters (Å^2×10^3) for OJH393v_0m. U_{eq} is defined as 1/3 of of the trace of the orthogonalised U_{IJ} tensor.

Atom	*x*	*y*	*z*	U(eq)
O1	-1881.6(11)	4814.1(8)	1233.2(7)	22.1(2)
O2	-2834.9(11)	4516.0(8)	-1018.5(8)	26.3(2)
O3	-3253.5(12)	2426.4(9)	-2706.9(8)	29.0(2)
O4	8853.3(12)	9091.9(9)	8119.5(9)	31.6(2)
O5	6908.6(15)	6982.9(10)	8954.6(9)	42.2(3)
O6	7168.8(13)	8871.3(9)	9383.2(9)	36.2(3)
N1	-100.8(13)	3367.1(10)	4131.9(9)	21.7(2)
N2	-3188.4(15)	1229.6(11)	3919.8(10)	31.2(3)
N3	1415.3(14)	4533.5(11)	2409.3(10)	28.7(3)
N4	3351.1(13)	5616.4(10)	4617.0(9)	22.7(3)
N5	6887.0(14)	7824.1(11)	8696.2(10)	27.3(3)
C1	-1452.9(16)	2530.1(12)	3310.3(11)	21.9(3)
C2	-1988.8(16)	2320.4(12)	2156.0(11)	22.1(3)
C3	-1028.0(16)	3030.0(12)	1862.6(11)	21.9(3)
C4	443.9(16)	3892.2(12)	2704.1(11)	23.0(3)
C5	845.9(16)	4042.9(12)	3857.2(11)	21.8(3)
C6	-2404.9(16)	1821.2(12)	3669.0(11)	24.2(3)
C7	-3539.2(17)	1367.1(13)	1294.0(12)	27.1(3)
C8	-1525.1(15)	2886.3(12)	661.0(11)	22.0(3)
C9	-1917.7(15)	3793.4(11)	383.3(11)	20.4(3)
C10	-2477.7(15)	3613.4(12)	-753.3(11)	21.1(3)
C11	-2679.9(15)	2508.5(12)	-1629.3(11)	23.3(3)
C12	-2296.7(17)	1608.5(13)	-1357.0(11)	26.3(3)
C13	-1714.7(17)	1805.6(12)	-223.0(11)	25.8(3)
C14	-821.0(16)	5979.2(12)	1453.1(12)	26.2(3)
C15	-1154.1(16)	6970.1(11)	2239.9(11)	22.7(3)

Table 2 Fractional Atomic Coordinates (×10⁴) and Equivalent Isotropic Displacement Parameters (Å²×10³) for OJH393v_0m. U_{eq} is defined as 1/3 of of the trace of the orthogonalised U_{IJ} tensor.

Atom	*x*	*y*	*z*	U(eq)
C16	-492.1(17)	7347.7(13)	3415.6(12)	27.9(3)
C17	-830.2(19)	8243.7(14)	4144.7(12)	33.7(3)
C18	-1830.1(19)	8769.3(13)	3708.4(13)	32.9(3)
C19	-2481.6(18)	8409.8(13)	2540.6(13)	31.6(3)
C20	-2139.9(17)	7514.2(13)	1809.9(12)	27.3(3)
C21	-4457.7(19)	4310.0(16)	-1407.1(19)	51.4(5)
C22	-3560(2)	1287.9(14)	-3633.6(12)	35.0(4)
C23	2318.4(16)	4941.8(12)	4823.3(11)	21.9(3)
C24	4701.5(16)	6453.2(12)	5498.5(12)	23.1(3)
C25	5766.5(16)	7164.2(13)	5242.4(12)	26.4(3)
C26	7133.4(17)	8035.9(13)	6084.8(13)	28.6(3)
C27	7518.0(16)	8262.5(12)	7240.5(12)	26.2(3)
C28	6480.2(16)	7572.1(12)	7493.5(12)	24.6(3)
C29	5062.4(16)	6643.1(12)	6647.4(11)	22.7(3)
C30	3932.1(16)	5910.1(12)	6849.2(11)	22.7(3)
C31	2600.1(16)	5073.1(12)	5958.5(11)	22.7(3)
C32	9961.3(17)	9799.3(14)	7871.2(14)	32.3(3)

Table 3 Anisotropic Displacement Parameters (Å²×10³) for OJH393v_0m. The Anisotropic displacement factor exponent takes the form: -2π²[h²a*²U_{11}+2hka*b*U_{12}+…].

Atom	U_{11}	U_{22}	U_{33}	U_{23}	U_{13}	U_{12}
O1	25.8(5)	14.3(4)	21.4(5)	3.6(4)	11.4(4)	2.8(4)
O2	26.4(5)	22.0(5)	26.8(5)	13.1(4)	5.9(4)	5.0(4)
O3	35.4(6)	27.4(5)	15.5(5)	7.7(4)	6.8(4)	3.3(4)
O4	25.5(5)	27.5(5)	30.2(5)	9.7(4)	5.5(4)	1.7(4)
O5	59.7(8)	32.3(6)	25.9(6)	14.1(5)	7.3(5)	12.3(5)
O6	36.3(6)	26.1(5)	28.3(5)	0.2(4)	7.2(5)	6.1(5)
N1	26.6(6)	20.0(5)	18.8(5)	7.9(4)	8.5(5)	10.8(5)
N2	34.2(7)	30.6(7)	27.8(6)	13.0(5)	13.3(5)	8.2(6)
N3	25.5(6)	37.9(7)	17.8(6)	12.8(5)	6.3(5)	4.4(5)
N4	24.4(6)	22.8(6)	20.8(6)	10.0(5)	7.3(5)	9.9(5)
N5	25.2(6)	24.2(6)	22.6(6)	6.3(5)	4.3(5)	4.6(5)
C1	26.6(7)	19.0(6)	19.5(6)	7.0(5)	9.2(5)	9.7(5)
C2	27.3(7)	19.7(6)	18.8(6)	6.1(5)	9.1(5)	11.9(6)
C3	27.5(7)	19.4(6)	18.2(6)	6.3(5)	9.0(5)	11.0(6)
C4	26.2(7)	22.6(7)	21.1(7)	9.1(5)	9.7(5)	10.8(6)
C5	25.3(7)	20.8(6)	20.4(6)	8.8(5)	9.4(6)	10.2(6)
C6	28.0(7)	22.8(7)	17.4(6)	5.6(5)	6.6(5)	10.0(6)
C7	28.8(7)	26.1(7)	20.6(7)	7.5(6)	8.1(6)	6.6(6)

Table 3 Anisotropic Displacement Parameters (Å^2×10^3) for OJH393v_0m. The Anisotropic displacement factor exponent takes the form: -2π^2[h^{2}a*^{2}U$_{11}$+2hka*b*U$_{12}$+…].

Atom	U11	U22	U33	U23	U13	U12
C8	22.7(6)	21.5(7)	18.1(6)	6.7(5)	7.3(5)	6.1(5)
C9	19.6(6)	17.7(6)	18.3(6)	4.0(5)	7.3(5)	3.3(5)
C10	20.0(6)	19.5(6)	21.1(6)	9.2(5)	7.2(5)	2.8(5)
C11	23.2(7)	24.9(7)	17.2(6)	7.8(5)	8.1(5)	2.8(5)
C12	32.1(7)	22.1(7)	19.2(7)	3.6(5)	11.2(6)	7.6(6)
C13	32.7(7)	22.1(7)	22.3(7)	8.3(6)	11.2(6)	11.7(6)
C14	27.4(7)	17.8(6)	26.6(7)	5.5(5)	12.8(6)	0.1(6)
C15	25.2(7)	15.5(6)	21.4(6)	5.5(5)	9.9(5)	0.1(5)
C16	32.1(8)	25.0(7)	24.6(7)	10.7(6)	10.1(6)	8.7(6)
C17	43.3(9)	29.5(8)	20.9(7)	5.9(6)	12.2(6)	9.2(7)
C18	40.2(8)	21.9(7)	32.6(8)	5.6(6)	18.6(7)	8.4(6)
C19	32.3(8)	24.2(7)	39.1(8)	15.4(6)	13.8(7)	9.8(6)
C20	30.1(7)	23.5(7)	22.3(7)	9.7(6)	7.6(6)	3.1(6)
C21	26.8(8)	37.3(9)	77.2(13)	30.4(9)	0.9(8)	7.5(7)
C22	43.6(9)	33.0(8)	16.1(7)	4.6(6)	9.1(6)	5.4(7)
C23	25.0(7)	19.3(6)	21.8(7)	8.6(5)	8.4(6)	11.2(6)
C24	25.4(7)	21.1(6)	22.9(7)	9.6(5)	8.0(5)	11.2(6)
C25	28.6(7)	27.4(7)	24.4(7)	12.6(6)	9.6(6)	11.4(6)
C26	29.0(7)	27.1(7)	31.5(8)	14.8(6)	12.2(6)	10.2(6)
C27	23.9(7)	20.0(7)	28.6(7)	8.3(6)	5.9(6)	7.2(6)
C28	27.7(7)	21.0(7)	23.3(7)	9.0(6)	7.4(6)	10.8(6)
C29	26.8(7)	18.8(6)	22.6(7)	8.8(5)	8.3(6)	11.2(6)
C30	29.7(7)	20.7(6)	17.5(6)	8.6(5)	7.3(5)	11.3(6)
C31	26.7(7)	20.3(6)	21.1(7)	9.2(5)	8.5(6)	9.5(6)
C32	23.7(7)	29.3(8)	38.7(8)	13.5(7)	9.7(6)	6.3(6)

Table 4 Bond Lengths for OJH393v_0m.

Atom	Atom	Length/Å	Atom	Atom	Length/Å
O1	C9	1.3793(15)	C5	C23	1.4822(19)
O1	C14	1.4614(16)	C8	C9	1.4005(19)
O2	C10	1.3749(16)	C8	C13	1.3942(18)
O2	C21	1.4302(19)	C9	C10	1.3951(18)
O3	C11	1.3626(16)	C10	C11	1.4046(18)
O3	C22	1.4346(17)	C11	C12	1.385(2)
O4	C27	1.3498(17)	C12	C13	1.3859(19)
O4	C32	1.4355(18)	C14	C15	1.4974(18)
O5	N5	1.2191(16)	C15	C16	1.3902(19)
O6	N5	1.2285(15)	C15	C20	1.389(2)
N1	C1	1.3373(17)	C16	C17	1.388(2)

Table 4 Bond Lengths for OJH393v_0m.

Atom	Atom	Length/Å	Atom	Atom	Length/Å
N1	C5	1.3351(18)	C17	C18	1.383(2)
N2	C6	1.1514(19)	C18	C19	1.384(2)
N3	C4	1.3455(18)	C19	C20	1.391(2)
N4	C23	1.3256(18)	C23	C31	1.4300(18)
N4	C24	1.3610(18)	C24	C25	1.417(2)
N5	C28	1.4638(18)	C24	C29	1.4171(19)
C1	C2	1.4067(18)	C25	C26	1.366(2)
C1	C6	1.4452(19)	C26	C27	1.412(2)
C2	C3	1.387(2)	C27	C28	1.382(2)
C2	C7	1.5046(19)	C28	C29	1.4166(19)
C3	C4	1.4203(19)	C29	C30	1.426(2)
C3	C8	1.4936(18)	C30	C31	1.3560(19)
C4	C5	1.4325(19)			

Table 5 Bond Angles for OJH393v_0m.

Atom	Atom	Atom	Angle/°	Atom	Atom	Atom	Angle/°
C9	O1	C14	115.73(10)	O3	C11	C10	115.01(12)
C10	O2	C21	112.44(11)	O3	C11	C12	125.30(12)
C11	O3	C22	116.89(11)	C12	C11	C10	119.70(12)
C27	O4	C32	118.21(11)	C11	C12	C13	119.78(12)
C5	N1	C1	119.14(11)	C12	C13	C8	121.67(13)
C23	N4	C24	119.67(11)	O1	C14	C15	107.37(10)
O5	N5	O6	123.94(12)	C16	C15	C14	120.21(13)
O5	N5	C28	118.24(11)	C20	C15	C14	120.79(12)
O6	N5	C28	117.81(12)	C20	C15	C16	119.00(13)
N1	C1	C2	124.50(12)	C17	C16	C15	120.34(14)
N1	C1	C6	115.99(11)	C18	C17	C16	120.31(14)
C2	C1	C6	119.50(12)	C17	C18	C19	119.81(14)
C1	C2	C7	120.70(12)	C18	C19	C20	119.90(14)
C3	C2	C1	116.94(12)	C15	C20	C19	120.63(13)
C3	C2	C7	122.37(12)	N4	C23	C5	119.10(12)
C2	C3	C4	120.02(12)	N4	C23	C31	121.13(12)
C2	C3	C8	120.38(12)	C31	C23	C5	119.77(12)
C4	C3	C8	119.60(12)	N4	C24	C25	117.85(12)
N3	C4	C3	120.20(12)	N4	C24	C29	122.61(13)
N3	C4	C5	121.97(12)	C29	C24	C25	119.54(13)
C3	C4	C5	117.83(12)	C26	C25	C24	121.17(13)
N1	C5	C4	121.48(12)	C25	C26	C27	120.77(13)
N1	C5	C23	115.23(11)	O4	C27	C26	124.42(13)
C4	C5	C23	123.30(12)	O4	C27	C28	117.36(13)
N2	C6	C1	177.87(14)	C28	C27	C26	118.20(13)

Table 5 Bond Angles for OJH393v_0m.

Atom	Atom	Atom	Angle/°	Atom	Atom	Atom	Angle/°
C9	C8	C3	120.94(11)	C27	C28	N5	117.63(12)
C13	C8	C3	120.46(12)	C27	C28	C29	123.07(13)
C13	C8	C9	118.44(12)	C29	C28	N5	119.30(12)
O1	C9	C8	118.95(11)	C24	C29	C30	116.74(12)
O1	C9	C10	120.42(12)	C28	C29	C24	117.23(13)
C10	C9	C8	120.35(12)	C28	C29	C30	125.98(12)
O2	C10	C9	120.09(11)	C31	C30	C29	119.79(12)
O2	C10	C11	119.87(11)	C30	C31	C23	120.06(13)
C9	C10	C11	120.04(12)				

Table 6 Hydrogen Bonds for OJH393v_0m.

D	H	A	d(D-H)/Å	d(H-A)/Å	d(D-A)/Å	D-H-A/°
N3	H3B	N4	0.88	1.99	2.6684(15)	132.4

Table 7 Torsion Angles for OJH393v_0m.

A	B	C	D	Angle/°	A	B	C	D	Angle/°
O1	C9	C10	O2	7.32(18)	C7	C2	C3	C4	-178.15(12)
O1	C9	C10	C11	-172.58(11)	C7	C2	C3	C8	1.30(19)
O1	C14	C15	C16	83.64(15)	C8	C3	C4	N3	-3.19(19)
O1	C14	C15	C20	-95.52(14)	C8	C3	C4	C5	177.09(11)
O2	C10	C11	O3	-0.80(18)	C8	C9	C10	O2	-178.83(11)
O2	C10	C11	C12	179.03(12)	C8	C9	C10	C11	1.27(19)
O3	C11	C12	C13	179.70(13)	C9	O1	C14	C15	170.17(11)
O4	C27	C28	N5	2.06(19)	C9	C8	C13	C12	-0.9(2)
O4	C27	C28	C29	-177.79(12)	C9	C10	C11	O3	179.11(11)
O5	N5	C28	C27	-124.61(14)	C9	C10	C11	C12	-1.1(2)
O5	N5	C28	C29	55.25(18)	C10	C11	C12	C13	-0.1(2)
O6	N5	C28	C27	56.23(17)	C11	C12	C13	C8	1.1(2)
O6	N5	C28	C29	-123.91(14)	C13	C8	C9	O1	173.65(12)
N1	C1	C2	C3	0.66(19)	C13	C8	C9	C10	-0.29(19)
N1	C1	C2	C7	-179.20(12)	C14	O1	C9	C8	119.99(13)
N1	C5	C23	N4	177.37(11)	C14	O1	C9	C10	-66.07(15)

Table 7 Torsion Angles for OJH393v_0m.

A	B	C	D	Angle/°	A	B	C	D	Angle/°
N1	C5	C23	C31	-2.97(18)	C14	C15	C16	C17	-178.32(13)
N3	C4	C5	N1	-177.27(12)	C14	C15	C20	C19	178.14(13)
N3	C4	C5	C23	2.6(2)	C15	C16	C17	C18	0.0(2)
N4	C23	C31	C30	0.9(2)	C16	C15	C20	C19	-1.0(2)
N4	C24	C25	C26	178.88(12)	C16	C17	C18	C19	-0.8(2)
N4	C24	C29	C28	-177.74(12)	C17	C18	C19	C20	0.6(2)
N4	C24	C29	C30	-0.10(19)	C18	C19	C20	C15	0.3(2)
N5	C28	C29	C24	178.47(11)	C20	C15	C16	C17	0.9(2)
N5	C28	C29	C30	1.1(2)	C21	O2	C10	C9	-99.00(16)
C1	N1	C5	C4	0.10(19)	C21	O2	C10	C11	80.90(17)
C1	N1	C5	C23	-179.78(11)	C22	O3	C11	C10	-176.62(12)
C1	C2	C3	C4	2.00(18)	C22	O3	C11	C12	3.6(2)
C1	C2	C3	C8	-178.56(11)	C23	N4	C24	C25	-179.35(11)
C2	C3	C4	N3	176.26(12)	C23	N4	C24	C29	-0.1(2)
C2	C3	C4	C5	-3.46(19)	C24	N4	C23	C5	179.38(11)
C2	C3	C8	C9	109.31(15)	C24	N4	C23	C31	-0.28(19)
C2	C3	C8	C13	-66.06(18)	C24	C25	C26	C27	-0.6(2)
C3	C4	C5	N1	2.43(19)	C24	C29	C30	C31	0.73(19)
C3	C4	C5	C23	-177.70(11)	C25	C24	C29	C28	1.49(19)
C3	C8	C9	O1	-1.81(19)	C25	C24	C29	C30	179.13(12)
C3	C8	C9	C10	-175.75(12)	C25	C26	C27	O4	178.87(13)
C3	C8	C13	C12	174.58(13)	C25	C26	C27	C28	0.5(2)
C4	C3	C8	C9	-71.24(17)	C26	C27	C28	N5	-179.45(12)
C4	C3	C8	C13	113.39(15)	C26	C27	C28	C29	0.7(2)
C4	C5	C23	N4	-2.5(2)	C27	C28	C29	C24	-1.7(2)
C4	C5	C23	C31	177.16(12)	C27	C28	C29	C30	-179.07(12)
C5	N1	C1	C2	-1.73(19)	C28	C29	C30	C31	178.13(12)
C5	N1	C1	C6	179.08(11)	C29	C24	C25	C26	-0.4(2)
C5	C23	C31	C30	-178.74(12)	C29	C30	C31	C23	-1.12(19)
C6	C1	C2	C3	179.83(12)	C32	O4	C27	C26	0.3(2)
C6	C1	C2	C7	-0.03(19)	C32	O4	C27	C28	178.65(12)

Table 8 Hydrogen Atom Coordinates (Å×10^4) and Isotropic Displacement Parameters (Å^2×10^3) for OJH393v_0m.

Atom	*x*	*y*	*z*	U(eq)
H3A	1139.33	4419.36	1693.35	34
H3B	2326.63	5067.08	2932.5	34
H7A	-4035.9	1661.06	751.78	41
H7B	-4196.26	1206.18	1690.24	41
H7C	-3395.13	613.1	878.51	41
H12	-2432.37	858.58	-1945.58	32
H13	-1438	1188.76	-43.5	31
H14A	-982.62	6046.43	726.8	31
H14B	266.39	6044.34	1813.26	31
H16	195.24	6990.62	3721.55	33
H17	-372.72	8497.16	4947.15	40
H18	-2068.78	9375.25	4209	40
H19	-3161.26	8774.15	2238.47	38
H20	-2585.03	7272.47	1009.01	33
H21A	-5021.66	3556.91	-2121.56	77
H21B	-4647.87	4998.1	-1541.65	77
H21C	-4815.68	4233.91	-824.51	77
H22A	-3989.11	1331.43	-4362.37	53
H22B	-4306.83	629.84	-3621	53
H22C	-2593.97	1124.85	-3550.53	53
H25	5525.39	7032.09	4471.03	32
H26	7833.48	8495.19	5890.78	34
H30	4111.75	6007.84	7605.35	27
H31	1853.25	4573.56	6088.78	27
H32A	10283.51	9252.67	7346.3	48
H32B	10867.17	10361.44	8583.47	48
H32C	9489.81	10266.56	7514.41	48

Chiral HPLC Chromatograms

4-(2-Methoxyphenyl)-5,6-dimethyl-2-(quinolin-2-yl)pyridin-3-ol (11)

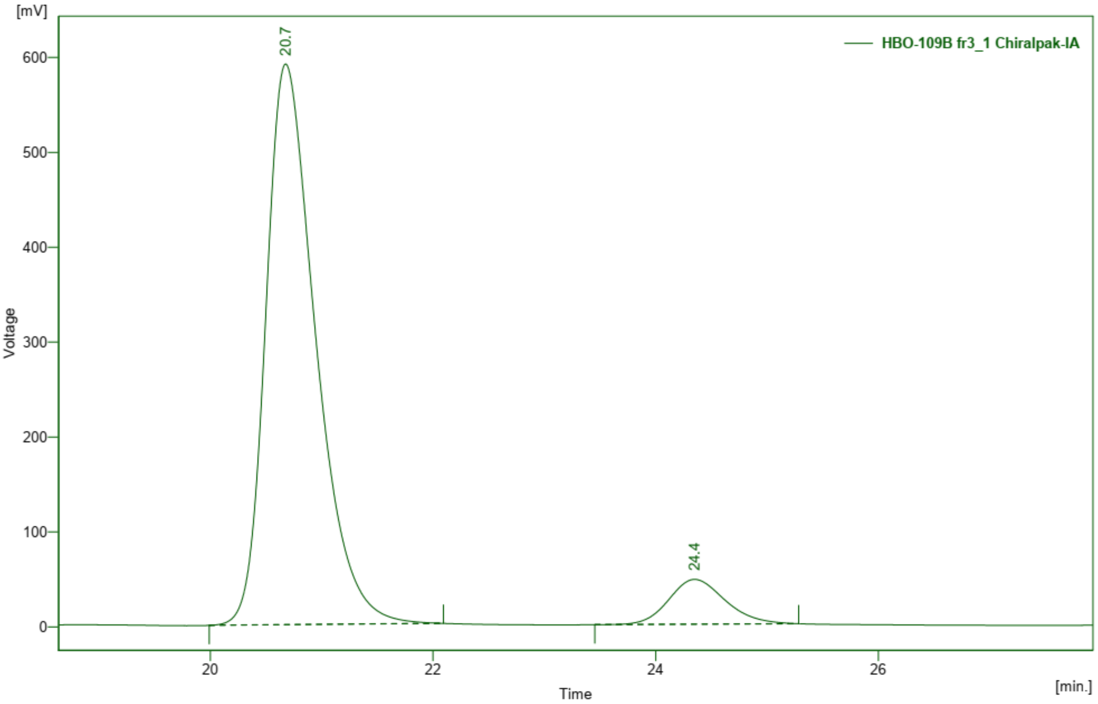

Result Table - Calculation Method Uncal

	Reten. Time [min]	Area [mV.s]	Height [mV]	Area [%]	Height [%]	W05 [min]
1	20.677	18369.636	590.983	91.7	92.6	0.47
2	24.350	1666.430	47.282	8.3	7.4	0.54
	Total	20036.065	638.264	100.0	100.0	

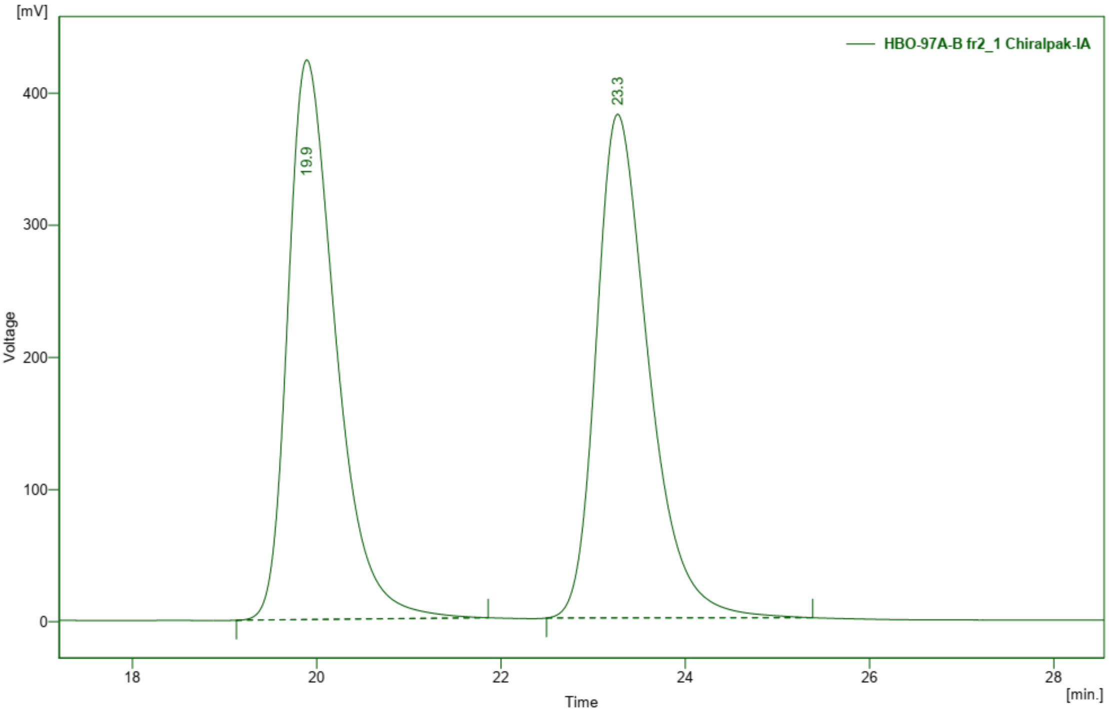

Result Table - Calculation Method Uncal

	Reten. Time [min]	Area [mV.s]	Height [mV]	Area [%]	Height [%]	W05 [min]
1	19.890	15293.819	423.493	50.4	52.6	0.54
2	23.267	15080.449	381.136	49.6	47.4	0.59
	Total	30374.268	804.628	100.0	100.0	

4-(2-Isopropoxyphenyl)-5,6-dimethyl-2-(quinolin-2-yl)pyridin-3-ol (12)

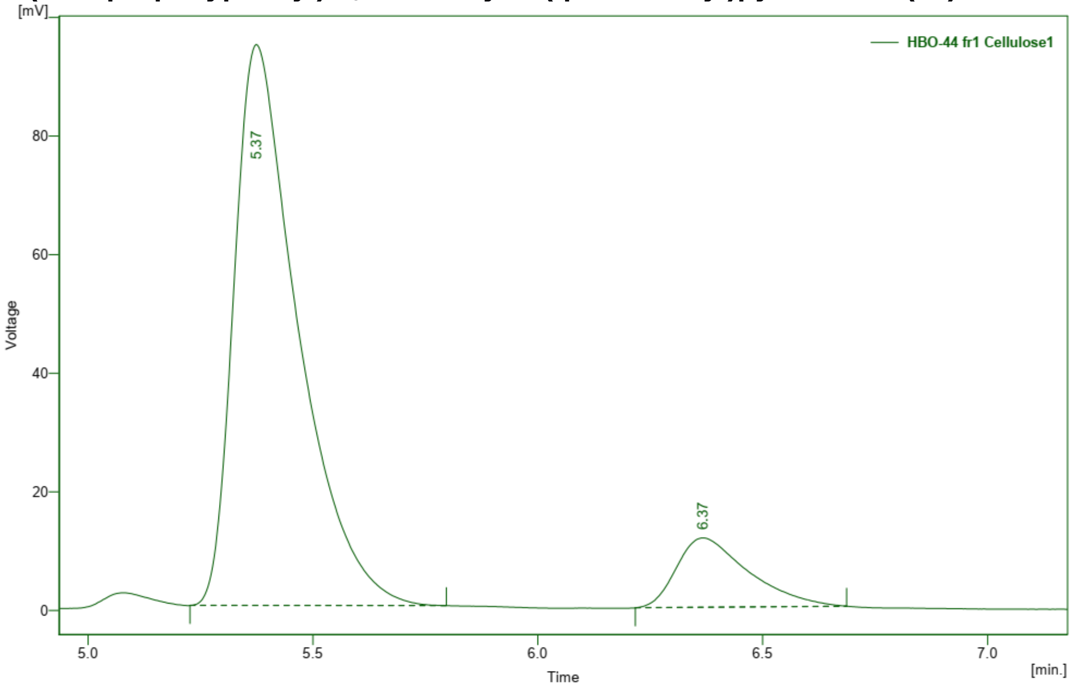

Result Table - Calculation Method Uncal

	Reten. Time [min]	Area [mV.s]	Height [mV]	Area [%]	Height [%]	W05 [min]
1	5.373	934.015	94.588	87.8	89.0	0.15
2	6.367	129.920	11.699	12.2	11.0	0.17
	Total	1063.936	106.287	100.0	100.0	

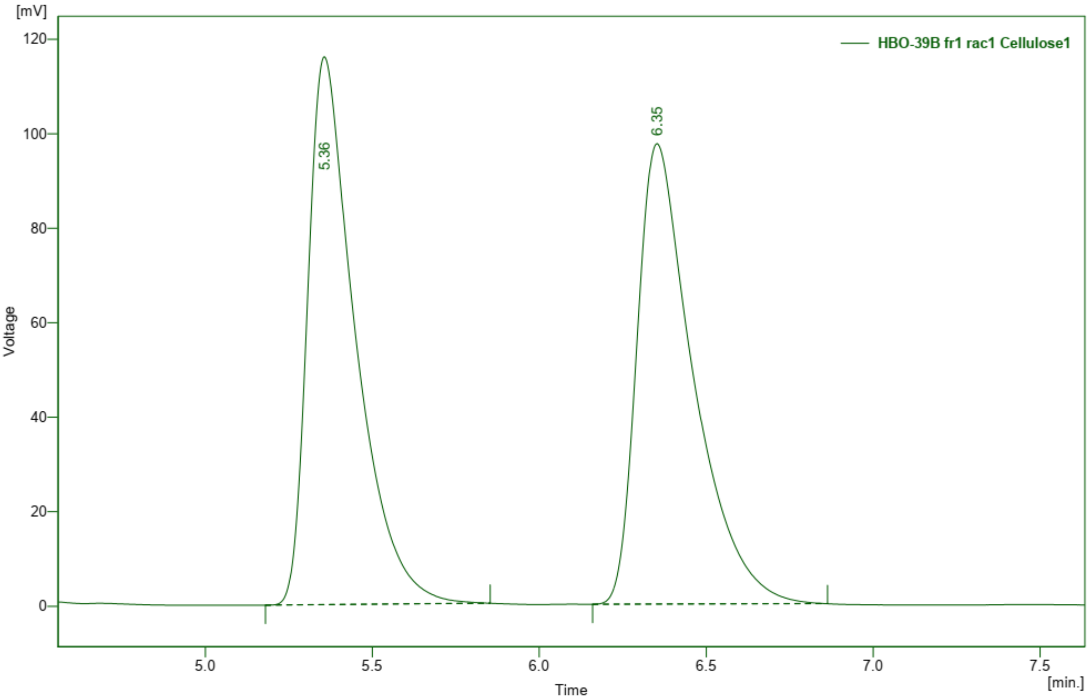

Result Table - Calculation Method Uncal

	Reten. Time [min]	Area [mV.s]	Height [mV]	Area [%]	Height [%]	W05 [min]
1	5.357	1149.062	115.994	50.1	54.3	0.15
2	6.353	1144.859	97.464	49.9	45.7	0.18
	Total	2293.921	213.458	100.0	100.0	

5,6-Dimethyl-4-(2-(methylsulfonyl)phenyl)-2-(quinolin-2-yl)pyridin-3-ol (13)

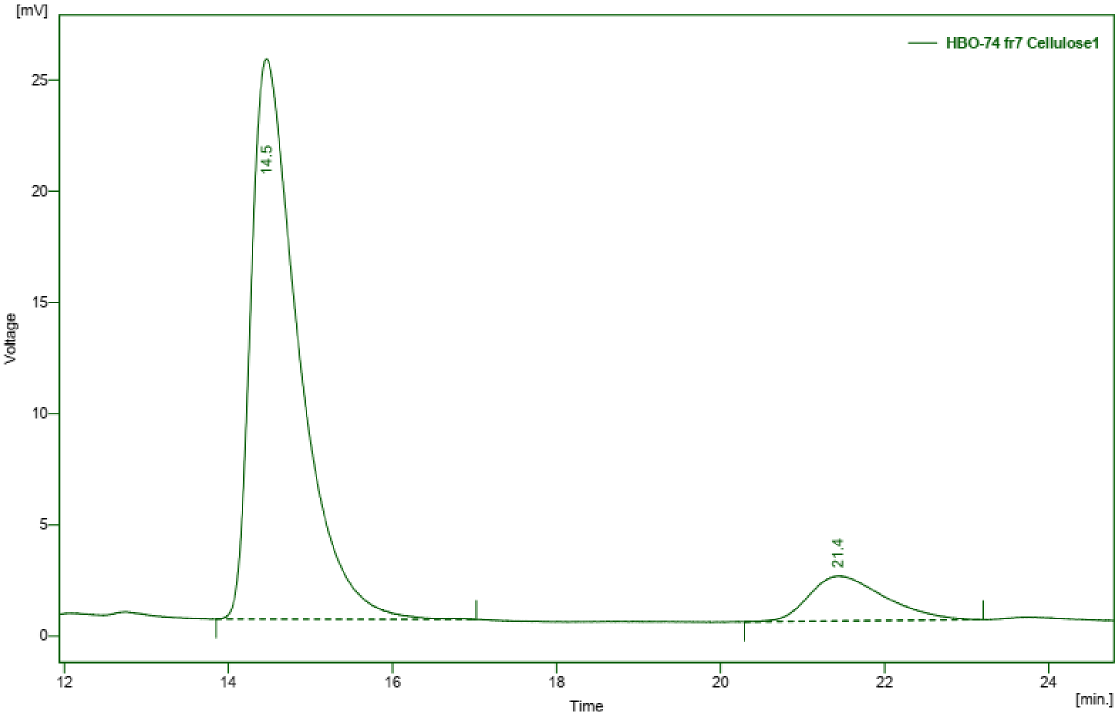

Result Table - Calculation Method Uncal

	Reten. Time [min]	Area [mV.s]	Height [mV]	Area [%]	Height [%]	W05 [min]
1	14.467	1016.023	25.198	88.6	92.5	0.59
2	21.427	130.514	2.031	11.4	7.5	1.01
	Total	1146.537	27.230	100.0	100.0	

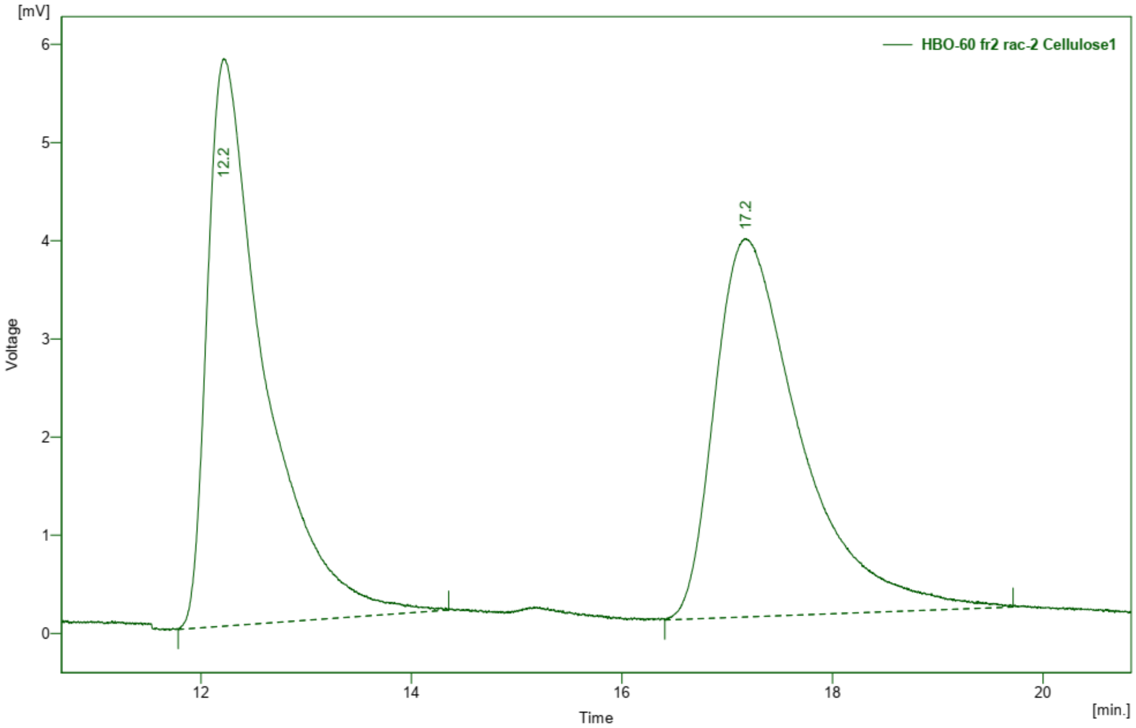

Result Table - Calculation Method Uncal

	Reten. Time [min]	Area [mV.s]	Height [mV]	Area [%]	Height [%]	W05 [min]
1	12.217	219.027	5.776	49.9	60.0	0.51
2	17.173	219.665	3.852	50.1	40.0	0.83
	Total	438.692	9.628	100.0	100.0	

5-Amino-4-(2-(benzyloxy)-3,4-dimethoxy-phenyl)-6-(6-methoxy-5-nitroquinolin-2-yl)-3-methylpicolinonitrile (15)

HPLC analysis

Sample Name LFVP0718

e.e. = 84%, YMC Chiral Art amylose-SA S-5 µm, IPA:hexane (1:1) 1.0 mL/min, 35 °C, λ = 252 nm

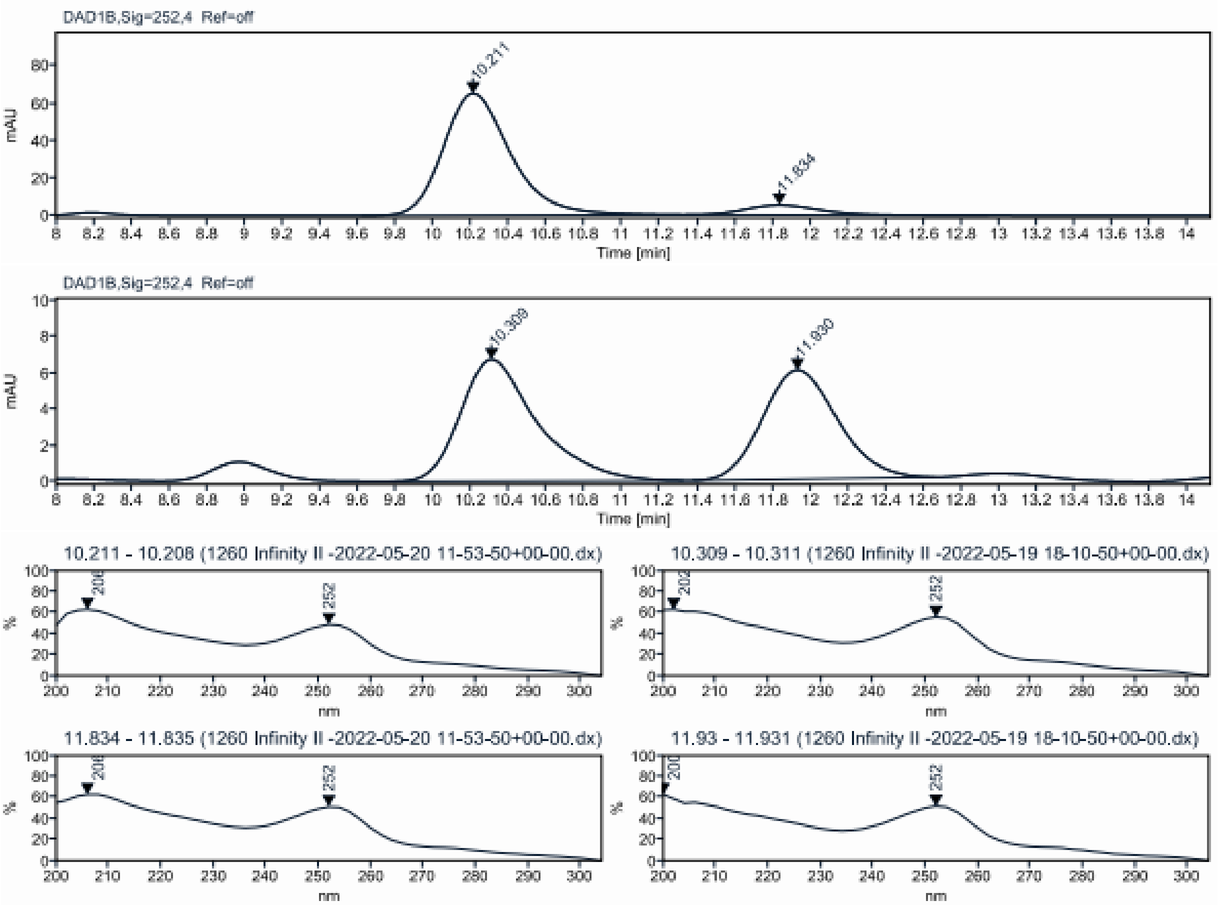

Injection Acquired Date 2022-05-20 11:54:53+00:00

RT [min]	Type	Width [min]	Area	Height	Area%	Name
10.211	BB	1.7089	1620.1507	65.1198	91.7799	
11.834	BB	1.6133	145.1061	5.3003	8.2201	
		Sum	1765.2568			

Injection Acquired Date 2022-05-19 18:28:03+00:00

RT [min]	Type	Width [min]	Area	Height	Area%	Name
10.309	BB	1.5533	190.4602	6.7242	53.3464	
11.930	BB	1.2800	166.5649	6.0211	46.6536	
		Sum	357.0251			

Methyl 5-Amino-4-(2-(benzyloxy)-3,4-dimethoxyphenyl)-6-(6-methoxy-5,8-dioxo-5,8-dihydroquinolin-2-yl)-3-methylpicolinate (17)

HPLC analysis

Sample Name LFVP0724

e.e. = 79%, YMC Chiral Art amylose-SA S-5 μm, IPA:hexane (3:7) 1.25 mL/min, 40 °C, λ = 204 nm

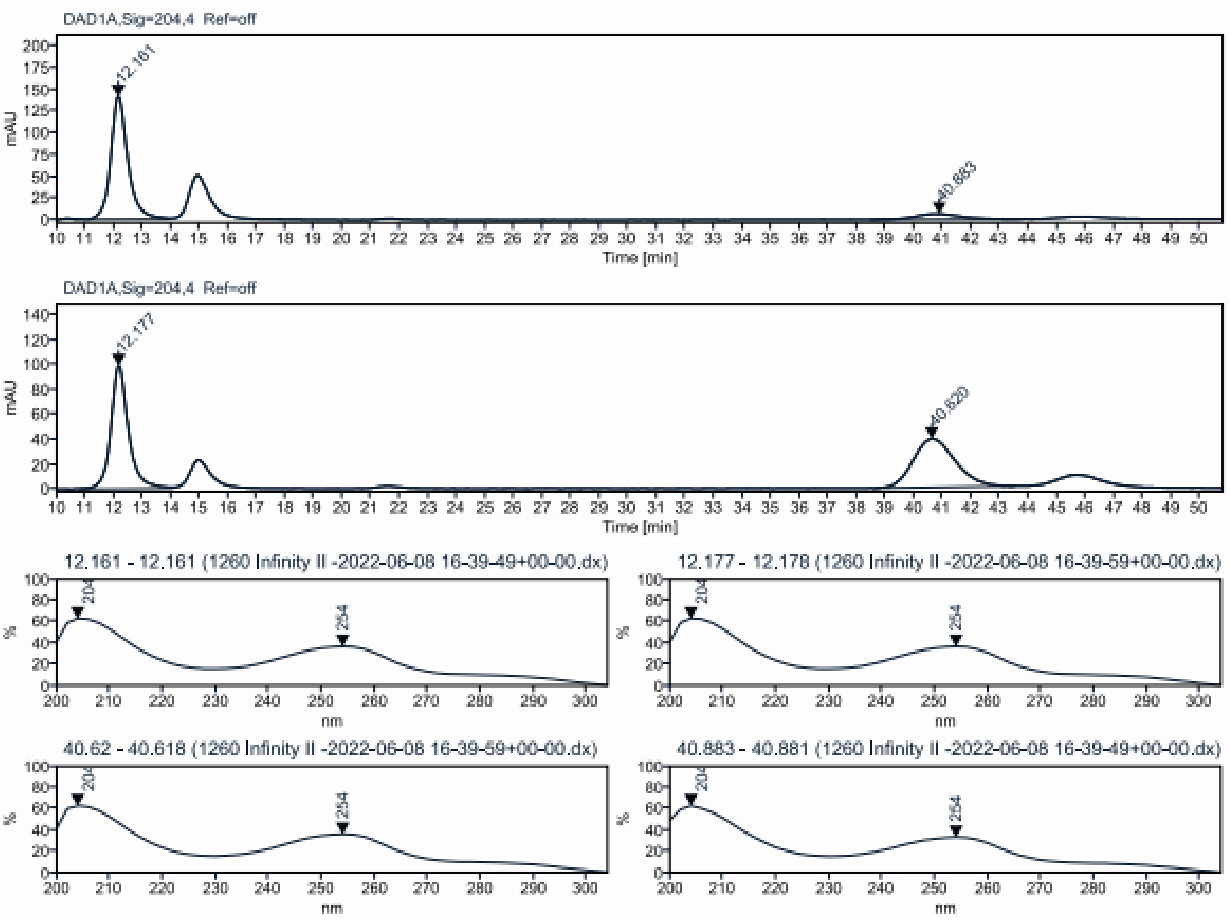

Injection Acquired Date 2022-06-08 17:44:51+00:00

RT [min]	Type	Width [min]	Area	Height	Area%	Name
12.177	VV	3.2496	4261.1478	98.8523	51.6316	
40.620	BM m	1.2083	3991.8414	38.9947	48.3684	
		Sum	8252.9892			

Injection Acquired Date 2022-06-08 16:43:39+00:00

RT [min]	Type	Width [min]	Area	Height	Area%	Name
12.161	BV	2.8726	5917.1509	141.1218	89.7270	
40.883	MM m	1.2398	677.4615	6.4479	10.2730	
		Sum	6594.6123			

Synthetic racemic methyl 5-amino-6-(7-amino-6-methoxy-5,8-dioxo-5,8-dihydroquinolin-2-yl)-4-(2-hydroxy-3,4-dimethoxyphenyl)-3-methylpicolinate (18)

HPLC analysis

Sample Name LFVP0730

e.e. = 0%, YMC Chiral Art amylose-SA S-5 μm, IPA:hexane (4:6) 0.7 mL/min, 40 °C, λ = 204 nm

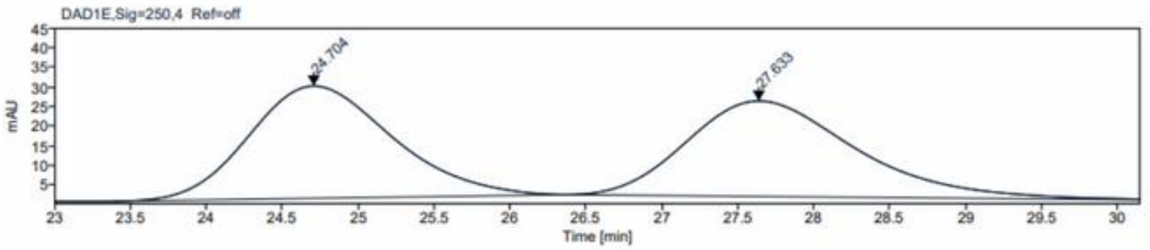

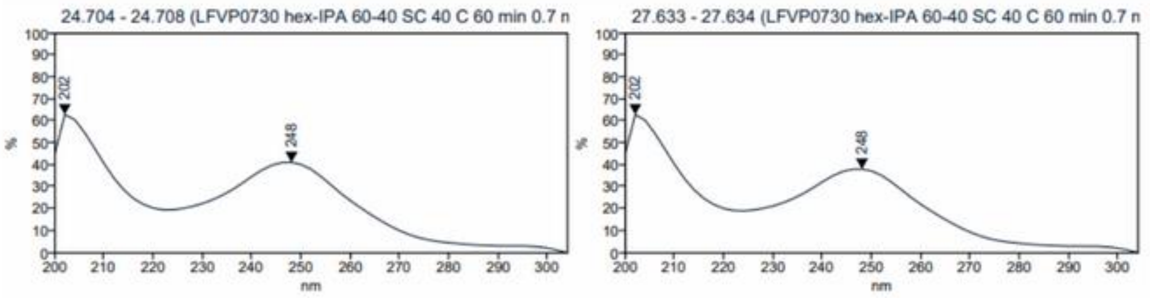

Injection Acquired Date 2022-08-31 14:58:10+00:00

RT [min]	Type	Width [min]	Area	Height	Area%	Name
24.704	BM m	0.9934	1879.4269	28.6479	49.9943	
27.633	BB	4.6400	1879.8572	24.3424	50.0057	
		Sum	3759.2840			

Synthetic enantioenriched (*P*)-methyl 5-amino-6-(7-amino-6-methoxy-5,8-dioxo-5,8-dihydroquinolin-2-yl)-4-(2-hydroxy-3,4-dimethoxyphenyl)-3-methylpicolinate (18)

HPLC analysis

Sample Name LFVP0737

e.e. = 79%, YMC Chiral Art amylose-SA S-5 μm, IPA:hexane (6:4) 0.7 mL/min, 40 °C, λ = 204 nm

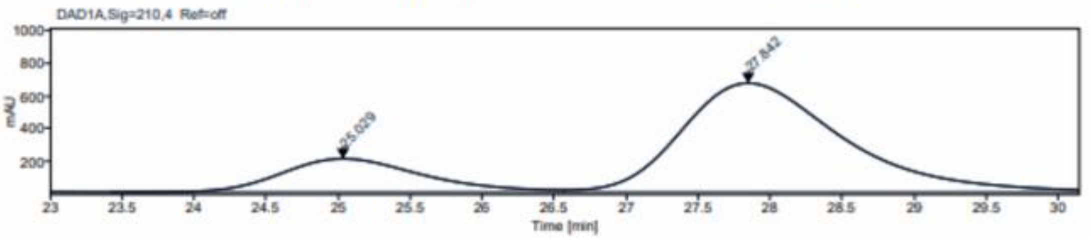

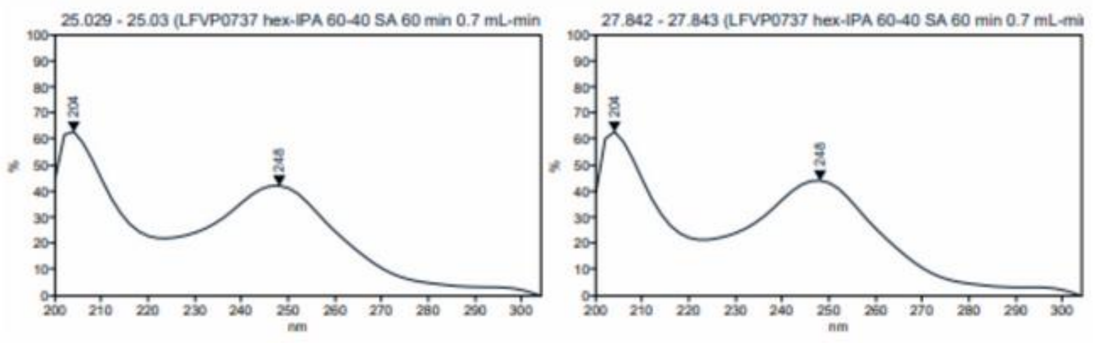

Injection Acquired Date 2022-09-15 19:31:05+00:00

RT [min]	Type	Width [min]	Area	Height	Area%	Name
25.029	BV	3.1464	2158.3560	203.8101	10.8468	
27.842	VB	5.6270	17740.2742	665.1292	89.1532	
		Sum	19898.6302			

(*M*)-Methyl 5-amino-6-(7-amino-6-methoxy-5,8-dioxo-5,8-dihydroquinolin-2-yl)-4-(2-hydroxy-3,4-dimethoxyphenyl)-3-methylpicolinate from natural streptonigrin (18)

HPLC analysis

Sample Name LFVP0736

e.e. = 85%, YMC Chiral Art amylose-SA S-5 μm, IPA:hexane (4:6) 0.7 mL/min, 40 °C, λ = 204 nm

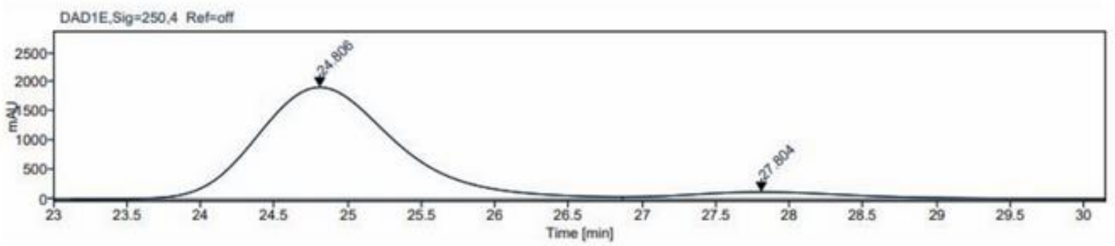

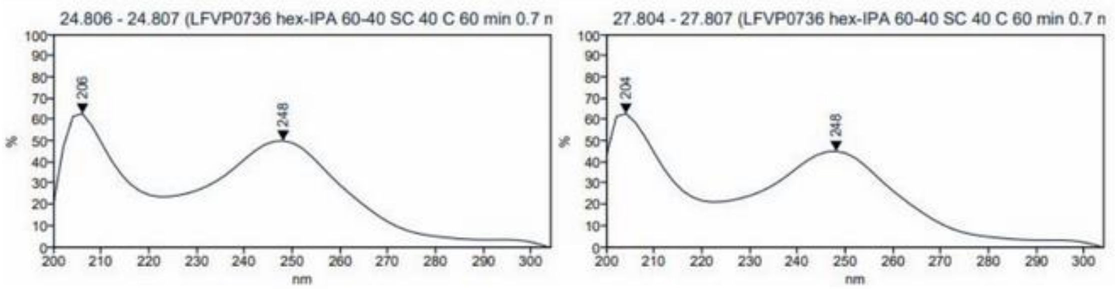

Injection Acquired Date 2022-09-08 15:20:35+00:00

RT [min]	Type	Width [min]	Area	Height	Area%	Name
24.806	BV	3.7468	126234.8388	1912.7525	92.2504	
27.804	VB	7.2932	10604.5543	120.4989	7.7496	
		Sum	136839.3930			

References:

(1) Boger, D. L.; Panek, J. S.; Duff, S. R. Inverse Electron Demand Diels-Alder Reactions of Heterocyclic Azadienes: Formal Total Synthesis of Streptonigrin. *J. Am. Chem. Soc.* **1985**, *107* (20), 5745–5754. https://doi.org/10.1021/ja00306a024.

(2) Donohoe, T. J.; Jones, C. R.; Barbosa, L. C. A. Total Synthesis of (±)-Streptonigrin: De Novo Construction of a Pentasubstituted Pyridine Using Ring-Closing Metathesis. *J. Am. Chem. Soc.* **2011**, *133* (41), 16418–16421. https://doi.org/10.1021/ja207835w.
